# Microbial regulation of soil carbon properties under nitrogen addition and plant inputs removal

**DOI:** 10.7717/peerj.7343

**Published:** 2019-07-17

**Authors:** Ran Wu, Xiaoqin Cheng, Wensong Zhou, Hairong Han

**Affiliations:** Beijing Key Laboratory of Forest Resources and Ecosystem Processes, Beijing Forestry University, Beijing, China

**Keywords:** Soil microorganisms, Regulation, Soil enzyme, Nitrogen addition, Soil carbon properties, Plant inputs removal

## Abstract

**Background:**

Soil microbial communities and their associated enzyme activities play key roles in carbon cycling in terrestrial ecosystems. Soil microbial communities are sensitive to resource availability, but the mechanisms of microbial regulation have not been thoroughly investigated. Here, we tested the mechanistic relationships between microbial responses and multiple interacting resources.

**Methods:**

We examined soil carbon properties, soil microbial community structure and carbon-related functions under nitrogen addition and plant inputs removal (litter removal (NL), root trench and litter removal (NRL)) in a pure *Larix principis-rupprechtii* plantation in northern China.

**Results:**

We found that nitrogen addition affected the soil microbial community structure, and that microbial biomass increased significantly once 100 kg ha^−1^ a^−1^ of nitrogen was added. The interactions between nitrogen addition and plant inputs removal significantly affected soil bacteria and their enzymatic activities (oxidases). The NL treatment enhanced soil microbial biomass under nitrogen addition. We also found that the biomass of gram-negative bacteria and saprotrophic fungi directly affected the soil microbial functions related to carbon turnover. The biomass of gram-negative bacteria and peroxidase activity were key factors controlling soil carbon dynamics. The interactions between nitrogen addition and plant inputs removal strengthened the correlation between the hydrolases and soil carbon.

**Conclusions:**

This study showed that nitrogen addition and plant inputs removal could alter soil enzyme activities and further affect soil carbon turnover via microbial regulation. The increase in soil microbial biomass and the microbial regulation of soil carbon both need to be considered when developing effective sustainable forest management practices for northern China. Moreover, further studies are also needed to exactly understand how the complex interaction between the plant and below-ground processes affects the soil microbial community structure.

## Introduction

In the past century, human activity has altered atmospheric nitrogen exchange in forest ecosystems (Deforest et al., 2004; Galloway, 1998), with global rates of nitrogen deposition having more than doubled (Vitousek et al., 1997). Nitrogen is an important element controlling soil quality, plant diversity and the productivity of forest ecosystems (Sophie, Kerstin & Michael, 2011). Variation in nitrogen may affect soil carbon properties by influencing how soil microbial community structure and its functions drive soil carbon transformation and turnover (Compton et al., 2004). Soil microorganisms act as decomposers and sensitive indicators of soil quality by regulating key process of soil carbon cycling, including lignin and cellulose degradation and soil carbon turnover (Lei et al., 2018; Li et al., 2018; Mele & Crowley, 2008; Sun et al., 2017).

Soil microbial community structure is sensitive to resource availability. Depending on resource availability and supply, soil microbial growth can either be stimulated or inhibited. Nitrogen addition may alter soil microbial community structure and functions in several ways. For example, the growth of soil microorganisms can be stimulated by low-levels of nitrogen addition, because nitrogen-limitation can be ameliorated by improving soil nitrogen availability (Zhu et al., 2016). Similarly, carbon-limitation can be relieved by enhancing aboveground net primary production (NPP) and litter decomposition (Sun et al., 2016; Zhang et al., 2008). De Deyn, Quirk & Bardgett (2011) found that nitrogen addition influenced microbial community structure by directly enhancing soil nitrogen availability, as well as by indirectly affecting soil microbial functions related to carbon turnover. This was accomplished through the stimulation of specific microbial groups that influence the soil carbon process. In contrast, it has been found that nitrogen deposition negatively affects soil microbial growth, composition and function (Zhang, Chen & Ruan, 2018). Overabundance of nitrogen can cause carbon-limitation due to subsequent decreases in litter decomposition rate. This inhibition of litter decomposition is also caused by decreases in phenol oxidase, and by reductions in pH or toxicity by aluminum mobilization (Treseder, 2010). Excessive nitrogen can also elicit production of ligninase from some microorganisms, including white rot fungi (Kundu & Chatterjee, 2006). Therefore, examining of soil microbial responses along a nitrogen addition gradient may help us to understand how nitrogen influences soil carbon processes via the microbial community structure and functions.

The microbial response to shifts in multiple interacting limiting resources is not well understood because few studies have been done (Zhang et al., 2015a; Zhang et al., 2015b). Changes in litter input can help improve soil nutrient availability by alleviating resource stresses (Li et al., 2015a; Li et al., 2015b). Soil carbon processes carried out by soil microbial communities rely on complex interactions with carbon input, which in turn are mediated by nitrogen availability (Bloom, Chapin & Mooney, 1985; Bohlen et al., 2001; Fisk & Fahey, 2001). The response of soil microbes to nitrogen addition is further mediated by carbon input changes due to shifts between carbon-limitation and nitrogen-limitation. Differences in microbial responses may be related to the quality of litter and roots (Liu et al., 2014). Soil microbial functions relating to carbon acquisition under nitrogen addition may also depend on the plant litter and roots (Alster et al., 2013).

A better understanding of how carbon availability specifically affects feedback between plants and microorganisms requires further research on soil microbial community structure and functions relative to different plant inputs removal scenarios. Zhao et al. (2017) conducted a litter removal experiment to study soil microbial community structure and function in a managed pine forest. However, it still remains unclear whether microbial community shifts are a direct result of nitrogen addition, or of the carbon inputs that are mediated by nitrogen addition (Li et al., 2015a). Previous work on nitrogen addition and plant inputs removal have suggested both positive and negative effects on soil microbial community structure and functions (Bachar et al., 2010; Shi et al., 2016; Li et al., 2015b). Therefore, the interaction between nitrogen and changes in carbon input needs to be considered further when analyzing microbial mechanisms under different nitrogen addition scenarios (Georgiou et al., 2017; Zhang et al., 2015a; Zhang et al., 2015b).

Microbial regulation of soil carbon is still not well understood. Ma et al. (2018) has explained the effect of nitrogen addition on soil total organic carbon and active carbon components. However, in this present study, soil carbon properties were used to further analyze the microbial regulation of soil carbon regulatory paths. Studying the direct and indirect effects of both microbial community structure on various functions and microbes on soil carbon properties, is necessary to better understand the belowground processes affecting carbon dynamics. For example, specific microbial groups have certain functions used to drive soil carbon decomposition and turnover (Wang et al., 2017). Likewise, soil enzyme activities are direct expression of soil microbial functions, relating to soil carbon dynamics (Wang et al., 2015). These interactions can be divided into oxidases and hydrolases activities (Bissett et al., 2011; You et al., 2014). Specifically, complex compounds like lignin are degraded by oxidases, which are produced primarily by fungi. Cellulose is degraded by hydrolases, which are produced primarily by bacteria and relate to soil carbon acquisition (You et al., 2014).

The experimental approach of the Detritus Input and Removal Treatment (DIRT) experiments is an efficient method for investigating the correlation between plant and soil microbial communities through litter removal and root exclusion treatments (Veres et al., 2015). Likewise, litter removal and root exclusion regulate soil microclimate by influencing evaporation and absorption of precipitation (Fekete et al., 2016). *Larix principis-rupprechti* is a common plantation tree species in the warm, temperate Taiyue Mountains of Shanxi province. In this study, a litter and root exclusion experiment designed in the method of the DIRT project and the treatments of variable nitrogen addition were established to examine feedback among plant litter, soil nutrient availability and soil microbial communities in short-term treatment in *L. principis-rupprechti* plantations. We measured hydrolase activities to assess soil microbial functions relating to cellulose and chitin degrading capacity. We also assessed oxidase activities to assess microbial functions relating to lignin degradation and their influence on soil carbon transformation. We analyzed how shifts in soil microbial community and functions drive variation in soil carbon properties. Furthermore, we investigated the direct and indirect effects of soil microbial communities on soil enzyme activities, and the direct and indirect effects of microbes on soil carbon properties under various nitrogen addition and plant inputs removal treatments. The objectives of this study were: (1) to assess the responses of soil microbial community structure and functions to nitrogen addition and plant inputs removal; and (2) to explore how nitrogen addition drives linkages among microbial community structure, functions and soil carbon properties under different plant inputs removal treatments. We hypothesized that nitrogen addition would change soil microbial community structure and functions with and without plant inputs removal, and that the soil microbial communities would regulate soil carbon properties, controlled by microbial functions.

## Material and Methods

### Study sites

Study sites were located in *Larix principis-rupprechtii* plantations in the Taiyue Mountain (36°35′–36°53′N, 111°91′–112°04′E) of Shanxi province in northern China. A warm temperate and continental monsoon climate is characteristic of the site. Its altitude ranges from 2,100 to 2,400 m, with an average annual temperature around 8.6 °C. The growing season starts in April and lasts until October, and a rainy season is observed from June to August. Average annual precipitation is nearly 600 mm. The predominant soil type throughout the study sites is Haplic luvisol. The dominant tree species are *Larix principis –rupprechtii* and *Betula platyphylla,* which are typical temperate tree species of northern China. *Lonicera japonica, Corylus mandshurica, Rubus corchorifolius, Rosa xanthina*, and *Lespedeza bicolor* are common shrub species encountered in the region.

### Experimental design and treatments

The experiment began in August 2014 in a 34-year-old *Larix principis-rupprechtii* plantation. Plots were established using a nested, two-factor design. Three replicate plots of two m × two m were established for each treatment. These plots were randomly placed, with a minimum distance of 0.5 m between plots. For the treatment, we used four nitrogen addition levels and three plant inputs removal treatments, which were nested within each nitrogen addition level. The three treatments of plant inputs removal included root trenching and litter removal (NRL), just litter removal (NL) and a control plot (C) where litter and roots were left intact. In the NRL treatment, living roots and aboveground litter were excluded, and trenches were excavated to a depth of 0.5 m. This was done to restrict roots entering the plots from nearby trees. Trenches were embedded with asbestos shingles and then refilled with soil. In the NL treatment, all aboveground inputs were removed. The aboveground litter was removed monthly during the growing season. Natural above- and below-ground litter inputs were left in the C treatment. The nitrogen addition treatments were N0 (0 kg ha^−1^ a^−1^), N1 (50 kg ha^−1^ a^−1^), N2 (100 kg ha^−1^ a^−1^) and N3 (150 kg ha^−1^ a^−1^). NH_4_NO_3_ was used for the nitrogen addition treatments, which were conducted once a month during the growing season from 2014 to 2016.

Soil samples were collected in August 2015 and 2016 at five random points in each plot at a depth of 0–10 cm. A metal corer with an inner diameter of five cm was used to collect samples in the field. The five soil samples from a single plot were mixed to create a composite sample. Soil samples were then stored in sealed bags and immediately taken back to the laboratory for analysis. A two mm sieve was used to remove gravel, roots, and large organic residues from the samples. Samples were then used to measure soil microbial community structure, soil enzyme activities and soil carbon properties. This data was used to assess correlations among the samples.

### Analyses of soil properties

Soil organic carbon (SOC) was analyzed with an elemental analyzer (FLASH 2000). Soil microbial biomass carbon (MBC) was determined via the fumigation-extraction method, and each sample was fumigated for 24 h at 25 °C with alcohol-free CHCl_3_. Dissolved organic carbon (DOC) and MBC were measured using a 0.5 M K_2_SO_4_ extracting agent and measured with a Multi N/C 3100 TOC analyzer (Chen et al., 2017). Soil microbial community structure was determined using phospholipid fatty acid (PLFA) analysis. PLFAs were calculated based on a 19:0 internal standard content. After addition of an internal standard, the phospholipid fraction was subjected to a mild alkaline methanolysis, and the resulting fatty acid methyl esters were separated on a gas chromatograph (Bossio & Scow, 1998). The following soil microbial groups were classified using diagnostic fatty acids as indicators: gram-positive bacteria (GP), gram-negative bacteria (GN), Saprotrophic fungi (Sap), arbuscular mycorrhizal fungi (AMF), and actinomycete (Act) (Table S1). The activities of phenol oxidase (PO) and peroxidase (PER) were determined using DOPA (3, 4- Dihydroxy- L- phenylalamine) as the substrate. Soil suspensions (i.e., one g fresh soil with 1. five mL 50 mmol L^−1^ sodium acetate buffer) and two mL five mmol L^−1^ L-DOPA were mixed for the phenol oxidase assay. The same suspension was used for peroxidase analyses with an addition of 0. two mL 0.3% H_2_O_2_ (Baldrian, 2009). The activities of β-glucosidase (BG), N-acetyl-β-glucosaminidase (NAG) and cellobiohydrolase (CBH) were measured with *p*-nitrophenol assays (You et al., 2014).

### Statistics

Analysis of variance (ANOVA) was used to assess variation in soil carbon, soil microbial communities and soil enzyme activities under various nitrogen addition and plant inputs removal treatments. Statistical analyses were performed using SPSS 19.0.

Redundancy analysis (RDA) was used to assess linkages between soil microbial composition, soil enzyme activity, and soil carbon properties. RDA was used with forward selection to filter the relative importance of individual explanatory variables, and to predict the variation in soil microbial community structure, enzyme activity and soil carbon under nitrogen addition both with and without plant inputs removal. These analyses were completed in CANOCO software for Windows 4.5.

Lastly, structural equation modelling (SEM) was used to test the hypothesis that soil microbial communities affect soil carbon properties by influencing soil enzyme activities through the combined effects of nitrogen addition and plant inputs removal. Path models were established based on existing literature, where bacteria and Sap represent the structural attributes of soil microbial communities and oxidases, and BG represents the soil microbial function driving carbon acquisition and oxidation (You et al., 2014). We combined the data from all treatments and estimated the model parameters using a maximum likelihood estimation in the Amos 22.0 software package. The adequacy of model fit was assessed by a *χ*^2^ test (*p* > 0.05, CMIN/df < 2), comparative fit index (CFI > 0.90) and by the root square mean error of approximation (RMSEA < 0.05) (You et al., 2014). Numbers on arrows are standardized direct path coefficients. *R*^2^ values represent the proportion of total variance explained for a specific dependent variable. Dash-line arrows indicate negative effects.

## Results

### Variation in soil microbial biomass under nitrogen addition

**Figure 1:**
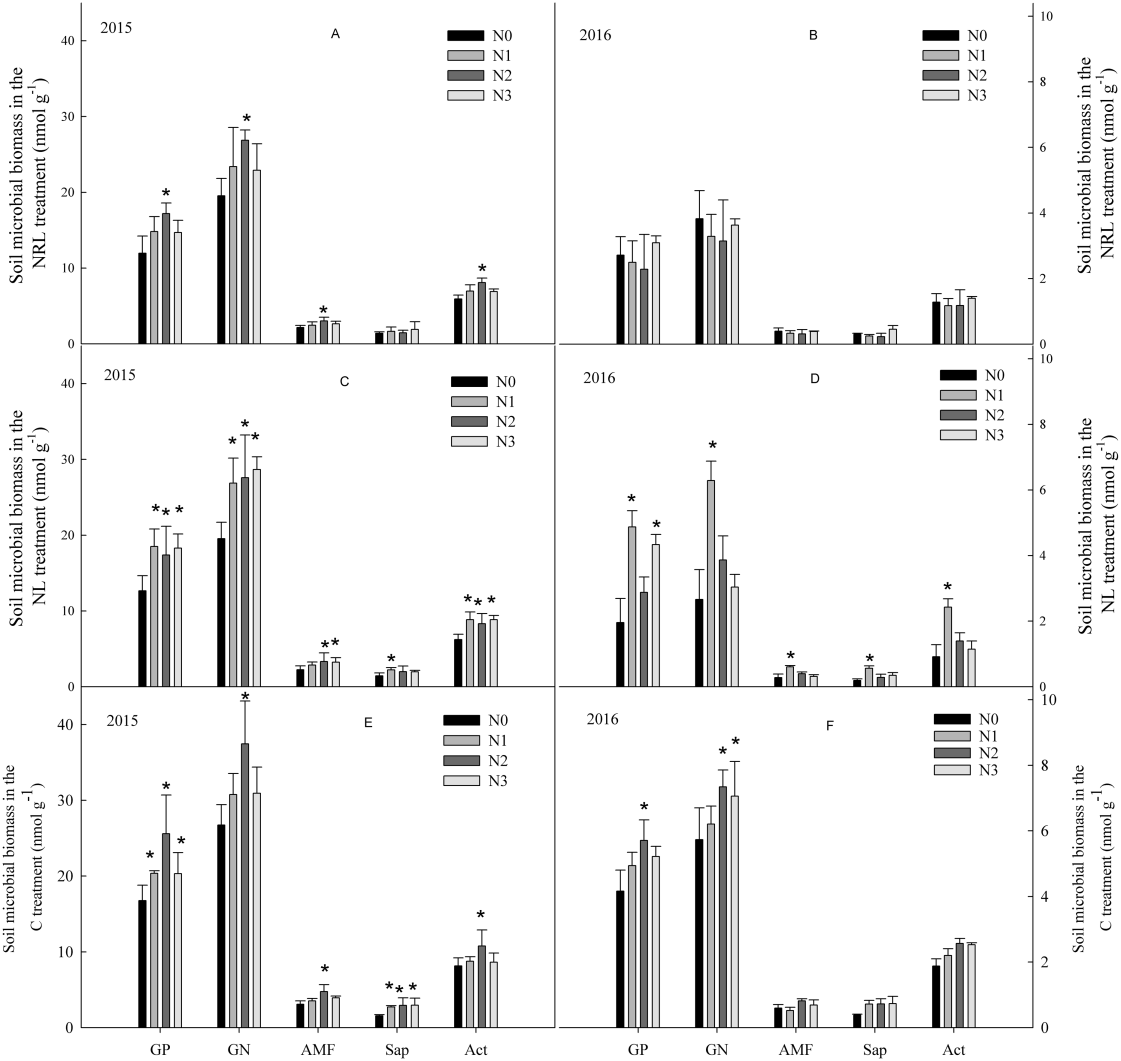
Soil microbial biomass under nitrogen addition in the NRL (A–B), NL (C–D) and C (E–F) treatments. GP, Gram-positive bacteria; GN, Gram-negative bacteria; AMF, arbuscular mycorrhizal fungi; Sap, Saprotrophic fungi; Act, actinomycete; NRL, root trench and remove litter; NL, litter removal; C, the control plot. N0, nitrogen addition at 0 kg ha^−1^ a^−1^; N1, nitrogen addition at 50 kg ha^−1^ a^−1^; N2, nitrogen addition at 100 kg ha^−1^ a^−1^; N3, nitrogen addition at 150 kg ha^−1^ a^−1^. * indicates that the microbial biomass differs significantly from the N0 treatment at each nitrogen level (*p* < 0.05).

In the C treatment, soil microbial biomass first increased, and then decreased with an increase in nitrogen addition level (Fig. 1). In the NL treatment, nitrogen addition enhanced soil microbial biomass. The increase in soil microbial biomass was significantly higher in the N1 level treatment than at other nitrogen levels in 2016. In the NRL treatment, soil microbial biomass increased under nitrogen addition in the first year, but decreased under the same treatment in 2016.

Soil microbial biomass was significantly influenced by sampling year (Table 1). There were also significant interactive effects between plant inputs removal and sampling year on the biomass of GP, GN and Act. Interactive effects were also found between nitrogen addition and plant inputs removal on the biomass of GP and GN, though the effects differed with year.

**Table 1:** Result (*F* value) of a three—way ANOVA on the effects of nitrogen addition, plant inputs removal and year on soil microbial biomass.

Treatments	GP	GN	AMF	Sap	Act
N	7.673^***^	1.863	6.644^**^	8.020^***^	1.117
L	34.228^***^	6.409^**^	5.641^**^	4.524^*^	1.643
Y	225.004^***^	1144.864^***^	511.431^***^	15.818^***^	171.889^***^
N * Y	1.365	0.117	0.606	0.975	3.473^*^
L * Y	10.023^***^	10.483^***^	0.046	0.442	8.476^**^
N * L	3.027^*^	2.612^*^	1.131	2.237	3.030*
N * L * Y	2.814^*^	3.151^*^	1.339	0.690	1.705

**Notes.**

GP Gram-positive bacteria GN Gram-negative bacteria AMF arbuscular mycorrhizal fungi Sap Saprotrophic fungi Act actinomycete N nitrogen addition L plant inputs removal Y year

Each of these variables were fitted in following statistical model, e.g., four levels of nitrogen addition * three plant inputs treatments * two years.

* *p* < 0.05.

** *p* < 0.01.

*** *p* < 0.001.

### Variation in soil enzyme activity under nitrogen addition

**Figure 2:**
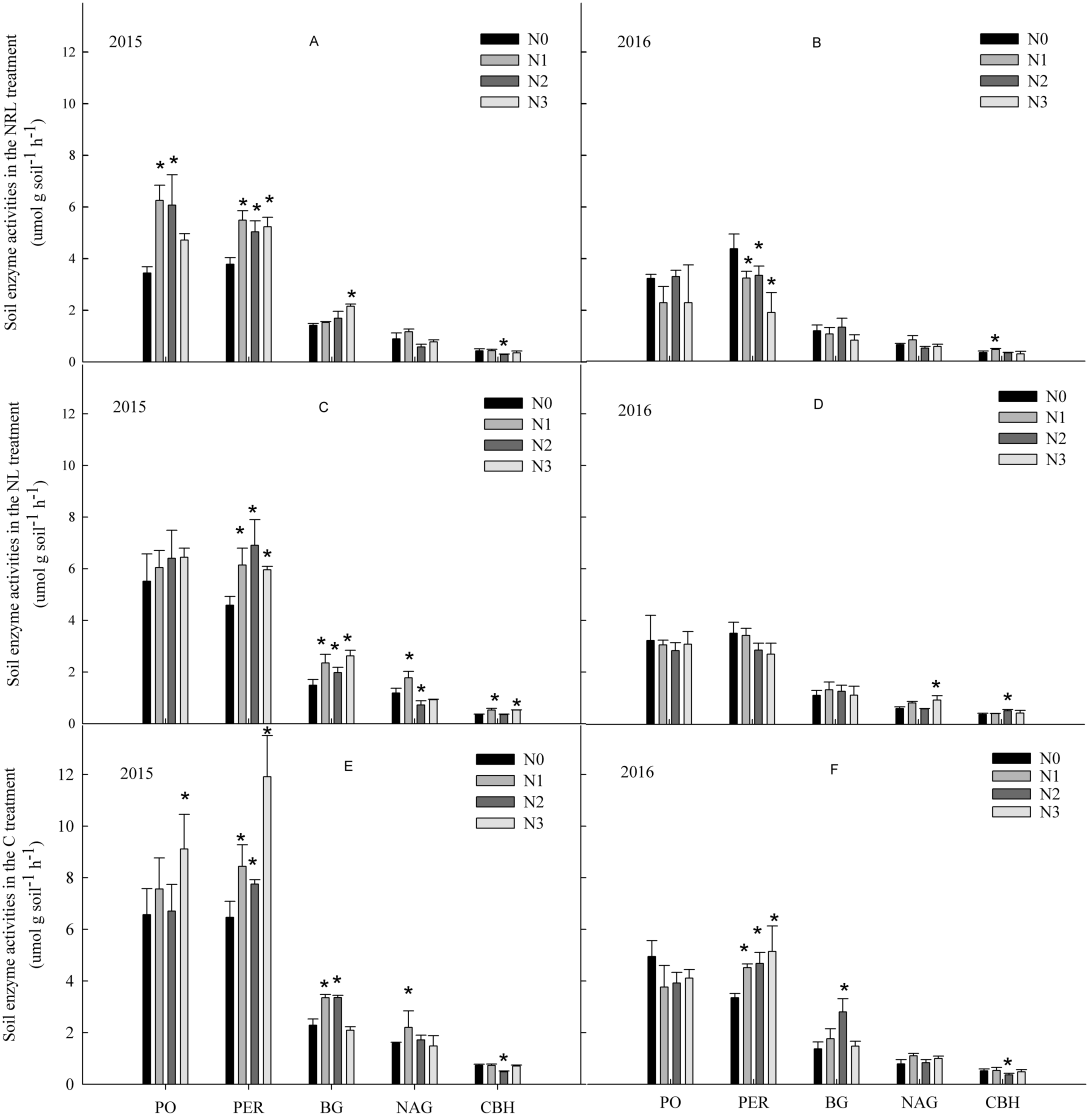
Soil enzyme activities under nitrogen addition in the NRL (A–B), NL (C-D) and C (E-F) treatments. PO, phenol oxidase; PER, peroxidase; BG, β-glucosidase; NAG, N-acetyl-β-glucosidase; CBH, cellobiohydrolase; NRL, root trench and remove litter; NL, litter removal; C, the control plot. N0, nitrogen addition at 0 kg ha^−1^ a^−1^; N1, nitrogen addition at 50 kg ha^−1^ a^−1^; N2, nitrogen addition at 100 kg ha^−1^ a^−1^; N3, nitrogen addition at 150 kg ha^−1^ a^−1^. * indicates that soil enzyme activities differs significantly from the N0 treatment at each nitrogen level (*p* < 0.05).

In the C treatment in 2015, nitrogen addition enhanced the activities of PO, PER, BG and NAG (Fig. 2). In contrast, the activity of PO had a decreased trend in 2016. In the NL and NRL treatment, PO and PER activities increased under nitrogen addition in 2015, but they decreased in 2016. Moreover, the N1 level treatment enhanced the activity of NAG in the C and NL treatments.

Nitrogen addition, plant inputs removal, and sampling year all significantly affected soil enzyme activities (Table 2). The effect of plant inputs removal or nitrogen addition on the activities of PER, NAG and CBH were also dependent on year. There were interactive effects between nitrogen addition and plant inputs removal on the activities of PER and BG in all years.

**Table 2:** Result (*F* value) of a three—way ANOVA on the effects of nitrogen addition, plant inputs removal and year on soil enzyme activities.

Treatments	PO	PER	BG	NAG	CBH
N	1.066	10.504^***^	16.875^***^	20.183^***^	13.420^***^
L	33.173^***^	111.121^***^	79.646^***^	54.570^***^	72.586^***^
Y	215.231^***^	382.211^***^	165.531^***^	97.786^***^	22.437^***^
N * Y	6.979^**^	19.828^***^	6.987^**^	6.723^**^	6.003^**^
L * Y	1.944	25.067^***^	2.482	13.460^***^	16.278^***^
N * L	2.723^*^	14.129^***^	12.178^***^	1.189	6.613^***^
N * L * Y	1.699	3.936^**^	4.254^**^	1.592	1.922

**Notes.**

PO phenol oxidase PER peroxidase BG β-glucosidase NAG N-acetyl-β-glucosidase CBH cellobiohydrolase N nitrogen addition L plant inputs removal Y year

Each of these variables were fitted in following statistical model, e.g., four levels of nitrogen addition * three plant inputs treatments * two years.

* *p* < 0.05.

** *p* < 0.01.

*** *p* < 0.001.

### Variation in soil carbon properties under nitrogen addition

In the C treatment in 2015, the N1 and N2 level treatments lowered SOC and DOC. However, there was no significant variation in SOC and DOC in the N1 and N2 level treatments in 2016 (Table 3). In the NL treatment in 2015, SOC and MBC first increased and then declined with nitrogen addition. DOC declined gradually with nitrogen addition in 2015. In the NRL treatment in 2015, SOC had a trend of decrease at the N3 levels. At the N2 level treatment, MBC was significantly higher than at the other nitrogen levels in 2015, but the opposite was true in 2016. DOC had a trend of decrease under nitrogen addition in the NRL treatment during both sampling years.

**Table 3:** The variation in soil carbon properties under nitrogen addition in the NRL, NL and C treatments.

Soil carbon	Plant inputs removal	Years	The levels of nitrogen addition			
			N0	N1	N2	N3
SOC (g kg^−1^)	NRL	2015	53.45 ± 7.42a	55.27 ± 13.37a	52.7 ± 3.52a	40.13 ± 6.1a
	NL		42.6 ± 6.42b	65.6 ± 8.43a	46.7 ± 2.85b	40.65 ± 0.35b
	C		51.23 ± 7.57a	44.37 ± 3.72ab	39.97 ± 1.01b	37.7 ± 6.01b
	NRL	2016	41.63 ± 3.43a	37.85 ± 2.51a	37.7 ± 1.73a	41.19 ± 2.23a
	NL		42.36 ± 1.91a	45.13 ± 4.4a	40.71 ± 1.37a	39.96 ± 3.16a
	C		45.04 ± 4.67ab	50.57 ± 0.94a	49.14 ± 1.71a	43.33 ± 3.5b
MBC (mg kg^−1^)	NRL	2015	1490.08 ± 49.36c	1115.13 ± 64.48d	2022.12 ± 26.69a	1675.74 ± 45.3b
	NL		1287.53 ± 19.1d	2166.46 ± 27.79a	1798.6 ± 46.93b	1581.09 ± 124.84c
	C		1492.85 ± 33.98b	1474.8 ± 38.83b	1423.57 ± 83.89b	1671.82 ± 48.68a
	NRL	2016	902.13 ± 81.86a	885.61 ± 82.73a	692.32 ± 306.33a	887.68 ± 49.31a
	NL		1022.38 ± 152.33a	822.7 ± 157.68a	985.09 ± 51.54a	920.88 ± 10.91a
	C		1064.01 ± 124.2a	934.6 ± 52.31a	1031.53 ± 159.29a	988.17 ± 28.51a
DOC (mg kg^−1^)	NRL	2015	138.25 ± 25.84a	133.39 ± 12.51a	135.07 ± 0.07a	126.25 ± 2.42a
	NL		140.02 ± 2.94a	132.52 ± 16.28ab	125.19 ± 4.76ab	115.01 ± 6.6b
	C		193.9 ± 3.74a	165.3 ± 6.93b	159.63 ± 23.77b	181.5 ± 2.42ab
	NRL	2016	173.5 ± 8.24a	161.59 ± 10.26a	154.72 ± 10.75b	158.67 ± 10.02ab
	NL		165.02 ± 7.88a	159.19 ± 7.31a	159.93 ± 10.39a	162.78 ± 15.69a
	C		179.92 ± 6.89a	179.51 ± 16.25a	191.83 ± 9.14a	173.69 ± 13.21a

**Notes.**

Different letters indicate significant differences under different nitrogen addition (*p* < 0.05). Values are mean ± standard errors (*n* = 3).

SOC soil organic carbon MBC soil microbial biomass carbon DOC dissolved organic carbon NRL root trench and remove litter NL litter removal C the control plot N0 zero rate of nitrogen addition N1 nitrogen addition at 50 kg ha^−1^ a^−1^ N2 nitrogen addition at 100 kg ha^−1^ a^−1^ N3 nitrogen addition at 150 kg ha^−1^ a^−1^

### Microbial regulatory pathways on soil enzymes under nitrogen addition

The pathway showed that GN and Sap had a direct and indirect effect on soil enzyme activities under nitrogen addition (Table 4). The biomass of GN had relatively strong total effects on the soil enzyme activities. For the C treatment, the biomass of GN had a direct and positive effect on the activities of PER and BG, while Sap had a direct effect on the activity of PER. In the NL and NRL treatments, the activities of PER and BG were directly affected by the biomass of GN. Compared to the total effects of plant inputs removal, we found that the total effect of GN augmented the activity of PER in the NL and NRL treatments, while the total effect of Sap reduced the activity of PER. The total effects of GN and Sap increased the activity of BG in the NL and NRL treatments.

**Table 4:** Path coefficients of the effects of soil microbes on soil enzyme activities under nitrogen addition in the NRL, NL and C treatments.

Factors	Effect	Soil enzyme	
		PER	BG
**In the NRL treatment**			
GN	Direct	0.716	0.733
	Indirect	0	0
	Total	0.716	0.733
Sap	Direct	0	0
	Indirect	0.204	0.210
	Total	0.204	0.210
**In the NL treatment**			
GN	Direct	0.844	0.749	
	Indirect	0	0	
	Total	0.844	0.749	
Sap	Direct	0	0	
	Indirect	0.384	0.341	
	Total	0.384	0.341	
**In the C treatment**			
GN	Direct	0.635	0.526	
	Indirect	0.091	0	
	Total	0.726	0.526	
Sap	Direct	0.329	0	
	Indirect	0.177	0.146	
	Total	0.506	0.146	

**Notes.**

PER peroxidase BG β-glucosidase GN Gram-negative bacteria Sap Saprotrophic fungi NRL root trench and remove litter NL litter removal C the control plot

### Soil enzyme regulatory pathways on soil carbon properties under nitrogen addition

The pathway showed that soil enzymes had both direct and indirect effects on soil carbon under nitrogen addition (Table 5). The activity of PER had relatively strong total effects on soil carbon. In the C treatment, SOC was directly and negatively affected by the activity of PER, while MBC was directly and positively influenced by PER activity. There was no direct effect of BG activity on soil carbon properties. In the NL treatment, the activities of PER and BG directly affected MBC, but only PER directly affected SOC. In the NRL treatment, MBC was directly affected by BG activity. However, PER and BG did not directly influence SOC. Compared to the total effects under nitrogen addition and plant inputs removal, we found that the total effect of PER reduced MBC and SOC in the NRL treatments. The total effect of BG augmented MBC and SOC in the NL treatments.

**Table 5:** Path coefficients of the effects of soil enzyme activities on soil carbon under nitrogen addition in the NRL, NL and C treatments.

Factors	Effect	Soil carbon	
		MBC	SOC	
**In the NRL treatment**			
PER	Direct	0	0	
	Indirect	0.437	0	
	Total	0.437	0	
BG	Direct	0.691	0	
	Indirect	0	0	
	Total	0.691	0	
**In the NL treatment**			
PER	Direct	0.556	0.479	
	Indirect	0.305	0	
	Total	0.861	0.479	
BG	Direct	0.399	0	
	Indirect	0.425	0.366	
	Total	0.824	0.366	
**In the C treatment**			
PER	Direct	0.825	−0.495	
	Indirect	0	0	
	Total	0.825	−0.495	
BG	Direct	0	0	
	Indirect	0.305	−0.183	
	Total	0.305	−0.183	

**Notes.**

PER peroxidase BG β-glucosidase MBC soil microbial biomass carbon SOC soil organic carbon NRL root trench and remove litter NL litter removal C the control plot

### Regulation of soil carbon by the soil microbial communities and enzymes under nitrogen addition

**Figure 3:**
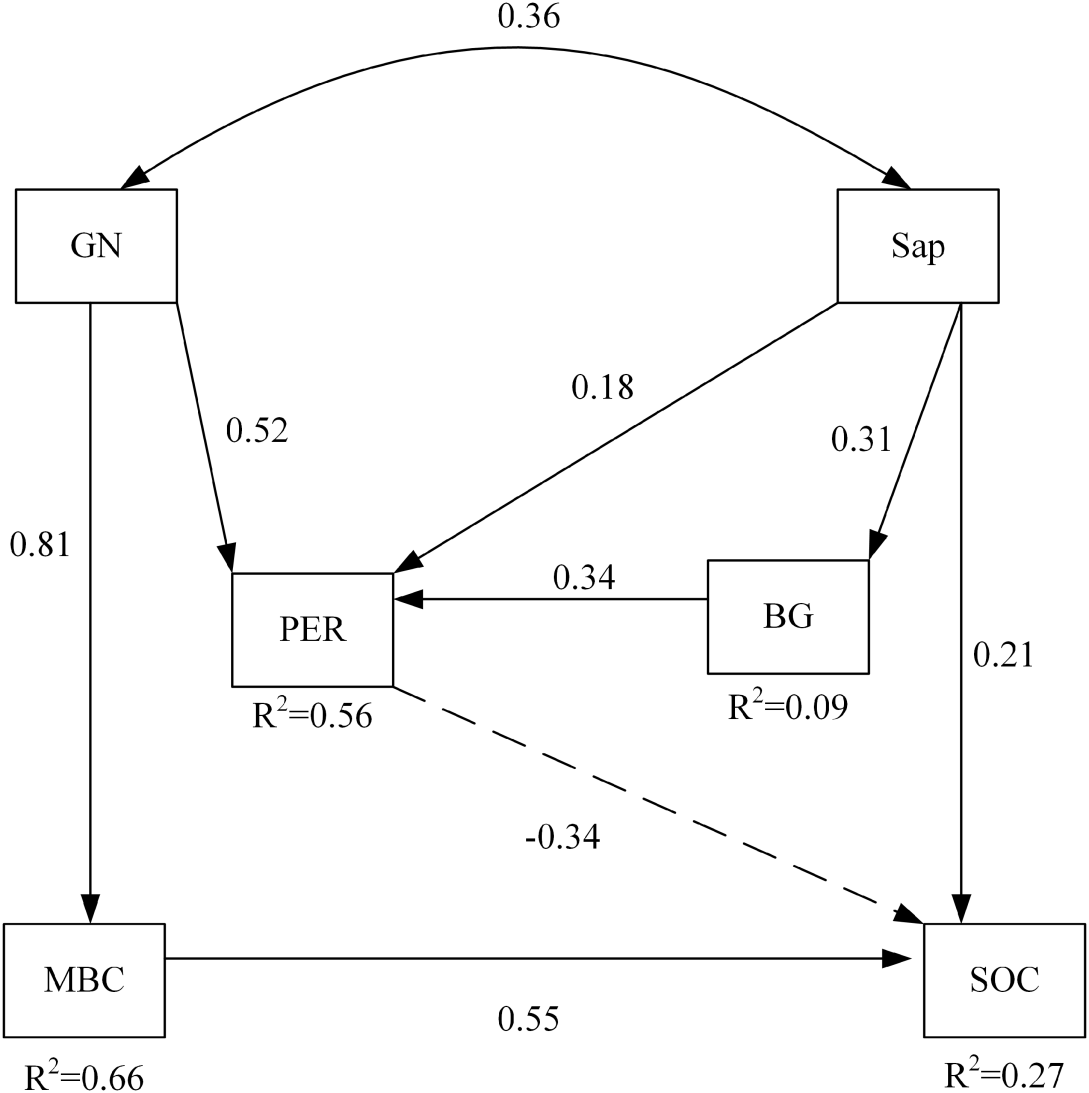
The structural equation model depicting the regulation of soil carbon by enzyme activities and the soil microbial community under the combined effects of nitrogen addition and plant inputs removal. Numbers on arrows are standardized direct path coefficients. *R*^2^ value represents the proportion of total variance explained for the specific dependent variable. Dash-line arrows indicate negative effects. PER, peroxidase; BG, β-glucosidase; GN, Gram-negative bacteria; Sap, Saprotrophic fungi. SOC, soil organic carbon; MBC, soil microbial biomass carbon.

Among soil microbial community and soil enzyme variables, RDA ordination indicated that the biomass of GN and the activity of PER significantly affected soil carbon properties under nitrogen addition (Table 6). The path of the variables under the interaction between nitrogen addition and plant inputs removal passed the statistical test for adequacy (*χ*^2^ = 4.468, *p* = 0.614, CNMI/df = 0.745, CFI = 1.000, GFI = 0.979, RMSEA <0.001) and the non-significant pathways were deleted (Fig. 3). The model explained 56% and 9% of the variance in the activities of PER and BG, respectively. Likewise, 66% and 27% of the variance in MBC and SOC were explained by this model. The path analysis indicated that GN and Sap regulated soil oxidases, hydrolases, and soil carbon properties under nitrogen addition and plant inputs removal. SOC was negatively and directly affected by PER activity, which was positively influenced by GN and Sap. GN and Sap directly and positively influenced soil carbon.

**Table 6:** Marginal and conditional effects of soil microbial composition and soil enzyme activities on soil carbon obtained from forward selection in Redundancy analysis (RDA) under nitrogen addition.

Variables	Lambda-A^a^	Lambda-B^b^	*p*^c^	F-ratio^d^
GN	0.515	0.515	**0.002**	72.223
PER	0.234	0.028	**0.020**	4.082

**Notes.**

a Describe marginal effects, which show the variance explained when the variable is used as the only factor.

b Describe conditional effects, which show the additional variance each variable explains when it is included in the model.

c The level of significance corresponding to Lambda-B when performing Monte Carlo test at the 0.05 significance level.

d The Monte Carlo test statistics corresponding to Lambda-B at the 0.05 significance level.

PER peroxidase GN Gram-negative bacteria

## Discussion

### The responses of soil microbial communities and functions to nitrogen addition and plant inputs removal

An important finding in our study include that soil microbial biomass increased under nitrogen addition and that microbial biomass was significantly higher in the N2 level treatment than the other levels. This result is consistent with previous observations showing increases in soil bacterial biomass and diversity after nitrogen addition (Nguyen et al., 2018), as well as increases in bacterial biomass in a lower-elevation forest and in fungal biomass in an upper-elevation forest after nitrogen addition (Cusack et al., 2011). This result shows that the N1 treatment enhanced the activity of NAG, which degrades chitin. This suggests that microbial turnover occurs more rapidly at the N1 level treatment, possibly because microbial cell walls consist of fungal chitin and bacterial peptidoglycan. Our findings also suggest that soil oxidase activities increased after the first year of nitrogen addition, and then decreased after the second year. Similar results were also observed in an Alaskan boreal forest, where carbon-degrading enzyme activities increased after short-term nitrogen addition (Allison, Czimczik & Treseder, 2008). However, declines in soil bacterial biomass have also been reported after three years of nitrogen addition in a secondary tropical forest of China and one year of nitrogen addition in North America (Li et al., 2015a; Li et al., 2015b; Ramirez, Craine & Fierer, 2012). It has also been found that long-term nitrogen addition reduces soil fungi via nitrogen saturation in temperate ecosystems (Demoling, Nilsson & Bååth, 2008). Our results show that nitrogen addition reduced SOC and DOC, and increased soil microbial biomass in the first year of treatment. It has been suggested that soil microbial biomass correlates negatively with SOC under nitrogen addition. This result was supported by the literature showing a negative relationship between soil bacterial biomass and SOC over three years of nitrogen addition in a temperate needle-broadleaved forest (Cheng, Fang & Yu, 2017). Similar results were reported by other studies that found declines in soil organic carbon under nitrogen addition in grasslands (Moinet et al., 2016).

It has been theorized that changes in soil organic matter may result from variation in soil nutrient availability following plant inputs removal Lietal2015. Our results show that nitrogen addition lowered soil microbial biomass and soil oxidases activities in the NRL treatment in 2016. The response of soil microbial communities to nitrogen addition did not align with the response of communities to the plant inputs removal. It has been indicated that plant inputs removal could potentially alter nutrient limitation under nitrogen addition (Sayer et al., 2012). Likewise, after the first year of root trenching and litter removal, soil microbial biomass and oxidase activities increased under nitrogen addition because they had been previously nitrogen-limited. However, these metrics declined in the second year due to a shift from nitrogen-limitation to carbon-limitation (Averill & Waring, 2018; Traoré et al., 2016). Microbial biomass was much higher at the N1 level than at other nitrogen addition levels in the second year of litter removal. This may have been because litter removal caused nitrogen-limitation, and nitrogen addition positively affected soil microbes at the N1 level. We also found that SOC and DOC decreased with nitrogen addition in the NRL treatments due to the shift in nutrient limitation. Our result is inconsistent with previous work showing that soil organic matter decomposition does not differ under nitrogen addition between root exclusion and natural states. This is because the presence of roots mediates the response of soil organic carbon decomposition to nitrogen addition (Moinet et al., 2016).

Plant inputs removal altered soil bacterial and fungal communities, which aligned with previous literature (Brant, Myrold & Sulzman, 2006; Kramer et al., 2010). These studies theorized that root-derived carbon was the predominant source of carbon for microbes. However, this theory was not supported by a study in a mixed-wood forest of northern China, where microbes and soil enzyme activities were not significantly affected by the interaction between nitrogen addition and plant inputs removal (Sun et al., 2016). As resources for fungal decomposers, nitrogen, litter and roots were the main factors influencing AMF and Sap. Meanwhile, bacteria mediated soil carbon processes via the combined effects of nitrogen addition and plant inputs removal. This was inconsistent with results from *M. laosensis* soils, where root exclusion did not change the biomass of AMF (Wan et al., 2015). Our results show that the biomass of GP was significantly influenced by nitrogen addition and plant inputs removal, while the biomass of GN was significantly affected by plant inputs removal and not by nitrogen addition. It is likely that GN first utilizes recent plant material with a lower C/N ratio as its carbon source, while GP tends to utilize more recalcitrant carbon first (Kramer & Gleixner, 2006).

### Nitrogen addition drives linkage between soil microbial community structure and soil carbon in different plant inputs removal

It has been theorized that the availability of nitrogen can modulate the microbial structure-function relationship (Nguyen et al., 2018). Here, we found that nitrogen addition lead to the direct and positive effect of GN on oxidases and hydrolases. Meanwhile, fungi (Sap) did not directly affect hydrolases. This result was also observed in the literature, where soil microbial community structure was not correlated with hydrolases under land-use change conditions (Tischer, Blagodatskaya & Hamer, 2015). GN had a strong total effect on soil oxidases and hydrolases under nitrogen addition, while Sap had little effect. This result was inconsistent with previous studies that suggest that soil enzyme activities are not always related to soil microbial community structure, such as those in the area of P-limitation (Tischer, Blagodatskaya & Hamer, 2014). After plant inputs removal lowered the carbon input, the direct effects of bacteria and fungi varied under nitrogen addition. However, the GN in each plant inputs removal directly affected the oxidase activities. It has been theorized that substrate complexity leads to the connection between soil microbial community and function (Baldrian, 2009). We also found that the total effect of GN increased PER activity and that the effect of Sap decreased PER activity in both the NL and NRL treatments. It has been suggested that decreases in carbon input weaken the effects of oxidases on complex carbon sources, while the carbon decreases enhance the effects of hydrolases on easily-decomposed carbon sources. This idea has been supported by previous studies showing that litter input increases lignin oxidase (Sun et al., 2016). Meanwhile, GN grew quickly to compete for soil organic compounds after altering carbon and nitrogen resources. This was consistent with previous studies (Bell et al., 2009; Waldrop, Balser & Firestone, 2000) that found that GN was correlated with hydrolases that degrade simple organic compounds.

Presently, understanding the linkages between soil enzyme activities and soil carbon properties is necessary for predicting shifts in soil microbial community and functions (Weand et al., 2010). Previous work has reported that soil enzyme activities are significantly correlated to SOC and MBC (Wang et al., 2013), which was consistent with our result that PER and BG activities directly and indirectly affected MBC and SOC under nitrogen addition with and without plant inputs removal. After the plant inputs removal, the direct effect on MBC and SOC changed. This was likely due to the functional shift from degrading complex to simple carbon resources. These results show that soil oxidases greatly influenced soil carbon properties under nitrogen addition. However, plant inputs removal enhanced the total effect of BG on soil carbon properties, which suggests that carbon acquisition was enhanced. Our finding is consistent with previous studies theorizing that nitrogen addition enhances linkages between oxidases and the loss of aliphatic carbon, which are related to decreases in soil carbon storage (Cusack et al., 2011). Meanwhile, the interaction between nitrogen addition and plant inputs removal converted poorer-quality soil (i.e., high lignin and low N) to higher-quality soil (i.e., low lignin and high N), which increased microbial substrate utilization and strengthened the correlation between the hydrolases and soil carbon properties (Ostertag et al., 2008; Gallo et al., 2005).

### The regulation of soil carbon properties under treatments of nitrogen addition with plant inputs removal

Research on the response of soil microbial community structure to ecosystem function has been growing rapidly (Lewis et al., 2010; Malchair et al., 2010). An increasing number of studies have been published in recent years on the linkages between soil microbial community structure, functions and soil carbon dynamics (Cusack et al., 2011; Ren et al., 2018; Sun et al., 2017; Veres et al., 2015). Variation in soil microbial communities is likely to lead to microbial function shifts. Specific soil microbial composition is involved in regulating specific soil enzyme activities, which are used to assess the function of soil carbon transformation and turnover (Waldrop & Firestone, 2004). Our results suggest that shifts in soil microbial community structure mediated microbial functions, and that their interactions regulated soil carbon turnover under treatments of nitrogen addition with plant inputs removal. Our results are an important addition to the literature on this topic (You et al., 2014; Brockett, Prescott & Grayston, 2012).

In this paper, the biomass of GN and the activity of PER were found to significantly affect soil carbon properties under nitrogen addition. The activity of PER increased with the biomass of GN at higher levels of nitrogen addition, which enhanced the response of soil carbon to linkages between oxidases and bacteria (Cusack et al., 2011). The changes in carbon inputs, in the form of litter and root exudates, altered soil microbial community utilization and influenced functional shifts in soil enzymes (Baumann et al., 2013; Tardy et al., 2015).

Our results also suggest that the interactions between nitrogen addition and plant inputs removal significantly affected GN. Furthermore, the biomass of GN and Sap directly regulated soil enzyme activities and soil carbon properties, because Sap was correlated with below- and above-ground carbon decomposition under plant inputs removal (Boldtburisch et al., 2018; Bennett et al., 2017). GN and Sap regulated SOC by affecting on the activity of PER, which was involved in lignin degradation. However, some previous have reported that oxidases are produced directly by fungi (Jiang, Cao & Zhang, 2014; Zhang et al., 2014).

## Conclusion

Our study revealed that moderate nitrogen addition increased soil microbial biomass. The interaction between nitrogen addition and plant inputs removal significantly affected soil microbial community structure and function. The biomass of gram-negative bacteria and saprotrophic fungi directly affected how soil microbial function relates to carbon turnover. Altogether, microbial community structure and function, the biomass of gram-negative bacteria and peroxidase activity were the key factors regulating soil carbon dynamics. This study suggests that nitrogen addition and plant inputs removal could alter soil enzyme activities and further affect soil carbon turnover via microbial regulations. These findings have important implications for forest management. The increase in soil microbial biomass and the microbial regulation on soil carbon both need to be considered when developing sustainable forest management practices for northern China. Moreover, further studies are also needed to more precisely understand the complex interaction between the plant and the below-ground processes and how it affects soil microbial community structure.

##  Supplemental Information

10.7717/peerj.7343/supp-1 Table S1 Classification of microbial community types GP, Gram-positive bacteria; GN, Gram-negative bacteria; AMF, arbuscular mycorrhizal fungi; Sap, Saprotrophic fungi, actinomycete: Act.

10.7717/peerj.7343/supp-2 File S1 Soil microbial PLFAs The raw data showed the soil microbial PLFAs files in the year of 2015 and 2016. Each file of rtf. represented the microbial PLFAs for each soil sample. In the Supplemental File, the Excel file named “Numbers” showed the plots names and the related rtf. file names.

## Additional Information and Declarations

### Data Availability

The following information was supplied regarding data availability: The raw measurements are available in the Supplemental Files.
