# Supplementary material for: Microbial regulation of soil carbon properties under nitrogen addition and plant inputs removal
Source: PeerJ. 2019 Jul 17;7:e7343. doi: 10.7717/peerj.7343 (PMC6642627; doi:10.7717/peerj.7343)
Supplement: Table S1 — GP, Gram-positive bacteria; GN, Gram-negative bacteria; AMF, arbuscular mycorrhizal fungi; Sap, Saprotrophic fungi, actinomycete: Act. [file peerj-07-7343-s001.docx]

**Supplemental Table S1** Classification of microbial community types.

| Microbial community types | PLFAs | Reference |
| --- | --- | --- |
| GP | 13:0 iso, 14:0 iso, 15:0 iso, 16:0 iso, 17:0 iso 18:0 iso, 19:0 iso, a15:0, a16:0, a17:0 | Chen et al. (2017); Ma et al. (2015) |
| GN | 15:1 w4c, 16:1 w6c, 16:1 w7c, 16:1 w9c, 18:1 w5c, 18:1 w7c, 18:1 w9c, 17:0 cyclo, 19:0 cyclo, | Lozano et al. (2014); Bowman et al. (1991); Ma et al. (2015); Chen et al. (2017); Nie et al. (2013) |
| Sap | 18:2w6c, 18:2w9c | Smith et al. (2014); You et al. (2014); Nie et al. (2013) |
| AMF | 16:1 w5c | You et al. (2014); Nie et al. (2013); Smith et al. (2014) |
| Act | 10Me16:0, 10Me17:0, 10Me18:0, 10Me19:0 | Brockett et al. (2012) |

GP: Gran-positive bacteria, GN: Gram-negative bacteria, AMF: arbuscular mycorrhizal fungi, Sap: Saprotrophic fungi, actinomycete: Act.
