# Supplementary material for: Microbial regulation of soil carbon properties under nitrogen addition and plant inputs removal
Source: PeerJ. 2019 Jul 17;7:e7343. doi: 10.7717/peerj.7343 (PMC6642627; doi:10.7717/peerj.7343)
Supplement: File S1 — The raw data showed the soil microbial PLFAs files in the year of 2015 and 2016. Each file of rtf. represented the microbial PLFAs for each soil sample. In the Supplemental File, the Excel file named “Numbers” showed the plots names and the related rtf. file names. [file peerj-07-7343-s002.zip › supplementary files/2015/1.rtf]

Volume: DATA            File: E164203.63A        Samp Ctr: 4                  ID Number: 29299 
Type: Samp                   Bottle: 3                        Method: PLFAD1 
Created: 4/20/2016 10:09:16 AM 
Sample ID: 1 


ÌØÕ÷·åÖÍÁôÊ±¼äRT	ÌØÕ÷·åÖµResponse	Ãæ»ý/·åÖµ±ÈAr/Ht	Òò×ÓÏà¹ØÏµÊýRFact	PLFAÏà¶ÔÁ´³¤¶ÈECL	PLFAs·åÃûPeak Name	¸Ã·åÕ¼×ÜÌáÈ¡Á¿µÄ°Ù·ÖÊýPercent	Comment1	Comment2	
0.7147	1.874E+9	0.015	----	7.6511	SOLVENT PEAK	----	< min rt		
0.7873	5611	0.019	----	8.1240		----	< min rt		
0.8872	1243	0.010	----	8.7751		----	< min rt		
0.9465	592	0.014	----	9.1604		----	< min rt		
1.1878	1777	0.011	----	10.7325		----			
1.2650	818	0.011	1.208	11.1718	10:0 2OH	0.04	ECL deviates -0.012		
1.3543	759	0.017	----	11.5973		----			
1.3920	1134	0.015	----	11.7772		----			
1.4396	3164	0.014	1.138	12.0025	12:0	0.14	ECL deviates  0.002	Reference  0.004	
1.4968	1912	0.017	----	12.2074		----			
1.5619	1012	0.016	----	12.4410		----			
1.6080	2881	0.013	1.094	12.6064	13:0 iso	0.12	ECL deviates -0.006	Reference -0.005	
1.6383	2067	0.015	1.088	12.7151	13:0 anteiso	0.08	ECL deviates  0.006	Reference  0.007	
1.6942	897	0.018	1.075	12.9153	13:1 w5c	0.04	ECL deviates -0.005		
1.7181	1606	0.015	1.071	13.0008	13:0	0.06	ECL deviates  0.001	Reference  0.002	
1.7859	734	0.017	----	13.1896	12:0 2OH	----	ECL deviates  0.003		
1.8769	1595	0.018	----	13.4432		----			
1.9360	34778	0.013	1.038	13.6079	14:0 iso	1.36	ECL deviates -0.006	Reference -0.006	
1.9769	899	0.012	1.033	13.7220	14:0 anteiso	0.03	ECL deviates  0.006	Reference  0.007	
1.9968	873	0.010	1.031	13.7775	14:1 w9c	0.03	ECL deviates  0.000		
2.0113	1601	0.013	----	13.8177		----			
2.0763	37250	0.014	1.021	13.9991	14:0	1.43	ECL deviates -0.001	Reference  0.000	
2.1051	477	0.011	----	14.0641		----			
2.1335	1057	0.015	----	14.1279	14:0 iso 3OH	----	ECL deviates  0.003		
2.1576	2328	0.021	----	14.1822		----			
2.2203	2061	0.022	----	14.3236		----			
2.2705	36302	0.017	1.005	14.4367	15:1 iso w6c	1.37	ECL deviates -0.002		
2.2896	5710	0.011	1.003	14.4797	15:4 w3c	0.22	ECL deviates -0.011		
2.3110	9289	0.014	1.001	14.5278	15:1 anteiso w9c	0.35	ECL deviates -0.002		
2.3496	163200	0.013	0.999	14.6147	15:0 iso	6.13	ECL deviates -0.002	Reference -0.002	
2.3914	115258	0.014	0.996	14.7090	15:0 anteiso	4.31	ECL deviates -0.002	Reference -0.002	
2.4183	820	0.009	0.994	14.7697	15:1 w9c	0.03	ECL deviates -0.001		
2.4569	6442	0.025	0.991	14.8565	15:1 w6c	0.24	ECL deviates -0.003		
2.5205	18062	0.015	0.987	14.9997	15:0	0.67	ECL deviates  0.000	Reference  0.000	
2.5487	6480	0.017	----	15.0535		----			
2.6103	1624	0.020	----	15.1703		----			
2.6406	2692	0.019	----	15.2280		----			
2.7257	4781	0.014	0.978	15.3896	16:1 w7c alcohol	0.18	ECL deviates -0.007		
2.7528	26664	0.019	0.976	15.4410	15:0 DMA	0.98	ECL deviates -0.009		
2.8132	68304	0.015	0.974	15.5557	16:0 N alcohol	2.50	ECL deviates -0.001		
2.8459	68755	0.015	0.973	15.6179	16:0 iso	2.52	ECL deviates -0.002	Reference -0.002	
2.8986	7240	0.016	0.971	15.7178	16:0 anteiso	0.26	ECL deviates  0.003	Reference  0.002	
2.9231	37973	0.016	0.970	15.7644	16:1 w9c	1.39	ECL deviates -0.011		
2.9530	287867	0.018	0.969	15.8211	16:1 w7c	10.49	ECL deviates -0.003		
3.0000	85469	0.016	0.968	15.9105	16:1 w5c	3.11	ECL deviates -0.001		
3.0489	320892	0.015	0.966	16.0030	16:0	11.66	ECL deviates  0.003	Reference  0.002	
3.0763	14599	0.020	----	16.0489		----			
3.1309	2398	0.017	0.964	16.1401	16:2 DMA	0.09	ECL deviates  0.002		
3.1665	5131	0.022	----	16.1996		----			
3.2026	2732	0.019	----	16.2599		----			
3.2362	1470	0.019	0.962	16.3163	16:1 w7c DMA	0.05	ECL deviates  0.006		
3.2988	159718	0.019	0.961	16.4208	16:0 10-methyl	5.77	ECL deviates  0.001		
3.3345	39020	0.017	----	16.4806		----			
3.3628	18772	0.019	0.960	16.5278	17:1 anteiso w9c	0.68	ECL deviates -0.008		
3.4189	40880	0.016	0.959	16.6217	17:0 iso	1.47	ECL deviates -0.002	Reference -0.003	
3.4758	48011	0.017	0.958	16.7167	17:0 anteiso	1.73	ECL deviates -0.004		
3.5201	28379	0.018	0.957	16.7908	17:1 w8c	1.02	ECL deviates -0.006		
3.5798	87507	0.018	0.957	16.8907	17:0 cyclo w7c	3.15	ECL deviates -0.003		
3.6452	14064	0.018	0.956	17.0001	17:0	0.51	ECL deviates  0.000	Reference -0.001	
3.6709	16387	0.016	0.956	17.0392	17:1 w7c 10-methyl	0.59	ECL deviates -0.004		
3.7141	4416	0.017	----	17.1052		----			
3.7479	1409	0.021	----	17.1567		----			
3.7982	2440	0.018	0.955	17.2333	16:0 2OH	0.09	ECL deviates -0.007		
3.9093	19115	0.017	0.954	17.4027	17:0 10-methyl	0.69	ECL deviates -0.004		
3.9459	1853	0.012	0.954	17.4584	17:0 DMA	0.07	ECL deviates  0.000		
3.9668	5679	0.021	----	17.4903		----			
4.0222	5527	0.014	0.954	17.5747	18:3 w6c	0.20	ECL deviates -0.005		
4.0413	18337	0.024	----	17.6038		----			
4.1165	60494	0.016	0.953	17.7185	18:2 w6c	2.17	ECL deviates -0.009		
4.1492	183978	0.018	0.953	17.7682	18:1 w9c	6.59	ECL deviates -0.006		
4.1864	302144	0.017	0.953	17.8249	18:1 w7c	10.83	ECL deviates -0.002		
4.2416	43486	0.022	----	17.9091		----			
4.3000	50706	0.017	0.953	17.9982	18:0	1.82	ECL deviates -0.002	Reference -0.004	
4.3568	19079	0.019	0.953	18.0804	18:1 w7c 10-methyl	0.68	ECL deviates -0.005		
4.4100	6368	0.026	0.953	18.1572	18:2 DMA	0.23	ECL deviates -0.003		
4.4569	4951	0.033	0.953	18.2250	18:1 w9c DMA	0.18	ECL deviates -0.012		
4.5158	1341	0.017	----	18.3102		----			
4.5696	71012	0.021	0.954	18.3880	18:0 10-methyl	2.55	ECL deviates -0.007		
4.6388	2067	0.021	0.954	18.4879	19:4 w6c	0.07	ECL deviates  0.003		
4.6876	5800	0.025	0.954	18.5585	19:3 w6c	0.21	ECL deviates -0.001		
4.7546	3577	0.025	0.955	18.6553	19:3 w3c	0.13	ECL deviates -0.003		
4.8165	9520	0.024	----	18.7447		----			
4.8623	8307	0.019	0.955	18.8109	19:1 w8c	0.30	ECL deviates  0.000		
4.9257	87315	0.022	0.955	18.9026	19:0 cyclo w7c	3.14	ECL deviates -0.007		
4.9941	71103	0.017	----	19.0014	19:0	----	ECL deviates  0.001		
5.0562	1667	0.016	----	19.0881		----			
5.1511	1259	0.016	----	19.2206		----			
5.1824	8792	0.019	----	19.2641		----			
5.2687	20684	0.030	----	19.3847		----			
5.3229	8313	0.020	0.958	19.4602	20:5 w3c	0.30	ECL deviates -0.022		
5.3591	1951	0.017	----	19.5108		----			
5.3893	4296	0.018	----	19.5529		----			
5.4218	7757	0.024	----	19.5983		----			
5.5403	17792	0.027	0.960	19.7636	20:1 w9c	0.64	ECL deviates -0.009		
5.5689	7988	0.024	0.960	19.8035	20:1 w8c	0.29	ECL deviates -0.009		
5.7108	16870	0.022	0.961	20.0015	20:0	0.61	ECL deviates  0.002	Reference -0.001	
5.7646	967	0.021	----	20.0756		----			
5.8131	1877	0.017	----	20.1422		----			
5.8432	5188	0.020	----	20.1837		----			
5.9269	2799	0.021	----	20.2988		----			
5.9579	3562	0.016	----	20.3415		----			
5.9869	19152	0.022	----	20.3814		----			
6.0548	794	0.016	----	20.4748		----			
6.1109	2712	0.027	----	20.5519		----			
6.1621	5860	0.027	----	20.6224		----			
6.2216	3407	0.029	----	20.7042		----			
6.2854	8463	0.018	0.965	20.7921	21:1 w8c	0.31	ECL deviates -0.006		
6.3441	8422	0.021	----	20.8727		----			
6.4028	13219	0.020	0.966	20.9535	21:1 w3c	0.48	ECL deviates -0.001		
6.4388	5462	0.023	0.966	21.0030	21:0	0.20	ECL deviates  0.003	Reference  0.000	
6.5171	2610	0.020	----	21.1112		----			
6.6042	3747	0.025	0.967	21.2315	22:5 w6c	0.14	ECL deviates -0.020		
6.6371	5580	0.018	----	21.2770		----			
6.6585	1503	0.013	0.967	21.3065	22:6 w3c	0.05	ECL deviates -0.025		
6.7655	1168	0.028	0.968	21.4544	22:5 w3c	0.04	ECL deviates -0.013		
6.8877	7663	0.028	0.968	21.6230	22:0 iso	0.28	ECL deviates  0.005		
6.9649	2353	0.029	0.968	21.7298	22:2 w6c	0.09	ECL deviates -0.009		
7.0004	2289	0.023	0.968	21.7788	22:1 w9c	0.08	ECL deviates  0.006		
7.0345	4094	0.027	----	21.8259		----			
7.1149	3730	0.017	0.969	21.9369	22:1 w3c	0.14	ECL deviates -0.010		
7.1601	18225	0.017	0.969	21.9994	22:0	0.66	ECL deviates -0.001	Reference -0.004	
7.2254	1322	0.028	----	22.0908		----			
7.3327	9509	0.019	----	22.2410		----			
7.6123	1072	0.024	----	22.6326		----			
7.7146	2620	0.019	----	22.7758		----			
7.7761	1205	0.017	----	22.8620		----			
7.8187	6688	0.020	0.967	22.9215	23:1 w4c	0.24	ECL deviates -0.005		
7.8751	3977	0.018	0.966	23.0005	23:0	0.14	ECL deviates  0.001	Reference -0.003	
7.9189	1012	0.020	----	23.0626		----			
8.0806	5089	0.020	----	23.2922		----			
8.3301	4940	0.029	----	23.6464		----			
8.3906	1049	0.017	----	23.7323		----			
8.4227	2186	0.025	0.959	23.7779	24:1 w9c	0.08	ECL deviates -0.009		
8.4985	2508	0.033	----	23.8854		----			
8.5779	14323	0.019	0.956	23.9981	24:0	0.51	ECL deviates -0.002	Reference -0.006	
8.6821	892	0.021	----	24.1461		----	> max rt		
8.9351	14374	0.021	----	24.5052		----	> max rt		
9.2350	13598	0.024	----	24.9308		----	> max rt		
9.4746	6509	0.020	----	25.2709		----	> max rt		

ECL Deviation: 0.007                            Reference ECL Shift: 0.004       Number Reference Peaks: 21
Total Response: 3061417                       Total Named: 2746539
Percent Named: 89.71%                         Total Amount: 2659846

(No search libraries specified in method PLFAD1.)
