# Supplementary material for: Microbial regulation of soil carbon properties under nitrogen addition and plant inputs removal
Source: PeerJ. 2019 Jul 17;7:e7343. doi: 10.7717/peerj.7343 (PMC6642627; doi:10.7717/peerj.7343)
Supplement: File S1 — The raw data showed the soil microbial PLFAs files in the year of 2015 and 2016. Each file of rtf. represented the microbial PLFAs for each soil sample. In the Supplemental File, the Excel file named “Numbers” showed the plots names and the related rtf. file names. [file peerj-07-7343-s002.zip › supplementary files/2015/2.rtf]

Volume: DATA            File: E164203.63A        Samp Ctr: 5                  ID Number: 29300 
Type: Samp                   Bottle: 4                        Method: PLFAD1 
Created: 4/20/2016 10:37:50 AM 
Sample ID: 2 


RT	Response	Ar/Ht	RFact	ECL	Peak Name	Percent	Comment1	Comment2	
0.7151	1.883E+9	0.015	----	7.6554	SOLVENT PEAK	----	< min rt		
0.7873	5353	0.019	----	8.1261		----	< min rt		
0.8872	848	0.010	----	8.7765		----	< min rt		
0.9467	640	0.013	----	9.1631		----	< min rt		
1.1880	1665	0.011	----	10.7352		----			
1.2652	460	0.009	1.208	11.1739	10:0 2OH	0.02	ECL deviates -0.010		
1.3918	908	0.014	----	11.7774		----			
1.4393	2562	0.015	1.138	12.0024	12:0	0.12	ECL deviates  0.002	Reference  0.003	
1.4964	1594	0.016	----	12.2073		----			
1.5621	844	0.015	----	12.4428		----			
1.6073	2358	0.013	1.094	12.6048	13:0 iso	0.10	ECL deviates -0.008	Reference -0.007	
1.6386	1736	0.014	1.088	12.7170	13:0 anteiso	0.08	ECL deviates  0.008	Reference  0.008	
1.6940	963	0.018	1.075	12.9159	13:1 w5c	0.04	ECL deviates -0.004		
1.7168	1015	0.012	1.071	12.9975	13:0	0.04	ECL deviates -0.003	Reference -0.003	
1.7859	659	0.015	----	13.1905	12:0 2OH	----	ECL deviates  0.004		
1.8767	1319	0.020	----	13.4436		----			
1.9358	24963	0.013	1.038	13.6084	14:0 iso	1.03	ECL deviates -0.006	Reference -0.006	
1.9553	1414	0.009	----	13.6626		----			
1.9776	791	0.013	1.033	13.7249	14:0 anteiso	0.03	ECL deviates  0.009	Reference  0.009	
1.9962	776	0.010	1.031	13.7767	14:1 w9c	0.03	ECL deviates -0.001		
2.0108	1241	0.013	----	13.8173		----			
2.0760	32698	0.014	1.021	13.9992	14:0	1.33	ECL deviates -0.001	Reference -0.001	
2.1026	520	0.011	----	14.0592		----			
2.1317	939	0.014	----	14.1249	14:0 iso 3OH	----	ECL deviates  0.000		
2.1570	2063	0.024	----	14.1818		----			
2.2244	1624	0.019	----	14.3337		----			
2.2700	30649	0.017	1.005	14.4364	15:1 iso w6c	1.23	ECL deviates -0.003		
2.2887	6731	0.012	1.003	14.4785	15:4 w3c	0.27	ECL deviates -0.012		
2.3103	6838	0.014	1.001	14.5272	15:1 anteiso w9c	0.27	ECL deviates -0.003		
2.3491	155286	0.013	0.999	14.6145	15:0 iso	6.18	ECL deviates -0.003	Reference -0.003	
2.3902	106134	0.014	0.996	14.7072	15:0 anteiso	4.21	ECL deviates -0.004	Reference -0.005	
2.4566	5591	0.024	0.991	14.8566	15:1 w6c	0.22	ECL deviates -0.003		
2.5204	17341	0.015	0.987	15.0004	15:0	0.68	ECL deviates  0.000	Reference -0.001	
2.5485	7009	0.017	----	15.0539		----			
2.6108	1570	0.018	----	15.1721		----			
2.6415	1802	0.018	----	15.2304		----			
2.7272	5064	0.016	0.978	15.3931	16:1 w7c alcohol	0.20	ECL deviates -0.003		
2.7528	24234	0.021	0.976	15.4417	15:0 DMA	0.94	ECL deviates -0.009		
2.8128	66801	0.016	0.974	15.5558	16:0 N alcohol	2.59	ECL deviates -0.001		
2.8459	61257	0.015	0.973	15.6185	16:0 iso	2.37	ECL deviates -0.001	Reference -0.003	
2.8985	7245	0.013	0.971	15.7184	16:0 anteiso	0.28	ECL deviates  0.003	Reference  0.002	
2.9246	36549	0.018	0.970	15.7679	16:1 w9c	1.41	ECL deviates -0.007		
2.9528	258399	0.016	0.969	15.8215	16:1 w7c	9.98	ECL deviates -0.003		
3.0005	77463	0.016	0.968	15.9122	16:1 w5c	2.99	ECL deviates  0.001		
3.0491	267549	0.015	0.966	16.0041	16:0	10.30	ECL deviates  0.004	Reference  0.003	
3.0767	14772	0.019	----	16.0503		----			
3.1320	1809	0.016	0.964	16.1427	16:2 DMA	0.07	ECL deviates  0.005		
3.1654	4561	0.020	----	16.1986		----			
3.2029	2472	0.018	----	16.2611		----			
3.2374	1449	0.020	0.962	16.3189	16:1 w7c DMA	0.06	ECL deviates  0.009		
3.3004	157836	0.019	0.961	16.4242	16:0 10-methyl	6.04	ECL deviates  0.004		
3.3357	38264	0.017	0.960	16.4832	17:1 iso w9c	1.46	ECL deviates -0.015		
3.3635	17854	0.018	0.960	16.5297	17:1 anteiso w9c	0.68	ECL deviates -0.006		
3.4186	39067	0.015	0.959	16.6219	17:0 iso	1.49	ECL deviates -0.002	Reference -0.004	
3.4761	41670	0.018	0.958	16.7180	17:0 anteiso	1.59	ECL deviates -0.002		
3.5201	25400	0.018	0.957	16.7915	17:1 w8c	0.97	ECL deviates -0.005		
3.5801	85470	0.018	0.957	16.8919	17:0 cyclo w7c	3.26	ECL deviates -0.002		
3.6448	12132	0.017	0.956	16.9999	17:0	0.46	ECL deviates  0.000	Reference -0.002	
3.6708	17098	0.016	0.956	17.0397	17:1 w7c 10-methyl	0.65	ECL deviates -0.004		
3.7134	4235	0.016	----	17.1047		----			
3.7503	1055	0.019	----	17.1609		----			
3.7997	2289	0.019	0.955	17.2363	16:0 2OH	0.09	ECL deviates -0.004		
3.9089	16319	0.017	0.954	17.4025	17:0 10-methyl	0.62	ECL deviates -0.004		
3.9456	2025	0.013	0.954	17.4586	17:0 DMA	0.08	ECL deviates  0.000		
3.9682	5297	0.020	----	17.4930		----			
4.0226	4771	0.014	0.954	17.5759	18:3 w6c	0.18	ECL deviates -0.004		
4.0423	15299	0.025	----	17.6058		----			
4.1165	55500	0.017	0.953	17.7190	18:2 w6c	2.11	ECL deviates -0.008		
4.1500	161158	0.019	0.953	17.7700	18:1 w9c	6.12	ECL deviates -0.005		
4.1867	267512	0.017	0.953	17.8259	18:1 w7c	10.16	ECL deviates -0.001		
4.2445	30719	0.021	0.953	17.9140	18:1 w5c	1.17	ECL deviates -0.009		
4.3000	43833	0.018	0.953	17.9986	18:0	1.66	ECL deviates -0.001	Reference -0.004	
4.3552	18293	0.018	0.953	18.0785	18:1 w7c 10-methyl	0.69	ECL deviates -0.006		
4.4092	5927	0.028	0.953	18.1565	18:2 DMA	0.23	ECL deviates -0.003		
4.4568	4298	0.029	0.953	18.2254	18:1 w9c DMA	0.16	ECL deviates -0.012		
4.5192	1256	0.018	----	18.3155		----			
4.5691	67445	0.020	0.954	18.3876	18:0 10-methyl	2.56	ECL deviates -0.007		
4.6372	2123	0.021	0.954	18.4860	19:4 w6c	0.08	ECL deviates  0.001		
4.6868	5568	0.025	0.954	18.5577	19:3 w6c	0.21	ECL deviates -0.002		
4.7563	3552	0.028	0.955	18.6582	19:3 w3c	0.14	ECL deviates  0.000		
4.8160	8721	0.020	----	18.7444		----			
4.8607	9533	0.019	0.955	18.8090	19:1 w8c	0.36	ECL deviates -0.002		
4.8968	9946	0.013	0.955	18.8612	19:1 w6c	0.38	ECL deviates  0.009		
4.9243	79949	0.019	0.955	18.9009	19:0 cyclo w7c	3.04	ECL deviates -0.009		
4.9944	73028	0.019	----	19.0021	19:0	----	ECL deviates  0.002		
5.0551	1517	0.020	----	19.0870		----			
5.1496	1621	0.018	----	19.2188		----			
5.1826	7039	0.018	----	19.2649		----			
5.2245	1274	0.013	0.957	19.3233	19:0 cyclo 9,10 DMA	0.05	ECL deviates  0.000		
5.2679	13991	0.026	----	19.3839		----			
5.3237	6178	0.018	0.958	19.4617	20:5 w3c	----	Below has same name		
5.3558	1326	0.016	----	19.5064	20:5 w3c	----	Above has same name		
5.3886	3698	0.020	----	19.5523		----			
5.4230	6735	0.022	----	19.6003		----			
5.5413	14910	0.024	0.960	19.7654	20:1 w9c	0.57	ECL deviates -0.007		
5.5696	7970	0.024	0.960	19.8048	20:1 w8c	0.30	ECL deviates -0.008		
5.7097	14161	0.020	0.961	20.0002	20:0	0.54	ECL deviates  0.000	Reference -0.003	
5.8123	1003	0.014	----	20.1414		----			
5.8423	3702	0.016	----	20.1827		----			
5.9875	23582	0.027	0.963	20.3825	20:0 10-methyl	0.90	ECL deviates -0.015		
6.1090	1709	0.024	----	20.5496		----			
6.1614	6199	0.026	----	20.6216		----			
6.2207	2442	0.027	----	20.7032		----			
6.2869	8389	0.020	0.965	20.7943	21:1 w8c	0.32	ECL deviates -0.004		
6.3453	5110	0.022	----	20.8746		----			
6.4014	15588	0.019	0.966	20.9517	21:1 w3c	0.60	ECL deviates -0.002		
6.4385	5896	0.024	0.966	21.0027	21:0	0.23	ECL deviates  0.003	Reference  0.000	
6.5172	2970	0.020	----	21.1115		----			
6.6043	3316	0.026	0.967	21.2318	22:5 w6c	0.13	ECL deviates -0.020		
6.6362	5923	0.022	----	21.2759		----			
6.7638	1187	0.026	0.968	21.4521	22:5 w3c	0.05	ECL deviates -0.016		
6.8853	9533	0.030	0.968	21.6200	22:0 iso	0.37	ECL deviates  0.002		
6.9622	1981	0.022	0.968	21.7261	22:2 w6c	0.08	ECL deviates -0.012		
6.9963	2212	0.022	0.968	21.7732	22:1 w9c	0.09	ECL deviates  0.000		
7.0361	3844	0.027	----	21.8282		----			
7.1126	4587	0.020	0.969	21.9338	22:1 w3c	0.18	ECL deviates -0.013		
7.1595	16501	0.018	0.969	21.9987	22:0	0.64	ECL deviates -0.001	Reference -0.005	
7.2202	1989	0.033	----	22.0835		----			
7.3327	8878	0.019	----	22.2411		----			
7.3863	1233	0.024	----	22.3161		----			
7.6146	1590	0.029	0.968	22.6357	23:3 w3c	0.06	ECL deviates -0.009		
7.7134	2328	0.017	----	22.7742		----			
7.7737	803	0.017	----	22.8585		----			
7.8173	8047	0.019	0.967	22.9196	23:1 w4c	0.31	ECL deviates -0.007		
7.8737	3239	0.017	0.966	22.9986	23:0	0.12	ECL deviates -0.001	Reference -0.005	
7.9224	971	0.020	----	23.0676		----			
8.0813	4891	0.020	----	23.2932		----			
8.3307	4480	0.022	0.960	23.6471	24:3 w3c	0.17	ECL deviates -0.008		
8.3920	1329	0.021	----	23.7342		----			
8.4223	1308	0.018	0.959	23.7771	24:1 w9c	0.05	ECL deviates -0.009		
8.5005	1061	0.021	----	23.8881		----			
8.5798	13473	0.019	0.956	24.0007	24:0	0.51	ECL deviates  0.001	Reference -0.003	
8.6824	840	0.018	----	24.1464		----	> max rt		
8.9357	10665	0.019	----	24.5058		----	> max rt		
9.2360	18161	0.021	----	24.9321		----	> max rt		
9.4749	7774	0.020	----	25.2711		----	> max rt		

ECL Deviation: 0.007                            Reference ECL Shift: 0.004       Number Reference Peaks: 21
Total Response: 2773215                       Total Named: 2593485
Percent Named: 93.52%                         Total Amount: 2515982
Profile Comment:   Review report comments.

(No search libraries specified in method PLFAD1.)
