# Supplementary material for: Microbial regulation of soil carbon properties under nitrogen addition and plant inputs removal
Source: PeerJ. 2019 Jul 17;7:e7343. doi: 10.7717/peerj.7343 (PMC6642627; doi:10.7717/peerj.7343)
Supplement: File S1 — The raw data showed the soil microbial PLFAs files in the year of 2015 and 2016. Each file of rtf. represented the microbial PLFAs for each soil sample. In the Supplemental File, the Excel file named “Numbers” showed the plots names and the related rtf. file names. [file peerj-07-7343-s002.zip › supplementary files/2015/3.rtf]

Volume: DATA            File: E164203.63A        Samp Ctr: 6                  ID Number: 29301 
Type: Samp                   Bottle: 5                        Method: PLFAD1 
Created: 4/20/2016 11:06:31 AM 
Sample ID: 3 


RT	Response	Ar/Ht	RFact	ECL	Peak Name	Percent	Comment1	Comment2	
0.7151	1.88E+9	0.015	----	7.6565	SOLVENT PEAK	----	< min rt		
0.7879	5011	0.020	----	8.1308		----	< min rt		
0.8871	1994	0.010	----	8.7771		----	< min rt		
1.1880	1772	0.011	----	10.7383		----			
1.2649	683	0.012	1.208	11.1751	10:0 2OH	0.03	ECL deviates -0.009		
1.2805	357	0.009	----	11.2496		----			
1.3538	958	0.018	----	11.5983		----			
1.3923	1079	0.014	----	11.7815		----			
1.4390	3363	0.014	1.138	12.0033	12:0	0.12	ECL deviates  0.003	Reference  0.002	
1.4967	2027	0.015	----	12.2101		----			
1.5611	1179	0.016	----	12.4408		----			
1.6076	3218	0.012	1.094	12.6076	13:0 iso	0.11	ECL deviates -0.005	Reference -0.006	
1.6358	2283	0.015	1.088	12.7084	13:0 anteiso	0.08	ECL deviates -0.001	Reference -0.002	
1.6933	892	0.016	1.075	12.9146	13:1 w5c	0.03	ECL deviates -0.005		
1.7178	1565	0.014	1.071	13.0021	13:0	0.05	ECL deviates  0.002	Reference  0.001	
1.7862	619	0.015	----	13.1928	12:0 2OH	----	ECL deviates  0.007		
1.8766	1620	0.017	----	13.4445		----			
1.9074	345	0.008	----	13.5302		----			
1.9363	31900	0.012	1.038	13.6107	14:0 iso	1.06	ECL deviates -0.003	Reference -0.005	
1.9550	1511	0.009	----	13.6630		----			
1.9766	1020	0.013	1.033	13.7229	14:0 anteiso	0.03	ECL deviates  0.007	Reference  0.006	
1.9960	1196	0.011	1.031	13.7770	14:1 w9c	0.04	ECL deviates  0.000		
2.0110	1693	0.013	----	13.8189		----			
2.0444	2644	0.013	1.025	13.9120	14:1 w5c	0.09	ECL deviates  0.001		
2.0759	38733	0.014	1.021	13.9997	14:0	1.27	ECL deviates  0.000	Reference -0.002	
2.1041	557	0.011	----	14.0634		----			
2.1317	988	0.014	----	14.1257	14:0 iso 3OH	----	ECL deviates  0.001		
2.1581	2714	0.024	----	14.1849		----			
2.2143	1655	0.020	----	14.3115		----			
2.2702	46452	0.017	1.005	14.4375	15:1 iso w6c	1.50	ECL deviates -0.002		
2.2875	7993	0.012	1.003	14.4765	15:4 w3c	0.26	ECL deviates -0.014		
2.3108	10855	0.013	1.001	14.5290	15:1 anteiso w9c	0.35	ECL deviates -0.001		
2.3489	195534	0.014	0.999	14.6146	15:0 iso	6.27	ECL deviates -0.002	Reference -0.004	
2.3907	134677	0.014	0.996	14.7088	15:0 anteiso	4.30	ECL deviates -0.002	Reference -0.004	
2.4166	1095	0.009	0.994	14.7672	15:1 w9c	0.03	ECL deviates -0.004		
2.4559	6572	0.021	0.991	14.8556	15:1 w6c	0.21	ECL deviates -0.004		
2.4756	933	0.012	0.990	14.9000	15:1 w5c	0.03	ECL deviates -0.013		
2.5195	21528	0.014	0.987	14.9989	15:0	0.68	ECL deviates -0.001	Reference -0.003	
2.5480	6661	0.016	----	15.0534		----			
2.6104	1495	0.019	----	15.1718		----			
2.6406	2557	0.017	----	15.2291		----			
2.7268	6640	0.016	0.978	15.3928	16:1 w7c alcohol	0.21	ECL deviates -0.004		
2.7526	30684	0.020	0.976	15.4418	15:0 DMA	0.96	ECL deviates -0.009		
2.8133	72704	0.015	0.974	15.5569	16:0 N alcohol	2.27	ECL deviates  0.000		
2.8455	75965	0.015	0.973	15.6181	16:0 iso	2.37	ECL deviates -0.002	Reference -0.003	
2.8977	8415	0.013	0.971	15.7171	16:0 anteiso	0.26	ECL deviates  0.002	Reference  0.001	
2.9236	50323	0.017	0.970	15.7663	16:1 w9c	1.57	ECL deviates -0.009		
2.9528	332407	0.016	0.969	15.8217	16:1 w7c	10.34	ECL deviates -0.003		
3.0005	117625	0.016	0.968	15.9123	16:1 w5c	3.65	ECL deviates  0.001		
3.0492	361380	0.015	0.966	16.0044	16:0	11.21	ECL deviates  0.004	Reference  0.003	
3.0761	14000	0.018	----	16.0494		----			
3.1296	2430	0.015	0.964	16.1387	16:2 DMA	0.08	ECL deviates  0.001		
3.1645	5975	0.020	----	16.1972		----			
3.2004	2723	0.017	----	16.2571		----			
3.2364	2080	0.022	0.962	16.3172	16:1 w7c DMA	0.06	ECL deviates  0.007		
3.2984	180461	0.019	0.961	16.4209	16:0 10-methyl	5.57	ECL deviates  0.001		
3.3348	43340	0.018	----	16.4817		----			
3.3620	22760	0.018	----	16.5272		----			
3.4183	46348	0.016	0.959	16.6214	17:0 iso	1.43	ECL deviates -0.002	Reference -0.004	
3.4755	52872	0.018	0.958	16.7170	17:0 anteiso	1.63	ECL deviates -0.003		
3.5196	35965	0.018	0.957	16.7907	17:1 w8c	1.11	ECL deviates -0.006		
3.5802	115832	0.019	0.957	16.8920	17:0 cyclo w7c	3.56	ECL deviates -0.002		
3.6444	16871	0.018	0.956	16.9992	17:0	0.52	ECL deviates -0.001	Reference -0.003	
3.6703	17160	0.017	0.956	17.0390	17:1 w7c 10-methyl	0.53	ECL deviates -0.004		
3.7133	4559	0.017	----	17.1045		----			
3.7468	1596	0.020	----	17.1555		----			
3.7987	2858	0.020	0.955	17.2346	16:0 2OH	0.09	ECL deviates -0.006		
3.9085	22572	0.017	0.954	17.4019	17:0 10-methyl	0.69	ECL deviates -0.005		
3.9446	2147	0.014	0.954	17.4570	17:0 DMA	0.07	ECL deviates -0.001		
3.9671	6539	0.022	----	17.4912		----			
4.0215	6787	0.013	0.954	17.5741	18:3 w6c	0.21	ECL deviates -0.006		
4.0406	21906	0.024	----	17.6032		----			
4.1169	82863	0.017	0.953	17.7194	18:2 w6c	2.54	ECL deviates -0.008		
4.1506	208229	0.017	0.953	17.7709	18:1 w9c	6.37	ECL deviates -0.004		
4.1864	352507	0.017	0.953	17.8254	18:1 w7c	10.78	ECL deviates -0.002		
4.2428	49439	0.022	----	17.9113		----			
4.3009	57913	0.018	0.953	17.9999	18:0	1.77	ECL deviates  0.000	Reference -0.002	
4.3549	23104	0.017	0.953	18.0780	18:1 w7c 10-methyl	0.71	ECL deviates -0.007		
4.4089	7577	0.027	0.953	18.1561	18:2 DMA	0.23	ECL deviates -0.004		
4.4544	4923	0.020	----	18.2218		----			
4.4902	1449	0.014	0.954	18.2736	18:1 w7c DMA	0.04	ECL deviates -0.009		
4.5173	1605	0.019	----	18.3127		----			
4.5689	85764	0.018	0.954	18.3873	18:0 10-methyl	2.63	ECL deviates -0.008		
4.6383	2128	0.019	0.954	18.4876	19:4 w6c	0.07	ECL deviates  0.003		
4.6842	9237	0.022	0.954	18.5540	19:3 w6c	0.28	ECL deviates -0.006		
4.7554	4529	0.025	0.955	18.6568	19:3 w3c	0.14	ECL deviates -0.001		
4.8145	10968	0.022	----	18.7422		----			
4.8614	13107	0.019	0.955	18.8099	19:1 w8c	0.40	ECL deviates -0.001		
4.9238	111352	0.021	0.955	18.9002	19:0 cyclo w7c	3.41	ECL deviates -0.010		
4.9939	78836	0.020	----	19.0014	19:0	----	ECL deviates  0.001		
5.0549	1937	0.016	----	19.0866		----			
5.1821	10788	0.026	----	19.2641		----			
5.2676	24699	0.032	----	19.3835		----			
5.3227	8380	0.021	0.958	19.4603	20:5 w3c	----	Below has same name		
5.3549	2060	0.016	----	19.5052	20:5 w3c	----	Above has same name		
5.3884	6295	0.019	----	19.5519		----			
5.4217	10151	0.024	----	19.5984		----			
5.5406	20791	0.025	0.960	19.7644	20:1 w9c	0.64	ECL deviates -0.008		
5.5708	9565	0.023	0.960	19.8065	20:1 w8c	0.29	ECL deviates -0.006		
5.6250	627	0.014	----	19.8822		----			
5.7098	20205	0.023	0.961	20.0005	20:0	0.62	ECL deviates  0.001	Reference -0.002	
5.7667	1345	0.019	----	20.0789		----			
5.8123	2056	0.014	----	20.1416		----			
5.8433	7942	0.021	----	20.1842		----			
5.9552	8168	0.026	----	20.3382		----			
5.9860	29894	0.022	----	20.3806		----			
6.0613	1273	0.018	----	20.4841		----			
6.1112	3844	0.027	----	20.5528		----			
6.1572	10077	0.026	----	20.6161		----			
6.2227	3846	0.031	----	20.7062		----			
6.2867	9460	0.018	0.965	20.7943	21:1 w8c	0.29	ECL deviates -0.004		
6.3449	8054	0.023	----	20.8744		----			
6.4023	17667	0.019	0.966	20.9534	21:1 w3c	0.55	ECL deviates -0.001		
6.4367	7050	0.022	0.966	21.0007	21:0	0.22	ECL deviates  0.001	Reference -0.003	
6.5183	3886	0.022	----	21.1134		----			
6.6031	5755	0.028	0.967	21.2305	22:5 w6c	0.18	ECL deviates -0.021		
6.6375	6939	0.025	----	21.2782		----			
6.7031	897	0.018	----	21.3687		----			
6.7644	1779	0.026	0.968	21.4535	22:5 w3c	0.06	ECL deviates -0.014		
6.8859	12342	0.033	0.968	21.6213	22:0 iso	0.38	ECL deviates  0.004		
6.9642	2258	0.020	0.968	21.7295	22:2 w6c	0.07	ECL deviates -0.009		
6.9936	3247	0.023	0.968	21.7701	22:1 w9c	0.10	ECL deviates -0.003		
7.0305	5552	0.028	0.968	21.8211	22:1 w8c	0.17	ECL deviates  0.008		
7.1144	5215	0.019	0.969	21.9371	22:1 w3c	0.16	ECL deviates -0.010		
7.1600	21012	0.019	0.969	22.0001	22:0	0.65	ECL deviates  0.000	Reference -0.004	
7.2439	2514	0.041	----	22.1175		----	> max ar/ht		
7.3334	9891	0.018	----	22.2429		----			
7.3830	959	0.022	----	22.3124		----			
7.6159	1250	0.023	0.968	22.6386	23:3 w3c	0.04	ECL deviates -0.006		
7.7114	2699	0.019	----	22.7723		----			
7.7742	1322	0.022	----	22.8604		----			
7.8169	8664	0.021	0.967	22.9202	23:1 w4c	0.27	ECL deviates -0.006		
7.8747	4789	0.020	0.966	23.0012	23:0	0.15	ECL deviates  0.001	Reference -0.003	
7.9181	1448	0.025	----	23.0628		----			
8.0811	5684	0.020	----	23.2942		----			
8.2939	726	0.016	0.961	23.5965	24:3 w6c	0.02	ECL deviates  0.006		
8.3299	6117	0.023	0.960	23.6476	24:3 w3c	0.19	ECL deviates -0.007		
8.3900	2111	0.022	----	23.7329		----			
8.4208	3147	0.022	0.959	23.7767	24:1 w9c	0.10	ECL deviates -0.010		
8.4981	2239	0.028	----	23.8865		----			
8.5329	772	0.016	0.957	23.9359	24:1 w3c	0.02	ECL deviates -0.013		
8.5769	17418	0.018	0.956	23.9984	24:0	0.53	ECL deviates -0.002	Reference -0.007	
8.6786	938	0.017	----	24.1428		----	> max rt		
8.9327	26249	0.020	----	24.5036		----	> max rt		
9.1661	910	0.019	----	24.8353		----	> max rt		
9.2353	18027	0.022	----	24.9335		----	> max rt		
9.4739	8260	0.020	----	25.2723		----	> max rt		

ECL Deviation: 0.007                            Reference ECL Shift: 0.004       Number Reference Peaks: 21
Total Response: 3618742                       Total Named: 3218204
Percent Named: 88.93%                         Total Amount: 3123924
Profile Comment:   Review report comments.

(No search libraries specified in method PLFAD1.)
