# Supplementary material for: Microbial regulation of soil carbon properties under nitrogen addition and plant inputs removal
Source: PeerJ. 2019 Jul 17;7:e7343. doi: 10.7717/peerj.7343 (PMC6642627; doi:10.7717/peerj.7343)
Supplement: File S1 — The raw data showed the soil microbial PLFAs files in the year of 2015 and 2016. Each file of rtf. represented the microbial PLFAs for each soil sample. In the Supplemental File, the Excel file named “Numbers” showed the plots names and the related rtf. file names. [file peerj-07-7343-s002.zip › supplementary files/2015/33.rtf]

Volume: DATA            File: E164203.63A        Samp Ctr: 37                ID Number: 29330 
Type: Samp                   Bottle: 12                      Method: PLFAD1 
Created: 4/21/2016 1:46:13 AM 
Sample ID: 33 


RT	Response	Ar/Ht	RFact	ECL	Peak Name	Percent	Comment1	Comment2	
0.7142	1.92E+9	0.015	----	7.6562	SOLVENT PEAK	----	< min rt		
0.8856	1774	0.012	----	8.7742		----	< min rt		
0.9458	1273	0.013	----	9.1664		----	< min rt		
1.0454	1313	0.014	----	9.8160		----	< min rt		
1.0742	953	0.015	1.326	10.0035	10:0	0.03	ECL deviates  0.003	Reference -0.001	
1.1872	2776	0.012	----	10.7405		----			
1.2257	571	0.013	1.220	10.9913	11:0	0.02	ECL deviates -0.009	Reference -0.013	
1.2629	1082	0.013	1.203	11.1722	10:0 2OH	0.04	ECL deviates -0.012		
1.3144	764	0.016	----	11.4181		----			
1.3541	1383	0.016	1.166	11.6075	12:0 iso	0.04	ECL deviates -0.005	Reference -0.009	
1.3660	579	0.008	----	11.6645		----			
1.3914	1907	0.016	----	11.7854		----			
1.4378	4485	0.014	1.136	12.0063	12:0	0.14	ECL deviates  0.006	Reference  0.002	
1.4954	2326	0.013	----	12.2129		----			
1.5614	1188	0.016	1.104	12.4493	12:0 DMA	0.04	ECL deviates -0.011		
1.6061	3752	0.012	1.094	12.6097	13:0 iso	0.11	ECL deviates -0.003	Reference -0.007	
1.6344	2355	0.014	1.088	12.7112	13:0 anteiso	0.07	ECL deviates  0.002	Reference -0.002	
1.6915	589	0.012	1.076	12.9159	13:1 w5c	0.02	ECL deviates -0.004		
1.7159	1316	0.012	1.072	13.0036	13:0	0.04	ECL deviates  0.004	Reference  0.000	
1.7824	810	0.016	----	13.1887	12:0 2OH	----	ECL deviates  0.003		
1.8751	2238	0.020	----	13.4470		----			
1.9338	54047	0.013	1.041	13.6106	14:0 iso	1.55	ECL deviates -0.003	Reference -0.007	
1.9757	1209	0.014	1.036	13.7274	14:0 anteiso	0.03	ECL deviates  0.011	Reference  0.008	
1.9939	1276	0.011	1.034	13.7780	14:1 w9c	0.04	ECL deviates  0.000		
2.0089	1930	0.012	----	13.8199		----			
2.0734	53515	0.015	1.024	13.9997	14:0	1.51	ECL deviates  0.000	Reference -0.004	
2.1015	753	0.012	----	14.0638		----			
2.1296	1431	0.014	----	14.1271	14:0 iso 3OH	----	ECL deviates  0.002		
2.1568	3716	0.024	----	14.1886		----			
2.2207	2766	0.021	----	14.3327		----			
2.2673	51207	0.017	1.009	14.4380	15:1 iso w6c	1.42	ECL deviates -0.001		
2.2855	7574	0.009	1.007	14.4789	15:4 w3c	0.21	ECL deviates -0.011		
2.3069	13206	0.014	1.006	14.5272	15:1 anteiso w9c	0.37	ECL deviates -0.003		
2.3460	218796	0.014	1.003	14.6156	15:0 iso	6.04	ECL deviates -0.001	Reference -0.005	
2.3874	164853	0.014	1.000	14.7089	15:0 anteiso	4.53	ECL deviates -0.002	Reference -0.005	
2.4521	8417	0.026	0.996	14.8549	15:1 w6c	0.23	ECL deviates -0.005		
2.5164	24727	0.015	0.992	15.0001	15:0	0.67	ECL deviates  0.000	Reference -0.003	
2.5445	8860	0.016	----	15.0540		----			
2.6073	1983	0.018	----	15.1731		----			
2.6365	3802	0.019	----	15.2286		----			
2.7225	7311	0.017	0.983	15.3918	16:1 w7c alcohol	0.20	ECL deviates -0.005		
2.7487	35482	0.020	0.981	15.4416	15:0 DMA	0.96	ECL deviates -0.009		
2.8092	95419	0.016	0.979	15.5565	16:0 N alcohol	2.57	ECL deviates -0.001		
2.8416	89336	0.016	0.978	15.6180	16:0 iso	2.40	ECL deviates -0.002	Reference -0.005	
2.8935	7959	0.011	0.976	15.7165	16:0 anteiso	0.21	ECL deviates  0.002	Reference -0.002	
2.9199	53984	0.017	0.975	15.7665	16:1 w9c	1.45	ECL deviates -0.008		
2.9489	394922	0.016	0.974	15.8217	16:1 w7c	10.58	ECL deviates -0.003		
2.9954	109522	0.016	0.973	15.9100	16:1 w5c	2.93	ECL deviates -0.001		
3.0452	439132	0.016	0.972	16.0043	16:0	11.74	Column Overload		
3.0718	20218	0.019	----	16.0488		----			
3.1250	3088	0.017	0.969	16.1378	16:2 DMA	0.08	ECL deviates  0.000		
3.1603	6740	0.021	----	16.1970		----			
3.1956	3356	0.018	----	16.2560		----			
3.2338	2227	0.021	0.967	16.3199	16:1 w7c DMA	0.06	ECL deviates  0.010		
3.2946	213082	0.019	0.966	16.4217	16:0 10-methyl	5.66	ECL deviates  0.002		
3.3298	50418	0.018	----	16.4805		----			
3.3579	26042	0.019	----	16.5277		----			
3.4137	53898	0.016	0.964	16.6210	17:0 iso	1.43	ECL deviates -0.003	Reference -0.006	
3.4714	61233	0.017	0.963	16.7176	17:0 anteiso	1.62	ECL deviates -0.003		
3.5152	36089	0.018	0.962	16.7909	17:1 w8c	0.96	ECL deviates -0.006		
3.5746	127509	0.019	0.962	16.8904	17:0 cyclo w7c	3.37	ECL deviates -0.003		
3.6393	17896	0.018	0.961	16.9986	17:0	0.47	ECL deviates -0.001	Reference -0.004	
3.6655	21542	0.018	0.960	17.0390	17:1 w7c 10-methyl	0.57	ECL deviates -0.004		
3.7080	5973	0.019	----	17.1037		----			
3.7434	1620	0.018	----	17.1578		----			
3.7935	3539	0.020	0.959	17.2342	16:0 2OH	0.09	ECL deviates -0.006		
3.9039	24492	0.018	0.959	17.4027	17:0 10-methyl	0.65	ECL deviates -0.004		
3.9399	2471	0.014	0.958	17.4576	17:0 DMA	0.07	ECL deviates  0.000		
3.9619	8243	0.022	----	17.4913		----			
4.0366	33166	0.030	----	17.6053		----			
4.1119	72779	0.017	0.958	17.7201	18:2 w6c	1.92	ECL deviates -0.007		
4.1445	266930	0.020	0.958	17.7698	18:1 w9c	7.03	ECL deviates -0.005		
4.1830	413874	0.018	0.957	17.8286	18:1 w7c	10.90	Column Overload		
4.2357	56607	0.022	----	17.9091		----			
4.2955	67348	0.018	0.957	18.0003	18:0	1.77	ECL deviates  0.000	Reference -0.002	
4.3510	26372	0.018	0.957	18.0806	18:1 w7c 10-methyl	0.69	ECL deviates -0.004		
4.4085	9059	0.028	0.957	18.1635	18:2 DMA	0.24	ECL deviates  0.004		
4.4525	4958	0.024	0.957	18.2271	18:1 w9c DMA	0.13	ECL deviates -0.010		
4.4833	1647	0.014	0.957	18.2716	18:1 w7c DMA	0.04	ECL deviates -0.011		
4.5120	1763	0.017	----	18.3130		----			
4.5636	102810	0.021	0.957	18.3875	18:0 10-methyl	2.71	ECL deviates -0.007		
4.6306	2824	0.020	0.957	18.4842	19:4 w6c	0.07	ECL deviates -0.001		
4.6783	9609	0.023	0.957	18.5531	19:3 w6c	0.25	ECL deviates -0.007		
4.7480	5271	0.027	0.958	18.6538	19:3 w3c	0.14	ECL deviates -0.005		
4.8088	10996	0.022	----	18.7415		----			
4.8549	11873	0.020	0.958	18.8080	19:1 w8c	0.31	ECL deviates -0.003		
4.8902	19589	0.017	0.958	18.8590	19:1 w6c	0.52	ECL deviates  0.007		
4.9189	101925	0.018	0.958	18.9004	19:0 cyclo w7c	2.69	ECL deviates -0.009		
4.9881	86082	0.019	----	19.0004	19:0	----	ECL deviates  0.000		
5.0495	1843	0.016	----	19.0860		----			
5.1400	2206	0.019	----	19.2121		----			
5.1764	11424	0.019	----	19.2628		----			
5.2624	24563	0.027	----	19.3828		----			
5.3167	8202	0.019	0.960	19.4584	20:5 w3c	0.22	ECL deviates -0.024		
5.3529	1787	0.016	----	19.5089		----			
5.3811	4682	0.019	----	19.5481		----			
5.4160	6276	0.019	----	19.5968		----			
5.5339	20502	0.025	0.961	19.7610	20:1 w9c	0.54	ECL deviates -0.012		
5.5645	8754	0.022	0.961	19.8037	20:1 w8c	0.23	ECL deviates -0.009		
5.7040	23020	0.022	0.962	19.9981	20:0	0.61	ECL deviates -0.002	Reference -0.004	
5.7580	985	0.018	----	20.0728		----			
5.8057	2185	0.016	----	20.1386		----			
5.8367	7139	0.021	----	20.1815		----			
5.9151	4627	0.020	----	20.2899		----			
5.9489	5748	0.017	----	20.3365		----			
5.9789	34743	0.022	----	20.3779		----			
6.0545	874	0.016	----	20.4824		----			
6.1045	4214	0.029	----	20.5515		----			
6.1508	9147	0.026	----	20.6155		----			
6.2090	4854	0.030	----	20.6959		----			
6.2791	11290	0.019	0.965	20.7927	21:1 w8c	0.30	ECL deviates -0.005		
6.3385	10006	0.022	----	20.8748		----			
6.3963	17197	0.022	0.965	20.9546	21:1 w3c	0.46	ECL deviates  0.001		
6.4311	7440	0.023	0.965	21.0028	21:0	0.20	ECL deviates  0.003	Reference  0.001	
6.5122	3017	0.019	----	21.1146		----			
6.5566	792	0.017	----	21.1759		----			
6.5974	4123	0.021	0.966	21.2321	22:5 w6c	0.11	ECL deviates -0.020		
6.6296	9895	0.023	----	21.2765		----			
6.8511	1273	0.016	----	21.5820		----			
6.8810	9629	0.027	0.966	21.6233	22:0 iso	0.26	ECL deviates  0.005		
6.9556	2610	0.023	0.966	21.7261	22:2 w6c	0.07	ECL deviates -0.012		
6.9908	2524	0.018	0.966	21.7748	22:1 w9c	0.07	ECL deviates  0.002		
7.0239	7051	0.026	0.966	21.8203	22:1 w8c	0.19	ECL deviates  0.007		
7.1092	4895	0.019	0.966	21.9381	22:1 w3c	0.13	ECL deviates -0.009		
7.1542	27309	0.019	0.966	22.0001	22:0	0.73	ECL deviates  0.000	Reference -0.002	
7.2169	1377	0.021	----	22.0878		----			
7.2454	889	0.020	----	22.1276		----			
7.3271	12514	0.020	----	22.2418		----			
7.3837	1706	0.030	----	22.3209		----			
7.4440	1749	0.024	----	22.4052		----			
7.4989	1266	0.024	0.964	22.4820	23:4 w6c	0.03	ECL deviates  0.011		
7.6063	3533	0.039	----	22.6321		----	> max ar/ht		
7.7101	4703	0.023	----	22.7772		----			
7.7684	2163	0.020	----	22.8587		----			
7.8114	10626	0.020	0.962	22.9188	23:1 w4c	0.28	ECL deviates -0.008		
7.8687	6111	0.018	0.961	22.9989	23:0	0.16	ECL deviates -0.001	Reference -0.003	
7.9126	1241	0.023	----	23.0611		----			
8.0754	7650	0.021	----	23.2920		----			
8.3273	6857	0.025	0.954	23.6494	24:3 w3c	0.18	ECL deviates -0.005		
8.3842	2601	0.021	----	23.7300		----			
8.4176	1922	0.019	0.952	23.7775	24:1 w9c	0.05	ECL deviates -0.009		
8.4902	2102	0.020	----	23.8805		----			
8.5251	705	0.016	----	23.9299		----			
8.5749	21544	0.021	0.949	24.0006	24:0	0.56	ECL deviates  0.001	Reference -0.001	
8.6779	1099	0.019	----	24.1468		----	> max rt		
8.9282	14670	0.019	----	24.5017		----	> max rt		
9.2328	21057	0.022	----	24.9339		----	> max rt		
9.4710	10705	0.021	----	25.2717		----	> max rt		

ECL Deviation: 0.007                            Reference ECL Shift: 0.005       Number Reference Peaks: 23
Total Response: 4191128                       Total Named: 3736373
Percent Named: 89.15%                         Total Amount: 3635386
Profile Comment:   Column Overload:  A peak's response is greater than 400000.0.  Dilute and re-run.

(No search libraries specified in method PLFAD1.)
