# Supplementary material for: Microbial regulation of soil carbon properties under nitrogen addition and plant inputs removal
Source: PeerJ. 2019 Jul 17;7:e7343. doi: 10.7717/peerj.7343 (PMC6642627; doi:10.7717/peerj.7343)
Supplement: File S1 — The raw data showed the soil microbial PLFAs files in the year of 2015 and 2016. Each file of rtf. represented the microbial PLFAs for each soil sample. In the Supplemental File, the Excel file named “Numbers” showed the plots names and the related rtf. file names. [file peerj-07-7343-s002.zip › supplementary files/2015/34.rtf]

Volume: DATA            File: E164213.59A        Samp Ctr: 3                  ID Number: 29331 
Type: Samp                   Bottle: 2                        Method: PLFAD1 
Created: 4/21/2016 9:33:50 AM 
Sample ID: 34 


RT	Response	Ar/Ht	RFact	ECL	Peak Name	Percent	Comment1	Comment2	
0.7144	1.902E+9	0.015	----	7.6561	SOLVENT PEAK	----	< min rt		
0.8856	1772	0.012	----	8.7710		----	< min rt		
0.9454	1689	0.013	----	9.1606		----	< min rt		
1.0462	2393	0.014	----	9.8171		----	< min rt		
1.0749	1120	0.018	1.322	10.0046	10:0	0.06	ECL deviates  0.004	Reference -0.003	
1.1876	1460	0.013	----	10.7388		----			
1.2642	625	0.012	1.195	11.1757	10:0 2OH	0.03	ECL deviates -0.008		
1.3142	664	0.016	----	11.4139		----			
1.3548	1284	0.015	1.157	11.6076	12:0 iso	0.06	ECL deviates -0.004	Reference -0.009	
1.3668	621	0.010	----	11.6644		----			
1.3921	2018	0.015	----	11.7854		----			
1.4382	4352	0.016	1.127	12.0047	12:0	0.20	ECL deviates  0.005	Reference  0.001	
1.4958	2530	0.016	----	12.2112		----			
1.5220	547	0.011	----	12.3056		----			
1.5611	1761	0.019	----	12.4457		----			
1.6066	4034	0.012	1.085	12.6090	13:0 iso	0.18	ECL deviates -0.003	Reference -0.007	
1.6335	2100	0.014	1.079	12.7054	13:0 anteiso	0.09	ECL deviates -0.004	Reference -0.007	
1.6916	1195	0.018	1.067	12.9139	13:1 w5c	0.05	ECL deviates -0.006		
1.7157	1352	0.013	1.062	13.0006	13:0	0.06	ECL deviates  0.001	Reference -0.002	
1.8734	1618	0.019	----	13.4408		----			
1.9341	37986	0.013	1.032	13.6099	14:0 iso	1.57	ECL deviates -0.004	Reference -0.006	
1.9745	1009	0.012	1.028	13.7227	14:0 anteiso	0.04	ECL deviates  0.007	Reference  0.005	
1.9953	900	0.010	1.025	13.7806	14:1 w9c	0.04	ECL deviates  0.003		
2.0090	1413	0.012	----	13.8189		----			
2.0741	34914	0.015	1.016	14.0002	14:0	1.42	ECL deviates  0.000	Reference -0.002	
2.1016	681	0.013	----	14.0626		----			
2.1290	949	0.013	----	14.1245	14:0 iso 3OH	----	ECL deviates  0.000		
2.1546	2127	0.020	----	14.1825		----			
2.2207	2050	0.018	----	14.3316		----			
2.2671	33518	0.017	1.001	14.4364	15:1 iso w6c	1.35	ECL deviates -0.003		
2.2868	6878	0.012	0.999	14.4809	15:4 w3c	0.28	ECL deviates -0.009		
2.3074	7692	0.014	0.998	14.5275	15:1 anteiso w9c	0.31	ECL deviates -0.003		
2.3458	162931	0.014	0.996	14.6140	15:0 iso	6.50	ECL deviates -0.003	Reference -0.004	
2.3876	115503	0.014	0.993	14.7085	15:0 anteiso	4.60	ECL deviates -0.002	Reference -0.004	
2.4523	6040	0.024	0.989	14.8545	15:1 w6c	0.24	ECL deviates -0.006		
2.5162	17730	0.015	0.985	14.9989	15:0	0.70	ECL deviates -0.001	Reference -0.002	
2.5447	7871	0.017	----	15.0534		----			
2.6070	1964	0.021	----	15.1721		----			
2.6376	1831	0.018	----	15.2303		----			
2.7224	5372	0.016	0.977	15.3918	16:1 w7c alcohol	0.21	ECL deviates -0.005		
2.7488	25424	0.020	0.976	15.4420	15:0 DMA	0.99	ECL deviates -0.009		
2.8091	73806	0.015	0.974	15.5567	16:0 N alcohol	2.88	ECL deviates  0.000		
2.8411	61717	0.015	0.973	15.6178	16:0 iso	2.41	ECL deviates -0.002	Reference -0.003	
2.8944	6975	0.016	0.971	15.7191	16:0 anteiso	0.27	ECL deviates  0.004	Reference  0.004	
2.9193	36947	0.017	0.971	15.7667	16:1 w9c	1.44	ECL deviates -0.008		
2.9479	271769	0.017	0.970	15.8211	16:1 w7c	10.57	ECL deviates -0.003		
2.9956	75554	0.015	0.969	15.9119	16:1 w5c	2.93	ECL deviates  0.001		
3.0438	273563	0.016	0.968	16.0032	16:0	10.61	ECL deviates  0.003	Reference  0.003	
3.0720	18245	0.020	----	16.0505		----			
3.1239	2172	0.016	0.966	16.1373	16:2 DMA	0.08	ECL deviates -0.001		
3.1612	4544	0.021	----	16.1997		----			
3.1956	2727	0.018	----	16.2574		----			
3.2317	1444	0.020	0.964	16.3177	16:1 w7c DMA	0.06	ECL deviates  0.008		
3.2935	164147	0.020	0.963	16.4212	16:0 10-methyl	6.34	ECL deviates  0.001		
3.3292	38645	0.017	----	16.4810		----			
3.3567	18005	0.019	----	16.5270		----			
3.4124	38248	0.016	0.962	16.6203	17:0 iso	1.47	ECL deviates -0.003	Reference -0.004	
3.4696	43235	0.017	0.961	16.7160	17:0 anteiso	1.67	ECL deviates -0.004		
3.5139	25044	0.018	0.961	16.7901	17:1 w8c	0.96	ECL deviates -0.007		
3.5736	89530	0.019	0.960	16.8901	17:0 cyclo w7c	3.45	ECL deviates -0.004		
3.6391	11828	0.017	0.960	16.9997	17:0	0.46	ECL deviates  0.000	Reference  0.000	
3.6639	18682	0.017	0.959	17.0377	17:1 w7c 10-methyl	0.72	ECL deviates -0.006		
3.7072	4880	0.017	----	17.1039		----			
3.7415	1297	0.021	----	17.1561		----			
3.7918	2043	0.018	0.959	17.2331	16:0 2OH	0.08	ECL deviates -0.007		
3.9021	16610	0.018	0.959	17.4016	17:0 10-methyl	0.64	ECL deviates -0.005		
3.9392	1632	0.012	0.959	17.4582	17:0 DMA	0.06	ECL deviates  0.000		
3.9611	5791	0.021	----	17.4917		----			
4.0348	20604	0.031	----	17.6042		----			
4.1090	49046	0.017	0.959	17.7177	18:2 w6c	1.89	ECL deviates -0.009		
4.1418	163063	0.020	0.959	17.7677	18:1 w9c	6.27	ECL deviates -0.007		
4.1774	248289	0.018	0.959	17.8222	18:1 w7c	9.54	ECL deviates -0.005		
4.2364	30541	0.023	----	17.9123		----			
4.2932	45256	0.018	0.959	17.9991	18:0	1.74	ECL deviates -0.001	Reference -0.001	
4.3493	16860	0.018	0.959	18.0802	18:1 w7c 10-methyl	0.65	ECL deviates -0.005		
4.4084	6666	0.027	0.959	18.1657	18:2 DMA	0.26	ECL deviates  0.006		
4.4507	3868	0.027	0.960	18.2268	18:1 w9c DMA	0.15	ECL deviates -0.010		
4.5109	926	0.017	----	18.3139		----			
4.5619	73850	0.021	0.960	18.3876	18:0 10-methyl	2.84	ECL deviates -0.007		
4.6300	2286	0.022	0.960	18.4861	19:4 w6c	0.09	ECL deviates  0.001		
4.6772	7504	0.025	0.961	18.5544	19:3 w6c	0.29	ECL deviates -0.006		
4.7432	3342	0.026	0.961	18.6498	19:3 w3c	0.13	ECL deviates -0.009		
4.8013	11317	0.022	0.962	18.7339	19:0 anteiso	0.44	ECL deviates  0.007	Reference  0.007	
4.8543	9434	0.019	0.962	18.8105	19:1 w8c	0.36	ECL deviates  0.000		
4.8891	9864	0.015	0.962	18.8608	19:1 w6c	0.38	ECL deviates  0.009		
4.9172	80320	0.018	0.962	18.9015	19:0 cyclo w7c	3.10	ECL deviates -0.008		
4.9858	69396	0.018	----	19.0007	19:0	----	ECL deviates  0.001		
5.0476	1504	0.021	----	19.0868		----			
5.1374	2062	0.021	----	19.2120		----			
5.1738	5358	0.018	----	19.2628		----			
5.2622	17778	0.026	0.965	19.3860	20:4 w6c	0.69	ECL deviates -0.017		
5.3149	8368	0.018	0.966	19.4596	20:5 w3c	----	Below has same name		
5.3458	1192	0.016	----	19.5026	20:5 w3c	----	Above has same name		
5.3817	4165	0.018	----	19.5527		----			
5.4147	6603	0.024	----	19.5987		----			
5.5331	15440	0.026	0.967	19.7638	20:1 w9c	0.60	ECL deviates -0.009		
5.5622	7481	0.022	0.967	19.8044	20:1 w8c	0.29	ECL deviates -0.009		
5.7010	15083	0.022	0.969	19.9980	20:0	0.59	ECL deviates -0.002	Reference -0.002	
5.8030	1494	0.017	----	20.1390		----			
5.8356	4532	0.018	----	20.1840		----			
5.9174	2259	0.016	----	20.2971		----			
5.9483	4201	0.016	----	20.3397		----			
5.9768	29224	0.024	----	20.3792		----			
6.1013	1934	0.027	----	20.5513		----			
6.1514	7603	0.027	----	20.6205		----			
6.2088	2981	0.027	----	20.6999		----			
6.2775	8112	0.019	0.972	20.7948	21:1 w8c	0.32	ECL deviates -0.003		
6.3374	5338	0.026	----	20.8776		----			
6.3932	16178	0.021	0.973	20.9547	21:1 w3c	0.63	ECL deviates  0.001		
6.4283	5026	0.021	0.973	21.0032	21:0	0.20	ECL deviates  0.003	Reference  0.002	
6.5103	2512	0.018	----	21.1162		----			
6.5953	2452	0.021	0.974	21.2332	22:5 w6c	0.10	ECL deviates -0.019		
6.6286	4423	0.021	----	21.2790		----			
6.8769	6942	0.027	0.974	21.6210	22:0 iso	0.27	ECL deviates  0.003		
6.9530	1356	0.020	0.974	21.7258	22:2 w6c	0.05	ECL deviates -0.013		
6.9884	1216	0.019	0.974	21.7746	22:1 w9c	0.05	ECL deviates  0.002		
7.0241	2216	0.019	----	21.8236		----			
7.1059	3531	0.016	0.974	21.9363	22:1 w3c	0.14	ECL deviates -0.011		
7.1516	15186	0.021	0.974	21.9993	22:0	0.59	ECL deviates -0.001	Reference -0.002	
7.2142	866	0.019	----	22.0870		----			
7.3256	9299	0.020	----	22.2430		----			
7.3761	1071	0.024	----	22.3137		----			
7.4412	945	0.020	----	22.4048		----			
7.6062	2211	0.034	0.971	22.6359	23:3 w3c	0.09	ECL deviates -0.009		
7.7048	2535	0.023	----	22.7739		----			
7.7662	1029	0.020	----	22.8600		----			
7.8096	8509	0.020	0.969	22.9207	23:1 w4c	0.33	ECL deviates -0.006		
7.8665	3372	0.018	0.968	23.0004	23:0	0.13	ECL deviates  0.000	Reference -0.002	
7.9153	1175	0.028	----	23.0696		----			
8.0732	4637	0.019	----	23.2934		----			
8.3237	4984	0.023	0.960	23.6485	24:3 w3c	0.19	ECL deviates -0.006		
8.3812	1304	0.021	----	23.7299		----			
8.4118	1791	0.020	----	23.7733		----			
8.4890	2295	0.034	----	23.8827		----			
8.5699	15016	0.018	0.954	23.9975	24:0	0.57	ECL deviates -0.003	Reference -0.006	
8.6725	926	0.018	----	24.1429		----	> max rt		
8.9241	18692	0.019	----	24.4995		----	> max rt		
9.2273	19974	0.025	----	24.9294		----	> max rt		
9.4659	8627	0.020	----	25.2677		----	> max rt		

ECL Deviation: 0.007                            Reference ECL Shift: 0.004       Number Reference Peaks: 24
Total Response: 2868017                       Total Named: 2567742
Percent Named: 89.53%                         Total Amount: 2502285
Profile Comment:   Review report comments.

(No search libraries specified in method PLFAD1.)
