# Supplementary material for: Microbial regulation of soil carbon properties under nitrogen addition and plant inputs removal
Source: PeerJ. 2019 Jul 17;7:e7343. doi: 10.7717/peerj.7343 (PMC6642627; doi:10.7717/peerj.7343)
Supplement: File S1 — The raw data showed the soil microbial PLFAs files in the year of 2015 and 2016. Each file of rtf. represented the microbial PLFAs for each soil sample. In the Supplemental File, the Excel file named “Numbers” showed the plots names and the related rtf. file names. [file peerj-07-7343-s002.zip › supplementary files/2015/35.rtf]

Volume: DATA            File: E164213.59A        Samp Ctr: 4                  ID Number: 29332 
Type: Samp                   Bottle: 3                        Method: PLFAD1 
Created: 4/21/2016 10:02:24 AM 
Sample ID: 35 


RT	Response	Ar/Ht	RFact	ECL	Peak Name	Percent	Comment1	Comment2	
0.7146	1.899E+9	0.015	----	7.6468	SOLVENT PEAK	----	< min rt		
0.8861	518	0.009	----	8.7663		----	< min rt		
1.1866	2507	0.012	----	10.7290		----			
1.2629	435	0.011	----	11.1665		----			
1.3906	553	0.011	----	11.7758		----			
1.4373	1416	0.012	1.127	11.9983	12:0	0.05	ECL deviates -0.002	Reference -0.003	
1.4952	1022	0.012	----	12.2070		----			
1.5603	597	0.012	----	12.4408		----			
1.6057	2351	0.012	1.085	12.6037	13:0 iso	0.08	ECL deviates -0.009	Reference -0.010	
1.6382	2194	0.016	1.079	12.7206	13:0 anteiso	0.07	ECL deviates  0.011	Reference  0.010	
1.6904	732	0.017	----	12.9081		----			
1.7157	1003	0.013	1.062	12.9990	13:0	0.03	ECL deviates -0.001	Reference -0.002	
1.8744	1737	0.018	----	13.4420		----			
1.9341	49495	0.013	1.032	13.6087	14:0 iso	1.51	ECL deviates -0.005	Reference -0.006	
1.9744	983	0.013	1.028	13.7210	14:0 anteiso	0.03	ECL deviates  0.005	Reference  0.004	
1.9955	1021	0.009	1.025	13.7797	14:1 w9c	0.03	ECL deviates  0.002		
2.0088	1750	0.014	----	13.8168		----			
2.0424	2071	0.010	1.020	13.9106	14:1 w5c	0.06	ECL deviates  0.000		
2.0740	39429	0.014	1.016	13.9989	14:0	1.18	ECL deviates -0.001	Reference -0.002	
2.1025	667	0.012	----	14.0636		----			
2.1294	1240	0.014	----	14.1242	14:0 iso 3OH	----	ECL deviates -0.001		
2.1544	2416	0.024	----	14.1808		----			
2.2217	2181	0.020	----	14.3329		----			
2.2678	43971	0.018	1.001	14.4370	15:1 iso w6c	1.30	ECL deviates -0.002		
2.2865	7604	0.011	0.999	14.4792	15:4 w3c	0.22	ECL deviates -0.011		
2.3081	11135	0.014	0.998	14.5280	15:1 anteiso w9c	0.33	ECL deviates -0.002		
2.3467	188682	0.014	0.996	14.6151	15:0 iso	5.54	ECL deviates -0.002	Reference -0.002	
2.3887	143836	0.014	0.993	14.7099	15:0 anteiso	4.21	ECL deviates -0.001	Reference -0.001	
2.4537	7943	0.025	0.989	14.8567	15:1 w6c	0.23	ECL deviates -0.003		
2.5183	21223	0.015	0.985	15.0023	15:0	0.62	ECL deviates  0.002	Reference  0.002	
2.5465	8772	0.018	----	15.0559		----			
2.6081	2037	0.021	----	15.1732		----			
2.6386	2923	0.021	----	15.2315		----			
2.7245	5512	0.014	0.977	15.3950	16:1 w7c alcohol	0.16	ECL deviates -0.002		
2.7500	33729	0.020	0.976	15.4435	15:0 DMA	0.97	ECL deviates -0.007		
2.8101	68903	0.016	0.974	15.5579	16:0 N alcohol	1.98	ECL deviates  0.001		
2.8431	81120	0.015	0.973	15.6208	16:0 iso	2.33	ECL deviates  0.001	Reference  0.001	
2.8954	8229	0.013	0.971	15.7203	16:0 anteiso	0.24	ECL deviates  0.005	Reference  0.005	
2.9219	47295	0.017	0.971	15.7708	16:1 w9c	1.35	ECL deviates -0.004		
2.9501	389728	0.017	0.970	15.8245	16:1 w7c	11.15	ECL deviates  0.000		
2.9975	105989	0.016	0.969	15.9149	16:1 w5c	3.03	ECL deviates  0.004		
3.0468	373237	0.016	0.968	16.0075	16:0	10.65	ECL deviates  0.008	Reference  0.008	
3.0743	20740	0.021	----	16.0536		----			
3.1264	2662	0.017	0.966	16.1408	16:2 DMA	0.08	ECL deviates  0.003		
3.1614	6155	0.022	----	16.1994		----			
3.1988	3346	0.020	----	16.2620		----			
3.2342	2030	0.018	0.964	16.3212	16:1 w7c DMA	0.06	ECL deviates  0.011		
3.2957	196683	0.020	0.963	16.4242	16:0 10-methyl	5.59	ECL deviates  0.004		
3.3316	40106	0.017	0.963	16.4844	17:1 iso w9c	1.14	ECL deviates -0.014		
3.3595	23862	0.018	0.962	16.5310	17:1 anteiso w9c	0.68	ECL deviates -0.005		
3.4140	46852	0.017	0.962	16.6222	17:0 iso	1.33	ECL deviates -0.001	Reference -0.001	
3.4729	54956	0.017	0.961	16.7209	17:0 anteiso	1.56	ECL deviates  0.001		
3.5157	33480	0.019	0.961	16.7925	17:1 w8c	0.95	ECL deviates -0.004		
3.5760	114246	0.018	0.960	16.8936	17:0 cyclo w7c	3.23	ECL deviates  0.000		
3.6414	15329	0.018	0.960	17.0026	17:0	0.43	ECL deviates  0.003	Reference  0.003	
3.6672	21106	0.017	0.959	17.0421	17:1 w7c 10-methyl	0.60	ECL deviates -0.001		
3.7098	5829	0.017	----	17.1072		----			
3.7447	1393	0.017	----	17.1605		----			
3.7944	2980	0.020	0.959	17.2365	16:0 2OH	0.08	ECL deviates -0.004		
3.9050	20559	0.019	0.959	17.4054	17:0 10-methyl	0.58	ECL deviates -0.002		
3.9406	2446	0.013	0.959	17.4597	17:0 DMA	0.07	ECL deviates  0.002		
3.9635	6292	0.023	----	17.4948		----			
4.0372	28356	0.031	0.959	17.6074	18:0 iso	0.80	ECL deviates -0.019	Reference -0.019	
4.1117	92594	0.016	0.959	17.7212	18:2 w6c	2.62	ECL deviates -0.006		
4.1458	240911	0.020	0.959	17.7732	18:1 w9c	6.81	ECL deviates -0.001		
4.1822	367493	0.017	0.959	17.8289	18:1 w7c	10.39	ECL deviates  0.002		
4.2376	47682	0.022	0.959	17.9135	18:1 w5c	1.35	ECL deviates -0.009		
4.2955	57089	0.018	0.959	18.0018	18:0	1.61	ECL deviates  0.002	Reference  0.002	
4.3505	21417	0.019	0.959	18.0814	18:1 w7c 10-methyl	0.61	ECL deviates -0.004		
4.4063	6822	0.026	0.959	18.1621	18:2 DMA	0.19	ECL deviates  0.002		
4.4511	4069	0.022	0.960	18.2268	18:1 w9c DMA	0.12	ECL deviates -0.010		
4.4859	1441	0.015	0.960	18.2771	18:1 w7c DMA	0.04	ECL deviates -0.005		
4.5114	1799	0.019	----	18.3139		----			
4.5634	92575	0.019	0.960	18.3892	18:0 10-methyl	2.62	ECL deviates -0.006		
4.6326	2630	0.020	0.960	18.4892	19:4 w6c	0.07	ECL deviates  0.004		
4.6804	7066	0.025	0.961	18.5583	19:3 w6c	0.20	ECL deviates -0.002		
4.7482	4431	0.028	0.961	18.6564	19:3 w3c	0.13	ECL deviates -0.002		
4.8092	10968	0.020	----	18.7445		----			
4.8537	11316	0.020	0.962	18.8089	19:1 w8c	0.32	ECL deviates -0.002		
4.8925	16490	0.016	0.962	18.8649	19:0 cyclo w9c	0.47	ECL deviates -0.007		
4.9178	81698	0.019	0.962	18.9016	19:0 cyclo w7c	2.32	ECL deviates -0.008		
4.9889	71935	0.020	----	19.0041	19:0	----	ECL deviates  0.004		
5.0491	1867	0.016	----	19.0881		----			
5.1430	1675	0.021	----	19.2190		----			
5.1764	8175	0.019	----	19.2655		----			
5.2641	22940	0.028	0.965	19.3878	20:4 w6c	0.65	ECL deviates -0.016		
5.3166	10333	0.021	0.966	19.4610	20:5 w3c	0.29	ECL deviates -0.021		
5.3512	1899	0.015	----	19.5092		----			
5.3839	4848	0.020	----	19.5549		----			
5.4155	8477	0.026	----	19.5989		----			
5.5331	21084	0.028	0.967	19.7628	20:1 w9c	0.60	ECL deviates -0.010		
5.5627	9212	0.022	0.967	19.8041	20:1 w8c	0.26	ECL deviates -0.009		
5.6144	740	0.016	----	19.8761		----			
5.7032	17868	0.021	0.969	19.9999	20:0	0.51	ECL deviates  0.000	Reference  0.001	
5.7602	738	0.018	----	20.0787		----			
5.8065	2060	0.016	----	20.1427		----			
5.8356	6100	0.019	----	20.1828		----			
5.9505	7331	0.023	----	20.3416		----			
5.9795	31945	0.023	----	20.3817		----			
6.0515	1033	0.017	----	20.4811		----			
6.1048	2573	0.028	----	20.5547		----			
6.1525	7240	0.027	----	20.6207		----			
6.2140	3273	0.028	----	20.7057		----			
6.2808	11037	0.021	0.972	20.7979	21:1 w8c	0.32	ECL deviates  0.000		
6.3388	8190	0.021	----	20.8781		----			
6.3975	19327	0.021	0.973	20.9591	21:1 w3c	0.55	ECL deviates  0.005		
6.4320	5066	0.020	0.973	21.0068	21:0	0.15	ECL deviates  0.007	Reference  0.007	
6.5102	3192	0.018	----	21.1145		----			
6.5983	4016	0.024	0.974	21.2356	22:5 w6c	0.12	ECL deviates -0.016		
6.6291	7004	0.024	----	21.2780		----			
6.7581	1666	0.021	0.974	21.4557	22:5 w3c	0.05	ECL deviates -0.012		
6.8794	9605	0.030	0.974	21.6225	22:0 iso	0.28	ECL deviates  0.005		
6.9561	2975	0.025	0.974	21.7281	22:2 w6c	0.09	ECL deviates -0.011		
6.9896	2262	0.021	0.974	21.7743	22:1 w9c	0.06	ECL deviates  0.001		
7.0251	3100	0.026	0.974	21.8231	22:1 w8c	0.09	ECL deviates  0.010		
7.1065	4806	0.019	0.974	21.9351	22:1 w3c	0.14	ECL deviates -0.012		
7.1520	18716	0.017	0.974	21.9978	22:0	0.54	ECL deviates -0.002	Reference -0.002	
7.2130	1846	0.025	----	22.0831		----			
7.3261	8488	0.019	----	22.2413		----			
7.6047	2107	0.035	----	22.6312		----			
7.7082	2982	0.020	----	22.7760		----			
7.7667	914	0.019	----	22.8580		----			
7.8111	7861	0.018	0.969	22.9201	23:1 w4c	0.22	ECL deviates -0.006		
7.8695	3818	0.017	0.968	23.0019	23:0	0.11	ECL deviates  0.002	Reference  0.002	
7.9156	1022	0.018	----	23.0672		----			
8.0763	4846	0.019	----	23.2947		----			
8.3261	4999	0.023	0.960	23.6485	24:3 w3c	0.14	ECL deviates -0.006		
8.3855	996	0.020	----	23.7327		----			
8.4156	1175	0.017	----	23.7753		----			
8.5730	16646	0.019	0.954	23.9982	24:0	0.47	ECL deviates -0.002	Reference -0.002	
8.6782	804	0.018	----	24.1472		----	> max rt		
8.9284	8157	0.021	----	24.5016		----	> max rt		
9.2312	15861	0.026	----	24.9305		----	> max rt		
9.4673	7639	0.020	----	25.2649		----	> max rt		

ECL Deviation: 0.007                            Reference ECL Shift: 0.006       Number Reference Peaks: 22
Total Response: 3714398                       Total Named: 3496812
Percent Named: 94.14%                         Total Amount: 3391159

(No search libraries specified in method PLFAD1.)
