# Supplementary material for: Microbial regulation of soil carbon properties under nitrogen addition and plant inputs removal
Source: PeerJ. 2019 Jul 17;7:e7343. doi: 10.7717/peerj.7343 (PMC6642627; doi:10.7717/peerj.7343)
Supplement: File S1 — The raw data showed the soil microbial PLFAs files in the year of 2015 and 2016. Each file of rtf. represented the microbial PLFAs for each soil sample. In the Supplemental File, the Excel file named “Numbers” showed the plots names and the related rtf. file names. [file peerj-07-7343-s002.zip › supplementary files/2015/36.rtf]

Volume: DATA            File: E164213.59A        Samp Ctr: 5                  ID Number: 29333 
Type: Samp                   Bottle: 4                        Method: PLFAD1 
Created: 4/21/2016 10:31:12 AM 
Sample ID: 36 


RT	Response	Ar/Ht	RFact	ECL	Peak Name	Percent	Comment1	Comment2	
0.7146	1.896E+9	0.014	----	7.6496	SOLVENT PEAK	----	< min rt		
0.8867	553	0.009	----	8.7732		----	< min rt		
0.9450	695	0.011	----	9.1590		----	< min rt		
1.1866	3891	0.012	----	10.7334		----			
1.2626	1006	0.014	----	11.1694		----			
1.3171	771	0.014	1.172	11.4292	10:0 3OH	0.02	ECL deviates -0.012		
1.3535	1271	0.017	1.157	11.6027	12:0 iso	0.03	ECL deviates -0.009		
1.3650	507	0.008	----	11.6573		----			
1.3905	2887	0.016	----	11.7790		----			
1.4370	4267	0.014	1.127	12.0007	12:0	0.11	ECL deviates  0.001	Reference -0.005	
1.4560	331	0.008	----	12.0701		----			
1.4948	3746	0.016	----	12.2094		----			
1.5201	695	0.011	----	12.3000		----			
1.5594	2250	0.020	----	12.4413		----			
1.6056	4917	0.013	1.085	12.6070	13:0 iso	0.12	ECL deviates -0.005	Reference -0.010	
1.6358	3873	0.017	1.079	12.7155	13:0 anteiso	0.10	ECL deviates  0.006	Reference  0.001	
1.6906	1604	0.018	1.067	12.9121	13:1 w5c	0.04	ECL deviates -0.008		
1.7150	1861	0.013	1.062	12.9996	13:0	0.05	ECL deviates  0.000	Reference -0.005	
1.7834	1033	0.018	----	13.1914	12:0 2OH	----	ECL deviates  0.005		
1.8744	2372	0.018	----	13.4451		----			
1.9336	70063	0.013	1.032	13.6101	14:0 iso	1.68	ECL deviates -0.004	Reference -0.008	
1.9731	1478	0.014	1.028	13.7203	14:0 anteiso	0.04	ECL deviates  0.004	Reference  0.001	
1.9934	1176	0.010	1.025	13.7769	14:1 w9c	0.03	ECL deviates -0.001		
2.0086	2144	0.014	----	13.8192		----			
2.0731	54558	0.014	1.016	13.9991	14:0	1.29	ECL deviates -0.001	Reference -0.004	
2.1017	935	0.013	----	14.0646		----			
2.1298	1524	0.015	----	14.1280	14:0 iso 3OH	----	ECL deviates  0.003		
2.1535	3770	0.023	----	14.1814		----			
2.2210	3372	0.020	----	14.3339		----			
2.2666	58265	0.017	1.001	14.4367	15:1 iso w6c	1.35	ECL deviates -0.002		
2.2849	9832	0.012	0.999	14.4782	15:4 w3c	0.23	ECL deviates -0.012		
2.3070	14279	0.014	0.998	14.5280	15:1 anteiso w9c	0.33	ECL deviates -0.002		
2.3461	284110	0.015	0.996	14.6163	15:0 iso	6.56	ECL deviates -0.001	Reference -0.004	
2.3874	202934	0.014	0.993	14.7096	15:0 anteiso	4.67	ECL deviates -0.001	Reference -0.004	
2.4515	9314	0.024	0.989	14.8544	15:1 w6c	0.21	ECL deviates -0.006		
2.5164	27393	0.015	0.985	15.0009	15:0	0.63	ECL deviates  0.001	Reference -0.002	
2.5445	11084	0.018	----	15.0547		----			
2.6065	2535	0.020	----	15.1727		----			
2.6368	3459	0.019	----	15.2304		----			
2.7222	7886	0.015	0.977	15.3930	16:1 w7c alcohol	0.18	ECL deviates -0.004		
2.7484	44814	0.021	0.976	15.4429	15:0 DMA	1.01	ECL deviates -0.008		
2.8083	80407	0.015	0.974	15.5568	16:0 N alcohol	1.82	ECL deviates  0.000		
2.8412	112334	0.015	0.973	15.6195	16:0 iso	2.53	ECL deviates  0.000	Reference -0.002	
2.8924	9981	0.013	0.971	15.7170	16:0 anteiso	0.22	ECL deviates  0.002	Reference  0.000	
2.9187	58998	0.017	0.971	15.7670	16:1 w9c	1.33	ECL deviates -0.008		
2.9493	446114	0.017	0.970	15.8253	16:1 w7c	10.03	Column Overload		
2.9957	133527	0.016	0.969	15.9136	16:1 w5c	3.00	ECL deviates  0.003		
3.0452	480485	0.015	0.968	16.0071	16:0	10.78	Column Overload		
3.0723	25992	0.020	----	16.0524		----			
3.1244	3851	0.017	0.966	16.1398	16:2 DMA	0.09	ECL deviates  0.002		
3.1599	8235	0.022	----	16.1990		----			
3.1972	4439	0.020	----	16.2615		----			
3.2304	2534	0.021	0.964	16.3171	16:1 w7c DMA	0.06	ECL deviates  0.007		
3.2944	278711	0.020	0.963	16.4242	16:0 10-methyl	6.22	ECL deviates  0.004		
3.3298	50832	0.019	0.963	16.4834	17:1 iso w9c	1.13	ECL deviates -0.015		
3.3566	31249	0.018	0.962	16.5283	17:1 anteiso w9c	0.70	ECL deviates -0.008		
3.4124	65450	0.016	0.962	16.6217	17:0 iso	1.46	ECL deviates -0.002	Reference -0.004	
3.4703	74361	0.018	0.961	16.7186	17:0 anteiso	1.66	ECL deviates -0.002		
3.5145	46677	0.017	0.961	16.7927	17:1 w8c	1.04	ECL deviates -0.004		
3.5748	158213	0.018	0.960	16.8936	17:0 cyclo w7c	3.52	ECL deviates  0.000		
3.6383	21032	0.018	0.960	16.9998	17:0	0.47	ECL deviates  0.000	Reference -0.002	
3.6648	28688	0.017	0.959	17.0404	17:1 w7c 10-methyl	0.64	ECL deviates -0.003		
3.7065	7384	0.017	----	17.1043		----			
3.7410	1777	0.017	----	17.1569		----			
3.7921	3669	0.020	0.959	17.2350	16:0 2OH	0.08	ECL deviates -0.005		
3.9022	30079	0.018	0.959	17.4032	17:0 10-methyl	0.67	ECL deviates -0.004		
3.9385	3123	0.012	0.959	17.4586	17:0 DMA	0.07	ECL deviates  0.000		
3.9594	8750	0.020	----	17.4906		----			
4.0347	30758	0.030	----	17.6055		----			
4.1096	88999	0.017	0.959	17.7200	18:2 w6c	1.98	ECL deviates -0.007		
4.1443	297770	0.018	0.959	17.7730	18:1 w9c	6.62	ECL deviates -0.002		
4.1810	440121	0.018	0.959	17.8290	18:1 w7c	9.78	Column Overload		
4.2363	60305	0.024	0.959	17.9136	18:1 w5c	1.34	ECL deviates -0.009		
4.2938	75968	0.019	0.959	18.0013	18:0	1.69	ECL deviates  0.001	Reference  0.000	
4.3484	28112	0.019	0.959	18.0803	18:1 w7c 10-methyl	0.63	ECL deviates -0.005		
4.4005	5834	0.019	0.959	18.1556	18:2 DMA	0.13	ECL deviates -0.004		
4.4153	3407	0.011	----	18.1770		----			
4.4493	4496	0.021	0.960	18.2262	18:1 w9c DMA	0.10	ECL deviates -0.011		
4.4791	2483	0.018	0.960	18.2694	18:1 w7c DMA	0.06	ECL deviates -0.013		
4.5077	1736	0.016	----	18.3106		----			
4.5613	126679	0.021	0.960	18.3882	18:0 10-methyl	2.82	ECL deviates -0.007		
4.6338	3270	0.019	0.960	18.4930	19:4 w6c	0.07	ECL deviates  0.008		
4.6764	9075	0.028	0.961	18.5546	19:3 w6c	0.20	ECL deviates -0.005		
4.7451	5372	0.027	0.961	18.6540	19:3 w3c	0.12	ECL deviates -0.004		
4.8075	14947	0.021	----	18.7441		----			
4.8536	13828	0.019	0.962	18.8108	19:1 w8c	0.31	ECL deviates  0.000		
4.8905	23669	0.017	0.962	18.8642	19:0 cyclo w9c	0.53	ECL deviates -0.008		
4.9156	107909	0.018	0.962	18.9004	19:0 cyclo w7c	2.41	ECL deviates -0.009		
4.9860	72776	0.019	----	19.0022	19:0	----	ECL deviates  0.002		
5.0474	2627	0.019	----	19.0878		----			
5.1377	2144	0.019	----	19.2137		----			
5.1728	11506	0.018	----	19.2626		----			
5.2578	26708	0.031	----	19.3812		----			
5.3132	7078	0.020	0.966	19.4584	20:5 w3c	0.16	ECL deviates -0.024		
5.3491	2504	0.016	----	19.5085		----			
5.3802	5735	0.022	----	19.5518		----			
5.4131	10325	0.026	----	19.5978		----			
5.5317	27619	0.027	0.967	19.7630	20:1 w9c	0.62	ECL deviates -0.010		
5.5599	12022	0.021	0.967	19.8023	20:1 w8c	0.27	ECL deviates -0.011		
5.6135	666	0.012	----	19.8771		----			
5.7002	24351	0.023	0.969	19.9980	20:0	0.55	ECL deviates -0.002	Reference -0.004	
5.7577	1376	0.019	----	20.0775		----			
5.8019	2774	0.016	----	20.1386		----			
5.8336	7000	0.020	----	20.1825		----			
5.9157	3981	0.018	----	20.2958		----			
5.9451	5914	0.015	----	20.3365		----			
5.9766	39466	0.022	----	20.3800		----			
6.0782	1615	0.017	----	20.5205		----			
6.1019	2140	0.017	----	20.5532		----			
6.1495	6557	0.020	----	20.6190		----			
6.1689	2919	0.016	0.972	20.6458	21:3 w3c	0.07	ECL deviates -0.008		
6.2109	4476	0.029	----	20.7038		----			
6.2768	14886	0.020	0.972	20.7949	21:1 w8c	0.34	ECL deviates -0.003		
6.3354	9743	0.023	----	20.8759		----			
6.3945	23518	0.019	0.973	20.9576	21:1 w3c	0.53	ECL deviates  0.004		
6.4269	6951	0.022	0.973	21.0024	21:0	0.16	ECL deviates  0.002	Reference  0.000	
6.5108	4402	0.021	----	21.1179		----			
6.5943	4856	0.027	0.974	21.2329	22:5 w6c	0.11	ECL deviates -0.019		
6.6272	9151	0.021	----	21.2782		----			
6.6939	723	0.015	----	21.3700		----			
6.8774	11779	0.030	0.974	21.6226	22:0 iso	0.27	ECL deviates  0.005		
6.9535	3382	0.025	0.974	21.7274	22:2 w6c	0.08	ECL deviates -0.011		
6.9892	2926	0.020	0.974	21.7766	22:1 w9c	0.07	ECL deviates  0.004		
7.0232	4764	0.025	0.974	21.8234	22:1 w8c	0.11	ECL deviates  0.010		
7.1053	6773	0.018	0.974	21.9364	22:1 w3c	0.15	ECL deviates -0.011		
7.1507	24581	0.019	0.974	21.9989	22:0	0.56	ECL deviates -0.001	Reference -0.004	
7.2140	1476	0.019	----	22.0875		----			
7.2429	892	0.020	----	22.1279		----			
7.3255	9872	0.020	----	22.2435		----			
7.3783	1177	0.024	----	22.3175		----			
7.4416	1414	0.019	----	22.4062		----			
7.4987	1151	0.024	0.972	22.4860	23:4 w6c	0.03	ECL deviates  0.015		
7.5355	828	0.019	----	22.5375		----			
7.6091	3447	0.041	0.971	22.6407	23:3 w3c	----	> max ar/ht		
7.7044	4085	0.023	----	22.7742		----			
7.7667	1720	0.024	----	22.8614		----			
7.8088	10930	0.020	0.969	22.9202	23:1 w4c	0.25	ECL deviates -0.006		
7.8672	5359	0.020	0.968	23.0020	23:0	0.12	ECL deviates  0.002	Reference -0.001	
7.9135	1752	0.022	----	23.0676		----			
8.0729	6903	0.020	----	23.2936		----			
8.3225	7117	0.028	0.960	23.6473	24:3 w3c	0.16	ECL deviates -0.007		
8.3813	2100	0.023	----	23.7306		----			
8.4147	2677	0.023	0.958	23.7779	24:1 w9c	0.06	ECL deviates -0.009		
8.4907	2028	0.023	----	23.8855		----			
8.5241	879	0.018	----	23.9330		----			
8.5704	19779	0.020	0.954	23.9985	24:0	0.44	ECL deviates -0.002	Reference -0.006	
8.6756	964	0.018	----	24.1476		----	> max rt		
8.9277	15567	0.019	----	24.5049		----	> max rt		
9.2284	19972	0.022	----	24.9311		----	> max rt		
9.4653	10074	0.021	----	25.2670		----	> max rt		

ECL Deviation: 0.007                            Reference ECL Shift: 0.004       Number Reference Peaks: 20
Total Response: 4796800                       Total Named: 4444287
Percent Named: 92.65%                         Total Amount: 4317481
Profile Comment:   Column Overload:  A peak's response is greater than 400000.0.  Dilute and re-run.

(No search libraries specified in method PLFAD1.)
