# Supplementary material for: Microbial regulation of soil carbon properties under nitrogen addition and plant inputs removal
Source: PeerJ. 2019 Jul 17;7:e7343. doi: 10.7717/peerj.7343 (PMC6642627; doi:10.7717/peerj.7343)
Supplement: File S1 — The raw data showed the soil microbial PLFAs files in the year of 2015 and 2016. Each file of rtf. represented the microbial PLFAs for each soil sample. In the Supplemental File, the Excel file named “Numbers” showed the plots names and the related rtf. file names. [file peerj-07-7343-s002.zip › supplementary files/2015/37.rtf]

Volume: DATA            File: E164213.59A        Samp Ctr: 6                  ID Number: 29334 
Type: Samp                   Bottle: 5                        Method: PLFAD1 
Created: 4/21/2016 10:59:53 AM 
Sample ID: 37 


RT	Response	Ar/Ht	RFact	ECL	Peak Name	Percent	Comment1	Comment2	
0.7146	1.897E+9	0.015	----	7.6392	SOLVENT PEAK	----	< min rt		
0.8862	427	0.008	----	8.7615		----	< min rt		
0.9447	1093	0.012	----	9.1443		----	< min rt		
0.9708	491	0.012	----	9.3152		----	< min rt		
1.1863	4341	0.012	1.242	10.7240	11:0 anteiso	0.14	ECL deviates  0.019	Reference  0.020	
1.2622	734	0.013	----	11.1606		----			
1.3527	827	0.017	----	11.5930	Phthalate 1	----	ECL deviates  0.006		
1.3650	332	0.008	----	11.6522		----			
1.3897	1256	0.015	----	11.7701		----			
1.4363	3484	0.014	1.127	11.9928	12:0	0.10	ECL deviates -0.007	Reference -0.008	
1.4942	2530	0.018	----	12.2030		----			
1.5593	1361	0.017	----	12.4372		----			
1.6047	3973	0.012	1.085	12.6006	13:0 iso	0.11	ECL deviates -0.012	Reference -0.013	
1.6365	3535	0.018	1.079	12.7149	13:0 anteiso	0.10	ECL deviates  0.006	Reference  0.004	
1.6903	841	0.013	----	12.9082		----			
1.7140	1980	0.013	1.062	12.9937	13:0	0.05	ECL deviates -0.006	Reference -0.008	
1.7829	653	0.016	----	13.1877	12:0 2OH	----	ECL deviates  0.002		
1.8738	1594	0.016	----	13.4418		----			
1.9324	51191	0.013	1.032	13.6056	14:0 iso	1.36	ECL deviates -0.009	Reference -0.011	
1.9730	765	0.009	1.028	13.7189	14:0 anteiso	0.02	ECL deviates  0.003	Reference  0.000	
1.9930	1107	0.011	1.025	13.7749	14:1 w9c	0.03	ECL deviates -0.003		
2.0081	1728	0.012	----	13.8169		----			
2.0413	2803	0.011	1.020	13.9098	14:1 w5c	0.07	ECL deviates -0.001		
2.0723	46527	0.014	1.016	13.9963	14:0	1.22	ECL deviates -0.004	Reference -0.007	
2.1011	629	0.011	----	14.0626		----			
2.1281	1467	0.015	----	14.1237	14:0 iso 3OH	----	ECL deviates -0.001		
2.1526	2755	0.023	----	14.1792		----			
2.2149	2191	0.020	----	14.3200		----			
2.2658	51106	0.018	1.001	14.4350	15:1 iso w6c	1.32	ECL deviates -0.004		
2.2847	9156	0.011	0.999	14.4778	15:4 w3c	0.24	ECL deviates -0.012		
2.3059	11161	0.014	0.998	14.5258	15:1 anteiso w9c	0.29	ECL deviates -0.004		
2.3449	232275	0.014	0.996	14.6139	15:0 iso	5.96	ECL deviates -0.003	Reference -0.006	
2.3867	171403	0.014	0.993	14.7084	15:0 anteiso	4.39	ECL deviates -0.003	Reference -0.006	
2.4517	8466	0.024	0.989	14.8555	15:1 w6c	0.22	ECL deviates -0.005		
2.5154	26558	0.015	0.985	14.9994	15:0	0.67	ECL deviates -0.001	Reference -0.004	
2.5435	10469	0.017	----	15.0537		----			
2.6058	1841	0.018	----	15.1723		----			
2.6355	2608	0.018	----	15.2290		----			
2.7220	6576	0.016	0.977	15.3938	16:1 w7c alcohol	0.17	ECL deviates -0.003		
2.7482	39065	0.022	0.976	15.4438	15:0 DMA	0.98	ECL deviates -0.007		
2.8082	77033	0.015	0.974	15.5581	16:0 N alcohol	1.93	ECL deviates  0.001		
2.8411	98450	0.016	0.973	15.6209	16:0 iso	2.47	ECL deviates  0.001	Reference -0.003	
2.8918	10261	0.015	0.971	15.7174	16:0 anteiso	0.26	ECL deviates  0.002	Reference -0.001	
2.9189	50660	0.016	0.971	15.7691	16:1 w9c	1.27	ECL deviates -0.006		
2.9485	385734	0.017	0.970	15.8255	16:1 w7c	9.65	ECL deviates  0.001		
2.9955	111142	0.016	0.969	15.9150	16:1 w5c	2.78	ECL deviates  0.004		
3.0449	414483	0.016	0.968	16.0086	16:0	10.34	Column Overload		
3.0721	22354	0.019	----	16.0540		----			
3.1249	3394	0.017	0.966	16.1425	16:2 DMA	0.08	ECL deviates  0.005		
3.1616	6667	0.024	----	16.2040		----			
3.1970	3764	0.019	----	16.2633		----			
3.2400	1877	0.018	----	16.3354		----			
3.2938	228532	0.021	0.963	16.4255	16:0 10-methyl	5.68	ECL deviates  0.006		
3.3306	46912	0.019	0.963	16.4871	17:1 iso w9c	1.16	ECL deviates -0.011		
3.3575	26649	0.018	0.962	16.5321	17:1 anteiso w9c	0.66	ECL deviates -0.004		
3.4135	55380	0.016	0.962	16.6259	17:0 iso	1.37	ECL deviates  0.002	Reference -0.002	
3.4706	65558	0.018	0.961	16.7215	17:0 anteiso	1.62	ECL deviates  0.001		
3.5151	41210	0.017	0.961	16.7961	17:1 w8c	1.02	ECL deviates -0.001		
3.5747	147663	0.018	0.960	16.8959	17:0 cyclo w7c	3.66	ECL deviates  0.002		
3.6389	19983	0.018	0.960	17.0034	17:0	0.49	ECL deviates  0.003	Reference -0.001	
3.6646	23110	0.016	0.959	17.0428	17:1 w7c 10-methyl	0.57	ECL deviates  0.000		
3.7077	6868	0.018	----	17.1087		----			
3.7449	1798	0.021	----	17.1655		----			
3.7929	3972	0.017	0.959	17.2389	16:0 2OH	0.10	ECL deviates -0.001		
3.9027	30280	0.017	0.959	17.4067	17:0 10-methyl	0.75	ECL deviates  0.000		
3.9390	2453	0.012	0.959	17.4621	17:0 DMA	0.06	ECL deviates  0.004		
3.9604	10200	0.021	----	17.4948		----			
4.0165	9495	0.016	0.959	17.5805	18:3 w6c	0.23	ECL deviates  0.000		
4.0351	22671	0.025	0.959	17.6090	18:0 iso	0.56	ECL deviates -0.018		
4.1108	102662	0.018	0.959	17.7246	18:2 w6c	2.54	ECL deviates -0.003		
4.1452	255307	0.020	0.959	17.7771	18:1 w9c	6.31	ECL deviates  0.003		
4.1810	384010	0.017	0.959	17.8319	18:1 w7c	9.49	ECL deviates  0.005		
4.2359	48346	0.022	0.959	17.9158	18:1 w5c	1.20	ECL deviates -0.007		
4.2943	67619	0.018	0.959	18.0050	18:0	1.67	ECL deviates  0.005	Reference  0.001	
4.3496	26527	0.018	0.959	18.0850	18:1 w7c 10-methyl	0.66	ECL deviates  0.000		
4.4031	9833	0.027	0.959	18.1623	18:2 DMA	0.24	ECL deviates  0.002		
4.4496	4698	0.021	0.960	18.2296	18:1 w9c DMA	0.12	ECL deviates -0.007		
4.4824	3294	0.018	0.960	18.2769	18:1 w7c DMA	0.08	ECL deviates -0.006		
4.5112	1909	0.016	----	18.3186		----			
4.5620	120128	0.020	0.960	18.3920	18:0 10-methyl	2.97	ECL deviates -0.003		
4.6302	2841	0.019	0.960	18.4906	19:4 w6c	0.07	ECL deviates  0.006		
4.6763	7468	0.026	0.961	18.5572	19:3 w6c	0.19	ECL deviates -0.003		
4.7461	5369	0.026	0.961	18.6582	19:3 w3c	0.13	ECL deviates  0.000		
4.8091	12273	0.023	----	18.7492		----			
4.8549	16822	0.021	0.962	18.8155	19:1 w8c	0.42	ECL deviates  0.005		
4.9179	132326	0.022	0.962	18.9065	19:0 cyclo w7c	3.28	ECL deviates -0.003		
4.9870	74700	0.020	----	19.0063	19:0	----	ECL deviates  0.006		
5.0484	2475	0.019	----	19.0920		----			
5.1415	2146	0.021	----	19.2217		----			
5.1753	8220	0.019	----	19.2688		----			
5.2614	24401	0.030	0.965	19.3888	20:4 w6c	0.61	ECL deviates -0.015		
5.3153	9283	0.021	0.966	19.4639	20:5 w3c	0.23	ECL deviates -0.018		
5.3469	1869	0.014	----	19.5080		----			
5.3808	7043	0.021	----	19.5551		----			
5.4147	9852	0.025	----	19.6025		----			
5.5329	24512	0.025	0.967	19.7671	20:1 w9c	0.61	ECL deviates -0.005		
5.5604	12593	0.022	0.967	19.8054	20:1 w8c	0.31	ECL deviates -0.008		
5.6134	613	0.014	----	19.8794		----			
5.7026	22335	0.021	0.969	20.0035	20:0	0.56	ECL deviates  0.004	Reference  0.000	
5.7541	839	0.019	----	20.0748		----			
5.8031	2562	0.015	----	20.1425		----			
5.8353	8607	0.021	----	20.1868		----			
5.9483	10024	0.027	----	20.3429		----			
5.9785	34351	0.023	0.971	20.3846	20:0 10-methyl	0.86	ECL deviates -0.012		
6.0498	1300	0.017	----	20.4830		----			
6.0793	1150	0.012	----	20.5238		----			
6.1039	2952	0.020	----	20.5577		----			
6.1504	8151	0.029	----	20.6220		----			
6.2117	4383	0.027	----	20.7066		----			
6.2779	14457	0.019	0.972	20.7979	21:1 w8c	0.36	ECL deviates  0.000		
6.3372	8854	0.024	----	20.8798		----			
6.3937	23623	0.021	0.973	20.9579	21:1 w3c	0.59	ECL deviates  0.004		
6.4300	6571	0.021	0.973	21.0080	21:0	0.16	ECL deviates  0.008	Reference  0.005	
6.5093	4565	0.019	----	21.1170		----			
6.5952	4793	0.026	0.974	21.2352	22:5 w6c	0.12	ECL deviates -0.017		
6.6299	7276	0.023	----	21.2829		----			
6.7577	1159	0.027	0.974	21.4586	22:5 w3c	0.03	ECL deviates -0.009		
6.8422	1360	0.020	----	21.5748		----			
6.8785	8129	0.025	0.974	21.6247	22:0 iso	0.20	ECL deviates  0.007		
6.9556	2414	0.026	0.974	21.7307	22:2 w6c	0.06	ECL deviates -0.008		
6.9895	3361	0.022	0.974	21.7774	22:1 w9c	0.08	ECL deviates  0.004		
7.0259	3145	0.023	----	21.8273		----			
7.1067	6411	0.018	0.974	21.9386	22:1 w3c	0.16	ECL deviates -0.008		
7.1494	22199	0.017	0.974	21.9973	22:0	0.56	ECL deviates -0.003	Reference -0.005	
7.2138	1078	0.016	----	22.0872		----			
7.2422	1451	0.024	----	22.1269		----			
7.3243	11023	0.020	----	22.2417		----			
7.3779	1549	0.025	----	22.3166		----			
7.4420	1376	0.023	----	22.4061		----			
7.4978	1202	0.019	0.972	22.4842	23:4 w6c	0.03	ECL deviates  0.013		
7.5357	820	0.019	----	22.5371		----			
7.6008	4126	0.042	----	22.6281		----	> max ar/ht		
7.7051	5117	0.022	----	22.7740		----			
7.7654	1986	0.022	----	22.8582		----			
7.8076	10385	0.022	0.969	22.9172	23:1 w4c	0.26	ECL deviates -0.009		
7.8663	5292	0.018	0.968	22.9992	23:0	0.13	ECL deviates -0.001	Reference -0.003	
7.9117	1794	0.024	----	23.0635		----			
8.0722	5903	0.020	----	23.2905		----			
8.2845	939	0.017	0.961	23.5907	24:3 w6c	0.02	ECL deviates  0.000		
8.3228	5905	0.025	----	23.6448		----			
8.3797	2140	0.023	----	23.7252		----			
8.4131	2552	0.019	----	23.7726		----			
8.4902	1980	0.025	----	23.8815		----			
8.5227	903	0.019	----	23.9274		----			
8.5700	20387	0.022	0.954	23.9943	24:0	0.50	ECL deviates -0.006	Reference -0.006	
8.6745	1193	0.018	----	24.1421		----	> max rt		
8.9283	7686	0.021	----	24.5011		----	> max rt		
9.2279	18946	0.022	----	24.9246		----	> max rt		
9.4661	9090	0.021	----	25.2614		----	> max rt		

ECL Deviation: 0.007                            Reference ECL Shift: 0.007       Number Reference Peaks: 21
Total Response: 4242218                       Total Named: 3996545
Percent Named: 94.21%                         Total Amount: 3878044
Profile Comment:   Column Overload:  A peak's response is greater than 400000.0.  Dilute and re-run.

(No search libraries specified in method PLFAD1.)
