# Supplementary material for: Microbial regulation of soil carbon properties under nitrogen addition and plant inputs removal
Source: PeerJ. 2019 Jul 17;7:e7343. doi: 10.7717/peerj.7343 (PMC6642627; doi:10.7717/peerj.7343)
Supplement: File S1 — The raw data showed the soil microbial PLFAs files in the year of 2015 and 2016. Each file of rtf. represented the microbial PLFAs for each soil sample. In the Supplemental File, the Excel file named “Numbers” showed the plots names and the related rtf. file names. [file peerj-07-7343-s002.zip › supplementary files/2015/38.rtf]

Volume: DATA            File: E164213.59A        Samp Ctr: 7                  ID Number: 29335 
Type: Samp                   Bottle: 6                        Method: PLFAD1 
Created: 4/21/2016 11:28:39 AM 
Sample ID: 38 


RT	Response	Ar/Ht	RFact	ECL	Peak Name	Percent	Comment1	Comment2	
0.7146	1.905E+9	0.014	----	7.6465	SOLVENT PEAK	----	< min rt		
0.8871	658	0.011	----	8.7722		----	< min rt		
0.9453	762	0.011	----	9.1587		----	< min rt		
1.1875	3880	0.012	----	10.7371		----			
1.2641	1075	0.013	1.195	11.1741	10:0 2OH	0.03	ECL deviates -0.010		
1.3186	782	0.016	1.172	11.4341	10:0 3OH	0.02	ECL deviates -0.007		
1.3538	1128	0.017	1.157	11.6017	12:0 iso	0.03	ECL deviates -0.010		
1.3667	424	0.008	----	11.6633		----			
1.3913	1294	0.014	----	11.7804		----			
1.4377	4013	0.015	1.127	12.0018	12:0	0.10	ECL deviates  0.002	Reference -0.002	
1.4955	2875	0.017	----	12.2098		----			
1.5231	459	0.011	----	12.3085		----			
1.5605	1873	0.017	----	12.4429		----			
1.6062	4489	0.012	1.085	12.6071	13:0 iso	0.11	ECL deviates -0.005	Reference -0.008	
1.6376	3460	0.016	1.079	12.7198	13:0 anteiso	0.09	ECL deviates  0.010	Reference  0.008	
1.6900	1086	0.016	----	12.9076		----			
1.7156	2479	0.014	1.062	12.9996	13:0	0.06	ECL deviates  0.000	Reference -0.003	
1.7415	526	0.013	----	13.0725		----			
1.7827	710	0.015	----	13.1873	12:0 2OH	----	ECL deviates  0.001		
1.8252	418	0.010	----	13.3057		----			
1.8746	2183	0.019	----	13.4435		----			
1.9340	53116	0.013	1.032	13.6093	14:0 iso	1.26	ECL deviates -0.005	Reference -0.007	
1.9737	1229	0.013	1.028	13.7200	14:0 anteiso	0.03	ECL deviates  0.004	Reference  0.002	
1.9949	1415	0.012	1.025	13.7790	14:1 w9c	0.03	ECL deviates  0.002		
2.0086	1880	0.012	----	13.8172		----			
2.0412	2921	0.013	1.020	13.9082	14:1 w5c	0.07	ECL deviates -0.003		
2.0737	55062	0.014	1.016	13.9990	14:0	1.28	ECL deviates -0.001	Reference -0.002	
2.1009	758	0.010	----	14.0608		----			
2.1299	1563	0.015	----	14.1262	14:0 iso 3OH	----	ECL deviates  0.001		
2.1539	2711	0.023	----	14.1805		----			
2.2143	2550	0.020	----	14.3168		----			
2.2670	56731	0.018	1.001	14.4359	15:1 iso w6c	1.30	ECL deviates -0.003		
2.2861	10620	0.012	0.999	14.4789	15:4 w3c	0.24	ECL deviates -0.011		
2.3073	14010	0.014	0.998	14.5269	15:1 anteiso w9c	0.32	ECL deviates -0.003		
2.3464	269793	0.014	0.996	14.6152	15:0 iso	6.15	ECL deviates -0.002	Reference -0.003	
2.3877	206858	0.014	0.993	14.7086	15:0 anteiso	4.70	ECL deviates -0.002	Reference -0.003	
2.4529	8550	0.021	0.989	14.8558	15:1 w6c	0.19	ECL deviates -0.004		
2.4722	1951	0.013	0.988	14.8993	15:1 w5c	0.04	ECL deviates -0.013		
2.5163	31131	0.014	0.985	14.9989	15:0	0.70	ECL deviates -0.001	Reference -0.002	
2.5442	13095	0.017	----	15.0524		----			
2.6058	2714	0.020	----	15.1697		----			
2.6368	3779	0.020	----	15.2286		----			
2.7240	7435	0.013	0.977	15.3946	16:1 w7c alcohol	0.17	ECL deviates -0.002		
2.7482	47433	0.022	0.976	15.4408	15:0 DMA	1.06	ECL deviates -0.010		
2.8086	82700	0.016	0.974	15.5558	16:0 N alcohol	1.84	ECL deviates -0.001		
2.8411	115789	0.015	0.973	15.6176	16:0 iso	2.58	ECL deviates -0.002	Reference -0.003	
2.8915	12830	0.016	0.971	15.7137	16:0 anteiso	0.29	ECL deviates -0.001	Reference -0.002	
2.9195	60538	0.015	0.971	15.7669	16:1 w9c	1.35	ECL deviates -0.008		
2.9485	439512	0.016	0.970	15.8222	16:1 w7c	9.76	Column Overload		
2.9955	127113	0.015	0.969	15.9118	16:1 w5c	2.82	ECL deviates  0.001		
3.0447	499152	0.014	0.968	16.0048	16:0	11.06	Column Overload		
3.0711	26270	0.019	----	16.0490		----			
3.1241	3856	0.015	0.966	16.1378	16:2 DMA	0.09	ECL deviates  0.000		
3.1606	7484	0.022	----	16.1988		----			
3.1971	4850	0.020	----	16.2600		----			
3.2316	2326	0.019	0.964	16.3177	16:1 w7c DMA	0.05	ECL deviates  0.008		
3.2935	270557	0.020	0.963	16.4213	16:0 10-methyl	5.97	ECL deviates  0.001		
3.3297	52314	0.018	----	16.4819		----			
3.3568	32658	0.017	----	16.5273		----			
3.4123	63267	0.016	0.962	16.6202	17:0 iso	1.39	ECL deviates -0.004	Reference -0.004	
3.4700	78093	0.017	0.961	16.7168	17:0 anteiso	1.72	ECL deviates -0.003		
3.5142	50883	0.018	0.961	16.7907	17:1 w8c	1.12	ECL deviates -0.006		
3.5740	157573	0.019	0.960	16.8909	17:0 cyclo w7c	3.46	ECL deviates -0.003		
3.6389	23251	0.018	0.960	16.9996	17:0	0.51	ECL deviates  0.000	Reference -0.001	
3.6642	30734	0.017	0.959	17.0383	17:1 w7c 10-methyl	0.68	ECL deviates -0.005		
3.7076	8940	0.018	----	17.1046		----			
3.7428	1852	0.017	----	17.1584		----			
3.7923	4840	0.020	0.959	17.2340	16:0 2OH	0.11	ECL deviates -0.006		
3.8461	559	0.014	----	17.3163		----			
3.9028	33482	0.018	0.959	17.4029	17:0 10-methyl	0.74	ECL deviates -0.004		
3.9392	2876	0.012	0.959	17.4586	17:0 DMA	0.06	ECL deviates  0.000		
3.9608	10671	0.023	----	17.4915		----			
4.0167	9297	0.015	0.959	17.5769	18:3 w6c	0.20	ECL deviates -0.003		
4.0355	26773	0.027	----	17.6056		----			
4.1096	128232	0.018	0.959	17.7189	18:2 w6c	2.82	ECL deviates -0.008		
4.1451	320100	0.018	0.959	17.7731	18:1 w9c	7.03	ECL deviates -0.001		
4.1811	450400	0.018	0.959	17.8281	18:1 w7c	9.89	Column Overload		
4.2359	61598	0.023	----	17.9119		----			
4.2929	85084	0.019	0.959	17.9990	18:0	1.87	ECL deviates -0.001	Reference -0.001	
4.3489	30945	0.019	0.959	18.0801	18:1 w7c 10-methyl	0.68	ECL deviates -0.005		
4.4020	9773	0.029	0.959	18.1568	18:2 DMA	0.21	ECL deviates -0.003		
4.4507	4868	0.023	0.960	18.2273	18:1 w9c DMA	0.11	ECL deviates -0.010		
4.4824	2803	0.018	0.960	18.2731	18:1 w7c DMA	0.06	ECL deviates -0.009		
4.5101	1998	0.016	----	18.3132		----			
4.5627	136668	0.020	0.960	18.3892	18:0 10-methyl	3.01	ECL deviates -0.006		
4.6304	3551	0.020	0.960	18.4872	19:4 w6c	0.08	ECL deviates  0.002		
4.6754	8232	0.025	0.961	18.5523	19:3 w6c	0.18	ECL deviates -0.008		
4.7468	6255	0.027	0.961	18.6555	19:3 w3c	0.14	ECL deviates -0.003		
4.8031	20815	0.025	0.962	18.7370	19:0 anteiso	0.46	ECL deviates  0.010	Reference  0.009	
4.8527	15348	0.020	0.962	18.8087	19:1 w8c	0.34	ECL deviates -0.002		
4.8925	24271	0.016	0.962	18.8663	19:0 cyclo w9c	0.53	ECL deviates -0.006		
4.9170	127535	0.019	0.962	18.9018	19:0 cyclo w7c	2.81	ECL deviates -0.008		
4.9867	77696	0.019	----	19.0025	19:0	----	ECL deviates  0.002		
5.0473	2935	0.017	----	19.0870		----			
5.1396	2294	0.021	----	19.2157		----			
5.1749	12242	0.018	----	19.2649		----			
5.2598	32997	0.032	----	19.3833		----			
5.3151	10998	0.020	0.966	19.4604	20:5 w3c	0.24	ECL deviates -0.022		
5.3505	4425	0.018	----	19.5099		----			
5.3810	6862	0.018	----	19.5524		----			
5.4132	14769	0.026	----	19.5973		----			
5.5318	29465	0.025	0.967	19.7627	20:1 w9c	0.65	ECL deviates -0.010		
5.5606	13193	0.023	0.967	19.8029	20:1 w8c	0.29	ECL deviates -0.010		
5.6163	930	0.014	----	19.8805		----			
5.6496	879	0.016	0.968	19.9270	20:1 w4c	0.02	ECL deviates -0.004		
5.7018	27621	0.021	0.969	19.9998	20:0	0.61	ECL deviates  0.000	Reference -0.001	
5.7539	1556	0.022	----	20.0718		----			
5.8024	3133	0.017	----	20.1388		----			
5.8340	10289	0.020	----	20.1825		----			
5.9459	11737	0.028	----	20.3373		----			
5.9766	35610	0.024	----	20.3797		----			
6.0535	2652	0.021	----	20.4859		----			
6.0785	891	0.009	----	20.5205		----			
6.1017	3495	0.020	----	20.5527		----			
6.1504	6539	0.022	----	20.6199		----			
6.1752	3372	0.017	0.972	20.6542	21:3 w3c	0.08	ECL deviates  0.001		
6.2120	5602	0.030	----	20.7051		----			
6.2769	15568	0.020	0.972	20.7949	21:1 w8c	0.35	ECL deviates -0.003		
6.3346	12426	0.025	----	20.8745		----			
6.3923	26234	0.019	0.973	20.9543	21:1 w3c	0.58	ECL deviates  0.000		
6.4286	9247	0.023	0.973	21.0046	21:0	0.21	ECL deviates  0.005	Reference  0.003	
6.5079	6071	0.024	----	21.1137		----			
6.5926	7223	0.031	0.974	21.2304	22:5 w6c	0.16	ECL deviates -0.021		
6.6278	10967	0.023	----	21.2788		----			
6.6917	1601	0.024	----	21.3669		----			
6.7508	3125	0.041	0.974	21.4482	22:5 w3c	----	> max ar/ht		
6.8763	14898	0.030	0.974	21.6212	22:0 iso	0.33	ECL deviates  0.003		
6.9537	4247	0.026	0.974	21.7277	22:2 w6c	0.09	ECL deviates -0.011		
6.9876	4515	0.023	0.974	21.7744	22:1 w9c	0.10	ECL deviates  0.002		
7.0212	5907	0.029	0.974	21.8207	22:1 w8c	0.13	ECL deviates  0.007		
7.1056	8545	0.021	0.974	21.9369	22:1 w3c	0.19	ECL deviates -0.010		
7.1493	32422	0.021	0.974	21.9972	22:0	0.72	ECL deviates -0.003	Reference -0.006	
7.2136	2717	0.022	----	22.0872		----			
7.2373	2601	0.027	----	22.1204		----			
7.3247	12199	0.020	----	22.2428		----			
7.3753	2441	0.032	----	22.3136		----			
7.4420	2495	0.027	----	22.4071		----			
7.4937	1539	0.023	0.972	22.4795	23:4 w6c	0.03	ECL deviates  0.008		
7.5384	1822	0.026	----	22.5421		----			
7.6055	5344	0.045	0.971	22.6360	23:3 w3c	----	> max ar/ht		
7.7048	6282	0.025	----	22.7751		----			
7.7646	2437	0.021	----	22.8589		----			
7.8064	12076	0.020	0.969	22.9175	23:1 w4c	0.27	ECL deviates -0.009		
7.8644	7397	0.018	0.968	22.9987	23:0	0.16	ECL deviates -0.001	Reference -0.005	
7.9112	2915	0.025	----	23.0650		----			
8.0714	8865	0.022	----	23.2920		----			
8.2840	1267	0.019	0.961	23.5934	24:3 w6c	0.03	ECL deviates  0.003		
8.3224	7399	0.023	0.960	23.6478	24:3 w3c	0.16	ECL deviates -0.007		
8.3793	2381	0.021	----	23.7285		----			
8.4115	3237	0.022	----	23.7742		----			
8.4888	2896	0.038	----	23.8838		----	> max ar/ht		
8.5700	26806	0.020	0.954	23.9989	24:0	0.59	ECL deviates -0.001	Reference -0.006	
8.6719	1504	0.019	----	24.1433		----	> max rt		
8.9271	4085	0.020	----	24.5052		----	> max rt		
9.2261	18929	0.020	----	24.9292		----	> max rt		
9.2576	2969	0.014	----	24.9738		----	> max rt		
9.4664	11352	0.021	----	25.2699		----	> max rt		

ECL Deviation: 0.007                            Reference ECL Shift: 0.005       Number Reference Peaks: 21
Total Response: 5030299                       Total Named: 4500048
Percent Named: 89.46%                         Total Amount: 4374960
Profile Comment:   Column Overload:  A peak's response is greater than 400000.0.  Dilute and re-run.

(No search libraries specified in method PLFAD1.)
