# Supplementary material for: Microbial regulation of soil carbon properties under nitrogen addition and plant inputs removal
Source: PeerJ. 2019 Jul 17;7:e7343. doi: 10.7717/peerj.7343 (PMC6642627; doi:10.7717/peerj.7343)
Supplement: File S1 — The raw data showed the soil microbial PLFAs files in the year of 2015 and 2016. Each file of rtf. represented the microbial PLFAs for each soil sample. In the Supplemental File, the Excel file named “Numbers” showed the plots names and the related rtf. file names. [file peerj-07-7343-s002.zip › supplementary files/2015/39.rtf]

Volume: DATA            File: E164213.59A        Samp Ctr: 8                  ID Number: 29336 
Type: Samp                   Bottle: 7                        Method: PLFAD1 
Created: 4/21/2016 11:57:09 AM 
Sample ID: 39 


RT	Response	Ar/Ht	RFact	ECL	Peak Name	Percent	Comment1	Comment2	
0.7144	1.894E+9	0.014	----	7.6518	SOLVENT PEAK	----	< min rt		
0.8863	833	0.009	----	8.7835		----	< min rt		
0.9451	1516	0.013	----	9.1662		----	< min rt		
0.9719	920	0.013	----	9.3403		----	< min rt		
1.1873	5153	0.012	----	10.7422		----			
1.2260	715	0.016	1.212	10.9947	11:0	0.02	ECL deviates -0.005	Reference -0.016	
1.2633	1758	0.015	1.195	11.1761	10:0 2OH	0.04	ECL deviates -0.008		
1.3201	1150	0.014	1.172	11.4468	10:0 3OH	0.02	ECL deviates  0.005		
1.3543	1980	0.021	1.157	11.6096	12:0 iso	0.04	ECL deviates -0.002	Reference -0.012	
1.3907	1818	0.015	----	11.7830		----			
1.4375	5643	0.015	1.127	12.0061	12:0	0.11	ECL deviates  0.006	Reference -0.002	
1.4953	3825	0.016	----	12.2136		----			
1.5216	523	0.011	----	12.3080		----			
1.5602	2163	0.018	----	12.4464		----			
1.6059	5951	0.013	1.085	12.6100	13:0 iso	0.12	ECL deviates -0.002	Reference -0.009	
1.6366	4535	0.019	1.079	12.7201	13:0 anteiso	0.09	ECL deviates  0.011	Reference  0.004	
1.6914	1205	0.015	1.067	12.9167	13:1 w5c	0.02	ECL deviates -0.003		
1.7155	2907	0.014	1.062	13.0033	13:0	0.06	ECL deviates  0.003	Reference -0.003	
1.7840	918	0.016	----	13.1946	12:0 2OH	----	ECL deviates  0.008		
1.8257	487	0.010	----	13.3109		----			
1.8766	2988	0.018	1.039	13.4526	13:0 DMA	0.06	ECL deviates -0.008		
1.9334	62442	0.014	1.032	13.6110	14:0 iso	1.16	ECL deviates -0.003	Reference -0.008	
1.9689	1461	0.012	1.028	13.7098	14:0 anteiso	0.03	ECL deviates -0.006	Reference -0.011	
1.9937	1772	0.011	1.025	13.7789	14:1 w9c	0.03	ECL deviates  0.001		
2.0080	2546	0.014	----	13.8187		----			
2.0408	4408	0.013	1.020	13.9102	14:1 w5c	0.08	ECL deviates -0.001		
2.0732	71693	0.014	1.016	14.0006	14:0	1.31	ECL deviates  0.001	Reference -0.004	
2.0998	705	0.009	----	14.0613		----			
2.1286	2219	0.014	----	14.1263	14:0 iso 3OH	----	ECL deviates  0.002		
2.1539	3962	0.024	----	14.1833		----			
2.2104	2730	0.021	----	14.3108		----			
2.2663	83637	0.017	1.001	14.4370	15:1 iso w6c	1.50	ECL deviates -0.002		
2.2843	11475	0.010	0.999	14.4776	15:4 w3c	0.21	ECL deviates -0.013		
2.3072	20790	0.014	0.998	14.5292	15:1 anteiso w9c	0.37	ECL deviates -0.001		
2.3459	334919	0.014	0.996	14.6166	15:0 iso	5.99	ECL deviates  0.000	Reference -0.004	
2.3872	238805	0.014	0.993	14.7099	15:0 anteiso	4.26	ECL deviates -0.001	Reference -0.005	
2.4519	11627	0.021	0.989	14.8557	15:1 w6c	0.21	ECL deviates -0.004		
2.4722	2191	0.012	0.988	14.9015	15:1 w5c	0.04	ECL deviates -0.011		
2.5152	38828	0.014	0.985	14.9987	15:0	0.69	ECL deviates -0.001	Reference -0.004	
2.5434	11793	0.017	----	15.0531		----			
2.6062	2999	0.024	----	15.1724		----			
2.6352	5393	0.021	----	15.2276		----			
2.7471	66890	0.023	0.976	15.4405	15:0 DMA	1.17	ECL deviates -0.010		
2.8081	92297	0.016	0.974	15.5567	16:0 N alcohol	1.62	ECL deviates  0.000		
2.8409	140388	0.016	0.973	15.6192	16:0 iso	2.45	ECL deviates -0.001	Reference -0.003	
2.8933	13238	0.013	0.971	15.7188	16:0 anteiso	0.23	ECL deviates  0.004	Reference  0.002	
2.9184	72713	0.016	0.971	15.7665	16:1 w9c	1.27	ECL deviates -0.008		
2.9492	573263	0.018	0.970	15.8252	16:1 w7c	9.99	Column Overload		
2.9952	182968	0.016	0.969	15.9127	16:1 w5c	3.18	ECL deviates  0.002		
3.0465	639068	0.015	0.968	16.0093	16:0	11.11	Column Overload		
3.0716	21890	0.020	----	16.0514		----			
3.1237	5835	0.017	0.966	16.1386	16:2 DMA	0.10	ECL deviates  0.001		
3.1597	9926	0.025	----	16.1987		----			
3.1957	4349	0.016	----	16.2590		----			
3.2315	3335	0.019	0.964	16.3188	16:1 w7c DMA	0.06	ECL deviates  0.009		
3.2933	304125	0.019	0.963	16.4222	16:0 10-methyl	5.26	ECL deviates  0.002		
3.3299	58849	0.018	0.963	16.4835	17:1 iso w9c	1.02	ECL deviates -0.014		
3.3569	40212	0.017	0.962	16.5286	17:1 anteiso w9c	0.70	ECL deviates -0.007		
3.4126	76844	0.016	0.962	16.6218	17:0 iso	1.33	ECL deviates -0.002	Reference -0.003	
3.4706	94420	0.017	0.961	16.7190	17:0 anteiso	1.63	ECL deviates -0.001		
3.5148	68255	0.018	0.961	16.7929	17:1 w8c	1.18	ECL deviates -0.004		
3.5743	199554	0.018	0.960	16.8926	17:0 cyclo w7c	3.44	ECL deviates -0.001		
3.6388	29149	0.018	0.960	17.0004	17:0	0.50	ECL deviates  0.000	Reference -0.001	
3.6649	26773	0.017	0.959	17.0404	17:1 w7c 10-methyl	0.46	ECL deviates -0.003		
3.7062	7832	0.017	----	17.1035		----			
3.7404	2191	0.021	----	17.1557		----			
3.7910	6057	0.019	0.959	17.2329	16:0 2OH	0.10	ECL deviates -0.007		
3.8437	581	0.013	----	17.3135		----			
3.9023	38809	0.017	0.959	17.4030	17:0 10-methyl	0.67	ECL deviates -0.004		
3.9389	3067	0.013	0.959	17.4590	17:0 DMA	0.05	ECL deviates  0.001		
3.9600	15422	0.022	----	17.4911		----			
4.0350	48947	0.029	----	17.6057		----			
4.1096	152171	0.018	0.959	17.7197	18:2 w6c	2.62	ECL deviates -0.007		
4.1451	427675	0.020	0.959	17.7739	18:1 w9c	7.37	Column Overload		
4.1834	614354	0.017	0.959	17.8324	18:1 w7c	10.58	Column Overload		
4.2343	81388	0.022	----	17.9101		----			
4.2933	102341	0.019	0.959	18.0003	18:0	1.76	ECL deviates  0.000	Reference -0.001	
4.3481	45351	0.019	0.959	18.0795	18:1 w7c 10-methyl	0.78	ECL deviates -0.006		
4.3992	11549	0.025	0.959	18.1534	18:2 DMA	0.20	ECL deviates -0.007		
4.4498	6016	0.024	0.960	18.2266	18:1 w9c DMA	0.10	ECL deviates -0.010		
4.4819	5439	0.029	0.960	18.2729	18:1 w7c DMA	0.09	ECL deviates -0.010		
4.5614	145500	0.021	0.960	18.3879	18:0 10-methyl	2.51	ECL deviates -0.007		
4.6263	4884	0.022	0.960	18.4818	19:4 w6c	0.08	ECL deviates -0.003		
4.6742	11264	0.026	0.961	18.5510	19:3 w6c	0.19	ECL deviates -0.009		
4.7461	8471	0.025	0.961	18.6549	19:3 w3c	0.15	ECL deviates -0.003		
4.8110	17465	0.023	----	18.7488		----			
4.8504	20192	0.019	0.962	18.8058	19:1 w8c	0.35	ECL deviates -0.005		
4.8832	35123	0.021	0.962	18.8532	19:1 w6c	0.61	ECL deviates  0.001		
4.9156	151995	0.019	0.962	18.9002	19:0 cyclo w7c	2.63	ECL deviates -0.010		
4.9845	84554	0.019	----	18.9997	19:0	----	ECL deviates  0.000		
5.0456	4606	0.019	----	19.0849		----			
5.1358	3111	0.019	----	19.2107		----			
5.1735	29012	0.019	----	19.2633		----			
5.2605	48548	0.030	----	19.3847		----			
5.3130	13804	0.020	0.966	19.4579	20:5 w3c	0.24	ECL deviates -0.024		
5.3485	4486	0.018	----	19.5074		----			
5.3799	10559	0.020	----	19.5511		----			
5.4121	16252	0.027	----	19.5960		----			
5.5317	37642	0.027	0.967	19.7629	20:1 w9c	0.65	ECL deviates -0.010		
5.5602	17221	0.025	0.967	19.8026	20:1 w8c	0.30	ECL deviates -0.010		
5.7000	36327	0.023	0.969	19.9977	20:0	0.63	ECL deviates -0.002	Reference -0.004	
5.7571	2340	0.021	----	20.0766		----			
5.8032	3504	0.018	----	20.1403		----			
5.8325	12879	0.020	----	20.1808		----			
5.9205	8017	0.020	----	20.3024		----			
5.9420	8996	0.017	----	20.3322		----			
5.9749	42415	0.023	----	20.3776		----			
6.0491	1607	0.016	----	20.4802		----			
6.1019	5851	0.030	----	20.5532		----			
6.1484	6893	0.021	----	20.6175		----			
6.1740	4848	0.019	0.972	20.6528	21:3 w3c	0.08	ECL deviates -0.001		
6.2180	8185	0.031	----	20.7138		----			
6.2765	18272	0.022	0.972	20.7947	21:1 w8c	0.32	ECL deviates -0.003		
6.3334	18000	0.023	----	20.8732		----			
6.3923	25687	0.019	0.973	20.9546	21:1 w3c	0.45	ECL deviates  0.001		
6.4271	11320	0.023	0.973	21.0028	21:0	0.20	ECL deviates  0.003	Reference  0.001	
6.5072	5861	0.022	----	21.1131		----			
6.5495	2125	0.018	----	21.1715		----			
6.5929	7025	0.020	0.974	21.2312	22:5 w6c	0.12	ECL deviates -0.021		
6.6249	22101	0.021	----	21.2752		----			
6.6925	2012	0.021	----	21.3684		----			
6.7548	4143	0.039	0.974	21.4542	22:5 w3c	----	> max ar/ht		
6.8775	20321	0.029	0.974	21.6233	22:0 iso	0.36	ECL deviates  0.005		
6.9510	5172	0.027	0.974	21.7245	22:2 w6c	0.09	ECL deviates -0.014		
6.9887	5760	0.022	0.974	21.7764	22:1 w9c	0.10	ECL deviates  0.004		
7.0211	7237	0.028	0.974	21.8211	22:1 w8c	0.13	ECL deviates  0.008		
7.1063	9071	0.020	0.974	21.9385	22:1 w3c	0.16	ECL deviates -0.009		
7.1501	53569	0.019	0.974	21.9988	22:0	0.94	ECL deviates -0.001	Reference -0.004	
7.2116	2751	0.025	----	22.0849		----			
7.2435	2820	0.027	----	22.1296		----			
7.3247	13102	0.020	----	22.2432		----			
7.3796	2641	0.028	----	22.3202		----			
7.4412	2342	0.025	----	22.4065		----			
7.4935	1633	0.021	0.972	22.4798	23:4 w6c	0.03	ECL deviates  0.009		
7.5349	1894	0.021	----	22.5378		----			
7.6037	6658	0.045	----	22.6342		----	> max ar/ht		
7.7029	8048	0.023	----	22.7732		----			
7.7665	3322	0.021	----	22.8622		----			
7.8064	12300	0.023	0.969	22.9182	23:1 w4c	0.21	ECL deviates -0.008		
7.8646	9618	0.018	0.968	22.9998	23:0	0.17	ECL deviates  0.000	Reference -0.005	
7.9118	2593	0.024	----	23.0666		----			
8.0315	1000	0.016	----	23.2364		----			
8.0722	11451	0.020	----	23.2941		----			
8.1136	635	0.014	----	23.3529		----			
8.2821	1523	0.017	0.961	23.5917	24:3 w6c	0.03	ECL deviates  0.001		
8.3217	9425	0.024	0.960	23.6479	24:3 w3c	0.16	ECL deviates -0.007		
8.3752	2943	0.024	----	23.7238		----			
8.4136	3498	0.025	0.958	23.7783	24:1 w9c	0.06	ECL deviates -0.008		
8.4852	1987	0.022	----	23.8798		----			
8.5248	807	0.017	0.955	23.9360	24:1 w3c	0.01	ECL deviates -0.013		
8.5704	33080	0.019	0.954	24.0006	24:0	0.57	ECL deviates  0.001	Reference -0.006	
8.6741	1825	0.017	----	24.1479		----	> max rt		
8.8016	888	0.020	----	24.3287		----	> max rt		
8.9237	11593	0.019	----	24.5018		----	> max rt		
9.0269	1315	0.028	----	24.6482		----	> max rt		
9.1535	2195	0.023	----	24.8279		----	> max rt		
9.2266	21587	0.024	----	24.9317		----	> max rt		
9.4643	14473	0.021	----	25.2690		----	> max rt		

ECL Deviation: 0.007                            Reference ECL Shift: 0.006       Number Reference Peaks: 22
Total Response: 6326251                       Total Named: 5736473
Percent Named: 90.68%                         Total Amount: 5570415
Profile Comment:   Column Overload:  A peak's response is greater than 400000.0.  Dilute and re-run.

(No search libraries specified in method PLFAD1.)
