# Supplementary material for: Microbial regulation of soil carbon properties under nitrogen addition and plant inputs removal
Source: PeerJ. 2019 Jul 17;7:e7343. doi: 10.7717/peerj.7343 (PMC6642627; doi:10.7717/peerj.7343)
Supplement: File S1 — The raw data showed the soil microbial PLFAs files in the year of 2015 and 2016. Each file of rtf. represented the microbial PLFAs for each soil sample. In the Supplemental File, the Excel file named “Numbers” showed the plots names and the related rtf. file names. [file peerj-07-7343-s002.zip › supplementary files/2015/4.rtf]

Volume: DATA            File: E164203.63A        Samp Ctr: 3                  ID Number: 29298 
Type: Samp                   Bottle: 2                        Method: PLFAD1 
Created: 4/20/2016 9:40:35 AM 
Sample ID: wuran4 


RT	Response	Ar/Ht	RFact	ECL	Peak Name	Percent	Comment1	Comment2	
0.7148	1.889E+9	0.014	----	7.6538	SOLVENT PEAK	----	< min rt		
0.7887	5578	0.019	----	8.1348		----	< min rt		
0.8869	1650	0.010	----	8.7781		----	< min rt		
1.1880	1496	0.012	----	10.7378		----			
1.2658	571	0.012	1.208	11.1787	10:0 2OH	0.03	ECL deviates -0.005		
1.3549	770	0.018	1.170	11.6031	12:0 iso	0.04	ECL deviates -0.009	Reference -0.010	
1.3672	357	0.009	----	11.6615		----			
1.3923	1035	0.014	----	11.7810		----			
1.4390	3116	0.014	1.138	12.0027	12:0	0.15	ECL deviates  0.003	Reference  0.002	
1.4969	1893	0.016	----	12.2102		----			
1.5617	1212	0.017	----	12.4423		----			
1.6078	2621	0.012	1.094	12.6074	13:0 iso	0.12	ECL deviates -0.005	Reference -0.006	
1.6396	2059	0.016	1.088	12.7213	13:0 anteiso	0.09	ECL deviates  0.012	Reference  0.011	
1.6925	1008	0.019	1.075	12.9109	13:1 w5c	0.05	ECL deviates -0.009		
1.7180	1152	0.013	1.071	13.0019	13:0	0.05	ECL deviates  0.002	Reference  0.001	
1.8752	1379	0.018	----	13.4396		----			
1.9071	374	0.008	----	13.5286		----			
1.9364	27472	0.013	1.038	13.6102	14:0 iso	1.20	ECL deviates -0.004	Reference -0.004	
1.9554	1872	0.011	----	13.6631		----			
1.9780	917	0.013	1.033	13.7259	14:0 anteiso	0.04	ECL deviates  0.010	Reference  0.010	
1.9978	812	0.010	1.031	13.7811	14:1 w9c	0.04	ECL deviates  0.004		
2.0115	1313	0.012	----	13.8193		----			
2.0761	28700	0.014	1.021	13.9992	14:0	1.23	ECL deviates -0.001	Reference -0.001	
2.1059	790	0.013	----	14.0665		----			
2.1327	1011	0.014	----	14.1269	14:0 iso 3OH	----	ECL deviates  0.002		
2.1576	3185	0.026	----	14.1830		----			
2.2241	2229	0.021	----	14.3326		----			
2.2698	28588	0.017	1.005	14.4354	15:1 iso w6c	1.21	ECL deviates -0.004		
2.2894	6371	0.013	1.003	14.4796	15:4 w3c	0.27	ECL deviates -0.011		
2.3103	8005	0.014	1.001	14.5267	15:1 anteiso w9c	0.34	ECL deviates -0.003		
2.3491	144316	0.013	0.999	14.6140	15:0 iso	6.05	ECL deviates -0.003	Reference -0.003	
2.3906	109681	0.014	0.996	14.7076	15:0 anteiso	4.58	ECL deviates -0.003	Reference -0.004	
2.4566	5600	0.025	0.991	14.8562	15:1 w6c	0.23	ECL deviates -0.004		
2.5201	15900	0.015	0.987	14.9991	15:0	0.66	ECL deviates -0.001	Reference -0.001	
2.5487	7774	0.016	----	15.0536		----			
2.6110	1900	0.019	----	15.1719		----			
2.6413	1993	0.020	----	15.2294		----			
2.7257	5900	0.015	0.978	15.3896	16:1 w7c alcohol	0.24	ECL deviates -0.007		
2.7532	24365	0.021	0.976	15.4418	15:0 DMA	1.00	ECL deviates -0.009		
2.8138	75286	0.015	0.974	15.5569	16:0 N alcohol	3.08	ECL deviates  0.000		
2.8461	59615	0.015	0.973	15.6182	16:0 iso	2.43	ECL deviates -0.002	Reference -0.002	
2.8979	6107	0.014	0.971	15.7164	16:0 anteiso	0.25	ECL deviates  0.001	Reference  0.001	
2.9236	34488	0.017	0.970	15.7652	16:1 w9c	1.40	ECL deviates -0.010		
2.9530	234348	0.017	0.969	15.8209	16:1 w7c	9.53	ECL deviates -0.003		
3.0005	70980	0.016	0.968	15.9112	16:1 w5c	2.88	ECL deviates  0.000		
3.0493	249648	0.015	0.966	16.0035	16:0	10.13	ECL deviates  0.003	Reference  0.003	
3.0774	15695	0.019	----	16.0504		----			
3.1313	1723	0.016	0.964	16.1405	16:2 DMA	0.07	ECL deviates  0.003		
3.1657	3742	0.022	----	16.1979		----			
3.2026	2529	0.019	----	16.2597		----			
3.2370	1518	0.021	0.962	16.3171	16:1 w7c DMA	0.06	ECL deviates  0.007		
3.3000	150047	0.019	0.961	16.4225	16:0 10-methyl	6.05	ECL deviates  0.002		
3.3349	41169	0.018	----	16.4808		----			
3.3641	18122	0.019	0.960	16.5295	17:1 anteiso w9c	0.73	ECL deviates -0.006		
3.4193	36857	0.016	0.959	16.6218	17:0 iso	1.48	ECL deviates -0.002	Reference -0.002	
3.4767	41386	0.017	0.958	16.7179	17:0 anteiso	1.66	ECL deviates -0.002		
3.5207	22400	0.019	0.957	16.7914	17:1 w8c	0.90	ECL deviates -0.006		
3.5806	80756	0.018	0.957	16.8914	17:0 cyclo w7c	3.24	ECL deviates -0.002		
3.6445	11130	0.017	0.956	16.9983	17:0	0.45	ECL deviates -0.002	Reference -0.002	
3.6715	19967	0.017	0.956	17.0397	17:1 w7c 10-methyl	0.80	ECL deviates -0.004		
3.7142	4218	0.017	----	17.1046		----			
3.7499	1321	0.020	----	17.1591		----			
3.7990	1777	0.020	0.955	17.2339	16:0 2OH	0.07	ECL deviates -0.006		
3.9091	15223	0.018	0.954	17.4016	17:0 10-methyl	0.61	ECL deviates -0.005		
3.9463	2135	0.013	0.954	17.4584	17:0 DMA	0.09	ECL deviates  0.000		
3.9676	4248	0.021	----	17.4908		----			
4.0430	19144	0.031	----	17.6057		----			
4.1165	47038	0.017	0.953	17.7177	18:2 w6c	1.88	ECL deviates -0.009		
4.1497	165940	0.019	0.953	17.7683	18:1 w9c	6.64	ECL deviates -0.006		
4.1856	250085	0.018	0.953	17.8230	18:1 w7c	10.01	ECL deviates -0.004		
4.2450	26825	0.022	0.953	17.9135	18:1 w5c	1.07	ECL deviates -0.009		
4.3005	44709	0.018	0.953	17.9982	18:0	1.79	ECL deviates -0.002	Reference -0.003	
4.3554	14219	0.019	0.953	18.0776	18:1 w7c 10-methyl	0.57	ECL deviates -0.007		
4.4089	5792	0.028	0.953	18.1549	18:2 DMA	0.23	ECL deviates -0.005		
4.4601	3865	0.026	0.953	18.2289	18:1 w9c DMA	0.15	ECL deviates -0.008		
4.5198	1226	0.016	----	18.3152		----			
4.5698	70532	0.020	0.954	18.3874	18:0 10-methyl	2.82	ECL deviates -0.008		
4.6387	1923	0.019	0.954	18.4870	19:4 w6c	0.08	ECL deviates  0.002		
4.6873	6476	0.025	0.954	18.5573	19:3 w6c	0.26	ECL deviates -0.003		
4.7379	1716	0.015	0.954	18.6303	19:0 iso	0.07	ECL deviates  0.000		
4.7530	1309	0.013	0.955	18.6522	19:3 w3c	0.05	ECL deviates -0.006		
4.8173	9996	0.020	----	18.7451		----			
4.8630	9229	0.017	0.955	18.8111	19:1 w8c	0.37	ECL deviates  0.000		
4.9000	12092	0.015	0.955	18.8646	19:0 cyclo w9c	0.48	ECL deviates -0.007		
4.9258	67548	0.018	0.955	18.9019	19:0 cyclo w7c	2.71	ECL deviates -0.008		
4.9947	74334	0.019	----	19.0015	19:0	----	ECL deviates  0.002		
5.0576	1164	0.017	----	19.0893		----			
5.1510	2797	0.021	----	19.2196		----			
5.1829	6533	0.018	----	19.2641		----			
5.2698	12476	0.025	0.958	19.3854	20:4 w6c	0.50	ECL deviates -0.018		
5.3234	5227	0.020	0.958	19.4603	20:5 w3c	0.21	ECL deviates -0.022		
5.3589	1945	0.017	----	19.5098		----			
5.3885	3572	0.018	----	19.5511		----			
5.4232	7760	0.024	----	19.5995		----			
5.5405	16187	0.025	0.960	19.7632	20:1 w9c	0.65	ECL deviates -0.009		
5.5704	8720	0.024	0.960	19.8049	20:1 w8c	0.35	ECL deviates -0.008		
5.7099	16156	0.022	0.961	19.9996	20:0	0.65	ECL deviates  0.000	Reference -0.002	
5.8111	2311	0.023	----	20.1390		----			
5.8426	3782	0.020	----	20.1823		----			
5.9601	5018	0.020	----	20.3439		----			
5.9880	24231	0.022	0.963	20.3823	20:0 10-methyl	0.98	ECL deviates -0.015		
6.1106	2544	0.028	----	20.5510		----			
6.1603	7754	0.026	----	20.6195		----			
6.2206	2863	0.027	----	20.7024		----			
6.2868	9071	0.018	0.965	20.7935	21:1 w8c	0.37	ECL deviates -0.005		
6.3478	5426	0.024	----	20.8774		----			
6.4037	18101	0.020	0.966	20.9543	21:1 w3c	0.73	ECL deviates  0.000		
6.4389	5890	0.022	0.966	21.0027	21:0	0.24	ECL deviates  0.003	Reference  0.000	
6.5188	3400	0.021	----	21.1132		----			
6.6027	3165	0.030	----	21.2291		----			
6.6377	5001	0.020	----	21.2775		----			
6.8870	7839	0.027	0.968	21.6220	22:0 iso	0.32	ECL deviates  0.004		
6.9609	1823	0.024	0.968	21.7242	22:2 w6c	0.07	ECL deviates -0.014		
6.9945	1651	0.020	0.968	21.7706	22:1 w9c	0.07	ECL deviates -0.002		
7.0349	3188	0.024	----	21.8264		----			
7.1153	4441	0.018	0.969	21.9375	22:1 w3c	0.18	ECL deviates -0.010		
7.1589	15508	0.018	0.969	21.9978	22:0	0.63	ECL deviates -0.002	Reference -0.005	
7.2207	1232	0.025	----	22.0843		----			
7.3346	9674	0.019	----	22.2438		----			
7.7153	1888	0.018	----	22.7772		----			
7.7663	1304	0.022	----	22.8487		----			
7.8186	8802	0.023	0.967	22.9219	23:1 w4c	0.36	ECL deviates -0.005		
7.8761	3503	0.019	0.966	23.0025	23:0	0.14	ECL deviates  0.002	Reference -0.002	
7.9190	1275	0.021	----	23.0634		----			
8.0823	4924	0.019	----	23.2953		----			
8.3305	5653	0.022	0.960	23.6480	24:3 w3c	0.23	ECL deviates -0.007		
8.4231	4201	0.038	0.959	23.7794	24:1 w9c	----	> max ar/ht		
8.4985	2259	0.032	----	23.8866		----			
8.5776	14977	0.018	0.956	23.9990	24:0	0.60	ECL deviates -0.001	Reference -0.006	
8.6869	942	0.022	----	24.1542		----	> max rt		
8.9374	6166	0.020	----	24.5101		----	> max rt		
9.0442	1077	0.020	----	24.6617		----	> max rt		
9.2381	18359	0.021	----	24.9373		----	> max rt		
9.4755	9128	0.020	----	25.2746		----	> max rt		

ECL Deviation: 0.007                            Reference ECL Shift: 0.005       Number Reference Peaks: 22
Total Response: 2688061                       Total Named: 2460992
Percent Named: 91.55%                         Total Amount: 2386725
Profile Comment:   Review report comments.

(No search libraries specified in method PLFAD1.)
