# Supplementary material for: Microbial regulation of soil carbon properties under nitrogen addition and plant inputs removal
Source: PeerJ. 2019 Jul 17;7:e7343. doi: 10.7717/peerj.7343 (PMC6642627; doi:10.7717/peerj.7343)
Supplement: File S1 — The raw data showed the soil microbial PLFAs files in the year of 2015 and 2016. Each file of rtf. represented the microbial PLFAs for each soil sample. In the Supplemental File, the Excel file named “Numbers” showed the plots names and the related rtf. file names. [file peerj-07-7343-s002.zip › supplementary files/2015/40.rtf]

Volume: DATA            File: E164213.59A        Samp Ctr: 9                  ID Number: 29337 
Type: Samp                   Bottle: 8                        Method: PLFAD1 
Created: 4/21/2016 12:25:37 PM 
Sample ID: 40 


RT	Response	Ar/Ht	RFact	ECL	Peak Name	Percent	Comment1	Comment2	
0.7145	1.917E+9	0.016	----	7.6532	SOLVENT PEAK	----	< min rt		
0.8861	582	0.009	----	8.7732		----	< min rt		
0.9459	353	0.008	----	9.1715		----	< min rt		
1.1872	2580	0.011	----	10.7425		----			
1.2627	1370	0.016	1.195	11.1743	10:0 2OH	0.04	ECL deviates -0.009		
1.3191	575	0.013	1.172	11.4431	10:0 3OH	0.02	ECL deviates  0.002		
1.3529	1001	0.016	1.157	11.6044	12:0 iso	0.03	ECL deviates -0.008		
1.3909	1226	0.014	----	11.7852		----			
1.4368	3386	0.013	1.127	12.0040	12:0	0.09	ECL deviates  0.004	Reference -0.006	
1.4949	1855	0.013	----	12.2137		----			
1.5592	1101	0.013	----	12.4444		----			
1.6053	3863	0.012	1.085	12.6097	13:0 iso	0.10	ECL deviates -0.003	Reference -0.011	
1.6339	2695	0.015	1.079	12.7121	13:0 anteiso	0.07	ECL deviates  0.003	Reference -0.006	
1.6896	619	0.012	1.067	12.9120	13:1 w5c	0.02	ECL deviates -0.008		
1.7149	1765	0.012	1.062	13.0028	13:0	0.04	ECL deviates  0.003	Reference -0.005	
1.7815	687	0.015	----	13.1894	12:0 2OH	----	ECL deviates  0.003		
1.8750	2263	0.018	1.039	13.4501	13:0 DMA	0.06	ECL deviates -0.010		
1.9327	45874	0.013	1.032	13.6108	14:0 iso	1.13	ECL deviates -0.003	Reference -0.010	
1.9688	1436	0.014	1.028	13.7114	14:0 anteiso	0.04	ECL deviates -0.005	Reference -0.011	
1.9929	1318	0.010	1.025	13.7786	14:1 w9c	0.03	ECL deviates  0.001		
2.0074	1841	0.013	----	13.8191		----			
2.0415	2723	0.011	1.020	13.9142	14:1 w5c	0.07	ECL deviates  0.003		
2.0720	55740	0.014	1.016	13.9989	14:0	1.35	ECL deviates -0.001	Reference -0.007	
2.0991	363	0.008	----	14.0615		----			
2.1275	1165	0.012	----	14.1258	14:0 iso 3OH	----	ECL deviates  0.001		
2.1523	2492	0.022	----	14.1818		----			
2.2201	2025	0.019	----	14.3347		----			
2.2660	78893	0.019	1.001	14.4382	15:1 iso w6c	1.88	ECL deviates -0.001		
2.3059	15476	0.014	0.998	14.5284	15:1 anteiso w9c	0.37	ECL deviates -0.002		
2.3450	281187	0.014	0.996	14.6165	15:0 iso	6.65	ECL deviates -0.001	Reference -0.006	
2.3866	209593	0.014	0.993	14.7105	15:0 anteiso	4.95	ECL deviates  0.000	Reference -0.006	
2.4507	7611	0.021	0.989	14.8552	15:1 w6c	0.18	ECL deviates -0.005		
2.4707	1062	0.010	0.988	14.9002	15:1 w5c	0.02	ECL deviates -0.012		
2.5148	29105	0.014	0.985	14.9999	15:0	0.68	ECL deviates  0.000	Reference -0.005	
2.5429	6359	0.016	----	15.0541		----			
2.6355	3131	0.016	----	15.2303		----			
2.7216	7170	0.012	0.977	15.3941	16:1 w7c alcohol	0.17	ECL deviates -0.002		
2.7465	45043	0.020	0.976	15.4416	15:0 DMA	1.04	ECL deviates -0.009		
2.8074	78235	0.016	0.974	15.5575	16:0 N alcohol	1.81	ECL deviates  0.001		
2.8400	114260	0.016	0.973	15.6194	16:0 iso	2.64	ECL deviates  0.000	Reference -0.005	
2.8914	11833	0.016	0.971	15.7174	16:0 anteiso	0.27	ECL deviates  0.002	Reference -0.002	
2.9191	57025	0.017	0.971	15.7701	16:1 w9c	1.32	ECL deviates -0.005		
2.9480	390047	0.018	0.970	15.8250	16:1 w7c	8.99	ECL deviates  0.001		
2.9946	132962	0.017	0.969	15.9138	16:1 w5c	3.06	ECL deviates  0.003		
3.0453	541580	0.016	0.968	16.0096	16:0	12.45	Column Overload		
3.0700	8043	0.012	----	16.0508		----			
3.0920	13329	0.016	----	16.0877		----			
3.1232	4791	0.016	0.966	16.1398	16:2 DMA	0.11	ECL deviates  0.002		
3.1626	9047	0.021	----	16.2058		----			
3.1951	4040	0.018	----	16.2601		----			
3.2307	2167	0.021	0.964	16.3198	16:1 w7c DMA	0.05	ECL deviates  0.010		
3.2922	216525	0.019	0.963	16.4226	16:0 10-methyl	4.96	ECL deviates  0.003		
3.3273	48613	0.019	----	16.4814		----			
3.3547	30676	0.018	----	16.5273		----			
3.4119	61558	0.017	0.962	16.6229	17:0 iso	1.41	ECL deviates -0.001	Reference -0.005	
3.4684	80905	0.017	0.961	16.7175	17:0 anteiso	1.85	ECL deviates -0.003		
3.5125	52507	0.018	0.961	16.7914	17:1 w8c	1.20	ECL deviates -0.006		
3.5726	137951	0.017	0.960	16.8919	17:0 cyclo w7c	3.15	ECL deviates -0.002		
3.6369	25815	0.019	0.960	16.9995	17:0	0.59	ECL deviates  0.000	Reference -0.004	
3.6634	15105	0.017	0.959	17.0404	17:1 w7c 10-methyl	0.34	ECL deviates -0.003		
3.7052	5646	0.018	----	17.1041		----			
3.7366	1980	0.018	----	17.1521		----			
3.7900	5604	0.019	0.959	17.2337	16:0 2OH	0.13	ECL deviates -0.007		
3.8436	628	0.014	----	17.3155		----			
3.9010	33541	0.018	0.959	17.4032	17:0 10-methyl	0.76	ECL deviates -0.004		
3.9375	2355	0.011	0.959	17.4589	17:0 DMA	0.05	ECL deviates  0.001		
3.9602	10983	0.023	----	17.4935		----			
4.0152	10283	0.014	0.959	17.5777	18:3 w6c	0.23	ECL deviates -0.002		
4.0331	29322	0.024	----	17.6049		----			
4.1085	123316	0.018	0.959	17.7201	18:2 w6c	2.81	ECL deviates -0.007		
4.1435	299786	0.020	0.959	17.7735	18:1 w9c	6.83	ECL deviates -0.001		
4.1800	470417	0.017	0.959	17.8293	18:1 w7c	10.72	Column Overload		
4.2319	74472	0.021	----	17.9085		----			
4.2915	82225	0.019	0.959	17.9996	18:0	1.87	ECL deviates  0.000	Reference -0.004	
4.3470	29893	0.018	0.959	18.0801	18:1 w7c 10-methyl	0.68	ECL deviates -0.005		
4.4007	11106	0.026	0.959	18.1576	18:2 DMA	0.25	ECL deviates -0.002		
4.4483	3917	0.021	0.960	18.2265	18:1 w9c DMA	0.09	ECL deviates -0.011		
4.4794	6102	0.020	0.960	18.2714	18:1 w7c DMA	0.14	ECL deviates -0.011		
4.5089	2002	0.015	----	18.3141		----			
4.5597	118042	0.020	0.960	18.3875	18:0 10-methyl	2.69	ECL deviates -0.008		
4.6282	3505	0.021	0.960	18.4865	19:4 w6c	0.08	ECL deviates  0.002		
4.6729	9740	0.024	0.961	18.5511	19:3 w6c	0.22	ECL deviates -0.009		
4.7449	6916	0.025	0.961	18.6552	19:3 w3c	0.16	ECL deviates -0.003		
4.8078	13433	0.024	----	18.7463		----			
4.8485	16392	0.019	0.962	18.8051	19:1 w8c	0.37	ECL deviates -0.006		
4.8904	21424	0.018	0.962	18.8657	19:0 cyclo w9c	0.49	ECL deviates -0.006		
4.9151	102384	0.020	0.962	18.9014	19:0 cyclo w7c	2.34	ECL deviates -0.008		
4.9855	77283	0.018	----	19.0032	19:0	----	ECL deviates  0.003		
5.0459	4363	0.019	----	19.0874		----			
5.1365	2792	0.021	----	19.2137		----			
5.1724	14216	0.018	----	19.2637		----			
5.2450	21952	0.022	----	19.3649		----			
5.2571	17311	0.015	----	19.3818		----			
5.3133	10234	0.020	0.966	19.4601	20:5 w3c	0.23	ECL deviates -0.022		
5.3481	3568	0.016	----	19.5087		----			
5.3792	8606	0.022	----	19.5521		----			
5.4117	13195	0.026	----	19.5974		----			
5.5330	31599	0.027	0.967	19.7665	20:1 w9c	0.73	ECL deviates -0.006		
5.5598	12158	0.022	0.967	19.8038	20:1 w8c	0.28	ECL deviates -0.009		
5.6146	1503	0.018	----	19.8802		----			
5.7015	30261	0.025	0.969	20.0014	20:0	0.70	ECL deviates  0.001	Reference -0.002	
5.7555	2256	0.019	----	20.0760		----			
5.8016	3779	0.017	----	20.1397		----			
5.8335	9990	0.023	----	20.1839		----			
5.9120	5283	0.015	----	20.2923		----			
5.9226	1533	0.006	----	20.3070		----			
5.9447	7879	0.019	----	20.3375		----			
5.9768	39934	0.023	----	20.3819		----			
6.0505	1724	0.018	----	20.4837		----			
6.1006	6458	0.029	----	20.5530		----			
6.1473	7331	0.024	----	20.6175		----			
6.1687	3444	0.016	0.972	20.6471	21:3 w3c	0.08	ECL deviates -0.007		
6.2131	6108	0.031	----	20.7084		----			
6.2755	14079	0.021	0.972	20.7947	21:1 w8c	0.33	ECL deviates -0.003		
6.3341	13088	0.026	----	20.8757		----			
6.3928	20242	0.020	0.973	20.9568	21:1 w3c	0.47	ECL deviates  0.003		
6.4274	9115	0.024	0.973	21.0046	21:0	0.21	ECL deviates  0.005	Reference  0.001	
6.5069	4253	0.021	----	21.1141		----			
6.5508	1893	0.018	----	21.1745		----			
6.5920	5150	0.022	0.974	21.2313	22:5 w6c	0.12	ECL deviates -0.021		
6.6263	11033	0.021	----	21.2785		----			
6.6508	2213	0.012	0.974	21.3122	22:6 w3c	0.05	ECL deviates -0.020		
6.6915	1889	0.021	----	21.3683		----			
6.7492	2921	0.039	0.974	21.4477	22:5 w3c	----	> max ar/ht		
6.8753	15488	0.034	0.974	21.6214	22:0 iso	0.36	ECL deviates  0.004		
6.9534	3772	0.022	0.974	21.7290	22:2 w6c	0.09	ECL deviates -0.010		
6.9847	4950	0.021	0.974	21.7721	22:1 w9c	0.11	ECL deviates -0.001		
7.0229	8684	0.031	----	21.8247		----			
7.1047	7440	0.022	0.974	21.9372	22:1 w3c	0.17	ECL deviates -0.010		
7.1490	34435	0.021	0.974	21.9983	22:0	0.80	ECL deviates -0.002	Reference -0.006	
7.2118	1775	0.023	----	22.0861		----			
7.2422	2631	0.028	----	22.1287		----			
7.3220	11743	0.020	----	22.2404		----			
7.3804	2490	0.030	----	22.3222		----			
7.4391	2306	0.027	----	22.4043		----			
7.4919	1632	0.024	0.972	22.4784	23:4 w6c	0.04	ECL deviates  0.007		
7.5367	1763	0.022	----	22.5410		----			
7.6012	5491	0.051	----	22.6314		----	> max ar/ht		
7.7026	6430	0.024	----	22.7733		----			
7.7643	2529	0.021	----	22.8598		----			
7.8058	9026	0.020	0.969	22.9179	23:1 w4c	0.21	ECL deviates -0.009		
7.8647	8101	0.019	0.968	23.0005	23:0	0.19	ECL deviates  0.000	Reference -0.005	
7.9102	2276	0.026	----	23.0649		----			
8.0701	8282	0.021	----	23.2915		----			
8.2824	818	0.015	0.961	23.5925	24:3 w6c	0.02	ECL deviates  0.002		
8.3207	6593	0.024	0.960	23.6468	24:3 w3c	0.15	ECL deviates -0.008		
8.3789	2413	0.020	----	23.7292		----			
8.4114	3629	0.020	----	23.7752		----			
8.4846	1979	0.025	----	23.8791		----			
8.5684	23785	0.019	0.954	23.9979	24:0	0.54	ECL deviates -0.002	Reference -0.009	
8.6730	1155	0.018	----	24.1461		----	> max rt		
8.9245	11635	0.019	----	24.5027		----	> max rt		
9.1523	1103	0.016	----	24.8257		----	> max rt		
9.2259	13389	0.024	----	24.9300		----	> max rt		
9.2548	2612	0.014	----	24.9710		----	> max rt		
9.4637	10059	0.022	----	25.2673		----	> max rt		

ECL Deviation: 0.007                            Reference ECL Shift: 0.006       Number Reference Peaks: 20
Total Response: 4888982                       Total Named: 4334519
Percent Named: 88.66%                         Total Amount: 4210328
Profile Comment:   Column Overload:  A peak's response is greater than 400000.0.  Dilute and re-run.

(No search libraries specified in method PLFAD1.)
