# Supplementary material for: Microbial regulation of soil carbon properties under nitrogen addition and plant inputs removal
Source: PeerJ. 2019 Jul 17;7:e7343. doi: 10.7717/peerj.7343 (PMC6642627; doi:10.7717/peerj.7343)
Supplement: File S1 — The raw data showed the soil microbial PLFAs files in the year of 2015 and 2016. Each file of rtf. represented the microbial PLFAs for each soil sample. In the Supplemental File, the Excel file named “Numbers” showed the plots names and the related rtf. file names. [file peerj-07-7343-s002.zip › supplementary files/2015/41.rtf]

Volume: DATA            File: E164213.59A        Samp Ctr: 10                ID Number: 29338 
Type: Samp                   Bottle: 9                        Method: PLFAD1 
Created: 4/21/2016 12:54:05 PM 
Sample ID: 41 


RT	Response	Ar/Ht	RFact	ECL	Peak Name	Percent	Comment1	Comment2	
0.7143	1.908E+9	0.015	----	7.6454	SOLVENT PEAK	----	< min rt		
0.8867	681	0.008	----	8.7709		----	< min rt		
0.9449	969	0.013	----	9.1529		----	< min rt		
0.9724	737	0.014	----	9.3328		----	< min rt		
1.1871	4885	0.012	----	10.7333		----			
1.2631	1976	0.015	----	11.1688		----			
1.3205	892	0.015	1.172	11.4427	10:0 3OH	0.02	ECL deviates  0.001		
1.3532	1375	0.017	----	11.5984		----			
1.3668	463	0.008	----	11.6634		----			
1.3913	2078	0.015	----	11.7803		----			
1.4376	5716	0.016	1.127	12.0012	12:0	0.12	ECL deviates  0.001	Reference -0.002	
1.4954	4174	0.016	----	12.2096		----			
1.5242	639	0.012	----	12.3128		----			
1.5606	1985	0.020	----	12.4436		----			
1.6058	5440	0.013	1.085	12.6059	13:0 iso	0.11	ECL deviates -0.006	Reference -0.010	
1.6376	4572	0.019	1.079	12.7204	13:0 anteiso	0.10	ECL deviates  0.011	Reference  0.008	
1.6910	1244	0.015	1.067	12.9119	13:1 w5c	0.03	ECL deviates -0.008		
1.7158	2686	0.016	1.062	13.0009	13:0	0.06	ECL deviates  0.001	Reference -0.002	
1.7827	955	0.015	----	13.1881	12:0 2OH	----	ECL deviates  0.002		
1.8274	431	0.010	----	13.3130		----			
1.8762	2878	0.019	1.039	13.4490	13:0 DMA	0.06	ECL deviates -0.011		
1.9333	62398	0.014	1.032	13.6084	14:0 iso	1.25	ECL deviates -0.006	Reference -0.009	
1.9730	1548	0.012	1.028	13.7193	14:0 anteiso	0.03	ECL deviates  0.003	Reference  0.000	
1.9929	1596	0.011	1.025	13.7748	14:1 w9c	0.03	ECL deviates -0.003		
2.0082	2479	0.012	----	13.8174		----			
2.0422	3690	0.012	1.020	13.9122	14:1 w5c	0.07	ECL deviates  0.001		
2.0729	71288	0.014	1.016	13.9979	14:0	1.40	ECL deviates -0.002	Reference -0.005	
2.1011	869	0.011	----	14.0627		----			
2.1283	2067	0.015	----	14.1240	14:0 iso 3OH	----	ECL deviates -0.001		
2.1540	4369	0.025	----	14.1822		----			
2.2194	3494	0.021	----	14.3299		----			
2.2659	78078	0.020	1.001	14.4349	15:1 iso w6c	1.52	ECL deviates -0.004		
2.3062	20458	0.014	0.998	14.5259	15:1 anteiso w9c	0.40	ECL deviates -0.004		
2.3457	302982	0.014	0.996	14.6153	15:0 iso	5.85	ECL deviates -0.002	Reference -0.004	
2.3867	232148	0.014	0.993	14.7079	15:0 anteiso	4.47	ECL deviates -0.003	Reference -0.006	
2.4512	9379	0.021	0.989	14.8537	15:1 w6c	0.18	ECL deviates -0.006		
2.4715	2043	0.012	0.988	14.8994	15:1 w5c	0.04	ECL deviates -0.013		
2.5153	35471	0.015	0.985	14.9984	15:0	0.68	ECL deviates -0.002	Reference -0.004	
2.5440	8819	0.017	----	15.0538		----			
2.6058	2530	0.023	----	15.1715		----			
2.6354	6403	0.021	----	15.2279		----			
2.7470	56729	0.025	0.976	15.4404	15:0 DMA	1.07	ECL deviates -0.010		
2.8076	89106	0.016	0.974	15.5558	16:0 N alcohol	1.68	ECL deviates -0.001		
2.8411	135522	0.016	0.973	15.6197	16:0 iso	2.56	ECL deviates  0.000	Reference -0.003	
2.8926	12280	0.013	0.971	15.7178	16:0 anteiso	0.23	ECL deviates  0.003	Reference  0.000	
2.9197	65299	0.017	0.971	15.7693	16:1 w9c	1.23	ECL deviates -0.006		
2.9486	488362	0.017	0.970	15.8245	16:1 w7c	9.18	Column Overload		
2.9954	164483	0.016	0.969	15.9136	16:1 w5c	3.09	ECL deviates  0.002		
3.0463	672851	0.015	0.968	16.0096	16:0	12.62	Column Overload		
3.0712	17975	0.018	----	16.0513		----			
3.1236	5896	0.017	0.966	16.1391	16:2 DMA	0.11	ECL deviates  0.001		
3.1597	9266	0.024	----	16.1995		----			
3.1954	5317	0.019	----	16.2592		----			
3.2311	2759	0.020	0.964	16.3190	16:1 w7c DMA	0.05	ECL deviates  0.009		
3.2928	265231	0.020	0.963	16.4223	16:0 10-methyl	4.95	ECL deviates  0.002		
3.3292	60357	0.019	0.963	16.4833	17:1 iso w9c	1.13	ECL deviates -0.015		
3.3563	33691	0.018	0.962	16.5287	17:1 anteiso w9c	0.63	ECL deviates -0.007		
3.4123	70438	0.017	0.962	16.6225	17:0 iso	1.31	ECL deviates -0.001	Reference -0.004	
3.4694	87539	0.017	0.961	16.7180	17:0 anteiso	1.63	ECL deviates -0.002		
3.5136	55392	0.019	0.961	16.7921	17:1 w8c	1.03	ECL deviates -0.005		
3.5739	173247	0.018	0.960	16.8931	17:0 cyclo w7c	3.22	ECL deviates  0.000		
3.6376	30566	0.018	0.960	16.9998	17:0	0.57	ECL deviates  0.000	Reference -0.003	
3.6637	20382	0.017	0.959	17.0398	17:1 w7c 10-methyl	0.38	ECL deviates -0.003		
3.7056	7031	0.018	----	17.1039		----			
3.7381	2151	0.019	----	17.1535		----			
3.7911	6120	0.019	0.959	17.2345	16:0 2OH	0.11	ECL deviates -0.006		
3.8459	691	0.015	----	17.3183		----			
3.9010	35034	0.018	0.959	17.4024	17:0 10-methyl	0.65	ECL deviates -0.005		
3.9591	15762	0.028	----	17.4912		----			
4.0332	45955	0.030	----	17.6046		----			
4.1088	131475	0.017	0.959	17.7200	18:2 w6c	2.44	ECL deviates -0.007		
4.1446	390962	0.020	0.959	17.7748	18:1 w9c	7.27	ECL deviates  0.000		
4.1808	589921	0.017	0.959	17.8301	18:1 w7c	10.96	Column Overload		
4.2326	96562	0.021	----	17.9092		----			
4.2924	106722	0.017	0.959	18.0007	18:0	1.98	ECL deviates  0.001	Reference -0.002	
4.3468	36253	0.017	0.959	18.0794	18:1 w7c 10-methyl	0.67	ECL deviates -0.006		
4.4001	11001	0.027	0.959	18.1565	18:2 DMA	0.20	ECL deviates -0.004		
4.4471	5709	0.023	0.960	18.2244	18:1 w9c DMA	0.11	ECL deviates -0.013		
4.4817	3185	0.018	0.960	18.2744	18:1 w7c DMA	0.06	ECL deviates -0.008		
4.5100	2393	0.017	----	18.3155		----			
4.5602	144380	0.020	0.960	18.3879	18:0 10-methyl	2.69	ECL deviates -0.007		
4.6276	4553	0.019	0.960	18.4855	19:4 w6c	0.08	ECL deviates  0.001		
4.6744	10227	0.026	0.961	18.5531	19:3 w6c	0.19	ECL deviates -0.007		
4.7447	7947	0.025	0.961	18.6548	19:3 w3c	0.15	ECL deviates -0.003		
4.8072	16527	0.025	----	18.7453		----			
4.8495	17492	0.019	0.962	18.8064	19:1 w8c	0.33	ECL deviates -0.004		
4.8841	33324	0.019	0.962	18.8565	19:1 w6c	0.62	ECL deviates  0.005		
4.9162	138234	0.018	0.962	18.9029	19:0 cyclo w7c	2.58	ECL deviates -0.007		
4.9855	86284	0.017	----	19.0031	19:0	----	ECL deviates  0.003		
5.0460	4811	0.017	----	19.0875		----			
5.0766	2658	0.017	----	19.1301		----			
5.1348	3396	0.020	----	19.2113		----			
5.1718	27191	0.019	----	19.2628		----			
5.2442	24114	0.016	----	19.3639		----			
5.2595	26502	0.020	----	19.3851		----			
5.3130	10387	0.020	0.966	19.4597	20:5 w3c	0.19	ECL deviates -0.022		
5.3510	5970	0.017	----	19.5127		----			
5.3810	9893	0.020	----	19.5545		----			
5.4114	19271	0.028	----	19.5969		----			
5.5299	35809	0.029	0.967	19.7621	20:1 w9c	0.67	ECL deviates -0.010		
5.5599	15702	0.026	0.967	19.8040	20:1 w8c	0.29	ECL deviates -0.009		
5.7007	38665	0.022	0.969	20.0003	20:0	0.73	ECL deviates  0.000	Reference -0.003	
5.7556	2716	0.021	----	20.0762		----			
5.8036	4303	0.018	----	20.1425		----			
5.8332	13423	0.020	----	20.1835		----			
5.9120	7547	0.021	----	20.2924		----			
5.9439	8283	0.018	----	20.3365		----			
5.9748	40380	0.025	----	20.3792		----			
6.1010	6849	0.028	----	20.5536		----			
6.1502	10158	0.033	----	20.6215		----			
6.2245	7598	0.031	----	20.7242		----			
6.2757	16265	0.020	0.972	20.7950	21:1 w8c	0.31	ECL deviates -0.003		
6.3346	19171	0.023	----	20.8763		----			
6.3920	20389	0.022	0.973	20.9557	21:1 w3c	0.38	ECL deviates  0.002		
6.4274	11871	0.024	0.973	21.0046	21:0	0.22	ECL deviates  0.005	Reference  0.001	
6.5078	5141	0.023	----	21.1153		----			
6.5475	2090	0.020	----	21.1700		----			
6.5923	5262	0.021	0.974	21.2317	22:5 w6c	0.10	ECL deviates -0.020		
6.6254	21686	0.022	----	21.2772		----			
6.6912	1753	0.020	----	21.3677		----			
6.7469	2087	0.028	----	21.4445		----			
6.7754	1419	0.021	0.974	21.4837	22:5 w3c	0.03	ECL deviates  0.016		
6.8759	19023	0.029	0.974	21.6220	22:0 iso	0.36	ECL deviates  0.004		
6.9549	4430	0.025	0.974	21.7308	22:2 w6c	0.08	ECL deviates -0.008		
6.9892	5340	0.023	0.974	21.7780	22:1 w9c	0.10	ECL deviates  0.005		
7.0194	8252	0.030	0.974	21.8196	22:1 w8c	0.16	ECL deviates  0.006		
7.1038	6958	0.022	0.974	21.9357	22:1 w3c	0.13	ECL deviates -0.011		
7.1496	51783	0.022	0.974	21.9988	22:0	0.98	ECL deviates -0.001	Reference -0.005	
7.2090	2581	0.023	----	22.0819		----			
7.2428	3269	0.032	----	22.1292		----			
7.3241	12863	0.022	----	22.2429		----			
7.3768	2445	0.028	----	22.3168		----			
7.4422	2889	0.030	----	22.4083		----			
7.4925	1581	0.021	0.972	22.4786	23:4 w6c	0.03	ECL deviates  0.008		
7.5399	2700	0.028	----	22.5451		----			
7.6034	4244	0.034	----	22.6340		----			
7.6361	1469	0.018	----	22.6798		----			
7.7017	9235	0.025	----	22.7716		----			
7.7661	3896	0.021	----	22.8617		----			
7.8065	11516	0.020	0.969	22.9182	23:1 w4c	0.22	ECL deviates -0.008		
7.8659	10856	0.021	0.968	23.0014	23:0	0.20	ECL deviates  0.001	Reference -0.003	
7.9070	3337	0.027	----	23.0596		----			
8.0702	14306	0.023	----	23.2907		----			
8.2837	1361	0.017	0.961	23.5931	24:3 w6c	0.03	ECL deviates  0.003		
8.3214	8682	0.024	----	23.6466		----			
8.3770	2602	0.022	----	23.7253		----			
8.4116	3974	0.022	----	23.7743		----			
8.4840	2849	0.028	----	23.8769		----			
8.5185	752	0.015	----	23.9257		----			
8.5689	32216	0.021	0.954	23.9971	24:0	0.60	ECL deviates -0.003	Reference -0.008	
8.6734	1555	0.019	----	24.1452		----	> max rt		
8.9249	10462	0.021	----	24.5015		----	> max rt		
9.1551	1098	0.018	----	24.8276		----	> max rt		
9.2280	19355	0.024	----	24.9309		----	> max rt		
9.4651	13852	0.022	----	25.2668		----	> max rt		

ECL Deviation: 0.007                            Reference ECL Shift: 0.005       Number Reference Peaks: 20
Total Response: 5941938                       Total Named: 5316259
Percent Named: 89.47%                         Total Amount: 5158177
Profile Comment:   Column Overload:  A peak's response is greater than 400000.0.  Dilute and re-run.

(No search libraries specified in method PLFAD1.)
