# Supplementary material for: Microbial regulation of soil carbon properties under nitrogen addition and plant inputs removal
Source: PeerJ. 2019 Jul 17;7:e7343. doi: 10.7717/peerj.7343 (PMC6642627; doi:10.7717/peerj.7343)
Supplement: File S1 — The raw data showed the soil microbial PLFAs files in the year of 2015 and 2016. Each file of rtf. represented the microbial PLFAs for each soil sample. In the Supplemental File, the Excel file named “Numbers” showed the plots names and the related rtf. file names. [file peerj-07-7343-s002.zip › supplementary files/2015/42.rtf]

Volume: DATA            File: E164213.59A        Samp Ctr: 11                ID Number: 29339 
Type: Samp                   Bottle: 10                      Method: PLFAD1 
Created: 4/21/2016 1:22:33 PM 
Sample ID: 42 


RT	Response	Ar/Ht	RFact	ECL	Peak Name	Percent	Comment1	Comment2	
0.7141	1.905E+9	0.015	----	7.6424	SOLVENT PEAK	----	< min rt		
0.8862	942	0.011	----	8.7664		----	< min rt		
0.9454	628	0.014	----	9.1556		----	< min rt		
1.1871	3332	0.012	----	10.7325		----			
1.2627	522	0.011	----	11.1662		----			
1.3536	1294	0.023	----	11.5996		----			
1.3668	504	0.009	----	11.6626		----			
1.3904	1607	0.014	----	11.7753		----			
1.4376	3358	0.015	1.127	12.0007	12:0	0.10	ECL deviates  0.001	Reference -0.002	
1.4953	2580	0.017	----	12.2084		----			
1.5209	420	0.009	----	12.3002		----			
1.5603	1786	0.018	----	12.4419		----			
1.6060	4336	0.012	1.085	12.6058	13:0 iso	0.12	ECL deviates -0.007	Reference -0.009	
1.6377	3672	0.017	1.079	12.7198	13:0 anteiso	0.10	ECL deviates  0.010	Reference  0.008	
1.6911	1063	0.015	1.067	12.9116	13:1 w5c	0.03	ECL deviates -0.008		
1.7156	1479	0.013	1.062	12.9997	13:0	0.04	ECL deviates  0.000	Reference -0.003	
1.7827	842	0.018	----	13.1876	12:0 2OH	----	ECL deviates  0.001		
1.8729	2149	0.019	----	13.4393		----			
1.9336	61960	0.013	1.032	13.6087	14:0 iso	1.61	ECL deviates -0.005	Reference -0.008	
1.9740	1178	0.011	1.028	13.7215	14:0 anteiso	0.03	ECL deviates  0.006	Reference  0.003	
1.9937	1275	0.010	1.025	13.7764	14:1 w9c	0.03	ECL deviates -0.001		
2.0082	2180	0.013	----	13.8168		----			
2.0409	3523	0.014	1.020	13.9082	14:1 w5c	0.09	ECL deviates -0.003		
2.0728	45449	0.014	1.016	13.9973	14:0	1.16	ECL deviates -0.003	Reference -0.005	
2.0998	1002	0.012	----	14.0591		----			
2.1296	1454	0.015	----	14.1265	14:0 iso 3OH	----	ECL deviates  0.002		
2.1536	3529	0.022	----	14.1808		----			
2.2207	2588	0.020	----	14.3324		----			
2.2665	49869	0.018	1.001	14.4359	15:1 iso w6c	1.25	ECL deviates -0.003		
2.2853	11400	0.013	0.999	14.4783	15:4 w3c	0.29	ECL deviates -0.012		
2.3068	12667	0.014	0.998	14.5269	15:1 anteiso w9c	0.32	ECL deviates -0.003		
2.3457	248149	0.013	0.996	14.6149	15:0 iso	6.21	ECL deviates -0.002	Reference -0.004	
2.3869	188727	0.014	0.993	14.7079	15:0 anteiso	4.71	ECL deviates -0.003	Reference -0.005	
2.4520	9172	0.025	0.989	14.8550	15:1 w6c	0.23	ECL deviates -0.005		
2.5160	23870	0.015	0.985	14.9996	15:0	0.59	ECL deviates  0.000	Reference -0.003	
2.5438	12408	0.017	----	15.0531		----			
2.6059	3220	0.018	----	15.1712		----			
2.6367	3284	0.020	----	15.2300		----			
2.7216	8655	0.016	0.977	15.3917	16:1 w7c alcohol	0.21	ECL deviates -0.005		
2.7478	40440	0.021	0.976	15.4415	15:0 DMA	0.99	ECL deviates -0.009		
2.8084	100839	0.016	0.974	15.5571	16:0 N alcohol	2.47	ECL deviates  0.000		
2.8408	99477	0.016	0.973	15.6188	16:0 iso	2.43	ECL deviates -0.001	Reference -0.003	
2.8935	9245	0.013	0.971	15.7192	16:0 anteiso	0.23	ECL deviates  0.004	Reference  0.002	
2.9186	61267	0.017	0.971	15.7670	16:1 w9c	1.49	ECL deviates -0.008		
2.9480	478446	0.017	0.970	15.8229	16:1 w7c	11.66	Column Overload		
2.9948	132616	0.015	0.969	15.9122	16:1 w5c	3.23	ECL deviates  0.001		
3.0437	424513	0.016	0.968	16.0049	16:0	10.32	Column Overload		
3.0713	29176	0.020	----	16.0511		----			
3.1238	2812	0.016	0.966	16.1391	16:2 DMA	0.07	ECL deviates  0.001		
3.1588	6609	0.022	----	16.1977		----			
3.1930	5140	0.019	----	16.2550		----			
3.2364	2610	0.021	0.964	16.3277	16:1 w7c DMA	0.06	ECL deviates  0.018		
3.2934	276169	0.020	0.963	16.4231	16:0 10-methyl	6.69	ECL deviates  0.003		
3.3282	58886	0.018	----	16.4814		----			
3.3564	28281	0.018	0.962	16.5287	17:1 anteiso w9c	0.68	ECL deviates -0.007		
3.4120	61989	0.017	0.962	16.6218	17:0 iso	1.50	ECL deviates -0.002	Reference -0.004	
3.4697	67715	0.018	0.961	16.7183	17:0 anteiso	1.64	ECL deviates -0.002		
3.5140	38808	0.017	0.961	16.7926	17:1 w8c	0.94	ECL deviates -0.004		
3.5729	149463	0.019	0.960	16.8913	17:0 cyclo w7c	3.61	ECL deviates -0.002		
3.6383	17030	0.018	0.960	17.0008	17:0	0.41	ECL deviates  0.001	Reference -0.002	
3.6638	31634	0.016	0.959	17.0399	17:1 w7c 10-methyl	0.76	ECL deviates -0.003		
3.7062	7917	0.017	----	17.1047		----			
3.7430	2170	0.020	----	17.1609		----			
3.7917	2588	0.018	0.959	17.2354	16:0 2OH	0.06	ECL deviates -0.005		
3.8467	742	0.015	----	17.3194		----			
3.9021	23645	0.019	0.959	17.4040	17:0 10-methyl	0.57	ECL deviates -0.003		
3.9378	2694	0.013	0.959	17.4587	17:0 DMA	0.06	ECL deviates  0.001		
3.9592	6759	0.021	----	17.4914		----			
4.0336	32258	0.029	----	17.6050		----			
4.1095	57775	0.018	0.959	17.7211	18:2 w6c	1.39	ECL deviates -0.006		
4.1421	256325	0.020	0.959	17.7708	18:1 w9c	6.18	ECL deviates -0.004		
4.1796	426425	0.017	0.959	17.8282	18:1 w7c	10.28	Column Overload		
4.2371	56353	0.021	0.959	17.9161	18:1 w5c	1.36	ECL deviates -0.007		
4.2933	67253	0.018	0.959	18.0020	18:0	1.62	ECL deviates  0.002	Reference -0.001	
4.3481	23714	0.018	0.959	18.0812	18:1 w7c 10-methyl	0.57	ECL deviates -0.004		
4.4030	4840	0.016	0.959	18.1607	18:2 DMA	0.12	ECL deviates  0.001		
4.4171	4638	0.015	----	18.1811		----			
4.4519	6561	0.029	0.960	18.2314	18:1 w9c DMA	0.16	ECL deviates -0.006		
4.5090	1841	0.017	----	18.3140		----			
4.5614	103034	0.021	0.960	18.3898	18:0 10-methyl	2.49	ECL deviates -0.005		
4.6310	3233	0.021	0.960	18.4905	19:4 w6c	0.08	ECL deviates  0.006		
4.6751	11201	0.024	0.961	18.5542	19:3 w6c	0.27	ECL deviates -0.006		
4.7282	2215	0.013	0.961	18.6310	19:0 iso	0.05	ECL deviates  0.001		
4.7398	2691	0.016	----	18.6478		----			
4.8070	13829	0.019	----	18.7450		----			
4.8517	13500	0.020	0.962	18.8097	19:1 w8c	0.33	ECL deviates -0.001		
4.8869	17792	0.016	0.962	18.8606	19:1 w6c	0.43	ECL deviates  0.009		
4.9164	109038	0.019	0.962	18.9032	19:0 cyclo w7c	2.64	ECL deviates -0.007		
4.9857	87035	0.019	----	19.0036	19:0	----	ECL deviates  0.004		
5.0463	1897	0.017	----	19.0880		----			
5.0871	795	0.017	----	19.1449		----			
5.1384	3223	0.020	----	19.2164		----			
5.1732	8405	0.019	----	19.2650		----			
5.2595	25030	0.028	----	19.3854		----			
5.3143	11255	0.019	0.966	19.4618	20:5 w3c	0.27	ECL deviates -0.020		
5.3502	2041	0.016	----	19.5119		----			
5.3808	6302	0.020	----	19.5545		----			
5.4122	9420	0.023	----	19.5984		----			
5.5309	27126	0.026	0.967	19.7638	20:1 w9c	0.66	ECL deviates -0.009		
5.5638	12618	0.025	0.967	19.8098	20:1 w8c	0.31	ECL deviates -0.003		
5.6463	631	0.015	0.968	19.9248	20:1 w4c	0.02	ECL deviates -0.006		
5.6989	21116	0.023	0.969	19.9982	20:0	0.51	ECL deviates -0.002	Reference -0.005	
5.7561	975	0.017	----	20.0772		----			
5.8021	3019	0.020	----	20.1408		----			
5.8335	7197	0.021	----	20.1842		----			
5.9439	10890	0.021	----	20.3369		----			
5.9750	54718	0.023	----	20.3798		----			
6.0466	1359	0.016	----	20.4788		----			
6.0817	1668	0.016	----	20.5272		----			
6.1018	2110	0.018	----	20.5550		----			
6.1474	11447	0.028	----	20.6182		----			
6.2112	4798	0.029	----	20.7064		----			
6.2772	12533	0.018	0.972	20.7976	21:1 w8c	0.31	ECL deviates  0.000		
6.3341	8716	0.025	----	20.8762		----			
6.3913	26609	0.020	0.973	20.9553	21:1 w3c	0.65	ECL deviates  0.001		
6.4262	6378	0.022	0.973	21.0035	21:0	0.16	ECL deviates  0.003	Reference -0.001	
6.5093	4292	0.020	----	21.1179		----			
6.5528	1328	0.018	----	21.1778		----			
6.5917	4287	0.021	0.974	21.2314	22:5 w6c	0.10	ECL deviates -0.021		
6.6258	7506	0.024	----	21.2783		----			
6.6897	1130	0.019	----	21.3663		----			
6.7517	1880	0.029	0.974	21.4517	22:5 w3c	0.05	ECL deviates -0.016		
6.8206	1087	0.018	----	21.5466		----			
6.8767	12146	0.030	0.974	21.6238	22:0 iso	0.30	ECL deviates  0.006		
6.9529	4101	0.027	0.974	21.7286	22:2 w6c	0.10	ECL deviates -0.010		
6.9870	3094	0.022	0.974	21.7757	22:1 w9c	0.08	ECL deviates  0.003		
7.0225	5297	0.027	----	21.8245		----			
7.1038	6776	0.021	0.974	21.9365	22:1 w3c	0.17	ECL deviates -0.010		
7.1489	20397	0.019	0.974	21.9985	22:0	0.50	ECL deviates -0.001	Reference -0.006	
7.2109	4420	0.033	----	22.0852		----			
7.3224	13988	0.018	----	22.2414		----			
7.3791	1428	0.020	----	22.3207		----			
7.4035	798	0.015	----	22.3549		----			
7.4395	1723	0.024	----	22.4053		----			
7.4957	1628	0.026	0.972	22.4840	23:4 w6c	0.04	ECL deviates  0.013		
7.5385	976	0.021	----	22.5440		----			
7.6105	4868	0.044	0.971	22.6448	23:3 w3c	----	> max ar/ht		
7.7030	3950	0.021	----	22.7742		----			
7.7636	1905	0.022	----	22.8591		----			
7.8063	11269	0.019	0.969	22.9189	23:1 w4c	0.27	ECL deviates -0.008		
7.8650	4817	0.019	0.968	23.0011	23:0	0.12	ECL deviates  0.001	Reference -0.004	
7.9125	1878	0.027	----	23.0684		----			
8.0708	5721	0.019	----	23.2926		----			
8.3206	8210	0.025	----	23.6465		----			
8.3785	1989	0.021	----	23.7286		----			
8.4122	2763	0.024	----	23.7763		----			
8.4864	2091	0.024	----	23.8815		----			
8.5197	876	0.017	----	23.9286		----			
8.5691	18613	0.020	0.954	23.9986	24:0	0.45	ECL deviates -0.001	Reference -0.008	
8.9268	10951	0.019	----	24.5055		----	> max rt		
9.2289	22339	0.022	----	24.9336		----	> max rt		
9.4649	9729	0.019	----	25.2680		----	> max rt		

ECL Deviation: 0.007                            Reference ECL Shift: 0.005       Number Reference Peaks: 20
Total Response: 4555912                       Total Named: 4098616
Percent Named: 89.96%                         Total Amount: 3983005
Profile Comment:   Column Overload:  A peak's response is greater than 400000.0.  Dilute and re-run.

(No search libraries specified in method PLFAD1.)
