# Supplementary material for: Microbial regulation of soil carbon properties under nitrogen addition and plant inputs removal
Source: PeerJ. 2019 Jul 17;7:e7343. doi: 10.7717/peerj.7343 (PMC6642627; doi:10.7717/peerj.7343)
Supplement: File S1 — The raw data showed the soil microbial PLFAs files in the year of 2015 and 2016. Each file of rtf. represented the microbial PLFAs for each soil sample. In the Supplemental File, the Excel file named “Numbers” showed the plots names and the related rtf. file names. [file peerj-07-7343-s002.zip › supplementary files/2015/43.rtf]

Volume: DATA            File: E164213.59A        Samp Ctr: 12                ID Number: 29340 
Type: Samp                   Bottle: 11                      Method: PLFAD1 
Created: 4/21/2016 1:51:00 PM 
Sample ID: 43 


RT	Response	Ar/Ht	RFact	ECL	Peak Name	Percent	Comment1	Comment2	
0.7143	1.884E+9	0.014	----	7.6485	SOLVENT PEAK	----	< min rt		
0.8863	937	0.011	----	8.7713		----	< min rt		
0.9455	777	0.011	----	9.1603		----	< min rt		
1.1872	3895	0.011	----	10.7367		----			
1.2625	1042	0.012	----	11.1686		----			
1.3538	1103	0.017	1.157	11.6041	12:0 iso	0.03	ECL deviates -0.008		
1.3652	518	0.009	----	11.6584		----			
1.3910	1356	0.015	----	11.7814		----			
1.4372	3865	0.015	1.127	12.0021	12:0	0.09	ECL deviates  0.002	Reference -0.004	
1.4947	2538	0.017	----	12.2095		----			
1.5230	453	0.012	----	12.3110		----			
1.5597	1809	0.016	----	12.4429		----			
1.6054	4956	0.012	1.085	12.6071	13:0 iso	0.11	ECL deviates -0.005	Reference -0.011	
1.6368	3854	0.019	1.079	12.7196	13:0 anteiso	0.08	ECL deviates  0.010	Reference  0.005	
1.6907	1278	0.016	1.067	12.9132	13:1 w5c	0.03	ECL deviates -0.007		
1.7151	1928	0.013	1.062	13.0008	13:0	0.04	ECL deviates  0.001	Reference -0.005	
1.7814	1132	0.016	----	13.1868	12:0 2OH	----	ECL deviates  0.001		
1.8732	2533	0.019	----	13.4430		----			
1.9331	70245	0.013	1.032	13.6101	14:0 iso	1.43	ECL deviates -0.004	Reference -0.009	
1.9696	1168	0.011	1.028	13.7120	14:0 anteiso	0.02	ECL deviates -0.004	Reference -0.009	
1.9925	1701	0.011	1.025	13.7760	14:1 w9c	0.03	ECL deviates -0.002		
2.0073	2595	0.014	----	13.8172		----			
2.0401	3960	0.014	1.020	13.9087	14:1 w5c	0.08	ECL deviates -0.002		
2.0725	58759	0.013	1.016	13.9991	14:0	1.18	ECL deviates -0.001	Reference -0.006	
2.1011	1048	0.012	----	14.0649		----			
2.1282	1525	0.014	----	14.1260	14:0 iso 3OH	----	ECL deviates  0.001		
2.1535	3572	0.023	----	14.1833		----			
2.2096	2526	0.021	----	14.3100		----			
2.2657	68153	0.017	1.001	14.4367	15:1 iso w6c	1.35	ECL deviates -0.002		
2.2844	12315	0.012	0.999	14.4789	15:4 w3c	0.24	ECL deviates -0.011		
2.3063	17325	0.014	0.998	14.5284	15:1 anteiso w9c	0.34	ECL deviates -0.002		
2.3452	326693	0.014	0.996	14.6163	15:0 iso	6.44	ECL deviates -0.001	Reference -0.006	
2.3867	236595	0.014	0.993	14.7100	15:0 anteiso	4.65	ECL deviates -0.001	Reference -0.006	
2.4511	11744	0.024	0.989	14.8556	15:1 w6c	0.23	ECL deviates -0.004		
2.5148	30330	0.015	0.985	14.9995	15:0	0.59	ECL deviates  0.000	Reference -0.005	
2.5430	14626	0.017	----	15.0541		----			
2.5848	1043	0.012	----	15.1337		----			
2.6054	2833	0.016	----	15.1729		----			
2.6344	5124	0.020	----	15.2280		----			
2.7205	10515	0.016	0.977	15.3921	16:1 w7c alcohol	0.20	ECL deviates -0.005		
2.7475	52900	0.020	0.976	15.4436	15:0 DMA	1.02	ECL deviates -0.007		
2.8075	96719	0.015	0.974	15.5577	16:0 N alcohol	1.86	ECL deviates  0.001		
2.8398	129027	0.016	0.973	15.6192	16:0 iso	2.48	ECL deviates  0.000	Reference -0.005	
2.8915	13933	0.015	0.971	15.7177	16:0 anteiso	0.27	ECL deviates  0.003	Reference -0.002	
2.9185	72074	0.017	0.971	15.7693	16:1 w9c	1.38	ECL deviates -0.006		
2.9480	573700	0.017	0.970	15.8253	16:1 w7c	11.01	Column Overload		
2.9940	185388	0.016	0.969	15.9130	16:1 w5c	3.55	ECL deviates  0.002		
3.0440	567531	0.015	0.968	16.0078	16:0	10.87	Column Overload		
3.0696	31302	0.020	----	16.0507		----			
3.1230	3752	0.015	0.966	16.1400	16:2 DMA	0.07	ECL deviates  0.002		
3.1570	8937	0.023	----	16.1970		----			
3.1944	5816	0.020	----	16.2596		----			
3.2291	2941	0.020	0.964	16.3177	16:1 w7c DMA	0.06	ECL deviates  0.008		
3.2936	331029	0.020	0.963	16.4257	16:0 10-methyl	6.31	ECL deviates  0.006		
3.3269	63583	0.018	----	16.4814		----			
3.3552	35661	0.018	0.962	16.5288	17:1 anteiso w9c	0.68	ECL deviates -0.007		
3.4110	78693	0.017	0.962	16.6222	17:0 iso	1.50	ECL deviates -0.001	Reference -0.006	
3.4683	87749	0.017	0.961	16.7181	17:0 anteiso	1.67	ECL deviates -0.002		
3.5120	56400	0.018	0.961	16.7913	17:1 w8c	1.07	ECL deviates -0.006		
3.5716	177561	0.019	0.960	16.8912	17:0 cyclo w7c	3.37	ECL deviates -0.002		
3.6364	23942	0.018	0.960	16.9997	17:0	0.45	ECL deviates  0.000	Reference -0.005	
3.6624	36823	0.017	0.959	17.0398	17:1 w7c 10-methyl	0.70	ECL deviates -0.003		
3.7047	9564	0.018	----	17.1044		----			
3.7376	2252	0.017	----	17.1548		----			
3.7890	3796	0.019	0.959	17.2333	16:0 2OH	0.07	ECL deviates -0.007		
3.8424	726	0.013	----	17.3149		----			
3.8996	32679	0.018	0.959	17.4023	17:0 10-methyl	0.62	ECL deviates -0.005		
3.9366	3049	0.013	0.959	17.4588	17:0 DMA	0.06	ECL deviates  0.001		
3.9578	8411	0.023	----	17.4912		----			
4.0141	9336	0.013	0.959	17.5773	18:3 w6c	0.18	ECL deviates -0.003		
4.0328	32014	0.025	----	17.6057		----			
4.1079	105247	0.019	0.959	17.7205	18:2 w6c	2.00	ECL deviates -0.007		
4.1416	333882	0.020	0.959	17.7720	18:1 w9c	6.33	ECL deviates -0.003		
4.1790	502307	0.016	0.959	17.8292	18:1 w7c	9.53	Column Overload		
4.2341	79659	0.021	0.959	17.9134	18:1 w5c	1.51	ECL deviates -0.010		
4.2912	88012	0.018	0.959	18.0006	18:0	1.67	ECL deviates  0.001	Reference -0.004	
4.3456	28167	0.018	0.959	18.0796	18:1 w7c 10-methyl	0.53	ECL deviates -0.005		
4.3989	11497	0.028	0.959	18.1566	18:2 DMA	0.22	ECL deviates -0.003		
4.4459	6759	0.023	0.960	18.2245	18:1 w9c DMA	0.13	ECL deviates -0.012		
4.4755	2130	0.015	----	18.2674		----			
4.5073	1544	0.016	----	18.3134		----			
4.5604	126727	0.020	0.960	18.3901	18:0 10-methyl	2.41	ECL deviates -0.005		
4.6278	3600	0.020	0.960	18.4875	19:4 w6c	0.07	ECL deviates  0.003		
4.6722	11706	0.024	0.961	18.5518	19:3 w6c	0.22	ECL deviates -0.008		
4.7263	2906	0.016	0.961	18.6300	19:0 iso	0.06	ECL deviates  0.000		
4.7421	2858	0.016	0.961	18.6529	19:3 w3c	0.05	ECL deviates -0.005		
4.8039	17511	0.022	----	18.7423		----			
4.8505	17290	0.022	0.962	18.8097	19:1 w8c	0.33	ECL deviates -0.001		
4.9144	134316	0.022	0.962	18.9020	19:0 cyclo w7c	2.56	ECL deviates -0.008		
4.9845	92950	0.020	----	19.0034	19:0	----	ECL deviates  0.003		
5.0439	2506	0.016	----	19.0863		----			
5.0852	626	0.015	----	19.1439		----			
5.1385	3444	0.022	----	19.2181		----			
5.1719	9401	0.021	----	19.2647		----			
5.2129	2916	0.011	0.965	19.3219	19:0 cyclo 9,10 DMA	0.06	ECL deviates -0.002		
5.2584	36419	0.027	0.965	19.3854	20:4 w6c	0.70	ECL deviates -0.018		
5.3121	16000	0.020	0.966	19.4602	20:5 w3c	0.31	ECL deviates -0.022		
5.3479	2605	0.016	----	19.5102		----			
5.3766	8417	0.020	----	19.5502		----			
5.4116	12788	0.026	----	19.5990		----			
5.5306	34722	0.025	0.967	19.7649	20:1 w9c	0.66	ECL deviates -0.008		
5.5610	14818	0.023	0.967	19.8073	20:1 w8c	0.28	ECL deviates -0.006		
5.6999	26783	0.022	0.969	20.0010	20:0	0.51	ECL deviates  0.001	Reference -0.004	
5.7535	1378	0.017	----	20.0751		----			
5.8000	3718	0.019	----	20.1394		----			
5.8322	8225	0.020	----	20.1838		----			
5.9449	11332	0.025	----	20.3395		----			
5.9740	52882	0.023	----	20.3797		----			
6.0526	1413	0.014	----	20.4884		----			
6.0826	1755	0.014	----	20.5299		----			
6.1005	3136	0.019	----	20.5545		----			
6.1446	8878	0.022	----	20.6155		----			
6.1722	2731	0.012	0.972	20.6536	21:3 w3c	0.05	ECL deviates  0.000		
6.2124	6429	0.029	----	20.7091		----			
6.2746	14607	0.019	0.972	20.7952	21:1 w8c	0.28	ECL deviates -0.003		
6.3326	10494	0.025	----	20.8753		----			
6.3909	29496	0.021	0.973	20.9558	21:1 w3c	0.57	ECL deviates  0.002		
6.4255	6955	0.024	0.973	21.0036	21:0	0.13	ECL deviates  0.004	Reference -0.002	
6.5066	5472	0.024	----	21.1154		----			
6.5491	1492	0.017	----	21.1738		----			
6.5935	5528	0.022	0.974	21.2349	22:5 w6c	0.11	ECL deviates -0.017		
6.6253	8390	0.024	----	21.2787		----			
6.6889	1316	0.017	----	21.3663		----			
6.7555	2408	0.024	0.974	21.4579	22:5 w3c	0.05	ECL deviates -0.010		
6.8102	1295	0.017	----	21.5332		----			
6.8734	15717	0.027	0.974	21.6202	22:0 iso	0.30	ECL deviates  0.002		
6.9496	4857	0.028	0.974	21.7251	22:2 w6c	0.09	ECL deviates -0.013		
6.9834	3654	0.020	0.974	21.7716	22:1 w9c	0.07	ECL deviates -0.001		
7.0176	6410	0.025	0.974	21.8188	22:1 w8c	0.12	ECL deviates  0.005		
7.1022	7421	0.018	0.974	21.9352	22:1 w3c	0.14	ECL deviates -0.012		
7.1484	27116	0.019	0.974	21.9988	22:0	0.52	ECL deviates -0.001	Reference -0.007	
7.2072	2414	0.021	----	22.0810		----			
7.2469	876	0.018	----	22.1366		----			
7.3212	14630	0.018	----	22.2406		----			
7.3756	1080	0.019	----	22.3168		----			
7.3974	704	0.015	----	22.3472		----			
7.4363	1367	0.021	----	22.4017		----			
7.4940	935	0.018	0.972	22.4824	23:4 w6c	0.02	ECL deviates  0.011		
7.5335	1019	0.018	----	22.5377		----			
7.6045	3665	0.030	0.971	22.6371	23:3 w3c	0.07	ECL deviates -0.008		
7.6429	1611	0.019	----	22.6909		----			
7.7023	3582	0.021	----	22.7740		----			
7.7609	2725	0.026	----	22.8561		----			
7.8061	11694	0.020	0.969	22.9193	23:1 w4c	0.22	ECL deviates -0.007		
7.8648	6625	0.020	0.968	23.0015	23:0	0.13	ECL deviates  0.002	Reference -0.005	
7.9104	2295	0.023	----	23.0661		----			
8.0713	6296	0.022	----	23.2939		----			
8.2817	883	0.016	0.961	23.5920	24:3 w6c	0.02	ECL deviates  0.002		
8.3202	11248	0.022	----	23.6464		----			
8.3777	3036	0.021	----	23.7280		----			
8.4128	4179	0.022	0.958	23.7776	24:1 w9c	0.08	ECL deviates -0.009		
8.4846	2835	0.024	----	23.8793		----			
8.5210	936	0.017	----	23.9309		----			
8.5678	23761	0.020	0.954	23.9972	24:0	0.45	ECL deviates -0.003	Reference -0.009	
8.6709	868	0.018	----	24.1433		----	> max rt		
8.9230	8832	0.020	----	24.5004		----	> max rt		
9.0321	1228	0.023	----	24.6550		----	> max rt		
9.2253	22556	0.022	----	24.9287		----	> max rt		
9.4633	9009	0.023	----	25.2658		----	> max rt		

ECL Deviation: 0.006                            Reference ECL Shift: 0.006       Number Reference Peaks: 20
Total Response: 5661746                       Total Named: 5206401
Percent Named: 91.96%                         Total Amount: 5053061
Profile Comment:   Column Overload:  A peak's response is greater than 400000.0.  Dilute and re-run.

(No search libraries specified in method PLFAD1.)
