# Supplementary material for: Microbial regulation of soil carbon properties under nitrogen addition and plant inputs removal
Source: PeerJ. 2019 Jul 17;7:e7343. doi: 10.7717/peerj.7343 (PMC6642627; doi:10.7717/peerj.7343)
Supplement: File S1 — The raw data showed the soil microbial PLFAs files in the year of 2015 and 2016. Each file of rtf. represented the microbial PLFAs for each soil sample. In the Supplemental File, the Excel file named “Numbers” showed the plots names and the related rtf. file names. [file peerj-07-7343-s002.zip › supplementary files/2015/44.rtf]

Volume: DATA            File: E164213.59A        Samp Ctr: 13                ID Number: 29341 
Type: Samp                   Bottle: 12                      Method: PLFAD1 
Created: 4/21/2016 2:19:28 PM 
Sample ID: 44 


RT	Response	Ar/Ht	RFact	ECL	Peak Name	Percent	Comment1	Comment2	
0.7146	1.897E+9	0.014	----	7.6374	SOLVENT PEAK	----	< min rt		
0.8856	740	0.010	----	8.7567		----	< min rt		
0.9445	1025	0.013	----	9.1419		----	< min rt		
0.9713	436	0.010	----	9.3167		----	< min rt		
1.1861	3983	0.012	1.242	10.7215	11:0 anteiso	0.14	ECL deviates  0.016	Reference  0.018	
1.2614	742	0.013	----	11.1566		----			
1.3179	626	0.015	1.172	11.4263	10:0 3OH	0.02	ECL deviates -0.015		
1.3529	1106	0.019	----	11.5937	Phthalate 1	----	ECL deviates  0.006		
1.3650	433	0.008	----	11.6518		----			
1.3897	1838	0.015	----	11.7696		----			
1.4363	3286	0.015	1.127	11.9924	12:0	0.10	ECL deviates -0.008	Reference -0.008	
1.4940	3161	0.019	----	12.2022		----			
1.5214	549	0.010	----	12.3006		----			
1.5592	2076	0.023	----	12.4366		----			
1.6048	3907	0.013	1.085	12.6008	13:0 iso	0.12	ECL deviates -0.012	Reference -0.013	
1.6365	4171	0.020	1.079	12.7150	13:0 anteiso	0.12	ECL deviates  0.006	Reference  0.004	
1.6896	1210	0.014	----	12.9059		----			
1.7144	1565	0.014	1.062	12.9952	13:0	0.05	ECL deviates -0.005	Reference -0.007	
1.7822	1159	0.021	----	13.1862	12:0 2OH	----	ECL deviates  0.000		
1.8726	2173	0.020	----	13.4388		----			
1.9324	45395	0.014	1.032	13.6060	14:0 iso	1.29	ECL deviates -0.008	Reference -0.011	
1.9734	1403	0.013	1.028	13.7206	14:0 anteiso	0.04	ECL deviates  0.005	Reference  0.002	
1.9925	1242	0.011	1.025	13.7738	14:1 w9c	0.04	ECL deviates -0.004		
2.0072	2053	0.014	----	13.8149		----			
2.0720	43978	0.015	1.016	13.9960	14:0	1.23	ECL deviates -0.004	Reference -0.007	
2.0998	982	0.013	----	14.0601		----			
2.1279	1696	0.016	----	14.1238	14:0 iso 3OH	----	ECL deviates -0.001		
2.1531	3421	0.023	----	14.1807		----			
2.2195	2970	0.020	----	14.3309		----			
2.2659	45467	0.017	1.001	14.4359	15:1 iso w6c	1.26	ECL deviates -0.003		
2.2836	8323	0.011	----	14.4760		----			
2.3057	12210	0.014	0.998	14.5261	15:1 anteiso w9c	0.34	ECL deviates -0.004		
2.3449	212501	0.013	0.996	14.6146	15:0 iso	5.84	ECL deviates -0.002	Reference -0.006	
2.3861	162640	0.014	0.993	14.7078	15:0 anteiso	4.46	ECL deviates -0.003	Reference -0.007	
2.4512	8522	0.026	0.989	14.8551	15:1 w6c	0.23	ECL deviates -0.005		
2.5153	21445	0.015	0.985	15.0000	15:0	0.58	ECL deviates  0.000	Reference -0.004	
2.5433	9506	0.019	----	15.0541		----			
2.6060	2445	0.020	----	15.1736		----			
2.6371	3339	0.023	----	15.2329		----			
2.7216	7767	0.016	0.977	15.3941	16:1 w7c alcohol	0.21	ECL deviates -0.003		
2.7475	34528	0.022	0.976	15.4434	15:0 DMA	0.93	ECL deviates -0.007		
2.8080	88844	0.016	0.974	15.5587	16:0 N alcohol	2.39	ECL deviates  0.002		
2.8403	84046	0.015	0.973	15.6204	16:0 iso	2.26	ECL deviates  0.001	Reference -0.004	
2.9186	60176	0.020	0.971	15.7695	16:1 w9c	1.61	ECL deviates -0.005		
2.9474	388456	0.017	0.970	15.8244	16:1 w7c	10.39	ECL deviates  0.000		
2.9945	109967	0.016	0.969	15.9143	16:1 w5c	2.94	ECL deviates  0.003		
3.0439	388954	0.015	0.968	16.0080	16:0	10.38	ECL deviates  0.008	Reference  0.003	
3.0708	20338	0.019	----	16.0530		----			
3.1236	3213	0.016	0.966	16.1416	16:2 DMA	0.09	ECL deviates  0.004		
3.1588	6374	0.021	----	16.2006		----			
3.1946	3805	0.019	----	16.2606		----			
3.2343	2300	0.021	0.964	16.3271	16:1 w7c DMA	0.06	ECL deviates  0.017		
3.2936	220149	0.019	0.963	16.4263	16:0 10-methyl	5.85	ECL deviates  0.006		
3.3283	51172	0.018	0.963	16.4846	17:1 iso w9c	1.36	ECL deviates -0.013		
3.3563	24887	0.018	0.962	16.5314	17:1 anteiso w9c	0.66	ECL deviates -0.005		
3.4121	54097	0.017	0.962	16.6249	17:0 iso	1.44	ECL deviates  0.001	Reference -0.004	
3.4695	57024	0.018	0.961	16.7211	17:0 anteiso	1.51	ECL deviates  0.001		
3.5133	34524	0.018	0.961	16.7944	17:1 w8c	0.91	ECL deviates -0.003		
3.5735	126623	0.018	0.960	16.8954	17:0 cyclo w7c	3.35	ECL deviates  0.002		
3.6369	15573	0.017	0.960	17.0016	17:0	0.41	ECL deviates  0.002	Reference -0.004	
3.6636	23647	0.016	0.959	17.0427	17:1 w7c 10-methyl	0.63	ECL deviates -0.001		
3.7055	5819	0.018	----	17.1068		----			
3.7415	1825	0.020	----	17.1619		----			
3.7915	2718	0.018	0.959	17.2383	16:0 2OH	0.07	ECL deviates -0.002		
3.8459	564	0.014	----	17.3214		----			
3.9007	20792	0.016	0.959	17.4051	17:0 10-methyl	0.55	ECL deviates -0.002		
3.9381	2632	0.013	0.959	17.4623	17:0 DMA	0.07	ECL deviates  0.004		
3.9592	6135	0.023	----	17.4946		----			
4.0339	29534	0.029	0.959	17.6088	18:0 iso	0.78	ECL deviates -0.018		
4.1084	63091	0.018	0.959	17.7227	18:2 w6c	1.67	ECL deviates -0.004		
4.1424	238218	0.018	0.959	17.7746	18:1 w9c	6.30	ECL deviates  0.000		
4.1784	396787	0.017	0.959	17.8296	18:1 w7c	10.50	ECL deviates  0.003		
4.2354	44187	0.022	0.959	17.9168	18:1 w5c	1.17	ECL deviates -0.006		
4.2906	61317	0.017	0.959	18.0011	18:0	1.62	ECL deviates  0.001	Reference -0.005	
4.3474	22854	0.019	0.959	18.0836	18:1 w7c 10-methyl	0.60	ECL deviates -0.001		
4.4017	8383	0.029	0.959	18.1621	18:2 DMA	0.22	ECL deviates  0.002		
4.4480	6015	0.030	0.960	18.2290	18:1 w9c DMA	0.16	ECL deviates -0.008		
4.5074	1617	0.017	----	18.3149		----			
4.5603	95953	0.019	0.960	18.3915	18:0 10-methyl	2.54	ECL deviates -0.003		
4.6275	2750	0.020	0.960	18.4886	19:4 w6c	0.07	ECL deviates  0.004		
4.6750	9175	0.024	0.961	18.5573	19:3 w6c	0.24	ECL deviates -0.003		
4.7277	2280	0.015	0.961	18.6336	19:0 iso	0.06	ECL deviates  0.004		
4.7445	2151	0.015	0.961	18.6579	19:3 w3c	0.06	ECL deviates  0.000		
4.8062	12349	0.022	----	18.7471		----			
4.8519	10866	0.021	0.962	18.8132	19:1 w8c	0.29	ECL deviates  0.002		
4.8873	15863	0.016	0.962	18.8643	19:0 cyclo w9c	0.42	ECL deviates -0.008		
4.9144	89750	0.020	0.962	18.9035	19:0 cyclo w7c	2.38	ECL deviates -0.006		
4.9839	83433	0.018	----	19.0040	19:0	----	ECL deviates  0.004		
5.0450	1915	0.017	----	19.0892		----			
5.1400	2486	0.022	----	19.2218		----			
5.1719	8124	0.018	----	19.2663		----			
5.2596	24938	0.029	0.965	19.3885	20:4 w6c	0.66	ECL deviates -0.015		
5.3123	10573	0.020	0.966	19.4620	20:5 w3c	0.28	ECL deviates -0.020		
5.3471	2052	0.015	----	19.5105		----			
5.3777	5732	0.020	----	19.5530		----			
5.4106	9648	0.025	----	19.5989		----			
5.5290	22565	0.027	0.967	19.7641	20:1 w9c	0.60	ECL deviates -0.009		
5.5615	10137	0.022	0.967	19.8092	20:1 w8c	0.27	ECL deviates -0.004		
5.7002	19931	0.023	0.969	20.0026	20:0	0.53	ECL deviates  0.003	Reference -0.004	
5.8028	2730	0.016	----	20.1444		----			
5.8338	6151	0.021	----	20.1872		----			
5.9450	8906	0.023	----	20.3407		----			
5.9753	43839	0.024	0.971	20.3827	20:0 10-methyl	1.17	ECL deviates -0.014		
6.0502	1058	0.017	----	20.4860		----			
6.0799	1413	0.014	----	20.5271		----			
6.1007	2502	0.020	----	20.5558		----			
6.1482	9372	0.027	----	20.6215		----			
6.2111	3954	0.028	----	20.7084		----			
6.2769	10714	0.019	0.972	20.7992	21:1 w8c	0.29	ECL deviates  0.001		
6.3323	7800	0.024	----	20.8758		----			
6.3913	20320	0.019	0.973	20.9572	21:1 w3c	0.55	ECL deviates  0.003		
6.4278	6263	0.021	0.973	21.0076	21:0	0.17	ECL deviates  0.008	Reference  0.002	
6.5074	3305	0.022	----	21.1171		----			
6.5915	4870	0.029	0.974	21.2328	22:5 w6c	0.13	ECL deviates -0.019		
6.6255	5444	0.020	----	21.2796		----			
6.6490	1753	0.013	0.974	21.3119	22:6 w3c	0.05	ECL deviates -0.020		
6.6932	725	0.017	----	21.3727		----			
6.7493	1562	0.031	0.974	21.4498	22:5 w3c	0.04	ECL deviates -0.018		
6.8757	11857	0.031	0.974	21.6237	22:0 iso	0.32	ECL deviates  0.006		
6.9496	3447	0.028	0.974	21.7254	22:2 w6c	0.09	ECL deviates -0.013		
6.9838	2401	0.019	0.974	21.7723	22:1 w9c	0.06	ECL deviates -0.001		
7.0198	5468	0.027	0.974	21.8219	22:1 w8c	0.15	ECL deviates  0.008		
7.1029	5530	0.019	0.974	21.9362	22:1 w3c	0.15	ECL deviates -0.011		
7.1475	21374	0.019	0.974	21.9975	22:0	0.57	ECL deviates -0.003	Reference -0.008	
7.2110	1264	0.019	----	22.0862		----			
7.2398	801	0.018	----	22.1264		----			
7.3217	11347	0.019	----	22.2410		----			
7.5996	2837	0.037	----	22.6296		----	> max ar/ht		
7.7023	3095	0.020	----	22.7731		----			
7.7627	1277	0.020	----	22.8576		----			
7.8055	9556	0.019	0.969	22.9174	23:1 w4c	0.26	ECL deviates -0.009		
7.8639	4680	0.019	0.968	22.9990	23:0	0.13	ECL deviates -0.001	Reference -0.006	
7.9099	1424	0.022	----	23.0640		----			
8.0721	5749	0.018	----	23.2936		----			
8.3211	7301	0.023	----	23.6458		----			
8.3767	2184	0.022	----	23.7244		----			
8.4124	2679	0.022	----	23.7749		----			
8.4861	2965	0.030	----	23.8792		----			
8.5686	19735	0.021	0.954	23.9958	24:0	0.52	ECL deviates -0.004	Reference -0.008	
8.6716	825	0.018	----	24.1416		----	> max rt		
8.9244	11504	0.019	----	24.4991		----	> max rt		
9.2283	22267	0.021	----	24.9289		----	> max rt		
9.4672	10377	0.018	----	25.2666		----	> max rt		

ECL Deviation: 0.008                            Reference ECL Shift: 0.007       Number Reference Peaks: 21
Total Response: 3965404                       Total Named: 3735117
Percent Named: 94.19%                         Total Amount: 3624552

(No search libraries specified in method PLFAD1.)
