# Supplementary material for: Microbial regulation of soil carbon properties under nitrogen addition and plant inputs removal
Source: PeerJ. 2019 Jul 17;7:e7343. doi: 10.7717/peerj.7343 (PMC6642627; doi:10.7717/peerj.7343)
Supplement: File S1 — The raw data showed the soil microbial PLFAs files in the year of 2015 and 2016. Each file of rtf. represented the microbial PLFAs for each soil sample. In the Supplemental File, the Excel file named “Numbers” showed the plots names and the related rtf. file names. [file peerj-07-7343-s002.zip › supplementary files/2015/45.rtf]

Volume: DATA            File: E164216.88A        Samp Ctr: 3                  ID Number: 29342 
Type: Samp                   Bottle: 14                      Method: PLFAD1 
Created: 4/21/2016 4:55:09 PM 
Sample ID: 45 


RT	Response	Ar/Ht	RFact	ECL	Peak Name	Percent	Comment1	Comment2	
0.7141	1.905E+9	0.017	----	7.6515	SOLVENT PEAK	----	< min rt		
0.8849	722	0.009	----	8.7662		----	< min rt		
0.9442	1228	0.013	----	9.1617		----	< min rt		
0.9711	377	0.009	----	9.3369		----	< min rt		
1.1859	4508	0.012	----	10.7355		----			
1.2613	1206	0.015	----	11.1683		----			
1.3193	881	0.014	1.181	11.4448	10:0 3OH	0.02	ECL deviates  0.004		
1.3529	1446	0.018	1.166	11.6050	12:0 iso	0.03	ECL deviates -0.007		
1.3646	446	0.008	----	11.6610		----			
1.3897	1738	0.015	----	11.7804		----			
1.4361	4821	0.015	1.136	12.0017	12:0	0.11	ECL deviates  0.002	Reference -0.005	
1.4943	3081	0.018	----	12.2117		----			
1.5217	549	0.013	----	12.3100		----			
1.5590	1919	0.019	----	12.4439		----			
1.6045	5028	0.013	1.095	12.6069	13:0 iso	0.11	ECL deviates -0.005	Reference -0.011	
1.6339	3927	0.016	1.089	12.7123	13:0 anteiso	0.09	ECL deviates  0.003	Reference -0.002	
1.6900	1347	0.017	1.077	12.9135	13:1 w5c	0.03	ECL deviates -0.006		
1.7143	2091	0.013	1.073	13.0008	13:0	0.04	ECL deviates  0.001	Reference -0.004	
1.7811	903	0.018	----	13.1879	12:0 2OH	----	ECL deviates  0.002		
1.8255	443	0.010	----	13.3117		----			
1.8731	2540	0.018	----	13.4443		----			
1.9327	67358	0.013	1.043	13.6103	14:0 iso	1.40	ECL deviates -0.004	Reference -0.007	
1.9717	1390	0.013	1.039	13.7191	14:0 anteiso	0.03	ECL deviates  0.003	Reference  0.000	
1.9930	1636	0.010	1.036	13.7785	14:1 w9c	0.03	ECL deviates  0.001		
2.0071	2564	0.012	----	13.8176		----			
2.0409	4329	0.014	1.031	13.9119	14:1 w5c	0.09	ECL deviates  0.001		
2.0725	60357	0.014	1.028	14.0001	14:0	1.24	ECL deviates  0.000	Reference -0.003	
2.0996	831	0.012	----	14.0617		----			
2.1285	1575	0.015	----	14.1268	14:0 iso 3OH	----	ECL deviates  0.002		
2.1528	3688	0.025	----	14.1817		----			
2.2163	2815	0.021	----	14.3251		----			
2.2668	87331	0.020	1.013	14.4390	15:1 iso w6c	1.76	ECL deviates  0.000		
2.3064	20523	0.015	1.010	14.5284	15:1 anteiso w9c	0.41	ECL deviates -0.002		
2.3458	303873	0.014	1.008	14.6172	15:0 iso	6.10	ECL deviates  0.000	Reference -0.002	
2.3872	228059	0.014	1.005	14.7106	15:0 anteiso	4.57	ECL deviates  0.000	Reference -0.002	
2.4516	12185	0.026	1.001	14.8561	15:1 w6c	0.24	ECL deviates -0.004		
2.5161	30403	0.015	0.998	15.0016	15:0	0.60	ECL deviates  0.002	Reference  0.000	
2.5445	10251	0.018	----	15.0554		----			
2.6068	2869	0.020	----	15.1738		----			
2.6359	5517	0.022	----	15.2289		----			
2.7224	8269	0.014	0.990	15.3930	16:1 w7c alcohol	0.16	ECL deviates -0.004		
2.7477	52876	0.020	0.989	15.4410	15:0 DMA	1.04	ECL deviates -0.010		
2.8087	82247	0.016	0.987	15.5567	16:0 N alcohol	1.62	ECL deviates  0.000		
2.8422	127743	0.016	0.986	15.6203	16:0 iso	2.51	ECL deviates  0.001	Reference  0.000	
2.9205	84248	0.020	0.983	15.7689	16:1 w9c	1.65	ECL deviates -0.006		
2.9509	558724	0.017	0.983	15.8267	16:1 w7c	10.94	Column Overload		
2.9977	197235	0.017	0.981	15.9154	16:1 w5c	3.86	ECL deviates  0.004		
3.0473	574088	0.015	0.980	16.0084	16:0	11.22	Column Overload		
3.0732	24396	0.019	----	16.0517		----			
3.1264	4362	0.016	0.979	16.1408	16:2 DMA	0.09	ECL deviates  0.003		
3.1604	10061	0.022	----	16.1975		----			
3.1977	4747	0.018	----	16.2600		----			
3.2338	2988	0.021	0.977	16.3204	16:1 w7c DMA	0.06	ECL deviates  0.010		
3.2955	295087	0.020	0.976	16.4236	16:0 10-methyl	5.74	ECL deviates  0.004		
3.3310	56447	0.019	0.975	16.4830	17:1 iso w9c	1.10	ECL deviates -0.015		
3.3590	35823	0.018	0.975	16.5298	17:1 anteiso w9c	0.70	ECL deviates -0.006		
3.4151	73270	0.016	0.974	16.6235	17:0 iso	1.42	ECL deviates  0.000	Reference  0.000	
3.4721	85750	0.018	0.973	16.7189	17:0 anteiso	1.66	ECL deviates -0.001		
3.5161	58375	0.018	0.973	16.7925	17:1 w8c	1.13	ECL deviates -0.005		
3.5763	175195	0.018	0.972	16.8932	17:0 cyclo w7c	3.40	ECL deviates  0.000		
3.6399	24795	0.018	0.972	16.9996	17:0	0.48	ECL deviates  0.000	Reference  0.000	
3.6661	27363	0.017	0.971	17.0395	17:1 w7c 10-methyl	0.53	ECL deviates -0.004		
3.7093	8042	0.017	----	17.1053		----			
3.7437	2595	0.020	----	17.1578		----			
3.7933	4020	0.017	0.971	17.2333	16:0 2OH	0.08	ECL deviates -0.007		
3.8486	781	0.013	----	17.3177		----			
3.9049	33882	0.019	0.970	17.4036	17:0 10-methyl	0.66	ECL deviates -0.003		
3.9407	2664	0.011	0.970	17.4581	17:0 DMA	0.05	ECL deviates  0.000		
3.9636	10621	0.025	----	17.4931		----			
4.0373	44409	0.031	----	17.6053		----			
4.1129	126411	0.018	0.970	17.7205	18:2 w6c	2.44	ECL deviates -0.007		
4.1468	340253	0.020	0.970	17.7723	18:1 w9c	6.58	ECL deviates -0.002		
4.1845	552116	0.017	0.969	17.8298	18:1 w7c	10.67	Column Overload		
4.2371	87396	0.022	----	17.9099		----			
4.2954	90023	0.018	0.969	17.9988	18:0	1.74	ECL deviates -0.001	Reference  0.000	
4.3514	34423	0.018	0.969	18.0796	18:1 w7c 10-methyl	0.67	ECL deviates -0.005		
4.4039	10047	0.027	0.969	18.1555	18:2 DMA	0.19	ECL deviates -0.004		
4.4508	6500	0.023	0.969	18.2232	18:1 w9c DMA	0.13	ECL deviates -0.014		
4.4837	2329	0.016	0.970	18.2707	18:1 w7c DMA	0.05	ECL deviates -0.012		
4.5109	1748	0.016	----	18.3101		----			
4.5645	120193	0.020	0.970	18.3875	18:0 10-methyl	2.32	ECL deviates -0.007		
4.6308	3894	0.021	0.970	18.4833	19:4 w6c	0.08	ECL deviates -0.002		
4.6758	9398	0.026	0.970	18.5482	19:3 w6c	0.18	ECL deviates -0.012		
4.7313	2319	0.012	0.970	18.6284	19:0 iso	0.04	ECL deviates -0.002		
4.7480	4010	0.018	0.970	18.6525	19:3 w3c	0.08	ECL deviates -0.006		
4.8121	16241	0.021	----	18.7451		----			
4.8529	15167	0.018	0.970	18.8041	19:1 w8c	0.29	ECL deviates -0.007		
4.9195	136065	0.023	0.970	18.9002	19:0 cyclo w7c	2.63	ECL deviates -0.010		
4.9891	80975	0.017	----	19.0007	19:0	----	ECL deviates  0.001		
5.0491	2757	0.016	----	19.0843		----			
5.1406	2341	0.018	----	19.2118		----			
5.1774	12643	0.019	----	19.2631		----			
5.2606	41124	0.028	----	19.3790		----			
5.3166	14004	0.019	----	19.4571		----			
5.3521	2849	0.016	0.971	19.5065	20:5 w3c	0.06	ECL deviates  0.024		
5.3818	8010	0.019	----	19.5479		----			
5.4152	12766	0.025	----	19.5945		----			
5.5345	32392	0.026	0.972	19.7607	20:1 w9c	0.63	ECL deviates -0.012		
5.5664	13176	0.025	0.972	19.8052	20:1 w8c	0.26	ECL deviates -0.008		
5.7041	30708	0.025	0.972	19.9972	20:0	0.59	ECL deviates -0.003	Reference -0.001	
5.7578	1635	0.017	----	20.0714		----			
5.8070	3616	0.018	----	20.1396		----			
5.8356	8742	0.021	----	20.1792		----			
5.9476	11449	0.027	----	20.3343		----			
5.9784	34811	0.023	----	20.3769		----			
6.0539	1268	0.017	----	20.4814		----			
6.1043	5014	0.027	----	20.5513		----			
6.1538	5089	0.020	----	20.6197		----			
6.1765	3746	0.017	0.971	20.6512	21:3 w3c	0.07	ECL deviates -0.002		
6.2214	4988	0.031	----	20.7133		----			
6.2797	12112	0.021	0.971	20.7942	21:1 w8c	0.23	ECL deviates -0.004		
6.3364	11576	0.022	----	20.8726		----			
6.3946	22843	0.021	0.970	20.9532	21:1 w3c	0.44	ECL deviates -0.001		
6.4303	6604	0.020	0.970	21.0027	21:0	0.13	ECL deviates  0.003	Reference  0.004	
6.5112	3089	0.020	----	21.1143		----			
6.5974	7847	0.027	0.969	21.2333	22:5 w6c	0.15	ECL deviates -0.019		
6.6282	10204	0.021	----	21.2758		----			
6.6549	3087	0.013	0.969	21.3126	22:6 w3c	0.06	ECL deviates -0.019		
6.6921	1756	0.019	----	21.3640		----			
6.7576	1663	0.023	0.968	21.4544	22:5 w3c	0.03	ECL deviates -0.013		
6.8796	12702	0.029	0.967	21.6228	22:0 iso	0.24	ECL deviates  0.005		
6.9572	2488	0.022	0.966	21.7300	22:2 w6c	0.05	ECL deviates -0.009		
6.9943	3476	0.022	0.965	21.7812	22:1 w9c	0.07	ECL deviates  0.008		
7.0230	4390	0.026	0.965	21.8209	22:1 w8c	0.08	ECL deviates  0.007		
7.1093	6147	0.020	0.964	21.9400	22:1 w3c	0.12	ECL deviates -0.007		
7.1535	33694	0.021	0.963	22.0010	22:0	0.65	ECL deviates  0.001	Reference  0.002	
7.2093	2243	0.024	----	22.0790		----			
7.2491	1482	0.025	----	22.1348		----			
7.3268	9775	0.018	----	22.2434		----			
7.5386	656	0.016	----	22.5399		----			
7.6117	1981	0.026	0.954	22.6422	23:3 w3c	0.04	ECL deviates -0.002		
7.6471	860	0.021	----	22.6918		----			
7.7067	5099	0.024	----	22.7751		----			
7.7660	3009	0.023	----	22.8581		----			
7.8112	10909	0.021	0.949	22.9215	23:1 w4c	0.21	ECL deviates -0.005		
7.8684	7446	0.018	0.947	23.0014	23:0	0.14	ECL deviates  0.001	Reference  0.001	
7.9173	2088	0.026	----	23.0707		----			
8.0758	7635	0.020	----	23.2951		----			
8.2860	859	0.016	0.933	23.5927	24:3 w6c	0.02	ECL deviates  0.002		
8.3245	6363	0.022	0.931	23.6472	24:3 w3c	0.12	ECL deviates -0.007		
8.3833	1832	0.020	----	23.7306		----			
8.4158	3007	0.020	0.927	23.7766	24:1 w9c	0.06	ECL deviates -0.010		
8.4904	1722	0.024	----	23.8823		----			
8.5255	652	0.014	----	23.9320		----			
8.5722	26149	0.020	0.920	23.9981	24:0	0.48	ECL deviates -0.002	Reference -0.004	
8.6742	829	0.018	----	24.1425		----	> max rt		
8.9285	11593	0.018	----	24.5028		----	> max rt		
9.1578	1049	0.019	----	24.8276		----	> max rt		
9.2296	17794	0.022	----	24.9292		----	> max rt		
9.2585	2607	0.012	----	24.9703		----	> max rt		
9.4676	10732	0.022	----	25.2665		----	> max rt		

ECL Deviation: 0.007                            Reference ECL Shift: 0.004       Number Reference Peaks: 19
Total Response: 5602157                       Total Named: 5112222
Percent Named: 91.25%                         Total Amount: 5016924
Profile Comment:   Column Overload:  A peak's response is greater than 400000.0.  Dilute and re-run.

(No search libraries specified in method PLFAD1.)
