# Supplementary material for: Microbial regulation of soil carbon properties under nitrogen addition and plant inputs removal
Source: PeerJ. 2019 Jul 17;7:e7343. doi: 10.7717/peerj.7343 (PMC6642627; doi:10.7717/peerj.7343)
Supplement: File S1 — The raw data showed the soil microbial PLFAs files in the year of 2015 and 2016. Each file of rtf. represented the microbial PLFAs for each soil sample. In the Supplemental File, the Excel file named “Numbers” showed the plots names and the related rtf. file names. [file peerj-07-7343-s002.zip › supplementary files/2015/46.rtf]

Volume: DATA            File: E164216.88A        Samp Ctr: 4                  ID Number: 29343 
Type: Samp                   Bottle: 15                      Method: PLFAD1 
Created: 4/21/2016 5:07:44 PM 
Sample ID: 46 


RT	Response	Ar/Ht	RFact	ECL	Peak Name	Percent	Comment1	Comment2	
0.7140	1.9E+9	0.015	----	7.6434	SOLVENT PEAK	----	< min rt		
0.8852	874	0.010	----	8.7613		----	< min rt		
0.9447	569	0.011	----	9.1514		----	< min rt		
1.1865	3581	0.012	----	10.7294		----			
1.2620	592	0.012	----	11.1626		----			
1.3663	369	0.010	----	11.6613		----			
1.3896	1108	0.015	----	11.7727		----			
1.4367	2711	0.015	1.136	11.9981	12:0	0.08	ECL deviates -0.002	Reference -0.002	
1.4948	2079	0.017	----	12.2070		----			
1.5599	1411	0.017	----	12.4410		----			
1.6052	3759	0.013	1.095	12.6040	13:0 iso	0.11	ECL deviates -0.008	Reference -0.008	
1.6382	3542	0.015	1.089	12.7225	13:0 anteiso	0.10	ECL deviates  0.013	Reference  0.013	
1.6899	1041	0.015	----	12.9085		----			
1.7148	1308	0.013	1.073	12.9978	13:0	0.04	ECL deviates -0.002	Reference -0.002	
1.7806	847	0.016	----	13.1819	12:0 2OH	----	ECL deviates -0.004		
1.8731	1947	0.020	----	13.4403		----			
1.9333	53870	0.013	1.043	13.6082	14:0 iso	1.46	ECL deviates -0.006	Reference -0.006	
1.9746	1118	0.014	1.039	13.7236	14:0 anteiso	0.03	ECL deviates  0.008	Reference  0.008	
1.9933	1128	0.009	1.036	13.7760	14:1 w9c	0.03	ECL deviates -0.002		
2.0084	2063	0.014	----	13.8179		----			
2.0422	2844	0.011	1.031	13.9123	14:1 w5c	0.08	ECL deviates  0.001		
2.0733	42315	0.014	1.028	13.9993	14:0	1.13	ECL deviates -0.001	Reference  0.000	
2.1009	936	0.012	----	14.0617		----			
2.1294	1321	0.015	----	14.1261	14:0 iso 3OH	----	ECL deviates  0.001		
2.1528	2572	0.021	----	14.1790		----			
2.2196	2346	0.019	----	14.3299		----			
2.2671	46943	0.018	1.013	14.4372	15:1 iso w6c	1.24	ECL deviates -0.002		
2.2860	11668	0.013	1.011	14.4800	15:4 w3c	0.31	ECL deviates -0.010		
2.3073	11297	0.014	1.010	14.5283	15:1 anteiso w9c	0.30	ECL deviates -0.002		
2.3464	249945	0.015	1.008	14.6167	15:0 iso	6.56	ECL deviates  0.000	Reference  0.000	
2.3879	190250	0.014	1.005	14.7104	15:0 anteiso	4.98	ECL deviates -0.001	Reference  0.000	
2.4528	8354	0.023	1.001	14.8570	15:1 w6c	0.22	ECL deviates -0.003		
2.5165	21808	0.015	0.998	15.0008	15:0	0.57	ECL deviates  0.001	Reference  0.001	
2.5446	12750	0.017	----	15.0541		----			
2.5867	928	0.011	----	15.1341		----			
2.6068	2633	0.015	----	15.1723		----			
2.6364	2782	0.020	----	15.2287		----			
2.7226	7668	0.014	0.990	15.3924	16:1 w7c alcohol	0.20	ECL deviates -0.004		
2.7485	41034	0.021	0.989	15.4416	15:0 DMA	1.06	ECL deviates -0.009		
2.8087	89803	0.017	0.987	15.5559	16:0 N alcohol	2.31	ECL deviates -0.001		
2.8416	94564	0.016	0.986	15.6185	16:0 iso	2.43	ECL deviates -0.001	Reference -0.001	
2.8930	9508	0.014	0.984	15.7162	16:0 anteiso	0.24	ECL deviates  0.001	Reference  0.001	
2.9207	57897	0.015	0.983	15.7687	16:1 w9c	1.48	ECL deviates -0.006		
2.9495	471724	0.016	0.983	15.8235	16:1 w7c	12.06	Column Overload		
2.9966	131195	0.015	0.981	15.9130	16:1 w5c	3.35	ECL deviates  0.002		
3.0455	406141	0.016	0.980	16.0052	16:0	10.36	Column Overload		
3.0733	29548	0.020	----	16.0517		----			
3.1257	2589	0.017	0.979	16.1396	16:2 DMA	0.07	ECL deviates  0.002		
3.1614	6234	0.020	----	16.1992		----			
3.1970	5387	0.020	----	16.2588		----			
3.2322	2392	0.021	0.977	16.3177	16:1 w7c DMA	0.06	ECL deviates  0.008		
3.2952	289361	0.019	0.976	16.4232	16:0 10-methyl	7.35	ECL deviates  0.003		
3.3297	56324	0.019	----	16.4811		----			
3.3576	26211	0.018	----	16.5278		----			
3.3820	1939	0.009	0.974	16.5686	17:1 anteiso w7c	0.05	ECL deviates -0.002		
3.4137	61580	0.016	0.974	16.6216	17:0 iso	1.56	ECL deviates -0.002	Reference -0.002	
3.4719	67183	0.017	0.973	16.7191	17:0 anteiso	1.70	ECL deviates -0.001		
3.5156	38056	0.018	0.973	16.7922	17:1 w8c	0.96	ECL deviates -0.005		
3.5752	141762	0.017	0.972	16.8921	17:0 cyclo w7c	3.59	ECL deviates -0.002		
3.6395	16214	0.018	0.972	16.9998	17:0	0.41	ECL deviates  0.000	Reference  0.000	
3.6653	33085	0.017	0.971	17.0391	17:1 w7c 10-methyl	0.84	ECL deviates -0.004		
3.7074	8189	0.015	----	17.1033		----			
3.7437	1881	0.020	----	17.1587		----			
3.7918	2195	0.017	0.971	17.2321	16:0 2OH	0.06	ECL deviates -0.008		
3.8478	661	0.015	----	17.3175		----			
3.9040	22965	0.018	0.970	17.4033	17:0 10-methyl	0.58	ECL deviates -0.004		
3.9394	2374	0.013	0.970	17.4572	17:0 DMA	0.06	ECL deviates -0.001		
3.9615	6499	0.021	----	17.4910		----			
4.0356	28767	0.029	----	17.6040		----			
4.1113	61986	0.018	0.970	17.7195	18:2 w6c	1.56	ECL deviates -0.008		
4.1441	251505	0.017	0.970	17.7695	18:1 w9c	6.35	ECL deviates -0.005		
4.1815	382858	0.018	0.969	17.8267	18:1 w7c	9.66	ECL deviates  0.000		
4.2385	53803	0.022	0.969	17.9136	18:1 w5c	1.36	ECL deviates -0.009		
4.2954	63440	0.018	0.969	18.0004	18:0	1.60	ECL deviates  0.000	Reference  0.000	
4.3503	19778	0.020	0.969	18.0798	18:1 w7c 10-methyl	0.50	ECL deviates -0.005		
4.4053	4663	0.016	0.969	18.1592	18:2 DMA	0.12	ECL deviates -0.001		
4.4209	4222	0.015	----	18.1817		----			
4.4507	4799	0.022	0.969	18.2248	18:1 w9c DMA	0.12	ECL deviates -0.012		
4.4840	1615	0.016	0.970	18.2729	18:1 w7c DMA	0.04	ECL deviates -0.010		
4.5121	1641	0.016	----	18.3135		----			
4.5638	98490	0.022	0.970	18.3883	18:0 10-methyl	2.49	ECL deviates -0.007		
4.6332	3172	0.019	0.970	18.4886	19:4 w6c	0.08	ECL deviates  0.004		
4.6765	8621	0.023	0.970	18.5511	19:3 w6c	0.22	ECL deviates -0.009		
4.7305	4339	0.028	0.970	18.6291	19:0 iso	0.11	ECL deviates -0.001		
4.8079	13452	0.021	----	18.7410		----			
4.8543	12550	0.020	0.970	18.8080	19:1 w8c	0.32	ECL deviates -0.003		
4.9167	112234	0.021	0.970	18.8982	19:0 cyclo w7c	2.83	ECL deviates -0.012		
4.9867	79828	0.018	----	18.9994	19:0	----	ECL deviates -0.001		
5.0479	1713	0.017	----	19.0847		----			
5.1408	1952	0.024	----	19.2142		----			
5.1742	5898	0.018	----	19.2608		----			
5.2129	2157	0.015	0.971	19.3147	19:0 cyclo 9,10 DMA	0.05	ECL deviates -0.009		
5.2614	20054	0.025	----	19.3823		----			
5.3141	9602	0.019	----	19.4558		----			
5.3486	1545	0.014	0.971	19.5039	20:5 w3c	0.04	ECL deviates  0.022		
5.3816	5236	0.020	----	19.5499		----			
5.4142	7657	0.023	----	19.5953		----			
5.5323	26604	0.027	0.972	19.7600	20:1 w9c	0.67	ECL deviates -0.013		
5.5637	11538	0.022	0.972	19.8038	20:1 w8c	0.29	ECL deviates -0.009		
5.7026	19234	0.020	0.972	19.9974	20:0	0.49	ECL deviates -0.003	Reference -0.003	
5.8051	2515	0.018	----	20.1393		----			
5.8354	6101	0.020	----	20.1812		----			
5.9769	44976	0.027	----	20.3773		----			
6.1034	766	0.014	----	20.5524		----			
6.1505	4802	0.020	----	20.6176		----			
6.1731	2578	0.016	0.971	20.6488	21:3 w3c	0.07	ECL deviates -0.005		
6.2136	3133	0.024	----	20.7050		----			
6.2793	10872	0.021	0.971	20.7959	21:1 w8c	0.27	ECL deviates -0.002		
6.3364	4292	0.022	----	20.8750		----			
6.3933	25533	0.018	0.970	20.9538	21:1 w3c	0.64	ECL deviates  0.000		
6.4292	3459	0.016	0.970	21.0035	21:0	0.09	ECL deviates  0.004	Reference  0.002	
6.5087	2549	0.016	----	21.1132		----			
6.5949	2111	0.019	0.969	21.2321	22:5 w6c	0.05	ECL deviates -0.020		
6.6282	3379	0.017	----	21.2782		----			
6.6518	1002	0.012	0.969	21.3108	22:6 w3c	0.03	ECL deviates -0.021		
6.7577	1302	0.026	0.968	21.4569	22:5 w3c	0.03	ECL deviates -0.011		
6.8785	9848	0.030	0.967	21.6235	22:0 iso	0.25	ECL deviates  0.006		
6.9539	3164	0.025	0.966	21.7276	22:2 w6c	0.08	ECL deviates -0.011		
6.9880	2562	0.022	0.965	21.7747	22:1 w9c	0.06	ECL deviates  0.002		
7.0224	3021	0.022	0.965	21.8221	22:1 w8c	0.08	ECL deviates  0.009		
7.1089	6785	0.021	0.964	21.9415	22:1 w3c	0.17	ECL deviates -0.005		
7.1508	18773	0.017	0.963	21.9994	22:0	0.47	ECL deviates -0.001	Reference -0.002	
7.2134	2517	0.023	----	22.0869		----			
7.3246	12522	0.021	----	22.2424		----			
7.3788	1281	0.020	----	22.3182		----			
7.4411	1083	0.023	----	22.4055		----			
7.4974	834	0.017	0.957	22.4842	23:4 w6c	0.02	ECL deviates  0.013		
7.6062	1331	0.022	0.954	22.6363	23:3 w3c	0.03	ECL deviates -0.008		
7.7061	2434	0.020	----	22.7761		----			
7.8093	9328	0.019	0.949	22.9204	23:1 w4c	0.23	ECL deviates -0.006		
7.8673	4059	0.019	0.947	23.0016	23:0	0.10	ECL deviates  0.002	Reference -0.001	
7.9158	1753	0.024	----	23.0702		----			
8.0731	4696	0.019	----	23.2929		----			
8.3225	5988	0.023	----	23.6457		----			
8.3815	1128	0.019	----	23.7291		----			
8.4132	2168	0.019	----	23.7740		----			
8.4857	1071	0.021	----	23.8767		----			
8.5718	18385	0.018	0.920	23.9984	24:0	0.44	ECL deviates -0.002	Reference -0.004	
8.7178	10402	0.028	----	24.2051		----	> max rt		
8.8485	7341	0.034	----	24.3900		----	> max rt		
8.9270	17069	0.023	----	24.5011		----	> max rt		
9.0238	2400	0.037	----	24.6381		----	> max rt		
9.1584	937	0.021	----	24.8287		----	> max rt		
9.2275	15162	0.022	----	24.9263		----	> max rt		
9.2589	1615	0.012	----	24.9709		----	> max rt		
9.4645	5132	0.019	----	25.2618		----	> max rt		

ECL Deviation: 0.007                            Reference ECL Shift: 0.004       Number Reference Peaks: 20
Total Response: 4303386                       Total Named: 3913966
Percent Named: 90.95%                         Total Amount: 3841869
Profile Comment:   Column Overload:  A peak's response is greater than 400000.0.  Dilute and re-run.

(No search libraries specified in method PLFAD1.)
