# Supplementary material for: Microbial regulation of soil carbon properties under nitrogen addition and plant inputs removal
Source: PeerJ. 2019 Jul 17;7:e7343. doi: 10.7717/peerj.7343 (PMC6642627; doi:10.7717/peerj.7343)
Supplement: File S1 — The raw data showed the soil microbial PLFAs files in the year of 2015 and 2016. Each file of rtf. represented the microbial PLFAs for each soil sample. In the Supplemental File, the Excel file named “Numbers” showed the plots names and the related rtf. file names. [file peerj-07-7343-s002.zip › supplementary files/2015/47.rtf]

Volume: DATA            File: E164216.88A        Samp Ctr: 5                  ID Number: 29344 
Type: Samp                   Bottle: 16                      Method: PLFAD1 
Created: 4/21/2016 5:27:17 PM 
Sample ID: 47 


RT	Response	Ar/Ht	RFact	ECL	Peak Name	Percent	Comment1	Comment2	
0.7142	1.904E+9	0.016	----	7.6346	SOLVENT PEAK	----	< min rt		
0.8849	2194	0.015	----	8.7530		----	< min rt		
1.0448	639	0.011	----	9.8003		----	< min rt		
1.1858	2056	0.012	1.250	10.7233	11:0 anteiso	0.06	ECL deviates  0.018	Reference  0.020	
1.2245	709	0.015	----	10.9768		----			
1.2613	650	0.011	----	11.1593		----			
1.2769	421	0.009	----	11.2340		----			
1.3117	695	0.015	----	11.4009		----			
1.3519	1448	0.014	----	11.5936	Phthalate 1	----	ECL deviates  0.006		
1.3640	522	0.008	----	11.6514		----			
1.3894	2726	0.014	----	11.7729		----			
1.4358	6068	0.015	1.136	11.9954	12:0	0.17	ECL deviates -0.005	Reference -0.006	
1.4930	3209	0.014	----	12.2031		----			
1.5195	510	0.012	----	12.2984		----			
1.5586	1646	0.017	----	12.4391		----			
1.6042	5635	0.012	1.095	12.6035	13:0 iso	0.15	ECL deviates -0.009	Reference -0.012	
1.6311	2982	0.014	1.089	12.7003	13:0 anteiso	0.08	ECL deviates -0.009	Reference -0.012	
1.6885	499	0.012	----	12.9070		----			
1.7132	2094	0.013	1.073	12.9960	13:0	0.06	ECL deviates -0.004	Reference -0.008	
1.7794	909	0.017	----	13.1828	12:0 2OH	----	ECL deviates -0.003		
1.8721	2004	0.018	----	13.4421		----			
1.9315	44442	0.014	1.043	13.6082	14:0 iso	1.16	ECL deviates -0.006	Reference -0.011	
1.9718	1725	0.015	1.039	13.7210	14:0 anteiso	0.04	ECL deviates  0.005	Reference  0.000	
1.9913	1300	0.010	1.036	13.7755	14:1 w9c	0.03	ECL deviates -0.002		
2.0058	2043	0.013	----	13.8160		----			
2.0711	50348	0.014	1.028	13.9986	14:0	1.30	ECL deviates -0.001	Reference -0.007	
2.0986	887	0.012	----	14.0623		----			
2.1264	1285	0.014	----	14.1252	14:0 iso 3OH	----	ECL deviates  0.000		
2.1524	3578	0.025	----	14.1840		----			
2.2188	3184	0.021	----	14.3343		----			
2.2644	50957	0.018	1.013	14.4374	15:1 iso w6c	1.29	ECL deviates -0.002		
2.2826	9503	0.012	1.011	14.4786	15:4 w3c	0.24	ECL deviates -0.012		
2.3047	12003	0.014	1.010	14.5287	15:1 anteiso w9c	0.30	ECL deviates -0.001		
2.3435	235511	0.014	1.008	14.6166	15:0 iso	5.95	ECL deviates  0.000	Reference -0.007	
2.3850	170953	0.014	1.005	14.7104	15:0 anteiso	4.31	ECL deviates -0.001	Reference -0.007	
2.4500	9320	0.026	1.001	14.8574	15:1 w6c	0.23	ECL deviates -0.003		
2.5135	25611	0.015	0.998	15.0012	15:0	0.64	ECL deviates  0.001	Reference -0.005	
2.5422	8999	0.017	----	15.0565		----			
2.6038	2435	0.020	----	15.1737		----			
2.6353	3342	0.020	----	15.2337		----			
2.7206	6858	0.015	0.990	15.3959	16:1 w7c alcohol	0.17	ECL deviates -0.001		
2.7471	39007	0.022	0.989	15.4463	15:0 DMA	0.97	ECL deviates -0.004		
2.8061	85964	0.016	0.987	15.5585	16:0 N alcohol	2.13	ECL deviates  0.002		
2.8387	93363	0.015	0.986	15.6205	16:0 iso	2.31	ECL deviates  0.001	Reference -0.007	
2.9183	65781	0.020	0.983	15.7718	16:1 w9c	1.62	ECL deviates -0.003		
2.9469	395156	0.018	0.983	15.8262	16:1 w7c	9.73	ECL deviates  0.002		
2.9936	122669	0.016	0.981	15.9149	16:1 w5c	3.02	ECL deviates  0.004		
3.0431	414972	0.015	0.980	16.0088	16:0	10.19	Column Overload		
3.0696	21021	0.019	----	16.0532		----			
3.1224	3140	0.016	0.979	16.1417	16:2 DMA	0.08	ECL deviates  0.004		
3.1574	7306	0.024	----	16.2004		----			
3.1939	3767	0.019	----	16.2615		----			
3.2333	2409	0.022	0.977	16.3275	16:1 w7c DMA	0.06	ECL deviates  0.017		
3.2922	238309	0.021	0.976	16.4262	16:0 10-methyl	5.83	ECL deviates  0.006		
3.3270	49301	0.018	0.975	16.4845	17:1 iso w9c	1.20	ECL deviates -0.014		
3.3552	27837	0.019	0.975	16.5316	17:1 anteiso w9c	0.68	ECL deviates -0.004		
3.4105	56434	0.016	0.974	16.6243	17:0 iso	1.38	ECL deviates  0.001	Reference -0.007	
3.4676	64532	0.018	0.973	16.7200	17:0 anteiso	1.57	ECL deviates  0.000		
3.5113	43836	0.017	0.973	16.7932	17:1 w8c	1.07	ECL deviates -0.004		
3.5715	146333	0.018	0.972	16.8940	17:0 cyclo w7c	3.56	ECL deviates  0.000		
3.6368	19358	0.018	0.972	17.0033	17:0	0.47	ECL deviates  0.003	Reference -0.005	
3.6620	24740	0.017	0.971	17.0422	17:1 w7c 10-methyl	0.60	ECL deviates -0.001		
3.7054	6437	0.017	----	17.1084		----			
3.7398	2086	0.021	----	17.1609		----			
3.7894	3388	0.018	0.971	17.2365	16:0 2OH	0.08	ECL deviates -0.004		
3.8419	619	0.014	----	17.3167		----			
3.9007	26170	0.018	0.970	17.4064	17:0 10-methyl	0.64	ECL deviates -0.001		
3.9383	2890	0.013	0.970	17.4637	17:0 DMA	0.07	ECL deviates  0.006		
3.9574	7067	0.022	----	17.4928		----			
4.0147	8918	0.013	0.970	17.5802	18:3 w6c	0.22	ECL deviates  0.000		
4.0318	25019	0.022	----	17.6063		----			
4.1084	110099	0.019	0.970	17.7232	18:2 w6c	2.67	ECL deviates -0.004		
4.1426	275104	0.019	0.970	17.7753	18:1 w9c	6.68	ECL deviates  0.001		
4.1788	434146	0.015	0.969	17.8305	18:1 w7c	10.55	Column Overload		
4.2362	50296	0.022	0.969	17.9181	18:1 w5c	1.22	ECL deviates -0.005		
4.2926	71781	0.018	0.969	18.0041	18:0	1.74	ECL deviates  0.004	Reference -0.004	
4.3475	31319	0.018	0.969	18.0836	18:1 w7c 10-methyl	0.76	ECL deviates -0.001		
4.4006	6108	0.018	0.969	18.1603	18:2 DMA	0.15	ECL deviates  0.000		
4.4161	3419	0.012	----	18.1828		----			
4.4505	7179	0.029	0.969	18.2323	18:1 w9c DMA	0.17	ECL deviates -0.005		
4.5102	1624	0.018	----	18.3187		----			
4.5613	111346	0.020	0.970	18.3925	18:0 10-methyl	2.71	ECL deviates -0.003		
4.6311	3212	0.021	0.970	18.4933	19:4 w6c	0.08	ECL deviates  0.008		
4.6743	10460	0.024	0.970	18.5556	19:3 w6c	0.25	ECL deviates -0.004		
4.7436	5836	0.025	0.970	18.6557	19:3 w3c	0.14	ECL deviates -0.003		
4.8076	15723	0.021	----	18.7482		----			
4.8508	15101	0.021	0.970	18.8106	19:1 w8c	0.37	ECL deviates  0.000		
4.9158	143433	0.022	0.970	18.9044	19:0 cyclo w7c	3.49	ECL deviates -0.005		
4.9848	84541	0.019	----	19.0041	19:0	----	ECL deviates  0.004		
5.0452	3109	0.022	----	19.0883		----			
5.1399	3355	0.022	----	19.2202		----			
5.1730	8406	0.019	----	19.2664		----			
5.2104	1910	0.012	0.971	19.3185	19:0 cyclo 9,10 DMA	----	Below has same name		
5.2160	801	0.005	----	19.3263	19:0 cyclo 9,10 DMA	----	Above has same name		
5.2583	28014	0.024	----	19.3852		----			
5.3125	13517	0.019	0.971	19.4607	20:5 w3c	0.33	ECL deviates -0.021		
5.3476	2024	0.015	----	19.5095		----			
5.3780	6911	0.021	----	19.5519		----			
5.4105	10756	0.023	----	19.5972		----			
5.5305	27299	0.028	0.972	19.7642	20:1 w9c	0.66	ECL deviates -0.008		
5.5588	11212	0.021	0.972	19.8036	20:1 w8c	0.27	ECL deviates -0.009		
5.6152	1013	0.018	----	19.8822		----			
5.6476	669	0.015	0.972	19.9273	20:1 w4c	0.02	ECL deviates -0.004		
5.7004	21908	0.022	0.972	20.0008	20:0	0.53	ECL deviates  0.001	Reference -0.007	
5.7553	865	0.018	----	20.0769		----			
5.8035	2748	0.017	----	20.1435		----			
5.8329	8418	0.019	----	20.1841		----			
5.9452	9089	0.023	----	20.3394		----			
5.9744	40302	0.023	----	20.3799		----			
6.0523	1216	0.016	----	20.4875		----			
6.0984	4607	0.028	----	20.5513		----			
6.1467	10990	0.029	----	20.6180		----			
6.2098	5476	0.031	----	20.7053		----			
6.2774	13363	0.021	0.971	20.7989	21:1 w8c	0.33	ECL deviates  0.001		
6.3349	9197	0.025	----	20.8783		----			
6.3926	23923	0.022	0.970	20.9581	21:1 w3c	0.58	ECL deviates  0.004		
6.4274	7536	0.024	0.970	21.0062	21:0	0.18	ECL deviates  0.006	Reference  0.000	
6.5078	4654	0.022	----	21.1170		----			
6.5606	1718	0.021	----	21.1897		----			
6.5929	4437	0.022	0.969	21.2342	22:5 w6c	0.11	ECL deviates -0.018		
6.6259	6350	0.018	----	21.2797		----			
6.6476	2401	0.015	0.969	21.3096	22:6 w3c	0.06	ECL deviates -0.022		
6.6892	1577	0.022	----	21.3669		----			
6.7516	2980	0.032	0.968	21.4528	22:5 w3c	0.07	ECL deviates -0.015		
6.8754	16777	0.035	0.967	21.6234	22:0 iso	----	> max ar/ht		
6.9523	5046	0.031	0.966	21.7293	22:2 w6c	0.12	ECL deviates -0.009		
6.9884	3807	0.020	0.965	21.7790	22:1 w9c	0.09	ECL deviates  0.006		
7.0194	8497	0.031	0.965	21.8217	22:1 w8c	0.21	ECL deviates  0.008		
7.1050	7773	0.020	0.964	21.9397	22:1 w3c	0.19	ECL deviates -0.007		
7.1491	24421	0.021	0.963	22.0003	22:0	0.59	ECL deviates  0.000	Reference -0.005	
7.2131	2107	0.022	----	22.0896		----			
7.2361	2729	0.028	----	22.1218		----			
7.3218	13808	0.024	----	22.2415		----			
7.3759	2077	0.022	----	22.3170		----			
7.4427	2273	0.023	----	22.4102		----			
7.4926	2425	0.028	0.957	22.4798	23:4 w6c	0.06	ECL deviates  0.009		
7.5370	1670	0.022	----	22.5417		----			
7.6043	4536	0.036	0.954	22.6356	23:3 w3c	----	> max ar/ht		
7.6435	1851	0.021	----	22.6904		----			
7.7052	5497	0.027	----	22.7765		----			
7.7658	3296	0.023	----	22.8611		----			
7.8072	12118	0.020	0.949	22.9188	23:1 w4c	0.29	ECL deviates -0.008		
7.8659	6346	0.019	0.947	23.0007	23:0	0.15	ECL deviates  0.001	Reference -0.003	
7.9091	2496	0.024	----	23.0617		----			
8.0712	7900	0.024	----	23.2905		----			
8.3233	10530	0.030	----	23.6463		----			
8.3828	4290	0.023	----	23.7303		----			
8.4121	4421	0.026	----	23.7716		----			
8.4854	3308	0.025	----	23.8750		----			
8.5211	1034	0.014	----	23.9254		----			
8.5694	22284	0.020	0.920	23.9935	24:0	0.51	ECL deviates -0.007	Reference -0.008	
8.6757	1023	0.018	----	24.1435		----	> max rt		
8.7610	2020	0.030	----	24.2639		----	> max rt		
8.9253	12949	0.020	----	24.4957		----	> max rt		
9.2275	22086	0.022	----	24.9220		----	> max rt		
9.4644	11968	0.020	----	25.2562		----	> max rt		

ECL Deviation: 0.007                            Reference ECL Shift: 0.008       Number Reference Peaks: 20
Total Response: 4465508                       Total Named: 4069256
Percent Named: 91.13%                         Total Amount: 4013494
Profile Comment:   Column Overload:  A peak's response is greater than 400000.0.  Dilute and re-run.

(No search libraries specified in method PLFAD1.)
