# Supplementary material for: Microbial regulation of soil carbon properties under nitrogen addition and plant inputs removal
Source: PeerJ. 2019 Jul 17;7:e7343. doi: 10.7717/peerj.7343 (PMC6642627; doi:10.7717/peerj.7343)
Supplement: File S1 — The raw data showed the soil microbial PLFAs files in the year of 2015 and 2016. Each file of rtf. represented the microbial PLFAs for each soil sample. In the Supplemental File, the Excel file named “Numbers” showed the plots names and the related rtf. file names. [file peerj-07-7343-s002.zip › supplementary files/2015/48.rtf]

Volume: DATA            File: E164216.88A        Samp Ctr: 6                  ID Number: 29345 
Type: Samp                   Bottle: 17                      Method: PLFAD1 
Created: 4/21/2016 5:46:58 PM 
Sample ID: 48 


RT	Response	Ar/Ht	RFact	ECL	Peak Name	Percent	Comment1	Comment2	
0.7140	1.912E+9	0.016	----	7.6361	SOLVENT PEAK	----	< min rt		
0.8849	921	0.011	----	8.7556		----	< min rt		
0.9446	912	0.014	----	9.1462		----	< min rt		
1.0444	597	0.012	----	9.7997		----	< min rt		
1.1854	2305	0.013	1.250	10.7226	11:0 anteiso	0.06	ECL deviates  0.018	Reference  0.018	
1.2600	946	0.012	----	11.1543		----			
1.3513	1391	0.015	----	11.5919	Phthalate 1	----	ECL deviates  0.005		
1.3644	516	0.008	----	11.6547		----			
1.3893	2363	0.015	----	11.7740		----			
1.4356	5122	0.015	1.136	11.9955	12:0	0.12	ECL deviates -0.005	Reference -0.007	
1.4930	2611	0.014	----	12.2039		----			
1.5208	414	0.010	----	12.3042		----			
1.5581	1499	0.016	----	12.4386		----			
1.6037	4548	0.012	1.095	12.6027	13:0 iso	0.10	ECL deviates -0.010	Reference -0.014	
1.6311	2371	0.012	1.089	12.7015	13:0 anteiso	0.05	ECL deviates -0.008	Reference -0.012	
1.6890	624	0.012	----	12.9099		----			
1.7133	1545	0.011	1.073	12.9973	13:0	0.03	ECL deviates -0.003	Reference -0.007	
1.7790	909	0.015	----	13.1826	12:0 2OH	----	ECL deviates -0.004		
1.8716	2206	0.017	----	13.4416		----			
1.9314	68216	0.014	1.043	13.6089	14:0 iso	1.43	ECL deviates -0.005	Reference -0.011	
1.9703	1145	0.011	1.039	13.7176	14:0 anteiso	0.02	ECL deviates  0.002	Reference -0.004	
1.9914	1319	0.011	1.036	13.7767	14:1 w9c	0.03	ECL deviates -0.001		
2.0061	2168	0.013	----	13.8178		----			
2.0387	4004	0.013	1.031	13.9089	14:1 w5c	0.08	ECL deviates -0.002		
2.0710	52981	0.014	1.028	13.9992	14:0	1.10	ECL deviates -0.001	Reference -0.007	
2.0989	759	0.011	----	14.0637		----			
2.1256	1141	0.013	----	14.1240	14:0 iso 3OH	----	ECL deviates -0.001		
2.1516	3382	0.024	----	14.1828		----			
2.2130	2579	0.020	----	14.3218		----			
2.2640	56196	0.018	1.013	14.4372	15:1 iso w6c	1.15	ECL deviates -0.002		
2.2837	11009	0.011	1.011	14.4817	15:4 w3c	0.22	ECL deviates -0.009		
2.3044	16153	0.014	1.010	14.5287	15:1 anteiso w9c	0.33	ECL deviates -0.001		
2.3438	289448	0.014	1.008	14.6176	15:0 iso	5.87	ECL deviates  0.001	Reference -0.006	
2.3854	223974	0.014	1.005	14.7118	15:0 anteiso	4.53	ECL deviates  0.001	Reference -0.006	
2.4494	10054	0.025	1.001	14.8566	15:1 w6c	0.20	ECL deviates -0.003		
2.5132	28561	0.015	0.998	15.0010	15:0	0.57	ECL deviates  0.001	Reference -0.006	
2.5416	11573	0.018	----	15.0560		----			
2.6036	2918	0.020	----	15.1739		----			
2.6340	4274	0.018	----	15.2316		----			
2.7196	9749	0.016	0.990	15.3944	16:1 w7c alcohol	0.19	ECL deviates -0.002		
2.7457	46508	0.020	0.989	15.4440	15:0 DMA	0.93	ECL deviates -0.006		
2.8058	85837	0.016	0.987	15.5582	16:0 N alcohol	1.70	ECL deviates  0.001		
2.8383	118391	0.016	0.986	15.6202	16:0 iso	2.35	ECL deviates  0.000	Reference -0.007	
2.8885	10671	0.014	0.984	15.7156	16:0 anteiso	0.21	ECL deviates  0.001	Reference -0.007	
2.9166	66759	0.016	0.983	15.7691	16:1 w9c	1.32	ECL deviates -0.006		
2.9467	511834	0.017	0.983	15.8262	16:1 w7c	10.12	Column Overload		
2.9942	179323	0.015	0.981	15.9166	16:1 w5c	3.54	ECL deviates  0.005		
3.0435	551349	0.015	0.980	16.0100	16:0	10.87	Column Overload		
3.0694	20410	0.015	----	16.0533		----			
3.0859	9433	0.012	----	16.0809		----			
3.1217	3397	0.015	0.979	16.1410	16:2 DMA	0.07	ECL deviates  0.003		
3.1566	8990	0.022	----	16.1995		----			
3.1955	5073	0.019	----	16.2646		----			
3.2296	2500	0.018	0.977	16.3217	16:1 w7c DMA	0.05	ECL deviates  0.012		
3.2920	312571	0.019	0.976	16.4262	16:0 10-methyl	6.14	ECL deviates  0.006		
3.3266	54672	0.017	0.975	16.4841	17:1 iso w9c	1.07	ECL deviates -0.014		
3.3541	33586	0.018	0.975	16.5302	17:1 anteiso w9c	0.66	ECL deviates -0.006		
3.4108	73302	0.017	0.974	16.6252	17:0 iso	1.44	ECL deviates  0.001	Reference -0.007	
3.4678	83308	0.018	0.973	16.7206	17:0 anteiso	1.63	ECL deviates  0.000		
3.5111	47314	0.018	0.973	16.7931	17:1 w8c	0.93	ECL deviates -0.004		
3.5716	169272	0.018	0.972	16.8945	17:0 cyclo w7c	3.31	ECL deviates  0.001		
3.6357	22903	0.018	0.972	17.0018	17:0	0.45	ECL deviates  0.002	Reference -0.007	
3.6615	31898	0.016	0.971	17.0418	17:1 w7c 10-methyl	0.62	ECL deviates -0.001		
3.7041	9180	0.018	----	17.1067		----			
3.7379	2711	0.020	----	17.1584		----			
3.7898	3148	0.018	0.971	17.2375	16:0 2OH	0.06	ECL deviates -0.003		
3.8419	686	0.013	----	17.3169		----			
3.9011	30677	0.018	0.970	17.4072	17:0 10-methyl	0.60	ECL deviates  0.000		
3.9353	2620	0.012	0.970	17.4594	17:0 DMA	0.05	ECL deviates  0.001		
3.9581	8249	0.022	----	17.4942		----			
4.0319	41311	0.027	0.970	17.6068	18:0 iso	0.81	ECL deviates -0.020		
4.1084	93330	0.018	0.970	17.7235	18:2 w6c	1.82	ECL deviates -0.004		
4.1426	328105	0.020	0.970	17.7756	18:1 w9c	6.40	ECL deviates  0.001		
4.1803	573469	0.018	0.969	17.8331	18:1 w7c	11.19	Column Overload		
4.2343	76937	0.020	0.969	17.9155	18:1 w5c	1.50	ECL deviates -0.007		
4.2914	88782	0.018	0.969	18.0026	18:0	1.73	ECL deviates  0.003	Reference -0.006	
4.3470	36992	0.019	0.969	18.0832	18:1 w7c 10-methyl	0.72	ECL deviates -0.002		
4.4022	4975	0.015	0.969	18.1630	18:2 DMA	----	Below has same name		
4.4084	5730	0.016	----	18.1719	18:2 DMA	----	Above has same name		
4.4464	5469	0.022	0.969	18.2267	18:1 w9c DMA	0.11	ECL deviates -0.010		
4.4781	1643	0.015	0.970	18.2726	18:1 w7c DMA	0.03	ECL deviates -0.010		
4.5088	1650	0.015	----	18.3169		----			
4.5600	120543	0.020	0.970	18.3910	18:0 10-methyl	2.35	ECL deviates -0.004		
4.6293	4084	0.021	0.970	18.4910	19:4 w6c	0.08	ECL deviates  0.006		
4.6730	9334	0.025	0.970	18.5541	19:3 w6c	0.18	ECL deviates -0.006		
4.7420	6232	0.026	0.970	18.6538	19:3 w3c	0.12	ECL deviates -0.005		
4.8059	16442	0.021	----	18.7461		----			
4.8489	14117	0.020	0.970	18.8082	19:1 w8c	0.28	ECL deviates -0.003		
4.8908	18956	0.014	0.970	18.8686	19:0 cyclo w9c	0.37	ECL deviates -0.003		
4.9151	141315	0.019	0.970	18.9037	19:0 cyclo w7c	2.76	ECL deviates -0.006		
4.9854	83857	0.019	----	19.0054	19:0	----	ECL deviates  0.005		
5.0453	2567	0.016	----	19.0888		----			
5.0876	1163	0.021	----	19.1478		----			
5.1382	1778	0.018	----	19.2183		----			
5.1710	7893	0.018	----	19.2639		----			
5.2583	32230	0.029	0.971	19.3856	20:4 w6c	0.63	ECL deviates -0.018		
5.3103	10811	0.017	0.971	19.4580	20:5 w3c	0.21	ECL deviates -0.024		
5.3468	2670	0.015	----	19.5088		----			
5.3781	9538	0.021	----	19.5524		----			
5.4111	12295	0.027	----	19.5984		----			
5.5292	32640	0.027	0.972	19.7629	20:1 w9c	0.64	ECL deviates -0.010		
5.5603	14015	0.025	0.972	19.8062	20:1 w8c	0.27	ECL deviates -0.007		
5.6488	1086	0.015	0.972	19.9294	20:1 w4c	0.02	ECL deviates -0.002		
5.7002	27303	0.023	0.972	20.0010	20:0	0.53	ECL deviates  0.001	Reference -0.007	
5.7547	1337	0.016	----	20.0765		----			
5.8005	4460	0.018	----	20.1398		----			
5.8326	7855	0.020	----	20.1841		----			
5.9435	7809	0.025	----	20.3375		----			
5.9749	32594	0.024	----	20.3809		----			
6.0496	1248	0.018	----	20.4842		----			
6.1015	5372	0.029	----	20.5560		----			
6.1474	4698	0.021	----	20.6196		----			
6.1717	3006	0.016	0.971	20.6531	21:3 w3c	0.06	ECL deviates -0.001		
6.2120	4713	0.030	----	20.7089		----			
6.2757	14481	0.019	0.971	20.7970	21:1 w8c	0.28	ECL deviates -0.001		
6.3323	9282	0.025	----	20.8752		----			
6.3920	25677	0.021	0.970	20.9578	21:1 w3c	0.50	ECL deviates  0.004		
6.4270	6768	0.021	0.970	21.0062	21:0	0.13	ECL deviates  0.006	Reference -0.001	
6.5084	3649	0.022	----	21.1184		----			
6.5548	1110	0.016	----	21.1823		----			
6.5938	4304	0.024	0.969	21.2360	22:5 w6c	0.08	ECL deviates -0.016		
6.6258	5059	0.019	----	21.2801		----			
6.6490	1972	0.013	0.969	21.3121	22:6 w3c	0.04	ECL deviates -0.020		
6.6944	1094	0.019	----	21.3746		----			
6.7490	1525	0.029	0.968	21.4498	22:5 w3c	0.03	ECL deviates -0.018		
6.8765	13479	0.031	0.967	21.6254	22:0 iso	0.26	ECL deviates  0.008		
6.9485	4055	0.026	0.966	21.7246	22:2 w6c	0.08	ECL deviates -0.014		
6.9867	3528	0.022	0.965	21.7774	22:1 w9c	0.07	ECL deviates  0.004		
7.0228	4817	0.028	----	21.8270		----			
7.1043	7393	0.018	0.964	21.9393	22:1 w3c	0.14	ECL deviates -0.008		
7.1494	26375	0.020	0.963	22.0015	22:0	0.51	ECL deviates  0.001	Reference -0.004	
7.2108	2378	0.021	----	22.0871		----			
7.2415	2153	0.028	----	22.1300		----			
7.3229	11990	0.021	----	22.2436		----			
7.3771	2095	0.029	----	22.3193		----			
7.4375	1920	0.029	----	22.4036		----			
7.4947	1265	0.023	0.957	22.4835	23:4 w6c	0.02	ECL deviates  0.012		
7.5334	1072	0.020	----	22.5375		----			
7.6046	4030	0.038	0.954	22.6368	23:3 w3c	----	> max ar/ht		
7.7026	4398	0.024	----	22.7736		----			
7.7646	1776	0.025	----	22.8602		----			
7.8064	10408	0.021	0.949	22.9186	23:1 w4c	0.20	ECL deviates -0.008		
7.8649	6876	0.019	0.947	23.0002	23:0	0.13	ECL deviates  0.000	Reference -0.004	
7.9082	2008	0.025	----	23.0613		----			
8.0710	6252	0.020	----	23.2911		----			
8.2801	1593	0.026	0.933	23.5863	24:3 w6c	0.03	ECL deviates -0.004		
8.3208	7993	0.025	----	23.6438		----			
8.3788	1796	0.020	----	23.7256		----			
8.4126	3693	0.020	----	23.7733		----			
8.4878	1918	0.026	----	23.8795		----			
8.5686	25066	0.019	0.920	23.9934	24:0	0.46	ECL deviates -0.007	Reference -0.009	
8.6721	606	0.014	----	24.1396		----	> max rt		
8.7746	1937	0.042	----	24.2842		----	> max rt		
8.9262	11092	0.022	----	24.4981		----	> max rt		
9.2241	16150	0.018	----	24.9185		----	> max rt		
9.2512	3121	0.015	----	24.9568		----	> max rt		
9.4632	10121	0.021	----	25.2559		----	> max rt		

ECL Deviation: 0.008                            Reference ECL Shift: 0.008       Number Reference Peaks: 21
Total Response: 5387846                       Total Named: 5067014
Percent Named: 94.05%                         Total Amount: 4978488
Profile Comment:   Column Overload:  A peak's response is greater than 400000.0.  Dilute and re-run.

(No search libraries specified in method PLFAD1.)
