# Supplementary material for: Microbial regulation of soil carbon properties under nitrogen addition and plant inputs removal
Source: PeerJ. 2019 Jul 17;7:e7343. doi: 10.7717/peerj.7343 (PMC6642627; doi:10.7717/peerj.7343)
Supplement: File S1 — The raw data showed the soil microbial PLFAs files in the year of 2015 and 2016. Each file of rtf. represented the microbial PLFAs for each soil sample. In the Supplemental File, the Excel file named “Numbers” showed the plots names and the related rtf. file names. [file peerj-07-7343-s002.zip › supplementary files/2015/49.rtf]

Volume: DATA            File: E164216.88A        Samp Ctr: 7                  ID Number: 29346 
Type: Samp                   Bottle: 18                      Method: PLFAD1 
Created: 4/21/2016 6:06:37 PM 
Sample ID: 49 


RT	Response	Ar/Ht	RFact	ECL	Peak Name	Percent	Comment1	Comment2	
0.7145	1.896E+9	0.017	----	7.6630	SOLVENT PEAK	----	< min rt		
0.8856	2531	0.012	----	8.7778		----	< min rt		
0.9455	644	0.011	----	9.1677		----	< min rt		
1.0459	808	0.013	----	9.8222		----	< min rt		
1.0741	1283	0.020	1.329	10.0058	10:0	0.02	ECL deviates  0.006	Reference -0.004	
1.1871	3363	0.014	----	10.7423		----			
1.2259	1102	0.016	1.221	10.9949	11:0	0.02	ECL deviates -0.005	Reference -0.013	
1.2628	2093	0.015	1.203	11.1747	10:0 2OH	0.03	ECL deviates -0.009		
1.3182	1188	0.016	1.181	11.4388	10:0 3OH	0.02	ECL deviates -0.003		
1.3539	2977	0.018	1.166	11.6093	12:0 iso	0.04	ECL deviates -0.003	Reference -0.010	
1.3911	3258	0.016	----	11.7868		----			
1.4370	8344	0.015	1.136	12.0063	12:0	0.12	ECL deviates  0.006	Reference  0.000	
1.4945	4783	0.015	----	12.2126		----			
1.5227	1010	0.014	----	12.3137		----			
1.5593	3066	0.019	----	12.4453		----			
1.6053	9242	0.013	1.095	12.6103	13:0 iso	0.13	ECL deviates -0.002	Reference -0.008	
1.6321	5837	0.017	1.089	12.7065	13:0 anteiso	0.08	ECL deviates -0.003	Reference -0.009	
1.6900	1545	0.015	1.077	12.9142	13:1 w5c	0.02	ECL deviates -0.006		
1.7146	3244	0.014	1.073	13.0027	13:0	0.04	ECL deviates  0.003	Reference -0.003	
1.7814	1337	0.018	----	13.1897	12:0 2OH	----	ECL deviates  0.003		
1.8252	592	0.011	----	13.3116		----			
1.8731	4118	0.018	----	13.4454		----			
1.9330	117344	0.014	1.043	13.6125	14:0 iso	1.52	ECL deviates -0.002	Reference -0.006	
1.9675	2089	0.013	1.039	13.7088	14:0 anteiso	0.03	ECL deviates -0.007	Reference -0.012	
1.9931	2497	0.012	1.036	13.7799	14:1 w9c	0.03	ECL deviates  0.002		
2.0069	4174	0.014	----	13.8184		----			
2.0398	6193	0.014	1.031	13.9103	14:1 w5c	0.08	ECL deviates -0.001		
2.0723	104259	0.014	1.028	14.0011	14:0	1.33	ECL deviates  0.001	Reference -0.003	
2.0996	1196	0.011	----	14.0632		----			
2.1273	2101	0.015	----	14.1258	14:0 iso 3OH	----	ECL deviates  0.001		
2.1618	6818	0.022	----	14.2038		----			
2.2112	4628	0.021	----	14.3153		----			
2.2658	155626	0.020	1.013	14.4387	15:1 iso w6c	1.96	ECL deviates  0.000		
2.3063	34605	0.015	1.010	14.5301	15:1 anteiso w9c	0.43	ECL deviates  0.000		
2.3463	538801	0.015	1.008	14.6204	15:0 iso	6.75	Column Overload		
2.3877	381598	0.014	1.005	14.7138	15:0 anteiso	4.77	ECL deviates  0.003	Reference -0.001	
2.4341	4597	0.012	----	14.8187		----			
2.4507	14031	0.021	1.001	14.8563	15:1 w6c	0.17	ECL deviates -0.004		
2.5150	49194	0.016	0.998	15.0015	15:0	0.61	ECL deviates  0.001	Reference -0.002	
2.5437	15429	0.017	----	15.0562		----			
2.6050	4901	0.023	----	15.1726		----			
2.6357	10223	0.022	----	15.2310		----			
2.7464	102492	0.024	0.989	15.4413	15:0 DMA	1.26	ECL deviates -0.009		
2.8077	97624	0.016	0.987	15.5575	16:0 N alcohol	1.20	ECL deviates  0.001		
2.8411	217899	0.016	0.986	15.6211	16:0 iso	2.67	ECL deviates  0.001	Reference -0.002	
2.9196	124381	0.018	0.983	15.7701	16:1 w9c	1.52	ECL deviates -0.005		
2.9508	862540	0.018	0.983	15.8294	16:1 w7c	10.54	Column Overload		
2.9965	294336	0.016	0.981	15.9161	16:1 w5c	3.59	ECL deviates  0.005		
3.0483	995968	0.016	0.980	16.0131	16:0	12.14	Column Overload		
3.0723	22409	0.013	----	16.0534		----			
3.0878	14025	0.014	----	16.0793		----			
3.1231	8055	0.017	0.979	16.1384	16:2 DMA	0.10	ECL deviates  0.000		
3.1579	17019	0.025	----	16.1966		----			
3.1968	7625	0.019	----	16.2617		----			
3.2322	4504	0.020	0.977	16.3209	16:1 w7c DMA	0.05	ECL deviates  0.011		
3.2943	488404	0.021	0.976	16.4248	16:0 10-methyl	5.93	Column Overload		
3.3289	74403	0.018	----	16.4828		----			
3.3563	59145	0.019	0.975	16.5287	17:1 anteiso w9c	0.72	ECL deviates -0.007		
3.4116	115703	0.018	0.974	16.6212	17:0 iso	1.40	ECL deviates -0.003	Reference -0.006	
3.4694	147836	0.017	0.973	16.7179	17:0 anteiso	1.79	ECL deviates -0.002		
3.5134	98949	0.017	0.973	16.7915	17:1 w8c	1.20	ECL deviates -0.005		
3.5741	270713	0.019	0.972	16.8933	17:0 cyclo w7c	3.27	ECL deviates  0.000		
3.6385	44100	0.018	0.972	17.0010	17:0	0.53	ECL deviates  0.001	Reference -0.002	
3.6631	36290	0.017	0.971	17.0387	17:1 w7c 10-methyl	0.44	ECL deviates -0.005		
3.7053	12800	0.018	----	17.1030		----			
3.7419	3825	0.019	----	17.1589		----			
3.7902	7283	0.021	0.971	17.2326	16:0 2OH	0.09	ECL deviates -0.008		
3.8449	1378	0.014	----	17.3160		----			
3.9012	55401	0.019	0.970	17.4018	17:0 10-methyl	0.67	ECL deviates -0.005		
3.9364	3744	0.011	0.970	17.4555	17:0 DMA	0.05	ECL deviates -0.003		
3.9594	15881	0.026	----	17.4906		----			
4.0340	66214	0.030	----	17.6044		----			
4.1108	215330	0.017	0.970	17.7216	18:2 w6c	2.60	ECL deviates -0.006		
4.1466	550133	0.019	0.970	17.7762	18:1 w9c	6.63	Column Overload		
4.1851	828882	0.018	0.969	17.8348	18:1 w7c	9.99	Column Overload		
4.2344	148675	0.021	----	17.9100		----			
4.2941	158210	0.018	0.969	18.0012	18:0	1.91	ECL deviates  0.001	Reference -0.002	
4.3486	46774	0.019	0.969	18.0801	18:1 w7c 10-methyl	0.56	ECL deviates -0.005		
4.4001	16966	0.026	0.969	18.1545	18:2 DMA	0.20	ECL deviates -0.006		
4.4467	11328	0.025	----	18.2217		----			
4.4828	2299	0.010	0.970	18.2739	18:1 w7c DMA	0.03	ECL deviates -0.009		
4.5089	2384	0.016	----	18.3116		----			
4.5623	198314	0.021	0.970	18.3888	18:0 10-methyl	2.39	ECL deviates -0.006		
4.6299	7598	0.022	0.970	18.4865	19:4 w6c	0.09	ECL deviates  0.002		
4.6734	15771	0.025	0.970	18.5493	19:3 w6c	0.19	ECL deviates -0.011		
4.7294	3435	0.011	0.970	18.6303	19:0 iso	0.04	ECL deviates  0.000		
4.7454	7529	0.018	0.970	18.6534	19:3 w3c	0.09	ECL deviates -0.005		
4.8076	26946	0.022	----	18.7434		----			
4.8506	23609	0.020	0.970	18.8056	19:1 w8c	0.28	ECL deviates -0.005		
4.8932	37982	0.017	0.970	18.8671	19:0 cyclo w9c	0.46	ECL deviates -0.005		
4.9171	171673	0.018	0.970	18.9016	19:0 cyclo w7c	2.07	ECL deviates -0.008		
4.9868	93020	0.019	----	19.0023	19:0	----	ECL deviates  0.002		
5.0455	5848	0.018	----	19.0843		----			
5.0885	757	0.016	----	19.1442		----			
5.1357	7628	0.021	----	19.2099		----			
5.1731	21360	0.020	----	19.2621		----			
5.2455	81902	0.032	----	19.3630		----			
5.3137	21512	0.022	0.971	19.4582	20:5 w3c	----	Below has same name		
5.3456	5016	0.017	----	19.5027	20:5 w3c	----	Above has same name		
5.3790	12872	0.020	----	19.5492		----			
5.4129	23148	0.027	----	19.5965		----			
5.5311	57211	0.027	0.972	19.7613	20:1 w9c	0.69	ECL deviates -0.011		
5.5623	20042	0.024	0.972	19.8049	20:1 w8c	0.24	ECL deviates -0.008		
5.7012	48431	0.023	0.972	19.9985	20:0	0.59	ECL deviates -0.001	Reference -0.005	
5.7570	3629	0.021	----	20.0758		----			
5.8002	5937	0.017	----	20.1358		----			
5.8322	14455	0.020	----	20.1801		----			
5.8715	776	0.011	----	20.2345		----			
5.9110	7562	0.017	----	20.2892		----			
5.9452	11621	0.019	----	20.3366		----			
5.9745	52524	0.023	----	20.3771		----			
6.0480	1687	0.015	----	20.4790		----			
6.1023	9683	0.029	----	20.5543		----			
6.1449	10513	0.023	----	20.6133		----			
6.1753	6165	0.017	0.971	20.6553	21:3 w3c	0.07	ECL deviates  0.002		
6.2195	11874	0.029	----	20.7166		----			
6.2758	21697	0.022	0.971	20.7946	21:1 w8c	0.26	ECL deviates -0.003		
6.3323	25381	0.023	----	20.8728		----			
6.3930	34267	0.021	0.970	20.9569	21:1 w3c	0.41	ECL deviates  0.003		
6.4261	13572	0.027	0.970	21.0029	21:0	0.16	ECL deviates  0.003	Reference -0.002	
6.5073	6470	0.022	----	21.1150		----			
6.5504	3613	0.021	----	21.1745		----			
6.5918	10194	0.023	0.969	21.2316	22:5 w6c	0.12	ECL deviates -0.020		
6.6260	15901	0.020	----	21.2789		----			
6.6505	2796	0.011	0.969	21.3128	22:6 w3c	0.03	ECL deviates -0.019		
6.6914	2836	0.018	----	21.3692		----			
6.7548	3902	0.035	0.968	21.4567	22:5 w3c	0.05	ECL deviates -0.011		
6.8147	2090	0.017	----	21.5396		----			
6.8763	24558	0.026	0.967	21.6246	22:0 iso	0.30	ECL deviates  0.007		
6.9521	5976	0.025	0.966	21.7293	22:2 w6c	0.07	ECL deviates -0.009		
6.9866	8829	0.021	0.965	21.7770	22:1 w9c	0.11	ECL deviates  0.004		
7.0171	11561	0.026	0.965	21.8191	22:1 w8c	0.14	ECL deviates  0.006		
7.1040	8932	0.021	0.964	21.9390	22:1 w3c	0.11	ECL deviates -0.008		
7.1487	51846	0.018	0.963	22.0007	22:0	0.62	ECL deviates  0.001	Reference -0.005	
7.2067	3592	0.026	----	22.0819		----			
7.2477	2900	0.028	----	22.1393		----			
7.3225	13193	0.019	----	22.2441		----			
7.3762	2208	0.030	----	22.3192		----			
7.4373	1380	0.021	----	22.4048		----			
7.4931	729	0.015	0.957	22.4829	23:4 w6c	0.01	ECL deviates  0.012		
7.5335	1299	0.015	----	22.5394		----			
7.6038	2248	0.022	0.954	22.6378	23:3 w3c	0.03	ECL deviates -0.007		
7.7014	6654	0.021	----	22.7745		----			
7.7666	4483	0.024	----	22.8658		----			
7.8064	13234	0.021	0.949	22.9215	23:1 w4c	0.16	ECL deviates -0.005		
7.8642	15103	0.020	0.947	23.0025	23:0	0.18	ECL deviates  0.003	Reference -0.005	
7.9066	3201	0.027	----	23.0624		----			
7.9735	1022	0.019	----	23.1572		----			
8.0341	1281	0.019	----	23.2430		----			
8.0708	9859	0.018	----	23.2950		----			
8.2811	1515	0.017	0.933	23.5931	24:3 w6c	0.02	ECL deviates  0.003		
8.3202	15480	0.025	0.931	23.6484	24:3 w3c	0.18	ECL deviates -0.006		
8.3768	5680	0.023	----	23.7286		----			
8.4129	6474	0.027	0.927	23.7796	24:1 w9c	0.07	ECL deviates -0.007		
8.4833	6243	0.031	----	23.8794		----			
8.5655	41788	0.019	0.920	23.9959	24:0	0.48	ECL deviates -0.004	Reference -0.013	
8.6713	1447	0.018	----	24.1458		----	> max rt		
8.7615	2565	0.031	----	24.2736		----	> max rt		
8.7932	916	0.017	----	24.3186		----	> max rt		
8.9238	20469	0.020	----	24.5036		----	> max rt		
9.0248	1522	0.021	----	24.6467		----	> max rt		
9.1503	6531	0.019	----	24.8245		----	> max rt		
9.2251	20450	0.022	----	24.9306		----	> max rt		
9.2549	4652	0.015	----	24.9729		----	> max rt		
9.4620	11820	0.023	----	25.2663		----	> max rt		
9.4909	1746	0.011	----	25.3072		----	> max rt		

ECL Deviation: 0.007                            Reference ECL Shift: 0.007       Number Reference Peaks: 21
Total Response: 9116174                       Total Named: 8189519
Percent Named: 89.84%                         Total Amount: 8062456
Profile Comment:   Column Overload:  A peak's response is greater than 400000.0.  Dilute and re-run.

(No search libraries specified in method PLFAD1.)
