# Supplementary material for: Microbial regulation of soil carbon properties under nitrogen addition and plant inputs removal
Source: PeerJ. 2019 Jul 17;7:e7343. doi: 10.7717/peerj.7343 (PMC6642627; doi:10.7717/peerj.7343)
Supplement: File S1 — The raw data showed the soil microbial PLFAs files in the year of 2015 and 2016. Each file of rtf. represented the microbial PLFAs for each soil sample. In the Supplemental File, the Excel file named “Numbers” showed the plots names and the related rtf. file names. [file peerj-07-7343-s002.zip › supplementary files/2015/5.rtf]

Volume: DATA            File: E164203.63A        Samp Ctr: 7                  ID Number: 29302 
Type: Samp                   Bottle: 6                        Method: PLFAD1 
Created: 4/20/2016 11:35:11 AM 
Sample ID: 5 


RT	Response	Ar/Ht	RFact	ECL	Peak Name	Percent	Comment1	Comment2	
0.7146	1.884E+9	0.015	----	7.6548	SOLVENT PEAK	----	< min rt		
0.7878	5181	0.018	----	8.1312		----	< min rt		
0.8869	1068	0.010	----	8.7824		----	< min rt		
0.9468	811	0.015	----	9.1717		----	< min rt		
1.1877	1661	0.013	----	10.7385		----			
1.2642	604	0.011	1.208	11.1739	10:0 2OH	0.02	ECL deviates -0.010		
1.3552	840	0.015	1.170	11.6069	12:0 iso	0.03	ECL deviates -0.005	Reference -0.008	
1.3668	367	0.008	----	11.6620		----			
1.3916	1258	0.014	----	11.7799		----			
1.4391	2942	0.014	1.138	12.0050	12:0	0.11	ECL deviates  0.005	Reference  0.002	
1.4969	1578	0.013	----	12.2120		----			
1.5616	906	0.014	----	12.4438		----			
1.6077	2978	0.012	1.094	12.6088	13:0 iso	0.10	ECL deviates -0.004	Reference -0.006	
1.6354	1684	0.013	1.088	12.7079	13:0 anteiso	0.06	ECL deviates -0.001	Reference -0.004	
1.6929	497	0.011	1.075	12.9137	13:1 w5c	0.02	ECL deviates -0.006		
1.7176	1038	0.012	1.071	13.0023	13:0	0.04	ECL deviates  0.002	Reference  0.000	
1.8762	1334	0.017	----	13.4437		----			
1.9365	40765	0.013	1.038	13.6114	14:0 iso	1.34	ECL deviates -0.003	Reference -0.004	
1.9768	810	0.012	1.033	13.7238	14:0 anteiso	0.03	ECL deviates  0.008	Reference  0.006	
1.9966	908	0.011	1.031	13.7788	14:1 w9c	0.03	ECL deviates  0.001		
2.0110	1396	0.012	----	13.8190		----			
2.0447	2239	0.012	1.025	13.9127	14:1 w5c	0.07	ECL deviates  0.002		
2.0767	35308	0.013	1.021	14.0018	14:0	1.14	ECL deviates  0.002	Reference  0.000	
2.1044	520	0.011	----	14.0641		----			
2.1315	640	0.011	----	14.1251	14:0 iso 3OH	----	ECL deviates  0.000		
2.1580	2271	0.022	----	14.1848		----			
2.2229	1622	0.021	----	14.3307		----			
2.2695	43590	0.018	1.005	14.4357	15:1 iso w6c	1.39	ECL deviates -0.003		
2.2889	9082	0.012	1.003	14.4793	15:4 w3c	0.29	ECL deviates -0.011		
2.3102	10820	0.014	1.001	14.5273	15:1 anteiso w9c	0.34	ECL deviates -0.003		
2.3490	212225	0.014	0.999	14.6146	15:0 iso	6.71	ECL deviates -0.002	Reference -0.003	
2.3911	158427	0.014	0.996	14.7093	15:0 anteiso	4.99	ECL deviates -0.002	Reference -0.003	
2.4160	844	0.008	0.994	14.7654	15:1 w9c	0.03	ECL deviates -0.006		
2.4563	5933	0.021	0.991	14.8560	15:1 w6c	0.19	ECL deviates -0.004		
2.5201	20399	0.014	0.987	14.9997	15:0	0.64	ECL deviates  0.000	Reference -0.001	
2.5488	8510	0.016	----	15.0542		----			
2.6100	737	0.011	----	15.1704		----			
2.6408	1276	0.016	----	15.2288		----			
2.7264	6920	0.014	0.978	15.3913	16:1 w7c alcohol	0.21	ECL deviates -0.005		
2.7522	34405	0.020	0.976	15.4402	15:0 DMA	1.06	ECL deviates -0.010		
2.8132	66646	0.015	0.974	15.5560	16:0 N alcohol	2.06	ECL deviates -0.001		
2.8458	80443	0.015	0.973	15.6179	16:0 iso	2.48	ECL deviates -0.002	Reference -0.003	
2.8976	8143	0.015	0.971	15.7162	16:0 anteiso	0.25	ECL deviates  0.001	Reference  0.000	
2.9244	50358	0.016	0.970	15.7670	16:1 w9c	1.55	ECL deviates -0.008		
2.9538	337626	0.017	0.969	15.8227	16:1 w7c	10.36	ECL deviates -0.002		
3.0008	96313	0.015	0.968	15.9120	16:1 w5c	2.95	ECL deviates  0.001		
3.0499	337601	0.015	0.966	16.0047	16:0	10.33	ECL deviates  0.005	Reference  0.004	
3.0776	20938	0.019	----	16.0508		----			
3.1316	2432	0.014	0.964	16.1410	16:2 DMA	0.07	ECL deviates  0.003		
3.1667	5945	0.022	----	16.1997		----			
3.2034	3480	0.018	----	16.2612		----			
3.2414	1823	0.020	0.962	16.3245	16:1 w7c DMA	0.06	ECL deviates  0.015		
3.2998	217446	0.020	0.961	16.4222	16:0 10-methyl	6.62	ECL deviates  0.002		
3.3359	43044	0.018	----	16.4825		----			
3.3639	27044	0.019	0.960	16.5293	17:1 anteiso w9c	0.82	ECL deviates -0.007		
3.4193	48863	0.015	0.959	16.6219	17:0 iso	1.48	ECL deviates -0.002	Reference -0.002	
3.4760	58356	0.018	0.958	16.7166	17:0 anteiso	1.77	ECL deviates -0.004		
3.5209	31410	0.018	0.957	16.7916	17:1 w8c	0.95	ECL deviates -0.005		
3.5811	110400	0.019	0.957	16.8922	17:0 cyclo w7c	3.34	ECL deviates -0.001		
3.6460	15004	0.018	0.956	17.0006	17:0	0.45	ECL deviates  0.001	Reference  0.000	
3.6712	24006	0.016	0.956	17.0391	17:1 w7c 10-methyl	0.73	ECL deviates -0.004		
3.7144	5729	0.016	----	17.1048		----			
3.7506	1490	0.019	----	17.1600		----			
3.7983	2418	0.019	0.955	17.2327	16:0 2OH	0.07	ECL deviates -0.007		
3.9098	20836	0.018	0.954	17.4026	17:0 10-methyl	0.63	ECL deviates -0.004		
3.9463	2388	0.012	0.954	17.4581	17:0 DMA	0.07	ECL deviates  0.000		
3.9663	7020	0.022	----	17.4887		----			
4.0431	27064	0.029	----	17.6056		----			
4.1183	72641	0.018	0.953	17.7202	18:2 w6c	2.19	ECL deviates -0.007		
4.1509	217657	0.019	0.953	17.7698	18:1 w9c	6.57	ECL deviates -0.005		
4.1881	350897	0.018	0.953	17.8266	18:1 w7c	10.59	ECL deviates  0.000		
4.2441	40000	0.024	----	17.9118		----			
4.3011	54155	0.017	0.953	17.9987	18:0	1.63	ECL deviates -0.001	Reference -0.002	
4.3564	22069	0.018	0.953	18.0788	18:1 w7c 10-methyl	0.67	ECL deviates -0.006		
4.4109	5591	0.022	0.953	18.1574	18:2 DMA	0.17	ECL deviates -0.003		
4.4259	2663	0.012	----	18.1791		----			
4.4572	5035	0.033	0.953	18.2243	18:1 w9c DMA	0.15	ECL deviates -0.013		
4.5186	1544	0.017	----	18.3132		----			
4.5697	92251	0.020	0.954	18.3869	18:0 10-methyl	2.79	ECL deviates -0.008		
4.6407	2827	0.019	0.954	18.4896	19:4 w6c	0.09	ECL deviates  0.005		
4.6863	6876	0.024	0.954	18.5554	19:3 w6c	0.21	ECL deviates -0.005		
4.7547	4522	0.026	0.955	18.6543	19:3 w3c	0.14	ECL deviates -0.004		
4.8160	11027	0.021	----	18.7428		----			
4.8614	10101	0.020	0.955	18.8085	19:1 w8c	0.31	ECL deviates -0.002		
4.8990	16170	0.015	0.955	18.8628	19:0 cyclo w9c	0.49	ECL deviates -0.009		
4.9254	93068	0.017	0.955	18.9009	19:0 cyclo w7c	2.82	ECL deviates -0.009		
4.9943	78750	0.019	----	19.0005	19:0	----	ECL deviates  0.001		
5.0567	1510	0.016	----	19.0877		----			
5.1518	1985	0.020	----	19.2203		----			
5.1830	7392	0.019	----	19.2639		----			
5.2694	21256	0.026	----	19.3844		----			
5.3228	7528	0.018	0.958	19.4590	20:5 w3c	0.23	ECL deviates -0.023		
5.3583	1889	0.017	----	19.5085		----			
5.3879	4592	0.019	----	19.5497		----			
5.4228	7965	0.024	----	19.5985		----			
5.5400	19699	0.027	0.960	19.7621	20:1 w9c	0.60	ECL deviates -0.011		
5.5691	9933	0.023	0.960	19.8026	20:1 w8c	0.30	ECL deviates -0.010		
5.7089	17750	0.022	0.961	19.9978	20:0	0.54	ECL deviates -0.002	Reference -0.004	
5.8126	2270	0.019	----	20.1405		----			
5.8442	4772	0.019	----	20.1840		----			
5.9855	28244	0.030	----	20.3783		----			
6.0560	887	0.015	----	20.4754		----			
6.1116	2427	0.026	----	20.5519		----			
6.1598	7577	0.026	----	20.6182		----			
6.2191	3385	0.030	----	20.6998		----			
6.2852	11061	0.019	0.965	20.7908	21:1 w8c	0.34	ECL deviates -0.007		
6.3447	6384	0.022	----	20.8726		----			
6.4015	20017	0.018	0.966	20.9508	21:1 w3c	0.61	ECL deviates -0.003		
6.4384	6458	0.023	0.966	21.0016	21:0	0.20	ECL deviates  0.002	Reference  0.000	
6.5190	4034	0.022	----	21.1130		----			
6.6019	4016	0.027	----	21.2276		----			
6.6370	6402	0.022	----	21.2760		----			
6.8858	10041	0.028	0.968	21.6199	22:0 iso	0.31	ECL deviates  0.002		
6.9635	2439	0.023	0.968	21.7273	22:2 w6c	0.07	ECL deviates -0.011		
6.9961	2364	0.020	0.968	21.7723	22:1 w9c	0.07	ECL deviates -0.001		
7.0328	5181	0.025	0.968	21.8230	22:1 w8c	0.16	ECL deviates  0.010		
7.1155	5541	0.019	0.969	21.9374	22:1 w3c	0.17	ECL deviates -0.010		
7.1578	17798	0.020	0.969	21.9958	22:0	0.55	ECL deviates -0.004	Reference -0.007	
7.2221	1555	0.020	----	22.0858		----			
7.3345	8408	0.021	----	22.2434		----			
7.6137	1437	0.025	----	22.6345		----			
7.7150	2184	0.018	----	22.7765		----			
7.7722	1097	0.022	----	22.8566		----			
7.8184	8308	0.019	0.967	22.9214	23:1 w4c	0.25	ECL deviates -0.005		
7.8765	3980	0.019	0.966	23.0028	23:0	0.12	ECL deviates  0.003	Reference -0.001	
7.9194	1275	0.020	----	23.0638		----			
8.0815	4976	0.019	----	23.2940		----			
8.3329	6530	0.025	0.960	23.6513	24:3 w3c	0.20	ECL deviates -0.003		
8.3922	1527	0.020	----	23.7356		----			
8.4222	2725	0.024	0.959	23.7781	24:1 w9c	0.08	ECL deviates -0.008		
8.4998	2560	0.025	----	23.8884		----			
8.5351	624	0.013	0.957	23.9385	24:1 w3c	0.02	ECL deviates -0.010		
8.5788	16462	0.019	0.956	24.0007	24:0	0.50	ECL deviates  0.001	Reference -0.004	
8.9345	8909	0.018	----	24.5061		----	> max rt		
9.2379	18137	0.020	----	24.9373		----	> max rt		
9.4737	7690	0.020	----	25.2724		----	> max rt		

ECL Deviation: 0.006                            Reference ECL Shift: 0.004       Number Reference Peaks: 22
Total Response: 3596499                       Total Named: 3261115
Percent Named: 90.67%                         Total Amount: 3158451

(No search libraries specified in method PLFAD1.)
