# Supplementary material for: Microbial regulation of soil carbon properties under nitrogen addition and plant inputs removal
Source: PeerJ. 2019 Jul 17;7:e7343. doi: 10.7717/peerj.7343 (PMC6642627; doi:10.7717/peerj.7343)
Supplement: File S1 — The raw data showed the soil microbial PLFAs files in the year of 2015 and 2016. Each file of rtf. represented the microbial PLFAs for each soil sample. In the Supplemental File, the Excel file named “Numbers” showed the plots names and the related rtf. file names. [file peerj-07-7343-s002.zip › supplementary files/2015/50.rtf]

Volume: DATA            File: E164216.88A        Samp Ctr: 8                  ID Number: 29347 
Type: Samp                   Bottle: 19                      Method: PLFAD1 
Created: 4/21/2016 6:26:03 PM 
Sample ID: 50 


RT	Response	Ar/Ht	RFact	ECL	Peak Name	Percent	Comment1	Comment2	
0.7139	1.906E+9	0.016	----	7.6382	SOLVENT PEAK	----	< min rt		
0.8851	525	0.007	----	8.7591		----	< min rt		
0.9441	536	0.011	----	9.1450		----	< min rt		
1.1855	2875	0.013	1.250	10.7243	11:0 anteiso	0.06	ECL deviates  0.019	Reference  0.018	
1.2231	642	0.013	----	10.9703		----			
1.2605	1425	0.014	----	11.1577		----			
1.3174	748	0.016	1.181	11.4305	10:0 3OH	0.02	ECL deviates -0.011		
1.3518	1239	0.016	----	11.5949	Phthalate 1	----	ECL deviates  0.008		
1.3647	492	0.009	----	11.6571		----			
1.3891	1907	0.015	----	11.7736		----			
1.4350	5088	0.014	1.136	11.9938	12:0	0.10	ECL deviates -0.006	Reference -0.010	
1.4925	2852	0.014	----	12.2031		----			
1.5581	1367	0.014	----	12.4393		----			
1.6036	5043	0.012	1.095	12.6030	13:0 iso	0.10	ECL deviates -0.009	Reference -0.014	
1.6311	2692	0.014	1.089	12.7018	13:0 anteiso	0.05	ECL deviates -0.008	Reference -0.012	
1.6882	659	0.012	----	12.9075		----			
1.7130	1752	0.012	1.073	12.9968	13:0	0.03	ECL deviates -0.003	Reference -0.008	
1.7802	908	0.015	----	13.1866	12:0 2OH	----	ECL deviates  0.000		
1.8238	526	0.011	----	13.3082		----			
1.8721	3002	0.017	----	13.4435		----			
1.9313	62809	0.013	1.043	13.6088	14:0 iso	1.13	ECL deviates -0.005	Reference -0.011	
1.9687	1800	0.014	1.039	13.7134	14:0 anteiso	0.03	ECL deviates -0.003	Reference -0.009	
1.9910	1974	0.011	1.036	13.7757	14:1 w9c	0.04	ECL deviates -0.002		
2.0060	2947	0.014	----	13.8178		----			
2.0383	5251	0.014	1.031	13.9079	14:1 w5c	0.09	ECL deviates -0.003		
2.0707	66639	0.014	1.028	13.9986	14:0	1.18	ECL deviates -0.001	Reference -0.008	
2.0995	1056	0.013	----	14.0651		----			
2.1253	1501	0.013	----	14.1237	14:0 iso 3OH	----	ECL deviates -0.001		
2.1585	5591	0.022	----	14.1986		----			
2.2088	3409	0.021	----	14.3124		----			
2.2640	107014	0.019	1.013	14.4375	15:1 iso w6c	1.87	ECL deviates -0.002		
2.3040	23021	0.014	1.010	14.5279	15:1 anteiso w9c	0.40	ECL deviates -0.002		
2.3439	383273	0.015	1.008	14.6182	15:0 iso	6.67	ECL deviates  0.001	Reference -0.006	
2.3854	282326	0.014	1.005	14.7120	15:0 anteiso	4.90	ECL deviates  0.001	Reference -0.006	
2.4494	13978	0.027	1.001	14.8567	15:1 w6c	0.24	ECL deviates -0.003		
2.5132	35398	0.014	0.998	15.0012	15:0	0.61	ECL deviates  0.001	Reference -0.006	
2.5418	11343	0.018	----	15.0565		----			
2.6035	3533	0.021	----	15.1737		----			
2.6334	7568	0.021	----	15.2307		----			
2.7204	10084	0.014	0.990	15.3960	16:1 w7c alcohol	0.17	ECL deviates -0.001		
2.7451	58676	0.021	0.989	15.4430	15:0 DMA	1.00	ECL deviates -0.007		
2.8062	91756	0.015	0.987	15.5591	16:0 N alcohol	1.56	ECL deviates  0.002		
2.8388	140503	0.016	0.986	15.6212	16:0 iso	2.39	ECL deviates  0.001	Reference -0.006	
2.8902	14877	0.014	0.984	15.7188	16:0 anteiso	0.25	ECL deviates  0.004	Reference -0.004	
2.9179	81511	0.015	0.983	15.7715	16:1 w9c	1.38	ECL deviates -0.003		
2.9475	560658	0.017	0.983	15.8277	16:1 w7c	9.51	Column Overload		
2.9944	199580	0.017	0.981	15.9168	16:1 w5c	3.38	ECL deviates  0.006		
3.0451	740779	0.015	0.980	16.0127	16:0	12.54	Column Overload		
3.0699	14090	0.013	----	16.0542		----			
3.0854	7183	0.012	----	16.0801		----			
3.1227	5684	0.018	0.979	16.1426	16:2 DMA	0.10	ECL deviates  0.005		
3.1572	10972	0.023	----	16.2004		----			
3.1928	5536	0.018	----	16.2601		----			
3.2301	3281	0.019	0.977	16.3225	16:1 w7c DMA	0.06	ECL deviates  0.012		
3.2919	313584	0.021	0.976	16.4259	16:0 10-methyl	5.28	ECL deviates  0.006		
3.3281	63814	0.019	0.975	16.4867	17:1 iso w9c	1.07	ECL deviates -0.011		
3.3556	39833	0.018	0.975	16.5327	17:1 anteiso w9c	0.67	ECL deviates -0.003		
3.4111	84403	0.016	0.974	16.6256	17:0 iso	1.42	ECL deviates  0.002	Reference -0.006	
3.4688	99173	0.018	0.973	16.7222	17:0 anteiso	1.67	ECL deviates  0.002		
3.5125	65345	0.018	0.973	16.7954	17:1 w8c	1.10	ECL deviates -0.002		
3.5716	166485	0.020	0.972	16.8944	17:0 cyclo w7c	2.80	ECL deviates  0.001		
3.6368	31093	0.018	0.972	17.0035	17:0	0.52	ECL deviates  0.003	Reference -0.005	
3.6620	24358	0.017	0.971	17.0424	17:1 w7c 10-methyl	0.41	ECL deviates -0.001		
3.7042	7724	0.018	----	17.1068		----			
3.7395	2974	0.020	----	17.1607		----			
3.7902	5716	0.022	0.971	17.2380	16:0 2OH	0.10	ECL deviates -0.002		
3.8433	933	0.014	----	17.3191		----			
3.9000	35823	0.019	0.970	17.4056	17:0 10-methyl	0.60	ECL deviates -0.001		
3.9380	2929	0.013	0.970	17.4634	17:0 DMA	0.05	ECL deviates  0.005		
3.9581	9961	0.027	----	17.4941		----			
4.0123	15786	0.014	0.970	17.5768	18:3 w6c	0.26	ECL deviates -0.003		
4.0334	38448	0.021	0.970	17.6090	18:0 iso	0.64	ECL deviates -0.018		
4.1080	150514	0.019	0.970	17.7227	18:2 w6c	2.52	ECL deviates -0.004		
4.1439	342016	0.020	0.970	17.7776	18:1 w9c	5.73	ECL deviates  0.003		
4.1809	607903	0.018	0.969	17.8339	18:1 w7c	10.18	Column Overload		
4.2329	100802	0.021	0.969	17.9132	18:1 w5c	1.69	ECL deviates -0.010		
4.2915	110645	0.018	0.969	18.0027	18:0	1.85	ECL deviates  0.003	Reference -0.006	
4.3464	31839	0.019	0.969	18.0823	18:1 w7c 10-methyl	0.53	ECL deviates -0.003		
4.4001	13019	0.026	0.969	18.1598	18:2 DMA	0.22	ECL deviates  0.000		
4.4434	7016	0.021	0.969	18.2225	18:1 w9c DMA	0.12	ECL deviates -0.015		
4.4818	2887	0.017	0.970	18.2779	18:1 w7c DMA	0.05	ECL deviates -0.005		
4.5057	1934	0.016	----	18.3125		----			
4.5599	106455	0.022	0.970	18.3907	18:0 10-methyl	1.78	ECL deviates -0.004		
4.6245	3727	0.018	0.970	18.4840	19:4 w6c	0.06	ECL deviates -0.001		
4.6734	11434	0.027	0.970	18.5547	19:3 w6c	0.19	ECL deviates -0.005		
4.7293	3498	0.014	0.970	18.6354	19:0 iso	0.06	ECL deviates  0.005		
4.7463	4805	0.018	0.970	18.6600	19:3 w3c	0.08	ECL deviates  0.002		
4.8065	18521	0.024	----	18.7470		----			
4.8494	16790	0.021	0.970	18.8089	19:1 w8c	0.28	ECL deviates -0.002		
4.8898	23318	0.017	0.970	18.8673	19:0 cyclo w9c	0.39	ECL deviates -0.005		
4.9141	102463	0.017	0.970	18.9024	19:0 cyclo w7c	1.72	ECL deviates -0.007		
4.9833	83692	0.019	----	19.0023	19:0	----	ECL deviates  0.002		
5.0455	6567	0.023	----	19.0892		----			
5.1366	6466	0.023	----	19.2162		----			
5.1704	12492	0.021	----	19.2633		----			
5.2419	61160	0.032	----	19.3627		----			
5.3119	16340	0.021	0.971	19.4603	20:5 w3c	0.27	ECL deviates -0.022		
5.3480	4135	0.015	----	19.5106		----			
5.3776	18238	0.021	----	19.5518		----			
5.4118	11364	0.017	----	19.5995		----			
5.4322	9734	0.020	0.971	19.6278	20:0 iso	0.16	ECL deviates  0.000	Reference -0.008	
5.5287	41798	0.027	0.972	19.7623	20:1 w9c	0.70	ECL deviates -0.010		
5.5612	17011	0.027	0.972	19.8075	20:1 w8c	0.29	ECL deviates -0.005		
5.6514	2144	0.017	0.972	19.9331	20:1 w4c	0.04	ECL deviates  0.002		
5.6996	39013	0.023	0.972	20.0003	20:0	0.65	ECL deviates  0.000	Reference -0.008	
5.7547	3741	0.020	----	20.0766		----			
5.8003	6717	0.018	----	20.1397		----			
5.8332	11483	0.021	----	20.1852		----			
5.9109	6781	0.020	----	20.2927		----			
5.9424	9551	0.018	----	20.3363		----			
5.9739	41425	0.025	----	20.3798		----			
6.1007	13207	0.041	----	20.5553		----	> max ar/ht		
6.1465	7973	0.022	----	20.6185		----			
6.1671	4663	0.016	0.971	20.6471	21:3 w3c	0.08	ECL deviates -0.007		
6.2224	10731	0.033	----	20.7235		----			
6.2751	14618	0.023	0.971	20.7965	21:1 w8c	0.25	ECL deviates -0.001		
6.3309	18547	0.027	----	20.8736		----			
6.3902	25082	0.022	0.970	20.9556	21:1 w3c	0.42	ECL deviates  0.002		
6.4245	12790	0.031	0.970	21.0030	21:0	0.21	ECL deviates  0.003	Reference -0.004	
6.5089	6158	0.026	----	21.1194		----			
6.5513	3493	0.019	----	21.1779		----			
6.5928	8160	0.027	0.969	21.2351	22:5 w6c	0.14	ECL deviates -0.017		
6.6251	9250	0.019	----	21.2796		----			
6.6492	3673	0.015	0.969	21.3128	22:6 w3c	0.06	ECL deviates -0.019		
6.6898	2871	0.024	----	21.3687		----			
6.7540	4430	0.034	0.968	21.4572	22:5 w3c	0.07	ECL deviates -0.010		
6.8385	4429	0.028	----	21.5737		----			
6.8750	20554	0.028	0.967	21.6240	22:0 iso	0.34	ECL deviates  0.006		
6.9567	6414	0.026	0.966	21.7366	22:2 w6c	0.11	ECL deviates -0.002		
6.9871	6813	0.022	0.965	21.7785	22:1 w9c	0.11	ECL deviates  0.006		
7.0191	12323	0.033	0.965	21.8226	22:1 w8c	0.21	ECL deviates  0.009		
7.1047	9672	0.022	0.964	21.9405	22:1 w3c	0.16	ECL deviates -0.006		
7.1485	43695	0.019	0.963	22.0009	22:0	0.73	ECL deviates  0.001	Reference -0.005	
7.2085	4112	0.025	----	22.0846		----			
7.2405	3752	0.023	----	22.1293		----			
7.2629	2007	0.017	----	22.1606		----			
7.3227	15664	0.023	----	22.2442		----			
7.3771	2157	0.018	----	22.3201		----			
7.4034	2239	0.020	----	22.3568		----			
7.4403	3591	0.031	----	22.4083		----			
7.4944	2759	0.028	0.957	22.4839	23:4 w6c	0.05	ECL deviates  0.013		
7.5350	2724	0.026	----	22.5405		----			
7.6025	6283	0.036	0.954	22.6348	23:3 w3c	----	> max ar/ht		
7.6425	1430	0.014	----	22.6906		----			
7.7023	6107	0.027	----	22.7742		----			
7.7361	1257	0.011	----	22.8213		----			
7.7629	4011	0.026	----	22.8588		----			
7.8056	11880	0.022	0.949	22.9184	23:1 w4c	0.19	ECL deviates -0.008		
7.8648	14200	0.019	0.947	23.0010	23:0	0.23	ECL deviates  0.001	Reference -0.004	
7.9081	3411	0.029	----	23.0622		----			
8.0698	8608	0.022	----	23.2905		----			
8.2837	2013	0.018	0.933	23.5927	24:3 w6c	0.03	ECL deviates  0.002		
8.3213	13311	0.023	----	23.6457		----			
8.3764	5389	0.022	----	23.7235		----			
8.4109	7029	0.021	----	23.7723		----			
8.4488	627	0.010	----	23.8257		----			
8.4868	3414	0.026	----	23.8794		----			
8.5237	1011	0.015	----	23.9316		----			
8.5684	36543	0.020	0.920	23.9946	24:0	0.58	ECL deviates -0.005	Reference -0.009	
8.6724	1098	0.018	----	24.1415		----	> max rt		
8.7528	3983	0.047	----	24.2549		----	> max rt		
8.9246	11660	0.021	----	24.4975		----	> max rt		
9.2255	17445	0.022	----	24.9223		----	> max rt		
9.2581	4194	0.016	----	24.9683		----	> max rt		
9.4659	11178	0.027	----	25.2616		----	> max rt		

ECL Deviation: 0.007                            Reference ECL Shift: 0.009       Number Reference Peaks: 22
Total Response: 6404374                       Total Named: 5901313
Percent Named: 92.15%                         Total Amount: 5796825
Profile Comment:   Column Overload:  A peak's response is greater than 400000.0.  Dilute and re-run.

(No search libraries specified in method PLFAD1.)
