# Supplementary material for: Microbial regulation of soil carbon properties under nitrogen addition and plant inputs removal
Source: PeerJ. 2019 Jul 17;7:e7343. doi: 10.7717/peerj.7343 (PMC6642627; doi:10.7717/peerj.7343)
Supplement: File S1 — The raw data showed the soil microbial PLFAs files in the year of 2015 and 2016. Each file of rtf. represented the microbial PLFAs for each soil sample. In the Supplemental File, the Excel file named “Numbers” showed the plots names and the related rtf. file names. [file peerj-07-7343-s002.zip › supplementary files/2015/51.rtf]

Volume: DATA            File: E164216.88A        Samp Ctr: 9                  ID Number: 29348 
Type: Samp                   Bottle: 20                      Method: PLFAD1 
Created: 4/21/2016 6:45:41 PM 
Sample ID: 51 


RT	Response	Ar/Ht	RFact	ECL	Peak Name	Percent	Comment1	Comment2	
0.7140	1.904E+9	0.017	----	7.6506	SOLVENT PEAK	----	< min rt		
0.8851	1219	0.010	----	8.7671		----	< min rt		
1.1863	1127	0.012	----	10.7377		----			
1.2618	616	0.012	----	11.1704		----			
1.3525	1076	0.017	1.166	11.6038	12:0 iso	0.04	ECL deviates -0.008		
1.3648	482	0.009	----	11.6622		----			
1.3897	1382	0.014	----	11.7810		----			
1.4363	3461	0.015	1.136	12.0035	12:0	0.12	ECL deviates  0.004	Reference -0.004	
1.4943	2030	0.016	----	12.2129		----			
1.5207	467	0.011	----	12.3078		----			
1.5589	1771	0.018	----	12.4447		----			
1.6045	3659	0.013	1.095	12.6085	13:0 iso	0.13	ECL deviates -0.004	Reference -0.011	
1.6330	2312	0.016	1.089	12.7108	13:0 anteiso	0.08	ECL deviates  0.001	Reference -0.005	
1.6895	1373	0.021	1.077	12.9139	13:1 w5c	0.05	ECL deviates -0.006		
1.7138	1131	0.013	1.073	13.0010	13:0	0.04	ECL deviates  0.001	Reference -0.006	
1.7804	778	0.018	----	13.1881	12:0 2OH	----	ECL deviates  0.002		
1.8720	1702	0.020	----	13.4436		----			
1.9317	45428	0.013	1.043	13.6102	14:0 iso	1.50	ECL deviates -0.004	Reference -0.010	
1.9713	1051	0.012	1.039	13.7206	14:0 anteiso	0.03	ECL deviates  0.005	Reference -0.001	
2.0063	2605	0.019	----	13.8183		----			
2.0393	2534	0.012	1.031	13.9104	14:1 w5c	0.08	ECL deviates -0.001		
2.0710	35445	0.014	1.028	13.9988	14:0	1.15	ECL deviates -0.001	Reference -0.007	
2.0968	634	0.010	----	14.0584		----			
2.1264	1005	0.013	----	14.1253	14:0 iso 3OH	----	ECL deviates  0.000		
2.1541	2881	0.024	----	14.1877		----			
2.2170	1997	0.021	----	14.3298		----			
2.2644	35178	0.018	1.013	14.4368	15:1 iso w6c	1.13	ECL deviates -0.002		
2.2827	8374	0.013	1.011	14.4781	15:4 w3c	0.27	ECL deviates -0.012		
2.3045	9119	0.014	1.010	14.5274	15:1 anteiso w9c	0.29	ECL deviates -0.003		
2.3435	197861	0.014	1.008	14.6156	15:0 iso	6.32	ECL deviates -0.001	Reference -0.007	
2.3852	147882	0.014	1.005	14.7097	15:0 anteiso	4.71	ECL deviates -0.001	Reference -0.007	
2.4499	6923	0.024	1.001	14.8559	15:1 w6c	0.22	ECL deviates -0.004		
2.5134	19182	0.015	0.998	14.9994	15:0	0.61	ECL deviates -0.001	Reference -0.006	
2.5418	9652	0.018	----	15.0541		----			
2.6039	2667	0.018	----	15.1721		----			
2.6354	2276	0.020	----	15.2318		----			
2.7190	6397	0.016	0.990	15.3905	16:1 w7c alcohol	0.20	ECL deviates -0.006		
2.7464	31984	0.021	0.989	15.4426	15:0 DMA	1.00	ECL deviates -0.008		
2.8059	85851	0.016	0.987	15.5556	16:0 N alcohol	2.68	ECL deviates -0.001		
2.8386	77465	0.015	0.986	15.6178	16:0 iso	2.42	ECL deviates -0.002	Reference -0.007	
2.8910	9391	0.014	0.984	15.7172	16:0 anteiso	0.29	ECL deviates  0.002	Reference -0.003	
2.9165	43202	0.016	0.983	15.7656	16:1 w9c	1.35	ECL deviates -0.009		
2.9458	368095	0.017	0.983	15.8214	16:1 w7c	11.46	ECL deviates -0.003		
2.9931	112504	0.016	0.981	15.9111	16:1 w5c	3.50	ECL deviates  0.000		
3.0414	356234	0.016	0.980	16.0028	16:0	11.07	ECL deviates  0.003	Reference -0.002	
3.0691	22557	0.020	----	16.0495		----			
3.1204	1957	0.015	0.979	16.1354	16:2 DMA	0.06	ECL deviates -0.003		
3.1571	4893	0.021	----	16.1967		----			
3.1928	3970	0.020	----	16.2564		----			
3.2256	2164	0.020	0.977	16.3114	16:1 w7c DMA	0.07	ECL deviates  0.001		
3.2917	225019	0.019	0.976	16.4220	16:0 10-methyl	6.96	ECL deviates  0.002		
3.3258	49459	0.018	----	16.4791		----			
3.3544	23072	0.019	----	16.5270		----			
3.4097	51450	0.016	0.974	16.6194	17:0 iso	1.59	ECL deviates -0.004	Reference -0.009	
3.4670	55357	0.018	0.973	16.7154	17:0 anteiso	1.71	ECL deviates -0.005		
3.5116	30129	0.018	0.973	16.7900	17:1 w8c	0.93	ECL deviates -0.007		
3.5715	120607	0.019	0.972	16.8903	17:0 cyclo w7c	3.72	ECL deviates -0.003		
3.6356	13902	0.018	0.972	16.9976	17:0	0.43	ECL deviates -0.002	Reference -0.007	
3.6620	25663	0.017	0.971	17.0385	17:1 w7c 10-methyl	0.79	ECL deviates -0.005		
3.7047	5956	0.018	----	17.1035		----			
3.7407	1652	0.020	----	17.1584		----			
3.7907	1976	0.019	0.971	17.2348	16:0 2OH	0.06	ECL deviates -0.005		
3.8992	18128	0.020	0.970	17.4002	17:0 10-methyl	0.56	ECL deviates -0.007		
3.9369	1787	0.011	0.970	17.4577	17:0 DMA	0.05	ECL deviates  0.000		
3.9560	4743	0.021	----	17.4869		----			
4.0319	23299	0.029	----	17.6026		----			
4.1057	61418	0.016	0.970	17.7151	18:2 w6c	1.89	ECL deviates -0.012		
4.1408	206653	0.020	0.970	17.7687	18:1 w9c	6.35	ECL deviates -0.006		
4.1764	341626	0.017	0.969	17.8229	18:1 w7c	10.50	ECL deviates -0.004		
4.2349	40180	0.021	----	17.9122		----			
4.2908	56094	0.018	0.969	17.9974	18:0	1.72	ECL deviates -0.003	Reference -0.007	
4.3457	18833	0.018	0.969	18.0771	18:1 w7c 10-methyl	0.58	ECL deviates -0.008		
4.3991	3326	0.015	0.969	18.1543	18:2 DMA	0.10	ECL deviates -0.006		
4.4153	3868	0.017	----	18.1778		----			
4.4485	3435	0.019	0.969	18.2257	18:1 w9c DMA	0.11	ECL deviates -0.011		
4.4809	1247	0.014	0.970	18.2725	18:1 w7c DMA	0.04	ECL deviates -0.010		
4.5078	1298	0.016	----	18.3114		----			
4.5591	82203	0.021	0.970	18.3855	18:0 10-methyl	2.53	ECL deviates -0.009		
4.6282	2543	0.021	0.970	18.4854	19:4 w6c	0.08	ECL deviates  0.000		
4.6723	7988	0.023	0.970	18.5491	19:3 w6c	0.25	ECL deviates -0.011		
4.7285	2103	0.016	0.970	18.6304	19:0 iso	0.06	ECL deviates  0.000		
4.7403	1379	0.012	----	18.6474		----			
4.8038	11669	0.023	----	18.7391		----		Reference  0.007	
4.8506	10699	0.020	0.970	18.8068	19:1 w8c	0.33	ECL deviates -0.004		
4.9128	106083	0.019	0.970	18.8966	19:0 cyclo w7c	3.26	ECL deviates -0.013		
4.9834	78615	0.018	----	18.9987	19:0	----	ECL deviates -0.001		
5.0451	1564	0.019	----	19.0848		----			
5.0845	850	0.016	----	19.1398		----			
5.1380	3051	0.023	----	19.2144		----			
5.1717	6452	0.021	----	19.2614		----			
5.2129	2519	0.016	0.971	19.3189	19:0 cyclo 9,10 DMA	0.08	ECL deviates -0.005		
5.2582	17192	0.025	----	19.3820		----			
5.3120	8712	0.019	----	19.4570		----			
5.3453	1601	0.015	0.971	19.5034	20:5 w3c	0.05	ECL deviates  0.021		
5.3771	5413	0.021	----	19.5477		----			
5.4103	7658	0.024	----	19.5940		----			
5.5290	22380	0.026	----	19.7595		----			
5.5605	8859	0.023	0.972	19.8034	20:1 w8c	0.27	ECL deviates -0.010		
5.6030	741	0.015	----	19.8627		----			
5.6979	17677	0.022	0.972	19.9950	20:0	0.54	ECL deviates -0.005	Reference -0.010	
5.8011	2960	0.023	----	20.1379		----			
5.8310	5126	0.020	----	20.1794		----			
5.9429	6872	0.020	----	20.3344		----			
5.9738	33982	0.025	----	20.3771		----			
6.0488	886	0.016	----	20.4810		----			
6.0971	2680	0.030	----	20.5479		----			
6.1464	8295	0.029	----	20.6162		----			
6.2102	4038	0.029	----	20.7046		----			
6.2743	10249	0.020	0.971	20.7934	21:1 w8c	0.32	ECL deviates -0.005		
6.3323	6659	0.026	----	20.8737		----			
6.3902	21292	0.022	0.970	20.9539	21:1 w3c	0.65	ECL deviates  0.000		
6.4258	5819	0.023	0.970	21.0032	21:0	0.18	ECL deviates  0.003	Reference -0.002	
6.5057	3952	0.022	----	21.1135		----			
6.5928	4175	0.027	0.969	21.2337	22:5 w6c	0.13	ECL deviates -0.018		
6.6238	4009	0.018	----	21.2765		----			
6.6502	1112	0.012	0.969	21.3131	22:6 w3c	0.03	ECL deviates -0.019		
6.6855	736	0.018	----	21.3617		----			
6.7499	1181	0.027	0.968	21.4506	22:5 w3c	0.04	ECL deviates -0.017		
6.8727	9876	0.035	0.967	21.6202	22:0 iso	----	> max ar/ht		
6.9497	3100	0.022	0.966	21.7265	22:2 w6c	0.09	ECL deviates -0.012		
6.9832	2688	0.021	0.965	21.7728	22:1 w9c	0.08	ECL deviates  0.000		
7.0181	4849	0.026	0.965	21.8209	22:1 w8c	0.15	ECL deviates  0.007		
7.1048	5840	0.024	0.964	21.9406	22:1 w3c	0.18	ECL deviates -0.006		
7.1467	18142	0.020	0.963	21.9984	22:0	0.55	ECL deviates -0.002	Reference -0.008	
7.2102	4824	0.034	----	22.0873		----			
7.3227	12950	0.021	----	22.2447		----			
7.3748	2620	0.038	----	22.3176		----	> max ar/ht		
7.4363	2175	0.028	----	22.4037		----			
7.4928	1417	0.023	0.957	22.4828	23:4 w6c	0.04	ECL deviates  0.012		
7.5328	1011	0.021	----	22.5387		----			
7.6073	2536	0.032	0.954	22.6431	23:3 w3c	0.08	ECL deviates -0.002		
7.6419	806	0.017	----	22.6914		----			
7.7016	3253	0.022	----	22.7750		----			
7.7612	2118	0.026	----	22.8585		----			
7.8034	10940	0.021	0.949	22.9174	23:1 w4c	0.33	ECL deviates -0.009		
7.8621	4253	0.019	0.947	22.9996	23:0	0.13	ECL deviates  0.000	Reference -0.008	
7.9076	1980	0.027	----	23.0640		----			
8.0696	4434	0.020	----	23.2933		----			
8.3219	6082	0.030	0.931	23.6505	24:3 w3c	0.18	ECL deviates -0.004		
8.3795	2520	0.022	----	23.7321		----			
8.4106	3117	0.025	----	23.7762		----			
8.4845	2065	0.023	----	23.8809		----			
8.5211	1151	0.021	----	23.9327		----			
8.5683	15375	0.020	0.920	23.9995	24:0	0.45	ECL deviates  0.000	Reference -0.009	
8.6726	714	0.017	----	24.1472		----	> max rt		
8.7622	2023	0.037	----	24.2741		----	> max rt		
8.9250	16659	0.021	----	24.5047		----	> max rt		
9.2256	18703	0.022	----	24.9304		----	> max rt		
9.4648	7401	0.020	----	25.2693		----	> max rt		

ECL Deviation: 0.007                            Reference ECL Shift: 0.007       Number Reference Peaks: 22
Total Response: 3654597                       Total Named: 3215209
Percent Named: 87.98%                         Total Amount: 3165138
Profile Comment:   Review report comments.

(No search libraries specified in method PLFAD1.)
