# Supplementary material for: Microbial regulation of soil carbon properties under nitrogen addition and plant inputs removal
Source: PeerJ. 2019 Jul 17;7:e7343. doi: 10.7717/peerj.7343 (PMC6642627; doi:10.7717/peerj.7343)
Supplement: File S1 — The raw data showed the soil microbial PLFAs files in the year of 2015 and 2016. Each file of rtf. represented the microbial PLFAs for each soil sample. In the Supplemental File, the Excel file named “Numbers” showed the plots names and the related rtf. file names. [file peerj-07-7343-s002.zip › supplementary files/2015/52.rtf]

Volume: DATA            File: E164216.88A        Samp Ctr: 10                ID Number: 29349 
Type: Samp                   Bottle: 21                      Method: PLFAD1 
Created: 4/21/2016 7:05:10 PM 
Sample ID: 52 


RT	Response	Ar/Ht	RFact	ECL	Peak Name	Percent	Comment1	Comment2	
0.7134	1.915E+9	0.015	----	7.6465	SOLVENT PEAK	----	< min rt		
0.8843	778	0.010	----	8.7620		----	< min rt		
1.1858	1506	0.012	----	10.7348		----			
1.2617	597	0.011	----	11.1708		----			
1.3530	812	0.016	1.166	11.6064	12:0 iso	0.03	ECL deviates -0.006		
1.3641	365	0.009	----	11.6595		----			
1.3890	1345	0.014	----	11.7783		----			
1.4362	3083	0.015	1.136	12.0040	12:0	0.11	ECL deviates  0.004	Reference -0.004	
1.4935	2150	0.017	----	12.2108		----			
1.5584	1454	0.020	----	12.4439		----			
1.6040	3412	0.013	1.095	12.6076	13:0 iso	0.12	ECL deviates -0.005	Reference -0.012	
1.6328	2373	0.014	1.089	12.7110	13:0 anteiso	0.08	ECL deviates  0.002	Reference -0.006	
1.6899	1251	0.018	1.077	12.9160	13:1 w5c	0.04	ECL deviates -0.004		
1.7135	1361	0.014	1.073	13.0006	13:0	0.05	ECL deviates  0.001	Reference -0.007	
1.7809	822	0.018	----	13.1901	12:0 2OH	----	ECL deviates  0.004		
1.8731	1708	0.021	----	13.4474		----			
1.9315	42623	0.014	1.043	13.6103	14:0 iso	1.43	ECL deviates -0.004	Reference -0.011	
1.9717	1060	0.012	1.039	13.7227	14:0 anteiso	0.04	ECL deviates  0.007	Reference  0.000	
1.9918	1011	0.010	1.036	13.7786	14:1 w9c	0.03	ECL deviates  0.001		
2.0051	1735	0.012	----	13.8157		----			
2.0712	38998	0.015	1.028	14.0002	14:0	1.29	ECL deviates  0.000	Reference -0.006	
2.0985	714	0.012	----	14.0632		----			
2.1257	1044	0.015	----	14.1246	14:0 iso 3OH	----	ECL deviates  0.000		
2.1528	2994	0.025	----	14.1857		----			
2.2182	2304	0.020	----	14.3336		----			
2.2643	39853	0.018	1.013	14.4376	15:1 iso w6c	1.30	ECL deviates -0.001		
2.2822	8732	0.012	1.011	14.4781	15:4 w3c	0.28	ECL deviates -0.012		
2.3040	10383	0.014	1.010	14.5273	15:1 anteiso w9c	0.34	ECL deviates -0.003		
2.3422	190553	0.014	1.008	14.6137	15:0 iso	6.17	ECL deviates -0.003	Reference -0.010	
2.3844	154801	0.015	1.005	14.7090	15:0 anteiso	5.00	ECL deviates -0.002	Reference -0.008	
2.4490	7497	0.025	1.001	14.8550	15:1 w6c	0.24	ECL deviates -0.005		
2.5132	19701	0.015	0.998	14.9999	15:0	0.63	ECL deviates  0.000	Reference -0.006	
2.5411	8083	0.017	----	15.0539		----			
2.6028	2373	0.020	----	15.1710		----			
2.6336	2634	0.021	----	15.2297		----			
2.7195	6528	0.015	0.990	15.3927	16:1 w7c alcohol	0.21	ECL deviates -0.004		
2.7450	34109	0.022	0.989	15.4412	15:0 DMA	1.08	ECL deviates -0.009		
2.8053	70031	0.016	0.987	15.5556	16:0 N alcohol	2.22	ECL deviates -0.001		
2.8376	80067	0.017	0.986	15.6171	16:0 iso	2.53	ECL deviates -0.003	Reference -0.009	
2.8899	7331	0.013	0.984	15.7163	16:0 anteiso	0.23	ECL deviates  0.001	Reference -0.005	
2.9166	49201	0.018	0.983	15.7670	16:1 w9c	1.55	ECL deviates -0.008		
2.9449	345839	0.017	0.983	15.8208	16:1 w7c	10.91	ECL deviates -0.004		
2.9917	102296	0.017	0.981	15.9098	16:1 w5c	3.22	ECL deviates -0.001		
3.0402	335168	0.015	0.980	16.0019	16:0	10.55	ECL deviates  0.002	Reference -0.004	
3.0677	13032	0.014	----	16.0484		----			
3.0833	6539	0.012	----	16.0744		----			
3.1198	2484	0.015	0.979	16.1355	16:2 DMA	0.08	ECL deviates -0.002		
3.1558	5576	0.021	----	16.1958		----			
3.1914	3213	0.017	----	16.2554		----			
3.2284	2279	0.021	0.977	16.3174	16:1 w7c DMA	0.07	ECL deviates  0.007		
3.2898	204059	0.020	0.976	16.4201	16:0 10-methyl	6.39	ECL deviates  0.000		
3.3251	41743	0.018	----	16.4792		----			
3.3531	24565	0.019	----	16.5262		----			
3.4095	47004	0.016	0.974	16.6204	17:0 iso	1.47	ECL deviates -0.003	Reference -0.009	
3.4661	53287	0.017	0.973	16.7153	17:0 anteiso	1.67	ECL deviates -0.005		
3.5100	32785	0.017	0.973	16.7887	17:1 w8c	1.02	ECL deviates -0.008		
3.5694	115491	0.019	0.972	16.8882	17:0 cyclo w7c	3.61	ECL deviates -0.005		
3.6348	13592	0.018	0.972	16.9977	17:0	0.42	ECL deviates -0.002	Reference -0.008	
3.6597	21998	0.017	0.971	17.0365	17:1 w7c 10-methyl	0.69	ECL deviates -0.007		
3.7030	5373	0.017	----	17.1024		----			
3.7393	1535	0.019	----	17.1577		----			
3.7878	2376	0.019	0.971	17.2317	16:0 2OH	0.07	ECL deviates -0.008		
3.8982	20161	0.016	0.970	17.4002	17:0 10-methyl	0.63	ECL deviates -0.007		
3.9350	2071	0.012	0.970	17.4563	17:0 DMA	0.06	ECL deviates -0.002		
3.9566	6430	0.022	----	17.4893		----			
4.0121	6371	0.015	0.970	17.5739	18:3 w6c	0.20	ECL deviates -0.006		
4.0311	20544	0.025	----	17.6028		----			
4.1052	63122	0.017	0.970	17.7160	18:2 w6c	1.97	ECL deviates -0.011		
4.1383	220958	0.020	0.970	17.7664	18:1 w9c	6.88	ECL deviates -0.008		
4.1747	358139	0.018	0.969	17.8220	18:1 w7c	11.15	ECL deviates -0.005		
4.2340	38232	0.021	----	17.9124		----			
4.2893	53088	0.018	0.969	17.9967	18:0	1.65	ECL deviates -0.003	Reference -0.009	
4.3448	19539	0.018	0.969	18.0775	18:1 w7c 10-methyl	0.61	ECL deviates -0.007		
4.3984	7073	0.028	0.969	18.1549	18:2 DMA	0.22	ECL deviates -0.005		
4.4474	3662	0.023	0.969	18.2258	18:1 w9c DMA	0.11	ECL deviates -0.011		
4.4779	1236	0.013	0.970	18.2699	18:1 w7c DMA	0.04	ECL deviates -0.013		
4.5047	1753	0.016	----	18.3085		----			
4.5579	93955	0.020	0.970	18.3854	18:0 10-methyl	2.93	ECL deviates -0.010		
4.6297	2627	0.021	0.970	18.4892	19:4 w6c	0.08	ECL deviates  0.004		
4.6716	6797	0.025	0.970	18.5499	19:3 w6c	0.21	ECL deviates -0.010		
4.7255	1573	0.012	0.970	18.6277	19:0 iso	0.05	ECL deviates -0.002		
4.7389	2589	0.018	----	18.6470		----			
4.8027	11450	0.021	----	18.7393		----		Reference  0.006	
4.8486	12047	0.020	0.970	18.8056	19:1 w8c	0.38	ECL deviates -0.005		
4.8858	13015	0.015	0.970	18.8594	19:1 w6c	0.41	ECL deviates  0.008		
4.9115	87567	0.019	0.970	18.8966	19:0 cyclo w7c	2.73	ECL deviates -0.013		
4.9825	80369	0.020	----	18.9992	19:0	----	ECL deviates -0.001		
5.0416	1972	0.018	----	19.0818		----			
5.1368	2801	0.025	----	19.2146		----			
5.1696	5817	0.017	----	19.2602		----			
5.2561	21405	0.029	----	19.3808		----			
5.3109	8755	0.020	0.971	19.4574	20:5 w3c	----	Below has same name		
5.3437	1910	0.016	----	19.5030	20:5 w3c	----	Above has same name		
5.3768	6000	0.020	----	19.5492		----			
5.4086	8591	0.026	----	19.5936		----			
5.5276	21370	0.028	----	19.7594		----			
5.5577	10494	0.023	0.972	19.8014	20:1 w8c	0.33	ECL deviates -0.012		
5.6976	18176	0.021	0.972	19.9965	20:0	0.57	ECL deviates -0.004	Reference -0.010	
5.8010	2504	0.018	----	20.1398		----			
5.8300	5385	0.018	----	20.1800		----			
5.9728	35693	0.028	----	20.3778		----			
6.0459	741	0.015	----	20.4790		----			
6.0999	3204	0.028	----	20.5538		----			
6.1449	5129	0.022	----	20.6162		----			
6.1682	2345	0.014	0.971	20.6485	21:3 w3c	0.07	ECL deviates -0.005		
6.2047	3851	0.030	----	20.6990		----			
6.2734	11896	0.019	0.971	20.7943	21:1 w8c	0.37	ECL deviates -0.004		
6.3304	6343	0.026	----	20.8732		----			
6.3897	20263	0.019	0.970	20.9553	21:1 w3c	0.63	ECL deviates  0.001		
6.4232	5851	0.023	0.970	21.0018	21:0	0.18	ECL deviates  0.002	Reference -0.006	
6.5035	3585	0.022	----	21.1127		----			
6.5883	4417	0.030	----	21.2297		----			
6.6232	5369	0.026	----	21.2779		----			
6.6861	675	0.016	----	21.3648		----			
6.8723	9160	0.032	0.967	21.6219	22:0 iso	0.28	ECL deviates  0.004		
6.9495	2096	0.021	0.966	21.7285	22:2 w6c	0.07	ECL deviates -0.010		
6.9842	2209	0.019	0.965	21.7765	22:1 w9c	0.07	ECL deviates  0.004		
7.0164	3229	0.022	0.965	21.8209	22:1 w8c	0.10	ECL deviates  0.007		
7.1009	5458	0.018	0.964	21.9375	22:1 w3c	0.17	ECL deviates -0.009		
7.1458	16363	0.020	0.963	21.9996	22:0	0.51	ECL deviates  0.000	Reference -0.009	
7.2076	1199	0.021	----	22.0860		----			
7.3187	8772	0.018	----	22.2414		----			
7.4385	901	0.023	----	22.4092		----			
7.6028	2441	0.037	0.954	22.6391	23:3 w3c	----	> max ar/ht		
7.6976	3194	0.021	----	22.7718		----			
7.7605	1648	0.024	----	22.8599		----			
7.8029	10187	0.021	0.949	22.9193	23:1 w4c	0.31	ECL deviates -0.007		
7.8615	4319	0.019	0.947	23.0012	23:0	0.13	ECL deviates  0.001	Reference -0.009	
7.9098	1639	0.026	----	23.0696		----			
8.0669	4609	0.019	----	23.2922		----			
8.3176	5112	0.024	0.931	23.6472	24:3 w3c	0.15	ECL deviates -0.008		
8.3746	1595	0.020	----	23.7279		----			
8.4125	2080	0.019	0.927	23.7816	24:1 w9c	0.06	ECL deviates -0.005		
8.4862	1312	0.021	----	23.8860		----			
8.5651	17023	0.019	0.920	23.9977	24:0	0.50	ECL deviates -0.002	Reference -0.014	
8.6704	695	0.017	----	24.1469		----	> max rt		
8.7662	1777	0.040	----	24.2826		----	> max rt		
8.9219	9778	0.018	----	24.5032		----	> max rt		
9.2226	20172	0.022	----	24.9292		----	> max rt		
9.4619	8109	0.022	----	25.2682		----	> max rt		

ECL Deviation: 0.007                            Reference ECL Shift: 0.008       Number Reference Peaks: 22
Total Response: 3568052                       Total Named: 3172692
Percent Named: 88.92%                         Total Amount: 3124269
Profile Comment:   Review report comments.

(No search libraries specified in method PLFAD1.)
