# Supplementary material for: Microbial regulation of soil carbon properties under nitrogen addition and plant inputs removal
Source: PeerJ. 2019 Jul 17;7:e7343. doi: 10.7717/peerj.7343 (PMC6642627; doi:10.7717/peerj.7343)
Supplement: File S1 — The raw data showed the soil microbial PLFAs files in the year of 2015 and 2016. Each file of rtf. represented the microbial PLFAs for each soil sample. In the Supplemental File, the Excel file named “Numbers” showed the plots names and the related rtf. file names. [file peerj-07-7343-s002.zip › supplementary files/2015/53.rtf]

Volume: DATA            File: E164216.88A        Samp Ctr: 11                ID Number: 29350 
Type: Samp                   Bottle: 22                      Method: PLFAD1 
Created: 4/21/2016 7:24:37 PM 
Sample ID: 53 


RT	Response	Ar/Ht	RFact	ECL	Peak Name	Percent	Comment1	Comment2	
0.7139	1.903E+9	0.020	----	7.6393	SOLVENT PEAK	----	< min rt		
0.8851	1120	0.010	----	8.7593		----	< min rt		
1.0456	1009	0.013	----	9.8090		----	< min rt		
1.0605	2224	0.014	----	9.9068		----	< min rt		
1.1853	2813	0.013	1.250	10.7228	11:0 anteiso	0.07	ECL deviates  0.018	Reference  0.017	
1.2046	664	0.011	----	10.8488		----			
1.2235	606	0.011	----	10.9725		----			
1.2609	1318	0.011	----	11.1591		----			
1.2770	2747	0.012	----	11.2366		----			
1.3171	1420	0.015	1.181	11.4283	10:0 3OH	0.03	ECL deviates -0.013		
1.3517	1722	0.016	----	11.5940	Phthalate 1	----	ECL deviates  0.007		
1.3644	837	0.010	----	11.6549		----			
1.3885	2928	0.015	----	11.7702		----			
1.4353	6119	0.016	1.136	11.9942	12:0	0.13	ECL deviates -0.006	Reference -0.009	
1.4667	665	0.011	----	12.1095	11:0 iso 3OH	----	ECL deviates -0.003		
1.4929	3631	0.015	----	12.2036		----			
1.5196	948	0.013	----	12.2997		----			
1.5582	2539	0.020	----	12.4387		----			
1.6036	5695	0.013	1.095	12.6021	13:0 iso	0.12	ECL deviates -0.010	Reference -0.014	
1.6318	4027	0.017	1.089	12.7038	13:0 anteiso	0.08	ECL deviates -0.006	Reference -0.010	
1.6886	1814	0.016	----	12.9079		----			
1.7127	2230	0.014	1.073	12.9947	13:0	0.05	ECL deviates -0.005	Reference -0.010	
1.7789	934	0.017	----	13.1818	12:0 2OH	----	ECL deviates -0.004		
1.8721	2671	0.020	----	13.4423		----			
1.9315	69177	0.014	1.043	13.6084	14:0 iso	1.38	ECL deviates -0.006	Reference -0.011	
1.9711	1475	0.013	1.039	13.7190	14:0 anteiso	0.03	ECL deviates  0.003	Reference -0.002	
1.9920	1733	0.011	1.036	13.7776	14:1 w9c	0.03	ECL deviates  0.000		
2.0057	2671	0.014	----	13.8158		----			
2.0710	69456	0.015	1.028	13.9985	14:0	1.37	ECL deviates -0.002	Reference -0.007	
2.0984	812	0.013	----	14.0618		----			
2.1266	1622	0.014	----	14.1256	14:0 iso 3OH	----	ECL deviates  0.001		
2.1522	4494	0.026	----	14.1834		----			
2.2180	3205	0.021	----	14.3323		----			
2.2646	87153	0.020	1.013	14.4377	15:1 iso w6c	1.69	ECL deviates -0.001		
2.3050	17484	0.015	1.010	14.5290	15:1 anteiso w9c	0.34	ECL deviates -0.001		
2.3442	294748	0.014	1.008	14.6178	15:0 iso	5.69	ECL deviates  0.001	Reference -0.005	
2.3854	220303	0.014	1.005	14.7110	15:0 anteiso	4.25	ECL deviates  0.000	Reference -0.006	
2.4498	11739	0.025	1.001	14.8567	15:1 w6c	0.23	ECL deviates -0.003		
2.5146	33931	0.015	0.998	15.0032	15:0	0.65	ECL deviates  0.003	Reference -0.003	
2.5424	12305	0.019	----	15.0565		----			
2.6047	1991	0.017	----	15.1749		----			
2.6345	4975	0.019	----	15.2316		----			
2.7203	8691	0.014	0.990	15.3948	16:1 w7c alcohol	0.16	ECL deviates -0.002		
2.7467	48265	0.021	0.989	15.4450	15:0 DMA	0.91	ECL deviates -0.006		
2.8070	111390	0.016	0.987	15.5596	16:0 N alcohol	2.11	ECL deviates  0.003		
2.8397	119454	0.017	0.986	15.6218	16:0 iso	2.26	ECL deviates  0.002	Reference -0.005	
2.9175	81329	0.019	0.983	15.7696	16:1 w9c	1.53	ECL deviates -0.005		
2.9483	562441	0.018	0.983	15.8282	16:1 w7c	10.59	Column Overload		
2.9943	167070	0.017	0.981	15.9158	16:1 w5c	3.14	ECL deviates  0.005		
3.0444	592885	0.015	0.980	16.0104	16:0	11.14	Column Overload		
3.0708	24226	0.019	----	16.0547		----			
3.1233	4076	0.017	0.979	16.1425	16:2 DMA	0.08	ECL deviates  0.005		
3.1590	9461	0.021	----	16.2024		----			
3.1937	4040	0.017	----	16.2605		----			
3.2309	3125	0.021	0.977	16.3228	16:1 w7c DMA	0.06	ECL deviates  0.013		
3.2923	274881	0.021	0.976	16.4257	16:0 10-methyl	5.14	ECL deviates  0.006		
3.3280	62411	0.019	0.975	16.4855	17:1 iso w9c	1.17	ECL deviates -0.013		
3.3551	34458	0.019	0.975	16.5308	17:1 anteiso w9c	0.64	ECL deviates -0.005		
3.4115	69652	0.016	0.974	16.6253	17:0 iso	1.30	ECL deviates  0.002	Reference -0.006	
3.4686	82043	0.017	0.973	16.7210	17:0 anteiso	1.53	ECL deviates  0.001		
3.5127	54143	0.018	0.973	16.7948	17:1 w8c	1.01	ECL deviates -0.002		
3.5727	175011	0.018	0.972	16.8954	17:0 cyclo w7c	3.26	ECL deviates  0.002		
3.6370	25770	0.018	0.972	17.0030	17:0	0.48	ECL deviates  0.003	Reference -0.005	
3.6629	25551	0.018	0.971	17.0429	17:1 w7c 10-methyl	0.48	ECL deviates  0.000		
3.7046	7629	0.018	----	17.1066		----			
3.7404	2408	0.019	----	17.1613		----			
3.7908	4743	0.019	0.971	17.2381	16:0 2OH	0.09	ECL deviates -0.002		
3.8430	623	0.013	----	17.3178		----			
3.9012	33064	0.017	0.970	17.4065	17:0 10-methyl	0.61	ECL deviates -0.001		
3.9362	2630	0.011	0.970	17.4600	17:0 DMA	0.05	ECL deviates  0.002		
3.9589	12789	0.023	----	17.4945		----			
4.0143	11225	0.011	0.970	17.5791	18:3 w6c	0.21	ECL deviates -0.001		
4.0326	35714	0.024	0.970	17.6071	18:0 iso	0.66	ECL deviates -0.019		
4.1088	188428	0.018	0.970	17.7233	18:2 w6c	3.50	ECL deviates -0.004		
4.1440	340423	0.019	0.970	17.7769	18:1 w9c	6.33	ECL deviates  0.002		
4.1807	601200	0.017	0.969	17.8329	18:1 w7c	11.17	Column Overload		
4.2319	82292	0.020	----	17.9110		----			
4.2924	91215	0.017	0.969	18.0033	18:0	1.70	ECL deviates  0.003	Reference -0.005	
4.3471	38055	0.019	0.969	18.0826	18:1 w7c 10-methyl	0.71	ECL deviates -0.002		
4.3983	11737	0.025	0.969	18.1566	18:2 DMA	0.22	ECL deviates -0.003		
4.4445	7894	0.022	0.969	18.2234	18:1 w9c DMA	0.15	ECL deviates -0.014		
4.4818	2603	0.017	0.970	18.2773	18:1 w7c DMA	0.05	ECL deviates -0.005		
4.5072	2822	0.018	----	18.3140		----			
4.5599	130838	0.019	0.970	18.3902	18:0 10-methyl	2.43	ECL deviates -0.005		
4.6265	3461	0.020	0.970	18.4864	19:4 w6c	0.06	ECL deviates  0.001		
4.6741	10247	0.024	0.970	18.5552	19:3 w6c	0.19	ECL deviates -0.005		
4.7462	8185	0.023	0.970	18.6594	19:3 w3c	0.15	ECL deviates  0.001		
4.8071	15053	0.022	----	18.7474		----			
4.8503	20768	0.020	0.970	18.8098	19:1 w8c	0.39	ECL deviates -0.001		
4.8868	23072	0.017	0.970	18.8625	19:0 cyclo w9c	0.43	ECL deviates -0.009		
4.9153	138651	0.018	0.970	18.9037	19:0 cyclo w7c	2.58	ECL deviates -0.006		
4.9849	103645	0.018	----	19.0043	19:0	----	ECL deviates  0.004		
5.0439	3483	0.019	----	19.0867		----			
5.1379	3653	0.019	----	19.2176		----			
5.1720	15092	0.019	----	19.2652		----			
5.2579	46427	0.029	----	19.3849		----			
5.3113	16655	0.021	0.971	19.4592	20:5 w3c	0.31	ECL deviates -0.023		
5.3484	3753	0.016	----	19.5109		----			
5.3770	8375	0.019	----	19.5508		----			
5.4098	14781	0.025	----	19.5965		----			
5.5299	31516	0.027	0.972	19.7638	20:1 w9c	0.59	ECL deviates -0.009		
5.5608	15576	0.024	0.972	19.8069	20:1 w8c	0.29	ECL deviates -0.006		
5.6983	31270	0.023	0.972	19.9985	20:0	0.58	ECL deviates -0.002	Reference -0.010	
5.7544	1665	0.018	----	20.0761		----			
5.7998	3253	0.016	----	20.1390		----			
5.8297	12933	0.022	----	20.1804		----			
5.9441	12240	0.028	----	20.3387		----			
5.9737	42017	0.023	----	20.3797		----			
6.0498	1578	0.017	----	20.4850		----			
6.1026	5175	0.027	----	20.5581		----			
6.1471	8225	0.022	----	20.6197		----			
6.1739	3799	0.015	0.971	20.6567	21:3 w3c	0.07	ECL deviates  0.003		
6.2087	6804	0.029	----	20.7049		----			
6.2756	17393	0.019	0.971	20.7975	21:1 w8c	0.32	ECL deviates  0.000		
6.3324	16281	0.023	----	20.8761		----			
6.3919	29950	0.022	0.970	20.9584	21:1 w3c	0.56	ECL deviates  0.004		
6.4255	9068	0.021	0.970	21.0049	21:0	0.17	ECL deviates  0.005	Reference -0.003	
6.5082	5327	0.021	----	21.1190		----			
6.5935	8688	0.024	0.969	21.2367	22:5 w6c	0.16	ECL deviates -0.015		
6.6236	10443	0.019	----	21.2781		----			
6.6476	3015	0.013	0.969	21.3112	22:6 w3c	0.06	ECL deviates -0.021		
6.6921	1048	0.015	----	21.3725		----			
6.7511	3094	0.022	0.968	21.4540	22:5 w3c	0.06	ECL deviates -0.014		
6.8750	15342	0.036	0.967	21.6248	22:0 iso	----	> max ar/ht		
6.9523	4208	0.027	0.966	21.7313	22:2 w6c	0.08	ECL deviates -0.007		
6.9894	3122	0.016	0.965	21.7825	22:1 w9c	0.06	ECL deviates  0.010		
7.0188	7056	0.027	0.965	21.8231	22:1 w8c	0.13	ECL deviates  0.010		
7.1031	8304	0.020	0.964	21.9393	22:1 w3c	0.15	ECL deviates -0.008		
7.1480	34030	0.020	0.963	22.0013	22:0	0.63	ECL deviates  0.001	Reference -0.006	
7.2066	851	0.016	----	22.0830		----			
7.3218	14868	0.020	----	22.2439		----			
7.3759	1856	0.028	----	22.3195		----			
7.4356	1665	0.025	----	22.4029		----			
7.4906	1262	0.020	0.957	22.4799	23:4 w6c	0.02	ECL deviates  0.009		
7.5324	1041	0.018	----	22.5382		----			
7.5996	5135	0.044	----	22.6322		----	> max ar/ht		
7.7008	7018	0.025	----	22.7735		----			
7.7628	2052	0.019	----	22.8602		----			
7.8039	14344	0.020	0.949	22.9176	23:1 w4c	0.26	ECL deviates -0.009		
7.8624	7986	0.021	0.947	22.9993	23:0	0.15	ECL deviates -0.001	Reference -0.007	
7.9082	2416	0.024	----	23.0639		----			
8.0697	10248	0.021	----	23.2923		----			
8.2819	1323	0.019	0.933	23.5921	24:3 w6c	0.02	ECL deviates  0.002		
8.3195	8502	0.026	----	23.6452		----			
8.3776	4906	0.026	----	23.7273		----			
8.4099	4586	0.025	----	23.7730		----			
8.4818	3209	0.025	----	23.8745		----			
8.5203	1307	0.018	----	23.9290		----			
8.5674	29036	0.021	0.920	23.9956	24:0	0.51	ECL deviates -0.004	Reference -0.010	
8.6699	1584	0.022	----	24.1404		----	> max rt		
8.7687	2929	0.043	----	24.2799		----	> max rt		
8.9242	12417	0.021	----	24.4997		----	> max rt		
9.2236	20185	0.019	----	24.9227		----	> max rt		
9.2533	2915	0.014	----	24.9647		----	> max rt		
9.4635	14040	0.021	----	25.2615		----	> max rt		

ECL Deviation: 0.007                            Reference ECL Shift: 0.008       Number Reference Peaks: 20
Total Response: 5837050                       Total Named: 5316998
Percent Named: 91.09%                         Total Amount: 5231052
Profile Comment:   Column Overload:  A peak's response is greater than 400000.0.  Dilute and re-run.

(No search libraries specified in method PLFAD1.)
