# Supplementary material for: Microbial regulation of soil carbon properties under nitrogen addition and plant inputs removal
Source: PeerJ. 2019 Jul 17;7:e7343. doi: 10.7717/peerj.7343 (PMC6642627; doi:10.7717/peerj.7343)
Supplement: File S1 — The raw data showed the soil microbial PLFAs files in the year of 2015 and 2016. Each file of rtf. represented the microbial PLFAs for each soil sample. In the Supplemental File, the Excel file named “Numbers” showed the plots names and the related rtf. file names. [file peerj-07-7343-s002.zip › supplementary files/2015/54.rtf]

Volume: DATA            File: E164216.88A        Samp Ctr: 12                ID Number: 29351 
Type: Samp                   Bottle: 23                      Method: PLFAD1 
Created: 4/21/2016 7:44:22 PM 
Sample ID: 54 


RT	Response	Ar/Ht	RFact	ECL	Peak Name	Percent	Comment1	Comment2	
0.7147	1.889E+9	0.014	----	7.6621	SOLVENT PEAK	----	< min rt		
0.8859	2347	0.014	----	8.7779		----	< min rt		
0.9455	836	0.011	----	9.1661		----	< min rt		
1.0453	446	0.011	----	9.8169		----	< min rt		
1.0743	1073	0.022	1.329	10.0060	10:0	0.02	ECL deviates  0.006	Reference -0.003	
1.1867	3500	0.013	----	10.7383		----			
1.2254	721	0.014	1.221	10.9906	11:0	0.01	ECL deviates -0.009	Reference -0.017	
1.2621	1149	0.014	----	11.1706		----			
1.3191	912	0.015	1.181	11.4427	10:0 3OH	0.02	ECL deviates  0.001		
1.3532	2069	0.017	1.166	11.6054	12:0 iso	0.04	ECL deviates -0.007		
1.3658	740	0.009	----	11.6656		----			
1.3904	3538	0.015	----	11.7829		----			
1.4104	495	0.007	----	11.8784		----			
1.4367	7263	0.015	1.136	12.0041	12:0	0.13	ECL deviates  0.004	Reference -0.002	
1.4688	414	0.007	----	12.1197	11:0 iso 3OH	----	ECL deviates  0.007		
1.4943	4417	0.015	----	12.2115		----			
1.5216	1012	0.012	----	12.3095		----			
1.5591	3350	0.020	----	12.4442		----			
1.6052	7311	0.013	1.095	12.6096	13:0 iso	0.13	ECL deviates -0.003	Reference -0.008	
1.6326	4608	0.018	1.089	12.7082	13:0 anteiso	0.08	ECL deviates -0.001	Reference -0.007	
1.6896	1993	0.017	1.077	12.9126	13:1 w5c	0.03	ECL deviates -0.007		
1.7144	3072	0.014	1.073	13.0018	13:0	0.05	ECL deviates  0.002	Reference -0.003	
1.7808	1281	0.018	----	13.1879	12:0 2OH	----	ECL deviates  0.002		
1.8246	475	0.010	----	13.3099		----			
1.8725	3208	0.019	----	13.4435		----			
1.9326	87945	0.013	1.043	13.6114	14:0 iso	1.45	ECL deviates -0.003	Reference -0.007	
1.9716	1970	0.013	1.039	13.7201	14:0 anteiso	0.03	ECL deviates  0.004	Reference -0.001	
1.9924	1878	0.011	1.036	13.7782	14:1 w9c	0.03	ECL deviates  0.001		
2.0072	3233	0.014	----	13.8196		----			
2.0721	81780	0.015	1.028	14.0006	14:0	1.33	ECL deviates  0.001	Reference -0.004	
2.0987	1256	0.013	----	14.0615		----			
2.1264	1751	0.015	----	14.1240	14:0 iso 3OH	----	ECL deviates -0.001		
2.1545	6103	0.027	----	14.1875		----			
2.2174	4778	0.021	----	14.3295		----			
2.2651	83479	0.017	1.013	14.4372	15:1 iso w6c	1.34	ECL deviates -0.002		
2.2830	15943	0.011	1.011	14.4778	15:4 w3c	0.25	ECL deviates -0.012		
2.3052	23703	0.015	1.010	14.5278	15:1 anteiso w9c	0.38	ECL deviates -0.002		
2.3451	389247	0.014	1.008	14.6179	15:0 iso	6.20	ECL deviates  0.001	Reference -0.003	
2.3862	305500	0.015	1.005	14.7109	15:0 anteiso	4.85	ECL deviates  0.000	Reference -0.004	
2.4507	15145	0.026	1.001	14.8564	15:1 w6c	0.24	ECL deviates -0.004		
2.5140	40171	0.015	0.998	14.9996	15:0	0.63	ECL deviates  0.000	Reference -0.004	
2.5422	14461	0.017	----	15.0538		----			
2.6040	4076	0.020	----	15.1712		----			
2.6355	5439	0.021	----	15.2309		----			
2.7198	12833	0.015	0.990	15.3911	16:1 w7c alcohol	0.20	ECL deviates -0.005		
2.7463	66556	0.022	0.989	15.4414	15:0 DMA	1.04	ECL deviates -0.009		
2.8066	113773	0.017	0.987	15.5560	16:0 N alcohol	1.77	ECL deviates -0.001		
2.8396	166537	0.016	0.986	15.6187	16:0 iso	2.59	ECL deviates -0.001	Reference -0.005	
2.8919	16707	0.016	0.984	15.7180	16:0 anteiso	0.26	ECL deviates  0.003	Reference -0.001	
2.9182	88094	0.017	0.983	15.7679	16:1 w9c	1.37	ECL deviates -0.007		
2.9478	705953	0.017	0.983	15.8241	16:1 w7c	10.96	Column Overload		
2.9941	226993	0.016	0.981	15.9121	16:1 w5c	3.52	ECL deviates  0.001		
3.0449	713730	0.016	0.980	16.0079	16:0	11.05	Column Overload		
3.0700	24647	0.015	----	16.0499		----			
3.0850	12411	0.013	----	16.0751		----			
3.1222	4862	0.017	0.979	16.1373	16:2 DMA	0.08	ECL deviates -0.001		
3.1570	10334	0.023	----	16.1957		----			
3.1925	6490	0.019	----	16.2551		----			
3.2283	3917	0.021	0.977	16.3150	16:1 w7c DMA	0.06	ECL deviates  0.005		
3.2922	396373	0.021	0.976	16.4219	16:0 10-methyl	6.11	ECL deviates  0.002		
3.3272	73727	0.017	----	16.4805		----			
3.3544	49455	0.019	----	16.5261		----			
3.4104	92115	0.016	0.974	16.6198	17:0 iso	1.42	ECL deviates -0.004	Reference -0.008	
3.4684	113003	0.017	0.973	16.7169	17:0 anteiso	1.74	ECL deviates -0.003		
3.5115	73265	0.019	0.973	16.7890	17:1 w8c	1.13	ECL deviates -0.008		
3.5724	235745	0.017	0.972	16.8911	17:0 cyclo w7c	3.62	ECL deviates -0.003		
3.6354	29980	0.018	0.972	16.9965	17:0	0.46	ECL deviates -0.003	Reference -0.007	
3.6608	40637	0.017	0.971	17.0358	17:1 w7c 10-methyl	0.62	ECL deviates -0.007		
3.7039	10055	0.018	----	17.1016		----			
3.7402	3133	0.019	----	17.1570		----			
3.7884	4586	0.017	0.971	17.2304	16:0 2OH	0.07	ECL deviates -0.010		
3.8419	901	0.014	----	17.3121		----			
3.8987	42622	0.018	0.970	17.3987	17:0 10-methyl	0.65	ECL deviates -0.008		
3.9346	4004	0.013	0.970	17.4536	17:0 DMA	0.06	ECL deviates -0.005		
3.9568	11978	0.023	----	17.4874		----			
4.0303	49735	0.030	----	17.5994		----			
4.1070	142154	0.016	0.970	17.7165	18:2 w6c	2.18	ECL deviates -0.011		
4.1435	466189	0.020	0.970	17.7722	18:1 w9c	7.14	Column Overload		
4.1802	697900	0.017	0.969	17.8282	18:1 w7c	10.69	Column Overload		
4.2326	89172	0.019	----	17.9081		----			
4.2905	111433	0.017	0.969	17.9965	18:0	1.71	ECL deviates -0.003	Reference -0.007	
4.3451	40424	0.018	0.969	18.0757	18:1 w7c 10-methyl	0.62	ECL deviates -0.009		
4.3988	8163	0.017	0.969	18.1534	18:2 DMA	----	Below has same name		
4.4130	6095	0.013	----	18.1739	18:2 DMA	----	Above has same name		
4.4480	8047	0.021	0.969	18.2244	18:1 w9c DMA	0.12	ECL deviates -0.013		
4.4800	2883	0.016	0.970	18.2708	18:1 w7c DMA	0.04	ECL deviates -0.012		
4.5070	3008	0.018	----	18.3097		----			
4.5594	184694	0.020	0.970	18.3854	18:0 10-methyl	2.83	ECL deviates -0.010		
4.6296	5001	0.020	0.970	18.4869	19:4 w6c	0.08	ECL deviates  0.002		
4.6722	14628	0.024	0.970	18.5485	19:3 w6c	0.22	ECL deviates -0.011		
4.7282	3370	0.014	0.970	18.6294	19:0 iso	0.05	ECL deviates -0.001		
4.7412	4539	0.017	----	18.6482		----			
4.8041	23301	0.022	----	18.7391		----		Reference  0.008	
4.8482	22843	0.019	0.970	18.8028	19:1 w8c	0.35	ECL deviates -0.008		
4.8890	27113	0.016	0.970	18.8618	19:1 w6c	0.42	ECL deviates  0.010		
4.9151	166845	0.019	0.970	18.8996	19:0 cyclo w7c	2.56	ECL deviates -0.010		
4.9833	105293	0.019	----	18.9982	19:0	----	ECL deviates -0.002		
5.0438	4131	0.018	----	19.0827		----			
5.0856	945	0.017	----	19.1411		----			
5.1358	7129	0.023	----	19.2109		----			
5.1691	13642	0.020	----	19.2574		----			
5.2121	5954	0.016	0.971	19.3174	19:0 cyclo 9,10 DMA	0.09	ECL deviates -0.006		
5.2568	43272	0.027	----	19.3797		----			
5.3101	17365	0.018	----	19.4540		----			
5.3462	3634	0.015	0.971	19.5044	20:5 w3c	0.06	ECL deviates  0.022		
5.3762	11760	0.021	----	19.5462		----			
5.4097	16821	0.026	----	19.5930		----			
5.5280	44613	0.027	----	19.7579		----			
5.5613	20391	0.026	0.972	19.8044	20:1 w8c	0.31	ECL deviates -0.009		
5.6454	1626	0.017	0.972	19.9217	20:1 w4c	0.02	ECL deviates -0.009		
5.6986	36203	0.020	0.972	19.9958	20:0	0.56	ECL deviates -0.004	Reference -0.009	
5.7527	2123	0.020	----	20.0708		----			
5.7974	5404	0.019	----	20.1327		----			
5.8306	12459	0.020	----	20.1788		----			
5.8735	723	0.009	----	20.2382		----			
5.9170	5648	0.017	----	20.2984		----			
5.9390	10143	0.018	----	20.3289		----			
5.9724	54081	0.024	----	20.3751		----			
6.0448	1998	0.017	----	20.4754		----			
6.0988	8165	0.031	----	20.5502		----			
6.1427	11173	0.022	----	20.6111		----			
6.1672	5759	0.017	0.971	20.6450	21:3 w3c	0.09	ECL deviates -0.009		
6.2092	9362	0.030	----	20.7033		----			
6.2726	22579	0.020	0.971	20.7911	21:1 w8c	0.35	ECL deviates -0.007		
6.3285	15368	0.026	----	20.8685		----			
6.3893	38228	0.020	0.970	20.9528	21:1 w3c	0.59	ECL deviates -0.001		
6.4242	12599	0.026	0.970	21.0011	21:0	0.19	ECL deviates  0.001	Reference -0.005	
6.5050	8496	0.025	----	21.1127		----			
6.5452	3400	0.019	----	21.1681		----			
6.5902	8490	0.026	0.969	21.2304	22:5 w6c	0.13	ECL deviates -0.022		
6.6235	13682	0.026	----	21.2764		----			
6.6884	3042	0.022	----	21.3659		----			
6.7517	4689	0.035	0.968	21.4533	22:5 w3c	----	> max ar/ht		
6.8147	2398	0.018	----	21.5403		----			
6.8721	24805	0.030	0.967	21.6196	22:0 iso	0.38	ECL deviates  0.002		
6.9484	6958	0.027	0.966	21.7249	22:2 w6c	0.11	ECL deviates -0.014		
6.9815	6045	0.019	0.965	21.7707	22:1 w9c	0.09	ECL deviates -0.002		
7.0183	14064	0.030	0.965	21.8215	22:1 w8c	0.21	ECL deviates  0.008		
7.1000	11757	0.021	0.964	21.9344	22:1 w3c	0.18	ECL deviates -0.013		
7.1478	39635	0.019	0.963	22.0004	22:0	0.60	ECL deviates  0.000	Reference -0.006	
7.2098	5051	0.026	----	22.0871		----			
7.2396	4562	0.028	----	22.1288		----			
7.3215	20007	0.024	----	22.2434		----			
7.3752	3283	0.021	----	22.3186		----			
7.3950	1891	0.014	----	22.3464		----			
7.4349	4430	0.028	----	22.4022		----			
7.4930	3511	0.025	0.957	22.4836	23:4 w6c	0.05	ECL deviates  0.013		
7.5325	3310	0.023	----	22.5388		----			
7.5979	10466	0.050	----	22.6304		----	> max ar/ht		
7.7013	10064	0.025	----	22.7751		----			
7.7613	7389	0.024	----	22.8592		----			
7.8038	17654	0.021	0.949	22.9187	23:1 w4c	0.26	ECL deviates -0.008		
7.8616	10557	0.021	0.947	22.9996	23:0	0.16	ECL deviates  0.000	Reference -0.009	
7.9081	4502	0.028	----	23.0654		----			
7.9645	1460	0.024	----	23.1452		----			
8.0701	10210	0.024	----	23.2948		----			
8.2796	1190	0.017	0.933	23.5915	24:3 w6c	0.02	ECL deviates  0.001		
8.3183	14771	0.024	----	23.6464		----			
8.3717	6552	0.023	----	23.7220		----			
8.4077	6079	0.023	----	23.7730		----			
8.4811	4563	0.026	----	23.8770		----			
8.5203	1897	0.020	----	23.9326		----			
8.5674	34700	0.020	0.920	23.9993	24:0	0.50	ECL deviates -0.001	Reference -0.010	
8.6701	1383	0.019	----	24.1447		----	> max rt		
8.7548	3495	0.037	----	24.2648		----	> max rt		
8.9211	9513	0.019	----	24.5003		----	> max rt		
9.0258	923	0.022	----	24.6486		----	> max rt		
9.1593	707	0.017	----	24.8379		----	> max rt		
9.2243	32160	0.025	----	24.9300		----	> max rt		
9.4613	15928	0.022	----	25.2657		----	> max rt		

ECL Deviation: 0.007                            Reference ECL Shift: 0.007       Number Reference Peaks: 23
Total Response: 7348819                       Total Named: 6447930
Percent Named: 87.74%                         Total Amount: 6341360
Profile Comment:   Column Overload:  A peak's response is greater than 400000.0.  Dilute and re-run.

(No search libraries specified in method PLFAD1.)
