# Supplementary material for: Microbial regulation of soil carbon properties under nitrogen addition and plant inputs removal
Source: PeerJ. 2019 Jul 17;7:e7343. doi: 10.7717/peerj.7343 (PMC6642627; doi:10.7717/peerj.7343)
Supplement: File S1 — The raw data showed the soil microbial PLFAs files in the year of 2015 and 2016. Each file of rtf. represented the microbial PLFAs for each soil sample. In the Supplemental File, the Excel file named “Numbers” showed the plots names and the related rtf. file names. [file peerj-07-7343-s002.zip › supplementary files/2015/55.rtf]

Volume: DATA            File: E164216.88A        Samp Ctr: 13                ID Number: 29352 
Type: Samp                   Bottle: 24                      Method: PLFAD1 
Created: 4/21/2016 8:03:51 PM 
Sample ID: 55 


RT	Response	Ar/Ht	RFact	ECL	Peak Name	Percent	Comment1	Comment2	
0.7140	1.91E+9	0.016	----	7.6544	SOLVENT PEAK	----	< min rt		
0.8854	805	0.010	----	8.7721		----	< min rt		
0.9446	1000	0.013	----	9.1582		----	< min rt		
0.9720	584	0.013	----	9.3371		----	< min rt		
1.0739	766	0.018	1.329	10.0016	10:0	0.02	ECL deviates  0.001	Reference -0.005	
1.1866	2796	0.013	----	10.7362		----			
1.2617	1620	0.016	----	11.1672		----			
1.3181	832	0.014	1.181	11.4366	10:0 3OH	0.02	ECL deviates -0.005		
1.3525	1419	0.015	1.166	11.6007	12:0 iso	0.04	ECL deviates -0.011		
1.3661	629	0.009	----	11.6659		----			
1.3901	2203	0.016	----	11.7807		----			
1.4368	5403	0.016	1.136	12.0038	12:0	0.14	ECL deviates  0.004	Reference -0.001	
1.4942	3294	0.017	----	12.2102		----			
1.5222	782	0.012	----	12.3109		----			
1.5590	2275	0.018	----	12.4432		----			
1.6050	4931	0.013	1.095	12.6083	13:0 iso	0.12	ECL deviates -0.004	Reference -0.009	
1.6324	3388	0.017	1.089	12.7069	13:0 anteiso	0.08	ECL deviates -0.003	Reference -0.007	
1.6899	1357	0.016	1.077	12.9134	13:1 w5c	0.03	ECL deviates -0.007		
1.7144	1940	0.014	1.073	13.0013	13:0	0.05	ECL deviates  0.001	Reference -0.003	
1.7812	1135	0.018	----	13.1884	12:0 2OH	----	ECL deviates  0.002		
1.8717	2377	0.019	----	13.4410		----			
1.9323	71784	0.014	1.043	13.6103	14:0 iso	1.67	ECL deviates -0.004	Reference -0.008	
1.9727	1245	0.014	1.039	13.7231	14:0 anteiso	0.03	ECL deviates  0.007	Reference  0.003	
1.9922	1323	0.010	1.036	13.7775	14:1 w9c	0.03	ECL deviates  0.000		
2.0067	2391	0.015	----	13.8179		----			
2.0719	58319	0.015	1.028	14.0000	14:0	1.34	ECL deviates  0.000	Reference -0.004	
2.0979	823	0.011	----	14.0594		----			
2.1268	1262	0.016	----	14.1249	14:0 iso 3OH	----	ECL deviates  0.000		
2.1527	3955	0.025	----	14.1833		----			
2.2182	2905	0.020	----	14.3313		----			
2.2648	55515	0.018	1.013	14.4367	15:1 iso w6c	1.26	ECL deviates -0.002		
2.2834	8585	0.010	1.011	14.4788	15:4 w3c	0.19	ECL deviates -0.011		
2.3046	15664	0.015	1.010	14.5266	15:1 anteiso w9c	0.35	ECL deviates -0.003		
2.3440	260365	0.014	1.008	14.6157	15:0 iso	5.86	ECL deviates -0.001	Reference -0.006	
2.3855	202579	0.014	1.005	14.7093	15:0 anteiso	4.54	ECL deviates -0.002	Reference -0.006	
2.4497	9453	0.025	1.001	14.8544	15:1 w6c	0.21	ECL deviates -0.006		
2.5136	27196	0.015	0.998	14.9989	15:0	0.61	ECL deviates -0.001	Reference -0.005	
2.5419	10922	0.017	----	15.0535		----			
2.6035	2749	0.019	----	15.1706		----			
2.6338	4081	0.021	----	15.2282		----			
2.7213	8132	0.013	0.990	15.3944	16:1 w7c alcohol	0.18	ECL deviates -0.002		
2.7458	43947	0.021	0.989	15.4408	15:0 DMA	0.97	ECL deviates -0.010		
2.8064	99397	0.016	0.987	15.5561	16:0 N alcohol	2.19	ECL deviates -0.001		
2.8391	111113	0.017	0.986	15.6183	16:0 iso	2.44	ECL deviates -0.001	Reference -0.006	
2.9170	72373	0.018	0.983	15.7663	16:1 w9c	1.59	ECL deviates -0.009		
2.9470	504493	0.019	0.983	15.8232	16:1 w7c	11.06	Column Overload		
2.9933	135336	0.016	0.981	15.9112	16:1 w5c	2.96	ECL deviates  0.000		
3.0428	512237	0.016	0.980	16.0051	16:0	11.21	Column Overload		
3.0689	18337	0.013	----	16.0489		----			
3.0860	9153	0.011	----	16.0774		----			
3.1224	3338	0.017	0.979	16.1384	16:2 DMA	0.07	ECL deviates  0.000		
3.1561	7281	0.022	----	16.1948		----			
3.1946	4139	0.019	----	16.2594		----			
3.2269	2961	0.020	0.977	16.3135	16:1 w7c DMA	0.06	ECL deviates  0.003		
3.2911	276789	0.019	0.976	16.4208	16:0 10-methyl	6.03	ECL deviates  0.001		
3.3271	58457	0.018	----	16.4812		----			
3.3549	30306	0.018	----	16.5277		----			
3.4103	63688	0.017	0.974	16.6205	17:0 iso	1.38	ECL deviates -0.003	Reference -0.008	
3.4675	75677	0.017	0.973	16.7163	17:0 anteiso	1.64	ECL deviates -0.004		
3.5112	44300	0.018	0.973	16.7895	17:1 w8c	0.96	ECL deviates -0.007		
3.5710	172205	0.018	0.972	16.8897	17:0 cyclo w7c	3.74	ECL deviates -0.004		
3.6353	21276	0.018	0.972	16.9974	17:0	0.46	ECL deviates -0.003	Reference -0.007	
3.6612	29934	0.016	0.971	17.0375	17:1 w7c 10-methyl	0.65	ECL deviates -0.006		
3.7033	7492	0.017	----	17.1017		----			
3.7411	2276	0.022	----	17.1594		----			
3.7879	3631	0.019	0.971	17.2308	16:0 2OH	0.08	ECL deviates -0.009		
3.8412	712	0.015	----	17.3121		----			
3.8989	29041	0.019	0.970	17.4002	17:0 10-methyl	0.63	ECL deviates -0.007		
3.9346	3120	0.014	0.970	17.4547	17:0 DMA	0.07	ECL deviates -0.003		
3.9565	7307	0.021	----	17.4880		----			
4.0119	8963	0.014	0.970	17.5727	18:3 w6c	0.19	ECL deviates -0.007		
4.0309	27451	0.025	----	17.6016		----			
4.1069	96091	0.019	0.970	17.7176	18:2 w6c	2.08	ECL deviates -0.010		
4.1409	343230	0.020	0.970	17.7695	18:1 w9c	7.43	ECL deviates -0.005		
4.1777	477721	0.017	0.969	17.8256	18:1 w7c	10.34	Column Overload		
4.2329	61756	0.023	----	17.9099		----			
4.2902	81661	0.018	0.969	17.9974	18:0	1.77	ECL deviates -0.003	Reference -0.008	
4.3446	28472	0.019	0.969	18.0763	18:1 w7c 10-methyl	0.62	ECL deviates -0.009		
4.3991	9128	0.028	0.969	18.1552	18:2 DMA	0.20	ECL deviates -0.005		
4.4469	5636	0.021	0.969	18.2243	18:1 w9c DMA	0.12	ECL deviates -0.013		
4.4817	2221	0.019	0.970	18.2746	18:1 w7c DMA	0.05	ECL deviates -0.008		
4.5049	2042	0.016	----	18.3081		----			
4.5585	138280	0.020	0.970	18.3856	18:0 10-methyl	2.99	ECL deviates -0.009		
4.6289	3797	0.020	0.970	18.4875	19:4 w6c	0.08	ECL deviates  0.002		
4.6705	9425	0.026	0.970	18.5475	19:3 w6c	0.20	ECL deviates -0.012		
4.7250	1610	0.010	0.970	18.6264	19:0 iso	0.03	ECL deviates -0.004		
4.7407	3535	0.019	0.970	18.6491	19:3 w3c	0.08	ECL deviates -0.009		
4.8040	17110	0.022	----	18.7406		----		Reference  0.008	
4.8484	15824	0.019	0.970	18.8047	19:1 w8c	0.34	ECL deviates -0.006		
4.8869	25294	0.018	0.970	18.8604	19:1 w6c	0.55	ECL deviates  0.008		
4.9142	111507	0.018	0.970	18.8998	19:0 cyclo w7c	2.41	ECL deviates -0.010		
4.9829	88299	0.019	----	18.9992	19:0	----	ECL deviates -0.001		
5.0440	2571	0.018	----	19.0847		----			
5.1340	4243	0.026	----	19.2103		----			
5.1699	13487	0.019	----	19.2603		----			
5.2564	32321	0.031	----	19.3810		----			
5.3110	11598	0.021	0.971	19.4571	20:5 w3c	----	Below has same name		
5.3457	3344	0.017	----	19.5055	20:5 w3c	----	Above has same name		
5.3774	6965	0.021	----	19.5497		----			
5.4091	12536	0.023	----	19.5940		----			
5.5283	32554	0.026	0.972	19.7602	20:1 w9c	0.71	ECL deviates -0.012		
5.5595	12045	0.022	0.972	19.8037	20:1 w8c	0.26	ECL deviates -0.009		
5.6123	1069	0.015	----	19.8774		----			
5.6334	1698	0.021	----	19.9069		----			
5.6976	26878	0.021	0.972	19.9964	20:0	0.58	ECL deviates -0.004	Reference -0.010	
5.7484	1259	0.017	----	20.0668		----			
5.8000	3203	0.018	----	20.1383		----			
5.8302	7963	0.020	----	20.1802		----			
5.9414	10130	0.025	----	20.3342		----			
5.9720	39225	0.023	----	20.3766		----			
6.0451	872	0.013	----	20.4780		----			
6.0982	4024	0.031	----	20.5516		----			
6.1437	6329	0.022	----	20.6147		----			
6.1677	3487	0.016	0.971	20.6479	21:3 w3c	0.08	ECL deviates -0.006		
6.2089	5663	0.029	----	20.7050		----			
6.2733	16329	0.022	0.971	20.7942	21:1 w8c	0.35	ECL deviates -0.004		
6.3314	11306	0.024	----	20.8747		----			
6.3908	28880	0.020	0.970	20.9570	21:1 w3c	0.63	ECL deviates  0.003		
6.4241	7507	0.022	0.970	21.0032	21:0	0.16	ECL deviates  0.003	Reference -0.005	
6.5058	5092	0.022	----	21.1160		----			
6.5497	980	0.015	----	21.1766		----			
6.5912	4937	0.020	0.969	21.2340	22:5 w6c	0.11	ECL deviates -0.018		
6.6235	8836	0.018	----	21.2787		----			
6.6474	1660	0.011	0.969	21.3116	22:6 w3c	0.04	ECL deviates -0.020		
6.6868	851	0.017	----	21.3660		----			
6.7498	1342	0.025	0.968	21.4531	22:5 w3c	0.03	ECL deviates -0.014		
6.8730	14202	0.028	0.967	21.6233	22:0 iso	0.31	ECL deviates  0.005		
6.9464	3745	0.024	0.966	21.7246	22:2 w6c	0.08	ECL deviates -0.014		
6.9833	2909	0.021	0.965	21.7757	22:1 w9c	0.06	ECL deviates  0.003		
7.0156	5804	0.024	0.965	21.8203	22:1 w8c	0.13	ECL deviates  0.007		
7.1006	6808	0.019	0.964	21.9377	22:1 w3c	0.15	ECL deviates -0.009		
7.1466	30878	0.019	0.963	22.0013	22:0	0.66	ECL deviates  0.001	Reference -0.008	
7.2079	2178	0.022	----	22.0870		----			
7.2405	1055	0.020	----	22.1326		----			
7.3199	12869	0.019	----	22.2438		----			
7.3745	769	0.014	----	22.3201		----			
7.4365	1332	0.019	----	22.4070		----			
7.4916	884	0.017	0.957	22.4842	23:4 w6c	0.02	ECL deviates  0.013		
7.5317	772	0.016	----	22.5404		----			
7.6037	2230	0.027	0.954	22.6411	23:3 w3c	0.05	ECL deviates -0.004		
7.6438	674	0.016	----	22.6973		----			
7.7007	4925	0.021	----	22.7770		----			
7.7616	2566	0.020	----	22.8623		----			
7.8025	13520	0.019	0.949	22.9195	23:1 w4c	0.29	ECL deviates -0.007		
7.8587	6372	0.017	0.947	22.9982	23:0	0.13	ECL deviates -0.002	Reference -0.013	
7.9089	2105	0.022	----	23.0693		----			
8.0675	7909	0.023	----	23.2939		----			
8.3176	10870	0.028	0.931	23.6482	24:3 w3c	0.23	ECL deviates -0.007		
8.3752	3899	0.025	----	23.7299		----			
8.4071	3820	0.024	----	23.7751		----			
8.4799	3402	0.028	----	23.8782		----			
8.5149	1453	0.018	----	23.9278		----			
8.5645	24735	0.019	0.920	23.9980	24:0	0.51	ECL deviates -0.002	Reference -0.015	
8.6677	1552	0.023	----	24.1442		----	> max rt		
8.7626	4341	0.048	----	24.2788		----	> max rt		
8.9201	11444	0.022	----	24.5020		----	> max rt		
9.1527	1243	0.022	----	24.8315		----	> max rt		
9.2230	25538	0.023	----	24.9311		----	> max rt		
9.4579	11857	0.022	----	25.2641		----	> max rt		

ECL Deviation: 0.008                            Reference ECL Shift: 0.008       Number Reference Peaks: 21
Total Response: 5108941                       Total Named: 4566024
Percent Named: 89.37%                         Total Amount: 4491702
Profile Comment:   Column Overload:  A peak's response is greater than 400000.0.  Dilute and re-run.

(No search libraries specified in method PLFAD1.)
