# Supplementary material for: Microbial regulation of soil carbon properties under nitrogen addition and plant inputs removal
Source: PeerJ. 2019 Jul 17;7:e7343. doi: 10.7717/peerj.7343 (PMC6642627; doi:10.7717/peerj.7343)
Supplement: File S1 — The raw data showed the soil microbial PLFAs files in the year of 2015 and 2016. Each file of rtf. represented the microbial PLFAs for each soil sample. In the Supplemental File, the Excel file named “Numbers” showed the plots names and the related rtf. file names. [file peerj-07-7343-s002.zip › supplementary files/2015/56.rtf]

Volume: DATA            File: E164216.88A        Samp Ctr: 15                ID Number: 29353 
Type: Samp                   Bottle: 2                        Method: PLFAD1 
Created: 4/21/2016 8:42:55 PM 
Sample ID: 56 


RT	Response	Ar/Ht	RFact	ECL	Peak Name	Percent	Comment1	Comment2	
0.7145	1.901E+9	0.016	----	7.6441	SOLVENT PEAK	----	< min rt		
0.8859	539	0.008	----	8.7772		----	< min rt		
1.1867	1750	0.013	----	10.7444		----			
1.2622	1017	0.013	1.190	11.1748	10:0 2OH	0.03	ECL deviates -0.009		
1.3094	965	0.018	----	11.4001		----			
1.3527	1352	0.018	1.155	11.6072	12:0 iso	0.04	ECL deviates -0.005	Reference -0.007	
1.3639	564	0.009	----	11.6606		----			
1.3895	2400	0.014	----	11.7832		----			
1.4361	5437	0.016	1.127	12.0049	12:0	0.16	ECL deviates  0.005	Reference  0.004	
1.4937	3536	0.016	----	12.2117		----			
1.5198	810	0.013	----	12.3055		----			
1.5585	2558	0.021	----	12.4446		----			
1.6044	4679	0.012	1.089	12.6097	13:0 iso	0.13	ECL deviates -0.003	Reference -0.003	
1.6324	2979	0.016	1.083	12.7104	13:0 anteiso	0.08	ECL deviates  0.001	Reference  0.001	
1.6889	1583	0.021	1.072	12.9134	13:1 w5c	0.04	ECL deviates -0.007		
1.7137	2593	0.016	1.067	13.0021	13:0	0.07	ECL deviates  0.002	Reference  0.002	
1.7801	994	0.019	----	13.1875	12:0 2OH	----	ECL deviates  0.001		
1.8718	2265	0.019	----	13.4434		----			
1.9317	45050	0.014	1.039	13.6105	14:0 iso	1.21	ECL deviates -0.004	Reference -0.003	
1.9735	1294	0.013	1.035	13.7270	14:0 anteiso	0.03	ECL deviates  0.011	Reference  0.012	
1.9917	1119	0.010	1.032	13.7779	14:1 w9c	0.03	ECL deviates  0.000		
2.0071	2213	0.014	----	13.8207		----			
2.0400	4223	0.013	1.027	13.9128	14:1 w5c	0.11	ECL deviates  0.002		
2.0712	46244	0.015	1.024	13.9996	14:0	1.23	ECL deviates  0.000	Reference  0.001	
2.1267	1122	0.013	----	14.1251	14:0 iso 3OH	----	ECL deviates  0.000		
2.1526	3389	0.025	----	14.1836		----			
2.2181	3355	0.021	----	14.3316		----			
2.2649	51184	0.017	1.009	14.4374	15:1 iso w6c	1.34	ECL deviates -0.002		
2.2828	9065	0.011	1.007	14.4778	15:4 w3c	0.24	ECL deviates -0.012		
2.3050	14439	0.014	1.006	14.5279	15:1 anteiso w9c	0.38	ECL deviates -0.002		
2.3437	236771	0.014	1.004	14.6153	15:0 iso	6.17	ECL deviates -0.002	Reference  0.000	
2.3851	171956	0.014	1.001	14.7090	15:0 anteiso	4.47	ECL deviates -0.002	Reference -0.001	
2.4504	9263	0.025	0.997	14.8564	15:1 w6c	0.24	ECL deviates -0.004		
2.5137	25553	0.015	0.994	14.9993	15:0	0.66	ECL deviates -0.001	Reference  0.001	
2.5418	7320	0.017	----	15.0527		----			
2.6036	2126	0.020	----	15.1702		----			
2.6343	3920	0.022	----	15.2286		----			
2.7194	6728	0.014	0.985	15.3901	16:1 w7c alcohol	0.17	ECL deviates -0.006		
2.7463	39663	0.021	0.984	15.4412	15:0 DMA	1.01	ECL deviates -0.009		
2.8059	74066	0.015	0.982	15.5546	16:0 N alcohol	1.89	ECL deviates -0.002		
2.8382	98191	0.016	0.981	15.6159	16:0 iso	2.50	ECL deviates -0.004	Reference -0.002	
2.8907	10088	0.016	0.979	15.7156	16:0 anteiso	0.26	ECL deviates  0.001	Reference  0.003	
2.9159	53229	0.017	0.978	15.7636	16:1 w9c	1.35	ECL deviates -0.011		
2.9457	404166	0.016	0.977	15.8202	16:1 w7c	10.25	Column Overload		
2.9936	157004	0.016	0.976	15.9112	16:1 w5c	3.98	ECL deviates  0.000		
3.0426	437030	0.015	0.975	16.0036	16:0	11.05	Column Overload		
3.0690	19728	0.020	----	16.0478		----			
3.1222	3347	0.016	0.973	16.1369	16:2 DMA	0.08	ECL deviates -0.001		
3.1564	6592	0.023	----	16.1943		----			
3.1924	3533	0.018	----	16.2546		----			
3.2296	2201	0.020	0.971	16.3169	16:1 w7c DMA	0.06	ECL deviates  0.007		
3.2912	216400	0.020	0.970	16.4202	16:0 10-methyl	5.45	ECL deviates  0.000		
3.3255	45087	0.018	0.969	16.4776	16:0 DMA	1.13	ECL deviates  0.014		
3.3538	28974	0.018	----	16.5251		----			
3.4101	53629	0.017	0.968	16.6195	17:0 iso	1.35	ECL deviates -0.004	Reference -0.002	
3.4669	67007	0.017	0.967	16.7147	17:0 anteiso	1.68	ECL deviates -0.006		
3.5113	46680	0.019	0.967	16.7890	17:1 w8c	1.17	ECL deviates -0.008		
3.5715	133049	0.017	0.966	16.8900	17:0 cyclo w7c	3.33	ECL deviates -0.004		
3.6360	18312	0.018	0.965	16.9980	17:0	0.46	ECL deviates -0.002	Reference  0.000	
3.6615	21101	0.018	0.965	17.0371	17:1 w7c 10-methyl	0.53	ECL deviates -0.006		
3.7041	5846	0.016	----	17.1022		----			
3.7392	2107	0.021	----	17.1558		----			
3.7893	3016	0.018	0.964	17.2325	16:0 2OH	0.08	ECL deviates -0.008		
3.8434	629	0.012	----	17.3152		----			
3.8992	25874	0.017	0.963	17.4006	17:0 10-methyl	0.65	ECL deviates -0.006		
3.9346	2094	0.011	0.963	17.4547	17:0 DMA	0.05	ECL deviates -0.003		
3.9567	7789	0.023	----	17.4885		----			
4.0302	34745	0.028	----	17.6010		----			
4.1067	88130	0.018	0.962	17.7180	18:2 w6c	2.20	ECL deviates -0.009		
4.1402	258472	0.019	0.962	17.7692	18:1 w9c	6.45	ECL deviates -0.005		
4.1781	459195	0.017	0.962	17.8271	18:1 w7c	11.46	Column Overload		
4.2330	56681	0.023	----	17.9111		----			
4.2904	67507	0.018	0.962	17.9990	18:0	1.68	ECL deviates -0.001	Reference  0.001	
4.3461	26357	0.018	0.962	18.0793	18:1 w7c 10-methyl	0.66	ECL deviates -0.006		
4.4117	8565	0.028	0.962	18.1740	18:2 DMA	0.21	ECL deviates  0.014		
4.4490	6096	0.028	0.962	18.2278	18:1 w9c DMA	0.15	ECL deviates -0.009		
4.5062	1414	0.017	----	18.3105		----			
4.5593	97346	0.021	0.962	18.3871	18:0 10-methyl	2.43	ECL deviates -0.008		
4.6267	2813	0.020	0.962	18.4843	19:4 w6c	0.07	ECL deviates -0.001		
4.6711	8688	0.025	0.962	18.5485	19:3 w6c	0.22	ECL deviates -0.012		
4.7398	4789	0.025	----	18.6476		----			
4.8006	16874	0.023	0.962	18.7354	19:0 anteiso	0.42	ECL deviates  0.008	Reference  0.010	
4.8469	14807	0.020	0.963	18.8023	19:1 w8c	0.37	ECL deviates -0.009		
4.9128	125455	0.020	0.963	18.8974	19:0 cyclo w7c	3.13	ECL deviates -0.012		
4.9832	74948	0.017	----	18.9991	19:0	----	ECL deviates -0.001		
5.0442	2263	0.017	----	19.0843		----			
5.1358	2345	0.018	----	19.2122		----			
5.1704	9648	0.019	----	19.2605		----			
5.2569	27506	0.031	----	19.3814		----			
5.3104	8886	0.020	----	19.4563		----			
5.3456	2332	0.015	0.964	19.5055	20:5 w3c	0.06	ECL deviates  0.023		
5.3774	8573	0.019	----	19.5499		----			
5.4099	11603	0.026	----	19.5953		----			
5.5288	26437	0.028	0.965	19.7614	20:1 w9c	0.66	ECL deviates -0.011		
5.5608	9921	0.024	0.965	19.8061	20:1 w8c	0.25	ECL deviates -0.007		
5.6446	869	0.014	0.966	19.9233	20:1 w4c	0.02	ECL deviates -0.008		
5.6980	23493	0.021	0.966	19.9979	20:0	0.59	ECL deviates -0.002	Reference -0.001	
5.7548	999	0.016	----	20.0762		----			
5.7996	3133	0.017	----	20.1381		----			
5.8321	7331	0.020	----	20.1831		----			
5.9160	2722	0.015	----	20.2988		----			
5.9424	5950	0.017	----	20.3352		----			
5.9738	31573	0.024	----	20.3786		----			
6.0495	924	0.015	----	20.4832		----			
6.0996	5114	0.027	----	20.5522		----			
6.1458	7848	0.031	----	20.6161		----			
6.2118	4741	0.030	----	20.7072		----			
6.2740	10105	0.020	0.968	20.7931	21:1 w8c	0.25	ECL deviates -0.005		
6.3318	10080	0.026	----	20.8729		----			
6.3901	17571	0.020	0.968	20.9534	21:1 w3c	0.44	ECL deviates -0.001		
6.4270	7464	0.026	0.968	21.0043	21:0	0.19	ECL deviates  0.004	Reference  0.004	
6.5064	3076	0.022	----	21.1138		----			
6.5535	1710	0.020	----	21.1788		----			
6.5910	4354	0.023	0.968	21.2305	22:5 w6c	0.11	ECL deviates -0.021		
6.6242	6134	0.019	----	21.2764		----			
6.6492	1703	0.018	0.968	21.3108	22:6 w3c	0.04	ECL deviates -0.021		
6.6927	963	0.017	----	21.3709		----			
6.7548	1372	0.027	0.968	21.4566	22:5 w3c	0.03	ECL deviates -0.011		
6.8735	15127	0.031	0.968	21.6204	22:0 iso	0.38	ECL deviates  0.003		
6.9528	3684	0.025	0.968	21.7298	22:2 w6c	0.09	ECL deviates -0.009		
6.9809	3942	0.022	0.968	21.7686	22:1 w9c	0.10	ECL deviates -0.004		
7.0188	6942	0.028	0.968	21.8208	22:1 w8c	0.17	ECL deviates  0.007		
7.1029	5715	0.022	0.967	21.9368	22:1 w3c	0.14	ECL deviates -0.010		
7.1460	27294	0.020	0.967	21.9964	22:0	0.68	ECL deviates -0.004	Reference -0.005	
7.2063	1449	0.020	----	22.0809		----			
7.2391	2094	0.028	----	22.1270		----			
7.3216	11215	0.020	----	22.2429		----			
7.3766	2303	0.032	----	22.3201		----			
7.4388	2210	0.023	----	22.4075		----			
7.4911	1477	0.024	0.965	22.4809	23:4 w6c	0.04	ECL deviates  0.010		
7.5302	1338	0.022	----	22.5359		----			
7.5952	4067	0.035	----	22.6271		----	> max ar/ht		
7.6416	1238	0.020	----	22.6923		----			
7.6998	5740	0.023	----	22.7742		----			
7.7610	3080	0.019	----	22.8600		----			
7.8042	9183	0.020	0.962	22.9207	23:1 w4c	0.23	ECL deviates -0.006		
7.8620	6759	0.017	0.961	23.0020	23:0	0.17	ECL deviates  0.002	Reference -0.002	
7.9089	1936	0.027	----	23.0684		----			
8.0690	6595	0.022	----	23.2954		----			
8.3163	10049	0.025	----	23.6460		----			
8.3745	3534	0.022	----	23.7285		----			
8.4108	4836	0.026	0.951	23.7800	24:1 w9c	0.12	ECL deviates -0.007		
8.4824	2984	0.028	----	23.8815		----			
8.5209	905	0.014	0.948	23.9361	24:1 w3c	0.02	ECL deviates -0.013		
8.5647	24334	0.018	0.947	23.9983	24:0	0.60	ECL deviates -0.002	Reference -0.007	
8.6685	1014	0.020	----	24.1455		----	> max rt		
8.7521	1056	0.024	----	24.2640		----	> max rt		
8.9226	10040	0.019	----	24.5058		----	> max rt		
9.2221	20358	0.022	----	24.9305		----	> max rt		
9.4619	11748	0.020	----	25.2707		----	> max rt		

ECL Deviation: 0.008                            Reference ECL Shift: 0.004       Number Reference Peaks: 22
Total Response: 4382838                       Total Named: 3949887
Percent Named: 90.12%                         Total Amount: 3854263
Profile Comment:   Column Overload:  A peak's response is greater than 400000.0.  Dilute and re-run.

(No search libraries specified in method PLFAD1.)
