# Supplementary material for: Microbial regulation of soil carbon properties under nitrogen addition and plant inputs removal
Source: PeerJ. 2019 Jul 17;7:e7343. doi: 10.7717/peerj.7343 (PMC6642627; doi:10.7717/peerj.7343)
Supplement: File S1 — The raw data showed the soil microbial PLFAs files in the year of 2015 and 2016. Each file of rtf. represented the microbial PLFAs for each soil sample. In the Supplemental File, the Excel file named “Numbers” showed the plots names and the related rtf. file names. [file peerj-07-7343-s002.zip › supplementary files/2015/57.rtf]

Volume: DATA            File: E164216.88A        Samp Ctr: 16                ID Number: 29354 
Type: Samp                   Bottle: 3                        Method: PLFAD1 
Created: 4/21/2016 9:02:24 PM 
Sample ID: 57 


RT	Response	Ar/Ht	RFact	ECL	Peak Name	Percent	Comment1	Comment2	
0.7143	1.909E+9	0.016	----	7.6643	SOLVENT PEAK	----	< min rt		
0.8596	877	0.013	----	8.6132		----	< min rt		
0.8716	583	0.006	----	8.6914		----	< min rt		
0.8853	2113	0.013	----	8.7811		----	< min rt		
0.9439	2975	0.012	----	9.1635		----	< min rt		
0.9597	689	0.009	----	9.2670		----	< min rt		
0.9707	568	0.009	----	9.3384		----	< min rt		
1.0445	4082	0.014	----	9.8209		----	< min rt		
1.0609	2124	0.011	----	9.9280		----	< min rt		
1.0726	1671	0.011	1.305	10.0044	10:0	0.04	ECL deviates  0.004	Reference -0.007	
1.0973	627	0.012	----	10.1655		----			
1.1483	1320	0.015	----	10.4989		----			
1.1852	3194	0.014	----	10.7402		----			
1.2239	1652	0.016	1.206	10.9931	11:0	0.03	ECL deviates -0.007	Reference -0.015	
1.2607	2462	0.015	1.190	11.1726	10:0 2OH	0.05	ECL deviates -0.011		
1.3169	1498	0.016	1.169	11.4409	10:0 3OH	0.03	ECL deviates  0.000		
1.3519	2473	0.019	1.155	11.6084	12:0 iso	0.05	ECL deviates -0.004	Reference -0.010	
1.3890	2294	0.016	----	11.7856		----			
1.4351	7459	0.014	1.127	12.0055	12:0	0.15	ECL deviates  0.005	Reference  0.000	
1.4927	3257	0.013	----	12.2124		----			
1.5581	1662	0.014	----	12.4473		----			
1.6033	5774	0.012	1.089	12.6098	13:0 iso	0.11	ECL deviates -0.003	Reference -0.007	
1.6308	2746	0.014	1.083	12.7084	13:0 anteiso	0.05	ECL deviates -0.001	Reference -0.005	
1.6886	900	0.013	1.072	12.9161	13:1 w5c	0.02	ECL deviates -0.004		
1.7130	2678	0.012	1.067	13.0038	13:0	0.05	ECL deviates  0.004	Reference  0.000	
1.7810	1012	0.015	----	13.1934	12:0 2OH	----	ECL deviates  0.007		
1.8236	445	0.010	----	13.3122		----			
1.8720	2993	0.018	----	13.4471		----			
1.9310	66292	0.014	1.039	13.6116	14:0 iso	1.20	ECL deviates -0.003	Reference -0.005	
1.9694	1849	0.015	1.035	13.7187	14:0 anteiso	0.03	ECL deviates  0.003	Reference  0.000	
1.9908	1795	0.010	1.032	13.7784	14:1 w9c	0.03	ECL deviates  0.001		
2.0054	3128	0.014	----	13.8191		----			
2.0386	4461	0.013	1.027	13.9115	14:1 w5c	0.08	ECL deviates  0.001		
2.0706	82621	0.014	1.024	14.0008	14:0	1.47	ECL deviates  0.001	Reference -0.001	
2.1250	1906	0.015	----	14.1238	14:0 iso 3OH	----	ECL deviates -0.001		
2.1587	4917	0.023	----	14.1999		----			
2.2076	3431	0.023	----	14.3103		----			
2.2641	86243	0.019	1.009	14.4379	15:1 iso w6c	1.52	ECL deviates -0.001		
2.3047	23111	0.015	1.006	14.5296	15:1 anteiso w9c	0.41	ECL deviates  0.000		
2.3438	327983	0.015	1.004	14.6179	15:0 iso	5.74	ECL deviates  0.001	Reference  0.000	
2.3851	266984	0.014	1.001	14.7113	15:0 anteiso	4.66	ECL deviates  0.000	Reference -0.001	
2.4489	12966	0.025	0.997	14.8553	15:1 w6c	0.23	ECL deviates -0.005		
2.5136	43746	0.016	0.994	15.0013	15:0	0.76	ECL deviates  0.001	Reference  0.001	
2.5413	8268	0.017	----	15.0538		----			
2.6010	2602	0.024	----	15.1672		----			
2.6335	7968	0.020	----	15.2289		----			
2.7447	61969	0.024	0.984	15.4401	15:0 DMA	1.06	ECL deviates -0.010		
2.8060	95004	0.017	0.982	15.5566	16:0 N alcohol	1.63	ECL deviates  0.000		
2.8391	159332	0.016	0.981	15.6194	16:0 iso	2.72	ECL deviates  0.000	Reference  0.000	
2.8896	13536	0.015	0.979	15.7153	16:0 anteiso	0.23	ECL deviates  0.000	Reference  0.001	
2.9173	66324	0.017	0.978	15.7679	16:1 w9c	1.13	ECL deviates -0.007		
2.9470	537268	0.018	0.977	15.8243	16:1 w7c	9.15	Column Overload		
2.9935	183993	0.016	0.976	15.9126	16:1 w5c	3.13	ECL deviates  0.001		
3.0453	779579	0.017	0.975	16.0097	16:0	13.24	Column Overload		
3.0667	18079	0.019	----	16.0454		----			
3.1220	7750	0.017	0.973	16.1380	16:2 DMA	0.13	ECL deviates  0.000		
3.1578	11023	0.025	----	16.1979		----			
3.1918	4655	0.017	----	16.2549		----			
3.2308	2953	0.020	0.971	16.3203	16:1 w7c DMA	0.05	ECL deviates  0.010		
3.2908	269021	0.020	0.970	16.4207	16:0 10-methyl	4.55	ECL deviates  0.001		
3.3269	55019	0.018	----	16.4813		----			
3.3533	38353	0.019	----	16.5254		----			
3.4101	76664	0.017	0.968	16.6206	17:0 iso	1.29	ECL deviates -0.003	Reference -0.002	
3.4673	106617	0.018	0.967	16.7165	17:0 anteiso	1.80	ECL deviates -0.004		
3.5112	68673	0.018	0.967	16.7900	17:1 w8c	1.16	ECL deviates -0.007		
3.5714	194898	0.018	0.966	16.8909	17:0 cyclo w7c	3.28	ECL deviates -0.003		
3.6355	36589	0.018	0.965	16.9983	17:0	0.62	ECL deviates -0.002	Reference -0.001	
3.6613	20931	0.018	0.965	17.0377	17:1 w7c 10-methyl	0.35	ECL deviates -0.006		
3.7028	7610	0.016	----	17.1012		----			
3.7361	2502	0.019	----	17.1520		----			
3.7892	7413	0.020	0.964	17.2332	16:0 2OH	0.12	ECL deviates -0.007		
3.8437	736	0.014	----	17.3166		----			
3.8997	40050	0.018	0.963	17.4022	17:0 10-methyl	0.67	ECL deviates -0.005		
3.9588	16612	0.026	----	17.4925		----			
4.0323	57823	0.032	----	17.6049		----			
4.1079	205225	0.019	0.962	17.7205	18:2 w6c	3.44	ECL deviates -0.007		
4.1427	412171	0.022	0.962	17.7736	18:1 w9c	6.91	Column Overload		
4.1806	672791	0.018	0.962	17.8316	18:1 w7c	11.28	Column Overload		
4.2312	120105	0.022	----	17.9090		----			
4.2913	124200	0.017	0.962	18.0007	18:0	2.08	ECL deviates  0.001	Reference  0.002	
4.3458	47420	0.019	0.962	18.0795	18:1 w7c 10-methyl	0.79	ECL deviates -0.006		
4.4000	12808	0.027	0.962	18.1577	18:2 DMA	0.21	ECL deviates -0.002		
4.4411	10719	0.032	----	18.2170		----			
4.5058	2227	0.017	----	18.3104		----			
4.5591	139475	0.021	0.962	18.3872	18:0 10-methyl	2.34	ECL deviates -0.008		
4.6259	4511	0.022	0.962	18.4837	19:4 w6c	0.08	ECL deviates -0.001		
4.6713	11950	0.025	0.962	18.5492	19:3 w6c	0.20	ECL deviates -0.011		
4.7428	8439	0.024	0.962	18.6523	19:3 w3c	0.14	ECL deviates -0.006		
4.8072	17762	0.023	----	18.7453		----			
4.8459	19156	0.020	0.963	18.8011	19:1 w8c	0.32	ECL deviates -0.010		
4.8797	35420	0.020	0.963	18.8499	19:1 w6c	0.59	ECL deviates -0.002		
4.9144	149711	0.018	0.963	18.8999	19:0 cyclo w7c	2.51	ECL deviates -0.010		
4.9829	89241	0.020	----	18.9989	19:0	----	ECL deviates -0.001		
5.0430	5425	0.017	----	19.0828		----			
5.0743	2410	0.017	----	19.1265		----			
5.1356	4884	0.021	----	19.2122		----			
5.1704	28876	0.020	----	19.2608		----			
5.2409	40611	0.020	----	19.3593		----			
5.2550	24049	0.016	----	19.3789		----			
5.3102	18396	0.023	----	19.4561		----			
5.3463	6095	0.017	0.964	19.5065	20:5 w3c	0.10	ECL deviates  0.024		
5.3765	11894	0.021	----	19.5487		----			
5.4089	22423	0.030	----	19.5939		----			
5.5299	41248	0.026	0.965	19.7630	20:1 w9c	0.69	ECL deviates -0.010		
5.5619	15281	0.027	0.965	19.8076	20:1 w8c	0.26	ECL deviates -0.005		
5.7004	44025	0.025	0.966	20.0011	20:0	0.74	ECL deviates  0.001	Reference  0.002	
5.7515	3881	0.020	----	20.0718		----			
5.7988	5916	0.018	----	20.1370		----			
5.8318	15175	0.023	----	20.1825		----			
5.9104	7889	0.021	----	20.2911		----			
5.9399	9925	0.018	----	20.3317		----			
5.9727	42678	0.026	----	20.3770		----			
6.0797	5536	0.021	----	20.5247		----			
6.0991	7173	0.020	----	20.5515		----			
6.1465	8537	0.023	----	20.6170		----			
6.1686	4531	0.017	0.967	20.6474	21:3 w3c	0.08	ECL deviates -0.006		
6.2223	11229	0.031	----	20.7215		----			
6.2737	14995	0.022	0.968	20.7925	21:1 w8c	0.25	ECL deviates -0.005		
6.3308	27552	0.024	----	20.8713		----			
6.3900	16091	0.022	0.968	20.9530	21:1 w3c	0.27	ECL deviates -0.001		
6.4249	14972	0.029	0.968	21.0013	21:0	0.25	ECL deviates  0.001	Reference  0.001	
6.5060	4779	0.023	----	21.1132		----			
6.5475	4008	0.022	----	21.1704		----			
6.5910	9729	0.021	0.968	21.2304	22:5 w6c	0.16	ECL deviates -0.021		
6.6240	24686	0.023	----	21.2760		----			
6.6886	2410	0.018	----	21.3651		----			
6.7523	4378	0.031	0.968	21.4529	22:5 w3c	----	Below has same name		
6.7710	351	0.004	----	21.4787	22:5 w3c	----	Above has same name		
6.8132	3223	0.027	----	21.5370		----			
6.8724	24973	0.030	0.968	21.6186	22:0 iso	0.42	ECL deviates  0.001		
6.9296	1672	0.009	----	21.6974		----			
6.9558	5039	0.019	0.968	21.7337	22:2 w6c	0.08	ECL deviates -0.005		
6.9838	8129	0.023	0.968	21.7723	22:1 w9c	0.14	ECL deviates -0.001		
7.0209	15579	0.033	----	21.8235		----			
7.1039	7727	0.022	0.967	21.9380	22:1 w3c	0.13	ECL deviates -0.009		
7.1476	58941	0.023	0.967	21.9983	22:0	0.99	ECL deviates -0.002	Reference -0.003	
7.2015	3516	0.022	----	22.0739		----			
7.2431	6404	0.037	----	22.1324		----	> max ar/ht		
7.3221	17489	0.024	----	22.2434		----			
7.3758	2289	0.020	----	22.3187		----			
7.3988	1506	0.013	----	22.3510		----			
7.4389	4208	0.032	----	22.4073		----			
7.4921	2731	0.027	0.965	22.4821	23:4 w6c	0.05	ECL deviates  0.011		
7.5340	3139	0.023	----	22.5409		----			
7.6035	7102	0.041	0.964	22.6386	23:3 w3c	----	> max ar/ht		
7.6348	805	0.007	----	22.6826		----			
7.6428	1241	0.012	----	22.6938		----			
7.7021	11938	0.027	----	22.7771		----			
7.7616	4764	0.022	----	22.8607		----			
7.8049	14281	0.022	0.962	22.9215	23:1 w4c	0.24	ECL deviates -0.005		
7.8617	14006	0.021	0.961	23.0013	23:0	0.23	ECL deviates  0.001	Reference -0.002	
7.9059	3569	0.030	----	23.0640		----			
7.9688	1063	0.021	----	23.1531		----			
8.0364	1385	0.016	----	23.2489		----			
8.0700	12239	0.020	----	23.2966		----			
8.3201	14297	0.029	0.953	23.6511	24:3 w3c	0.24	ECL deviates -0.004		
8.3767	6907	0.021	----	23.7315		----			
8.4086	9108	0.027	0.951	23.7767	24:1 w9c	0.15	ECL deviates -0.010		
8.4799	4482	0.029	----	23.8778		----			
8.5157	1368	0.016	----	23.9285		----			
8.5655	36496	0.018	0.947	23.9992	24:0	0.60	ECL deviates -0.001	Reference -0.006	
8.6683	1990	0.021	----	24.1450		----	> max rt		
8.7558	3072	0.030	----	24.2691		----	> max rt		
8.7910	909	0.015	----	24.3190		----	> max rt		
8.9227	10820	0.020	----	24.5059		----	> max rt		
9.0220	998	0.021	----	24.6466		----	> max rt		
9.1521	2130	0.020	----	24.8313		----	> max rt		
9.2242	20171	0.020	----	24.9335		----	> max rt		
9.2521	4206	0.015	----	24.9732		----	> max rt		
9.4627	16195	0.024	----	25.2720		----	> max rt		

ECL Deviation: 0.007                            Reference ECL Shift: 0.005       Number Reference Peaks: 23
Total Response: 6752191                       Total Named: 5881900
Percent Named: 87.11%                         Total Amount: 5750146
Profile Comment:   Column Overload:  A peak's response is greater than 400000.0.  Dilute and re-run.

(No search libraries specified in method PLFAD1.)
