# Supplementary material for: Microbial regulation of soil carbon properties under nitrogen addition and plant inputs removal
Source: PeerJ. 2019 Jul 17;7:e7343. doi: 10.7717/peerj.7343 (PMC6642627; doi:10.7717/peerj.7343)
Supplement: File S1 — The raw data showed the soil microbial PLFAs files in the year of 2015 and 2016. Each file of rtf. represented the microbial PLFAs for each soil sample. In the Supplemental File, the Excel file named “Numbers” showed the plots names and the related rtf. file names. [file peerj-07-7343-s002.zip › supplementary files/2015/58.rtf]

Volume: DATA            File: E164216.88A        Samp Ctr: 17                ID Number: 29355 
Type: Samp                   Bottle: 4                        Method: PLFAD1 
Created: 4/21/2016 9:22:03 PM 
Sample ID: 58 


RT	Response	Ar/Ht	RFact	ECL	Peak Name	Percent	Comment1	Comment2	
0.7139	1.915E+9	0.016	----	7.6587	SOLVENT PEAK	----	< min rt		
0.8596	541	0.012	----	8.6107		----	< min rt		
0.8846	1789	0.012	----	8.7741		----	< min rt		
0.9441	440	0.010	----	9.1629		----	< min rt		
1.0447	762	0.010	----	9.8206		----	< min rt		
1.0723	1229	0.018	1.305	10.0007	10:0	0.03	ECL deviates  0.001	Reference -0.009	
1.1481	719	0.015	----	10.4964		----			
1.1853	3037	0.014	----	10.7395		----			
1.2248	893	0.014	1.206	10.9980	11:0	0.02	ECL deviates -0.002	Reference -0.009	
1.2602	1597	0.015	----	11.1697		----			
1.3178	1369	0.015	1.169	11.4445	10:0 3OH	0.03	ECL deviates  0.003		
1.3514	1763	0.015	1.155	11.6055	12:0 iso	0.04	ECL deviates -0.007		
1.3635	590	0.008	----	11.6630		----			
1.3883	1989	0.015	----	11.7813		----			
1.4348	6861	0.014	1.127	12.0038	12:0	0.14	ECL deviates  0.004	Reference -0.001	
1.4923	3045	0.014	----	12.2107		----			
1.5192	468	0.012	----	12.3071		----			
1.5576	2122	0.017	----	12.4451		----			
1.6031	6607	0.013	1.089	12.6087	13:0 iso	0.13	ECL deviates -0.004	Reference -0.008	
1.6308	4066	0.016	1.083	12.7084	13:0 anteiso	0.08	ECL deviates -0.001	Reference -0.005	
1.6880	1009	0.014	1.072	12.9139	13:1 w5c	0.02	ECL deviates -0.006		
1.7127	2950	0.014	1.067	13.0024	13:0	0.06	ECL deviates  0.002	Reference -0.001	
1.7811	851	0.017	----	13.1935	12:0 2OH	----	ECL deviates  0.007		
1.8227	401	0.009	----	13.3094		----			
1.8723	3112	0.019	----	13.4478		----			
1.9308	69197	0.014	1.039	13.6109	14:0 iso	1.34	ECL deviates -0.003	Reference -0.006	
1.9696	1537	0.013	1.035	13.7192	14:0 anteiso	0.03	ECL deviates  0.003	Reference  0.001	
1.9914	1823	0.011	1.032	13.7800	14:1 w9c	0.04	ECL deviates  0.003		
2.0057	2701	0.013	----	13.8200		----			
2.0380	4346	0.014	1.027	13.9101	14:1 w5c	0.08	ECL deviates -0.001		
2.0703	73384	0.014	1.024	14.0002	14:0	1.40	ECL deviates  0.000	Reference -0.002	
2.0993	800	0.010	----	14.0660		----			
2.1247	1971	0.014	----	14.1233	14:0 iso 3OH	----	ECL deviates -0.002		
2.1613	6204	0.023	----	14.2059		----			
2.2061	3285	0.020	----	14.3072		----			
2.2643	97256	0.019	1.009	14.4386	15:1 iso w6c	1.83	ECL deviates  0.000		
2.3043	25307	0.014	1.006	14.5289	15:1 anteiso w9c	0.47	ECL deviates -0.001		
2.3436	336464	0.015	1.004	14.6176	15:0 iso	6.29	ECL deviates  0.001	Reference -0.001	
2.3850	249329	0.014	1.001	14.7111	15:0 anteiso	4.65	ECL deviates  0.000	Reference -0.001	
2.4494	12501	0.026	0.997	14.8567	15:1 w6c	0.23	ECL deviates -0.003		
2.5134	35812	0.015	0.994	15.0012	15:0	0.66	ECL deviates  0.001	Reference  0.000	
2.5418	8980	0.017	----	15.0551		----			
2.6037	2931	0.022	----	15.1726		----			
2.6334	7272	0.020	----	15.2291		----			
2.7195	8922	0.014	0.985	15.3924	16:1 w7c alcohol	0.16	ECL deviates -0.004		
2.7443	56572	0.022	0.984	15.4396	15:0 DMA	1.04	ECL deviates -0.011		
2.8051	84823	0.017	0.982	15.5551	16:0 N alcohol	1.55	ECL deviates -0.002		
2.8381	145509	0.016	0.981	15.6177	16:0 iso	2.66	ECL deviates -0.002	Reference -0.002	
2.8898	11855	0.012	0.979	15.7159	16:0 anteiso	0.22	ECL deviates  0.001	Reference  0.001	
2.9174	75238	0.018	0.978	15.7683	16:1 w9c	1.37	ECL deviates -0.007		
2.9467	559828	0.018	0.977	15.8240	16:1 w7c	10.20	Column Overload		
2.9939	190213	0.017	0.976	15.9136	16:1 w5c	3.46	ECL deviates  0.003		
3.0438	622754	0.016	0.975	16.0073	16:0	11.31	Column Overload		
3.0687	14557	0.014	----	16.0490		----			
3.0869	6301	0.012	----	16.0796		----			
3.1225	7086	0.018	0.973	16.1393	16:2 DMA	0.13	ECL deviates  0.001		
3.1577	11282	0.024	----	16.1981		----			
3.1941	4596	0.017	----	16.2592		----			
3.2290	3137	0.020	0.971	16.3175	16:1 w7c DMA	0.06	ECL deviates  0.008		
3.2913	300419	0.020	0.970	16.4220	16:0 10-methyl	5.43	ECL deviates  0.002		
3.3272	54096	0.018	----	16.4821		----			
3.3543	38234	0.019	----	16.5275		----			
3.4107	74417	0.016	0.968	16.6220	17:0 iso	1.34	ECL deviates -0.002	Reference -0.001	
3.4679	95016	0.017	0.967	16.7178	17:0 anteiso	1.71	ECL deviates -0.003		
3.5116	67486	0.017	0.967	16.7910	17:1 w8c	1.22	ECL deviates -0.006		
3.5719	196247	0.018	0.966	16.8919	17:0 cyclo w7c	3.53	ECL deviates -0.002		
3.6358	29549	0.019	0.965	16.9989	17:0	0.53	ECL deviates -0.001	Reference  0.000	
3.6617	24221	0.018	0.965	17.0386	17:1 w7c 10-methyl	0.44	ECL deviates -0.005		
3.7047	8014	0.016	----	17.1044		----			
3.7389	2935	0.020	----	17.1565		----			
3.7886	5870	0.020	0.964	17.2325	16:0 2OH	0.11	ECL deviates -0.008		
3.8437	817	0.013	----	17.3167		----			
3.9003	41715	0.018	0.963	17.4033	17:0 10-methyl	0.75	ECL deviates -0.004		
3.9587	16341	0.028	----	17.4925		----			
4.0309	50027	0.030	----	17.6030		----			
4.1077	153523	0.018	0.962	17.7203	18:2 w6c	2.75	ECL deviates -0.007		
4.1430	373545	0.020	0.962	17.7743	18:1 w9c	6.70	ECL deviates  0.000		
4.1805	598007	0.017	0.962	17.8316	18:1 w7c	10.72	Column Overload		
4.2336	87725	0.021	----	17.9128		----			
4.2912	100253	0.020	0.962	18.0007	18:0	1.80	ECL deviates  0.001	Reference  0.002	
4.3477	43602	0.019	0.962	18.0822	18:1 w7c 10-methyl	0.78	ECL deviates -0.003		
4.3990	13362	0.028	0.962	18.1563	18:2 DMA	0.24	ECL deviates -0.004		
4.4481	6358	0.023	0.962	18.2271	18:1 w9c DMA	0.11	ECL deviates -0.010		
4.4758	2602	0.016	----	18.2671		----			
4.5075	1756	0.015	----	18.3129		----			
4.5600	150974	0.021	0.962	18.3886	18:0 10-methyl	2.71	ECL deviates -0.006		
4.6310	4135	0.022	0.962	18.4911	19:4 w6c	0.07	ECL deviates  0.006		
4.6707	12351	0.025	0.962	18.5483	19:3 w6c	0.22	ECL deviates -0.012		
4.7418	7853	0.024	0.962	18.6509	19:3 w3c	0.14	ECL deviates -0.007		
4.8062	15673	0.021	----	18.7437		----			
4.8473	17066	0.023	0.963	18.8031	19:1 w8c	0.31	ECL deviates -0.008		
4.8863	25934	0.016	0.963	18.8594	19:1 w6c	0.47	ECL deviates  0.007		
4.9153	147890	0.018	0.963	18.9012	19:0 cyclo w7c	2.65	ECL deviates -0.009		
4.9848	79913	0.019	----	19.0014	19:0	----	ECL deviates  0.001		
5.0437	4173	0.016	----	19.0837		----			
5.1352	6753	0.021	----	19.2114		----			
5.1710	22652	0.021	----	19.2615		----			
5.2569	51005	0.034	----	19.3814		----			
5.3112	14027	0.022	0.964	19.4572	20:5 w3c	0.25	ECL deviates -0.025		
5.3481	3148	0.015	----	19.5087		----			
5.3783	9689	0.020	----	19.5510		----			
5.4114	17460	0.029	----	19.5971		----			
5.5300	34039	0.028	0.965	19.7628	20:1 w9c	0.61	ECL deviates -0.010		
5.5619	15219	0.026	0.965	19.8073	20:1 w8c	0.27	ECL deviates -0.006		
5.6995	33800	0.023	0.966	19.9995	20:0	0.61	ECL deviates -0.001	Reference  0.001	
5.7530	2617	0.020	----	20.0734		----			
5.8025	4134	0.021	----	20.1417		----			
5.8327	13057	0.022	----	20.1833		----			
5.8740	737	0.011	----	20.2403		----			
5.9425	15490	0.030	----	20.3348		----			
5.9748	42925	0.027	----	20.3794		----			
6.0798	3825	0.015	----	20.5243		----			
6.0855	1217	0.005	----	20.5321		----	< min ar/ht		
6.1024	5998	0.018	----	20.5554		----			
6.1454	14353	0.036	----	20.6148		----	> max ar/ht		
6.2206	11367	0.031	----	20.7185		----			
6.2741	16715	0.021	0.968	20.7923	21:1 w8c	0.30	ECL deviates -0.006		
6.3312	23334	0.024	----	20.8712		----			
6.3922	17438	0.020	0.968	20.9553	21:1 w3c	0.31	ECL deviates  0.001		
6.4274	14845	0.035	0.968	21.0038	21:0	0.27	ECL deviates  0.004	Reference  0.004	
6.5042	6341	0.024	----	21.1098		----			
6.5463	3444	0.021	----	21.1678		----			
6.5933	9884	0.023	0.968	21.2327	22:5 w6c	0.18	ECL deviates -0.019		
6.6256	20108	0.025	----	21.2772		----			
6.6900	2969	0.023	----	21.3660		----			
6.7520	5800	0.035	0.968	21.4514	22:5 w3c	----	> max ar/ht		
6.8747	30881	0.036	0.968	21.6206	22:0 iso	----	> max ar/ht		
6.9290	1942	0.008	----	21.6955		----			
6.9481	5684	0.019	----	21.7218		----			
6.9844	8582	0.024	0.968	21.7719	22:1 w9c	0.15	ECL deviates -0.001		
7.0201	19671	0.036	0.968	21.8211	22:1 w8c	----	> max ar/ht		
7.1043	9169	0.021	0.967	21.9373	22:1 w3c	0.17	ECL deviates -0.010		
7.1475	48667	0.020	0.967	21.9968	22:0	0.88	ECL deviates -0.003	Reference -0.003	
7.1844	1397	0.007	----	22.0486		----			
7.2006	5475	0.025	----	22.0712		----			
7.2395	7351	0.035	----	22.1259		----			
7.3229	18702	0.026	----	22.2430		----			
7.3670	4097	0.023	----	22.3048		----			
7.4009	3284	0.021	----	22.3525		----			
7.4357	4417	0.029	----	22.4013		----			
7.4897	3634	0.024	0.965	22.4771	23:4 w6c	0.07	ECL deviates  0.006		
7.5330	4156	0.027	----	22.5379		----			
7.6072	11686	0.057	0.964	22.6420	23:3 w3c	----	> max ar/ht		
7.7038	11396	0.025	----	22.7777		----			
7.7651	8758	0.029	----	22.8638		----			
7.8045	15123	0.023	0.962	22.9191	23:1 w4c	0.27	ECL deviates -0.007		
7.8637	13239	0.023	0.961	23.0021	23:0	0.24	ECL deviates  0.002	Reference  0.001	
7.9061	4968	0.030	----	23.0623		----			
7.9645	2186	0.029	----	23.1450		----			
8.0345	2321	0.021	----	23.2442		----			
8.0715	11189	0.021	----	23.2966		----			
8.1110	820	0.017	----	23.3525		----			
8.1759	1234	0.024	----	23.4445		----			
8.2367	2832	0.033	----	23.5307		----			
8.3202	19763	0.030	0.953	23.6490	24:3 w3c	0.35	ECL deviates -0.006		
8.3764	12023	0.027	----	23.7286		----			
8.3980	1584	0.006	----	23.7592		----			
8.4058	10215	0.029	----	23.7703		----			
8.4846	6689	0.028	----	23.8820		----			
8.5158	2466	0.017	----	23.9262		----			
8.5670	33953	0.021	0.947	23.9988	24:0	0.60	ECL deviates -0.001	Reference -0.004	
8.6703	4199	0.036	----	24.1451		----	> max rt		
8.7567	6138	0.036	----	24.2676		----	> max rt		
8.7943	1554	0.018	----	24.3209		----	> max rt		
8.9239	13593	0.022	----	24.5046		----	> max rt		
9.0260	1285	0.024	----	24.6493		----	> max rt		
9.0758	705	0.015	----	24.7198		----	> max rt		
9.1578	3054	0.029	----	24.8361		----	> max rt		
9.2256	21833	0.020	----	24.9322		----	> max rt		
9.2547	3459	0.014	----	24.9734		----	> max rt		
9.4633	16338	0.025	----	25.2692		----	> max rt		

ECL Deviation: 0.006                            Reference ECL Shift: 0.004       Number Reference Peaks: 22
Total Response: 6351765                       Total Named: 5497535
Percent Named: 86.55%                         Total Amount: 5431973
Profile Comment:   Column Overload:  A peak's response is greater than 400000.0.  Dilute and re-run.

(No search libraries specified in method PLFAD1.)
