# Supplementary material for: Microbial regulation of soil carbon properties under nitrogen addition and plant inputs removal
Source: PeerJ. 2019 Jul 17;7:e7343. doi: 10.7717/peerj.7343 (PMC6642627; doi:10.7717/peerj.7343)
Supplement: File S1 — The raw data showed the soil microbial PLFAs files in the year of 2015 and 2016. Each file of rtf. represented the microbial PLFAs for each soil sample. In the Supplemental File, the Excel file named “Numbers” showed the plots names and the related rtf. file names. [file peerj-07-7343-s002.zip › supplementary files/2015/6.rtf]

Volume: DATA            File: E164203.63A        Samp Ctr: 8                  ID Number: 29303 
Type: Samp                   Bottle: 7                        Method: PLFAD1 
Created: 4/20/2016 12:03:50 PM 
Sample ID: 6 


RT	Response	Ar/Ht	RFact	ECL	Peak Name	Percent	Comment1	Comment2	
0.7144	1.895E+9	0.014	----	7.6555	SOLVENT PEAK	----	< min rt		
0.7876	5116	0.018	----	8.1321		----	< min rt		
0.8864	810	0.009	----	8.7753		----	< min rt		
0.9447	330	0.008	----	9.1601		----	< min rt		
1.1871	1857	0.011	----	10.7366		----			
1.2635	810	0.011	1.208	11.1724	10:0 2OH	0.02	ECL deviates -0.011		
1.3543	1257	0.015	1.170	11.6042	12:0 iso	0.04	ECL deviates -0.008		
1.3912	1273	0.013	----	11.7801		----			
1.4382	4573	0.014	1.138	12.0033	12:0	0.13	ECL deviates  0.003	Reference -0.001	
1.4962	2114	0.015	----	12.2114		----			
1.5611	1585	0.017	----	12.4440		----			
1.6071	4368	0.012	1.094	12.6087	13:0 iso	0.12	ECL deviates -0.004	Reference -0.008	
1.6346	2720	0.015	1.088	12.7072	13:0 anteiso	0.07	ECL deviates -0.002	Reference -0.006	
1.6926	965	0.020	1.075	12.9147	13:1 w5c	0.03	ECL deviates -0.005		
1.7171	1868	0.014	1.071	13.0026	13:0	0.05	ECL deviates  0.003	Reference -0.001	
1.7862	784	0.016	----	13.1952	12:0 2OH	----	ECL deviates  0.009		
1.8761	1687	0.014	----	13.4453		----			
1.9357	53706	0.013	1.038	13.6113	14:0 iso	1.38	ECL deviates -0.003	Reference -0.006	
1.9960	656	0.009	1.031	13.7790	14:1 w9c	0.02	ECL deviates  0.002		
2.0104	1211	0.011	----	13.8193		----			
2.0439	2328	0.012	1.025	13.9124	14:1 w5c	0.06	ECL deviates  0.001		
2.0760	47150	0.013	1.021	14.0018	14:0	1.19	ECL deviates  0.002	Reference -0.002	
2.1321	656	0.011	----	14.1284	14:0 iso 3OH	----	ECL deviates  0.004		
2.1610	2664	0.022	----	14.1933		----			
2.2129	1960	0.018	----	14.3102		----			
2.2701	58378	0.016	1.005	14.4389	15:1 iso w6c	1.45	ECL deviates  0.000		
2.2881	7921	0.010	1.003	14.4794	15:4 w3c	0.20	ECL deviates -0.011		
2.3103	18471	0.014	1.001	14.5293	15:1 anteiso w9c	0.46	ECL deviates -0.001		
2.3490	244360	0.014	0.999	14.6165	15:0 iso	6.04	ECL deviates -0.001	Reference -0.004	
2.3910	183433	0.013	0.996	14.7111	15:0 anteiso	4.52	ECL deviates  0.000	Reference -0.003	
2.4158	1042	0.008	0.994	14.7668	15:1 w9c	0.03	ECL deviates -0.004		
2.4558	8248	0.024	0.991	14.8568	15:1 w6c	0.20	ECL deviates -0.003		
2.5198	24273	0.015	0.987	15.0010	15:0	0.59	ECL deviates  0.001	Reference -0.002	
2.5479	7534	0.016	----	15.0545		----			
2.6111	1109	0.016	----	15.1744		----			
2.6404	2859	0.016	----	15.2299		----			
2.7266	7835	0.015	0.978	15.3935	16:1 w7c alcohol	0.19	ECL deviates -0.003		
2.7522	42136	0.020	0.976	15.4421	15:0 DMA	1.02	ECL deviates -0.008		
2.8131	76450	0.015	0.974	15.5577	16:0 N alcohol	1.84	ECL deviates  0.001		
2.8459	107258	0.016	0.973	15.6199	16:0 iso	2.58	ECL deviates  0.000	Reference -0.002	
2.8972	9058	0.013	0.971	15.7172	16:0 anteiso	0.22	ECL deviates  0.002	Reference  0.000	
2.9241	61396	0.017	0.970	15.7682	16:1 w9c	1.47	ECL deviates -0.007		
2.9540	413694	0.018	0.969	15.8249	16:1 w7c	9.92	Column Overload		
3.0006	134179	0.017	0.968	15.9133	16:1 w5c	3.21	ECL deviates  0.002		
3.0506	447774	0.016	0.966	16.0075	16:0	10.70	Column Overload		
3.0769	12708	0.014	----	16.0515		----			
3.0926	6422	0.014	----	16.0778		----			
3.1300	2572	0.015	0.964	16.1403	16:2 DMA	0.06	ECL deviates  0.002		
3.1652	7414	0.021	----	16.1990		----			
3.2017	3452	0.017	----	16.2601		----			
3.2374	2498	0.018	0.962	16.3196	16:1 w7c DMA	0.06	ECL deviates  0.010		
3.3000	236996	0.020	0.961	16.4244	16:0 10-methyl	5.63	ECL deviates  0.004		
3.3357	48257	0.018	0.960	16.4840	17:1 iso w9c	1.15	ECL deviates -0.014		
3.3619	30286	0.018	----	16.5277		----			
3.4181	59216	0.015	0.959	16.6216	17:0 iso	1.40	ECL deviates -0.002	Reference -0.004	
3.4757	70544	0.016	0.958	16.7179	17:0 anteiso	1.67	ECL deviates -0.002		
3.5205	45046	0.018	0.957	16.7928	17:1 w8c	1.07	ECL deviates -0.004		
3.5802	129080	0.019	0.957	16.8926	17:0 cyclo w7c	3.05	ECL deviates -0.001		
3.6444	19912	0.017	0.956	16.9998	17:0	0.47	ECL deviates  0.000	Reference -0.003	
3.6704	22491	0.016	0.956	17.0397	17:1 w7c 10-methyl	0.53	ECL deviates -0.004		
3.7121	5496	0.017	----	17.1032		----			
3.7474	2182	0.021	----	17.1570		----			
3.7978	3116	0.019	0.955	17.2336	16:0 2OH	0.07	ECL deviates -0.007		
3.8506	604	0.013	----	17.3142		----			
3.9088	27620	0.016	0.954	17.4028	17:0 10-methyl	0.65	ECL deviates -0.004		
3.9447	3163	0.014	0.954	17.4575	17:0 DMA	0.07	ECL deviates -0.001		
3.9674	7612	0.021	----	17.4921		----			
4.0420	37351	0.028	----	17.6057		----			
4.1170	73465	0.017	0.953	17.7199	18:2 w6c	1.73	ECL deviates -0.007		
4.1524	326950	0.018	0.953	17.7738	18:1 w9c	7.71	ECL deviates -0.001		
4.1894	486911	0.017	0.953	17.8303	18:1 w7c	11.48	Column Overload		
4.2426	63373	0.021	----	17.9113		----			
4.3013	77802	0.018	0.953	18.0007	18:0	1.83	ECL deviates  0.001	Reference -0.002	
4.3568	27407	0.018	0.953	18.0810	18:1 w7c 10-methyl	0.65	ECL deviates -0.004		
4.4085	8569	0.026	0.953	18.1557	18:2 DMA	0.20	ECL deviates -0.004		
4.4570	6304	0.030	0.953	18.2257	18:1 w9c DMA	0.15	ECL deviates -0.011		
4.5185	1821	0.017	----	18.3147		----			
4.5698	113963	0.019	0.954	18.3887	18:0 10-methyl	2.69	ECL deviates -0.006		
4.6406	3466	0.022	0.954	18.4910	19:4 w6c	0.08	ECL deviates  0.006		
4.6833	8525	0.024	0.954	18.5526	19:3 w6c	0.20	ECL deviates -0.007		
4.7387	1737	0.012	0.954	18.6327	19:0 iso	0.04	ECL deviates  0.003		
4.7526	3302	0.017	0.955	18.6528	19:3 w3c	0.08	ECL deviates -0.006		
4.8152	15151	0.022	----	18.7432		----			
4.8599	11795	0.020	0.955	18.8079	19:1 w8c	0.28	ECL deviates -0.003		
4.8978	21738	0.016	0.955	18.8626	19:0 cyclo w9c	0.51	ECL deviates -0.009		
4.9238	98453	0.017	0.955	18.9002	19:0 cyclo w7c	2.33	ECL deviates -0.010		
4.9943	78673	0.020	----	19.0021	19:0	----	ECL deviates  0.002		
5.0552	2554	0.016	----	19.0871		----			
5.1480	3099	0.018	----	19.2166		----			
5.1816	14445	0.020	----	19.2634		----			
5.2693	28624	0.029	0.958	19.3858	20:4 w6c	0.68	ECL deviates -0.018		
5.3225	8949	0.021	0.958	19.4600	20:5 w3c	0.21	ECL deviates -0.022		
5.3565	3750	0.018	----	19.5075		----			
5.3903	5759	0.019	----	19.5547		----			
5.4205	12714	0.024	----	19.5968		----			
5.5395	28725	0.026	0.960	19.7628	20:1 w9c	0.68	ECL deviates -0.010		
5.5698	10923	0.023	0.960	19.8051	20:1 w8c	0.26	ECL deviates -0.008		
5.6221	901	0.015	----	19.8781		----			
5.7095	24904	0.023	0.961	20.0001	20:0	0.59	ECL deviates  0.000	Reference -0.003	
5.7658	1425	0.020	----	20.0776		----			
5.8116	2658	0.017	----	20.1406		----			
5.8434	6629	0.020	----	20.1843		----			
5.9273	3281	0.017	----	20.2998		----			
5.9550	5478	0.018	----	20.3378		----			
5.9865	20523	0.020	----	20.3812		----			
6.0189	2671	0.016	----	20.4258		----			
6.0576	901	0.014	----	20.4790		----			
6.1059	2777	0.021	----	20.5455		----			
6.1111	1562	0.012	----	20.5526		----			
6.1594	8463	0.031	----	20.6191		----			
6.2245	4437	0.029	----	20.7086		----			
6.2866	11986	0.019	0.965	20.7941	21:1 w8c	0.29	ECL deviates -0.004		
6.3450	9501	0.022	----	20.8744		----			
6.4023	18430	0.021	0.966	20.9533	21:1 w3c	0.44	ECL deviates -0.001		
6.4374	8282	0.023	0.966	21.0015	21:0	0.20	ECL deviates  0.002	Reference -0.002	
6.5179	3485	0.020	----	21.1128		----			
6.6045	6389	0.026	0.967	21.2325	22:5 w6c	0.15	ECL deviates -0.019		
6.6366	11449	0.023	----	21.2768		----			
6.7037	895	0.018	----	21.3695		----			
6.7632	812	0.015	0.968	21.4518	22:5 w3c	----	Below has same name		
6.7830	962	0.018	----	21.4791	22:5 w3c	----	Above has same name		
6.8861	15930	0.026	0.968	21.6216	22:0 iso	0.38	ECL deviates  0.004		
6.9628	3257	0.025	0.968	21.7276	22:2 w6c	0.08	ECL deviates -0.011		
6.9986	2918	0.019	0.968	21.7770	22:1 w9c	0.07	ECL deviates  0.004		
7.0291	6526	0.030	0.968	21.8192	22:1 w8c	0.16	ECL deviates  0.006		
7.1137	4946	0.017	0.969	21.9361	22:1 w3c	0.12	ECL deviates -0.011		
7.1575	30556	0.018	0.969	21.9967	22:0	0.73	ECL deviates -0.003	Reference -0.007	
7.2217	1278	0.020	----	22.0865		----			
7.2561	1114	0.023	----	22.1347		----			
7.3327	9599	0.019	----	22.2420		----			
7.5472	971	0.018	----	22.5425		----			
7.6116	3483	0.035	----	22.6328		----	> max ar/ht		
7.7139	4211	0.019	----	22.7761		----			
7.7730	3580	0.023	----	22.8590		----			
7.8169	10101	0.019	0.967	22.9205	23:1 w4c	0.24	ECL deviates -0.006		
7.8746	6550	0.019	0.966	23.0013	23:0	0.16	ECL deviates  0.001	Reference -0.004	
7.9177	2105	0.024	----	23.0624		----			
8.0833	8151	0.019	----	23.2976		----			
8.2943	895	0.016	0.961	23.5975	24:3 w6c	0.02	ECL deviates  0.007		
8.3326	10631	0.024	0.960	23.6519	24:3 w3c	0.25	ECL deviates -0.003		
8.3894	3395	0.023	----	23.7325		----			
8.4224	3711	0.027	0.959	23.7795	24:1 w9c	0.09	ECL deviates -0.007		
8.4972	2993	0.027	----	23.8857		----			
8.5328	1016	0.016	0.957	23.9363	24:1 w3c	0.02	ECL deviates -0.013		
8.5778	24768	0.022	0.956	24.0002	24:0	0.59	ECL deviates  0.000	Reference -0.006	
8.6808	807	0.016	----	24.1466		----	> max rt		
8.9332	13808	0.018	----	24.5051		----	> max rt		
9.0338	978	0.023	----	24.6481		----	> max rt		
9.1667	880	0.018	----	24.8370		----	> max rt		
9.2363	23621	0.023	----	24.9358		----	> max rt		
9.4746	10663	0.021	----	25.2744		----	> max rt		

ECL Deviation: 0.007                            Reference ECL Shift: 0.004       Number Reference Peaks: 19
Total Response: 4567956                       Total Named: 4176223
Percent Named: 91.42%                         Total Amount: 4043924
Profile Comment:   Column Overload:  A peak's response is greater than 400000.0.  Dilute and re-run.

(No search libraries specified in method PLFAD1.)
