# Supplementary material for: Microbial regulation of soil carbon properties under nitrogen addition and plant inputs removal
Source: PeerJ. 2019 Jul 17;7:e7343. doi: 10.7717/peerj.7343 (PMC6642627; doi:10.7717/peerj.7343)
Supplement: File S1 — The raw data showed the soil microbial PLFAs files in the year of 2015 and 2016. Each file of rtf. represented the microbial PLFAs for each soil sample. In the Supplemental File, the Excel file named “Numbers” showed the plots names and the related rtf. file names. [file peerj-07-7343-s002.zip › supplementary files/2015/7.rtf]

Volume: DATA            File: E164203.63A        Samp Ctr: 9                  ID Number: 29304 
Type: Samp                   Bottle: 8                        Method: PLFAD1 
Created: 4/20/2016 12:32:33 PM 
Sample ID: 7 


RT	Response	Ar/Ht	RFact	ECL	Peak Name	Percent	Comment1	Comment2	
0.7145	1.906E+9	0.015	----	7.6515	SOLVENT PEAK	----	< min rt		
0.7877	4740	0.018	----	8.1282		----	< min rt		
0.8863	960	0.009	----	8.7765		----	< min rt		
0.9463	622	0.012	----	9.1670		----	< min rt		
1.1878	2069	0.012	----	10.7383		----			
1.2645	942	0.014	1.208	11.1745	10:0 2OH	0.03	ECL deviates -0.009		
1.3545	1209	0.019	1.170	11.6029	12:0 iso	0.04	ECL deviates -0.009	Reference -0.012	
1.3671	542	0.009	----	11.6626		----			
1.3922	1684	0.017	----	11.7820		----			
1.4393	4296	0.015	1.138	12.0054	12:0	0.14	ECL deviates  0.005	Reference  0.003	
1.4967	2448	0.016	----	12.2108		----			
1.5247	489	0.012	----	12.3113		----			
1.5614	1580	0.016	----	12.4427		----			
1.6080	3525	0.012	1.094	12.6097	13:0 iso	0.11	ECL deviates -0.003	Reference -0.005	
1.6367	2788	0.015	1.088	12.7127	13:0 anteiso	0.09	ECL deviates  0.003	Reference  0.001	
1.6932	1267	0.016	1.075	12.9150	13:1 w5c	0.04	ECL deviates -0.005		
1.7180	1825	0.013	1.071	13.0035	13:0	0.05	ECL deviates  0.003	Reference  0.001	
1.7862	866	0.019	----	13.1935	12:0 2OH	----	ECL deviates  0.007		
1.8773	1952	0.018	----	13.4469		----			
1.9089	408	0.008	----	13.5351		----			
1.9363	46811	0.013	1.038	13.6114	14:0 iso	1.36	ECL deviates -0.003	Reference -0.005	
1.9758	1036	0.012	1.033	13.7212	14:0 anteiso	0.03	ECL deviates  0.005	Reference  0.003	
1.9968	984	0.009	1.031	13.7799	14:1 w9c	0.03	ECL deviates  0.002		
2.0113	2027	0.013	----	13.8201		----			
2.0442	2894	0.012	1.025	13.9119	14:1 w5c	0.08	ECL deviates  0.001		
2.0762	43287	0.014	1.021	14.0009	14:0	1.24	ECL deviates  0.001	Reference -0.001	
2.1032	606	0.013	----	14.0620		----			
2.1315	1151	0.013	----	14.1257	14:0 iso 3OH	----	ECL deviates  0.001		
2.1570	2497	0.022	----	14.1830		----			
2.2158	2052	0.021	----	14.3154		----			
2.2701	48220	0.017	1.005	14.4376	15:1 iso w6c	1.36	ECL deviates -0.001		
2.2886	8125	0.012	1.003	14.4793	15:4 w3c	0.23	ECL deviates -0.011		
2.3102	12985	0.014	1.001	14.5281	15:1 anteiso w9c	0.37	ECL deviates -0.002		
2.3493	215126	0.014	0.999	14.6160	15:0 iso	6.03	ECL deviates -0.001	Reference -0.003	
2.3909	160590	0.014	0.996	14.7096	15:0 anteiso	4.49	ECL deviates -0.001	Reference -0.003	
2.4557	7877	0.024	0.991	14.8557	15:1 w6c	0.22	ECL deviates -0.004		
2.5199	23333	0.015	0.987	15.0001	15:0	0.65	ECL deviates  0.000	Reference -0.002	
2.5479	8003	0.016	----	15.0537		----			
2.6104	1696	0.017	----	15.1722		----			
2.6395	2151	0.017	----	15.2274		----			
2.7265	6865	0.014	0.978	15.3926	16:1 w7c alcohol	0.19	ECL deviates -0.004		
2.7528	36157	0.021	0.976	15.4426	15:0 DMA	0.99	ECL deviates -0.008		
2.8125	83382	0.015	0.974	15.5559	16:0 N alcohol	2.28	ECL deviates -0.001		
2.8455	94399	0.015	0.973	15.6184	16:0 iso	2.58	ECL deviates -0.001	Reference -0.003	
2.8970	8127	0.015	0.971	15.7163	16:0 anteiso	0.22	ECL deviates  0.001	Reference -0.001	
2.9223	52704	0.017	0.970	15.7643	16:1 w9c	1.44	ECL deviates -0.011		
2.9528	391875	0.017	0.969	15.8223	16:1 w7c	10.66	ECL deviates -0.002		
2.9996	117305	0.016	0.968	15.9111	16:1 w5c	3.19	ECL deviates  0.000		
3.0490	396240	0.015	0.966	16.0045	16:0	10.75	ECL deviates  0.005	Reference  0.002	
3.0761	20062	0.020	----	16.0499		----			
3.1295	2360	0.015	0.964	16.1391	16:2 DMA	0.06	ECL deviates  0.001		
3.1651	6227	0.021	----	16.1986		----			
3.2000	3406	0.018	----	16.2570		----			
3.2379	1937	0.020	0.962	16.3202	16:1 w7c DMA	0.05	ECL deviates  0.010		
3.2986	221850	0.018	0.961	16.4218	16:0 10-methyl	5.98	ECL deviates  0.002		
3.3338	48807	0.017	----	16.4805		----			
3.3618	27140	0.018	----	16.5273		----			
3.4174	51952	0.016	0.959	16.6204	17:0 iso	1.40	ECL deviates -0.003	Reference -0.006	
3.4743	63341	0.017	0.958	16.7155	17:0 anteiso	1.70	ECL deviates -0.005		
3.5196	38794	0.018	0.957	16.7912	17:1 w8c	1.04	ECL deviates -0.006		
3.5784	124958	0.018	0.957	16.8896	17:0 cyclo w7c	3.36	ECL deviates -0.004		
3.6439	17091	0.018	0.956	16.9991	17:0	0.46	ECL deviates -0.001	Reference -0.003	
3.6689	21876	0.016	0.956	17.0374	17:1 w7c 10-methyl	0.59	ECL deviates -0.006		
3.7131	5708	0.018	----	17.1048		----			
3.7476	1933	0.021	----	17.1574		----			
3.7970	2789	0.018	0.955	17.2326	16:0 2OH	0.07	ECL deviates -0.008		
3.8528	678	0.014	----	17.3178		----			
3.9081	24408	0.018	0.954	17.4020	17:0 10-methyl	0.65	ECL deviates -0.005		
3.9433	3234	0.014	0.954	17.4556	17:0 DMA	0.09	ECL deviates -0.002		
3.9650	6846	0.022	----	17.4887		----			
4.0405	32704	0.029	----	17.6039		----			
4.1151	70623	0.018	0.953	17.7175	18:2 w6c	1.89	ECL deviates -0.010		
4.1486	267394	0.018	0.953	17.7686	18:1 w9c	7.16	ECL deviates -0.006		
4.1863	431922	0.018	0.953	17.8260	18:1 w7c	11.56	Column Overload		
4.2416	48691	0.021	----	17.9104		----			
4.2990	65227	0.018	0.953	17.9978	18:0	1.75	ECL deviates -0.002	Reference -0.005	
4.3544	22344	0.018	0.953	18.0782	18:1 w7c 10-methyl	0.60	ECL deviates -0.007		
4.4072	8225	0.026	0.953	18.1545	18:2 DMA	0.22	ECL deviates -0.005		
4.4600	6345	0.031	0.953	18.2308	18:1 w9c DMA	0.17	ECL deviates -0.006		
4.5175	2129	0.018	----	18.3139		----			
4.5683	103672	0.019	0.954	18.3874	18:0 10-methyl	2.78	ECL deviates -0.008		
4.6356	2740	0.019	0.954	18.4847	19:4 w6c	0.07	ECL deviates  0.000		
4.6817	7821	0.023	0.954	18.5514	19:3 w6c	0.21	ECL deviates -0.009		
4.7510	4564	0.026	0.955	18.6515	19:3 w3c	0.12	ECL deviates -0.007		
4.8142	13305	0.021	----	18.7428		----			
4.8597	12014	0.020	0.955	18.8087	19:1 w8c	0.32	ECL deviates -0.002		
4.8966	18147	0.016	----	18.8620		----			
4.9243	102623	0.020	0.955	18.9021	19:0 cyclo w7c	2.75	ECL deviates -0.008		
4.9940	85211	0.016	----	19.0029	19:0	----	ECL deviates  0.003		
5.0551	3130	0.021	----	19.0882		----			
5.1478	2518	0.019	----	19.2176		----			
5.1797	9902	0.019	----	19.2621		----			
5.2668	22107	0.030	----	19.3837		----			
5.3211	6578	0.018	0.958	19.4595	20:5 w3c	0.18	ECL deviates -0.023		
5.3563	2435	0.016	----	19.5086		----			
5.3879	5683	0.020	----	19.5528		----			
5.4211	11666	0.024	----	19.5991		----			
5.5393	25833	0.027	0.960	19.7642	20:1 w9c	0.70	ECL deviates -0.008		
5.5684	10239	0.023	0.960	19.8048	20:1 w8c	0.28	ECL deviates -0.008		
5.6215	982	0.015	----	19.8790		----			
5.6589	742	0.016	0.961	19.9312	20:1 w4c	0.02	ECL deviates  0.000		
5.7081	22806	0.022	0.961	19.9999	20:0	0.62	ECL deviates  0.000	Reference -0.005	
5.7640	1098	0.020	----	20.0768		----			
5.8114	3038	0.018	----	20.1421		----			
5.8423	6496	0.019	----	20.1846		----			
5.8793	665	0.015	----	20.2357		----			
5.9563	8104	0.028	----	20.3416		----			
5.9850	24919	0.023	----	20.3811		----			
6.0548	1380	0.019	----	20.4772		----			
6.0886	1650	0.014	----	20.5237		----			
6.1091	2454	0.019	----	20.5520		----			
6.1569	8868	0.028	----	20.6178		----			
6.2177	4857	0.030	----	20.7015		----			
6.2821	13630	0.018	0.965	20.7901	21:1 w8c	0.37	ECL deviates -0.008		
6.3419	9789	0.025	----	20.8725		----			
6.3992	19870	0.020	0.966	20.9513	21:1 w3c	0.54	ECL deviates -0.003		
6.4353	8771	0.025	0.966	21.0010	21:0	0.24	ECL deviates  0.001	Reference -0.005	
6.5150	4638	0.022	----	21.1112		----			
6.5609	1550	0.019	----	21.1747		----			
6.6013	4230	0.022	0.967	21.2306	22:5 w6c	0.11	ECL deviates -0.021		
6.6346	8218	0.024	----	21.2765		----			
6.6994	1300	0.021	----	21.3662		----			
6.8821	15947	0.031	0.968	21.6188	22:0 iso	0.43	ECL deviates  0.001		
6.9575	2686	0.020	----	21.7230		----			
6.9949	4142	0.022	0.968	21.7747	22:1 w9c	0.11	ECL deviates  0.002		
7.0294	7034	0.028	0.968	21.8224	22:1 w8c	0.19	ECL deviates  0.009		
7.1115	5608	0.020	0.969	21.9359	22:1 w3c	0.15	ECL deviates -0.011		
7.1559	23765	0.019	0.969	21.9973	22:0	0.65	ECL deviates -0.003	Reference -0.010	
7.2182	1924	0.024	----	22.0845		----			
7.2496	2042	0.028	----	22.1285		----			
7.3321	12204	0.022	----	22.2443		----			
7.3863	1978	0.026	----	22.3202		----			
7.4480	2229	0.025	----	22.4068		----			
7.4995	1456	0.026	0.968	22.4790	23:4 w6c	0.04	ECL deviates  0.008		
7.5426	1433	0.024	----	22.5393		----			
7.6075	5027	0.038	----	22.6302		----	> max ar/ht		
7.7098	6799	0.022	----	22.7737		----			
7.7706	2758	0.024	----	22.8589		----			
7.8140	11246	0.021	0.967	22.9198	23:1 w4c	0.31	ECL deviates -0.007		
7.8718	5731	0.019	0.966	23.0008	23:0	0.16	ECL deviates  0.001	Reference -0.008	
7.9168	2230	0.027	----	23.0647		----			
8.0792	6745	0.019	----	23.2955		----			
8.2930	963	0.018	0.961	23.5995	24:3 w6c	0.03	ECL deviates  0.009		
8.3305	9294	0.023	0.960	23.6528	24:3 w3c	0.25	ECL deviates -0.002		
8.3881	2886	0.020	----	23.7346		----			
8.4214	3912	0.023	0.959	23.7820	24:1 w9c	0.11	ECL deviates -0.005		
8.4953	3823	0.030	----	23.8871		----			
8.5282	1339	0.018	----	23.9338		----			
8.5757	22301	0.020	0.956	24.0014	24:0	0.60	ECL deviates  0.001	Reference -0.009	
8.6808	1956	0.027	----	24.1508		----	> max rt		
8.9324	18021	0.018	----	24.5085		----	> max rt		
9.2331	25159	0.024	----	24.9359		----	> max rt		
9.4730	9796	0.022	----	25.2771		----	> max rt		

ECL Deviation: 0.006                            Reference ECL Shift: 0.005       Number Reference Peaks: 22
Total Response: 4147908                       Total Named: 3678373
Percent Named: 88.68%                         Total Amount: 3561991
Profile Comment:   Column Overload:  A peak's response is greater than 400000.0.  Dilute and re-run.

(No search libraries specified in method PLFAD1.)
