# Supplementary material for: Microbial regulation of soil carbon properties under nitrogen addition and plant inputs removal
Source: PeerJ. 2019 Jul 17;7:e7343. doi: 10.7717/peerj.7343 (PMC6642627; doi:10.7717/peerj.7343)
Supplement: File S1 — The raw data showed the soil microbial PLFAs files in the year of 2015 and 2016. Each file of rtf. represented the microbial PLFAs for each soil sample. In the Supplemental File, the Excel file named “Numbers” showed the plots names and the related rtf. file names. [file peerj-07-7343-s002.zip › supplementary files/2015/8.rtf]

Volume: DATA            File: E164203.63A        Samp Ctr: 10                ID Number: 29305 
Type: Samp                   Bottle: 9                        Method: PLFAD1 
Created: 4/20/2016 1:01:02 PM 
Sample ID: 8 


RT	Response	Ar/Ht	RFact	ECL	Peak Name	Percent	Comment1	Comment2	
0.7145	1.89E+9	0.015	----	7.6556	SOLVENT PEAK	----	< min rt		
0.7873	4270	0.018	----	8.1301		----	< min rt		
0.8867	1510	0.011	----	8.7774		----	< min rt		
0.9464	509	0.010	----	9.1714		----	< min rt		
1.1875	2018	0.013	----	10.7400		----			
1.2631	1231	0.013	----	11.1711		----			
1.3534	1007	0.015	1.170	11.6007	12:0 iso	0.03	ECL deviates -0.011		
1.3672	358	0.008	----	11.6663		----			
1.3920	1428	0.012	----	11.7840		----			
1.4384	3873	0.014	1.138	12.0051	12:0	0.10	ECL deviates  0.005	Reference  0.000	
1.4959	1756	0.011	----	12.2109		----			
1.5616	1212	0.016	----	12.4461		----			
1.6070	4280	0.012	1.094	12.6090	13:0 iso	0.11	ECL deviates -0.003	Reference -0.008	
1.6343	2558	0.015	1.088	12.7068	13:0 anteiso	0.06	ECL deviates -0.003	Reference -0.007	
1.6924	1221	0.016	1.075	12.9147	13:1 w5c	0.03	ECL deviates -0.005		
1.7168	1699	0.012	1.071	13.0020	13:0	0.04	ECL deviates  0.002	Reference -0.003	
1.7851	863	0.016	----	13.1928	12:0 2OH	----	ECL deviates  0.007		
1.8760	1811	0.016	----	13.4460		----			
1.9353	51706	0.013	1.038	13.6110	14:0 iso	1.20	ECL deviates -0.003	Reference -0.007	
1.9951	556	0.007	1.031	13.7776	14:1 w9c	0.01	ECL deviates  0.000		
2.0108	1450	0.011	----	13.8212		----			
2.0429	2965	0.012	1.025	13.9107	14:1 w5c	0.07	ECL deviates  0.000		
2.0752	51152	0.013	1.021	14.0005	14:0	1.17	ECL deviates  0.001	Reference -0.004	
2.1315	665	0.010	----	14.1278	14:0 iso 3OH	----	ECL deviates  0.003		
2.1577	2783	0.022	----	14.1870		----			
2.2206	1624	0.020	----	14.3284		----			
2.2697	68169	0.017	1.005	14.4390	15:1 iso w6c	1.54	ECL deviates  0.000		
2.2874	9641	0.011	1.003	14.4788	15:4 w3c	0.22	ECL deviates -0.011		
2.3094	15781	0.014	1.001	14.5284	15:1 anteiso w9c	0.35	ECL deviates -0.002		
2.3491	294943	0.013	0.999	14.6176	15:0 iso	6.61	ECL deviates  0.001	Reference -0.003	
2.3903	207753	0.013	0.996	14.7105	15:0 anteiso	4.64	ECL deviates  0.000	Reference -0.004	
2.4156	1274	0.011	0.994	14.7675	15:1 w9c	0.03	ECL deviates -0.003		
2.4555	7990	0.021	0.991	14.8574	15:1 w6c	0.18	ECL deviates -0.003		
2.4751	999	0.009	0.990	14.9014	15:1 w5c	0.02	ECL deviates -0.011		
2.5191	27257	0.015	0.987	15.0005	15:0	0.60	ECL deviates  0.000	Reference -0.003	
2.5474	8732	0.018	----	15.0547		----			
2.6101	1039	0.013	----	15.1737		----			
2.6400	2884	0.016	----	15.2303		----			
2.7260	8033	0.015	0.978	15.3936	16:1 w7c alcohol	0.18	ECL deviates -0.003		
2.7513	44014	0.020	0.976	15.4415	15:0 DMA	0.96	ECL deviates -0.009		
2.8125	77445	0.016	0.974	15.5578	16:0 N alcohol	1.69	ECL deviates  0.001		
2.8451	112163	0.015	0.973	15.6196	16:0 iso	2.45	ECL deviates  0.000	Reference -0.004	
2.8954	9796	0.015	0.971	15.7150	16:0 anteiso	0.21	ECL deviates  0.000	Reference -0.004	
2.9224	67203	0.017	0.970	15.7663	16:1 w9c	1.46	ECL deviates -0.009		
2.9531	462372	0.017	0.969	15.8245	16:1 w7c	10.05	Column Overload		
2.9999	162377	0.016	0.968	15.9133	16:1 w5c	3.53	ECL deviates  0.002		
3.0499	520196	0.015	0.966	16.0078	16:0	11.28	Column Overload		
3.0761	19135	0.019	----	16.0516		----			
3.1291	3568	0.016	0.964	16.1402	16:2 DMA	0.08	ECL deviates  0.002		
3.1643	8973	0.021	----	16.1989		----			
3.2024	3911	0.017	----	16.2626		----			
3.2361	2391	0.021	0.962	16.3189	16:1 w7c DMA	0.05	ECL deviates  0.009		
3.2989	277558	0.020	0.961	16.4240	16:0 10-methyl	5.98	ECL deviates  0.004		
3.3350	53820	0.018	0.960	16.4843	17:1 iso w9c	1.16	ECL deviates -0.014		
3.3630	31823	0.017	0.960	16.5310	17:1 anteiso w9c	0.69	ECL deviates -0.005		
3.4181	71550	0.016	0.959	16.6231	17:0 iso	1.54	ECL deviates -0.001	Reference -0.004	
3.4757	76309	0.017	0.958	16.7193	17:0 anteiso	1.64	ECL deviates -0.001		
3.5200	48038	0.018	0.957	16.7934	17:1 w8c	1.03	ECL deviates -0.004		
3.5788	152624	0.018	0.957	16.8917	17:0 cyclo w7c	3.28	ECL deviates -0.002		
3.6443	23147	0.019	0.956	17.0012	17:0	0.50	ECL deviates  0.001	Reference -0.003	
3.6702	22743	0.016	0.956	17.0408	17:1 w7c 10-methyl	0.49	ECL deviates -0.003		
3.7124	6351	0.018	----	17.1051		----			
3.7477	2153	0.018	----	17.1589		----			
3.7980	4197	0.021	0.955	17.2355	16:0 2OH	0.09	ECL deviates -0.005		
3.8529	801	0.015	----	17.3192		----			
3.9075	30747	0.017	0.954	17.4024	17:0 10-methyl	0.66	ECL deviates -0.005		
3.9447	3201	0.013	0.954	17.4591	17:0 DMA	0.07	ECL deviates  0.001		
3.9672	11251	0.023	----	17.4933		----			
4.0404	39204	0.029	----	17.6049		----			
4.1162	88341	0.018	0.953	17.7203	18:2 w6c	1.89	ECL deviates -0.007		
4.1507	308489	0.019	0.953	17.7729	18:1 w9c	6.60	ECL deviates -0.002		
4.1886	543455	0.017	0.953	17.8306	18:1 w7c	11.62	Column Overload		
4.2419	70302	0.022	----	17.9118		----			
4.2997	83649	0.018	0.953	18.0000	18:0	1.79	ECL deviates  0.000	Reference -0.004	
4.3552	31420	0.019	0.953	18.0804	18:1 w7c 10-methyl	0.67	ECL deviates -0.005		
4.4061	11544	0.026	0.953	18.1540	18:2 DMA	0.25	ECL deviates -0.006		
4.4569	7017	0.033	0.953	18.2273	18:1 w9c DMA	0.15	ECL deviates -0.010		
4.5156	1892	0.017	----	18.3122		----			
4.5675	112407	0.021	0.954	18.3872	18:0 10-methyl	2.41	ECL deviates -0.008		
4.6380	3651	0.021	0.954	18.4891	19:4 w6c	0.08	ECL deviates  0.004		
4.6821	8068	0.023	0.954	18.5527	19:3 w6c	0.17	ECL deviates -0.007		
4.7513	6417	0.026	0.955	18.6528	19:3 w3c	0.14	ECL deviates -0.005		
4.8156	13956	0.022	----	18.7457		----			
4.8583	12384	0.022	0.955	18.8074	19:1 w8c	0.27	ECL deviates -0.004		
4.9002	20316	0.016	0.955	18.8679	19:0 cyclo w9c	0.44	ECL deviates -0.004		
4.9235	117569	0.020	0.955	18.9016	19:0 cyclo w7c	2.52	ECL deviates -0.008		
4.9932	87961	0.018	----	19.0023	19:0	----	ECL deviates  0.002		
5.0534	2428	0.015	----	19.0865		----			
5.1461	2724	0.018	----	19.2158		----			
5.1803	11536	0.018	----	19.2635		----			
5.2676	33328	0.031	----	19.3853		----			
5.3214	10570	0.019	0.958	19.4604	20:5 w3c	----	Below has same name		
5.3542	3072	0.017	----	19.5062	20:5 w3c	----	Above has same name		
5.3866	7256	0.020	----	19.5515		----			
5.4195	14011	0.025	----	19.5973		----			
5.5392	29058	0.026	0.960	19.7645	20:1 w9c	0.63	ECL deviates -0.008		
5.5686	12357	0.024	0.960	19.8054	20:1 w8c	0.27	ECL deviates -0.008		
5.6574	730	0.014	0.961	19.9294	20:1 w4c	0.02	ECL deviates -0.002		
5.7087	26702	0.021	0.961	20.0010	20:0	0.58	ECL deviates  0.001	Reference -0.004	
5.7621	1265	0.017	----	20.0745		----			
5.8107	3056	0.018	----	20.1414		----			
5.8420	7636	0.018	----	20.1844		----			
5.9208	4086	0.020	----	20.2928		----			
5.9529	4310	0.016	----	20.3370		----			
5.9844	23391	0.024	----	20.3804		----			
6.0583	1247	0.018	----	20.4821		----			
6.1108	3745	0.029	----	20.5543		----			
6.1578	7622	0.034	----	20.6190		----			
6.2276	4697	0.032	----	20.7151		----			
6.2835	12122	0.018	0.965	20.7920	21:1 w8c	0.26	ECL deviates -0.006		
6.3414	12547	0.024	----	20.8717		----			
6.3999	18666	0.020	0.966	20.9521	21:1 w3c	0.40	ECL deviates -0.002		
6.4346	8975	0.025	0.966	20.9999	21:0	0.19	ECL deviates  0.000	Reference -0.006	
6.5157	3768	0.020	----	21.1120		----			
6.5601	1460	0.019	----	21.1733		----			
6.6017	4542	0.020	0.967	21.2308	22:5 w6c	0.10	ECL deviates -0.021		
6.6353	9987	0.025	----	21.2772		----			
6.6993	907	0.016	----	21.3657		----			
6.8837	13536	0.024	0.968	21.6205	22:0 iso	0.29	ECL deviates  0.003		
6.9647	2152	0.020	0.968	21.7325	22:2 w6c	0.05	ECL deviates -0.006		
6.9935	3236	0.022	0.968	21.7724	22:1 w9c	0.07	ECL deviates -0.001		
7.0292	4927	0.026	0.968	21.8216	22:1 w8c	0.11	ECL deviates  0.008		
7.1149	5075	0.019	0.969	21.9400	22:1 w3c	0.11	ECL deviates -0.007		
7.1581	28586	0.020	0.969	21.9998	22:0	0.62	ECL deviates  0.000	Reference -0.007	
7.2152	1114	0.021	----	22.0797		----			
7.2484	966	0.022	----	22.1263		----			
7.3309	9686	0.020	----	22.2418		----			
7.6094	2741	0.028	----	22.6320		----			
7.7113	3895	0.020	----	22.7748		----			
7.7692	1998	0.021	----	22.8560		----			
7.8141	9977	0.019	0.967	22.9189	23:1 w4c	0.22	ECL deviates -0.008		
7.8722	7044	0.019	0.966	23.0004	23:0	0.15	ECL deviates  0.000	Reference -0.007	
7.9142	1580	0.022	----	23.0600		----			
8.0784	7448	0.018	----	23.2931		----			
8.2910	895	0.015	0.961	23.5952	24:3 w6c	0.02	ECL deviates  0.005		
8.3280	10097	0.022	0.960	23.6478	24:3 w3c	0.22	ECL deviates -0.007		
8.3889	2455	0.020	----	23.7343		----			
8.4209	4246	0.022	0.959	23.7798	24:1 w9c	0.09	ECL deviates -0.007		
8.4956	2286	0.028	----	23.8860		----			
8.5306	820	0.016	0.957	23.9358	24:1 w3c	0.02	ECL deviates -0.013		
8.5754	25456	0.019	0.956	23.9994	24:0	0.55	ECL deviates -0.001	Reference -0.009	
8.6800	865	0.018	----	24.1479		----	> max rt		
8.9315	17325	0.019	----	24.5053		----	> max rt		
9.2363	24490	0.021	----	24.9385		----	> max rt		
9.4723	9595	0.021	----	25.2738		----	> max rt		

ECL Deviation: 0.007                            Reference ECL Shift: 0.005       Number Reference Peaks: 19
Total Response: 5019134                       Total Named: 4604062
Percent Named: 91.73%                         Total Amount: 4467741
Profile Comment:   Column Overload:  A peak's response is greater than 400000.0.  Dilute and re-run.

(No search libraries specified in method PLFAD1.)
