# Supplementary material for: Microbial regulation of soil carbon properties under nitrogen addition and plant inputs removal
Source: PeerJ. 2019 Jul 17;7:e7343. doi: 10.7717/peerj.7343 (PMC6642627; doi:10.7717/peerj.7343)
Supplement: File S1 — The raw data showed the soil microbial PLFAs files in the year of 2015 and 2016. Each file of rtf. represented the microbial PLFAs for each soil sample. In the Supplemental File, the Excel file named “Numbers” showed the plots names and the related rtf. file names. [file peerj-07-7343-s002.zip › supplementary files/2016/52.rtf]

Volume: DATA            File: E17C203.64A       Samp Ctr: 5                   ID Number: 5025 
Type: Samp                   Bottle: 16                      Method: PLFAD1 
Created: 12/20/2017 10:26:09 AM 
Sample ID: 52 


RT	Response	Ar/Ht	RFact	ECL	Peak Name	Percent	Comment1	Comment2	
0.7659	1.679E+9	0.016	----	7.6913	SOLVENT PEAK	----	< min rt		
1.8122	692	0.012	1.030	12.7185	13:0 anteiso	0.13	ECL deviates  0.009	Reference  0.016	
1.9890	1040	0.017	----	13.2229		----			
2.1407	3760	0.016	1.043	13.6050	14:0 iso	0.72	ECL deviates -0.009	Reference -0.006	
2.1861	1048	0.014	1.043	13.7194	14:0 anteiso	0.20	ECL deviates  0.003	Reference  0.006	
2.2167	727	0.013	1.044	13.7964	14:1 w8c	0.14	ECL deviates -0.005		
2.2706	759	0.014	----	13.9322		----			
2.2955	3890	0.015	1.045	13.9949	14:0	0.74	ECL deviates -0.005	Reference -0.004	
2.3578	1155	0.013	----	14.1248	14:0 iso 3OH	----	ECL deviates  0.000		
2.4563	864	0.015	----	14.3286		----			
2.5092	6018	0.018	1.046	14.4382	15:1 iso w6c	1.15	ECL deviates -0.001		
2.5322	862	0.012	1.046	14.4858	15:4 w3c	0.16	ECL deviates -0.004		
2.5526	850	0.011	1.046	14.5282	15:1 anteiso w9c	0.16	ECL deviates -0.002		
2.5939	26053	0.015	1.046	14.6136	15:0 iso	4.97	ECL deviates -0.003	Reference -0.004	
2.6400	17467	0.015	1.046	14.7092	15:0 anteiso	3.33	ECL deviates -0.002	Reference -0.003	
2.7066	1852	0.016	1.045	14.8469	15:1 w7c	0.35	ECL deviates  0.010		
2.7801	2254	0.015	1.045	14.9992	15:0	0.43	ECL deviates -0.001	Reference -0.003	
2.8113	1003	0.015	----	15.0547		----			
3.0335	4172	0.019	1.042	15.4475	15:0 DMA	0.79	ECL deviates -0.003		
3.1022	12057	0.016	1.041	15.5688	16:3 w6c	2.29	ECL deviates -0.007		
3.1316	10693	0.016	1.040	15.6209	16:0 iso	2.03	ECL deviates  0.001	Reference -0.003	
3.1886	2098	0.014	1.039	15.7214	16:0 anteiso	0.40	ECL deviates  0.006	Reference  0.002	
3.2178	5345	0.015	1.039	15.7731	16:1 w9c	1.01	ECL deviates -0.002		
3.2458	37686	0.017	1.038	15.8225	16:1 w7c	7.14	ECL deviates -0.002		
3.2964	11739	0.017	1.037	15.9120	16:1 w5c	2.22	ECL deviates  0.001		
3.3463	55628	0.016	1.036	16.0001	16:0	10.52	ECL deviates  0.000	Reference -0.005	
3.3765	2509	0.017	----	16.0483		----			
3.6149	25329	0.020	1.031	16.4246	16:0 10-methyl	4.77	ECL deviates  0.005		
3.6608	84771	0.017	1.030	16.4971	17:1 iso w9c	15.94	ECL deviates -0.001		
3.7412	7221	0.016	1.028	16.6240	17:0 iso	1.36	ECL deviates  0.000	Reference -0.006	
3.8021	7603	0.017	1.027	16.7202	17:0 anteiso	1.42	ECL deviates  0.000		
3.8518	3359	0.019	1.026	16.7986	17:1 w8c	0.63	ECL deviates  0.002		
3.9137	15467	0.018	1.024	16.8962	17:0 cyclo w7c	2.89	ECL deviates  0.003		
3.9804	2270	0.016	1.023	17.0016	17:0	0.42	ECL deviates  0.002	Reference -0.006	
4.0077	3083	0.017	1.022	17.0418	17:1 w7c 10-methyl	0.58	ECL deviates -0.001		
4.0547	703	0.014	----	17.1105		----			
4.1189	893	0.017	----	17.2043		----			
4.1397	695	0.015	1.019	17.2346	16:0 2OH	0.13	ECL deviates -0.006		
4.2579	3310	0.016	1.016	17.4074	17:0 10-methyl	0.61	ECL deviates  0.000		
4.3192	1639	0.026	----	17.4968		----			
4.3768	1639	0.015	1.013	17.5810	18:3 w6c	0.30	ECL deviates  0.001		
4.4041	2230	0.017	1.012	17.6209	18:0 iso	0.41	ECL deviates -0.006	Reference -0.014	
4.4327	853	0.015	----	17.6626		----			
4.4768	9756	0.018	1.011	17.7270	18:2 w6c	1.80	ECL deviates  0.000		
4.5096	26584	0.018	1.010	17.7749	18:1 w9c	4.90	ECL deviates  0.000		
4.5458	45661	0.019	1.009	17.8277	18:1 w7c	8.41	ECL deviates  0.001		
4.6036	6882	0.020	----	17.9121		----			
4.6649	9359	0.018	1.006	18.0016	18:0	1.72	ECL deviates  0.002	Reference -0.007	
4.7250	4189	0.017	1.004	18.0860	18:1 w7c 10-methyl	0.77	ECL deviates  0.001		
4.7853	1125	0.022	1.003	18.1703	18:2 DMA	0.21	ECL deviates  0.010		
4.9439	12662	0.018	0.999	18.3922	18:0 10-methyl	2.31	ECL deviates -0.003		
5.0609	3818	0.020	0.996	18.5557	19:3 w6c	0.69	ECL deviates -0.004		
5.2012	1740	0.028	----	18.7519		----			
5.2471	1654	0.018	0.992	18.8159	19:1 w8c	0.30	ECL deviates  0.005		
5.2894	2438	0.017	0.991	18.8752	19:0 cyclo w9c	0.44	ECL deviates  0.003		
5.3143	12039	0.017	0.990	18.9099	19:0 cyclo w7c	2.18	ECL deviates  0.000		
5.3828	57170	0.018	----	19.0056	19:0	----	ECL deviates  0.006		
5.5375	754	0.016	----	19.2154		----			
5.5798	921	0.014	----	19.2728		----			
5.6533	1902	0.020	----	19.3725		----			
5.6744	769	0.013	0.982	19.4011	20:4 w6c	0.14	ECL deviates -0.002		
5.8260	1098	0.017	----	19.6064		----			
5.9027	1630	0.016	----	19.7104		----			
5.9481	3490	0.035	0.976	19.7718	20:1 w9c	----	> max ar/ht		
6.1182	2424	0.019	0.973	20.0023	20:0	0.43	ECL deviates  0.002	Reference -0.007	
6.2619	1182	0.021	----	20.1976		----			
6.3757	4100	0.017	----	20.3521		----			
6.4042	29979	0.019	0.968	20.3907	20:0 10-methyl	5.29	ECL deviates -0.006		
6.4399	946	0.015	----	20.4392		----			
6.5717	2780	0.021	----	20.6182		----			
6.6531	2382	0.022	----	20.7289		----			
6.7061	1294	0.017	0.963	20.8007	21:1 w8c	0.23	ECL deviates  0.003		
6.7695	807	0.019	----	20.8868		----			
6.8228	1588	0.015	0.962	20.9593	21:1 w3c	0.28	ECL deviates  0.005		
7.0629	872	0.018	----	21.2851		----			
7.3213	1113	0.024	----	21.6357		----			
7.3684	825	0.018	----	21.6996		----			
7.4598	2300	0.019	----	21.8236		----			
7.5436	617	0.016	0.956	21.9373	22:1 w3c	0.11	ECL deviates -0.010		
7.5920	3379	0.016	0.956	22.0030	22:0	0.59	ECL deviates  0.003	Reference -0.004	
7.7819	102513	0.019	----	22.2645		----			
8.0887	2106	0.019	----	22.6872		----			
8.2591	1242	0.016	0.960	22.9219	23:1 w4c	0.22	ECL deviates -0.005		
8.3147	620	0.015	0.960	22.9984	23:0	0.11	ECL deviates -0.002	Reference -0.006	
8.5279	956	0.018	----	23.2986		----			
8.7967	1980	0.032	----	23.6769		----			
8.8365	1182	0.023	----	23.7329		----			
8.9422	1521	0.016	----	23.8816		----			
9.0253	2989	0.016	0.975	23.9986	24:0	0.53	ECL deviates -0.001	Reference -0.003	
9.3895	7565	0.019	----	24.5110		----	> max rt		

ECL Deviation: 0.004                            Reference ECL Shift: 0.007       Number Reference Peaks: 18
Total Response: 692296                         Total Named: 536051
Percent Named: 77.43%                         Total Amount: 551334
Profile Comment:   Review report comments.

(No search libraries specified in method PLFAD1.)
