# Supplementary material for: Microbial regulation of soil carbon properties under nitrogen addition and plant inputs removal
Source: PeerJ. 2019 Jul 17;7:e7343. doi: 10.7717/peerj.7343 (PMC6642627; doi:10.7717/peerj.7343)
Supplement: File S1 — The raw data showed the soil microbial PLFAs files in the year of 2015 and 2016. Each file of rtf. represented the microbial PLFAs for each soil sample. In the Supplemental File, the Excel file named “Numbers” showed the plots names and the related rtf. file names. [file peerj-07-7343-s002.zip › supplementary files/2016/53.rtf]

Volume: DATA            File: E17C203.64A       Samp Ctr: 6                   ID Number: 5026 
Type: Samp                   Bottle: 17                      Method: PLFAD1 
Created: 12/20/2017 10:54:33 AM 
Sample ID: 53 


RT	Response	Ar/Ht	RFact	ECL	Peak Name	Percent	Comment1	Comment2	
0.7659	1.693E+9	0.015	----	7.6875	SOLVENT PEAK	----	< min rt		
0.9553	1253	0.013	----	8.7682		----	< min rt		
1.8125	714	0.012	1.030	12.7194	13:0 anteiso	0.16	ECL deviates  0.010	Reference  0.017	
1.9914	1114	0.014	----	13.2285		----			
2.1416	2854	0.015	1.043	13.6066	14:0 iso	0.63	ECL deviates -0.007	Reference -0.004	
2.1630	496	0.011	----	13.6605		----			
2.1869	1271	0.014	1.043	13.7205	14:0 anteiso	0.28	ECL deviates  0.005	Reference  0.008	
2.2151	857	0.015	----	13.7916		----			
2.2719	642	0.013	----	13.9346		----			
2.2957	3288	0.015	1.045	13.9944	14:0	0.73	ECL deviates -0.006	Reference -0.003	
2.3591	1291	0.014	----	14.1262	14:0 iso 3OH	----	ECL deviates  0.001		
2.4571	809	0.015	----	14.3291		----			
2.5097	4380	0.018	1.046	14.4380	15:1 iso w6c	0.97	ECL deviates -0.001		
2.5333	792	0.014	1.046	14.4868	15:4 w3c	0.18	ECL deviates -0.003		
2.5536	558	0.012	1.046	14.5289	15:1 anteiso w9c	0.12	ECL deviates -0.001		
2.5943	20520	0.015	1.046	14.6131	15:0 iso	4.55	ECL deviates -0.004	Reference -0.003	
2.6402	13787	0.015	1.046	14.7081	15:0 anteiso	3.06	ECL deviates -0.003	Reference -0.003	
2.7064	1859	0.014	1.045	14.8451	15:1 w7c	0.41	ECL deviates  0.008		
2.7810	2303	0.014	1.045	14.9994	15:0	0.51	ECL deviates -0.001	Reference -0.001	
2.8104	750	0.016	----	15.0516		----			
3.0325	3208	0.021	1.042	15.4439	15:0 DMA	0.71	ECL deviates -0.007		
3.1033	10389	0.016	1.041	15.5689	16:3 w6c	2.29	ECL deviates -0.007		
3.1321	7451	0.016	1.040	15.6199	16:0 iso	1.64	ECL deviates  0.000	Reference -0.002	
3.1564	657	0.012	----	15.6627		----			
3.1894	1803	0.014	1.039	15.7211	16:0 anteiso	0.40	ECL deviates  0.006	Reference  0.004	
3.2169	4049	0.014	1.039	15.7696	16:1 w9c	0.89	ECL deviates -0.005		
3.2455	27997	0.016	1.038	15.8201	16:1 w7c	6.17	ECL deviates -0.004		
3.2976	8440	0.017	1.037	15.9122	16:1 w5c	1.86	ECL deviates  0.001		
3.3471	41471	0.014	1.036	15.9996	16:0	9.12	ECL deviates  0.000	Reference -0.003	
3.3773	1885	0.016	----	16.0476		----			
3.6152	18948	0.019	1.031	16.4230	16:0 10-methyl	4.14	ECL deviates  0.003		
3.6608	97369	0.017	1.030	16.4951	17:1 iso w9c	21.27	ECL deviates -0.003		
3.7414	5154	0.016	1.028	16.6222	17:0 iso	1.12	ECL deviates -0.001	Reference -0.006	
3.8026	6276	0.016	1.027	16.7188	17:0 anteiso	1.37	ECL deviates -0.002		
3.8516	2490	0.015	1.026	16.7962	17:1 w8c	0.54	ECL deviates -0.001		
3.9140	10992	0.017	1.024	16.8946	17:0 cyclo w7c	2.39	ECL deviates  0.001		
3.9795	1708	0.015	1.023	16.9979	17:0	0.37	ECL deviates -0.002	Reference -0.007	
4.0077	2261	0.017	1.022	17.0397	17:1 w7c 10-methyl	0.49	ECL deviates -0.004		
4.1201	830	0.017	----	17.2038		----			
4.1431	546	0.013	1.019	17.2375	16:0 2OH	0.12	ECL deviates -0.003		
4.2582	2340	0.014	1.016	17.4056	17:0 10-methyl	0.50	ECL deviates -0.001		
4.3151	1324	0.023	----	17.4886		----			
4.3766	1814	0.015	1.013	17.5786	18:3 w6c	0.39	ECL deviates -0.001		
4.4034	1707	0.017	1.012	17.6177	18:0 iso	0.37	ECL deviates -0.009	Reference -0.015	
4.4351	809	0.015	----	17.6639		----			
4.4774	9522	0.017	1.011	17.7257	18:2 w6c	2.04	ECL deviates -0.001		
4.5097	20992	0.018	1.010	17.7729	18:1 w9c	4.50	ECL deviates -0.002		
4.5462	32106	0.019	1.009	17.8262	18:1 w7c	6.87	ECL deviates -0.001		
4.6073	4493	0.021	1.007	17.9155	18:1 w5c	0.96	ECL deviates -0.007		
4.6652	7433	0.018	1.006	18.0000	18:0	1.59	ECL deviates  0.000	Reference -0.006	
4.7241	2784	0.017	1.004	18.0827	18:1 w7c 10-methyl	0.59	ECL deviates -0.002		
4.7854	1339	0.031	1.003	18.1683	18:2 DMA	0.28	ECL deviates  0.008		
4.9448	10181	0.020	0.999	18.3913	18:0 10-methyl	2.16	ECL deviates -0.004		
5.0610	3767	0.018	0.996	18.5539	19:3 w6c	0.80	ECL deviates -0.006		
5.1951	1308	0.025	----	18.7414		----		Reference  0.007	
5.2474	1299	0.016	0.992	18.8145	19:1 w8c	0.27	ECL deviates  0.004		
5.2788	1961	0.019	0.991	18.8584	19:1 w6c	0.41	ECL deviates  0.006		
5.3139	9359	0.017	0.990	18.9074	19:0 cyclo w7c	1.97	ECL deviates -0.002		
5.3837	58349	0.018	----	19.0051	19:0	----	ECL deviates  0.005		
5.5369	695	0.017	----	19.2129		----			
5.5784	767	0.015	----	19.2691		----			
5.6749	2670	0.025	0.982	19.3999	20:4 w6c	0.56	ECL deviates -0.003		
5.8231	1006	0.017	----	19.6009		----			
5.9027	1749	0.021	----	19.7088		----			
5.9490	1479	0.019	0.976	19.7716	20:1 w9c	0.31	ECL deviates -0.001		
5.9760	838	0.021	0.976	19.8082	20:1 w8c	0.17	ECL deviates -0.005		
6.1169	2102	0.019	0.973	19.9990	20:0	0.43	ECL deviates -0.001	Reference -0.009	
6.3739	4075	0.016	----	20.3483		----			
6.4051	31237	0.018	0.968	20.3907	20:0 10-methyl	6.41	ECL deviates -0.006		
6.4392	690	0.019	----	20.4371		----			
6.5734	3078	0.020	----	20.6195		----			
6.6530	2683	0.024	----	20.7276		----			
6.7067	1157	0.016	0.963	20.8006	21:1 w8c	0.24	ECL deviates  0.003		
6.7698	910	0.019	----	20.8863		----			
6.8221	1551	0.017	0.962	20.9574	21:1 w3c	0.32	ECL deviates  0.003		
6.8781	2056	0.027	----	21.0335		----			
7.0644	701	0.016	----	21.2865		----			
7.3185	789	0.016	----	21.6315		----			
7.3675	817	0.017	----	21.6980		----			
7.4626	3167	0.021	----	21.8271		----			
7.5915	2884	0.017	0.956	22.0022	22:0	0.59	ECL deviates  0.002	Reference -0.005	
7.7814	110672	0.019	----	22.2641		----			
8.0867	2072	0.018	----	22.6851		----			
8.2602	1422	0.017	0.960	22.9243	23:1 w4c	0.29	ECL deviates -0.002		
8.5263	732	0.015	----	23.2975		----			
8.7938	2026	0.030	----	23.6746		----			
8.8351	1157	0.020	----	23.7327		----			
8.9439	1764	0.017	----	23.8860		----			
9.0226	2393	0.018	0.975	23.9969	24:0	0.50	ECL deviates -0.003	Reference -0.007	
9.3897	12727	0.019	----	24.5141		----	> max rt		

ECL Deviation: 0.004                            Reference ECL Shift: 0.007       Number Reference Peaks: 18
Total Response: 614798                         Total Named: 461711
Percent Named: 75.10%                         Total Amount: 471422

(No search libraries specified in method PLFAD1.)
