# Supplementary material for: Microbial regulation of soil carbon properties under nitrogen addition and plant inputs removal
Source: PeerJ. 2019 Jul 17;7:e7343. doi: 10.7717/peerj.7343 (PMC6642627; doi:10.7717/peerj.7343)
Supplement: File S1 — The raw data showed the soil microbial PLFAs files in the year of 2015 and 2016. Each file of rtf. represented the microbial PLFAs for each soil sample. In the Supplemental File, the Excel file named “Numbers” showed the plots names and the related rtf. file names. [file peerj-07-7343-s002.zip › supplementary files/2016/54.rtf]

Volume: DATA            File: E17C203.64A       Samp Ctr: 7                   ID Number: 5027 
Type: Samp                   Bottle: 18                      Method: PLFAD1 
Created: 12/20/2017 11:22:57 AM 
Sample ID: 54 


RT	Response	Ar/Ht	RFact	ECL	Peak Name	Percent	Comment1	Comment2	
0.7667	1.665E+9	0.015	----	7.6938	SOLVENT PEAK	----	< min rt		
0.9559	1302	0.012	----	8.7726		----	< min rt		
1.3005	475	0.012	----	10.7284		----			
1.6488	610	0.014	----	12.1974		----			
1.7757	617	0.013	1.028	12.6034	13:0 iso	0.11	ECL deviates -0.009	Reference -0.003	
1.8124	1273	0.012	1.030	12.7208	13:0 anteiso	0.23	ECL deviates  0.011	Reference  0.017	
1.9916	1386	0.015	----	13.2307		----			
2.1418	5539	0.016	1.043	13.6087	14:0 iso	0.99	ECL deviates -0.005	Reference -0.003	
2.1873	1486	0.013	1.043	13.7229	14:0 anteiso	0.27	ECL deviates  0.007	Reference  0.009	
2.2164	1123	0.014	1.044	13.7962	14:1 w8c	0.20	ECL deviates -0.005		
2.2704	894	0.014	----	13.9323		----			
2.2962	5069	0.014	1.045	13.9970	14:0	0.91	ECL deviates -0.003	Reference -0.002	
2.3593	1314	0.015	----	14.1279	14:0 iso 3OH	----	ECL deviates  0.003		
2.4554	780	0.015	----	14.3267		----			
2.5095	6851	0.017	1.046	14.4388	15:1 iso w6c	1.23	ECL deviates  0.000		
2.5316	1033	0.011	1.046	14.4844	15:4 w3c	0.19	ECL deviates -0.006		
2.5533	1063	0.013	1.046	14.5294	15:1 anteiso w9c	0.19	ECL deviates -0.001		
2.5941	28156	0.014	1.046	14.6137	15:0 iso	5.07	ECL deviates -0.003	Reference -0.004	
2.6402	19263	0.015	1.046	14.7091	15:0 anteiso	3.47	ECL deviates -0.002	Reference -0.003	
2.7067	1935	0.016	1.045	14.8466	15:1 w7c	0.35	ECL deviates  0.010		
2.7808	2503	0.014	1.045	14.9999	15:0	0.45	ECL deviates  0.000	Reference -0.002	
2.8112	907	0.016	----	15.0540		----			
3.0073	746	0.013	1.042	15.4002	16:1 w7c alcohol	0.13	ECL deviates  0.004		
3.0320	3446	0.017	1.042	15.4440	15:0 DMA	0.62	ECL deviates -0.007		
3.1030	12458	0.016	1.041	15.5692	16:3 w6c	2.23	ECL deviates -0.007		
3.1311	9846	0.015	1.040	15.6189	16:0 iso	1.76	ECL deviates -0.001	Reference -0.004	
3.1563	599	0.011	----	15.6634		----			
3.1888	1835	0.014	1.039	15.7208	16:0 anteiso	0.33	ECL deviates  0.006	Reference  0.003	
3.2168	5718	0.016	1.039	15.7703	16:1 w9c	1.02	ECL deviates -0.005		
3.2464	42292	0.016	1.038	15.8224	16:1 w7c	7.55	ECL deviates -0.002		
3.2983	12462	0.017	1.037	15.9142	16:1 w5c	2.22	ECL deviates  0.003		
3.3472	56580	0.015	1.036	16.0006	16:0	10.09	ECL deviates  0.001	Reference -0.003	
3.3775	2493	0.016	----	16.0486		----			
3.6163	26793	0.018	1.031	16.4255	16:0 10-methyl	4.75	ECL deviates  0.006		
3.6614	82714	0.017	1.030	16.4966	17:1 iso w9c	14.66	ECL deviates -0.001		
3.7421	7150	0.016	1.028	16.6240	17:0 iso	1.26	ECL deviates  0.000	Reference -0.005	
3.8032	7503	0.017	1.027	16.7203	17:0 anteiso	1.33	ECL deviates  0.000		
3.8511	3493	0.017	1.026	16.7960	17:1 w8c	0.62	ECL deviates -0.001		
3.9145	17228	0.018	1.024	16.8960	17:0 cyclo w7c	3.04	ECL deviates  0.002		
3.9814	2237	0.015	1.023	17.0015	17:0	0.39	ECL deviates  0.001	Reference -0.004	
4.0085	2952	0.015	1.022	17.0414	17:1 w7c 10-methyl	0.52	ECL deviates -0.002		
4.0540	665	0.015	----	17.1079		----			
4.1212	678	0.017	----	17.2059		----			
4.2585	2922	0.017	1.016	17.4065	17:0 10-methyl	0.51	ECL deviates  0.000		
4.3191	1492	0.026	----	17.4950		----			
4.3755	1610	0.016	1.013	17.5773	18:3 w6c	0.28	ECL deviates -0.003		
4.4056	2238	0.018	1.012	17.6214	18:0 iso	0.39	ECL deviates -0.005	Reference -0.012	
4.4340	782	0.014	----	17.6628		----			
4.4789	8198	0.018	1.011	17.7284	18:2 w6c	1.43	ECL deviates  0.001		
4.5093	27480	0.019	1.010	17.7727	18:1 w9c	4.77	ECL deviates -0.002		
4.5465	47049	0.019	1.009	17.8270	18:1 w7c	8.17	ECL deviates  0.000		
4.6066	7093	0.020	1.007	17.9149	18:1 w5c	1.23	ECL deviates -0.008		
4.6661	9630	0.017	1.006	18.0018	18:0	1.67	ECL deviates  0.002	Reference -0.005	
4.7247	4271	0.015	1.004	18.0839	18:1 w7c 10-methyl	0.74	ECL deviates -0.001		
4.7830	928	0.022	1.003	18.1654	18:2 DMA	0.16	ECL deviates  0.005		
4.9437	12719	0.019	0.999	18.3901	18:0 10-methyl	2.19	ECL deviates -0.005		
5.0619	3319	0.017	0.996	18.5555	19:3 w6c	0.57	ECL deviates -0.005		
5.1973	1781	0.026	----	18.7447		----			
5.2459	1573	0.017	0.992	18.8127	19:1 w8c	0.27	ECL deviates  0.002		
5.2854	2383	0.017	0.991	18.8680	19:0 cyclo w9c	0.41	ECL deviates -0.004		
5.3148	14193	0.017	0.990	18.9091	19:0 cyclo w7c	2.42	ECL deviates -0.001		
5.3841	56001	0.016	----	19.0059	19:0	----	ECL deviates  0.006		
5.5797	1048	0.014	----	19.2711		----			
5.6517	1588	0.020	----	19.3688		----			
5.6738	998	0.014	0.982	19.3988	20:4 w6c	0.17	ECL deviates -0.005		
5.8255	1067	0.017	----	19.6044		----			
5.9022	1393	0.016	----	19.7084		----			
5.9482	2317	0.023	0.976	19.7707	20:1 w9c	0.39	ECL deviates -0.002		
5.9758	1054	0.021	0.976	19.8081	20:1 w8c	0.18	ECL deviates -0.005		
6.1175	2774	0.019	0.973	20.0003	20:0	0.46	ECL deviates  0.000	Reference -0.008	
6.2606	1131	0.022	----	20.1948		----			
6.3744	4081	0.017	----	20.3493		----			
6.4041	29276	0.019	0.968	20.3898	20:0 10-methyl	4.87	ECL deviates -0.007		
6.4404	1057	0.017	----	20.4391		----			
6.5721	2842	0.021	----	20.6180		----			
6.6548	2354	0.023	----	20.7304		----			
6.7061	1408	0.016	0.963	20.8001	21:1 w8c	0.23	ECL deviates  0.002		
6.7692	1047	0.019	----	20.8858		----			
6.8245	2026	0.017	0.962	20.9610	21:1 w3c	0.34	ECL deviates  0.007		
7.0623	906	0.016	----	21.2840		----			
7.4594	2241	0.020	0.957	21.8233	22:1 w8c	0.37	ECL deviates  0.010		
7.5425	690	0.016	0.956	21.9362	22:1 w3c	0.11	ECL deviates -0.011		
7.5904	3577	0.016	0.956	22.0012	22:0	0.59	ECL deviates  0.001	Reference -0.006	
7.7821	94588	0.018	----	22.2656		----			
8.0869	2276	0.020	----	22.6860		----			
8.2579	1410	0.017	0.960	22.9217	23:1 w4c	0.23	ECL deviates -0.005		
8.3136	977	0.018	0.960	22.9986	23:0	0.16	ECL deviates -0.001	Reference -0.008	
8.5271	1152	0.018	----	23.2995		----			
8.7969	1423	0.027	----	23.6798		----			
8.9417	1478	0.017	----	23.8839		----			
9.0243	3129	0.016	0.975	24.0003	24:0	0.53	ECL deviates  0.000	Reference -0.005	
9.3904	18213	0.019	----	24.5162		----	> max rt		

ECL Deviation: 0.005                            Reference ECL Shift: 0.007       Number Reference Peaks: 19
Total Response: 701820                         Total Named: 568847
Percent Named: 81.05%                         Total Amount: 581214

(No search libraries specified in method PLFAD1.)
