# Supplementary material for: Microbial regulation of soil carbon properties under nitrogen addition and plant inputs removal
Source: PeerJ. 2019 Jul 17;7:e7343. doi: 10.7717/peerj.7343 (PMC6642627; doi:10.7717/peerj.7343)
Supplement: File S1 — The raw data showed the soil microbial PLFAs files in the year of 2015 and 2016. Each file of rtf. represented the microbial PLFAs for each soil sample. In the Supplemental File, the Excel file named “Numbers” showed the plots names and the related rtf. file names. [file peerj-07-7343-s002.zip › supplementary files/2016/55.rtf]

Volume: DATA            File: E17C203.64A       Samp Ctr: 8                   ID Number: 5028 
Type: Samp                   Bottle: 19                      Method: PLFAD1 
Created: 12/20/2017 11:53:24 AM 
Sample ID: 55 


RT	Response	Ar/Ht	RFact	ECL	Peak Name	Percent	Comment1	Comment2	
0.7656	1.675E+9	0.016	----	7.6890	SOLVENT PEAK	----	< min rt		
0.9542	1409	0.012	----	8.7642		----	< min rt		
1.8117	901	0.011	1.030	12.7187	13:0 anteiso	0.17	ECL deviates  0.009	Reference  0.014	
1.9906	1213	0.015	----	13.2283		----			
2.1406	4311	0.016	1.043	13.6057	14:0 iso	0.84	ECL deviates -0.008	Reference -0.006	
2.1862	1213	0.013	1.043	13.7204	14:0 anteiso	0.24	ECL deviates  0.004	Reference  0.006	
2.2163	818	0.015	1.044	13.7961	14:1 w8c	0.16	ECL deviates -0.006		
2.2953	4306	0.017	1.045	13.9949	14:0	0.84	ECL deviates -0.005	Reference -0.004	
2.3573	1162	0.011	----	14.1239	14:0 iso 3OH	----	ECL deviates -0.001		
2.4541	697	0.015	----	14.3241		----			
2.5091	5374	0.018	1.046	14.4380	15:1 iso w6c	1.05	ECL deviates -0.001		
2.5319	909	0.012	1.046	14.4851	15:4 w3c	0.18	ECL deviates -0.005		
2.5519	825	0.012	1.046	14.5266	15:1 anteiso w9c	0.16	ECL deviates -0.003		
2.5937	26190	0.015	1.046	14.6130	15:0 iso	5.10	ECL deviates -0.004	Reference -0.005	
2.6399	17923	0.014	1.046	14.7087	15:0 anteiso	3.49	ECL deviates -0.002	Reference -0.003	
2.7065	1788	0.015	1.045	14.8463	15:1 w7c	0.35	ECL deviates  0.009		
2.7792	2472	0.013	1.045	14.9967	15:0	0.48	ECL deviates -0.003	Reference -0.005	
2.8100	1187	0.015	----	15.0518		----			
3.0080	593	0.012	1.042	15.4015	16:1 w7c alcohol	0.12	ECL deviates  0.005		
3.0333	3511	0.018	1.042	15.4462	15:0 DMA	0.68	ECL deviates -0.004		
3.1031	12153	0.017	1.041	15.5694	16:3 w6c	2.36	ECL deviates -0.006		
3.1314	10137	0.016	1.040	15.6194	16:0 iso	1.97	ECL deviates  0.000	Reference -0.003	
3.1889	2112	0.017	1.039	15.7210	16:0 anteiso	0.41	ECL deviates  0.006	Reference  0.003	
3.2177	5154	0.017	1.039	15.7719	16:1 w9c	1.00	ECL deviates -0.003		
3.2467	36487	0.017	1.038	15.8231	16:1 w7c	7.06	ECL deviates -0.001		
3.2989	11266	0.017	1.037	15.9153	16:1 w5c	2.18	ECL deviates  0.004		
3.3475	47159	0.015	1.036	16.0011	16:0	9.11	ECL deviates  0.001	Reference -0.003	
3.3765	2702	0.017	----	16.0471		----			
3.6164	25040	0.019	1.031	16.4257	16:0 10-methyl	4.81	ECL deviates  0.006		
3.6620	91623	0.017	1.030	16.4977	17:1 iso w9c	17.59	ECL deviates  0.000		
3.7432	6911	0.016	1.028	16.6257	17:0 iso	1.32	ECL deviates  0.002	Reference -0.003	
3.8035	7289	0.018	1.027	16.7209	17:0 anteiso	1.39	ECL deviates  0.001		
3.8529	3053	0.016	1.026	16.7988	17:1 w8c	0.58	ECL deviates  0.002		
3.9157	14894	0.019	1.024	16.8978	17:0 cyclo w7c	2.84	ECL deviates  0.004		
3.9812	1946	0.015	1.023	17.0012	17:0	0.37	ECL deviates  0.001	Reference -0.004	
4.0084	3551	0.014	1.022	17.0412	17:1 w7c 10-methyl	0.68	ECL deviates -0.002		
4.0549	663	0.013	----	17.1091		----			
4.1209	881	0.018	----	17.2055		----			
4.2590	2736	0.017	1.016	17.4072	17:0 10-methyl	0.52	ECL deviates  0.000		
4.3183	1531	0.024	----	17.4938		----			
4.3774	1674	0.016	1.013	17.5801	18:3 w6c	0.32	ECL deviates  0.000		
4.4060	2045	0.018	1.012	17.6219	18:0 iso	0.39	ECL deviates -0.005	Reference -0.011	
4.4365	730	0.013	----	17.6664		----			
4.4790	7709	0.018	1.011	17.7284	18:2 w6c	1.45	ECL deviates  0.001		
4.5114	24881	0.017	1.010	17.7757	18:1 w9c	4.68	ECL deviates  0.001		
4.5476	38424	0.018	1.009	17.8286	18:1 w7c	7.22	ECL deviates  0.002		
4.6089	5684	0.021	1.007	17.9181	18:1 w5c	1.07	ECL deviates -0.005		
4.6673	8687	0.017	1.006	18.0033	18:0	1.63	ECL deviates  0.003	Reference -0.003	
4.7267	3276	0.017	1.004	18.0866	18:1 w7c 10-methyl	0.61	ECL deviates  0.002		
4.7878	1156	0.027	1.003	18.1720	18:2 DMA	0.22	ECL deviates  0.012		
4.9461	13177	0.020	0.999	18.3933	18:0 10-methyl	2.45	ECL deviates -0.002		
5.0633	3682	0.019	0.996	18.5572	19:3 w6c	0.68	ECL deviates -0.003		
5.1996	1756	0.025	----	18.7477		----			
5.2495	1630	0.017	0.992	18.8175	19:1 w8c	0.30	ECL deviates  0.007		
5.2873	1963	0.017	0.991	18.8703	19:0 cyclo w9c	0.36	ECL deviates -0.002		
5.3155	11499	0.018	0.990	18.9098	19:0 cyclo w7c	2.12	ECL deviates  0.000		
5.3845	56784	0.018	----	19.0062	19:0	----	ECL deviates  0.006		
5.5367	935	0.016	----	19.2126		----			
5.5807	628	0.013	----	19.2722		----			
5.6508	1283	0.019	----	19.3673		----			
5.6748	819	0.014	0.982	19.3998	20:4 w6c	0.15	ECL deviates -0.004		
5.8261	1108	0.018	----	19.6049		----			
5.9038	1665	0.017	----	19.7101		----			
5.9485	2222	0.021	0.976	19.7708	20:1 w9c	0.40	ECL deviates -0.002		
5.9770	1297	0.022	0.976	19.8094	20:1 w8c	0.24	ECL deviates -0.004		
6.1189	2218	0.018	0.973	20.0016	20:0	0.40	ECL deviates  0.002	Reference -0.006	
6.2593	644	0.014	----	20.1924		----			
6.3751	3712	0.016	----	20.3498		----			
6.4045	29324	0.018	0.968	20.3898	20:0 10-methyl	5.29	ECL deviates -0.007		
6.4408	909	0.015	----	20.4391		----			
6.4695	627	0.013	----	20.4781		----			
6.5716	2909	0.021	----	20.6169		----			
6.6540	2538	0.024	----	20.7287		----			
6.7089	1422	0.016	0.963	20.8034	21:1 w8c	0.26	ECL deviates  0.005		
6.8253	2070	0.016	0.962	20.9615	21:1 w3c	0.37	ECL deviates  0.007		
6.8758	1045	0.024	----	21.0301		----			
7.3673	588	0.013	----	21.6975		----			
7.4613	2936	0.018	----	21.8250		----			
7.5446	721	0.016	0.956	21.9382	22:1 w3c	0.13	ECL deviates -0.009		
7.5905	2658	0.017	0.956	22.0005	22:0	0.47	ECL deviates  0.001	Reference -0.006	
7.7843	104648	0.018	----	22.2676		----			
8.0895	2305	0.018	----	22.6885		----			
8.2587	1501	0.017	0.960	22.9218	23:1 w4c	0.27	ECL deviates -0.005		
8.5256	724	0.016	----	23.2962		----			
8.7966	2209	0.028	----	23.6780		----			
8.8347	1230	0.020	----	23.7317		----			
8.9418	2112	0.016	----	23.8826		----			
9.0241	2659	0.017	0.975	23.9986	24:0	0.48	ECL deviates -0.001	Reference -0.005	
9.3896	13999	0.017	----	24.5135		----	> max rt		
9.4891	638	0.014	----	24.6537		----	> max rt		

ECL Deviation: 0.005                            Reference ECL Shift: 0.006       Number Reference Peaks: 17
Total Response: 671157                         Total Named: 525043
Percent Named: 78.23%                         Total Amount: 536630

(No search libraries specified in method PLFAD1.)
