# Supplementary material for: Microbial regulation of soil carbon properties under nitrogen addition and plant inputs removal
Source: PeerJ. 2019 Jul 17;7:e7343. doi: 10.7717/peerj.7343 (PMC6642627; doi:10.7717/peerj.7343)
Supplement: File S1 — The raw data showed the soil microbial PLFAs files in the year of 2015 and 2016. Each file of rtf. represented the microbial PLFAs for each soil sample. In the Supplemental File, the Excel file named “Numbers” showed the plots names and the related rtf. file names. [file peerj-07-7343-s002.zip › supplementary files/2016/56.rtf]

Volume: DATA            File: E17C203.64A       Samp Ctr: 9                   ID Number: 5029 
Type: Samp                   Bottle: 20                      Method: PLFAD1 
Created: 12/20/2017 12:23:43 PM 
Sample ID: 56 


RT	Response	Ar/Ht	RFact	ECL	Peak Name	Percent	Comment1	Comment2	
0.7652	1.644E+9	0.016	----	7.6845	SOLVENT PEAK	----	< min rt		
0.9525	559	0.011	----	8.7527		----	< min rt		
1.8120	988	0.013	1.030	12.7193	13:0 anteiso	0.26	ECL deviates  0.010	Reference  0.016	
1.9904	1204	0.015	----	13.2272		----			
2.1409	2478	0.015	1.043	13.6058	14:0 iso	0.67	ECL deviates -0.008	Reference -0.006	
2.1621	465	0.010	----	13.6592		----			
2.1869	1254	0.014	1.043	13.7216	14:0 anteiso	0.34	ECL deviates  0.006	Reference  0.008	
2.2152	915	0.014	1.044	13.7928	14:1 w8c	0.25	ECL deviates -0.009		
2.2695	732	0.016	----	13.9294		----			
2.2950	2444	0.015	1.045	13.9937	14:0	0.66	ECL deviates -0.006	Reference -0.005	
2.3581	1049	0.012	----	14.1250	14:0 iso 3OH	----	ECL deviates  0.000		
2.4574	594	0.014	----	14.3304		----			
2.5093	3088	0.017	1.046	14.4378	15:1 iso w6c	0.84	ECL deviates -0.001		
2.5303	707	0.014	1.046	14.4813	15:4 w3c	0.19	ECL deviates -0.009		
2.5939	14705	0.014	1.046	14.6128	15:0 iso	4.00	ECL deviates -0.004	Reference -0.004	
2.6401	11849	0.016	1.046	14.7085	15:0 anteiso	3.22	ECL deviates -0.003	Reference -0.003	
2.7067	1798	0.013	1.045	14.8463	15:1 w7c	0.49	ECL deviates  0.009		
2.7801	1534	0.015	1.045	14.9982	15:0	0.42	ECL deviates -0.002	Reference -0.003	
2.8115	618	0.014	----	15.0540		----			
3.0344	2041	0.018	1.042	15.4478	15:0 DMA	0.55	ECL deviates -0.003		
3.1029	13456	0.016	1.041	15.5688	16:3 w6c	3.64	ECL deviates -0.007		
3.1311	5624	0.016	1.040	15.6185	16:0 iso	1.52	ECL deviates -0.001	Reference -0.004	
3.1551	642	0.011	----	15.6609		----			
3.1884	1547	0.013	1.039	15.7197	16:0 anteiso	0.42	ECL deviates  0.005	Reference  0.002	
3.2182	3133	0.015	1.039	15.7724	16:1 w9c	0.85	ECL deviates -0.003		
3.2465	21437	0.017	1.038	15.8223	16:1 w7c	5.79	ECL deviates -0.002		
3.2979	6796	0.015	1.037	15.9131	16:1 w5c	1.83	ECL deviates  0.002		
3.3465	29906	0.016	1.036	15.9989	16:0	8.06	ECL deviates -0.001	Reference -0.005	
3.3766	1918	0.016	----	16.0468		----			
3.6160	14646	0.019	1.031	16.4247	16:0 10-methyl	3.93	ECL deviates  0.005		
3.6610	87968	0.016	1.030	16.4957	17:1 iso w9c	23.56	ECL deviates -0.002		
3.7430	3876	0.015	1.028	16.6251	17:0 iso	1.04	ECL deviates  0.001	Reference -0.003	
3.8037	4404	0.017	1.027	16.7208	17:0 anteiso	1.18	ECL deviates  0.001		
3.8526	1884	0.016	1.026	16.7979	17:1 w8c	0.50	ECL deviates  0.001		
3.9143	9070	0.019	1.024	16.8954	17:0 cyclo w7c	2.42	ECL deviates  0.002		
3.9823	1248	0.015	1.023	17.0026	17:0	0.33	ECL deviates  0.003	Reference -0.003	
4.0090	2126	0.014	1.022	17.0418	17:1 w7c 10-methyl	0.56	ECL deviates -0.001		
4.1210	1259	0.022	----	17.2054		----			
4.2600	1608	0.016	1.016	17.4084	17:0 10-methyl	0.42	ECL deviates  0.001		
4.3183	1033	0.024	----	17.4936		----			
4.3775	1535	0.015	1.013	17.5801	18:3 w6c	0.40	ECL deviates  0.000		
4.4075	1478	0.018	1.012	17.6240	18:0 iso	0.39	ECL deviates -0.003	Reference -0.009	
4.4338	685	0.014	----	17.6623		----			
4.4782	4694	0.015	1.011	17.7272	18:2 w6c	1.23	ECL deviates  0.000		
4.5104	15318	0.018	1.010	17.7742	18:1 w9c	4.02	ECL deviates  0.000		
4.5483	24872	0.018	1.009	17.8295	18:1 w7c	6.53	ECL deviates  0.003		
4.6095	2892	0.021	1.007	17.9188	18:1 w5c	0.76	ECL deviates -0.004		
4.6673	6171	0.016	1.006	18.0032	18:0	1.61	ECL deviates  0.003	Reference -0.003	
4.7269	1986	0.017	1.004	18.0869	18:1 w7c 10-methyl	0.52	ECL deviates  0.002		
4.9451	7397	0.019	0.999	18.3920	18:0 10-methyl	1.92	ECL deviates -0.003		
5.0622	3511	0.019	0.996	18.5558	19:3 w6c	0.91	ECL deviates -0.004		
5.1990	1291	0.026	----	18.7471		----			
5.2481	1147	0.017	0.992	18.8157	19:1 w8c	0.30	ECL deviates  0.005		
5.2789	1785	0.019	0.991	18.8588	19:1 w6c	0.46	ECL deviates  0.007		
5.3143	7320	0.018	0.990	18.9083	19:0 cyclo w7c	1.88	ECL deviates -0.001		
5.3838	60253	0.018	----	19.0055	19:0	----	ECL deviates  0.005		
5.5804	647	0.013	----	19.2722		----			
5.8244	759	0.015	----	19.6029		----			
5.9026	1970	0.019	----	19.7091		----			
5.9485	1014	0.017	0.976	19.7712	20:1 w9c	0.26	ECL deviates -0.001		
6.1188	2062	0.018	0.973	20.0022	20:0	0.52	ECL deviates  0.002	Reference -0.006	
6.3753	4293	0.015	----	20.3508		----			
6.4048	31653	0.018	0.968	20.3909	20:0 10-methyl	7.97	ECL deviates -0.006		
6.4415	884	0.015	----	20.4408		----			
6.4663	544	0.013	----	20.4745		----			
6.5716	2854	0.019	----	20.6176		----			
6.6539	2428	0.025	----	20.7295		----			
6.7057	1030	0.016	0.963	20.7998	21:1 w8c	0.26	ECL deviates  0.002		
6.8251	1772	0.018	0.962	20.9621	21:1 w3c	0.44	ECL deviates  0.008		
7.3684	564	0.013	----	21.7001		----			
7.4597	2217	0.020	----	21.8242		----			
7.5891	2551	0.017	0.956	21.9998	22:0	0.63	ECL deviates  0.000	Reference -0.008	
7.7823	107454	0.020	----	22.2664		----			
8.0884	2151	0.018	----	22.6886		----			
8.2573	1452	0.016	0.960	22.9216	23:1 w4c	0.36	ECL deviates -0.005		
8.5261	824	0.016	----	23.2987		----			
8.7937	1253	0.027	----	23.6761		----			
8.9424	1736	0.018	----	23.8858		----			
9.0225	2637	0.020	0.975	23.9987	24:0	0.67	ECL deviates -0.001	Reference -0.007	
9.3907	6153	0.019	----	24.5177		----	> max rt		

ECL Deviation: 0.004                            Reference ECL Shift: 0.006       Number Reference Peaks: 17
Total Response: 517823                         Total Named: 376805
Percent Named: 72.77%                         Total Amount: 384545

(No search libraries specified in method PLFAD1.)
