# Supplementary material for: Microbial regulation of soil carbon properties under nitrogen addition and plant inputs removal
Source: PeerJ. 2019 Jul 17;7:e7343. doi: 10.7717/peerj.7343 (PMC6642627; doi:10.7717/peerj.7343)
Supplement: File S1 — The raw data showed the soil microbial PLFAs files in the year of 2015 and 2016. Each file of rtf. represented the microbial PLFAs for each soil sample. In the Supplemental File, the Excel file named “Numbers” showed the plots names and the related rtf. file names. [file peerj-07-7343-s002.zip › supplementary files/2016/57.rtf]

Volume: DATA            File: E17C203.64A       Samp Ctr: 10                 ID Number: 5030 
Type: Samp                   Bottle: 21                      Method: PLFAD1 
Created: 12/20/2017 12:54:16 PM 
Sample ID: 57 


RT	Response	Ar/Ht	RFact	ECL	Peak Name	Percent	Comment1	Comment2	
0.7652	1.68E+9	0.015	----	7.6897	SOLVENT PEAK	----	< min rt		
0.9518	498	0.011	----	8.7539		----	< min rt		
1.8108	1036	0.012	1.030	12.7183	13:0 anteiso	0.31	ECL deviates  0.009	Reference  0.012	
1.9887	1038	0.015	----	13.2256		----			
2.1393	2328	0.017	1.043	13.6045	14:0 iso	0.71	ECL deviates -0.010	Reference -0.010	
2.1850	1269	0.013	1.043	13.7194	14:0 anteiso	0.39	ECL deviates  0.003	Reference  0.003	
2.2148	829	0.015	1.044	13.7945	14:1 w8c	0.25	ECL deviates -0.007		
2.2688	576	0.013	----	13.9304		----			
2.2941	2378	0.015	1.045	13.9941	14:0	0.73	ECL deviates -0.006	Reference -0.007	
2.3573	981	0.014	----	14.1260	14:0 iso 3OH	----	ECL deviates  0.001		
2.4560	643	0.014	----	14.3303		----			
2.5083	2705	0.015	1.046	14.4385	15:1 iso w6c	0.83	ECL deviates  0.000		
2.5928	12674	0.015	1.046	14.6132	15:0 iso	3.90	ECL deviates -0.004	Reference -0.007	
2.6393	10370	0.016	1.046	14.7094	15:0 anteiso	3.19	ECL deviates -0.002	Reference -0.005	
2.7045	2033	0.014	1.045	14.8444	15:1 w7c	0.63	ECL deviates  0.007		
2.7795	1268	0.014	1.045	14.9994	15:0	0.39	ECL deviates -0.001	Reference -0.004	
3.0322	2225	0.020	1.042	15.4463	15:0 DMA	0.68	ECL deviates -0.004		
3.1018	14038	0.015	1.041	15.5691	16:3 w6c	4.30	ECL deviates -0.007		
3.1307	5131	0.015	1.040	15.6203	16:0 iso	1.57	ECL deviates  0.001	Reference -0.004	
3.1545	582	0.011	----	15.6623		----			
3.1888	1388	0.015	1.039	15.7229	16:0 anteiso	0.42	ECL deviates  0.008	Reference  0.003	
3.2159	2669	0.015	1.039	15.7707	16:1 w9c	0.82	ECL deviates -0.004		
3.2453	18589	0.017	1.038	15.8226	16:1 w7c	5.68	ECL deviates -0.002		
3.2968	5716	0.016	1.037	15.9135	16:1 w5c	1.74	ECL deviates  0.002		
3.3460	28659	0.016	1.036	16.0003	16:0	8.74	ECL deviates  0.000	Reference -0.005	
3.3758	1405	0.015	----	16.0479		----			
3.6145	12144	0.019	1.031	16.4246	16:0 10-methyl	3.68	ECL deviates  0.005		
3.6595	67359	0.016	1.030	16.4956	17:1 iso w9c	20.41	ECL deviates -0.002		
3.7417	3525	0.015	1.028	16.6252	17:0 iso	1.07	ECL deviates  0.001	Reference -0.005	
3.8028	4049	0.015	1.027	16.7215	17:0 anteiso	1.22	ECL deviates  0.001		
3.8511	1736	0.017	1.026	16.7978	17:1 w8c	0.52	ECL deviates  0.001		
3.9134	7979	0.018	1.024	16.8961	17:0 cyclo w7c	2.40	ECL deviates  0.002		
3.9797	1041	0.014	1.023	17.0006	17:0	0.31	ECL deviates  0.001	Reference -0.007	
4.0082	1617	0.014	1.022	17.0426	17:1 w7c 10-methyl	0.49	ECL deviates -0.001		
4.1184	658	0.013	----	17.2037		----			
4.2579	1609	0.016	1.016	17.4073	17:0 10-methyl	0.48	ECL deviates  0.000		
4.3153	1019	0.021	----	17.4911		----			
4.3773	1513	0.016	1.013	17.5816	18:3 w6c	0.45	ECL deviates  0.002		
4.4060	1287	0.017	1.012	17.6235	18:0 iso	0.38	ECL deviates -0.003	Reference -0.011	
4.4348	644	0.015	----	17.6655		----			
4.4766	5264	0.018	1.011	17.7266	18:2 w6c	1.56	ECL deviates  0.000		
4.5094	14956	0.018	1.010	17.7744	18:1 w9c	4.44	ECL deviates  0.000		
4.5453	23666	0.017	1.009	17.8269	18:1 w7c	7.02	ECL deviates  0.000		
4.6069	3281	0.022	1.007	17.9168	18:1 w5c	0.97	ECL deviates -0.006		
4.6653	5875	0.015	1.006	18.0021	18:0	1.74	ECL deviates  0.002	Reference -0.006	
4.7242	1982	0.015	1.004	18.0847	18:1 w7c 10-methyl	0.59	ECL deviates  0.000		
4.9444	7077	0.020	0.999	18.3925	18:0 10-methyl	2.08	ECL deviates -0.002		
5.0617	2841	0.018	0.996	18.5565	19:3 w6c	0.83	ECL deviates -0.003		
5.2455	924	0.017	0.992	18.8134	19:1 w8c	0.27	ECL deviates  0.003		
5.2774	1406	0.018	0.991	18.8580	19:1 w6c	0.41	ECL deviates  0.006		
5.3127	6405	0.016	0.990	18.9074	19:0 cyclo w7c	1.87	ECL deviates -0.002		
5.3829	62200	0.018	----	19.0055	19:0	----	ECL deviates  0.005		
5.9001	1890	0.018	----	19.7065		----			
5.9465	858	0.018	0.976	19.7694	20:1 w9c	0.25	ECL deviates -0.003		
6.1160	1528	0.017	0.973	19.9991	20:0	0.44	ECL deviates -0.001	Reference -0.010	
6.3714	3350	0.015	----	20.3460		----			
6.4029	29161	0.019	0.968	20.3889	20:0 10-methyl	8.30	ECL deviates -0.008		
6.4394	513	0.013	----	20.4385		----			
6.5715	2299	0.018	----	20.6178		----			
6.6503	2204	0.024	----	20.7249		----			
6.7057	893	0.016	0.963	20.8002	21:1 w8c	0.25	ECL deviates  0.002		
6.8214	1194	0.015	0.962	20.9573	21:1 w3c	0.34	ECL deviates  0.003		
7.3657	701	0.016	----	21.6962		----			
7.4572	1959	0.018	0.957	21.8204	22:1 w8c	0.55	ECL deviates  0.007		
7.5900	2139	0.017	0.956	22.0007	22:0	0.60	ECL deviates  0.001	Reference -0.007	
7.7809	86338	0.019	----	22.2639		----			
8.0874	2059	0.017	----	22.6863		----			
8.2598	923	0.016	0.960	22.9240	23:1 w4c	0.26	ECL deviates -0.002		
8.5265	649	0.015	----	23.2980		----			
8.7980	1441	0.027	----	23.6805		----			
8.9412	1728	0.017	----	23.8822		----			
9.0244	1778	0.017	0.975	23.9994	24:0	0.51	ECL deviates -0.001	Reference -0.005	
9.3876	9903	0.019	----	24.5110		----	> max rt		

ECL Deviation: 0.004                            Reference ECL Shift: 0.007       Number Reference Peaks: 17
Total Response: 443013                         Total Named: 333275
Percent Named: 75.23%                         Total Amount: 339932

(No search libraries specified in method PLFAD1.)
