# Supplementary material for: Microbial regulation of soil carbon properties under nitrogen addition and plant inputs removal
Source: PeerJ. 2019 Jul 17;7:e7343. doi: 10.7717/peerj.7343 (PMC6642627; doi:10.7717/peerj.7343)
Supplement: File S1 — The raw data showed the soil microbial PLFAs files in the year of 2015 and 2016. Each file of rtf. represented the microbial PLFAs for each soil sample. In the Supplemental File, the Excel file named “Numbers” showed the plots names and the related rtf. file names. [file peerj-07-7343-s002.zip › supplementary files/2016/58.rtf]

Volume: DATA            File: E17C203.64A       Samp Ctr: 11                 ID Number: 5031 
Type: Samp                   Bottle: 22                      Method: PLFAD1 
Created: 12/20/2017 1:24:40 PM 
Sample ID: 58 


RT	Response	Ar/Ht	RFact	ECL	Peak Name	Percent	Comment1	Comment2	
0.7658	1.673E+9	0.015	----	7.6889	SOLVENT PEAK	----	< min rt		
0.9535	634	0.012	----	8.7599		----	< min rt		
1.5850	662	0.015	1.013	11.9963	12:0	0.08	ECL deviates -0.004	Reference  0.001	
1.7753	739	0.014	1.028	12.6050	13:0 iso	0.09	ECL deviates -0.007	Reference -0.005	
1.8114	1276	0.015	1.030	12.7206	13:0 anteiso	0.15	ECL deviates  0.011	Reference  0.014	
1.9907	1247	0.016	----	13.2308		----			
2.1413	7962	0.015	1.043	13.6097	14:0 iso	0.97	ECL deviates -0.004	Reference -0.004	
2.1865	1511	0.015	1.043	13.7234	14:0 anteiso	0.19	ECL deviates  0.007	Reference  0.007	
2.2161	1303	0.015	1.044	13.7976	14:1 w8c	0.16	ECL deviates -0.004		
2.2954	8407	0.016	1.045	13.9970	14:0	1.03	ECL deviates -0.003	Reference -0.004	
2.3587	1325	0.013	----	14.1288	14:0 iso 3OH	----	ECL deviates  0.004		
2.4567	714	0.016	----	14.3315		----			
2.5092	11681	0.018	1.046	14.4401	15:1 iso w6c	1.43	ECL deviates  0.001		
2.5304	1334	0.010	1.046	14.4839	15:4 w3c	0.16	ECL deviates -0.006		
2.5527	1938	0.013	1.046	14.5301	15:1 anteiso w9c	0.24	ECL deviates  0.000		
2.5941	45605	0.015	1.046	14.6157	15:0 iso	5.60	ECL deviates -0.001	Reference -0.004	
2.6399	31392	0.016	1.046	14.7104	15:0 anteiso	3.85	ECL deviates -0.001	Reference -0.003	
2.7072	2253	0.015	----	14.8495		----			
2.7803	4048	0.015	1.045	15.0007	15:0	0.50	ECL deviates  0.001	Reference -0.003	
2.8107	1192	0.015	----	15.0547		----			
2.9146	882	0.015	----	15.2381		----			
3.0320	7058	0.023	1.042	15.4455	15:0 DMA	0.86	ECL deviates -0.005		
3.1025	13741	0.015	1.041	15.5699	16:3 w6c	1.68	ECL deviates -0.006		
3.1312	17794	0.015	1.040	15.6206	16:0 iso	2.17	ECL deviates  0.001	Reference -0.004	
3.1865	2692	0.016	1.039	15.7182	16:0 anteiso	0.33	ECL deviates  0.003	Reference -0.001	
3.2165	9315	0.017	1.039	15.7713	16:1 w9c	1.14	ECL deviates -0.004		
3.2461	62082	0.018	1.038	15.8235	16:1 w7c	7.57	ECL deviates -0.001		
3.2977	20360	0.017	1.037	15.9146	16:1 w5c	2.48	ECL deviates  0.003		
3.3470	91498	0.015	1.036	16.0016	16:0	11.13	ECL deviates  0.002	Reference -0.004	
3.3773	3086	0.017	----	16.0497		----			
3.4377	677	0.015	1.035	16.1451	16:2 DMA	0.08	ECL deviates  0.007		
3.4736	1125	0.021	----	16.2018		----			
3.6152	40437	0.017	1.031	16.4252	16:0 10-methyl	4.90	ECL deviates  0.005		
3.6607	79988	0.017	1.030	16.4969	17:1 iso w9c	9.67	ECL deviates -0.001		
3.7417	11134	0.015	1.028	16.6247	17:0 iso	1.34	ECL deviates  0.001	Reference -0.005	
3.8031	13083	0.017	1.027	16.7215	17:0 anteiso	1.58	ECL deviates  0.001		
3.8501	5104	0.018	1.026	16.7957	17:1 w8c	0.61	ECL deviates -0.001		
3.9138	29684	0.019	1.024	16.8962	17:0 cyclo w7c	3.57	ECL deviates  0.003		
3.9803	3807	0.017	1.023	17.0011	17:0	0.46	ECL deviates  0.001	Reference -0.006	
4.0069	4043	0.016	1.022	17.0404	17:1 w7c 10-methyl	0.49	ECL deviates -0.003		
4.0522	945	0.015	----	17.1065		----			
4.1178	750	0.014	----	17.2024		----			
4.1418	961	0.015	1.019	17.2374	16:0 2OH	0.11	ECL deviates -0.003		
4.2568	5039	0.017	1.016	17.4054	17:0 10-methyl	0.60	ECL deviates -0.002		
4.3184	2143	0.020	----	17.4954		----			
4.3775	1976	0.014	1.013	17.5816	18:3 w6c	0.24	ECL deviates  0.002		
4.4020	4229	0.019	1.012	17.6174	18:0 iso	0.50	ECL deviates -0.009	Reference -0.017	
4.4291	991	0.014	----	17.6570		----			
4.4758	12701	0.017	1.011	17.7251	18:2 w6c	1.51	ECL deviates -0.002		
4.5086	43285	0.017	1.010	17.7731	18:1 w9c	5.13	ECL deviates -0.001		
4.5455	80917	0.017	1.009	17.8270	18:1 w7c	9.59	ECL deviates  0.000		
4.6045	11220	0.025	1.007	17.9131	18:1 w5c	1.33	ECL deviates -0.010		
4.6649	14821	0.018	1.006	18.0014	18:0	1.75	ECL deviates  0.001	Reference -0.007	
4.7229	7458	0.017	1.004	18.0827	18:1 w7c 10-methyl	0.88	ECL deviates -0.002		
4.7805	1730	0.024	1.003	18.1633	18:2 DMA	0.20	ECL deviates  0.003		
4.9433	20944	0.021	0.999	18.3909	18:0 10-methyl	2.46	ECL deviates -0.004		
5.0605	4587	0.019	0.996	18.5548	19:3 w6c	0.54	ECL deviates -0.005		
5.1401	679	0.015	0.994	18.6662	19:3 w3c	0.08	ECL deviates  0.008		
5.1978	2175	0.026	----	18.7470		----			
5.2440	2165	0.018	0.992	18.8116	19:1 w8c	0.25	ECL deviates  0.001		
5.2851	3436	0.016	0.991	18.8690	19:0 cyclo w9c	0.40	ECL deviates -0.003		
5.3135	22120	0.018	0.990	18.9088	19:0 cyclo w7c	2.57	ECL deviates -0.001		
5.3826	61401	0.018	----	19.0054	19:0	----	ECL deviates  0.005		
5.4470	592	0.013	0.987	19.0928	19:1 w7c 10-methyl	0.07	ECL deviates -0.010		
5.5343	2107	0.017	----	19.2113		----			
5.5775	1725	0.015	----	19.2698		----			
5.6138	1096	0.015	0.983	19.3190	19:0 cyclo 9,10 DMA	0.13	ECL deviates -0.005		
5.6507	2739	0.019	----	19.3690		----			
5.6728	1986	0.017	0.982	19.3991	20:4 w6c	0.23	ECL deviates -0.004		
5.7300	585	0.014	0.981	19.4766	20:5 w3c	0.07	ECL deviates -0.005		
5.7927	967	0.017	0.979	19.5616	20:3 w6c	0.11	ECL deviates -0.005		
5.8251	1545	0.019	----	19.6056		----			
5.8990	1268	0.016	----	19.7057		----			
5.9473	3261	0.022	0.976	19.7713	20:1 w9c	0.37	ECL deviates -0.001		
5.9690	563	0.013	0.976	19.8006	20:1 w8c	0.06	ECL deviates -0.012		
6.1155	3964	0.020	0.973	19.9993	20:0	0.45	ECL deviates -0.001	Reference -0.010	
6.2586	1748	0.018	----	20.1938		----			
6.3720	4170	0.016	----	20.3480		----			
6.4015	29447	0.017	0.968	20.3881	20:0 10-methyl	3.35	ECL deviates -0.009		
6.5070	1001	0.023	----	20.5316		----			
6.5693	3611	0.021	----	20.6162		----			
6.6509	2860	0.022	----	20.7272		----			
6.7029	1956	0.015	0.963	20.7979	21:1 w8c	0.22	ECL deviates  0.000		
6.7652	1905	0.019	----	20.8825		----			
6.8217	1910	0.017	0.962	20.9594	21:1 w3c	0.22	ECL deviates  0.005		
6.8751	3447	0.031	----	21.0319		----			
7.0595	1386	0.018	----	21.2826		----			
7.3122	2654	0.026	0.957	21.6259	22:0 iso	0.30	ECL deviates  0.008		
7.3396	3653	0.032	----	21.6631		----			
7.4595	8083	0.029	----	21.8261		----			
7.5430	1047	0.019	0.956	21.9396	22:1 w3c	0.12	ECL deviates -0.007		
7.5884	5038	0.019	0.956	22.0012	22:0	0.57	ECL deviates  0.001	Reference -0.009	
7.7800	91292	0.018	----	22.2655		----			
8.0839	2073	0.018	----	22.6849		----			
8.1573	1225	0.020	----	22.7862		----			
8.2143	1397	0.027	----	22.8648		----			
8.2572	2095	0.019	0.960	22.9241	23:1 w4c	0.24	ECL deviates -0.002		
8.3132	1463	0.017	0.960	23.0014	23:0	0.16	ECL deviates  0.001	Reference -0.008	
8.3530	996	0.022	----	23.0574		----			
8.5244	1756	0.020	----	23.2992		----			
8.7810	1896	0.016	0.969	23.6612	24:3 w3c	0.22	ECL deviates  0.007		
8.7916	1308	0.012	----	23.6761		----			
8.8371	4199	0.022	----	23.7404		----			
8.9412	2786	0.016	----	23.8873		----			
9.0205	4070	0.017	0.975	23.9991	24:0	0.47	ECL deviates -0.001	Reference -0.010	
9.3875	9428	0.020	----	24.5168		----	> max rt		

ECL Deviation: 0.005                            Reference ECL Shift: 0.007       Number Reference Peaks: 20
Total Response: 998997                         Total Named: 833211
Percent Named: 83.40%                         Total Amount: 851670

(No search libraries specified in method PLFAD1.)
