# Supplementary material for: Microbial regulation of soil carbon properties under nitrogen addition and plant inputs removal
Source: PeerJ. 2019 Jul 17;7:e7343. doi: 10.7717/peerj.7343 (PMC6642627; doi:10.7717/peerj.7343)
Supplement: File S1 — The raw data showed the soil microbial PLFAs files in the year of 2015 and 2016. Each file of rtf. represented the microbial PLFAs for each soil sample. In the Supplemental File, the Excel file named “Numbers” showed the plots names and the related rtf. file names. [file peerj-07-7343-s002.zip › supplementary files/2016/59.rtf]

Volume: DATA            File: E17C203.64A       Samp Ctr: 12                 ID Number: 5032 
Type: Samp                   Bottle: 23                      Method: PLFAD1 
Created: 12/20/2017 1:55:00 PM 
Sample ID: 59 


RT	Response	Ar/Ht	RFact	ECL	Peak Name	Percent	Comment1	Comment2	
0.7650	1.669E+9	0.016	----	7.6896	SOLVENT PEAK	----	< min rt		
0.9513	494	0.010	----	8.7514		----	< min rt		
1.5852	822	0.017	1.013	12.0021	12:0	0.11	ECL deviates  0.002	Reference  0.002	
1.6481	481	0.010	----	12.2030		----			
1.7729	867	0.013	1.028	12.6015	13:0 iso	0.12	ECL deviates -0.011	Reference -0.012	
1.8108	1426	0.014	1.030	12.7224	13:0 anteiso	0.19	ECL deviates  0.013	Reference  0.012	
1.9892	1582	0.015	----	13.2300		----			
2.1400	7309	0.016	1.043	13.6087	14:0 iso	1.00	ECL deviates -0.005	Reference -0.008	
2.1856	1655	0.015	1.043	13.7232	14:0 anteiso	0.23	ECL deviates  0.007	Reference  0.005	
2.2153	1320	0.016	1.044	13.7978	14:1 w8c	0.18	ECL deviates -0.004		
2.2684	822	0.015	----	13.9313		----			
2.2946	6912	0.014	1.045	13.9969	14:0	0.94	ECL deviates -0.003	Reference -0.006	
2.3569	1668	0.013	----	14.1266	14:0 iso 3OH	----	ECL deviates  0.002		
2.4565	752	0.016	----	14.3324		----			
2.5074	10863	0.021	1.046	14.4376	15:1 iso w6c	1.48	ECL deviates -0.001		
2.5523	1358	0.013	1.046	14.5303	15:1 anteiso w9c	0.19	ECL deviates  0.000		
2.5925	45603	0.014	1.046	14.6133	15:0 iso	6.23	ECL deviates -0.004	Reference -0.007	
2.6388	25868	0.015	1.046	14.7089	15:0 anteiso	3.53	ECL deviates -0.002	Reference -0.006	
2.7055	2108	0.016	1.045	14.8467	15:1 w7c	0.29	ECL deviates  0.010		
2.7794	3808	0.014	1.045	14.9994	15:0	0.52	ECL deviates -0.001	Reference -0.004	
2.8105	1312	0.016	----	15.0549		----			
2.9124	655	0.016	----	15.2345		----			
3.0319	5857	0.022	1.042	15.4454	15:0 DMA	0.80	ECL deviates -0.005		
3.1018	13957	0.016	1.041	15.5686	16:3 w6c	1.90	ECL deviates -0.007		
3.1301	14876	0.016	1.040	15.6185	16:0 iso	2.02	ECL deviates -0.001	Reference -0.006	
3.1877	2442	0.015	1.039	15.7201	16:0 anteiso	0.33	ECL deviates  0.005	Reference  0.001	
3.2168	8454	0.018	1.039	15.7714	16:1 w9c	1.15	ECL deviates -0.004		
3.2456	54021	0.017	1.038	15.8222	16:1 w7c	7.32	ECL deviates -0.002		
3.2972	15362	0.017	1.037	15.9133	16:1 w5c	2.08	ECL deviates  0.002		
3.3464	74783	0.015	1.036	16.0001	16:0	10.12	ECL deviates  0.000	Reference -0.005	
3.3776	3273	0.019	----	16.0497		----			
3.6154	34988	0.019	1.031	16.4246	16:0 10-methyl	4.71	ECL deviates  0.005		
3.6600	105006	0.017	1.030	16.4949	17:1 iso w9c	14.12	ECL deviates -0.003		
3.7415	9777	0.015	1.028	16.6233	17:0 iso	1.31	ECL deviates  0.000	Reference -0.006	
3.8015	10588	0.016	1.027	16.7179	17:0 anteiso	1.42	ECL deviates -0.002		
3.8503	4039	0.016	1.026	16.7949	17:1 w8c	0.54	ECL deviates -0.002		
3.9136	22518	0.018	1.024	16.8946	17:0 cyclo w7c	3.01	ECL deviates  0.001		
3.9800	3016	0.017	1.023	16.9992	17:0	0.40	ECL deviates -0.001	Reference -0.006	
4.0074	3982	0.017	1.022	17.0397	17:1 w7c 10-methyl	0.53	ECL deviates -0.004		
4.0535	895	0.013	----	17.1069		----			
4.1190	642	0.012	----	17.2026		----			
4.1423	792	0.013	1.019	17.2366	16:0 2OH	0.11	ECL deviates -0.004		
4.2571	4223	0.016	1.016	17.4041	17:0 10-methyl	0.56	ECL deviates -0.003		
4.3182	2235	0.025	----	17.4933		----			
4.3767	2171	0.018	1.013	17.5787	18:3 w6c	0.29	ECL deviates -0.001		
4.4033	2983	0.018	1.012	17.6175	18:0 iso	0.39	ECL deviates -0.009	Reference -0.015	
4.4336	1079	0.016	----	17.6618		----			
4.4776	11552	0.018	1.011	17.7259	18:2 w6c	1.52	ECL deviates -0.001		
4.5089	34072	0.017	1.010	17.7716	18:1 w9c	4.49	ECL deviates -0.003		
4.5453	60022	0.017	1.009	17.8248	18:1 w7c	7.91	ECL deviates -0.002		
4.6051	9023	0.022	----	17.9119		----			
4.6641	12630	0.018	1.006	17.9980	18:0	1.66	ECL deviates -0.002	Reference -0.008	
4.7239	5633	0.017	1.004	18.0820	18:1 w7c 10-methyl	0.74	ECL deviates -0.003		
4.7794	1539	0.022	1.003	18.1596	18:2 DMA	0.20	ECL deviates  0.000		
4.8299	1059	0.025	1.002	18.2302	18:1 w9c DMA	0.14	ECL deviates -0.007		
4.9446	17637	0.019	0.999	18.3906	18:0 10-methyl	2.30	ECL deviates -0.004		
5.0607	4463	0.017	0.996	18.5529	19:3 w6c	0.58	ECL deviates -0.007		
5.1960	2403	0.026	----	18.7420		----		Reference  0.008	
5.2457	2497	0.016	0.992	18.8115	19:1 w8c	0.32	ECL deviates  0.001		
5.2787	3568	0.018	0.991	18.8577	19:1 w6c	0.46	ECL deviates  0.006		
5.3127	16871	0.017	0.990	18.9052	19:0 cyclo w7c	2.18	ECL deviates -0.005		
5.3831	62129	0.017	----	19.0035	19:0	----	ECL deviates  0.004		
5.4507	847	0.019	0.987	19.0953	19:1 w7c 10-methyl	0.11	ECL deviates -0.008		
5.5360	1722	0.017	----	19.2109		----			
5.5794	1958	0.017	----	19.2698		----			
5.6116	870	0.016	----	19.3134		----			
5.6503	2298	0.017	----	19.3659		----			
5.6738	1925	0.019	0.982	19.3977	20:4 w6c	0.25	ECL deviates -0.006		
5.7304	532	0.013	0.981	19.4746	20:5 w3c	0.07	ECL deviates -0.008		
5.7605	769	0.017	----	19.5154		----			
5.7966	1014	0.016	0.979	19.5643	20:3 w6c	0.13	ECL deviates -0.002		
5.8256	1890	0.019	----	19.6036		----			
5.9001	1662	0.017	----	19.7045		----			
5.9469	3395	0.025	0.976	19.7679	20:1 w9c	0.43	ECL deviates -0.005		
5.9737	1810	0.021	0.976	19.8043	20:1 w8c	0.23	ECL deviates -0.009		
6.1170	3649	0.018	0.973	19.9986	20:0	0.46	ECL deviates -0.001	Reference -0.008	
6.2577	1274	0.017	----	20.1898		----			
6.3743	5277	0.017	----	20.3484		----			
6.4038	33593	0.019	0.968	20.3885	20:0 10-methyl	4.24	ECL deviates -0.009		
6.4397	1624	0.016	----	20.4373		----			
6.4673	900	0.015	----	20.4748		----			
6.5126	1342	0.028	----	20.5364		----			
6.5708	4323	0.023	----	20.6155		----			
6.6510	3597	0.026	----	20.7246		----			
6.7059	2526	0.017	0.963	20.7992	21:1 w8c	0.32	ECL deviates  0.001		
6.7674	2993	0.020	----	20.8827		----			
6.8230	3494	0.019	0.962	20.9583	21:1 w3c	0.44	ECL deviates  0.004		
6.8754	2900	0.036	----	21.0296		----	> max ar/ht		
6.9406	736	0.016	----	21.1182		----			
6.9668	926	0.022	----	21.1538		----			
7.0631	1828	0.019	----	21.2847		----			
7.3160	2387	0.026	0.957	21.6285	22:0 iso	0.30	ECL deviates  0.011		
7.3391	1389	0.017	----	21.6599		----			
7.3659	1749	0.024	----	21.6962		----			
7.4593	5411	0.024	0.957	21.8232	22:1 w8c	0.68	ECL deviates  0.010		
7.5433	1133	0.017	0.956	21.9373	22:1 w3c	0.14	ECL deviates -0.010		
7.5889	5129	0.016	0.956	21.9993	22:0	0.64	ECL deviates -0.001	Reference -0.008	
7.7819	117537	0.019	----	22.2657		----			
8.0851	2016	0.017	----	22.6843		----			
8.1560	783	0.019	----	22.7822		----			
8.2581	1800	0.015	0.960	22.9232	23:1 w4c	0.23	ECL deviates -0.003		
8.3137	1083	0.017	0.960	22.9998	23:0	0.14	ECL deviates  0.000	Reference -0.008	
8.5246	1650	0.018	----	23.2974		----			
8.7861	2200	0.028	----	23.6665		----			
8.8357	2063	0.019	----	23.7367		----			
8.9416	3062	0.020	----	23.8861		----			
9.0217	4496	0.018	0.975	23.9991	24:0	0.57	ECL deviates -0.001	Reference -0.008	
9.3903	10365	0.018	----	24.5195		----	> max rt		

ECL Deviation: 0.005                            Reference ECL Shift: 0.008       Number Reference Peaks: 21
Total Response: 946010                         Total Named: 749520
Percent Named: 79.23%                         Total Amount: 765795

(No search libraries specified in method PLFAD1.)
