# Supplementary material for: Microbial regulation of soil carbon properties under nitrogen addition and plant inputs removal
Source: PeerJ. 2019 Jul 17;7:e7343. doi: 10.7717/peerj.7343 (PMC6642627; doi:10.7717/peerj.7343)
Supplement: File S1 — The raw data showed the soil microbial PLFAs files in the year of 2015 and 2016. Each file of rtf. represented the microbial PLFAs for each soil sample. In the Supplemental File, the Excel file named “Numbers” showed the plots names and the related rtf. file names. [file peerj-07-7343-s002.zip › supplementary files/2016/60.rtf]

Volume: DATA            File: E17C203.64A       Samp Ctr: 13                 ID Number: 5033 
Type: Samp                   Bottle: 24                      Method: PLFAD1 
Created: 12/20/2017 2:25:22 PM 
Sample ID: 60 


RT	Response	Ar/Ht	RFact	ECL	Peak Name	Percent	Comment1	Comment2	
0.7658	1.684E+9	0.016	----	7.6932	SOLVENT PEAK	----	< min rt		
1.8107	658	0.013	1.030	12.7176	13:0 anteiso	0.10	ECL deviates  0.008	Reference  0.011	
1.9893	1015	0.019	----	13.2268		----			
2.1402	4670	0.015	1.043	13.6063	14:0 iso	0.71	ECL deviates -0.008	Reference -0.007	
2.1872	995	0.016	1.043	13.7245	14:0 anteiso	0.15	ECL deviates  0.009	Reference  0.009	
2.2151	683	0.014	1.044	13.7948	14:1 w8c	0.10	ECL deviates -0.007		
2.2689	727	0.013	----	13.9300		----			
2.2948	5254	0.016	1.045	13.9951	14:0	0.81	ECL deviates -0.005	Reference -0.005	
2.3578	726	0.013	----	14.1264	14:0 iso 3OH	----	ECL deviates  0.002		
2.4584	785	0.015	----	14.3344		----			
2.5086	7848	0.018	1.046	14.4383	15:1 iso w6c	1.20	ECL deviates -0.001		
2.5524	1185	0.014	1.046	14.5289	15:1 anteiso w9c	0.18	ECL deviates -0.001		
2.5932	30752	0.015	1.046	14.6134	15:0 iso	4.72	ECL deviates -0.004	Reference -0.006	
2.6393	21513	0.016	1.046	14.7086	15:0 anteiso	3.30	ECL deviates -0.002	Reference -0.005	
2.7061	1450	0.015	1.045	14.8469	15:1 w7c	0.22	ECL deviates  0.010		
2.7796	3171	0.015	1.045	14.9988	15:0	0.49	ECL deviates -0.001	Reference -0.004	
2.8103	978	0.015	----	15.0537		----			
2.9134	1028	0.019	----	15.2356		----			
3.0328	4970	0.022	1.042	15.4464	15:0 DMA	0.76	ECL deviates -0.004		
3.1022	9969	0.015	1.041	15.5690	16:3 w6c	1.52	ECL deviates -0.007		
3.1308	12901	0.016	1.040	15.6195	16:0 iso	1.97	ECL deviates  0.000	Reference -0.004	
3.1871	1869	0.016	1.039	15.7188	16:0 anteiso	0.28	ECL deviates  0.004	Reference  0.000	
3.2163	6310	0.017	1.039	15.7704	16:1 w9c	0.96	ECL deviates -0.005		
3.2454	47140	0.017	1.038	15.8218	16:1 w7c	7.18	ECL deviates -0.003		
3.2973	15349	0.016	1.037	15.9134	16:1 w5c	2.34	ECL deviates  0.002		
3.3461	77218	0.016	1.036	15.9995	16:0	11.74	ECL deviates -0.001	Reference -0.005	
3.3777	2478	0.017	----	16.0499		----			
3.6143	31790	0.019	1.031	16.4232	16:0 10-methyl	4.81	ECL deviates  0.003		
3.6603	76582	0.016	1.030	16.4957	17:1 iso w9c	11.57	ECL deviates -0.002		
3.7411	9646	0.015	1.028	16.6231	17:0 iso	1.45	ECL deviates -0.001	Reference -0.006	
3.8019	10196	0.016	1.027	16.7190	17:0 anteiso	1.54	ECL deviates -0.001		
3.8501	4149	0.019	1.026	16.7950	17:1 w8c	0.62	ECL deviates -0.002		
3.9142	23175	0.018	1.024	16.8961	17:0 cyclo w7c	3.48	ECL deviates  0.003		
3.9804	3282	0.018	1.023	17.0005	17:0	0.49	ECL deviates  0.001	Reference -0.006	
4.0079	3355	0.015	1.022	17.0411	17:1 w7c 10-methyl	0.50	ECL deviates -0.002		
4.0525	817	0.014	----	17.1062		----			
4.1422	863	0.015	1.019	17.2371	16:0 2OH	0.13	ECL deviates -0.003		
4.2568	4113	0.016	1.016	17.4044	17:0 10-methyl	0.61	ECL deviates -0.003		
4.3184	2178	0.026	----	17.4945		----			
4.3751	2190	0.016	1.013	17.5772	18:3 w6c	0.33	ECL deviates -0.003		
4.4034	3979	0.024	1.012	17.6185	18:0 iso	0.59	ECL deviates -0.008	Reference -0.015	
4.4769	11518	0.017	1.011	17.7257	18:2 w6c	1.71	ECL deviates -0.001		
4.5089	37582	0.018	1.010	17.7725	18:1 w9c	5.57	ECL deviates -0.002		
4.5454	68076	0.017	1.009	17.8258	18:1 w7c	10.07	ECL deviates -0.001		
4.6044	10000	0.021	----	17.9119		----			
4.6649	13425	0.018	1.006	18.0002	18:0	1.98	ECL deviates  0.000	Reference -0.007	
4.7236	6505	0.019	1.004	18.0825	18:1 w7c 10-methyl	0.96	ECL deviates -0.003		
4.7824	1483	0.024	1.003	18.1647	18:2 DMA	0.22	ECL deviates  0.005		
4.8288	977	0.023	1.002	18.2297	18:1 w9c DMA	0.14	ECL deviates -0.007		
4.9436	17148	0.021	0.999	18.3901	18:0 10-methyl	2.51	ECL deviates -0.005		
5.0607	2595	0.016	0.996	18.5538	19:3 w6c	0.38	ECL deviates -0.006		
5.1972	2241	0.025	----	18.7446		----			
5.2456	2232	0.017	0.992	18.8122	19:1 w8c	0.32	ECL deviates  0.001		
5.2877	3252	0.018	0.991	18.8710	19:0 cyclo w9c	0.47	ECL deviates -0.001		
5.3137	19645	0.018	0.990	18.9074	19:0 cyclo w7c	2.85	ECL deviates -0.002		
5.3828	60034	0.018	----	19.0039	19:0	----	ECL deviates  0.004		
5.5349	1047	0.016	----	19.2103		----			
5.5800	1489	0.015	----	19.2714		----			
5.6499	2413	0.019	----	19.3660		----			
5.6737	1092	0.014	0.982	19.3983	20:4 w6c	0.16	ECL deviates -0.005		
5.8239	2437	0.028	----	19.6018		----			
5.9023	1579	0.018	----	19.7081		----			
5.9479	5162	0.032	0.976	19.7700	20:1 w9c	0.74	ECL deviates -0.003		
6.1163	4328	0.019	0.973	19.9982	20:0	0.62	ECL deviates -0.002	Reference -0.009	
6.2581	1086	0.014	----	20.1907		----			
6.3731	2608	0.013	----	20.3469		----			
6.4024	22581	0.019	0.968	20.3867	20:0 10-methyl	3.20	ECL deviates -0.010		
6.5719	2192	0.019	----	20.6171		----			
6.6531	2424	0.022	----	20.7274		----			
6.7042	1792	0.015	0.963	20.7968	21:1 w8c	0.25	ECL deviates -0.001		
6.7659	1630	0.017	----	20.8806		----			
6.8199	2429	0.016	0.962	20.9539	21:1 w3c	0.34	ECL deviates  0.000		
6.8677	978	0.020	----	21.0188		----		Reference  0.012	
7.0619	1320	0.018	----	21.2826		----			
7.4591	3264	0.021	0.957	21.8217	22:1 w8c	0.46	ECL deviates  0.008		
7.5423	767	0.016	0.956	21.9347	22:1 w3c	0.11	ECL deviates -0.012		
7.5895	5132	0.016	0.956	21.9987	22:0	0.72	ECL deviates -0.001	Reference -0.007	
7.7786	87894	0.019	----	22.2594		----			
7.9299	810	0.021	0.957	22.4679	23:4 w6c	0.11	ECL deviates -0.003		
8.0871	1768	0.017	----	22.6847		----			
8.1533	878	0.018	----	22.7760		----			
8.2576	2425	0.016	0.960	22.9198	23:1 w4c	0.34	ECL deviates -0.007		
8.3133	1399	0.020	0.960	22.9965	23:0	0.20	ECL deviates -0.003	Reference -0.008	
8.5241	1664	0.017	----	23.2934		----			
8.7919	2107	0.030	----	23.6709		----			
8.8355	2020	0.022	----	23.7323		----			
8.9400	1910	0.017	----	23.8795		----			
9.0226	4967	0.018	0.975	23.9959	24:0	0.71	ECL deviates -0.004	Reference -0.007	
9.3889	7054	0.019	----	24.5119		----	> max rt		

ECL Deviation: 0.005                            Reference ECL Shift: 0.008       Number Reference Peaks: 19
Total Response: 809502                         Total Named: 667812
Percent Named: 82.50%                         Total Amount: 681884

(No search libraries specified in method PLFAD1.)
