# Supplementary material for: Microbial regulation of soil carbon properties under nitrogen addition and plant inputs removal
Source: PeerJ. 2019 Jul 17;7:e7343. doi: 10.7717/peerj.7343 (PMC6642627; doi:10.7717/peerj.7343)
Supplement: File S1 — The raw data showed the soil microbial PLFAs files in the year of 2015 and 2016. Each file of rtf. represented the microbial PLFAs for each soil sample. In the Supplemental File, the Excel file named “Numbers” showed the plots names and the related rtf. file names. [file peerj-07-7343-s002.zip › supplementary files/2016/61.rtf]

Volume: DATA            File: E17C203.64A       Samp Ctr: 16                 ID Number: 5034 
Type: Samp                   Bottle: 2                        Method: PLFAD1 
Created: 12/20/2017 3:31:39 PM 
Sample ID: 61 


RT	Response	Ar/Ht	RFact	ECL	Peak Name	Percent	Comment1	Comment2	
0.7647	1.702E+9	0.016	----	7.6926	SOLVENT PEAK	----	< min rt		
0.9511	705	0.011	----	8.7545		----	< min rt		
1.8112	985	0.013	1.012	12.7197	13:0 anteiso	0.20	ECL deviates  0.010	Reference  0.017	
1.9903	1263	0.014	----	13.2294		----			
2.1397	3594	0.014	1.030	13.6057	14:0 iso	0.76	ECL deviates -0.008	Reference -0.004	
2.1604	409	0.009	----	13.6579		----			
2.1861	1243	0.013	1.032	13.7227	14:0 anteiso	0.26	ECL deviates  0.007	Reference  0.011	
2.2145	801	0.013	1.033	13.7941	14:1 w8c	0.17	ECL deviates -0.008		
2.2694	606	0.012	----	13.9327		----			
2.2946	3579	0.014	1.035	13.9961	14:0	0.76	ECL deviates -0.004	Reference -0.001	
2.3570	1561	0.015	----	14.1251	14:0 iso 3OH	----	ECL deviates  0.000		
2.4569	633	0.014	----	14.3315		----			
2.5085	4417	0.017	1.038	14.4382	15:1 iso w6c	0.94	ECL deviates -0.001		
2.5313	719	0.011	1.038	14.4853	15:4 w3c	0.15	ECL deviates -0.005		
2.5533	824	0.015	1.038	14.5309	15:1 anteiso w9c	0.18	ECL deviates  0.001		
2.5924	22111	0.015	1.038	14.6116	15:0 iso	4.70	ECL deviates -0.005	Reference -0.004	
2.6390	15659	0.015	1.039	14.7078	15:0 anteiso	3.33	ECL deviates -0.003	Reference -0.002	
2.7053	2373	0.018	1.039	14.8448	15:1 w7c	0.50	ECL deviates  0.008		
2.7794	2378	0.016	1.039	14.9980	15:0	0.51	ECL deviates -0.002	Reference -0.001	
2.8108	975	0.015	----	15.0535		----			
3.0325	3742	0.021	1.037	15.4451	15:0 DMA	0.80	ECL deviates -0.005		
3.1021	12206	0.016	1.037	15.5679	16:3 w6c	2.59	ECL deviates -0.008		
3.1304	8909	0.016	1.036	15.6180	16:0 iso	1.89	ECL deviates -0.002	Reference -0.002	
3.1552	577	0.011	----	15.6618		----			
3.1866	1779	0.014	1.036	15.7171	16:0 anteiso	0.38	ECL deviates  0.002	Reference  0.001	
3.2171	3910	0.015	1.035	15.7710	16:1 w9c	0.83	ECL deviates -0.004		
3.2451	29442	0.018	1.035	15.8204	16:1 w7c	6.24	ECL deviates -0.004		
3.2972	8531	0.017	1.034	15.9125	16:1 w5c	1.81	ECL deviates  0.001		
3.3462	42359	0.015	1.034	15.9989	16:0	8.97	ECL deviates -0.001	Reference -0.002	
3.3767	2021	0.016	----	16.0474		----			
3.6148	18895	0.019	1.030	16.4240	16:0 10-methyl	3.98	ECL deviates  0.004		
3.6606	101476	0.017	1.029	16.4964	17:1 iso w9c	21.38	ECL deviates -0.002		
3.7412	5692	0.015	1.027	16.6238	17:0 iso	1.20	ECL deviates  0.000	Reference -0.002	
3.8022	6393	0.016	1.026	16.7203	17:0 anteiso	1.34	ECL deviates  0.000		
3.8508	2766	0.018	1.025	16.7972	17:1 w8c	0.58	ECL deviates  0.000		
3.9132	12028	0.019	1.024	16.8958	17:0 cyclo w7c	2.52	ECL deviates  0.002		
3.9804	1880	0.017	1.022	17.0020	17:0	0.39	ECL deviates  0.002	Reference -0.001	
4.0071	2860	0.016	1.022	17.0411	17:1 w7c 10-methyl	0.60	ECL deviates -0.002		
4.0538	587	0.014	----	17.1093		----			
4.1216	1039	0.017	----	17.2082		----			
4.1414	661	0.014	1.019	17.2371	16:0 2OH	0.14	ECL deviates -0.003		
4.2582	2978	0.017	1.017	17.4077	17:0 10-methyl	0.62	ECL deviates  0.001		
4.3178	1570	0.026	----	17.4946		----			
4.3769	1805	0.016	1.014	17.5809	18:3 w6c	0.38	ECL deviates  0.001		
4.4070	1790	0.017	1.013	17.6248	18:0 iso	0.37	ECL deviates -0.002	Reference -0.005	
4.4343	850	0.015	----	17.6647		----			
4.4775	8355	0.017	1.012	17.7277	18:2 w6c	1.73	ECL deviates  0.001		
4.5091	21869	0.018	1.011	17.7738	18:1 w9c	4.53	ECL deviates -0.001		
4.5466	33190	0.018	1.010	17.8286	18:1 w7c	6.87	ECL deviates  0.002		
4.6071	4602	0.022	1.009	17.9168	18:1 w5c	0.95	ECL deviates -0.006		
4.6671	7428	0.018	1.008	18.0044	18:0	1.53	ECL deviates  0.004	Reference  0.001	
4.7250	2740	0.015	1.006	18.0853	18:1 w7c 10-methyl	0.56	ECL deviates  0.000		
4.9442	12057	0.020	1.002	18.3914	18:0 10-methyl	2.47	ECL deviates -0.004		
5.0618	3584	0.019	0.999	18.5556	19:3 w6c	0.73	ECL deviates -0.004		
5.1970	1975	0.026	----	18.7443		----			
5.2495	1523	0.017	0.995	18.8176	19:1 w8c	0.31	ECL deviates  0.007		
5.2804	2265	0.020	0.995	18.8607	19:1 w6c	0.46	ECL deviates  0.009		
5.3151	8290	0.016	0.994	18.9092	19:0 cyclo w7c	1.69	ECL deviates -0.001		
5.3849	62128	0.017	----	19.0065	19:0	----	ECL deviates  0.006		
5.5807	620	0.013	----	19.2726		----			
5.6503	1462	0.022	----	19.3672		----			
5.6762	736	0.014	0.987	19.4024	20:4 w6c	0.15	ECL deviates -0.001		
5.7969	671	0.016	0.984	19.5664	20:3 w6c	0.14	ECL deviates  0.000		
5.8232	840	0.016	----	19.6022		----			
5.9019	1886	0.020	----	19.7091		----			
5.9492	1337	0.019	0.982	19.7734	20:1 w9c	0.27	ECL deviates  0.001		
5.9746	851	0.017	0.981	19.8080	20:1 w8c	0.17	ECL deviates -0.005		
6.1174	2000	0.019	0.979	20.0020	20:0	0.40	ECL deviates  0.002	Reference -0.003	
6.3748	3312	0.014	----	20.3512		----			
6.4041	29120	0.017	0.975	20.3910	20:0 10-methyl	5.82	ECL deviates -0.006		
6.5718	2622	0.018	----	20.6185		----			
6.6545	2730	0.023	----	20.7306		----			
6.7052	1469	0.017	0.972	20.7994	21:1 w8c	0.29	ECL deviates  0.001		
6.7691	874	0.019	----	20.8861		----			
6.8227	1495	0.016	0.971	20.9589	21:1 w3c	0.30	ECL deviates  0.005		
7.3694	1001	0.016	----	21.7024		----			
7.4598	2981	0.023	----	21.8252		----			
7.5911	2138	0.015	0.970	22.0038	22:0	0.42	ECL deviates  0.004	Reference -0.001	
7.7819	116580	0.018	----	22.2670		----			
8.0879	2386	0.018	----	22.6890		----			
8.2586	1219	0.015	0.978	22.9243	23:1 w4c	0.24	ECL deviates -0.002		
8.5259	762	0.017	----	23.2977		----			
8.7988	1796	0.025	----	23.6802		----			
8.8335	889	0.018	----	23.7289		----			
8.9423	1871	0.020	----	23.8813		----			
9.0242	2336	0.019	1.001	23.9961	24:0	0.48	ECL deviates -0.004	Reference -0.006	
9.3887	9561	0.017	----	24.5069		----	> max rt		
9.4910	564	0.013	----	24.6502		----	> max rt		

ECL Deviation: 0.004                            Reference ECL Shift: 0.006       Number Reference Peaks: 17
Total Response: 633173                         Total Named: 478058
Percent Named: 75.50%                         Total Amount: 488211

(No search libraries specified in method PLFAD1.)
