# Supplementary material for: Microbial regulation of soil carbon properties under nitrogen addition and plant inputs removal
Source: PeerJ. 2019 Jul 17;7:e7343. doi: 10.7717/peerj.7343 (PMC6642627; doi:10.7717/peerj.7343)
Supplement: File S1 — The raw data showed the soil microbial PLFAs files in the year of 2015 and 2016. Each file of rtf. represented the microbial PLFAs for each soil sample. In the Supplemental File, the Excel file named “Numbers” showed the plots names and the related rtf. file names. [file peerj-07-7343-s002.zip › supplementary files/2016/62.rtf]

Volume: DATA            File: E17C203.64A       Samp Ctr: 17                 ID Number: 5035 
Type: Samp                   Bottle: 3                        Method: PLFAD1 
Created: 12/20/2017 4:00:13 PM 
Sample ID: 62 


RT	Response	Ar/Ht	RFact	ECL	Peak Name	Percent	Comment1	Comment2	
0.7650	1.68E+9	0.016	----	7.6970	SOLVENT PEAK	----	< min rt		
0.9519	1024	0.012	----	8.7618		----	< min rt		
1.5246	1181	0.024	----	11.7501		----			
1.5857	947	0.021	0.989	12.0041	12:0	0.15	ECL deviates  0.004	Reference  0.008	
1.6473	597	0.011	----	12.2010		----			
1.7718	710	0.013	1.009	12.5987	13:0 iso	0.11	ECL deviates -0.014	Reference -0.011	
1.8101	1397	0.012	1.012	12.7210	13:0 anteiso	0.23	ECL deviates  0.012	Reference  0.014	
1.9886	1551	0.016	----	13.2292		----			
2.1393	5250	0.016	1.030	13.6085	14:0 iso	0.86	ECL deviates -0.006	Reference -0.005	
2.1584	596	0.009	----	13.6566		----			
2.1855	1684	0.013	1.032	13.7248	14:0 anteiso	0.28	ECL deviates  0.009	Reference  0.009	
2.2152	1232	0.016	1.033	13.7995	14:1 w8c	0.20	ECL deviates -0.002		
2.2688	944	0.014	----	13.9344		----			
2.2940	5660	0.015	1.035	13.9979	14:0	0.94	ECL deviates -0.002	Reference -0.002	
2.3574	1740	0.014	----	14.1292	14:0 iso 3OH	----	ECL deviates  0.004		
2.4556	857	0.014	----	14.3320		----			
2.5074	6479	0.019	1.038	14.4389	15:1 iso w6c	1.07	ECL deviates  0.000		
2.5308	1112	0.012	1.038	14.4872	15:4 w3c	0.18	ECL deviates -0.003		
2.5507	1042	0.012	1.038	14.5283	15:1 anteiso w9c	0.17	ECL deviates -0.002		
2.5922	30859	0.015	1.038	14.6140	15:0 iso	5.12	ECL deviates -0.003	Reference -0.004	
2.6382	21242	0.015	1.039	14.7089	15:0 anteiso	3.53	ECL deviates -0.002	Reference -0.003	
2.7051	2481	0.017	1.039	14.8470	15:1 w7c	0.41	ECL deviates  0.010		
2.7786	3115	0.016	1.039	14.9988	15:0	0.52	ECL deviates -0.001	Reference -0.003	
2.8087	1227	0.015	----	15.0524		----			
2.9122	565	0.014	----	15.2350		----			
3.0054	754	0.013	1.038	15.3994	16:1 w7c alcohol	0.13	ECL deviates  0.003		
3.0302	3716	0.016	1.037	15.4433	15:0 DMA	0.62	ECL deviates -0.007		
3.1005	14454	0.016	1.037	15.5673	16:3 w6c	2.40	ECL deviates -0.009		
3.1296	10960	0.015	1.036	15.6186	16:0 iso	1.82	ECL deviates -0.001	Reference -0.004	
3.1536	642	0.010	----	15.6610		----			
3.1864	2198	0.015	1.036	15.7189	16:0 anteiso	0.36	ECL deviates  0.004	Reference  0.001	
3.2147	5659	0.016	1.035	15.7687	16:1 w9c	0.94	ECL deviates -0.006		
3.2443	38907	0.017	1.035	15.8211	16:1 w7c	6.44	ECL deviates -0.003		
3.2958	12010	0.017	1.034	15.9118	16:1 w5c	1.99	ECL deviates  0.001		
3.3455	58287	0.016	1.034	15.9996	16:0	9.63	ECL deviates  0.000	Reference -0.003	
3.3755	2752	0.018	----	16.0474		----			
3.6129	26568	0.019	1.030	16.4225	16:0 10-methyl	4.37	ECL deviates  0.003		
3.6593	104270	0.016	1.029	16.4960	17:1 iso w9c	17.14	ECL deviates -0.002		
3.7397	7664	0.015	1.027	16.6231	17:0 iso	1.26	ECL deviates -0.001	Reference -0.004	
3.8006	8789	0.016	1.026	16.7193	17:0 anteiso	1.44	ECL deviates -0.001		
3.8500	3697	0.017	1.025	16.7973	17:1 w8c	0.61	ECL deviates  0.000		
3.9123	16448	0.019	1.024	16.8958	17:0 cyclo w7c	2.69	ECL deviates  0.002		
3.9789	2442	0.016	1.022	17.0011	17:0	0.40	ECL deviates  0.001	Reference -0.003	
4.0068	3366	0.016	1.022	17.0420	17:1 w7c 10-methyl	0.55	ECL deviates -0.001		
4.0519	660	0.014	----	17.1078		----			
4.1171	1016	0.018	----	17.2030		----			
4.1402	690	0.015	1.019	17.2367	16:0 2OH	0.11	ECL deviates -0.003		
4.2564	3439	0.015	1.017	17.4063	17:0 10-methyl	0.56	ECL deviates -0.001		
4.3184	1818	0.026	----	17.4968		----			
4.3755	2027	0.016	1.014	17.5801	18:3 w6c	0.33	ECL deviates  0.000		
4.4054	2279	0.017	1.013	17.6237	18:0 iso	0.37	ECL deviates -0.003	Reference -0.008	
4.4332	918	0.016	----	17.6642		----			
4.4757	9088	0.017	1.012	17.7263	18:2 w6c	1.47	ECL deviates -0.001		
4.5081	27115	0.019	1.011	17.7736	18:1 w9c	4.38	ECL deviates -0.001		
4.5443	44566	0.019	1.010	17.8264	18:1 w7c	7.20	ECL deviates -0.001		
4.6048	6515	0.020	1.009	17.9147	18:1 w5c	1.05	ECL deviates -0.008		
4.6644	10152	0.018	1.008	18.0017	18:0	1.64	ECL deviates  0.002	Reference -0.003	
4.7236	4178	0.017	1.006	18.0845	18:1 w7c 10-methyl	0.67	ECL deviates -0.001		
4.7837	960	0.019	1.005	18.1684	18:2 DMA	0.15	ECL deviates  0.008		
4.8191	1792	0.026	----	18.2178		----			
4.9432	14236	0.021	1.002	18.3911	18:0 10-methyl	2.28	ECL deviates -0.004		
5.0611	4200	0.018	0.999	18.5557	19:3 w6c	0.67	ECL deviates -0.004		
5.1968	1763	0.025	----	18.7451		----			
5.2456	1948	0.017	0.995	18.8133	19:1 w8c	0.31	ECL deviates  0.002		
5.3124	17297	0.020	0.994	18.9065	19:0 cyclo w7c	2.75	ECL deviates -0.003		
5.3826	57775	0.018	----	19.0045	19:0	----	ECL deviates  0.004		
5.5352	1313	0.017	----	19.2120		----			
5.5771	897	0.014	----	19.2690		----			
5.6502	1519	0.018	----	19.3683		----			
5.6743	1115	0.015	0.987	19.4010	20:4 w6c	0.18	ECL deviates -0.002		
5.7942	827	0.016	0.984	19.5639	20:3 w6c	0.13	ECL deviates -0.002		
5.8249	1594	0.020	----	19.6057		----			
5.9012	1756	0.019	----	19.7095		----			
5.9447	2593	0.022	0.982	19.7685	20:1 w9c	0.41	ECL deviates -0.004		
5.9725	1455	0.023	0.981	19.8063	20:1 w8c	0.23	ECL deviates -0.007		
6.1153	3174	0.017	0.979	20.0004	20:0	0.50	ECL deviates  0.000	Reference -0.006	
6.2587	889	0.017	----	20.1951		----			
6.3713	4102	0.015	----	20.3479		----			
6.4026	32786	0.019	0.975	20.3904	20:0 10-methyl	5.11	ECL deviates -0.007		
6.5704	3635	0.021	----	20.6181		----			
6.6479	3223	0.024	----	20.7232		----			
6.7047	1639	0.016	0.972	20.8003	21:1 w8c	0.25	ECL deviates  0.002		
6.7654	1571	0.019	----	20.8828		----			
6.8203	2017	0.016	0.971	20.9572	21:1 w3c	0.31	ECL deviates  0.003		
6.8701	2327	0.032	----	21.0249		----			
7.0599	809	0.014	----	21.2831		----			
7.3141	2028	0.028	0.969	21.6291	22:0 iso	0.31	ECL deviates  0.011		
7.3352	886	0.012	----	21.6579		----			
7.3645	2576	0.021	----	21.6977		----			
7.4590	5778	0.025	----	21.8263		----			
7.5406	1001	0.017	0.969	21.9374	22:1 w3c	0.16	ECL deviates -0.010		
7.5884	4842	0.018	0.970	22.0024	22:0	0.75	ECL deviates  0.002	Reference -0.004	
7.7802	118094	0.020	----	22.2670		----			
8.0854	3127	0.018	----	22.6882		----			
8.1525	812	0.019	----	22.7809		----			
8.2575	1760	0.016	0.978	22.9257	23:1 w4c	0.28	ECL deviates -0.001		
8.3134	1012	0.017	0.979	23.0029	23:0	0.16	ECL deviates  0.003	Reference -0.004	
8.5241	1117	0.016	----	23.2985		----			
8.7927	3314	0.026	----	23.6754		----			
8.8342	2124	0.022	----	23.7335		----			
8.9401	2954	0.017	----	23.8821		----			
9.0212	4638	0.017	1.001	23.9959	24:0	0.74	ECL deviates -0.004	Reference -0.011	
9.3853	15040	0.018	----	24.5068		----	> max rt		
9.4896	991	0.016	----	24.6531		----	> max rt		

ECL Deviation: 0.005                            Reference ECL Shift: 0.006       Number Reference Peaks: 20
Total Response: 796271                         Total Named: 612975
Percent Named: 76.98%                         Total Amount: 625620

(No search libraries specified in method PLFAD1.)
