# Supplementary material for: Microbial regulation of soil carbon properties under nitrogen addition and plant inputs removal
Source: PeerJ. 2019 Jul 17;7:e7343. doi: 10.7717/peerj.7343 (PMC6642627; doi:10.7717/peerj.7343)
Supplement: File S1 — The raw data showed the soil microbial PLFAs files in the year of 2015 and 2016. Each file of rtf. represented the microbial PLFAs for each soil sample. In the Supplemental File, the Excel file named “Numbers” showed the plots names and the related rtf. file names. [file peerj-07-7343-s002.zip › supplementary files/2016/63.rtf]

Volume: DATA            File: E17C203.64A       Samp Ctr: 18                 ID Number: 5036 
Type: Samp                   Bottle: 4                        Method: PLFAD1 
Created: 12/20/2017 4:29:37 PM 
Sample ID: 63 


RT	Response	Ar/Ht	RFact	ECL	Peak Name	Percent	Comment1	Comment2	
0.7650	1.691E+9	0.016	----	7.7066	SOLVENT PEAK	----	< min rt		
0.9508	724	0.012	----	8.7641		----	< min rt		
1.9889	1155	0.018	----	13.2377		----			
2.1390	2858	0.014	1.030	13.6143	14:0 iso	0.60	ECL deviates  0.000	Reference -0.006	
2.2684	767	0.013	----	13.9393		----			
2.2934	2933	0.015	1.035	14.0019	14:0	0.62	ECL deviates  0.002	Reference -0.004	
2.3563	1204	0.012	----	14.1322	14:0 iso 3OH	----	ECL deviates  0.007		
2.4560	819	0.014	----	14.3375		----			
2.5067	3451	0.016	1.038	14.4419	15:1 iso w6c	0.73	ECL deviates  0.003		
2.5355	697	0.013	1.038	14.5012	15:4 w3c	0.15	ECL deviates  0.011		
2.5493	628	0.014	1.038	14.5296	15:1 anteiso w9c	0.13	ECL deviates  0.000		
2.5913	17982	0.015	1.038	14.6162	15:0 iso	3.79	ECL deviates -0.001	Reference -0.006	
2.6375	13326	0.015	1.039	14.7113	15:0 anteiso	2.81	ECL deviates  0.000	Reference -0.005	
2.7781	2094	0.015	1.039	15.0008	15:0	0.44	ECL deviates  0.001	Reference -0.004	
2.8093	939	0.015	----	15.0564		----			
3.0308	3051	0.021	1.037	15.4464	15:0 DMA	0.64	ECL deviates -0.004		
3.1001	13888	0.015	1.037	15.5684	16:3 w6c	2.92	ECL deviates -0.007		
3.1294	8487	0.017	1.036	15.6200	16:0 iso	1.79	ECL deviates  0.000	Reference -0.004	
3.1847	1091	0.014	1.036	15.7175	16:0 anteiso	0.23	ECL deviates  0.003	Reference -0.002	
3.2143	3807	0.016	1.035	15.7696	16:1 w9c	0.80	ECL deviates -0.005		
3.2437	29338	0.017	1.035	15.8213	16:1 w7c	6.16	ECL deviates -0.003		
3.2952	8032	0.016	1.034	15.9121	16:1 w5c	1.69	ECL deviates  0.001		
3.3444	42008	0.016	1.034	15.9987	16:0	8.81	ECL deviates -0.001	Reference -0.005	
3.3762	3169	0.019	----	16.0495		----			
3.6130	20462	0.019	1.030	16.4232	16:0 10-methyl	4.28	ECL deviates  0.003		
3.6590	109782	0.016	1.029	16.4957	17:1 iso w9c	22.92	ECL deviates -0.002		
3.7395	5700	0.016	1.027	16.6227	17:0 iso	1.19	ECL deviates -0.001	Reference -0.005	
3.7996	5822	0.017	1.026	16.7176	17:0 anteiso	1.21	ECL deviates -0.003		
3.8482	2519	0.016	1.025	16.7942	17:1 w8c	0.52	ECL deviates -0.003		
3.9117	13042	0.018	1.024	16.8945	17:0 cyclo w7c	2.71	ECL deviates  0.001		
3.9787	1739	0.015	1.022	17.0002	17:0	0.36	ECL deviates  0.000	Reference -0.003	
4.0055	3075	0.015	1.022	17.0396	17:1 w7c 10-methyl	0.64	ECL deviates -0.004		
4.0505	715	0.016	----	17.1052		----			
4.2549	2520	0.015	1.017	17.4030	17:0 10-methyl	0.52	ECL deviates -0.004		
4.3147	1437	0.025	----	17.4902		----			
4.3736	1881	0.016	1.014	17.5761	18:3 w6c	0.39	ECL deviates -0.004		
4.4030	1728	0.016	1.013	17.6189	18:0 iso	0.36	ECL deviates -0.008	Reference -0.011	
4.4305	782	0.016	----	17.6590		----			
4.4751	6600	0.015	1.012	17.7241	18:2 w6c	1.36	ECL deviates -0.003		
4.5075	21681	0.019	1.011	17.7714	18:1 w9c	4.45	ECL deviates -0.003		
4.5435	33734	0.018	1.010	17.8238	18:1 w7c	6.92	ECL deviates -0.003		
4.6036	4563	0.020	----	17.9115		----			
4.6631	9127	0.017	1.008	17.9981	18:0	1.87	ECL deviates -0.002	Reference -0.005	
4.7226	3052	0.017	1.006	18.0813	18:1 w7c 10-methyl	0.62	ECL deviates -0.004		
4.7879	969	0.017	1.005	18.1725	18:2 DMA	0.20	ECL deviates  0.012		
4.8156	2017	0.023	----	18.2111		----			
4.9417	11041	0.021	1.002	18.3870	18:0 10-methyl	2.24	ECL deviates -0.008		
5.0590	3742	0.024	0.999	18.5507	19:3 w6c	0.76	ECL deviates -0.009		
5.1956	2051	0.025	----	18.7412		----			
5.2457	1707	0.016	0.995	18.8111	19:1 w8c	0.34	ECL deviates  0.000		
5.2790	2654	0.020	0.995	18.8576	19:1 w6c	0.54	ECL deviates  0.006		
5.3117	11356	0.019	0.994	18.9031	19:0 cyclo w7c	2.29	ECL deviates -0.007		
5.3814	59095	0.018	----	19.0004	19:0	----	ECL deviates  0.000		
5.5777	915	0.013	----	19.2671		----			
5.6739	1716	0.027	0.987	19.3979	20:4 w6c	0.34	ECL deviates -0.005		
5.8225	1713	0.028	----	19.5998		----			
5.9016	1514	0.019	----	19.7074		----			
5.9473	1693	0.021	0.982	19.7695	20:1 w9c	0.34	ECL deviates -0.003		
5.9730	1134	0.018	0.981	19.8043	20:1 w8c	0.23	ECL deviates -0.009		
6.1150	3108	0.019	0.979	19.9973	20:0	0.62	ECL deviates -0.003	Reference -0.006	
6.3256	1066	0.021	----	20.2831		----			
6.3702	3867	0.016	----	20.3437		----			
6.4011	27079	0.019	0.975	20.3857	20:0 10-methyl	5.36	ECL deviates -0.011		
6.4408	710	0.012	----	20.4395		----			
6.4663	1326	0.019	----	20.4741		----			
6.5688	2879	0.022	----	20.6131		----			
6.6485	3868	0.028	----	20.7214		----			
6.7034	1920	0.018	0.972	20.7959	21:1 w8c	0.38	ECL deviates -0.002		
6.7672	1059	0.021	----	20.8825		----			
6.8219	3267	0.018	0.971	20.9568	21:1 w3c	0.64	ECL deviates  0.003		
6.8635	1352	0.024	0.971	21.0132	21:0	0.27	ECL deviates  0.013	Reference  0.009	
7.0606	874	0.016	----	21.2816		----			
7.3150	1210	0.028	0.969	21.6280	22:0 iso	0.24	ECL deviates  0.010		
7.3661	2124	0.018	----	21.6975		----			
7.4577	3703	0.020	0.969	21.8223	22:1 w8c	0.73	ECL deviates  0.009		
7.5415	994	0.015	0.969	21.9364	22:1 w3c	0.20	ECL deviates -0.011		
7.5883	3370	0.015	0.970	22.0002	22:0	0.66	ECL deviates  0.000	Reference -0.005	
7.7805	122415	0.018	----	22.2656		----			
8.0854	3244	0.019	----	22.6867		----			
8.1511	831	0.019	----	22.7776		----			
8.2571	1862	0.016	0.978	22.9240	23:1 w4c	0.37	ECL deviates -0.002		
8.5257	1076	0.019	----	23.3000		----			
8.7969	3226	0.022	----	23.6809		----			
8.9395	2558	0.018	----	23.8814		----			
9.0208	3129	0.018	1.001	23.9957	24:0	0.64	ECL deviates -0.004	Reference -0.011	
9.3865	7287	0.020	----	24.5095		----	> max rt		
9.4896	2223	0.018	----	24.6544		----	> max rt		

ECL Deviation: 0.005                            Reference ECL Shift: 0.006       Number Reference Peaks: 16
Total Response: 656816                         Total Named: 483137
Percent Named: 73.56%                         Total Amount: 492613

(No search libraries specified in method PLFAD1.)
