# Supplementary material for: Microbial regulation of soil carbon properties under nitrogen addition and plant inputs removal
Source: PeerJ. 2019 Jul 17;7:e7343. doi: 10.7717/peerj.7343 (PMC6642627; doi:10.7717/peerj.7343)
Supplement: File S1 — The raw data showed the soil microbial PLFAs files in the year of 2015 and 2016. Each file of rtf. represented the microbial PLFAs for each soil sample. In the Supplemental File, the Excel file named “Numbers” showed the plots names and the related rtf. file names. [file peerj-07-7343-s002.zip › supplementary files/2016/64.rtf]

Volume: DATA            File: E17C203.64A       Samp Ctr: 19                 ID Number: 5037 
Type: Samp                   Bottle: 5                        Method: PLFAD1 
Created: 12/20/2017 4:57:59 PM 
Sample ID: 64 


RT	Response	Ar/Ht	RFact	ECL	Peak Name	Percent	Comment1	Comment2	
0.7653	1.687E+9	0.015	----	7.7017	SOLVENT PEAK	----	< min rt		
0.9520	660	0.011	----	8.7651		----	< min rt		
1.7730	710	0.014	1.009	12.6046	13:0 iso	0.08	ECL deviates -0.008	Reference -0.008	
1.8095	680	0.014	1.012	12.7214	13:0 anteiso	0.08	ECL deviates  0.012	Reference  0.012	
1.9906	1234	0.019	----	13.2361		----			
2.1398	8439	0.015	1.030	13.6115	14:0 iso	1.00	ECL deviates -0.003	Reference -0.004	
2.2664	959	0.014	----	13.9300		----			
2.2940	6447	0.014	1.035	13.9993	14:0	0.77	ECL deviates -0.001	Reference -0.002	
2.3572	1077	0.012	----	14.1303	14:0 iso 3OH	----	ECL deviates  0.005		
2.4545	759	0.013	----	14.3311		----			
2.5071	7880	0.018	1.038	14.4397	15:1 iso w6c	0.94	ECL deviates  0.001		
2.5298	1527	0.012	1.038	14.4865	15:4 w3c	0.18	ECL deviates -0.004		
2.5511	1406	0.012	1.038	14.5304	15:1 anteiso w9c	0.17	ECL deviates  0.000		
2.5925	44526	0.014	1.038	14.6159	15:0 iso	5.31	ECL deviates -0.001	Reference -0.004	
2.6377	33226	0.015	1.039	14.7092	15:0 anteiso	3.96	ECL deviates -0.002	Reference -0.004	
2.7091	830	0.016	1.039	14.8565	15:1 w6c	0.10	ECL deviates -0.004		
2.7789	4347	0.015	1.039	15.0005	15:0	0.52	ECL deviates  0.000	Reference -0.002	
2.8092	1992	0.014	----	15.0544		----			
3.0033	1194	0.013	1.038	15.3967	16:1 w7c alcohol	0.14	ECL deviates  0.000		
3.0308	6141	0.019	1.037	15.4453	15:0 DMA	0.73	ECL deviates -0.005		
3.1002	13956	0.015	1.037	15.5678	16:3 w6c	1.66	ECL deviates -0.008		
3.1295	18950	0.015	1.036	15.6194	16:0 iso	2.25	ECL deviates  0.000	Reference -0.004	
3.1845	2143	0.016	1.036	15.7164	16:0 anteiso	0.25	ECL deviates  0.001	Reference -0.002	
3.2153	8689	0.015	1.035	15.7708	16:1 w9c	1.03	ECL deviates -0.004		
3.2442	68911	0.017	1.035	15.8218	16:1 w7c	8.19	ECL deviates -0.003		
3.2963	18057	0.016	1.034	15.9137	16:1 w5c	2.14	ECL deviates  0.003		
3.3454	85861	0.016	1.034	16.0003	16:0	10.19	ECL deviates  0.000	Reference -0.004	
3.3755	5261	0.019	----	16.0482		----			
3.4731	692	0.015	----	16.2025		----			
3.6141	48246	0.020	1.030	16.4253	16:0 10-methyl	5.70	ECL deviates  0.005		
3.6595	105527	0.016	1.029	16.4970	17:1 iso w9c	12.46	ECL deviates -0.001		
3.7403	12438	0.016	1.027	16.6247	17:0 iso	1.47	ECL deviates  0.001	Reference -0.003	
3.8011	13248	0.018	1.026	16.7208	17:0 anteiso	1.56	ECL deviates  0.001		
3.8489	5518	0.018	1.025	16.7964	17:1 w8c	0.65	ECL deviates -0.001		
3.9132	28346	0.019	1.024	16.8980	17:0 cyclo w7c	3.33	ECL deviates  0.004		
3.9794	3630	0.017	1.022	17.0026	17:0	0.43	ECL deviates  0.003	Reference -0.002	
4.0070	6866	0.018	1.022	17.0429	17:1 w7c 10-methyl	0.81	ECL deviates  0.000		
4.0518	1398	0.014	----	17.1083		----			
4.1398	1089	0.018	1.019	17.2367	16:0 2OH	0.13	ECL deviates -0.004		
4.2563	5263	0.018	1.017	17.4066	17:0 10-methyl	0.61	ECL deviates  0.000		
4.3177	2454	0.024	----	17.4962		----			
4.3752	2484	0.018	1.014	17.5802	18:3 w6c	0.29	ECL deviates  0.000		
4.4022	3171	0.018	1.013	17.6197	18:0 iso	0.37	ECL deviates -0.007	Reference -0.012	
4.4349	987	0.016	----	17.6673		----			
4.4753	15427	0.017	1.012	17.7263	18:2 w6c	1.79	ECL deviates -0.001		
4.5078	46865	0.018	1.011	17.7737	18:1 w9c	5.44	ECL deviates -0.001		
4.5445	74202	0.017	1.010	17.8273	18:1 w7c	8.60	ECL deviates  0.000		
4.6079	8634	0.023	1.009	17.9197	18:1 w5c	1.00	ECL deviates -0.003		
4.6630	15197	0.016	1.008	18.0002	18:0	1.76	ECL deviates  0.000	Reference -0.005	
4.7223	6363	0.018	1.006	18.0832	18:1 w7c 10-methyl	0.73	ECL deviates -0.002		
4.7810	2203	0.027	1.005	18.1651	18:2 DMA	0.25	ECL deviates  0.005		
4.8344	1647	0.029	1.004	18.2396	18:1 w9c DMA	0.19	ECL deviates  0.003		
4.9425	24590	0.018	1.002	18.3905	18:0 10-methyl	2.83	ECL deviates -0.004		
5.0596	3401	0.020	0.999	18.5539	19:3 w6c	0.39	ECL deviates -0.006		
5.1390	708	0.017	0.998	18.6648	19:3 w3c	0.08	ECL deviates  0.006		
5.1958	3159	0.023	----	18.7441		----			
5.2439	3144	0.019	0.995	18.8113	19:1 w8c	0.36	ECL deviates  0.000		
5.3119	26125	0.019	0.994	18.9062	19:0 cyclo w7c	2.98	ECL deviates -0.004		
5.3814	57350	0.018	----	19.0032	19:0	----	ECL deviates  0.003		
5.5334	1735	0.016	----	19.2098		----			
5.5791	689	0.013	----	19.2719		----			
5.6133	1131	0.016	0.988	19.3185	19:0 cyclo 9,10 DMA	0.13	ECL deviates -0.005		
5.6490	1805	0.017	----	19.3669		----			
5.6712	1378	0.014	0.987	19.3971	20:4 w6c	0.16	ECL deviates -0.006		
5.7967	1201	0.023	0.984	19.5677	20:3 w6c	0.14	ECL deviates  0.001		
5.8237	1600	0.019	----	19.6044		----			
5.9016	846	0.012	----	19.7102		----			
5.9244	1135	0.012	0.982	19.7413	20:2 w6c	0.13	ECL deviates  0.001		
5.9449	3750	0.019	0.982	19.7691	20:1 w9c	0.42	ECL deviates -0.004		
5.9731	2363	0.022	0.981	19.8074	20:1 w8c	0.27	ECL deviates -0.006		
6.1166	3813	0.021	0.979	20.0025	20:0	0.43	ECL deviates  0.002	Reference -0.004	
6.2239	656	0.016	----	20.1481		----			
6.2567	1104	0.014	----	20.1926		----			
6.3728	2508	0.013	----	20.3502		----			
6.4009	19038	0.019	0.975	20.3883	20:0 10-methyl	2.13	ECL deviates -0.009		
6.5699	2804	0.022	----	20.6177		----			
6.6476	3149	0.023	----	20.7231		----			
6.7046	3045	0.018	0.972	20.8004	21:1 w8c	0.34	ECL deviates  0.002		
6.8207	4562	0.017	0.971	20.9580	21:1 w3c	0.51	ECL deviates  0.004		
6.8712	1605	0.023	----	21.0266		----			
6.9372	610	0.015	----	21.1165		----			
7.0627	774	0.017	----	21.2872		----			
7.3406	4798	0.040	----	21.6655		----	> max ar/ht		
7.3632	2454	0.020	----	21.6962		----			
7.4573	7141	0.029	----	21.8243		----			
7.5417	1898	0.018	0.969	21.9391	22:1 w3c	0.21	ECL deviates -0.008		
7.5891	4568	0.018	0.970	22.0036	22:0	0.51	ECL deviates  0.004	Reference -0.004	
7.7785	110414	0.018	----	22.2650		----			
8.0858	2839	0.018	----	22.6892		----			
8.1506	956	0.017	----	22.7786		----			
8.2574	1950	0.016	0.978	22.9259	23:1 w4c	0.22	ECL deviates -0.001		
8.3110	649	0.015	0.979	23.0000	23:0	0.07	ECL deviates  0.000	Reference -0.007	
8.5244	912	0.017	----	23.2994		----			
8.7941	4409	0.026	----	23.6778		----			
8.8344	2929	0.021	----	23.7344		----			
8.9385	2071	0.018	----	23.8805		----			
9.0218	3959	0.017	1.001	23.9973	24:0	0.45	ECL deviates -0.003	Reference -0.010	
9.3840	7306	0.019	----	24.5055		----	> max rt		
9.4896	1847	0.017	----	24.6537		----	> max rt		

ECL Deviation: 0.004                            Reference ECL Shift: 0.006       Number Reference Peaks: 18
Total Response: 1030395                       Total Named: 852694
Percent Named: 82.75%                         Total Amount: 871340

(No search libraries specified in method PLFAD1.)
