# Supplementary material for: Microbial regulation of soil carbon properties under nitrogen addition and plant inputs removal
Source: PeerJ. 2019 Jul 17;7:e7343. doi: 10.7717/peerj.7343 (PMC6642627; doi:10.7717/peerj.7343)
Supplement: File S1 — The raw data showed the soil microbial PLFAs files in the year of 2015 and 2016. Each file of rtf. represented the microbial PLFAs for each soil sample. In the Supplemental File, the Excel file named “Numbers” showed the plots names and the related rtf. file names. [file peerj-07-7343-s002.zip › supplementary files/2016/65.rtf]

Volume: DATA            File: E17C203.64A       Samp Ctr: 20                 ID Number: 5038 
Type: Samp                   Bottle: 6                        Method: PLFAD1 
Created: 12/20/2017 5:26:20 PM 
Sample ID: 65 


RT	Response	Ar/Ht	RFact	ECL	Peak Name	Percent	Comment1	Comment2	
0.7652	1.762E+9	0.016	----	7.6951	SOLVENT PEAK	----	< min rt		
0.9525	1029	0.012	----	8.7623		----	< min rt		
1.5886	2362	0.021	0.989	12.0096	12:0	0.24	ECL deviates  0.010	Reference  0.017	
1.6495	747	0.016	----	12.2040		----			
1.7750	727	0.014	1.009	12.6054	13:0 iso	0.08	ECL deviates -0.007	Reference -0.001	
1.8093	777	0.013	1.012	12.7149	13:0 anteiso	0.08	ECL deviates  0.005	Reference  0.011	
1.9909	1394	0.017	----	13.2315		----			
2.1403	8665	0.017	1.030	13.6079	14:0 iso	0.92	ECL deviates -0.006	Reference -0.003	
2.1849	645	0.013	1.032	13.7201	14:0 anteiso	0.07	ECL deviates  0.004	Reference  0.008	
2.2673	1306	0.016	----	13.9277		----			
2.2951	8399	0.015	1.035	13.9975	14:0	0.89	ECL deviates -0.003	Reference  0.000	
2.3579	1651	0.015	----	14.1273	14:0 iso 3OH	----	ECL deviates  0.002		
2.4568	905	0.013	----	14.3315		----			
2.5083	9624	0.018	1.038	14.4378	15:1 iso w6c	1.02	ECL deviates -0.001		
2.5293	1412	0.011	1.038	14.4812	15:4 w3c	0.15	ECL deviates -0.009		
2.5527	1549	0.013	1.038	14.5295	15:1 anteiso w9c	0.16	ECL deviates  0.000		
2.5937	46504	0.015	1.038	14.6143	15:0 iso	4.95	ECL deviates -0.003	Reference -0.001	
2.6394	32842	0.016	1.039	14.7086	15:0 anteiso	3.50	ECL deviates -0.002	Reference -0.001	
2.7097	1123	0.017	1.039	14.8537	15:1 w6c	0.12	ECL deviates -0.006		
2.7799	5448	0.014	1.039	14.9987	15:0	0.58	ECL deviates -0.001	Reference  0.000	
2.8101	1968	0.016	----	15.0519		----			
2.9127	823	0.014	----	15.2330		----			
2.9757	542	0.012	----	15.3443		----			
3.0060	939	0.013	1.038	15.3978	16:1 w7c alcohol	0.10	ECL deviates  0.001		
3.0317	6763	0.020	1.037	15.4432	15:0 DMA	0.72	ECL deviates -0.007		
3.1021	17370	0.016	1.037	15.5675	16:3 w6c	1.85	ECL deviates -0.008		
3.1312	22076	0.016	1.036	15.6188	16:0 iso	2.35	ECL deviates -0.001	Reference -0.001	
3.1868	2517	0.014	1.036	15.7169	16:0 anteiso	0.27	ECL deviates  0.002	Reference  0.002	
3.2167	8813	0.017	1.035	15.7697	16:1 w9c	0.94	ECL deviates -0.005		
3.2455	65692	0.017	1.035	15.8205	16:1 w7c	6.97	ECL deviates -0.004		
3.2972	19221	0.017	1.034	15.9117	16:1 w5c	2.04	ECL deviates  0.001		
3.3462	96342	0.016	1.034	15.9982	16:0	10.21	ECL deviates -0.002	Reference -0.002	
3.3768	4956	0.017	----	16.0468		----			
3.4363	953	0.016	1.032	16.1409	16:2 DMA	0.10	ECL deviates  0.003		
3.4731	760	0.017	----	16.1991		----			
3.6159	44431	0.020	1.030	16.4249	16:0 10-methyl	4.69	ECL deviates  0.005		
3.6615	128211	0.017	1.029	16.4969	17:1 iso w9c	13.52	ECL deviates -0.001		
3.7413	12913	0.017	1.027	16.6231	17:0 iso	1.36	ECL deviates -0.001	Reference -0.002	
3.8016	15033	0.017	1.026	16.7184	17:0 anteiso	1.58	ECL deviates -0.002		
3.8508	6299	0.017	1.025	16.7962	17:1 w8c	0.66	ECL deviates -0.001		
3.9136	27199	0.018	1.024	16.8954	17:0 cyclo w7c	2.86	ECL deviates  0.002		
3.9801	4714	0.018	1.022	17.0005	17:0	0.49	ECL deviates  0.001	Reference -0.001	
4.0074	6267	0.016	1.022	17.0404	17:1 w7c 10-methyl	0.66	ECL deviates -0.003		
4.0535	1471	0.015	----	17.1078		----			
4.1418	1805	0.022	1.019	17.2366	16:0 2OH	0.19	ECL deviates -0.004		
4.2574	7045	0.017	1.017	17.4054	17:0 10-methyl	0.73	ECL deviates -0.002		
4.3190	3124	0.028	----	17.4952		----			
4.3767	3959	0.017	1.014	17.5794	18:3 w6c	0.41	ECL deviates -0.001		
4.4045	3873	0.018	1.013	17.6201	18:0 iso	0.40	ECL deviates -0.007	Reference -0.009	
4.4334	1542	0.017	----	17.6622		----			
4.4772	23896	0.016	1.012	17.7261	18:2 w6c	2.48	ECL deviates -0.001		
4.5091	50218	0.019	1.011	17.7727	18:1 w9c	5.21	ECL deviates -0.002		
4.5462	78810	0.018	1.010	17.8269	18:1 w7c	8.17	ECL deviates  0.000		
4.6048	11823	0.023	----	17.9124		----			
4.6658	17945	0.019	1.008	18.0014	18:0	1.85	ECL deviates  0.001	Reference -0.001	
4.7232	7772	0.019	1.006	18.0816	18:1 w7c 10-methyl	0.80	ECL deviates -0.003		
4.7828	2691	0.025	1.005	18.1648	18:2 DMA	0.28	ECL deviates  0.005		
4.8328	3787	0.036	1.004	18.2346	18:1 w9c DMA	----	> max ar/ht		
4.9433	28493	0.021	1.002	18.3888	18:0 10-methyl	2.93	ECL deviates -0.006		
5.0159	984	0.017	1.000	18.4902	19:4 w6c	0.10	ECL deviates  0.005		
5.0602	4816	0.021	0.999	18.5520	19:3 w6c	0.49	ECL deviates -0.008		
5.1157	577	0.013	0.998	18.6295	19:0 iso	0.06	ECL deviates -0.001		
5.1406	1058	0.017	0.998	18.6643	19:3 w3c	0.11	ECL deviates  0.006		
5.1989	3883	0.025	----	18.7457		----			
5.2453	4173	0.017	0.995	18.8104	19:1 w8c	0.43	ECL deviates -0.001		
5.2786	5343	0.018	0.995	18.8570	19:1 w6c	0.54	ECL deviates  0.005		
5.3121	24409	0.018	0.994	18.9036	19:0 cyclo w7c	2.49	ECL deviates -0.006		
5.3828	67225	0.018	----	19.0024	19:0	----	ECL deviates  0.002		
5.4479	993	0.017	----	19.0908		----			
5.5377	1346	0.019	----	19.2129		----			
5.5794	1965	0.016	----	19.2696		----			
5.6149	560	0.011	0.988	19.3178	19:0 cyclo 9,10 DMA	0.06	ECL deviates -0.006		
5.6494	3249	0.019	----	19.3647		----			
5.6744	2105	0.017	0.987	19.3986	20:4 w6c	0.21	ECL deviates -0.005		
5.7292	617	0.016	0.986	19.4731	20:5 w3c	0.06	ECL deviates -0.009		
5.8004	952	0.020	0.984	19.5699	20:3 w6c	0.10	ECL deviates  0.004		
5.8244	1596	0.016	----	19.6026		----			
5.9012	1194	0.016	----	19.7069		----			
5.9471	4273	0.022	0.982	19.7693	20:1 w9c	0.43	ECL deviates -0.003		
5.9750	2676	0.022	0.981	19.8073	20:1 w8c	0.27	ECL deviates -0.006		
6.1169	6087	0.021	0.979	20.0001	20:0	0.61	ECL deviates  0.000	Reference -0.004	
6.2265	692	0.016	----	20.1488		----			
6.2599	1546	0.017	----	20.1941		----			
6.3725	4484	0.017	----	20.3469		----			
6.4020	28824	0.017	0.975	20.3869	20:0 10-methyl	2.88	ECL deviates -0.010		
6.5104	907	0.021	----	20.5340		----			
6.5693	3635	0.024	----	20.6140		----			
6.6514	3737	0.024	----	20.7253		----			
6.7049	3274	0.016	0.972	20.7979	21:1 w8c	0.33	ECL deviates  0.000		
6.7687	1974	0.021	----	20.8844		----			
6.8201	5782	0.016	0.971	20.9542	21:1 w3c	0.58	ECL deviates  0.000		
6.8676	1910	0.025	----	21.0187		----		Reference  0.015	
6.9373	834	0.017	----	21.1134		----			
7.0624	1562	0.017	----	21.2838		----			
7.3107	3785	0.028	0.969	21.6215	22:0 iso	0.38	ECL deviates  0.004		
7.3408	996	0.013	----	21.6625		----			
7.3644	1737	0.018	----	21.6945		----			
7.4592	6959	0.028	----	21.8236		----			
7.5426	1922	0.017	0.969	21.9370	22:1 w3c	0.19	ECL deviates -0.010		
7.5884	6367	0.018	0.970	21.9993	22:0	0.63	ECL deviates -0.001	Reference -0.004	
7.7809	129189	0.019	----	22.2648		----			
8.0842	2448	0.024	----	22.6833		----			
8.1538	1805	0.020	----	22.7792		----			
8.2590	3102	0.017	0.978	22.9243	23:1 w4c	0.31	ECL deviates -0.002		
8.3119	1653	0.018	0.979	22.9974	23:0	0.17	ECL deviates -0.003	Reference -0.006	
8.5258	2085	0.018	----	23.2972		----			
8.7780	4100	0.032	0.992	23.6509	24:3 w3c	0.42	ECL deviates -0.004		
8.8356	3082	0.024	----	23.7317		----			
8.9424	2977	0.018	----	23.8815		----			
9.0224	5716	0.017	1.001	23.9937	24:0	0.59	ECL deviates -0.006	Reference -0.009	
9.3879	8249	0.019	----	24.5061		----	> max rt		
9.4903	621	0.013	----	24.6496		----	> max rt		

ECL Deviation: 0.005                            Reference ECL Shift: 0.007       Number Reference Peaks: 21
Total Response: 1177420                       Total Named: 955489
Percent Named: 81.15%                         Total Amount: 978975
Profile Comment:   Review report comments.

(No search libraries specified in method PLFAD1.)
