# Supplementary material for: Microbial regulation of soil carbon properties under nitrogen addition and plant inputs removal
Source: PeerJ. 2019 Jul 17;7:e7343. doi: 10.7717/peerj.7343 (PMC6642627; doi:10.7717/peerj.7343)
Supplement: File S1 — The raw data showed the soil microbial PLFAs files in the year of 2015 and 2016. Each file of rtf. represented the microbial PLFAs for each soil sample. In the Supplemental File, the Excel file named “Numbers” showed the plots names and the related rtf. file names. [file peerj-07-7343-s002.zip › supplementary files/2016/66.rtf]

Volume: DATA            File: E17C203.64A       Samp Ctr: 21                 ID Number: 5039 
Type: Samp                   Bottle: 7                        Method: PLFAD1 
Created: 12/20/2017 5:54:54 PM 
Sample ID: 66 


RT	Response	Ar/Ht	RFact	ECL	Peak Name	Percent	Comment1	Comment2	
0.7650	1.728E+9	0.016	----	7.6952	SOLVENT PEAK	----	< min rt		
0.8788	816	0.017	----	8.3436		----	< min rt		
0.9525	1569	0.012	----	8.7631		----	< min rt		
1.2966	566	0.012	0.924	10.7204	11:0 anteiso	0.05	ECL deviates  0.015		
1.5865	1288	0.019	0.989	12.0055	12:0	0.11	ECL deviates  0.006	Reference  0.010	
1.6484	613	0.013	----	12.2030		----			
1.7733	882	0.014	1.009	12.6020	13:0 iso	0.08	ECL deviates -0.010	Reference -0.007	
1.8101	812	0.014	1.012	12.7196	13:0 anteiso	0.07	ECL deviates  0.010	Reference  0.014	
1.8963	1914	0.029	1.019	12.9947	13:0	0.17	ECL deviates -0.005	Reference -0.002	
1.9912	1516	0.017	----	13.2339		----			
2.1405	9679	0.017	1.030	13.6096	14:0 iso	0.86	ECL deviates -0.004	Reference -0.002	
2.1846	766	0.013	1.032	13.7207	14:0 anteiso	0.07	ECL deviates  0.005	Reference  0.007	
2.2146	1135	0.024	1.033	13.7962	14:1 w8c	0.10	ECL deviates -0.006		
2.2951	13063	0.017	1.035	13.9987	14:0	1.17	ECL deviates -0.001	Reference  0.000	
2.3584	1682	0.014	----	14.1292	14:0 iso 3OH	----	ECL deviates  0.004		
2.4554	1296	0.016	----	14.3294		----			
2.5081	11713	0.018	1.038	14.4383	15:1 iso w6c	1.05	ECL deviates -0.001		
2.5297	1701	0.011	1.038	14.4829	15:4 w3c	0.15	ECL deviates -0.007		
2.5528	1900	0.013	1.038	14.5306	15:1 anteiso w9c	0.17	ECL deviates  0.001		
2.5933	59559	0.015	1.038	14.6140	15:0 iso	5.35	ECL deviates -0.003	Reference -0.002	
2.6400	38680	0.015	1.039	14.7106	15:0 anteiso	3.47	ECL deviates  0.000	Reference  0.000	
2.7103	995	0.017	1.039	14.8557	15:1 w6c	0.09	ECL deviates -0.004		
2.7807	6658	0.016	1.039	15.0006	15:0	0.60	ECL deviates  0.001	Reference  0.001	
2.8114	2469	0.016	----	15.0549		----			
2.9139	1267	0.014	----	15.2357		----			
2.9766	513	0.011	----	15.3464		----			
3.0074	960	0.010	1.038	15.4007	16:1 w7c alcohol	0.09	ECL deviates  0.004		
3.0333	7583	0.020	1.037	15.4464	15:0 DMA	0.68	ECL deviates -0.004		
3.1036	17394	0.016	1.037	15.5704	16:3 w6c	1.56	ECL deviates -0.005		
3.1317	25054	0.016	1.036	15.6201	16:0 iso	2.25	ECL deviates  0.000	Reference  0.000	
3.1873	3175	0.016	1.036	15.7180	16:0 anteiso	0.28	ECL deviates  0.003	Reference  0.003	
3.2171	11037	0.018	1.035	15.7706	16:1 w9c	0.99	ECL deviates -0.004		
3.2464	70417	0.017	1.035	15.8224	16:1 w7c	6.30	ECL deviates -0.002		
3.2983	21388	0.017	1.034	15.9139	16:1 w5c	1.91	ECL deviates  0.003		
3.3483	116302	0.016	1.034	16.0019	16:0	10.39	ECL deviates  0.002	Reference  0.001	
3.3769	5546	0.017	----	16.0472		----			
3.4361	1537	0.017	1.032	16.1408	16:2 DMA	0.14	ECL deviates  0.003		
3.4777	1816	0.023	----	16.2065		----			
3.6165	54494	0.019	1.030	16.4258	16:0 10-methyl	4.85	ECL deviates  0.006		
3.6623	128674	0.017	1.029	16.4982	17:1 iso w9c	11.44	ECL deviates  0.000		
3.7423	16789	0.015	1.027	16.6247	17:0 iso	1.49	ECL deviates  0.001	Reference  0.000	
3.8025	20213	0.016	1.026	16.7199	17:0 anteiso	1.79	ECL deviates  0.000		
3.8520	8778	0.020	1.025	16.7981	17:1 w8c	0.78	ECL deviates  0.001		
3.9144	36724	0.018	1.024	16.8967	17:0 cyclo w7c	3.25	ECL deviates  0.003		
3.9809	5939	0.019	1.022	17.0018	17:0	0.52	ECL deviates  0.002	Reference  0.000	
4.0084	7551	0.018	1.022	17.0418	17:1 w7c 10-methyl	0.67	ECL deviates -0.001		
4.0553	1772	0.016	----	17.1104		----			
4.1430	1951	0.021	1.019	17.2383	16:0 2OH	0.17	ECL deviates -0.002		
4.2579	8358	0.018	1.017	17.4060	17:0 10-methyl	0.73	ECL deviates -0.001		
4.2953	895	0.013	1.016	17.4605	17:0 DMA	0.08	ECL deviates  0.002		
4.3214	3378	0.021	----	17.4986		----			
4.3778	3465	0.017	1.014	17.5810	18:3 w6c	0.30	ECL deviates  0.001		
4.4039	4333	0.019	1.013	17.6189	18:0 iso	0.38	ECL deviates -0.008	Reference -0.010	
4.4348	1504	0.015	----	17.6641		----			
4.4776	20406	0.018	1.012	17.7265	18:2 w6c	1.79	ECL deviates -0.001		
4.5106	54858	0.017	1.011	17.7746	18:1 w9c	4.80	ECL deviates  0.000		
4.5467	82806	0.018	1.010	17.8274	18:1 w7c	7.23	ECL deviates  0.000		
4.6056	12784	0.021	1.009	17.9134	18:1 w5c	1.12	ECL deviates -0.010		
4.6662	20937	0.018	1.008	18.0018	18:0	1.82	ECL deviates  0.002	Reference -0.001	
4.7261	8460	0.017	1.006	18.0854	18:1 w7c 10-methyl	0.74	ECL deviates  0.000		
4.7833	3100	0.025	1.005	18.1653	18:2 DMA	0.27	ECL deviates  0.005		
4.8351	2152	0.025	1.004	18.2376	18:1 w9c DMA	0.19	ECL deviates  0.001		
4.9440	35580	0.018	1.002	18.3897	18:0 10-methyl	3.08	ECL deviates -0.005		
5.0189	982	0.020	1.000	18.4943	19:4 w6c	0.08	ECL deviates  0.009		
5.0621	7798	0.018	0.999	18.5545	19:3 w6c	0.67	ECL deviates -0.006		
5.1391	1745	0.022	0.998	18.6620	19:3 w3c	0.15	ECL deviates  0.004		
5.1995	3976	0.026	----	18.7463		----			
5.2470	7095	0.018	0.995	18.8126	19:1 w8c	0.61	ECL deviates  0.002		
5.2822	3656	0.012	0.995	18.8618	19:1 w6c	0.31	ECL deviates  0.010		
5.2865	2273	0.007	0.994	18.8678	19:0 cyclo w9c	0.20	ECL deviates -0.004		
5.3145	30953	0.018	0.994	18.9069	19:0 cyclo w7c	2.66	ECL deviates -0.003		
5.3847	67133	0.017	----	19.0049	19:0	----	ECL deviates  0.005		
5.4509	842	0.015	0.991	19.0949	19:1 w7c 10-methyl	0.07	ECL deviates -0.008		
5.5376	1963	0.016	----	19.2127		----			
5.5803	2073	0.015	----	19.2707		----			
5.6192	2114	0.018	0.988	19.3236	19:0 cyclo 9,10 DMA	0.18	ECL deviates  0.000		
5.6510	3586	0.017	----	19.3668		----			
5.6735	1854	0.015	0.987	19.3974	20:4 w6c	0.16	ECL deviates -0.006		
5.8254	4432	0.025	----	19.6038		----			
5.9013	1115	0.015	----	19.7070		----			
5.9489	4955	0.021	0.982	19.7718	20:1 w9c	0.42	ECL deviates -0.001		
5.9734	3643	0.018	0.981	19.8050	20:1 w8c	0.31	ECL deviates -0.008		
6.1166	6982	0.022	0.979	19.9997	20:0	0.59	ECL deviates  0.000	Reference -0.004	
6.2243	763	0.015	----	20.1458		----			
6.2595	2615	0.016	----	20.1937		----			
6.3726	6393	0.018	----	20.3472		----			
6.4022	35732	0.019	0.975	20.3874	20:0 10-methyl	3.01	ECL deviates -0.010		
6.4678	1581	0.021	----	20.4765		----			
6.5135	3431	0.029	----	20.5384		----			
6.5709	9330	0.023	----	20.6163		----			
6.6495	6443	0.027	----	20.7230		----			
6.7058	7536	0.021	0.972	20.7995	21:1 w8c	0.63	ECL deviates  0.001		
6.7682	4930	0.022	----	20.8841		----			
6.8230	12598	0.019	0.971	20.9585	21:1 w3c	1.06	ECL deviates  0.005		
6.8799	10212	0.029	----	21.0358		----			
6.9402	3357	0.021	----	21.1179		----			
6.9700	3916	0.030	----	21.1585		----			
7.0647	3052	0.021	----	21.2873		----			
7.2051	1475	0.031	0.969	21.4785	22:5 w3c	0.12	ECL deviates  0.011		
7.2803	1452	0.028	----	21.5808		----			
7.3135	4786	0.021	0.969	21.6261	22:0 iso	0.40	ECL deviates  0.008		
7.3433	2443	0.014	----	21.6666		----			
7.3670	3037	0.018	----	21.6988		----			
7.4250	1809	0.022	0.969	21.7778	22:1 w9c	0.15	ECL deviates  0.005		
7.4613	15366	0.021	----	21.8272		----			
7.5438	3699	0.018	0.969	21.9395	22:1 w3c	0.31	ECL deviates -0.007		
7.5906	7955	0.016	0.970	22.0032	22:0	0.67	ECL deviates  0.003	Reference -0.001	
7.6224	929	0.010	----	22.0471		----			
7.6306	1041	0.012	----	22.0583		----			
7.7842	129450	0.019	----	22.2705	Phthalate 2	----	ECL deviates -0.012		
8.0889	3397	0.019	----	22.6910		----			
8.1540	2599	0.021	----	22.7809		----			
8.1959	2947	0.027	----	22.8387		----			
8.2583	5043	0.017	0.978	22.9248	23:1 w4c	0.43	ECL deviates -0.002		
8.3141	2438	0.017	0.979	23.0019	23:0	0.21	ECL deviates  0.002	Reference -0.003	
8.3584	2353	0.022	----	23.0640		----			
8.5263	2389	0.018	----	23.2997		----			
8.7862	9225	0.032	----	23.6644		----			
8.8359	7846	0.026	----	23.7342		----			
8.9448	12129	0.020	----	23.8870		----			
8.9845	1761	0.017	1.000	23.9427	24:1 w3c	0.15	ECL deviates -0.006		
9.0230	9213	0.020	1.001	23.9967	24:0	0.80	ECL deviates -0.003	Reference -0.008	
9.2076	2711	0.040	----	24.2559		----	> max rt		
9.3886	6911	0.020	----	24.5098		----	> max rt		
9.4933	2857	0.022	----	24.6568		----	> max rt		

ECL Deviation: 0.005                            Reference ECL Shift: 0.005       Number Reference Peaks: 21
Total Response: 1296354                       Total Named: 1134346
Percent Named: 87.50%                         Total Amount: 1156608

(No search libraries specified in method PLFAD1.)
