# Supplementary material for: Microbial regulation of soil carbon properties under nitrogen addition and plant inputs removal
Source: PeerJ. 2019 Jul 17;7:e7343. doi: 10.7717/peerj.7343 (PMC6642627; doi:10.7717/peerj.7343)
Supplement: File S1 — The raw data showed the soil microbial PLFAs files in the year of 2015 and 2016. Each file of rtf. represented the microbial PLFAs for each soil sample. In the Supplemental File, the Excel file named “Numbers” showed the plots names and the related rtf. file names. [file peerj-07-7343-s002.zip › supplementary files/2016/67.rtf]

Volume: DATA            File: E17C203.64A       Samp Ctr: 22                 ID Number: 5040 
Type: Samp                   Bottle: 8                        Method: PLFAD1 
Created: 12/20/2017 6:23:33 PM 
Sample ID: 67 


RT	Response	Ar/Ht	RFact	ECL	Peak Name	Percent	Comment1	Comment2	
0.7651	1.693E+9	0.016	----	7.6997	SOLVENT PEAK	----	< min rt		
0.8793	824	0.016	----	8.3497		----	< min rt		
0.9526	2575	0.015	----	8.7676		----	< min rt		
1.2975	575	0.012	----	10.7311		----			
1.3865	776	0.014	0.952	11.1745	10:0 2OH	0.07	ECL deviates -0.009		
1.5904	2405	0.024	----	12.0217		----			
1.6450	897	0.019	----	12.1962		----			
1.7722	651	0.014	1.009	12.6017	13:0 iso	0.06	ECL deviates -0.011	Reference -0.010	
1.8103	592	0.014	1.012	12.7234	13:0 anteiso	0.05	ECL deviates  0.014	Reference  0.014	
1.8867	2090	0.032	1.019	12.9671	13:1 w3c	0.19	ECL deviates -0.003		
1.9914	1540	0.020	----	13.2373		----			
2.1400	5691	0.015	1.030	13.6108	14:0 iso	0.53	ECL deviates -0.003	Reference -0.003	
2.2949	9802	0.016	1.035	14.0004	14:0	0.91	ECL deviates  0.000	Reference  0.000	
2.3576	1477	0.013	----	14.1297	14:0 iso 3OH	----	ECL deviates  0.005		
2.3931	861	0.019	----	14.2029		----			
2.4545	1102	0.016	----	14.3295		----			
2.5079	9506	0.018	1.038	14.4398	15:1 iso w6c	0.88	ECL deviates  0.001		
2.5292	1515	0.012	1.038	14.4836	15:4 w3c	0.14	ECL deviates -0.007		
2.5523	1395	0.014	1.038	14.5313	15:1 anteiso w9c	0.13	ECL deviates  0.001		
2.5930	54156	0.015	1.038	14.6152	15:0 iso	5.04	ECL deviates -0.002	Reference -0.003	
2.6392	32329	0.015	1.039	14.7104	15:0 anteiso	3.01	ECL deviates -0.001	Reference -0.001	
2.7086	1066	0.017	1.039	14.8536	15:1 w6c	0.10	ECL deviates -0.006		
2.7797	6220	0.015	1.039	15.0004	15:0	0.58	ECL deviates  0.000	Reference -0.001	
2.8101	2159	0.015	----	15.0540		----			
2.9122	2084	0.019	----	15.2340		----			
2.9758	717	0.012	----	15.3461		----			
3.0081	683	0.010	1.038	15.4031	16:1 w7c alcohol	0.06	ECL deviates  0.006		
3.0311	7455	0.019	1.037	15.4437	15:0 DMA	0.69	ECL deviates -0.007		
3.1023	15885	0.016	1.037	15.5692	16:3 w6c	1.48	ECL deviates -0.007		
3.1313	24521	0.016	1.036	15.6203	16:0 iso	2.28	ECL deviates  0.001	Reference -0.001	
3.1855	3124	0.015	1.036	15.7158	16:0 anteiso	0.29	ECL deviates  0.001	Reference  0.000	
3.2168	8158	0.018	1.035	15.7712	16:1 w9c	0.76	ECL deviates -0.004		
3.2457	61862	0.018	1.035	15.8220	16:1 w7c	5.74	ECL deviates -0.002		
3.2972	15281	0.017	1.034	15.9129	16:1 w5c	1.42	ECL deviates  0.002		
3.3193	3153	0.010	1.034	15.9518	16:1 w3c	0.29	ECL deviates -0.001		
3.3475	124985	0.015	1.034	16.0016	16:0	11.58	ECL deviates  0.002	Reference  0.000	
3.3778	4365	0.017	----	16.0494		----			
3.4362	2184	0.020	1.032	16.1417	16:2 DMA	0.20	ECL deviates  0.004		
3.4912	1635	0.028	----	16.2286		----			
3.6155	46821	0.018	1.030	16.4248	16:0 10-methyl	4.32	ECL deviates  0.005		
3.6611	121476	0.017	1.029	16.4969	17:1 iso w9c	11.20	ECL deviates -0.001		
3.7424	15484	0.017	1.027	16.6254	17:0 iso	1.43	ECL deviates  0.002	Reference  0.000	
3.8025	20149	0.017	1.026	16.7202	17:0 anteiso	1.85	ECL deviates  0.000		
3.8513	7644	0.019	1.025	16.7974	17:1 w8c	0.70	ECL deviates  0.000		
3.9132	34079	0.019	1.024	16.8952	17:0 cyclo w7c	3.13	ECL deviates  0.002		
3.9804	6627	0.018	1.022	17.0013	17:0	0.61	ECL deviates  0.001	Reference -0.001	
4.0073	6027	0.016	1.022	17.0406	17:1 w7c 10-methyl	0.55	ECL deviates -0.003		
4.0537	1646	0.014	----	17.1082		----			
4.0856	619	0.015	----	17.1549		----			
4.1447	2651	0.021	1.019	17.2410	16:0 2OH	0.24	ECL deviates  0.001		
4.2575	7556	0.016	1.017	17.4055	17:0 10-methyl	0.69	ECL deviates -0.001		
4.2955	560	0.013	1.016	17.4611	17:0 DMA	0.05	ECL deviates  0.003		
4.3196	3424	0.020	----	17.4962		----			
4.3785	4228	0.020	1.014	17.5821	18:3 w6c	0.38	ECL deviates  0.002		
4.4042	5197	0.018	1.013	17.6196	18:0 iso	0.47	ECL deviates -0.007	Reference -0.009	
4.4342	1781	0.017	----	17.6633		----			
4.4772	28355	0.018	1.012	17.7261	18:2 w6c	2.57	ECL deviates -0.001		
4.5099	56471	0.017	1.011	17.7738	18:1 w9c	5.12	ECL deviates -0.001		
4.5465	91367	0.018	1.010	17.8272	18:1 w7c	8.28	ECL deviates  0.000		
4.6046	18782	0.022	----	17.9119		----			
4.6651	24476	0.018	1.008	18.0002	18:0	2.21	ECL deviates  0.000	Reference -0.002	
4.7234	10894	0.018	1.006	18.0816	18:1 w7c 10-methyl	0.98	ECL deviates -0.003		
4.7783	3417	0.025	1.005	18.1583	18:2 DMA	0.31	ECL deviates -0.002		
4.8404	4406	0.037	1.004	18.2449	18:1 w9c DMA	----	> max ar/ht		
4.9444	25340	0.019	1.002	18.3901	18:0 10-methyl	2.28	ECL deviates -0.005		
4.9687	3658	0.013	1.001	18.4241	18:0 DMA	0.33	ECL deviates -0.006		
5.0118	4232	0.022	1.000	18.4843	19:4 w6c	0.38	ECL deviates -0.001		
5.0601	7227	0.021	0.999	18.5516	19:3 w6c	0.65	ECL deviates -0.008		
5.1157	533	0.012	0.998	18.6293	19:0 iso	0.05	ECL deviates -0.001		
5.1417	2100	0.017	0.998	18.6655	19:3 w3c	0.19	ECL deviates  0.007		
5.1967	4229	0.028	----	18.7423		----			
5.2453	5636	0.020	0.995	18.8102	19:1 w8c	0.50	ECL deviates -0.001		
5.2766	11239	0.019	0.995	18.8538	19:1 w6c	1.00	ECL deviates  0.002		
5.3140	32232	0.018	0.994	18.9060	19:0 cyclo w7c	2.87	ECL deviates -0.004		
5.3842	67708	0.018	----	19.0040	19:0	----	ECL deviates  0.004		
5.4504	1872	0.017	0.991	19.0940	19:1 w7c 10-methyl	0.17	ECL deviates -0.009		
5.5377	3415	0.022	----	19.2126		----			
5.5807	7967	0.016	----	19.2711		----			
5.6140	2527	0.017	0.988	19.3164	19:0 cyclo 9,10 DMA	0.22	ECL deviates -0.007		
5.6507	10881	0.025	----	19.3663		----			
5.7031	2124	0.023	----	19.4374		----			
5.7617	1064	0.017	----	19.5170		----			
5.8253	6219	0.029	----	19.6036		----			
5.8652	888	0.018	----	19.6577		----			
5.8999	2046	0.017	----	19.7049		----			
5.9459	6622	0.025	0.982	19.7674	20:1 w9c	0.58	ECL deviates -0.005		
5.9736	3218	0.018	0.981	19.8051	20:1 w8c	0.28	ECL deviates -0.008		
6.1171	8992	0.023	0.979	20.0002	20:0	0.79	ECL deviates  0.000	Reference -0.003	
6.2257	868	0.016	----	20.1476		----			
6.2596	3724	0.017	----	20.1935		----			
6.3416	969	0.016	----	20.3049		----			
6.3734	4678	0.017	----	20.3481		----			
6.4038	29770	0.019	0.975	20.3893	20:0 10-methyl	2.60	ECL deviates -0.008		
6.5092	2420	0.024	----	20.5323		----			
6.5720	5790	0.022	----	20.6176		----			
6.6512	6067	0.027	----	20.7251		----			
6.7052	4313	0.019	0.972	20.7985	21:1 w8c	0.38	ECL deviates  0.000		
6.7673	5198	0.019	----	20.8827		----			
6.8220	4896	0.017	0.971	20.9570	21:1 w3c	0.43	ECL deviates  0.003		
6.8774	7009	0.032	----	21.0323		----			
6.9389	2008	0.018	----	21.1160		----			
6.9713	2545	0.028	----	21.1601		----			
7.0626	6017	0.019	----	21.2844		----			
7.1974	1643	0.031	0.969	21.4679	22:5 w3c	0.14	ECL deviates  0.000		
7.2572	1449	0.026	----	21.5494		----			
7.3151	10984	0.028	0.969	21.6281	22:0 iso	0.95	ECL deviates  0.010		
7.3653	4580	0.019	----	21.6965		----			
7.4247	2204	0.025	0.969	21.7773	22:1 w9c	0.19	ECL deviates  0.004		
7.4619	13639	0.023	----	21.8280		----			
7.5438	2842	0.018	0.969	21.9394	22:1 w3c	0.25	ECL deviates -0.008		
7.5912	13755	0.016	0.970	22.0041	22:0	1.20	ECL deviates  0.004	Reference -0.001	
7.6261	735	0.015	----	22.0522		----			
7.7831	124315	0.018	----	22.2690	Phthalate 2	----	ECL deviates -0.014		
7.9353	946	0.019	0.973	22.4792	23:4 w6c	0.08	ECL deviates  0.008		
8.0882	5150	0.020	----	22.6903		----			
8.1570	3017	0.021	----	22.7852		----			
8.2022	3873	0.029	----	22.8478		----			
8.2602	5248	0.018	0.978	22.9278	23:1 w4c	0.46	ECL deviates  0.001		
8.3134	2993	0.018	0.979	23.0013	23:0	0.26	ECL deviates  0.001	Reference -0.004	
8.3569	2337	0.024	----	23.0623		----			
8.5247	3790	0.018	----	23.2979		----			
8.7857	6514	0.017	----	23.6644		----			
8.7930	6083	0.016	----	23.6746		----			
8.8374	10960	0.027	----	23.7370		----			
8.9013	1003	0.015	----	23.8267		----			
8.9424	8908	0.022	----	23.8844		----			
8.9831	1565	0.017	1.000	23.9415	24:1 w3c	0.14	ECL deviates -0.007		
9.0224	11720	0.021	1.001	23.9968	24:0	1.05	ECL deviates -0.003	Reference -0.009	
9.2078	1969	0.034	----	24.2571		----	> max rt		
9.3902	11012	0.020	----	24.5132		----	> max rt		
9.4890	4411	0.021	----	24.6520		----	> max rt		

ECL Deviation: 0.005                            Reference ECL Shift: 0.005       Number Reference Peaks: 18
Total Response: 1302029                       Total Named: 1094867
Percent Named: 84.09%                         Total Amount: 1119943
Profile Comment:   Review report comments.

(No search libraries specified in method PLFAD1.)
