# Supplementary material for: Microbial regulation of soil carbon properties under nitrogen addition and plant inputs removal
Source: PeerJ. 2019 Jul 17;7:e7343. doi: 10.7717/peerj.7343 (PMC6642627; doi:10.7717/peerj.7343)
Supplement: File S1 — The raw data showed the soil microbial PLFAs files in the year of 2015 and 2016. Each file of rtf. represented the microbial PLFAs for each soil sample. In the Supplemental File, the Excel file named “Numbers” showed the plots names and the related rtf. file names. [file peerj-07-7343-s002.zip › supplementary files/2016/68.rtf]

Volume: DATA            File: E17C203.64A       Samp Ctr: 23                 ID Number: 5041 
Type: Samp                   Bottle: 9                        Method: PLFAD1 
Created: 12/20/2017 6:52:05 PM 
Sample ID: 68 


RT	Response	Ar/Ht	RFact	ECL	Peak Name	Percent	Comment1	Comment2	
0.7654	1.689E+9	0.016	----	7.7002	SOLVENT PEAK	----	< min rt		
0.9531	1717	0.013	----	8.7692		----	< min rt		
1.2987	529	0.012	----	10.7296		----			
1.3857	666	0.014	----	11.1648		----			
1.5886	862	0.017	0.989	12.0122	12:0	0.08	ECL deviates  0.012	Reference  0.017	
1.7730	730	0.014	1.009	12.6021	13:0 iso	0.07	ECL deviates -0.010	Reference -0.008	
1.8100	609	0.013	1.012	12.7205	13:0 anteiso	0.06	ECL deviates  0.011	Reference  0.013	
1.8929	1656	0.031	1.019	12.9857	13:0	0.15	ECL deviates -0.014	Reference -0.013	
1.9907	1443	0.020	----	13.2349		----			
2.1396	8960	0.016	1.030	13.6099	14:0 iso	0.82	ECL deviates -0.004	Reference -0.005	
2.1835	738	0.013	1.032	13.7207	14:0 anteiso	0.07	ECL deviates  0.005	Reference  0.004	
2.2067	1054	0.021	1.032	13.7790	14:1 w9c	0.10	ECL deviates  0.002		
2.2938	11143	0.016	1.035	13.9985	14:0	1.03	ECL deviates -0.002	Reference -0.003	
2.3565	1589	0.015	----	14.1286	14:0 iso 3OH	----	ECL deviates  0.004		
2.3898	828	0.020	----	14.1974		----			
2.4526	949	0.016	----	14.3273		----			
2.5070	14920	0.019	1.038	14.4397	15:1 iso w6c	1.38	ECL deviates  0.001		
2.5511	2167	0.015	1.038	14.5308	15:1 anteiso w9c	0.20	ECL deviates  0.001		
2.5920	56673	0.015	1.038	14.6152	15:0 iso	5.26	ECL deviates -0.002	Reference -0.005	
2.6377	34268	0.015	1.039	14.7096	15:0 anteiso	3.18	ECL deviates -0.001	Reference -0.004	
2.7068	1247	0.019	1.039	14.8525	15:1 w6c	0.12	ECL deviates -0.007		
2.7782	5905	0.016	1.039	14.9999	15:0	0.55	ECL deviates  0.000	Reference -0.004	
2.8087	1688	0.016	----	15.0545		----			
2.9115	2166	0.018	----	15.2360		----			
3.0292	8531	0.021	1.037	15.4438	15:0 DMA	0.79	ECL deviates -0.007		
3.1007	17121	0.016	1.037	15.5700	16:3 w6c	1.59	ECL deviates -0.006		
3.1294	23954	0.016	1.036	15.6207	16:0 iso	2.22	ECL deviates  0.001	Reference -0.004	
3.1838	3031	0.014	1.036	15.7167	16:0 anteiso	0.28	ECL deviates  0.002	Reference -0.003	
3.2148	10253	0.017	1.035	15.7716	16:1 w9c	0.95	ECL deviates -0.003		
3.2435	70208	0.018	1.035	15.8222	16:1 w7c	6.49	ECL deviates -0.002		
3.2952	19766	0.016	1.034	15.9135	16:1 w5c	1.83	ECL deviates  0.002		
3.3156	5091	0.011	1.034	15.9494	16:1 w3c	0.47	ECL deviates -0.003		
3.3457	131871	0.015	1.034	16.0027	16:0	12.17	ECL deviates  0.003	Reference -0.003	
3.3746	3222	0.016	----	16.0487		----			
3.3977	1320	0.014	----	16.0851		----			
3.4334	1368	0.015	1.032	16.1416	16:2 DMA	0.13	ECL deviates  0.004		
3.4701	1376	0.021	----	16.1997		----			
3.4855	499	0.010	----	16.2240		----			
3.6122	48765	0.019	1.030	16.4244	16:0 10-methyl	4.48	ECL deviates  0.004		
3.6587	120092	0.017	1.029	16.4980	17:1 iso w9c	11.03	ECL deviates  0.000		
3.7395	15888	0.017	1.027	16.6257	17:0 iso	1.46	ECL deviates  0.002	Reference -0.005	
3.7993	18725	0.018	1.026	16.7202	17:0 anteiso	1.72	ECL deviates  0.000		
3.8481	8161	0.020	1.025	16.7973	17:1 w8c	0.75	ECL deviates  0.000		
3.9112	33377	0.017	1.024	16.8971	17:0 cyclo w7c	3.05	ECL deviates  0.003		
3.9777	6732	0.019	1.022	17.0023	17:0	0.61	ECL deviates  0.002	Reference -0.005	
4.0046	5851	0.017	1.022	17.0419	17:1 w7c 10-methyl	0.53	ECL deviates -0.001		
4.0513	1561	0.016	----	17.1101		----			
4.0850	706	0.016	----	17.1592		----			
4.1395	2287	0.022	1.019	17.2388	16:0 2OH	0.21	ECL deviates -0.001		
4.2547	8480	0.018	1.017	17.4070	17:0 10-methyl	0.77	ECL deviates  0.000		
4.3177	3197	0.026	----	17.4990		----			
4.3748	4022	0.018	1.014	17.5823	18:3 w6c	0.36	ECL deviates  0.002		
4.3978	4735	0.018	1.013	17.6158	18:0 iso	0.43	ECL deviates -0.011	Reference -0.019	
4.4310	1637	0.016	----	17.6643		----			
4.4742	28121	0.017	1.012	17.7273	18:2 w6c	2.54	ECL deviates  0.000		
4.5068	56397	0.018	1.011	17.7750	18:1 w9c	5.09	ECL deviates  0.000		
4.5432	92317	0.018	1.010	17.8280	18:1 w7c	8.33	ECL deviates  0.001		
4.6011	22093	0.019	----	17.9125		----			
4.6625	22310	0.017	1.008	18.0022	18:0	2.01	ECL deviates  0.002	Reference -0.006	
4.7215	9671	0.018	1.006	18.0847	18:1 w7c 10-methyl	0.87	ECL deviates  0.000		
4.7782	2437	0.020	1.005	18.1638	18:2 DMA	0.22	ECL deviates  0.004		
4.8182	4428	0.030	----	18.2197		----			
4.9418	26836	0.019	1.002	18.3923	18:0 10-methyl	2.40	ECL deviates -0.003		
4.9667	3035	0.012	1.001	18.4270	18:0 DMA	0.27	ECL deviates -0.003		
5.0123	789	0.018	1.000	18.4907	19:4 w6c	0.07	ECL deviates  0.006		
5.0585	6279	0.019	0.999	18.5552	19:3 w6c	0.56	ECL deviates -0.005		
5.1388	1626	0.020	0.998	18.6673	19:3 w3c	0.14	ECL deviates  0.009		
5.1976	3424	0.025	----	18.7494		----			
5.2445	4487	0.017	0.995	18.8148	19:1 w8c	0.40	ECL deviates  0.004		
5.2819	5558	0.019	0.994	18.8670	19:0 cyclo w9c	0.49	ECL deviates -0.005		
5.3114	24155	0.016	0.994	18.9082	19:0 cyclo w7c	2.14	ECL deviates -0.002		
5.3814	65976	0.018	----	19.0060	19:0	----	ECL deviates  0.006		
5.4471	698	0.012	0.991	19.0952	19:1 w7c 10-methyl	0.06	ECL deviates -0.008		
5.5351	2506	0.018	----	19.2148		----			
5.5764	2163	0.014	----	19.2710		----			
5.6138	1638	0.014	0.988	19.3218	19:0 cyclo 9,10 DMA	0.14	ECL deviates -0.002		
5.6488	8345	0.023	----	19.3693		----			
5.7625	762	0.015	----	19.5238		----			
5.8210	5048	0.031	----	19.6034		----			
5.8984	1119	0.014	----	19.7085		----			
5.9441	6456	0.023	0.982	19.7705	20:1 w9c	0.57	ECL deviates -0.002		
5.9712	2440	0.028	0.981	19.8074	20:1 w8c	0.21	ECL deviates -0.006		
6.1157	7628	0.024	0.979	20.0036	20:0	0.67	ECL deviates  0.004	Reference -0.005	
6.1760	784	0.018	----	20.0854		----			
6.2229	982	0.016	----	20.1491		----			
6.2587	2188	0.017	----	20.1976		----			
6.3352	1592	0.022	----	20.3014		----			
6.3694	5193	0.016	----	20.3478		----			
6.4003	33913	0.021	0.975	20.3897	20:0 10-methyl	2.95	ECL deviates -0.007		
6.4652	1555	0.019	----	20.4777		----			
6.5077	3394	0.029	----	20.5353		----			
6.5687	6785	0.024	----	20.6180		----			
6.6481	5197	0.025	----	20.7258		----			
6.7048	3336	0.018	0.972	20.8027	21:1 w8c	0.29	ECL deviates  0.005		
6.7665	3184	0.019	----	20.8864		----			
6.8200	4401	0.018	0.971	20.9589	21:1 w3c	0.38	ECL deviates  0.005		
6.8789	5070	0.031	----	21.0388		----			
6.9429	1062	0.019	----	21.1259		----			
6.9683	1367	0.019	----	21.1605		----			
7.0605	2318	0.017	----	21.2858		----			
7.1962	1584	0.027	0.969	21.4704	22:5 w3c	0.14	ECL deviates  0.003		
7.2578	1171	0.024	----	21.5541		----			
7.3129	4660	0.019	0.969	21.6290	22:0 iso	0.40	ECL deviates  0.011		
7.3436	2799	0.016	----	21.6707		----			
7.3614	3833	0.020	----	21.6949		----			
7.4219	2591	0.024	0.969	21.7772	22:1 w9c	0.22	ECL deviates  0.004		
7.4602	14652	0.026	----	21.8293		----			
7.5438	2925	0.020	0.969	21.9429	22:1 w3c	0.25	ECL deviates -0.004		
7.5894	8816	0.019	0.970	22.0049	22:0	0.76	ECL deviates  0.005	Reference -0.003	
7.6275	2709	0.031	----	22.0574		----			
7.6949	1725	0.033	----	22.1503		----			
7.7794	125943	0.017	----	22.2669		----			
7.8403	1825	0.027	----	22.3508		----			
7.9399	1130	0.022	0.973	22.4880	23:4 w6c	0.10	ECL deviates  0.017		
7.9861	1390	0.022	----	22.5518		----			
8.0468	1294	0.028	0.974	22.6354	23:3 w3c	0.11	ECL deviates -0.009		
8.0838	3629	0.021	----	22.6864		----			
8.1531	2384	0.021	----	22.7820		----			
8.1972	3941	0.030	----	22.8428		----			
8.2576	3916	0.017	0.978	22.9260	23:1 w4c	0.34	ECL deviates  0.000		
8.3120	2885	0.018	0.979	23.0009	23:0	0.25	ECL deviates  0.001	Reference -0.006	
8.3515	1927	0.025	----	23.0563		----			
8.5245	1937	0.019	----	23.2986		----			
8.7818	4549	0.018	0.992	23.6592	24:3 w3c	0.40	ECL deviates  0.005		
8.7873	4643	0.018	----	23.6669		----			
8.8365	9376	0.029	----	23.7358		----			
8.9420	8523	0.022	----	23.8836		----			
8.9835	1314	0.017	1.000	23.9418	24:1 w3c	0.12	ECL deviates -0.007		
9.0216	7939	0.019	1.001	23.9950	24:0	0.71	ECL deviates -0.005	Reference -0.010	
9.2087	1103	0.024	----	24.2572		----	> max rt		
9.3875	17703	0.020	----	24.5076		----	> max rt		
9.4899	2482	0.018	----	24.6510		----	> max rt		
9.5392	1052	0.019	----	24.7201		----	> max rt		

ECL Deviation: 0.005                            Reference ECL Shift: 0.008       Number Reference Peaks: 21
Total Response: 1402124                       Total Named: 1097369
Percent Named: 78.26%                         Total Amount: 1119754

(No search libraries specified in method PLFAD1.)
