# Supplementary material for: Microbial regulation of soil carbon properties under nitrogen addition and plant inputs removal
Source: PeerJ. 2019 Jul 17;7:e7343. doi: 10.7717/peerj.7343 (PMC6642627; doi:10.7717/peerj.7343)
Supplement: File S1 — The raw data showed the soil microbial PLFAs files in the year of 2015 and 2016. Each file of rtf. represented the microbial PLFAs for each soil sample. In the Supplemental File, the Excel file named “Numbers” showed the plots names and the related rtf. file names. [file peerj-07-7343-s002.zip › supplementary files/2016/69.rtf]

Volume: DATA            File: E17C203.64A       Samp Ctr: 24                 ID Number: 5042 
Type: Samp                   Bottle: 10                      Method: PLFAD1 
Created: 12/20/2017 7:20:27 PM 
Sample ID: 69 


RT	Response	Ar/Ht	RFact	ECL	Peak Name	Percent	Comment1	Comment2	
0.7652	1.704E+9	0.015	----	7.6966	SOLVENT PEAK	----	< min rt		
0.8813	1336	0.016	----	8.3582		----	< min rt		
0.9513	1171	0.009	----	8.7568		----	< min rt		
1.2973	531	0.011	0.924	10.7210	11:0 anteiso	0.05	ECL deviates  0.016		
1.3858	497	0.013	----	11.1638		----			
1.5871	2524	0.025	0.989	12.0058	12:0	0.24	ECL deviates  0.006	Reference  0.012	
1.6476	633	0.012	----	12.1992		----			
1.7735	978	0.014	1.009	12.6019	13:0 iso	0.10	ECL deviates -0.010	Reference -0.006	
1.8120	985	0.015	1.012	12.7250	13:0 anteiso	0.10	ECL deviates  0.016	Reference  0.020	
1.8950	1652	0.028	1.019	12.9902	13:0	0.16	ECL deviates -0.010	Reference -0.006	
1.9898	1441	0.017	----	13.2304		----			
2.1402	9688	0.016	1.030	13.6093	14:0 iso	0.97	ECL deviates -0.005	Reference -0.003	
2.1853	1004	0.015	1.032	13.7228	14:0 anteiso	0.10	ECL deviates  0.007	Reference  0.008	
2.2129	1102	0.020	1.033	13.7924	14:1 w8c	0.11	ECL deviates -0.009		
2.2945	11744	0.016	1.035	13.9980	14:0	1.18	ECL deviates -0.002	Reference -0.001	
2.3568	1617	0.014	----	14.1269	14:0 iso 3OH	----	ECL deviates  0.002		
2.3925	1106	0.025	----	14.2006		----			
2.4559	1304	0.017	----	14.3316		----			
2.5080	10856	0.017	1.038	14.4392	15:1 iso w6c	1.09	ECL deviates  0.000		
2.5297	1476	0.011	1.038	14.4842	15:4 w3c	0.15	ECL deviates -0.006		
2.5528	1927	0.015	1.038	14.5317	15:1 anteiso w9c	0.19	ECL deviates  0.002		
2.5929	50097	0.014	1.038	14.6146	15:0 iso	5.04	ECL deviates -0.002	Reference -0.003	
2.6387	32786	0.015	1.039	14.7093	15:0 anteiso	3.30	ECL deviates -0.002	Reference -0.002	
2.7085	1897	0.024	1.039	14.8535	15:1 w6c	0.19	ECL deviates -0.007		
2.7791	6300	0.016	1.039	14.9991	15:0	0.63	ECL deviates -0.001	Reference -0.002	
2.8105	2154	0.016	----	15.0550		----			
2.9125	1643	0.018	----	15.2350		----			
3.0314	7312	0.020	1.037	15.4450	15:0 DMA	0.73	ECL deviates -0.006		
3.1013	16760	0.015	1.037	15.5684	16:3 w6c	1.68	ECL deviates -0.007		
3.1305	21427	0.016	1.036	15.6200	16:0 iso	2.15	ECL deviates  0.000	Reference -0.002	
3.1853	2934	0.016	1.036	15.7166	16:0 anteiso	0.29	ECL deviates  0.002	Reference -0.001	
3.2160	8444	0.016	1.035	15.7709	16:1 w9c	0.85	ECL deviates -0.004		
3.2451	52934	0.016	1.035	15.8222	16:1 w7c	5.31	ECL deviates -0.002		
3.2647	5595	0.011	1.035	15.8569	16:1 w6c	0.56	ECL deviates -0.007		
3.2970	16812	0.015	1.034	15.9139	16:1 w5c	1.68	ECL deviates  0.003		
3.3183	2139	0.009	1.034	15.9515	16:1 w3c	0.21	ECL deviates -0.001		
3.3462	110892	0.015	1.034	16.0007	16:0	11.10	ECL deviates  0.001	Reference -0.002	
3.3758	4102	0.017	----	16.0477		----			
3.4349	1579	0.016	1.032	16.1412	16:2 DMA	0.16	ECL deviates  0.003		
3.4744	1684	0.029	----	16.2036		----			
3.6139	42170	0.019	1.030	16.4241	16:0 10-methyl	4.21	ECL deviates  0.004		
3.6598	105027	0.017	1.029	16.4968	17:1 iso w9c	10.46	ECL deviates -0.001		
3.7409	13447	0.016	1.027	16.6250	17:0 iso	1.34	ECL deviates  0.001	Reference -0.002	
3.8011	16870	0.016	1.026	16.7202	17:0 anteiso	1.68	ECL deviates  0.000		
3.8495	5727	0.019	1.025	16.7967	17:1 w8c	0.57	ECL deviates  0.000		
3.9127	30626	0.018	1.024	16.8967	17:0 cyclo w7c	3.04	ECL deviates  0.003		
3.9785	5612	0.017	1.022	17.0007	17:0	0.56	ECL deviates  0.001	Reference -0.004	
4.0059	5179	0.017	1.022	17.0409	17:1 w7c 10-methyl	0.51	ECL deviates -0.002		
4.0518	1360	0.015	----	17.1078		----			
4.1410	2321	0.021	1.019	17.2381	16:0 2OH	0.23	ECL deviates -0.002		
4.2570	7145	0.017	1.017	17.4074	17:0 10-methyl	0.70	ECL deviates  0.000		
4.3189	3922	0.024	----	17.4978		----			
4.3759	4022	0.017	1.014	17.5810	18:3 w6c	0.40	ECL deviates  0.001		
4.4032	4293	0.020	1.013	17.6207	18:0 iso	0.42	ECL deviates -0.006	Reference -0.011	
4.4317	1638	0.017	----	17.6623		----			
4.4763	21726	0.017	1.012	17.7274	18:2 w6c	2.13	ECL deviates  0.000		
4.5088	48027	0.017	1.011	17.7749	18:1 w9c	4.70	ECL deviates  0.000		
4.5452	77568	0.018	1.010	17.8281	18:1 w7c	7.59	ECL deviates  0.001		
4.6036	16775	0.022	1.009	17.9132	18:1 w5c	1.64	ECL deviates -0.010		
4.6642	19985	0.019	1.008	18.0017	18:0	1.95	ECL deviates  0.002	Reference -0.004	
4.7247	8459	0.018	1.006	18.0862	18:1 w7c 10-methyl	0.82	ECL deviates  0.001		
4.7809	2806	0.024	1.005	18.1648	18:2 DMA	0.27	ECL deviates  0.005		
4.8264	4068	0.042	1.004	18.2283	18:1 w9c DMA	----	> max ar/ht		
4.8932	758	0.017	----	18.3216		----			
4.9437	26363	0.017	1.002	18.3921	18:0 10-methyl	2.56	ECL deviates -0.003		
4.9688	3044	0.012	1.001	18.4271	18:0 DMA	0.30	ECL deviates -0.003		
5.0170	1229	0.020	1.000	18.4944	19:4 w6c	0.12	ECL deviates  0.009		
5.0619	5920	0.020	0.999	18.5570	19:3 w6c	0.57	ECL deviates -0.003		
5.1401	1107	0.016	0.998	18.6662	19:3 w3c	0.11	ECL deviates  0.008		
5.1994	3129	0.027	----	18.7490		----			
5.2458	4037	0.019	0.995	18.8138	19:1 w8c	0.39	ECL deviates  0.003		
5.2781	7175	0.020	0.995	18.8589	19:1 w6c	0.69	ECL deviates  0.007		
5.3122	23628	0.017	0.994	18.9064	19:0 cyclo w7c	2.27	ECL deviates -0.003		
5.3827	68819	0.017	----	19.0049	19:0	----	ECL deviates  0.005		
5.4480	1913	0.020	0.991	19.0937	19:1 w7c 10-methyl	0.18	ECL deviates -0.009		
5.5358	3390	0.018	----	19.2129		----			
5.5782	4675	0.018	----	19.2705		----			
5.6166	2683	0.017	0.988	19.3228	19:0 cyclo 9,10 DMA	0.26	ECL deviates -0.001		
5.6507	9361	0.028	----	19.3691		----			
5.7281	747	0.015	0.986	19.4743	20:5 w3c	0.07	ECL deviates -0.008		
5.7609	864	0.017	----	19.5188		----			
5.8244	5261	0.026	----	19.6052		----			
5.8621	667	0.015	----	19.6564		----			
5.9470	6822	0.029	0.982	19.7718	20:1 w9c	0.65	ECL deviates -0.001		
5.9720	2978	0.022	0.981	19.8057	20:1 w8c	0.28	ECL deviates -0.007		
6.1176	6947	0.023	0.979	20.0036	20:0	0.66	ECL deviates  0.004	Reference -0.003	
6.2265	780	0.017	----	20.1513		----			
6.2599	2585	0.017	----	20.1967		----			
6.3401	1037	0.019	----	20.3055		----			
6.3729	5217	0.015	----	20.3499		----			
6.4011	32794	0.021	0.975	20.3882	20:0 10-methyl	3.10	ECL deviates -0.009		
6.4655	1657	0.017	----	20.4755		----			
6.5065	3501	0.026	----	20.5312		----			
6.5683	6240	0.023	----	20.6150		----			
6.6514	5482	0.025	----	20.7277		----			
6.7040	5306	0.019	0.972	20.7991	21:1 w8c	0.50	ECL deviates  0.001		
6.7651	5149	0.020	----	20.8819		----			
6.8197	5068	0.020	0.971	20.9560	21:1 w3c	0.48	ECL deviates  0.002		
6.8757	10019	0.030	----	21.0321		----			
6.9386	2241	0.019	----	21.1177		----			
6.9681	3150	0.028	----	21.1578		----			
7.0608	4413	0.019	----	21.2838		----			
7.1860	1783	0.029	0.969	21.4541	22:5 w3c	0.17	ECL deviates -0.014		
7.3141	7422	0.025	0.969	21.6283	22:0 iso	0.70	ECL deviates  0.011		
7.3385	6621	0.028	----	21.6615		----			
7.4205	2439	0.023	0.969	21.7731	22:1 w9c	0.23	ECL deviates  0.000		
7.4601	15652	0.023	----	21.8269		----			
7.5396	2677	0.018	0.969	21.9350	22:1 w3c	0.25	ECL deviates -0.012		
7.5888	10007	0.018	0.970	22.0019	22:0	0.94	ECL deviates  0.002	Reference -0.004	
7.6241	1282	0.019	----	22.0505		----			
7.7810	113121	0.018	----	22.2670		----			
7.9327	1747	0.024	0.973	22.4761	23:4 w6c	0.16	ECL deviates  0.005		
8.0182	1591	0.032	0.974	22.5941	23:3 w6c	0.15	ECL deviates  0.004		
8.0853	4309	0.027	----	22.6866		----			
8.1570	3869	0.022	----	22.7855		----			
8.2004	3285	0.028	----	22.8453		----			
8.2577	4004	0.019	0.978	22.9244	23:1 w4c	0.38	ECL deviates -0.002		
8.3141	2958	0.019	0.979	23.0021	23:0	0.28	ECL deviates  0.002	Reference -0.003	
8.3545	3761	0.028	----	23.0588		----			
8.5262	3790	0.022	----	23.2994		----			
8.7783	10459	0.032	0.992	23.6529	24:3 w3c	1.00	ECL deviates -0.002		
8.8344	11710	0.026	----	23.7315		----			
8.9405	9414	0.024	----	23.8802		----			
8.9816	1445	0.017	1.000	23.9378	24:1 w3c	0.14	ECL deviates -0.011		
9.0230	8921	0.020	1.001	23.9959	24:0	0.87	ECL deviates -0.004	Reference -0.008	
9.1824	1538	0.017	----	24.2192		----	> max rt		
9.2066	1224	0.018	----	24.2532		----	> max rt		
9.3898	10027	0.020	----	24.5099		----	> max rt		
9.4877	1493	0.018	----	24.6471		----	> max rt		

ECL Deviation: 0.006                            Reference ECL Shift: 0.007       Number Reference Peaks: 21
Total Response: 1295317                       Total Named: 1013375
Percent Named: 78.23%                         Total Amount: 1036528
Profile Comment:   Review report comments.

(No search libraries specified in method PLFAD1.)
