# Supplementary material for: Microbial regulation of soil carbon properties under nitrogen addition and plant inputs removal
Source: PeerJ. 2019 Jul 17;7:e7343. doi: 10.7717/peerj.7343 (PMC6642627; doi:10.7717/peerj.7343)
Supplement: File S1 — The raw data showed the soil microbial PLFAs files in the year of 2015 and 2016. Each file of rtf. represented the microbial PLFAs for each soil sample. In the Supplemental File, the Excel file named “Numbers” showed the plots names and the related rtf. file names. [file peerj-07-7343-s002.zip › supplementary files/2016/70.rtf]

Volume: DATA            File: E17C203.64A       Samp Ctr: 25                 ID Number: 5043 
Type: Samp                   Bottle: 11                      Method: PLFAD1 
Created: 12/20/2017 7:48:49 PM 
Sample ID: 70 


RT	Response	Ar/Ht	RFact	ECL	Peak Name	Percent	Comment1	Comment2	
0.7650	1.675E+9	0.016	----	7.6954	SOLVENT PEAK	----	< min rt		
0.9496	547	0.012	----	8.7472		----	< min rt		
1.8107	537	0.013	1.012	12.7212	13:0 anteiso	0.12	ECL deviates  0.012	Reference  0.015	
1.9896	1073	0.016	----	13.2307		----			
2.1393	3426	0.014	1.030	13.6078	14:0 iso	0.81	ECL deviates -0.006	Reference -0.005	
2.1593	498	0.011	----	13.6581		----			
2.2671	632	0.013	----	13.9297		----			
2.2933	3209	0.015	1.035	13.9955	14:0	0.76	ECL deviates -0.005	Reference -0.004	
2.3566	1175	0.013	----	14.1271	14:0 iso 3OH	----	ECL deviates  0.002		
2.4555	595	0.014	----	14.3314		----			
2.5075	3486	0.018	1.038	14.4389	15:1 iso w6c	0.83	ECL deviates  0.000		
2.5317	768	0.013	1.038	14.4889	15:4 w3c	0.18	ECL deviates -0.001		
2.5517	585	0.013	1.038	14.5303	15:1 anteiso w9c	0.14	ECL deviates  0.000		
2.5919	18822	0.015	1.038	14.6134	15:0 iso	4.48	ECL deviates -0.004	Reference -0.005	
2.6376	13394	0.015	1.039	14.7077	15:0 anteiso	3.19	ECL deviates -0.003	Reference -0.005	
2.7791	1721	0.014	1.039	15.0001	15:0	0.41	ECL deviates  0.000	Reference -0.002	
2.8087	1062	0.016	----	15.0526		----			
3.0045	465	0.012	1.038	15.3985	16:1 w7c alcohol	0.11	ECL deviates  0.002		
3.0315	2578	0.018	1.037	15.4461	15:0 DMA	0.61	ECL deviates -0.004		
3.1008	13532	0.016	1.037	15.5685	16:3 w6c	3.22	ECL deviates -0.007		
3.1293	8090	0.016	1.036	15.6188	16:0 iso	1.92	ECL deviates -0.001	Reference -0.004	
3.1851	1161	0.015	1.036	15.7174	16:0 anteiso	0.28	ECL deviates  0.002	Reference -0.001	
3.2154	3740	0.015	1.035	15.7709	16:1 w9c	0.89	ECL deviates -0.004		
3.2437	29979	0.018	1.035	15.8207	16:1 w7c	7.12	ECL deviates -0.004		
3.2955	8032	0.016	1.034	15.9123	16:1 w5c	1.91	ECL deviates  0.001		
3.3446	36838	0.015	1.034	15.9989	16:0	8.73	ECL deviates -0.001	Reference -0.005	
3.3759	2843	0.019	----	16.0489		----			
3.6135	19696	0.020	1.030	16.4247	16:0 10-methyl	4.65	ECL deviates  0.005		
3.6589	94227	0.017	1.029	16.4965	17:1 iso w9c	22.23	ECL deviates -0.001		
3.7392	4989	0.016	1.027	16.6236	17:0 iso	1.18	ECL deviates  0.000	Reference -0.005	
3.8001	5343	0.016	1.026	16.7199	17:0 anteiso	1.26	ECL deviates  0.000		
3.8490	2235	0.016	1.025	16.7972	17:1 w8c	0.53	ECL deviates  0.000		
3.9116	11763	0.018	1.024	16.8963	17:0 cyclo w7c	2.76	ECL deviates  0.003		
3.9790	1301	0.015	1.022	17.0027	17:0	0.31	ECL deviates  0.003	Reference -0.003	
4.0063	3047	0.016	1.022	17.0428	17:1 w7c 10-methyl	0.71	ECL deviates  0.000		
4.2556	2129	0.014	1.017	17.4069	17:0 10-methyl	0.50	ECL deviates  0.000		
4.3145	1021	0.022	----	17.4929		----			
4.3738	1669	0.016	1.014	17.5795	18:3 w6c	0.39	ECL deviates  0.000		
4.4048	1514	0.016	1.013	17.6248	18:0 iso	0.35	ECL deviates -0.002	Reference -0.008	
4.4342	663	0.016	----	17.6676		----			
4.4755	6279	0.016	1.012	17.7279	18:2 w6c	1.46	ECL deviates  0.001		
4.5082	18958	0.017	1.011	17.7756	18:1 w9c	4.40	ECL deviates  0.001		
4.5432	28515	0.018	1.010	17.8267	18:1 w7c	6.61	ECL deviates  0.000		
4.6061	4178	0.020	1.009	17.9185	18:1 w5c	0.97	ECL deviates -0.004		
4.6632	6727	0.016	1.008	18.0020	18:0	1.55	ECL deviates  0.002	Reference -0.005	
4.7228	2286	0.017	1.006	18.0854	18:1 w7c 10-methyl	0.53	ECL deviates  0.000		
4.8180	2222	0.046	----	18.2183		----	> max ar/ht		
4.9427	9570	0.021	1.002	18.3926	18:0 10-methyl	2.20	ECL deviates -0.002		
5.0592	3073	0.017	0.999	18.5553	19:3 w6c	0.70	ECL deviates -0.005		
5.1952	1455	0.025	----	18.7451		----			
5.2480	1186	0.016	0.995	18.8189	19:1 w8c	0.27	ECL deviates  0.008		
5.2839	1983	0.020	0.994	18.8690	19:0 cyclo w9c	0.45	ECL deviates -0.003		
5.3131	9015	0.019	0.994	18.9098	19:0 cyclo w7c	2.05	ECL deviates  0.000		
5.3822	61461	0.016	----	19.0063	19:0	----	ECL deviates  0.006		
5.5368	789	0.018	----	19.2166		----			
5.6698	1866	0.034	0.987	19.3974	20:4 w6c	0.42	ECL deviates -0.006		
5.8218	1315	0.026	----	19.6041		----			
5.9026	958	0.019	----	19.7139		----			
5.9469	1576	0.021	0.982	19.7742	20:1 w9c	0.35	ECL deviates  0.002		
5.9727	748	0.018	0.981	19.8092	20:1 w8c	0.17	ECL deviates -0.004		
6.1157	1888	0.018	0.979	20.0036	20:0	0.42	ECL deviates  0.004	Reference -0.005	
6.3706	2909	0.014	----	20.3497		----			
6.4014	22903	0.019	0.975	20.3915	20:0 10-methyl	5.12	ECL deviates -0.006		
6.5691	2598	0.019	----	20.6192		----			
6.6490	2620	0.023	----	20.7276		----			
6.7047	1129	0.015	0.972	20.8031	21:1 w8c	0.25	ECL deviates  0.005		
6.8212	1808	0.016	0.971	20.9612	21:1 w3c	0.40	ECL deviates  0.007		
7.3148	1221	0.025	----	21.6329		----			
7.3653	1572	0.019	----	21.7017		----			
7.4585	3557	0.020	----	21.8285		----			
7.5862	1821	0.016	0.970	22.0022	22:0	0.40	ECL deviates  0.002	Reference -0.007	
7.7793	103974	0.022	----	22.2687	Phthalate 2	----	ECL deviates -0.014		
8.0861	2004	0.018	----	22.6921		----			
8.2554	1394	0.017	0.978	22.9258	23:1 w4c	0.31	ECL deviates -0.001		
8.7946	2230	0.023	----	23.6808		----			
8.9409	2184	0.020	----	23.8861		----			
9.0199	1644	0.017	1.001	23.9968	24:0	0.38	ECL deviates -0.003	Reference -0.012	
9.3869	6866	0.019	----	24.5116		----	> max rt		
9.4868	1103	0.016	----	24.6518		----	> max rt		

ECL Deviation: 0.004                            Reference ECL Shift: 0.007       Number Reference Peaks: 16
Total Response: 462847                         Total Named: 426825
Percent Named: 92.22%                         Total Amount: 436036

(No search libraries specified in method PLFAD1.)
