# Supplementary material for: Microbial regulation of soil carbon properties under nitrogen addition and plant inputs removal
Source: PeerJ. 2019 Jul 17;7:e7343. doi: 10.7717/peerj.7343 (PMC6642627; doi:10.7717/peerj.7343)
Supplement: File S1 — The raw data showed the soil microbial PLFAs files in the year of 2015 and 2016. Each file of rtf. represented the microbial PLFAs for each soil sample. In the Supplemental File, the Excel file named “Numbers” showed the plots names and the related rtf. file names. [file peerj-07-7343-s002.zip › supplementary files/2016/71.rtf]

Volume: DATA            File: E17C203.64A       Samp Ctr: 26                 ID Number: 5044 
Type: Samp                   Bottle: 12                      Method: PLFAD1 
Created: 12/20/2017 8:17:12 PM 
Sample ID: 71 


RT	Response	Ar/Ht	RFact	ECL	Peak Name	Percent	Comment1	Comment2	
0.7649	1.693E+9	0.016	----	7.6978	SOLVENT PEAK	----	< min rt		
0.8801	1364	0.017	----	8.3543		----	< min rt		
0.9497	1055	0.013	----	8.7503		----	< min rt		
1.2973	631	0.014	----	10.7267		----			
1.7726	594	0.013	1.009	12.6026	13:0 iso	0.07	ECL deviates -0.010	Reference -0.009	
1.8097	656	0.014	1.012	12.7207	13:0 anteiso	0.08	ECL deviates  0.011	Reference  0.012	
1.9903	1123	0.015	----	13.2343		----			
2.1394	8407	0.017	1.030	13.6097	14:0 iso	1.08	ECL deviates -0.004	Reference -0.005	
2.1843	721	0.014	1.032	13.7226	14:0 anteiso	0.09	ECL deviates  0.007	Reference  0.006	
2.2672	762	0.013	----	13.9312		----			
2.2946	6314	0.015	1.035	14.0000	14:0	0.81	ECL deviates  0.000	Reference -0.001	
2.3564	1326	0.014	----	14.1279	14:0 iso 3OH	----	ECL deviates  0.003		
2.4566	799	0.017	----	14.3348		----			
2.5072	7123	0.017	1.038	14.4392	15:1 iso w6c	0.92	ECL deviates  0.000		
2.5302	1164	0.012	1.038	14.4867	15:4 w3c	0.15	ECL deviates -0.004		
2.5515	1312	0.012	1.038	14.5305	15:1 anteiso w9c	0.17	ECL deviates  0.001		
2.5922	38151	0.015	1.038	14.6147	15:0 iso	4.94	ECL deviates -0.002	Reference -0.004	
2.6383	25433	0.015	1.039	14.7098	15:0 anteiso	3.29	ECL deviates -0.001	Reference -0.003	
2.7080	889	0.016	1.039	14.8537	15:1 w6c	0.12	ECL deviates -0.006		
2.7789	4230	0.015	1.039	14.9999	15:0	0.55	ECL deviates  0.000	Reference -0.002	
2.8092	1939	0.015	----	15.0537		----			
2.9114	876	0.014	----	15.2341		----			
3.0308	6651	0.022	1.037	15.4447	15:0 DMA	0.86	ECL deviates -0.006		
3.1012	15976	0.016	1.037	15.5689	16:3 w6c	2.06	ECL deviates -0.007		
3.1295	17060	0.015	1.036	15.6189	16:0 iso	2.20	ECL deviates -0.001	Reference -0.004	
3.1839	2491	0.016	1.036	15.7149	16:0 anteiso	0.32	ECL deviates  0.000	Reference -0.003	
3.2147	7307	0.017	1.035	15.7692	16:1 w9c	0.94	ECL deviates -0.006		
3.2440	55962	0.017	1.035	15.8210	16:1 w7c	7.22	ECL deviates -0.003		
3.2956	16096	0.017	1.034	15.9119	16:1 w5c	2.07	ECL deviates  0.001		
3.3453	73194	0.016	1.034	15.9997	16:0	9.43	ECL deviates  0.000	Reference -0.004	
3.3750	4711	0.018	----	16.0470		----			
3.4729	2688	0.050	----	16.2017		----	> max ar/ht		
3.6140	37979	0.020	1.030	16.4247	16:0 10-methyl	4.87	ECL deviates  0.005		
3.6594	117764	0.017	1.029	16.4964	17:1 iso w9c	15.10	ECL deviates -0.002		
3.7404	11033	0.016	1.027	16.6245	17:0 iso	1.41	ECL deviates  0.001	Reference -0.003	
3.8006	12693	0.018	1.026	16.7196	17:0 anteiso	1.62	ECL deviates -0.001		
3.8493	5833	0.018	1.025	16.7965	17:1 w8c	0.75	ECL deviates  0.000		
3.9118	23564	0.019	1.024	16.8954	17:0 cyclo w7c	3.01	ECL deviates  0.002		
3.9794	3811	0.018	1.022	17.0022	17:0	0.49	ECL deviates  0.002	Reference -0.002	
4.0061	6303	0.016	1.022	17.0414	17:1 w7c 10-methyl	0.80	ECL deviates -0.002		
4.0522	1267	0.015	----	17.1085		----			
4.1427	1696	0.023	1.019	17.2407	16:0 2OH	0.22	ECL deviates  0.000		
4.2555	5362	0.015	1.017	17.4053	17:0 10-methyl	0.68	ECL deviates -0.002		
4.3163	2063	0.027	----	17.4939		----			
4.3754	2803	0.017	1.014	17.5802	18:3 w6c	0.35	ECL deviates  0.000		
4.4037	3043	0.018	1.013	17.6215	18:0 iso	0.38	ECL deviates -0.005	Reference -0.010	
4.4342	1023	0.015	----	17.6660		----			
4.4752	13303	0.018	1.012	17.7259	18:2 w6c	1.68	ECL deviates -0.001		
4.5082	39466	0.018	1.011	17.7740	18:1 w9c	4.97	ECL deviates  0.000		
4.5444	56149	0.018	1.010	17.8269	18:1 w7c	7.07	ECL deviates  0.000		
4.6048	10436	0.021	1.009	17.9149	18:1 w5c	1.31	ECL deviates -0.008		
4.6635	12987	0.018	1.008	18.0006	18:0	1.63	ECL deviates  0.001	Reference -0.005	
4.7229	4594	0.017	1.006	18.0837	18:1 w7c 10-methyl	0.58	ECL deviates -0.001		
4.7816	1581	0.021	1.005	18.1657	18:2 DMA	0.20	ECL deviates  0.006		
4.8164	3145	0.032	----	18.2144		----			
4.9431	23004	0.020	1.002	18.3911	18:0 10-methyl	2.87	ECL deviates -0.004		
5.0603	5030	0.021	0.999	18.5548	19:3 w6c	0.63	ECL deviates -0.005		
5.1970	3178	0.025	----	18.7456		----			
5.2469	2955	0.017	0.995	18.8154	19:1 w8c	0.37	ECL deviates  0.004		
5.2875	3663	0.018	0.994	18.8720	19:0 cyclo w9c	0.45	ECL deviates  0.000		
5.3122	17782	0.017	0.994	18.9064	19:0 cyclo w7c	2.20	ECL deviates -0.003		
5.3824	67641	0.016	----	19.0044	19:0	----	ECL deviates  0.004		
5.5337	2326	0.017	----	19.2102		----			
5.6165	1226	0.017	0.988	19.3227	19:0 cyclo 9,10 DMA	0.15	ECL deviates -0.001		
5.6493	2527	0.019	----	19.3674		----			
5.6690	1154	0.013	0.987	19.3941	20:4 w6c	0.14	ECL deviates -0.009		
5.8221	3602	0.035	----	19.6022		----	> max ar/ht		
5.9014	816	0.012	----	19.7100		----			
5.9446	4521	0.026	0.982	19.7686	20:1 w9c	0.55	ECL deviates -0.004		
5.9717	2062	0.021	0.981	19.8055	20:1 w8c	0.25	ECL deviates -0.007		
6.1156	3860	0.021	0.979	20.0012	20:0	0.47	ECL deviates  0.001	Reference -0.005	
6.2281	794	0.018	----	20.1538		----			
6.2591	1211	0.018	----	20.1958		----			
6.3715	4666	0.016	----	20.3484		----			
6.4025	31283	0.019	0.975	20.3905	20:0 10-methyl	3.80	ECL deviates -0.007		
6.4649	1621	0.018	----	20.4752		----			
6.5020	1832	0.024	----	20.5256		----			
6.5697	4563	0.024	----	20.6175		----			
6.6500	3539	0.023	----	20.7264		----			
6.7037	2771	0.017	0.972	20.7992	21:1 w8c	0.34	ECL deviates  0.001		
6.7665	1298	0.024	----	20.8846		----			
6.8194	3977	0.017	0.971	20.9563	21:1 w3c	0.48	ECL deviates  0.002		
6.8734	2742	0.023	----	21.0297		----			
6.9360	686	0.016	----	21.1149		----			
6.9662	1095	0.022	----	21.1560		----			
7.0589	759	0.017	----	21.2822		----			
7.1878	1280	0.029	0.969	21.4576	22:5 w3c	0.15	ECL deviates -0.010		
7.3120	4008	0.034	0.969	21.6267	22:0 iso	0.48	ECL deviates  0.009		
7.3383	5074	0.030	----	21.6625		----			
7.4181	1763	0.022	0.969	21.7711	22:1 w9c	0.21	ECL deviates -0.002		
7.4575	9645	0.021	----	21.8247		----			
7.5420	1709	0.020	0.969	21.9397	22:1 w3c	0.21	ECL deviates -0.007		
7.5884	3099	0.016	0.970	22.0028	22:0	0.37	ECL deviates  0.003	Reference -0.004	
7.7797	123330	0.017	----	22.2668		----			
8.0856	2138	0.020	----	22.6891		----			
8.1559	1381	0.018	----	22.7861		----			
8.2567	1894	0.016	0.978	22.9252	23:1 w4c	0.23	ECL deviates -0.001		
8.3121	896	0.016	0.979	23.0017	23:0	0.11	ECL deviates  0.002	Reference -0.006	
8.5230	705	0.017	----	23.2975		----			
8.7811	4904	0.031	0.992	23.6598	24:3 w3c	0.61	ECL deviates  0.005		
8.8337	4447	0.021	----	23.7336		----			
8.9418	3923	0.018	----	23.8853		----			
9.0212	3288	0.017	1.001	23.9968	24:0	0.41	ECL deviates -0.003	Reference -0.011	
9.1801	1306	0.024	----	24.2198		----	> max rt		
9.3875	6360	0.019	----	24.5108		----	> max rt		
9.4887	1024	0.015	----	24.6528		----	> max rt		

ECL Deviation: 0.004                            Reference ECL Shift: 0.006       Number Reference Peaks: 19
Total Response: 995188                         Total Named: 786261
Percent Named: 79.01%                         Total Amount: 802468

(No search libraries specified in method PLFAD1.)
