# Supplementary material for: Microbial regulation of soil carbon properties under nitrogen addition and plant inputs removal
Source: PeerJ. 2019 Jul 17;7:e7343. doi: 10.7717/peerj.7343 (PMC6642627; doi:10.7717/peerj.7343)
Supplement: File S1 — The raw data showed the soil microbial PLFAs files in the year of 2015 and 2016. Each file of rtf. represented the microbial PLFAs for each soil sample. In the Supplemental File, the Excel file named “Numbers” showed the plots names and the related rtf. file names. [file peerj-07-7343-s002.zip › supplementary files/2016/72.rtf]

Volume: DATA            File: E17C203.64A       Samp Ctr: 28                 ID Number: 5045 
Type: Samp                   Bottle: 14                      Method: PLFAD1 
Created: 12/20/2017 9:14:15 PM 
Sample ID: 72 


RT	Response	Ar/Ht	RFact	ECL	Peak Name	Percent	Comment1	Comment2	
0.7652	1.697E+9	0.016	----	7.7162	SOLVENT PEAK	----	< min rt		
0.9501	814	0.012	----	8.7659		----	< min rt		
1.9888	861	0.020	----	13.2365		----			
2.1389	1588	0.014	1.026	13.6130	14:0 iso	0.45	ECL deviates -0.001	Reference -0.006	
2.2936	1916	0.016	1.032	14.0010	14:0	0.55	ECL deviates  0.001	Reference -0.003	
2.3570	701	0.012	----	14.1323	14:0 iso 3OH	----	ECL deviates  0.007		
2.5073	2584	0.019	1.037	14.4419	15:1 iso w6c	0.75	ECL deviates  0.003		
2.5917	12791	0.015	1.038	14.6159	15:0 iso	3.69	ECL deviates -0.001	Reference -0.005	
2.6379	9956	0.015	1.039	14.7111	15:0 anteiso	2.88	ECL deviates  0.000	Reference -0.004	
2.7782	1425	0.014	1.040	15.0002	15:0	0.41	ECL deviates  0.000	Reference -0.003	
2.8087	759	0.016	----	15.0545		----			
3.0308	2023	0.017	1.039	15.4462	15:0 DMA	0.58	ECL deviates -0.004		
3.1007	11186	0.016	1.039	15.5694	16:3 w6c	3.23	ECL deviates -0.006		
3.1290	5915	0.015	1.038	15.6194	16:0 iso	1.71	ECL deviates  0.000	Reference -0.003	
3.1857	835	0.015	1.038	15.7193	16:0 anteiso	0.24	ECL deviates  0.004	Reference  0.001	
3.2154	2784	0.015	1.038	15.7717	16:1 w9c	0.80	ECL deviates -0.003		
3.2436	20600	0.016	1.037	15.8214	16:1 w7c	5.95	ECL deviates -0.003		
3.2948	6060	0.016	1.037	15.9119	16:1 w5c	1.75	ECL deviates  0.001		
3.3444	29538	0.015	1.036	15.9993	16:0	8.52	ECL deviates -0.001	Reference -0.004	
3.3741	2135	0.017	----	16.0466		----			
3.6129	16492	0.018	1.032	16.4230	16:0 10-methyl	4.74	ECL deviates  0.003		
3.6582	86134	0.017	1.031	16.4944	17:1 iso w9c	24.72	ECL deviates -0.004		
3.7400	4356	0.015	1.030	16.6235	17:0 iso	1.25	ECL deviates  0.000	Reference -0.003	
3.7999	4490	0.017	1.029	16.7179	17:0 anteiso	1.29	ECL deviates -0.002		
3.8489	1922	0.017	1.028	16.7951	17:1 w8c	0.55	ECL deviates -0.002		
3.9116	9639	0.019	1.027	16.8941	17:0 cyclo w7c	2.75	ECL deviates  0.001		
3.9783	1272	0.016	1.025	16.9992	17:0	0.36	ECL deviates -0.001	Reference -0.003	
4.0060	2873	0.016	1.025	17.0399	17:1 w7c 10-methyl	0.82	ECL deviates -0.003		
4.2550	1789	0.015	1.020	17.4028	17:0 10-methyl	0.51	ECL deviates -0.004		
4.3143	1121	0.025	----	17.4892		----			
4.3739	1497	0.016	1.017	17.5763	18:3 w6c	0.42	ECL deviates -0.004		
4.4034	1414	0.018	1.016	17.6192	18:0 iso	0.40	ECL deviates -0.007	Reference -0.010	
4.4744	4637	0.016	1.015	17.7228	18:2 w6c	1.31	ECL deviates -0.004		
4.5071	16113	0.017	1.014	17.7704	18:1 w9c	4.55	ECL deviates -0.004		
4.5439	25193	0.017	1.013	17.8241	18:1 w7c	7.10	ECL deviates -0.003		
4.6089	3091	0.022	1.012	17.9187	18:1 w5c	0.87	ECL deviates -0.004		
4.6633	6212	0.018	1.010	17.9981	18:0	1.75	ECL deviates -0.002	Reference -0.004	
4.7229	2221	0.016	1.009	18.0814	18:1 w7c 10-methyl	0.62	ECL deviates -0.004		
4.9421	8796	0.020	1.004	18.3873	18:0 10-methyl	2.46	ECL deviates -0.008		
5.0593	2556	0.019	1.002	18.5509	19:3 w6c	0.71	ECL deviates -0.009		
5.1941	1142	0.023	----	18.7391		----		Reference  0.009	
5.2447	1126	0.017	0.998	18.8097	19:1 w8c	0.31	ECL deviates -0.001		
5.3117	9295	0.021	0.996	18.9031	19:0 cyclo w7c	2.58	ECL deviates -0.007		
5.3811	59321	0.016	----	19.0001	19:0	----	ECL deviates  0.000		
5.5317	764	0.016	----	19.2046		----			
5.8228	1788	0.040	----	19.6000		----	> max ar/ht		
5.9007	842	0.018	----	19.7058		----			
5.9421	1627	0.021	0.984	19.7620	20:1 w9c	0.45	ECL deviates -0.011		
5.9729	924	0.020	0.984	19.8038	20:1 w8c	0.25	ECL deviates -0.009		
6.1163	1594	0.019	0.981	19.9986	20:0	0.44	ECL deviates -0.001	Reference -0.004	
6.3702	2873	0.017	----	20.3428		----			
6.4006	20301	0.018	0.978	20.3840	20:0 10-methyl	5.52	ECL deviates -0.013		
6.4392	702	0.016	----	20.4363		----			
6.4651	741	0.015	----	20.4715		----			
6.5699	2170	0.020	----	20.6137		----			
6.6509	2257	0.022	----	20.7235		----			
6.7037	1062	0.015	0.975	20.7951	21:1 w8c	0.29	ECL deviates -0.003		
6.8220	1604	0.015	0.974	20.9555	21:1 w3c	0.43	ECL deviates  0.001		
7.3649	608	0.013	----	21.6943		----			
7.4600	2099	0.021	----	21.8237		----			
7.5861	1395	0.015	0.975	21.9954	22:0	0.38	ECL deviates -0.005	Reference -0.009	
7.7783	99239	0.019	----	22.2617		----			
8.0849	1606	0.019	----	22.6867		----			
8.2542	905	0.017	0.987	22.9214	23:1 w4c	0.25	ECL deviates -0.005		
8.7937	1607	0.022	----	23.6803		----			
8.9407	1441	0.019	----	23.8875		----			
9.0220	1477	0.016	1.017	24.0021	24:0	0.42	ECL deviates  0.002	Reference -0.004	
9.3871	8314	0.017	----	24.5166		----	> max rt		
9.4908	981	0.015	----	24.6627		----	> max rt		

ECL Deviation: 0.005                            Reference ECL Shift: 0.005       Number Reference Peaks: 16
Total Response: 475964                         Total Named: 351207
Percent Named: 73.79%                         Total Amount: 359406

(No search libraries specified in method PLFAD1.)
