# Supplementary material for: Microbial regulation of soil carbon properties under nitrogen addition and plant inputs removal
Source: PeerJ. 2019 Jul 17;7:e7343. doi: 10.7717/peerj.7343 (PMC6642627; doi:10.7717/peerj.7343)
Supplement: File S1 — The raw data showed the soil microbial PLFAs files in the year of 2015 and 2016. Each file of rtf. represented the microbial PLFAs for each soil sample. In the Supplemental File, the Excel file named “Numbers” showed the plots names and the related rtf. file names. [file peerj-07-7343-s002.zip › supplementary files/2016/73.rtf]

Volume: DATA            File: E17C203.64A       Samp Ctr: 29                 ID Number: 5046 
Type: Samp                   Bottle: 15                      Method: PLFAD1 
Created: 12/20/2017 9:42:38 PM 
Sample ID: 73 


RT	Response	Ar/Ht	RFact	ECL	Peak Name	Percent	Comment1	Comment2	
0.7647	1.705E+9	0.016	----	7.7055	SOLVENT PEAK	----	< min rt		
0.9509	1069	0.013	----	8.7634		----	< min rt		
1.2977	443	0.011	0.893	10.7209	11:0 anteiso	0.06	ECL deviates  0.016		
1.8108	566	0.013	1.002	12.7195	13:0 anteiso	0.08	ECL deviates  0.010	Reference  0.018	
1.9899	1188	0.018	----	13.2287		----			
2.1399	5670	0.016	1.026	13.6065	14:0 iso	0.85	ECL deviates -0.008	Reference -0.003	
2.2946	5783	0.016	1.032	13.9963	14:0	0.87	ECL deviates -0.004	Reference -0.001	
2.3571	1220	0.014	----	14.1257	14:0 iso 3OH	----	ECL deviates  0.001		
2.5082	6688	0.019	1.037	14.4382	15:1 iso w6c	1.01	ECL deviates -0.001		
2.5292	953	0.012	1.037	14.4815	15:4 w3c	0.14	ECL deviates -0.009		
2.5521	1062	0.011	1.038	14.5288	15:1 anteiso w9c	0.16	ECL deviates -0.001		
2.5929	34142	0.015	1.038	14.6133	15:0 iso	5.17	ECL deviates -0.004	Reference -0.002	
2.6389	22248	0.015	1.039	14.7084	15:0 anteiso	3.37	ECL deviates -0.003	Reference -0.002	
2.7096	809	0.017	1.039	14.8546	15:1 w6c	0.12	ECL deviates -0.005		
2.7793	3339	0.015	1.040	14.9986	15:0	0.51	ECL deviates -0.001	Reference -0.001	
2.8102	1319	0.015	----	15.0536		----			
2.9123	643	0.014	----	15.2342		----			
3.0074	780	0.012	1.039	15.4023	16:1 w7c alcohol	0.12	ECL deviates  0.006		
3.0315	4382	0.019	1.039	15.4449	15:0 DMA	0.66	ECL deviates -0.006		
3.1016	13932	0.016	1.039	15.5690	16:3 w6c	2.11	ECL deviates -0.007		
3.1310	14490	0.016	1.038	15.6210	16:0 iso	2.20	ECL deviates  0.001	Reference  0.000	
3.1849	1762	0.014	1.038	15.7163	16:0 anteiso	0.27	ECL deviates  0.001	Reference  0.000	
3.2158	6307	0.016	1.038	15.7710	16:1 w9c	0.95	ECL deviates -0.004		
3.2451	49329	0.017	1.037	15.8227	16:1 w7c	7.46	ECL deviates -0.002		
3.2976	13652	0.017	1.037	15.9157	16:1 w5c	2.06	ECL deviates  0.005		
3.3181	1598	0.008	1.036	15.9519	16:1 w3c	0.24	ECL deviates  0.000		
3.3463	68856	0.016	1.036	16.0017	16:0	10.41	ECL deviates  0.002	Reference  0.000	
3.3769	3144	0.018	----	16.0502		----			
3.6156	31822	0.019	1.032	16.4274	16:0 10-methyl	4.79	ECL deviates  0.007		
3.6603	103927	0.016	1.031	16.4980	17:1 iso w9c	15.64	ECL deviates  0.000		
3.7413	9398	0.015	1.030	16.6260	17:0 iso	1.41	ECL deviates  0.002	Reference -0.001	
3.8016	9889	0.016	1.029	16.7212	17:0 anteiso	1.48	ECL deviates  0.001		
3.8508	4844	0.017	1.028	16.7990	17:1 w8c	0.73	ECL deviates  0.002		
3.9134	21187	0.019	1.027	16.8980	17:0 cyclo w7c	3.17	ECL deviates  0.004		
3.9810	3165	0.017	1.025	17.0046	17:0	0.47	ECL deviates  0.005	Reference  0.001	
4.0082	4601	0.017	1.025	17.0443	17:1 w7c 10-methyl	0.69	ECL deviates  0.001		
4.0535	938	0.013	----	17.1105		----			
4.1430	725	0.013	1.022	17.2411	16:0 2OH	0.11	ECL deviates  0.001		
4.2578	5030	0.019	1.020	17.4087	17:0 10-methyl	0.75	ECL deviates  0.002		
4.3191	1894	0.027	----	17.4982		----			
4.3764	2151	0.016	1.017	17.5819	18:3 w6c	0.32	ECL deviates  0.002		
4.4040	2669	0.017	1.016	17.6221	18:0 iso	0.40	ECL deviates -0.004	Reference -0.009	
4.4323	963	0.016	----	17.6635		----			
4.4769	13018	0.018	1.015	17.7286	18:2 w6c	1.93	ECL deviates  0.001		
4.5092	34268	0.017	1.014	17.7757	18:1 w9c	5.07	ECL deviates  0.001		
4.5458	51832	0.018	1.013	17.8292	18:1 w7c	7.66	ECL deviates  0.002		
4.6056	8179	0.020	1.012	17.9164	18:1 w5c	1.21	ECL deviates -0.007		
4.6653	11490	0.018	1.010	18.0035	18:0	1.69	ECL deviates  0.004	Reference -0.001	
4.7244	4506	0.017	1.009	18.0861	18:1 w7c 10-methyl	0.66	ECL deviates  0.001		
4.7836	1200	0.023	1.008	18.1688	18:2 DMA	0.18	ECL deviates  0.009		
4.8224	1422	0.028	1.007	18.2230	18:1 w9c DMA	0.21	ECL deviates -0.014		
4.9438	18013	0.019	1.004	18.3925	18:0 10-methyl	2.64	ECL deviates -0.003		
5.0611	3438	0.018	1.002	18.5563	19:3 w6c	0.50	ECL deviates -0.004		
5.2000	2486	0.026	----	18.7503		----			
5.2460	2103	0.018	0.998	18.8146	19:1 w8c	0.31	ECL deviates  0.004		
5.2868	2544	0.017	0.997	18.8714	19:0 cyclo w9c	0.37	ECL deviates -0.001		
5.3139	14410	0.019	0.996	18.9092	19:0 cyclo w7c	2.09	ECL deviates -0.001		
5.3829	59190	0.017	----	19.0056	19:0	----	ECL deviates  0.006		
5.5375	1303	0.017	----	19.2156		----			
5.5799	550	0.014	----	19.2732		----			
5.6506	1594	0.017	----	19.3691		----			
5.6728	956	0.013	0.989	19.3993	20:4 w6c	0.14	ECL deviates -0.004		
5.8227	2832	0.033	----	19.6028		----			
5.9041	1972	0.019	----	19.7134		----			
5.9461	3040	0.021	0.984	19.7703	20:1 w9c	0.44	ECL deviates -0.002		
5.9745	1559	0.022	0.984	19.8090	20:1 w8c	0.22	ECL deviates -0.004		
6.1147	3137	0.019	0.981	19.9992	20:0	0.45	ECL deviates -0.001	Reference -0.006	
6.2237	608	0.015	----	20.1470		----			
6.2594	1070	0.018	----	20.1953		----			
6.3727	4142	0.018	----	20.3489		----			
6.4021	24257	0.020	0.978	20.3887	20:0 10-methyl	3.46	ECL deviates -0.008		
6.4367	744	0.011	----	20.4355		----			
6.4649	1137	0.017	----	20.4738		----			
6.5692	3360	0.023	----	20.6151		----			
6.6359	4365	0.027	----	20.7054		----			
6.7035	1741	0.016	0.975	20.7969	21:1 w8c	0.25	ECL deviates -0.001		
6.7689	1077	0.024	----	20.8855		----			
6.8223	2515	0.017	0.974	20.9578	21:1 w3c	0.36	ECL deviates  0.004		
6.8735	1250	0.027	----	21.0273		----			
7.3104	3166	0.037	0.973	21.6210	22:0 iso	----	> max ar/ht		
7.3666	4648	0.023	----	21.6974		----			
7.4615	4221	0.024	----	21.8263		----			
7.5444	986	0.016	0.975	21.9389	22:1 w3c	0.14	ECL deviates -0.008		
7.5891	3069	0.018	0.975	21.9996	22:0	0.44	ECL deviates  0.000	Reference -0.005	
7.7814	108807	0.019	----	22.2655		----			
8.0856	4227	0.019	----	22.6862		----			
8.2574	1560	0.018	0.987	22.9238	23:1 w4c	0.22	ECL deviates -0.003		
8.3127	1114	0.023	0.988	23.0003	23:0	0.16	ECL deviates  0.000	Reference -0.002	
8.5256	823	0.017	----	23.2993		----			
8.7961	5257	0.023	----	23.6793		----			
8.8385	1841	0.024	----	23.7387		----			
8.9418	1910	0.017	----	23.8838		----			
9.0242	2789	0.016	1.017	23.9995	24:0	0.41	ECL deviates -0.001	Reference -0.001	
9.3891	8878	0.017	----	24.5118		----	> max rt		
9.4891	3064	0.017	----	24.6521		----	> max rt		

ECL Deviation: 0.005                            Reference ECL Shift: 0.005       Number Reference Peaks: 17
Total Response: 842823                         Total Named: 669345
Percent Named: 79.42%                         Total Amount: 688575
Profile Comment:   Review report comments.

(No search libraries specified in method PLFAD1.)
