# Supplementary material for: Microbial regulation of soil carbon properties under nitrogen addition and plant inputs removal
Source: PeerJ. 2019 Jul 17;7:e7343. doi: 10.7717/peerj.7343 (PMC6642627; doi:10.7717/peerj.7343)
Supplement: File S1 — The raw data showed the soil microbial PLFAs files in the year of 2015 and 2016. Each file of rtf. represented the microbial PLFAs for each soil sample. In the Supplemental File, the Excel file named “Numbers” showed the plots names and the related rtf. file names. [file peerj-07-7343-s002.zip › supplementary files/2016/74.rtf]

Volume: DATA            File: E17C203.64A       Samp Ctr: 30                 ID Number: 5047 
Type: Samp                   Bottle: 16                      Method: PLFAD1 
Created: 12/20/2017 10:11:15 PM 
Sample ID: 74 


RT	Response	Ar/Ht	RFact	ECL	Peak Name	Percent	Comment1	Comment2	
0.7652	1.69E+9	0.016	----	7.7070	SOLVENT PEAK	----	< min rt		
0.9512	598	0.012	----	8.7641		----	< min rt		
1.7729	594	0.013	0.999	12.6021	13:0 iso	0.11	ECL deviates -0.010	Reference -0.006	
1.8106	677	0.012	1.002	12.7221	13:0 anteiso	0.13	ECL deviates  0.013	Reference  0.017	
1.9908	1106	0.015	----	13.2332		----			
2.1401	5374	0.017	1.026	13.6084	14:0 iso	1.05	ECL deviates -0.006	Reference -0.003	
2.1852	551	0.013	1.028	13.7218	14:0 anteiso	0.11	ECL deviates  0.006	Reference  0.009	
2.2695	819	0.014	----	13.9337		----			
2.2946	4181	0.016	1.032	13.9969	14:0	0.82	ECL deviates -0.003	Reference -0.001	
2.3584	1250	0.012	----	14.1287	14:0 iso 3OH	----	ECL deviates  0.004		
2.4568	554	0.012	----	14.3318		----			
2.5088	5404	0.018	1.037	14.4392	15:1 iso w6c	1.07	ECL deviates  0.000		
2.5310	784	0.011	1.037	14.4850	15:4 w3c	0.16	ECL deviates -0.005		
2.5555	976	0.013	1.038	14.5357	15:1 anteiso w9c	0.19	ECL deviates  0.006		
2.5941	25802	0.016	1.038	14.6153	15:0 iso	5.11	ECL deviates -0.002	Reference  0.000	
2.6396	17563	0.015	1.039	14.7093	15:0 anteiso	3.48	ECL deviates -0.002	Reference  0.000	
2.7098	683	0.017	1.039	14.8541	15:1 w6c	0.14	ECL deviates -0.006		
2.7798	2329	0.014	1.040	14.9985	15:0	0.46	ECL deviates -0.001	Reference  0.000	
2.8105	1301	0.015	----	15.0529		----			
3.0333	3930	0.020	1.039	15.4465	15:0 DMA	0.78	ECL deviates -0.004		
3.1027	14346	0.016	1.039	15.5691	16:3 w6c	2.84	ECL deviates -0.007		
3.1312	10248	0.016	1.038	15.6194	16:0 iso	2.03	ECL deviates  0.000	Reference  0.000	
3.1889	1231	0.014	1.038	15.7214	16:0 anteiso	0.24	ECL deviates  0.006	Reference  0.007	
3.2166	4324	0.015	1.038	15.7702	16:1 w9c	0.86	ECL deviates -0.005		
3.2462	38236	0.016	1.037	15.8224	16:1 w7c	7.57	ECL deviates -0.002		
3.2991	9955	0.018	1.037	15.9160	16:1 w5c	1.97	ECL deviates  0.005		
3.3471	45017	0.016	1.036	16.0007	16:0	8.90	ECL deviates  0.001	Reference  0.001	
3.3770	2738	0.018	----	16.0479		----			
3.6167	22950	0.019	1.032	16.4263	16:0 10-methyl	4.52	ECL deviates  0.006		
3.6611	101193	0.016	1.031	16.4963	17:1 iso w9c	19.92	ECL deviates -0.002		
3.7432	6081	0.016	1.030	16.6259	17:0 iso	1.20	ECL deviates  0.002	Reference  0.002	
3.8031	6621	0.017	1.029	16.7205	17:0 anteiso	1.30	ECL deviates  0.000		
3.8524	3433	0.017	1.028	16.7983	17:1 w8c	0.67	ECL deviates  0.001		
3.9148	13985	0.018	1.027	16.8968	17:0 cyclo w7c	2.74	ECL deviates  0.003		
3.9811	2029	0.015	1.025	17.0014	17:0	0.40	ECL deviates  0.001	Reference  0.001	
4.0078	3830	0.017	1.025	17.0403	17:1 w7c 10-methyl	0.75	ECL deviates -0.003		
4.0536	744	0.015	----	17.1073		----			
4.1419	1056	0.022	1.022	17.2361	16:0 2OH	0.21	ECL deviates -0.004		
4.2581	3115	0.016	1.020	17.4056	17:0 10-methyl	0.61	ECL deviates -0.001		
4.3181	1272	0.025	----	17.4931		----			
4.3772	1830	0.017	1.017	17.5794	18:3 w6c	0.36	ECL deviates -0.001		
4.4058	1808	0.017	1.016	17.6210	18:0 iso	0.35	ECL deviates -0.006	Reference -0.006	
4.4369	762	0.016	----	17.6664		----			
4.4775	7553	0.016	1.015	17.7257	18:2 w6c	1.46	ECL deviates -0.001		
4.5109	23111	0.017	1.014	17.7744	18:1 w9c	4.47	ECL deviates  0.000		
4.5468	33599	0.016	1.013	17.8268	18:1 w7c	6.50	ECL deviates  0.000		
4.6084	4904	0.021	1.012	17.9167	18:1 w5c	0.95	ECL deviates -0.006		
4.6664	7807	0.019	1.010	18.0013	18:0	1.51	ECL deviates  0.001	Reference  0.000	
4.7253	2820	0.017	1.009	18.0835	18:1 w7c 10-methyl	0.54	ECL deviates -0.001		
4.8195	2390	0.048	----	18.2151		----	> max ar/ht		
4.9455	11895	0.021	1.004	18.3910	18:0 10-methyl	2.28	ECL deviates -0.004		
5.0632	3182	0.016	1.002	18.5554	19:3 w6c	0.61	ECL deviates -0.005		
5.1984	1733	0.024	----	18.7441		----			
5.2496	1456	0.018	0.998	18.8157	19:1 w8c	0.28	ECL deviates  0.005		
5.2878	1792	0.017	0.997	18.8689	19:0 cyclo w9c	0.34	ECL deviates -0.003		
5.3133	8729	0.016	0.996	18.9046	19:0 cyclo w7c	1.66	ECL deviates -0.005		
5.3841	57708	0.017	----	19.0034	19:0	----	ECL deviates  0.003		
5.5371	1242	0.016	----	19.2112		----			
5.6547	1035	0.018	----	19.3709		----			
5.6723	648	0.013	0.989	19.3948	20:4 w6c	0.12	ECL deviates -0.009		
5.8248	1905	0.037	----	19.6019		----	> max ar/ht		
5.9044	1327	0.017	----	19.7100		----			
5.9461	2154	0.022	0.984	19.7667	20:1 w9c	0.40	ECL deviates -0.006		
5.9744	1243	0.022	0.984	19.8051	20:1 w8c	0.23	ECL deviates -0.008		
6.1168	1882	0.019	0.981	19.9986	20:0	0.35	ECL deviates -0.001	Reference -0.004	
6.3732	2935	0.015	----	20.3462		----			
6.4037	23462	0.016	0.978	20.3875	20:0 10-methyl	4.38	ECL deviates -0.010		
6.5722	2860	0.019	----	20.6160		----			
6.6527	3179	0.027	----	20.7250		----			
6.7049	1201	0.016	0.975	20.7958	21:1 w8c	0.22	ECL deviates -0.002		
6.8228	2121	0.017	0.974	20.9556	21:1 w3c	0.39	ECL deviates  0.002		
7.3160	1793	0.033	0.973	21.6264	22:0 iso	0.33	ECL deviates  0.009		
7.3661	3428	0.021	----	21.6947		----			
7.4604	4453	0.024	0.974	21.8229	22:1 w8c	0.83	ECL deviates  0.009		
7.5465	783	0.017	0.975	21.9400	22:1 w3c	0.15	ECL deviates -0.007		
7.5909	1894	0.017	0.975	22.0004	22:0	0.35	ECL deviates  0.000	Reference -0.002	
7.7818	104270	0.018	----	22.2649		----			
8.0872	3710	0.019	----	22.6878		----			
8.2578	1115	0.017	0.987	22.9241	23:1 w4c	0.21	ECL deviates -0.002		
8.7964	4115	0.020	----	23.6809		----			
8.8395	961	0.020	----	23.7415		----			
8.9405	2340	0.018	----	23.8837		----			
9.0234	1996	0.016	1.017	24.0003	24:0	0.39	ECL deviates  0.000	Reference -0.002	
9.3884	6634	0.019	----	24.5139		----	> max rt		
9.4909	2526	0.016	----	24.6581		----	> max rt		

ECL Deviation: 0.005                            Reference ECL Shift: 0.005       Number Reference Peaks: 18
Total Response: 658461                         Total Named: 511733
Percent Named: 77.72%                         Total Amount: 524001

(No search libraries specified in method PLFAD1.)
