# Supplementary material for: Microbial regulation of soil carbon properties under nitrogen addition and plant inputs removal
Source: PeerJ. 2019 Jul 17;7:e7343. doi: 10.7717/peerj.7343 (PMC6642627; doi:10.7717/peerj.7343)
Supplement: File S1 — The raw data showed the soil microbial PLFAs files in the year of 2015 and 2016. Each file of rtf. represented the microbial PLFAs for each soil sample. In the Supplemental File, the Excel file named “Numbers” showed the plots names and the related rtf. file names. [file peerj-07-7343-s002.zip › supplementary files/2016/75.rtf]

Volume: DATA            File: E17C203.64A       Samp Ctr: 31                 ID Number: 5048 
Type: Samp                   Bottle: 17                      Method: PLFAD1 
Created: 12/20/2017 10:39:56 PM 
Sample ID: 75 


RT	Response	Ar/Ht	RFact	ECL	Peak Name	Percent	Comment1	Comment2	
0.7654	1.663E+9	0.015	----	7.7116	SOLVENT PEAK	----	< min rt		
1.6463	735	0.016	----	12.1985		----			
1.8088	523	0.013	1.002	12.7172	13:0 anteiso	0.12	ECL deviates  0.008	Reference  0.011	
2.1394	4145	0.016	1.026	13.6084	14:0 iso	0.98	ECL deviates -0.006	Reference -0.005	
2.2697	837	0.014	----	13.9363		----			
2.2939	3422	0.015	1.032	13.9973	14:0	0.81	ECL deviates -0.003	Reference -0.002	
2.3576	1290	0.013	----	14.1292	14:0 iso 3OH	----	ECL deviates  0.004		
2.4557	563	0.013	----	14.3320		----			
2.5076	4267	0.017	1.037	14.4392	15:1 iso w6c	1.02	ECL deviates  0.000		
2.5304	942	0.013	1.037	14.4863	15:4 w3c	0.22	ECL deviates -0.004		
2.5506	670	0.012	1.038	14.5281	15:1 anteiso w9c	0.16	ECL deviates -0.002		
2.5924	23844	0.015	1.038	14.6144	15:0 iso	5.69	ECL deviates -0.003	Reference -0.004	
2.6379	16679	0.016	1.039	14.7084	15:0 anteiso	3.98	ECL deviates -0.003	Reference -0.004	
2.7786	2056	0.013	1.040	14.9991	15:0	0.49	ECL deviates -0.001	Reference -0.003	
2.8106	944	0.015	----	15.0560		----			
3.0315	3593	0.021	1.039	15.4466	15:0 DMA	0.86	ECL deviates -0.004		
3.1012	9613	0.016	1.039	15.5697	16:3 w6c	2.29	ECL deviates -0.006		
3.1298	8488	0.017	1.038	15.6203	16:0 iso	2.03	ECL deviates  0.001	Reference -0.002	
3.1853	1153	0.015	1.038	15.7184	16:0 anteiso	0.28	ECL deviates  0.003	Reference  0.001	
3.2165	4891	0.016	1.038	15.7735	16:1 w9c	1.17	ECL deviates -0.001		
3.2445	32301	0.016	1.037	15.8230	16:1 w7c	7.70	ECL deviates -0.001		
3.2961	10310	0.017	1.037	15.9142	16:1 w5c	2.46	ECL deviates  0.003		
3.3449	42808	0.016	1.036	16.0005	16:0	10.19	ECL deviates  0.001	Reference -0.003	
3.3758	2826	0.017	----	16.0496		----			
3.4471	942	0.015	----	16.1622		----			
3.6130	25534	0.019	1.032	16.4242	16:0 10-methyl	6.06	ECL deviates  0.004		
3.6583	51008	0.018	1.031	16.4958	17:1 iso w9c	12.09	ECL deviates -0.002		
3.7401	5799	0.016	1.030	16.6250	17:0 iso	1.37	ECL deviates  0.001	Reference -0.003	
3.8010	5666	0.017	1.029	16.7211	17:0 anteiso	1.34	ECL deviates  0.001		
3.8489	2623	0.017	1.028	16.7968	17:1 w8c	0.62	ECL deviates  0.000		
3.9120	15382	0.018	1.027	16.8963	17:0 cyclo w7c	3.63	ECL deviates  0.003		
3.9797	1797	0.017	1.025	17.0033	17:0	0.42	ECL deviates  0.003	Reference -0.001	
4.0056	3108	0.015	1.025	17.0412	17:1 w7c 10-methyl	0.73	ECL deviates -0.002		
4.0523	730	0.014	----	17.1094		----			
4.1179	1559	0.024	----	17.2051		----			
4.2572	2302	0.016	1.020	17.4084	17:0 10-methyl	0.54	ECL deviates  0.001		
4.3166	1256	0.026	----	17.4951		----			
4.3768	1516	0.016	1.017	17.5829	18:3 w6c	0.35	ECL deviates  0.003		
4.4025	1712	0.017	1.016	17.6205	18:0 iso	0.40	ECL deviates -0.006	Reference -0.011	
4.4303	640	0.014	----	17.6610		----			
4.4745	6277	0.017	1.015	17.7255	18:2 w6c	1.46	ECL deviates -0.002		
4.5075	22282	0.017	1.014	17.7737	18:1 w9c	5.19	ECL deviates -0.001		
4.5439	37145	0.017	1.013	17.8267	18:1 w7c	8.65	ECL deviates  0.000		
4.6088	4063	0.022	1.012	17.9214	18:1 w5c	0.94	ECL deviates -0.002		
4.6641	7381	0.018	1.010	18.0021	18:0	1.71	ECL deviates  0.002	Reference -0.003	
4.7239	3484	0.019	1.009	18.0857	18:1 w7c 10-methyl	0.81	ECL deviates  0.001		
4.8180	2448	0.045	----	18.2172		----	> max ar/ht		
4.9435	10836	0.022	1.004	18.3924	18:0 10-methyl	2.50	ECL deviates -0.003		
5.0594	1696	0.017	1.002	18.5542	19:3 w6c	0.39	ECL deviates -0.006		
5.1949	1413	0.023	----	18.7435		----			
5.2468	1350	0.019	0.998	18.8158	19:1 w8c	0.31	ECL deviates  0.005		
5.3126	14177	0.018	0.996	18.9077	19:0 cyclo w7c	3.25	ECL deviates -0.002		
5.3823	54745	0.016	----	19.0050	19:0	----	ECL deviates  0.005		
5.6719	1479	0.027	0.989	19.3984	20:4 w6c	0.34	ECL deviates -0.005		
5.8241	717	0.018	----	19.6050		----			
5.9040	1757	0.025	----	19.7134		----			
5.9475	1683	0.018	0.984	19.7725	20:1 w9c	0.38	ECL deviates  0.000		
5.9718	1024	0.021	0.984	19.8056	20:1 w8c	0.23	ECL deviates -0.007		
6.1171	1778	0.019	0.981	20.0028	20:0	0.40	ECL deviates  0.003	Reference -0.003	
6.3717	1923	0.015	----	20.3478		----			
6.4010	15506	0.017	0.978	20.3875	20:0 10-methyl	3.48	ECL deviates -0.009		
6.5691	1562	0.019	----	20.6153		----			
6.6520	1853	0.024	----	20.7277		----			
6.7017	1156	0.015	0.975	20.7950	21:1 w8c	0.26	ECL deviates -0.003		
6.8210	2299	0.020	0.974	20.9567	21:1 w3c	0.51	ECL deviates  0.003		
7.4039	837	0.020	0.974	21.7488	22:2 w6c	0.19	ECL deviates  0.010		
7.5883	1646	0.015	0.975	21.9995	22:0	0.37	ECL deviates  0.000	Reference -0.006	
7.7800	58041	0.018	----	22.2647		----			
8.0865	973	0.018	----	22.6889		----			
8.2584	1192	0.018	0.987	22.9267	23:1 w4c	0.27	ECL deviates  0.000		
9.0222	1502	0.016	1.017	23.9992	24:0	0.35	ECL deviates -0.001	Reference -0.004	
9.3860	1237	0.017	----	24.5104		----	> max rt		

ECL Deviation: 0.004                            Reference ECL Shift: 0.005       Number Reference Peaks: 16
Total Response: 506663                         Total Named: 424944
Percent Named: 83.87%                         Total Amount: 435149

(No search libraries specified in method PLFAD1.)
