# Supplementary material for: Microbial regulation of soil carbon properties under nitrogen addition and plant inputs removal
Source: PeerJ. 2019 Jul 17;7:e7343. doi: 10.7717/peerj.7343 (PMC6642627; doi:10.7717/peerj.7343)
Supplement: File S1 — The raw data showed the soil microbial PLFAs files in the year of 2015 and 2016. Each file of rtf. represented the microbial PLFAs for each soil sample. In the Supplemental File, the Excel file named “Numbers” showed the plots names and the related rtf. file names. [file peerj-07-7343-s002.zip › supplementary files/2016/76.rtf]

Volume: DATA            File: E17C203.64A       Samp Ctr: 32                 ID Number: 5049 
Type: Samp                   Bottle: 18                      Method: PLFAD1 
Created: 12/20/2017 11:08:26 PM 
Sample ID: 76 


RT	Response	Ar/Ht	RFact	ECL	Peak Name	Percent	Comment1	Comment2	
0.7653	1.671E+9	0.016	----	7.7061	SOLVENT PEAK	----	< min rt		
0.8801	1096	0.016	----	8.3585		----	< min rt		
1.5875	853	0.019	0.973	12.0093	12:0	0.08	ECL deviates  0.009	Reference  0.016	
1.7749	1118	0.015	0.999	12.6072	13:0 iso	0.11	ECL deviates -0.005	Reference  0.000	
1.8093	767	0.014	1.002	12.7170	13:0 anteiso	0.08	ECL deviates  0.008	Reference  0.013	
1.8944	1056	0.022	1.011	12.9887	13:0	0.11	ECL deviates -0.011	Reference -0.007	
1.9926	1043	0.018	----	13.2371		----			
2.1407	9782	0.015	1.026	13.6097	14:0 iso	1.02	ECL deviates -0.004	Reference -0.001	
2.2956	10281	0.017	1.032	13.9989	14:0	1.08	ECL deviates -0.001	Reference  0.002	
2.3590	1341	0.011	----	14.1299	14:0 iso 3OH	----	ECL deviates  0.005		
2.4560	712	0.014	----	14.3302		----			
2.5093	12121	0.018	1.037	14.4403	15:1 iso w6c	1.28	ECL deviates  0.001		
2.5312	1557	0.011	1.037	14.4855	15:4 w3c	0.16	ECL deviates -0.005		
2.5536	1929	0.014	1.038	14.5318	15:1 anteiso w9c	0.20	ECL deviates  0.002		
2.5943	56116	0.015	1.038	14.6157	15:0 iso	5.95	ECL deviates -0.001	Reference  0.000	
2.6403	35636	0.016	1.039	14.7108	15:0 anteiso	3.78	ECL deviates  0.000	Reference  0.001	
2.7113	1329	0.019	1.039	14.8575	15:1 w6c	0.14	ECL deviates -0.003		
2.7809	5699	0.016	1.040	15.0009	15:0	0.60	ECL deviates  0.001	Reference  0.002	
2.8114	1785	0.016	----	15.0548		----			
2.9148	1707	0.015	----	15.2375		----			
3.0334	8174	0.022	1.039	15.4471	15:0 DMA	0.87	ECL deviates -0.003		
3.1034	12142	0.016	1.039	15.5708	16:3 w6c	1.29	ECL deviates -0.005		
3.1319	21563	0.017	1.038	15.6211	16:0 iso	2.29	ECL deviates  0.001	Reference  0.002	
3.1860	2700	0.016	1.038	15.7167	16:0 anteiso	0.29	ECL deviates  0.002	Reference  0.002	
3.2167	9972	0.018	1.038	15.7710	16:1 w9c	1.06	ECL deviates -0.004		
3.2465	80541	0.018	1.037	15.8237	16:1 w7c	8.53	ECL deviates -0.001		
3.2980	24256	0.016	1.037	15.9147	16:1 w5c	2.57	ECL deviates  0.004		
3.3479	103593	0.016	1.036	16.0025	16:0	10.95	ECL deviates  0.003	Reference  0.002	
3.3774	5063	0.020	----	16.0491		----			
3.4356	740	0.015	1.035	16.1410	16:2 DMA	0.08	ECL deviates  0.003		
3.4738	1274	0.023	----	16.2014		----			
3.6165	51174	0.019	1.032	16.4268	16:0 10-methyl	5.39	ECL deviates  0.007		
3.6615	86625	0.017	1.031	16.4979	17:1 iso w9c	9.12	ECL deviates  0.000		
3.7422	13794	0.016	1.030	16.6253	17:0 iso	1.45	ECL deviates  0.002	Reference  0.001	
3.8028	15843	0.018	1.029	16.7210	17:0 anteiso	1.66	ECL deviates  0.001		
3.8527	6673	0.016	1.028	16.7998	17:1 w8c	0.70	ECL deviates  0.003		
3.9146	34217	0.019	1.027	16.8976	17:0 cyclo w7c	3.59	ECL deviates  0.004		
3.9819	4778	0.017	1.025	17.0036	17:0	0.50	ECL deviates  0.004	Reference  0.002	
4.0083	6074	0.017	1.025	17.0421	17:1 w7c 10-methyl	0.64	ECL deviates -0.001		
4.0541	1462	0.015	----	17.1090		----			
4.1436	1050	0.013	1.022	17.2396	16:0 2OH	0.11	ECL deviates -0.001		
4.2581	6644	0.017	1.020	17.4067	17:0 10-methyl	0.69	ECL deviates  0.000		
4.3215	2646	0.025	----	17.4992		----			
4.3773	2491	0.015	1.017	17.5806	18:3 w6c	0.26	ECL deviates  0.001		
4.4032	5382	0.023	1.016	17.6185	18:0 iso	0.56	ECL deviates -0.008	Reference -0.010	
4.4773	18373	0.016	1.015	17.7265	18:2 w6c	1.90	ECL deviates -0.001		
4.5099	46476	0.017	1.014	17.7742	18:1 w9c	4.81	ECL deviates  0.000		
4.5466	86332	0.018	1.013	17.8277	18:1 w7c	8.93	ECL deviates  0.001		
4.6058	14595	0.021	1.012	17.9141	18:1 w5c	1.51	ECL deviates -0.009		
4.6656	16739	0.018	1.010	18.0014	18:0	1.73	ECL deviates  0.001	Reference -0.001	
4.7253	8268	0.019	1.009	18.0848	18:1 w7c 10-methyl	0.85	ECL deviates  0.000		
4.7828	2358	0.027	1.008	18.1650	18:2 DMA	0.24	ECL deviates  0.005		
4.8298	1596	0.025	1.007	18.2307	18:1 w9c DMA	0.16	ECL deviates -0.006		
4.9453	23920	0.020	1.004	18.3920	18:0 10-methyl	2.45	ECL deviates -0.003		
5.0623	4688	0.019	1.002	18.5554	19:3 w6c	0.48	ECL deviates -0.005		
5.1401	1266	0.023	1.000	18.6641	19:3 w3c	0.13	ECL deviates  0.006		
5.1962	3054	0.023	----	18.7425		----			
5.2479	3126	0.020	0.998	18.8145	19:1 w8c	0.32	ECL deviates  0.004		
5.3125	28479	0.019	0.996	18.9048	19:0 cyclo w7c	2.90	ECL deviates -0.005		
5.3840	58048	0.017	----	19.0046	19:0	----	ECL deviates  0.005		
5.4498	1213	0.019	0.993	19.0940	19:1 w7c 10-methyl	0.12	ECL deviates -0.009		
5.5355	4575	0.020	----	19.2104		----			
5.5808	1216	0.016	----	19.2720		----			
5.6164	2860	0.018	0.990	19.3203	19:0 cyclo 9,10 DMA	0.29	ECL deviates -0.003		
5.6514	4441	0.018	----	19.3679		----			
5.6742	2812	0.017	0.989	19.3988	20:4 w6c	0.28	ECL deviates -0.005		
5.7296	1298	0.018	0.988	19.4741	20:5 w3c	0.13	ECL deviates -0.008		
5.7922	2679	0.028	0.987	19.5591	20:3 w6c	0.27	ECL deviates -0.007		
5.8258	3591	0.027	----	19.6047		----			
5.9475	10515	0.043	0.984	19.7700	20:1 w9c	----	> max ar/ht		
6.1182	5064	0.022	0.981	20.0019	20:0	0.51	ECL deviates  0.002	Reference -0.002	
6.2245	956	0.017	----	20.1460		----			
6.2590	1735	0.017	----	20.1928		----			
6.3731	3817	0.016	----	20.3475		----			
6.4010	22341	0.022	0.978	20.3853	20:0 10-methyl	2.23	ECL deviates -0.012		
6.5041	2050	0.024	----	20.5250		----			
6.5713	4132	0.026	----	20.6161		----			
6.6504	3417	0.024	----	20.7233		----			
6.7042	2549	0.018	0.975	20.7963	21:1 w8c	0.25	ECL deviates -0.002		
6.7666	1919	0.020	----	20.8809		----			
6.8222	3367	0.016	0.974	20.9562	21:1 w3c	0.33	ECL deviates  0.002		
6.8788	4158	0.023	----	21.0331		----			
6.9396	750	0.017	----	21.1157		----			
6.9694	1844	0.027	----	21.1563		----			
7.0607	1571	0.025	----	21.2806		----			
7.2014	1727	0.033	0.973	21.4719	22:5 w3c	0.17	ECL deviates  0.004		
7.3150	4850	0.037	0.973	21.6264	22:0 iso	----	> max ar/ht		
7.3404	4585	0.022	----	21.6609		----			
7.3658	2635	0.017	----	21.6955		----			
7.4191	2563	0.023	0.974	21.7680	22:1 w9c	0.25	ECL deviates -0.005		
7.4628	9406	0.023	----	21.8274		----			
7.5424	1758	0.017	0.975	21.9357	22:1 w3c	0.17	ECL deviates -0.011		
7.5901	4641	0.018	0.975	22.0006	22:0	0.46	ECL deviates  0.001	Reference -0.003	
7.7792	87223	0.017	----	22.2624		----			
8.0882	2456	0.017	----	22.6903		----			
8.1554	1995	0.019	----	22.7835		----			
8.2164	1258	0.021	----	22.8678		----			
8.2574	2085	0.017	0.987	22.9247	23:1 w4c	0.21	ECL deviates -0.002		
8.3129	1434	0.017	0.988	23.0015	23:0	0.14	ECL deviates  0.002	Reference -0.002	
8.3522	1054	0.020	----	23.0567		----			
8.5251	1241	0.020	----	23.3000		----			
8.7893	6031	0.030	----	23.6718		----			
8.8345	6399	0.024	----	23.7354		----			
8.9413	3099	0.018	----	23.8856		----			
9.0210	5552	0.020	1.017	23.9976	24:0	0.58	ECL deviates -0.002	Reference -0.006	
9.1812	925	0.017	----	24.2231		----	> max rt		
9.2081	594	0.014	----	24.2609		----	> max rt		
9.3854	7462	0.020	----	24.5102		----	> max rt		
9.4891	1647	0.016	----	24.6562		----	> max rt		

ECL Deviation: 0.005                            Reference ECL Shift: 0.006       Number Reference Peaks: 20
Total Response: 1158494                       Total Named: 956831
Percent Named: 82.59%                         Total Amount: 994938
Profile Comment:   Review report comments.

(No search libraries specified in method PLFAD1.)
