# Supplementary material for: Microbial regulation of soil carbon properties under nitrogen addition and plant inputs removal
Source: PeerJ. 2019 Jul 17;7:e7343. doi: 10.7717/peerj.7343 (PMC6642627; doi:10.7717/peerj.7343)
Supplement: File S1 — The raw data showed the soil microbial PLFAs files in the year of 2015 and 2016. Each file of rtf. represented the microbial PLFAs for each soil sample. In the Supplemental File, the Excel file named “Numbers” showed the plots names and the related rtf. file names. [file peerj-07-7343-s002.zip › supplementary files/2016/77.rtf]

Volume: DATA            File: E17C203.64A       Samp Ctr: 33                 ID Number: 5050 
Type: Samp                   Bottle: 19                      Method: PLFAD1 
Created: 12/20/2017 11:37:06 PM 
Sample ID: 77 


RT	Response	Ar/Ht	RFact	ECL	Peak Name	Percent	Comment1	Comment2	
0.7651	1.676E+9	0.016	----	7.7109	SOLVENT PEAK	----	< min rt		
0.9504	479	0.010	----	8.7634		----	< min rt		
1.3843	534	0.013	----	11.1643		----			
1.5841	1170	0.019	0.973	12.0023	12:0	0.11	ECL deviates  0.002	Reference  0.005	
1.6481	668	0.013	----	12.2066		----			
1.7721	925	0.013	0.999	12.6024	13:0 iso	0.09	ECL deviates -0.010	Reference -0.009	
1.8100	711	0.012	1.002	12.7233	13:0 anteiso	0.07	ECL deviates  0.014	Reference  0.015	
1.9897	1445	0.021	----	13.2339		----			
2.1390	8801	0.016	1.026	13.6093	14:0 iso	0.88	ECL deviates -0.005	Reference -0.006	
2.2667	884	0.013	----	13.9307		----			
2.2933	11479	0.014	1.032	13.9974	14:0	1.16	ECL deviates -0.003	Reference -0.004	
2.3562	2388	0.014	----	14.1282	14:0 iso 3OH	----	ECL deviates  0.003		
2.4546	986	0.017	----	14.3314		----			
2.5073	14970	0.018	1.037	14.4402	15:1 iso w6c	1.51	ECL deviates  0.001		
2.5506	2256	0.014	1.038	14.5296	15:1 anteiso w9c	0.23	ECL deviates  0.000		
2.5919	58417	0.015	1.038	14.6151	15:0 iso	5.92	ECL deviates -0.002	Reference -0.004	
2.6382	35606	0.015	1.039	14.7105	15:0 anteiso	3.61	ECL deviates -0.001	Reference -0.003	
2.7165	2397	0.019	----	14.8723		----			
2.7783	6062	0.016	1.040	14.9998	15:0	0.61	ECL deviates  0.000	Reference -0.003	
2.8086	1545	0.017	----	15.0539		----			
2.9104	1946	0.017	----	15.2338		----			
3.0038	1061	0.011	1.039	15.3989	16:1 w7c alcohol	0.11	ECL deviates  0.002		
3.0292	7292	0.019	1.039	15.4438	15:0 DMA	0.74	ECL deviates -0.007		
3.1005	14546	0.016	1.039	15.5698	16:3 w6c	1.47	ECL deviates -0.006		
3.1289	23767	0.015	1.038	15.6198	16:0 iso	2.41	ECL deviates  0.000	Reference -0.004	
3.1828	2611	0.015	1.038	15.7151	16:0 anteiso	0.26	ECL deviates  0.000	Reference -0.004	
3.2140	9249	0.017	1.038	15.7703	16:1 w9c	0.94	ECL deviates -0.005		
3.2433	67177	0.017	1.037	15.8220	16:1 w7c	6.80	ECL deviates -0.002		
3.2952	19927	0.016	1.037	15.9137	16:1 w5c	2.02	ECL deviates  0.003		
3.3164	4262	0.011	1.036	15.9513	16:1 w3c	0.43	ECL deviates -0.001		
3.3451	127180	0.015	1.036	16.0020	16:0	12.85	ECL deviates  0.002	Reference -0.002	
3.3742	2853	0.016	----	16.0482		----			
3.4325	979	0.014	1.035	16.1402	16:2 DMA	0.10	ECL deviates  0.002		
3.6127	46306	0.018	1.032	16.4247	16:0 10-methyl	4.66	ECL deviates  0.005		
3.6582	102925	0.017	1.031	16.4966	17:1 iso w9c	10.36	ECL deviates -0.001		
3.7395	15056	0.016	1.030	16.6250	17:0 iso	1.51	ECL deviates  0.001	Reference -0.004	
3.7998	17309	0.017	1.029	16.7202	17:0 anteiso	1.74	ECL deviates  0.000		
3.8486	7626	0.017	1.028	16.7973	17:1 w8c	0.76	ECL deviates  0.000		
3.9112	28793	0.018	1.027	16.8961	17:0 cyclo w7c	2.88	ECL deviates  0.002		
3.9782	5713	0.017	1.025	17.0018	17:0	0.57	ECL deviates  0.002	Reference -0.004	
4.0053	4601	0.017	1.025	17.0416	17:1 w7c 10-methyl	0.46	ECL deviates -0.002		
4.0516	1095	0.014	----	17.1092		----			
4.1402	1232	0.015	1.022	17.2386	16:0 2OH	0.12	ECL deviates -0.002		
4.2558	7340	0.017	1.020	17.4072	17:0 10-methyl	0.73	ECL deviates  0.000		
4.3191	3341	0.028	----	17.4995		----			
4.3757	3829	0.017	1.017	17.5821	18:3 w6c	0.38	ECL deviates  0.002		
4.4004	4347	0.019	1.016	17.6182	18:0 iso	0.43	ECL deviates -0.008	Reference -0.014	
4.4339	1312	0.015	----	17.6670		----			
4.4752	26499	0.018	1.015	17.7272	18:2 w6c	2.62	ECL deviates  0.000		
4.5069	50885	0.017	1.014	17.7736	18:1 w9c	5.03	ECL deviates -0.001		
4.5438	83976	0.019	1.013	17.8273	18:1 w7c	8.30	ECL deviates  0.000		
4.6013	25979	0.021	----	17.9113		----			
4.6622	21543	0.018	1.010	18.0000	18:0	2.12	ECL deviates  0.000	Reference -0.006	
4.7229	8277	0.017	1.009	18.0850	18:1 w7c 10-methyl	0.81	ECL deviates  0.000		
4.7821	2530	0.022	1.008	18.1677	18:2 DMA	0.25	ECL deviates  0.008		
4.8285	2201	0.028	1.007	18.2325	18:1 w9c DMA	0.22	ECL deviates -0.005		
4.9429	26062	0.019	1.004	18.3922	18:0 10-methyl	2.55	ECL deviates -0.003		
5.0161	894	0.019	1.003	18.4945	19:4 w6c	0.09	ECL deviates  0.009		
5.0612	3532	0.021	1.002	18.5574	19:3 w6c	0.35	ECL deviates -0.003		
5.1404	1439	0.024	1.000	18.6679	19:3 w3c	0.14	ECL deviates  0.010		
5.1895	3743	0.025	0.999	18.7366	19:0 anteiso	0.36	ECL deviates  0.009	Reference  0.003	
5.2440	4081	0.018	0.998	18.8127	19:1 w8c	0.40	ECL deviates  0.002		
5.2789	4814	0.019	0.997	18.8613	19:1 w6c	0.47	ECL deviates  0.009		
5.3126	19755	0.016	0.996	18.9083	19:0 cyclo w7c	1.92	ECL deviates -0.002		
5.3803	60891	0.019	----	19.0029	19:0	----	ECL deviates  0.003		
5.4962	1402	0.014	----	19.1604		----			
5.5311	906	0.015	----	19.2078		----			
5.5782	2451	0.015	----	19.2717		----			
5.6153	970	0.013	0.990	19.3221	19:0 cyclo 9,10 DMA	0.09	ECL deviates -0.001		
5.6474	6980	0.019	----	19.3657		----			
5.6701	2535	0.014	0.989	19.3966	20:4 w6c	0.24	ECL deviates -0.007		
5.7268	1260	0.019	0.988	19.4735	20:5 w3c	0.12	ECL deviates -0.009		
5.7966	2080	0.030	0.987	19.5683	20:3 w6c	0.20	ECL deviates  0.002		
5.8226	2168	0.019	----	19.6036		----			
5.9462	6178	0.027	0.984	19.7714	20:1 w9c	0.59	ECL deviates -0.001		
5.9676	1779	0.019	0.984	19.8005	20:1 w8c	0.17	ECL deviates -0.013		
6.1130	5956	0.021	0.981	19.9978	20:0	0.57	ECL deviates -0.002	Reference -0.009	
6.2222	589	0.013	----	20.1459		----			
6.2552	1148	0.016	----	20.1905		----			
6.3715	3374	0.016	----	20.3482		----			
6.4014	20015	0.019	0.978	20.3887	20:0 10-methyl	1.91	ECL deviates -0.008		
6.5280	1187	0.023	----	20.5602		----			
6.5722	1757	0.017	----	20.6202		----			
6.5959	1024	0.015	0.976	20.6522	21:3 w3c	0.10	ECL deviates -0.001		
6.6506	3739	0.023	----	20.7263		----			
6.7031	3084	0.017	0.975	20.7975	21:1 w8c	0.29	ECL deviates -0.001		
6.7643	3311	0.019	----	20.8804		----			
6.8191	5708	0.018	0.974	20.9547	21:1 w3c	0.54	ECL deviates  0.001		
6.8730	2528	0.031	----	21.0278		----			
7.0612	2059	0.017	----	21.2836		----			
7.3121	4877	0.036	0.973	21.6248	22:0 iso	----	> max ar/ht		
7.3660	1594	0.018	----	21.6980		----			
7.4581	5540	0.028	0.974	21.8232	22:1 w8c	0.53	ECL deviates  0.010		
7.5434	1600	0.017	0.975	21.9392	22:1 w3c	0.15	ECL deviates -0.008		
7.5887	8518	0.017	0.975	22.0008	22:0	0.81	ECL deviates  0.001	Reference -0.005	
7.7787	106111	0.018	----	22.2637		----			
8.0852	1718	0.017	----	22.6878		----			
8.1516	1544	0.017	----	22.7798		----			
8.2144	866	0.017	----	22.8668		----			
8.2557	1745	0.015	0.987	22.9239	23:1 w4c	0.17	ECL deviates -0.003		
8.3101	2276	0.016	0.988	22.9991	23:0	0.22	ECL deviates -0.001	Reference -0.006	
8.5232	1966	0.019	----	23.2987		----			
8.6216	1808	0.019	----	23.4371		----			
8.7937	3776	0.035	----	23.6790		----	> max ar/ht		
8.8327	2266	0.023	----	23.7338		----			
8.9400	1769	0.018	----	23.8847		----			
8.9686	687	0.015	----	23.9249		----			
9.0217	7387	0.017	1.017	23.9995	24:0	0.73	ECL deviates -0.001	Reference -0.005	
9.3846	7473	0.018	----	24.5096		----	> max rt		
9.4877	1017	0.016	----	24.6544		----	> max rt		

ECL Deviation: 0.005                            Reference ECL Shift: 0.007       Number Reference Peaks: 20
Total Response: 1208976                       Total Named: 1001409
Percent Named: 82.83%                         Total Amount: 1029941
Profile Comment:   Review report comments.

(No search libraries specified in method PLFAD1.)
