# Supplementary material for: Microbial regulation of soil carbon properties under nitrogen addition and plant inputs removal
Source: PeerJ. 2019 Jul 17;7:e7343. doi: 10.7717/peerj.7343 (PMC6642627; doi:10.7717/peerj.7343)
Supplement: File S1 — The raw data showed the soil microbial PLFAs files in the year of 2015 and 2016. Each file of rtf. represented the microbial PLFAs for each soil sample. In the Supplemental File, the Excel file named “Numbers” showed the plots names and the related rtf. file names. [file peerj-07-7343-s002.zip › supplementary files/2016/78.rtf]

Volume: DATA            File: E17C203.64A       Samp Ctr: 34                 ID Number: 5051 
Type: Samp                   Bottle: 20                      Method: PLFAD1 
Created: 12/21/2017 12:05:37 AM 
Sample ID: 78 


RT	Response	Ar/Ht	RFact	ECL	Peak Name	Percent	Comment1	Comment2	
0.7654	1.66E+9	0.016	----	7.7166	SOLVENT PEAK	----	< min rt		
0.9494	478	0.011	----	8.7618		----	< min rt		
1.9891	932	0.020	----	13.2347		----			
2.1386	4978	0.018	1.026	13.6106	14:0 iso	0.88	ECL deviates -0.003	Reference -0.007	
2.1824	766	0.016	1.028	13.7208	14:0 anteiso	0.14	ECL deviates  0.005	Reference  0.002	
2.2932	4925	0.016	1.032	13.9993	14:0	0.88	ECL deviates -0.001	Reference -0.004	
2.3559	962	0.012	----	14.1295	14:0 iso 3OH	----	ECL deviates  0.005		
2.5064	7325	0.019	1.037	14.4403	15:1 iso w6c	1.31	ECL deviates  0.001		
2.5505	1218	0.015	1.038	14.5314	15:1 anteiso w9c	0.22	ECL deviates  0.001		
2.5914	29524	0.015	1.038	14.6156	15:0 iso	5.30	ECL deviates -0.001	Reference -0.006	
2.6370	20036	0.016	1.039	14.7099	15:0 anteiso	3.60	ECL deviates -0.001	Reference -0.006	
2.7053	1167	0.022	1.039	14.8508	15:1 w6c	0.21	ECL deviates -0.009		
2.7773	3373	0.015	1.040	14.9996	15:0	0.61	ECL deviates  0.000	Reference -0.005	
2.8087	993	0.016	----	15.0558		----			
2.9109	1012	0.019	----	15.2363		----			
3.0292	4857	0.020	1.039	15.4454	15:0 DMA	0.87	ECL deviates -0.005		
3.0995	12138	0.016	1.039	15.5694	16:3 w6c	2.18	ECL deviates -0.006		
3.1280	13432	0.016	1.038	15.6199	16:0 iso	2.41	ECL deviates  0.000	Reference -0.005	
3.1827	1730	0.014	1.038	15.7163	16:0 anteiso	0.31	ECL deviates  0.001	Reference -0.004	
3.2142	4807	0.016	1.038	15.7720	16:1 w9c	0.86	ECL deviates -0.003		
3.2430	37121	0.018	1.037	15.8230	16:1 w7c	6.66	ECL deviates -0.001		
3.2950	12334	0.017	1.037	15.9148	16:1 w5c	2.21	ECL deviates  0.004		
3.3447	64139	0.016	1.036	16.0025	16:0	11.50	ECL deviates  0.002	Reference -0.003	
3.3747	2355	0.016	----	16.0503		----			
3.6124	25720	0.017	1.032	16.4255	16:0 10-methyl	4.59	ECL deviates  0.005		
3.6581	72548	0.017	1.031	16.4977	17:1 iso w9c	12.95	ECL deviates  0.000		
3.7389	7986	0.016	1.030	16.6252	17:0 iso	1.42	ECL deviates  0.001	Reference -0.005	
3.8002	10173	0.017	1.029	16.7220	17:0 anteiso	1.81	ECL deviates  0.002		
3.8483	4621	0.017	1.028	16.7979	17:1 w8c	0.82	ECL deviates  0.001		
3.9114	19253	0.018	1.027	16.8974	17:0 cyclo w7c	3.42	ECL deviates  0.004		
3.9771	3158	0.016	1.025	17.0012	17:0	0.56	ECL deviates  0.001	Reference -0.005	
4.0039	3558	0.017	1.025	17.0407	17:1 w7c 10-methyl	0.63	ECL deviates -0.003		
4.0502	785	0.015	----	17.1083		----			
4.1409	892	0.014	1.022	17.2406	16:0 2OH	0.16	ECL deviates  0.000		
4.2549	4513	0.018	1.020	17.4069	17:0 10-methyl	0.80	ECL deviates  0.000		
4.3164	1570	0.023	----	17.4966		----			
4.3742	2345	0.017	1.017	17.5810	18:3 w6c	0.41	ECL deviates  0.001		
4.4007	2246	0.018	1.016	17.6196	18:0 iso	0.40	ECL deviates -0.007	Reference -0.014	
4.4291	809	0.015	----	17.6611		----			
4.4734	18283	0.016	1.015	17.7256	18:2 w6c	3.21	ECL deviates -0.001		
4.5065	27927	0.018	1.014	17.7740	18:1 w9c	4.90	ECL deviates  0.000		
4.5420	45206	0.019	1.013	17.8257	18:1 w7c	7.93	ECL deviates -0.001		
4.6010	7529	0.022	----	17.9118		----			
4.6621	11274	0.019	1.010	18.0009	18:0	1.97	ECL deviates  0.001	Reference -0.006	
4.7219	4094	0.015	1.009	18.0847	18:1 w7c 10-methyl	0.71	ECL deviates  0.000		
4.7803	1020	0.021	1.008	18.1662	18:2 DMA	0.18	ECL deviates  0.006		
4.9411	15160	0.020	1.004	18.3908	18:0 10-methyl	2.63	ECL deviates -0.004		
5.0595	2568	0.020	1.002	18.5561	19:3 w6c	0.45	ECL deviates -0.004		
5.1988	1828	0.027	----	18.7505		----			
5.2432	1916	0.019	0.998	18.8124	19:1 w8c	0.33	ECL deviates  0.002		
5.2847	1973	0.014	0.997	18.8704	19:0 cyclo w9c	0.34	ECL deviates -0.002		
5.3115	11915	0.019	0.996	18.9078	19:0 cyclo w7c	2.05	ECL deviates -0.002		
5.3813	57011	0.017	----	19.0052	19:0	----	ECL deviates  0.005		
5.5320	700	0.015	----	19.2099		----			
5.6478	3937	0.031	----	19.3672		----			
5.7932	731	0.016	0.987	19.5647	20:3 w6c	0.12	ECL deviates -0.002		
5.8192	1358	0.019	----	19.6000		----			
5.9454	3652	0.030	0.984	19.7713	20:1 w9c	0.62	ECL deviates -0.001		
5.9719	768	0.016	0.984	19.8073	20:1 w8c	0.13	ECL deviates -0.006		
6.1157	3039	0.020	0.981	20.0025	20:0	0.52	ECL deviates  0.003	Reference -0.005	
6.2560	794	0.015	----	20.1927		----			
6.3728	2933	0.016	----	20.3510		----			
6.3991	17793	0.018	0.978	20.3867	20:0 10-methyl	3.01	ECL deviates -0.010		
6.4373	772	0.018	----	20.4384		----			
6.5695	1772	0.020	----	20.6176		----			
6.6501	2453	0.022	----	20.7269		----			
6.7030	1463	0.015	0.975	20.7985	21:1 w8c	0.25	ECL deviates  0.000		
6.7645	953	0.020	----	20.8818		----			
6.8195	2543	0.018	0.974	20.9563	21:1 w3c	0.43	ECL deviates  0.002		
6.8743	1454	0.024	----	21.0308		----			
7.3070	2125	0.038	0.973	21.6191	22:0 iso	----	> max ar/ht		
7.4545	1903	0.019	0.974	21.8198	22:1 w8c	0.32	ECL deviates  0.006		
7.5411	758	0.015	0.975	21.9376	22:1 w3c	0.13	ECL deviates -0.009		
7.5853	3550	0.017	0.975	21.9976	22:0	0.60	ECL deviates -0.002	Reference -0.010	
7.7774	82047	0.021	----	22.2635		----			
8.0863	1277	0.018	----	22.6911		----			
8.2552	913	0.015	0.987	22.9249	23:1 w4c	0.16	ECL deviates -0.002		
8.3112	1228	0.018	0.988	23.0024	23:0	0.21	ECL deviates  0.002	Reference -0.005	
8.7894	2377	0.033	----	23.6751		----			
8.8319	912	0.020	----	23.7349		----			
8.9374	1364	0.018	----	23.8832		----			
9.0198	3668	0.017	1.017	23.9991	24:0	0.65	ECL deviates -0.001	Reference -0.007	
9.3853	4721	0.018	----	24.5131		----	> max rt		
9.4870	680	0.014	----	24.6562		----	> max rt		

ECL Deviation: 0.004                            Reference ECL Shift: 0.006       Number Reference Peaks: 17
Total Response: 689267                         Total Named: 564226
Percent Named: 81.86%                         Total Amount: 579896
Profile Comment:   Review report comments.

(No search libraries specified in method PLFAD1.)
