# Supplementary material for: Microbial regulation of soil carbon properties under nitrogen addition and plant inputs removal
Source: PeerJ. 2019 Jul 17;7:e7343. doi: 10.7717/peerj.7343 (PMC6642627; doi:10.7717/peerj.7343)
Supplement: File S1 — The raw data showed the soil microbial PLFAs files in the year of 2015 and 2016. Each file of rtf. represented the microbial PLFAs for each soil sample. In the Supplemental File, the Excel file named “Numbers” showed the plots names and the related rtf. file names. [file peerj-07-7343-s002.zip › supplementary files/2016/79.rtf]

Volume: DATA            File: E17C203.64A       Samp Ctr: 35                 ID Number: 5052 
Type: Samp                   Bottle: 21                      Method: PLFAD1 
Created: 12/21/2017 12:33:59 AM 
Sample ID: 79 


RT	Response	Ar/Ht	RFact	ECL	Peak Name	Percent	Comment1	Comment2	
0.7658	1.665E+9	0.016	----	7.7134	SOLVENT PEAK	----	< min rt		
0.9509	451	0.010	----	8.7649		----	< min rt		
1.7730	765	0.014	0.999	12.6035	13:0 iso	0.08	ECL deviates -0.009	Reference -0.006	
1.8089	528	0.013	1.002	12.7182	13:0 anteiso	0.06	ECL deviates  0.009	Reference  0.011	
1.9911	1342	0.020	----	13.2356		----			
2.1398	8034	0.018	1.026	13.6096	14:0 iso	0.87	ECL deviates -0.004	Reference -0.004	
2.1852	738	0.013	1.028	13.7239	14:0 anteiso	0.08	ECL deviates  0.008	Reference  0.009	
2.2947	9127	0.016	1.032	13.9993	14:0	1.00	ECL deviates -0.001	Reference  0.000	
2.3571	1700	0.013	----	14.1281	14:0 iso 3OH	----	ECL deviates  0.003		
2.4553	597	0.015	----	14.3309		----			
2.5076	9885	0.019	1.037	14.4389	15:1 iso w6c	1.08	ECL deviates  0.000		
2.5291	1430	0.012	1.037	14.4833	15:4 w3c	0.16	ECL deviates -0.007		
2.5515	1494	0.013	1.038	14.5297	15:1 anteiso w9c	0.16	ECL deviates  0.000		
2.5925	50136	0.015	1.038	14.6144	15:0 iso	5.51	ECL deviates -0.003	Reference -0.003	
2.6384	28895	0.015	1.039	14.7091	15:0 anteiso	3.17	ECL deviates -0.002	Reference -0.003	
2.7081	1024	0.017	1.039	14.8530	15:1 w6c	0.11	ECL deviates -0.007		
2.7790	5195	0.016	1.040	14.9993	15:0	0.57	ECL deviates -0.001	Reference -0.002	
2.8094	1486	0.017	----	15.0535		----			
2.9111	1256	0.015	----	15.2331		----			
3.0043	1374	0.014	1.039	15.3977	16:1 w7c alcohol	0.15	ECL deviates  0.001		
3.0315	6769	0.021	1.039	15.4458	15:0 DMA	0.74	ECL deviates -0.005		
3.1010	13750	0.016	1.039	15.5686	16:3 w6c	1.51	ECL deviates -0.007		
3.1302	20004	0.016	1.038	15.6202	16:0 iso	2.20	ECL deviates  0.001	Reference -0.001	
3.1842	2823	0.016	1.038	15.7157	16:0 anteiso	0.31	ECL deviates  0.001	Reference -0.001	
3.2153	9619	0.018	1.038	15.7705	16:1 w9c	1.06	ECL deviates -0.004		
3.2450	68948	0.018	1.037	15.8230	16:1 w7c	7.57	ECL deviates -0.001		
3.2962	24874	0.017	1.037	15.9135	16:1 w5c	2.73	ECL deviates  0.002		
3.3464	101017	0.015	1.036	16.0022	16:0	11.07	ECL deviates  0.002	Reference  0.000	
3.3755	4398	0.019	----	16.0481		----			
3.4710	2791	0.042	----	16.1989		----	> max ar/ht		
3.6143	47760	0.019	1.032	16.4252	16:0 10-methyl	5.22	ECL deviates  0.005		
3.6591	108127	0.016	1.031	16.4960	17:1 iso w9c	11.80	ECL deviates -0.002		
3.7405	14116	0.016	1.030	16.6245	17:0 iso	1.54	ECL deviates  0.001	Reference -0.002	
3.8007	14834	0.018	1.029	16.7195	17:0 anteiso	1.61	ECL deviates -0.001		
3.8505	7508	0.018	1.028	16.7982	17:1 w8c	0.82	ECL deviates  0.001		
3.9124	27532	0.019	1.027	16.8960	17:0 cyclo w7c	2.99	ECL deviates  0.002		
3.9807	4938	0.018	1.025	17.0038	17:0	0.54	ECL deviates  0.004	Reference  0.000	
4.0066	5922	0.017	1.025	17.0415	17:1 w7c 10-methyl	0.64	ECL deviates -0.002		
4.0519	1325	0.017	----	17.1077		----			
4.1430	1959	0.026	1.022	17.2405	16:0 2OH	0.21	ECL deviates  0.000		
4.2564	6772	0.016	1.020	17.4061	17:0 10-methyl	0.73	ECL deviates -0.001		
4.3159	2610	0.030	----	17.4929		----			
4.3754	2549	0.016	1.017	17.5796	18:3 w6c	0.27	ECL deviates  0.000		
4.4022	4504	0.018	1.016	17.6187	18:0 iso	0.48	ECL deviates -0.008	Reference -0.012	
4.4294	1368	0.016	----	17.6584		----			
4.4759	22079	0.016	1.015	17.7263	18:2 w6c	2.37	ECL deviates -0.001		
4.5081	47611	0.018	1.014	17.7733	18:1 w9c	5.11	ECL deviates -0.001		
4.5449	85829	0.018	1.013	17.8270	18:1 w7c	9.20	ECL deviates  0.000		
4.6033	16460	0.023	----	17.9121		----			
4.6648	17372	0.019	1.010	18.0019	18:0	1.86	ECL deviates  0.002	Reference -0.002	
4.7233	8008	0.018	1.009	18.0836	18:1 w7c 10-methyl	0.85	ECL deviates -0.001		
4.7815	2181	0.027	1.008	18.1649	18:2 DMA	0.23	ECL deviates  0.005		
4.8267	2124	0.027	1.007	18.2280	18:1 w9c DMA	0.23	ECL deviates -0.009		
4.9438	21686	0.021	1.004	18.3915	18:0 10-methyl	2.30	ECL deviates -0.004		
5.0143	795	0.019	1.003	18.4900	19:4 w6c	0.08	ECL deviates  0.005		
5.0603	3793	0.020	1.002	18.5542	19:3 w6c	0.40	ECL deviates -0.006		
5.1405	828	0.015	1.000	18.6662	19:3 w3c	0.09	ECL deviates  0.008		
5.1998	3112	0.026	----	18.7490		----			
5.2446	3265	0.019	0.998	18.8114	19:1 w8c	0.34	ECL deviates  0.001		
5.2876	3427	0.016	0.997	18.8715	19:0 cyclo w9c	0.36	ECL deviates  0.000		
5.3126	24209	0.019	0.996	18.9065	19:0 cyclo w7c	2.55	ECL deviates -0.003		
5.3830	58629	0.018	----	19.0047	19:0	----	ECL deviates  0.005		
5.5348	1600	0.016	----	19.2109		----			
5.5795	1096	0.015	----	19.2716		----			
5.6137	969	0.014	0.990	19.3180	19:0 cyclo 9,10 DMA	0.10	ECL deviates -0.006		
5.6504	3401	0.017	----	19.3678		----			
5.6725	1868	0.015	0.989	19.3978	20:4 w6c	0.20	ECL deviates -0.006		
5.7302	564	0.013	0.988	19.4762	20:5 w3c	0.06	ECL deviates -0.006		
5.7951	1843	0.026	0.987	19.5643	20:3 w6c	0.19	ECL deviates -0.002		
5.8236	1709	0.016	----	19.6031		----			
5.9462	6098	0.027	0.984	19.7696	20:1 w9c	0.63	ECL deviates -0.003		
5.9746	1854	0.023	0.984	19.8080	20:1 w8c	0.19	ECL deviates -0.005		
6.1166	4804	0.019	0.981	20.0009	20:0	0.50	ECL deviates  0.001	Reference -0.004	
6.2259	873	0.016	----	20.1490		----			
6.2574	1335	0.016	----	20.1917		----			
6.3721	3337	0.015	----	20.3472		----			
6.4022	21466	0.018	0.978	20.3880	20:0 10-methyl	2.22	ECL deviates -0.009		
6.5699	1730	0.018	----	20.6153		----			
6.5968	933	0.014	0.976	20.6518	21:3 w3c	0.10	ECL deviates -0.002		
6.6492	3123	0.023	----	20.7228		----			
6.7022	2057	0.015	0.975	20.7946	21:1 w8c	0.21	ECL deviates -0.003		
6.7640	2115	0.021	----	20.8783		----			
6.8199	3598	0.017	0.974	20.9541	21:1 w3c	0.37	ECL deviates  0.000		
6.8744	2094	0.027	----	21.0280		----			
7.0583	1410	0.022	----	21.2781		----			
7.3123	4219	0.042	0.973	21.6235	22:0 iso	----	> max ar/ht		
7.3371	1543	0.015	----	21.6572		----			
7.3632	1726	0.020	----	21.6927		----			
7.4572	6002	0.031	0.974	21.8206	22:1 w8c	0.62	ECL deviates  0.007		
7.5418	1137	0.017	0.975	21.9356	22:1 w3c	0.12	ECL deviates -0.011		
7.5879	5299	0.018	0.975	21.9983	22:0	0.55	ECL deviates -0.002	Reference -0.006	
7.7791	108727	0.019	----	22.2629		----			
8.0849	2052	0.020	----	22.6863		----			
8.1525	997	0.017	----	22.7798		----			
8.2563	2041	0.017	0.987	22.9235	23:1 w4c	0.21	ECL deviates -0.003		
8.3133	1335	0.016	0.988	23.0024	23:0	0.14	ECL deviates  0.002	Reference -0.002	
8.5242	1247	0.016	----	23.2990		----			
8.7929	3192	0.028	----	23.6768		----			
8.8357	2896	0.025	----	23.7370		----			
8.9403	1568	0.018	----	23.8841		----			
9.0220	5249	0.019	1.017	23.9991	24:0	0.56	ECL deviates -0.001	Reference -0.004	
9.3880	4798	0.018	----	24.5138		----	> max rt		
9.4886	955	0.016	----	24.6552		----	> max rt		

ECL Deviation: 0.004                            Reference ECL Shift: 0.005       Number Reference Peaks: 19
Total Response: 1112007                       Total Named: 923276
Percent Named: 83.03%                         Total Amount: 949394
Profile Comment:   Review report comments.

(No search libraries specified in method PLFAD1.)
