# Supplementary material for: Microbial regulation of soil carbon properties under nitrogen addition and plant inputs removal
Source: PeerJ. 2019 Jul 17;7:e7343. doi: 10.7717/peerj.7343 (PMC6642627; doi:10.7717/peerj.7343)
Supplement: File S1 — The raw data showed the soil microbial PLFAs files in the year of 2015 and 2016. Each file of rtf. represented the microbial PLFAs for each soil sample. In the Supplemental File, the Excel file named “Numbers” showed the plots names and the related rtf. file names. [file peerj-07-7343-s002.zip › supplementary files/2016/80.rtf]

Volume: DATA            File: E17C203.64A       Samp Ctr: 36                 ID Number: 5053 
Type: Samp                   Bottle: 22                      Method: PLFAD1 
Created: 12/21/2017 1:02:31 AM 
Sample ID: 80 


RT	Response	Ar/Ht	RFact	ECL	Peak Name	Percent	Comment1	Comment2	
0.7660	1.646E+9	0.015	----	7.7283	SOLVENT PEAK	----	< min rt		
1.7719	562	0.014	0.999	12.6122	13:0 iso	0.10	ECL deviates  0.000	Reference -0.009	
1.9895	1028	0.021	----	13.2416		----			
2.1383	5132	0.016	1.026	13.6150	14:0 iso	0.98	ECL deviates  0.001	Reference -0.007	
2.2658	602	0.013	----	13.9347		----			
2.2929	4175	0.015	1.032	14.0026	14:0	0.80	ECL deviates  0.003	Reference -0.005	
2.3559	1045	0.013	----	14.1333	14:0 iso 3OH	----	ECL deviates  0.009		
2.4532	643	0.015	----	14.3338		----			
2.5060	4650	0.018	1.037	14.4425	15:1 iso w6c	0.90	ECL deviates  0.003		
2.5302	901	0.013	1.037	14.4925	15:4 w3c	0.17	ECL deviates  0.002		
2.5498	923	0.013	1.038	14.5328	15:1 anteiso w9c	0.18	ECL deviates  0.003		
2.5905	27200	0.014	1.038	14.6167	15:0 iso	5.27	ECL deviates  0.000	Reference -0.007	
2.6366	19127	0.015	1.039	14.7116	15:0 anteiso	3.70	ECL deviates  0.001	Reference -0.006	
2.7772	2879	0.014	1.040	15.0014	15:0	0.56	ECL deviates  0.001	Reference -0.005	
2.8065	1296	0.014	----	15.0537		----			
3.0288	5336	0.021	1.039	15.4458	15:0 DMA	1.03	ECL deviates -0.005		
3.0986	13300	0.016	1.039	15.5688	16:3 w6c	2.58	ECL deviates -0.007		
3.1275	13072	0.016	1.038	15.6198	16:0 iso	2.53	ECL deviates  0.000	Reference -0.006	
3.1816	1703	0.016	1.038	15.7152	16:0 anteiso	0.33	ECL deviates  0.000	Reference -0.006	
3.2114	4946	0.016	1.038	15.7677	16:1 w9c	0.96	ECL deviates -0.007		
3.2421	37373	0.016	1.037	15.8218	16:1 w7c	7.23	ECL deviates -0.003		
3.2935	11177	0.016	1.037	15.9125	16:1 w5c	2.16	ECL deviates  0.001		
3.3428	50665	0.016	1.036	15.9994	16:0	9.79	ECL deviates -0.001	Reference -0.007	
3.3726	3444	0.018	----	16.0471		----			
3.6115	28204	0.019	1.032	16.4237	16:0 10-methyl	5.43	ECL deviates  0.004		
3.6562	77650	0.017	1.031	16.4942	17:1 iso w9c	14.93	ECL deviates -0.004		
3.7376	7122	0.014	1.030	16.6225	17:0 iso	1.37	ECL deviates -0.001	Reference -0.007	
3.7986	8571	0.018	1.029	16.7186	17:0 anteiso	1.64	ECL deviates -0.002		
3.8470	3423	0.016	1.028	16.7950	17:1 w8c	0.66	ECL deviates -0.002		
3.9100	15005	0.018	1.027	16.8942	17:0 cyclo w7c	2.87	ECL deviates  0.001		
3.9758	2279	0.016	1.025	16.9980	17:0	0.44	ECL deviates -0.002	Reference -0.007	
4.0027	4964	0.016	1.025	17.0377	17:1 w7c 10-methyl	0.95	ECL deviates -0.006		
4.0483	891	0.014	----	17.1041		----			
4.2533	3625	0.016	1.020	17.4029	17:0 10-methyl	0.69	ECL deviates -0.004		
4.3138	1554	0.026	----	17.4911		----			
4.3720	2052	0.016	1.017	17.5758	18:3 w6c	0.39	ECL deviates -0.004		
4.3985	2261	0.017	1.016	17.6145	18:0 iso	0.43	ECL deviates -0.012	Reference -0.017	
4.4306	651	0.014	----	17.6612		----			
4.4722	10457	0.017	1.015	17.7219	18:2 w6c	1.98	ECL deviates -0.005		
4.5049	26550	0.017	1.014	17.7695	18:1 w9c	5.02	ECL deviates -0.005		
4.5406	40765	0.017	1.013	17.8215	18:1 w7c	7.70	ECL deviates -0.005		
4.6015	5476	0.022	----	17.9103		----			
4.6600	9752	0.017	1.010	17.9955	18:0	1.84	ECL deviates -0.004	Reference -0.009	
4.7201	3179	0.015	1.009	18.0798	18:1 w7c 10-methyl	0.60	ECL deviates -0.005		
4.9404	14425	0.021	1.004	18.3870	18:0 10-methyl	2.70	ECL deviates -0.008		
5.0588	1847	0.017	1.002	18.5522	19:3 w6c	0.34	ECL deviates -0.008		
5.1923	1898	0.023	----	18.7384		----		Reference  0.007	
5.2423	1721	0.018	0.998	18.8082	19:1 w8c	0.32	ECL deviates -0.003		
5.3093	15585	0.019	0.996	18.9015	19:0 cyclo w7c	2.89	ECL deviates -0.008		
5.3788	56561	0.017	----	18.9985	19:0	----	ECL deviates -0.001		
5.6477	1098	0.021	----	19.3636		----			
5.6683	883	0.015	0.989	19.3916	20:4 w6c	0.16	ECL deviates -0.012		
5.8194	1856	0.027	----	19.5967		----			
5.9016	1002	0.017	----	19.7082		----			
5.9433	2643	0.022	0.984	19.7648	20:1 w9c	0.48	ECL deviates -0.008		
5.9707	1358	0.021	0.984	19.8020	20:1 w8c	0.25	ECL deviates -0.011		
6.1136	2664	0.021	0.981	19.9960	20:0	0.49	ECL deviates -0.004	Reference -0.008	
6.3690	2429	0.015	----	20.3421		----			
6.3995	17435	0.018	0.978	20.3834	20:0 10-methyl	3.18	ECL deviates -0.014		
6.5682	1971	0.025	----	20.6120		----			
6.6479	2614	0.024	----	20.7199		----			
6.6995	1812	0.017	0.975	20.7898	21:1 w8c	0.33	ECL deviates -0.008		
6.7657	729	0.017	----	20.8796		----			
6.8181	3343	0.017	0.974	20.9505	21:1 w3c	0.61	ECL deviates -0.004		
6.8672	1365	0.027	----	21.0171		----		Reference  0.013	
7.3084	1122	0.026	0.973	21.6171	22:0 iso	0.20	ECL deviates -0.001		
7.3630	1013	0.016	----	21.6914		----			
7.4565	1911	0.026	0.974	21.8185	22:1 w8c	0.35	ECL deviates  0.005		
7.5406	930	0.016	0.975	21.9328	22:1 w3c	0.17	ECL deviates -0.014		
7.5853	2680	0.016	0.975	21.9937	22:0	0.49	ECL deviates -0.006	Reference -0.010	
7.7762	84785	0.018	----	22.2579		----			
8.0852	1592	0.017	----	22.6859		----			
8.2547	1529	0.017	0.987	22.9206	23:1 w4c	0.28	ECL deviates -0.006		
8.5215	725	0.016	----	23.2949		----			
8.7943	1639	0.021	----	23.6788		----			
8.9387	825	0.019	----	23.8821		----			
9.0204	2864	0.018	1.017	23.9971	24:0	0.54	ECL deviates -0.003	Reference -0.006	
9.3855	5017	0.017	----	24.5110		----	> max rt		
9.4869	843	0.014	----	24.6538		----	> max rt		

ECL Deviation: 0.006                            Reference ECL Shift: 0.009       Number Reference Peaks: 18
Total Response: 644859                         Total Named: 523733
Percent Named: 81.22%                         Total Amount: 536287

(No search libraries specified in method PLFAD1.)
