# Supplementary material for: Microbial regulation of soil carbon properties under nitrogen addition and plant inputs removal
Source: PeerJ. 2019 Jul 17;7:e7343. doi: 10.7717/peerj.7343 (PMC6642627; doi:10.7717/peerj.7343)
Supplement: File S1 — The raw data showed the soil microbial PLFAs files in the year of 2015 and 2016. Each file of rtf. represented the microbial PLFAs for each soil sample. In the Supplemental File, the Excel file named “Numbers” showed the plots names and the related rtf. file names. [file peerj-07-7343-s002.zip › supplementary files/2016/81.rtf]

Volume: DATA            File: E17C203.64A       Samp Ctr: 37                 ID Number: 5054 
Type: Samp                   Bottle: 23                      Method: PLFAD1 
Created: 12/21/2017 1:30:53 AM 
Sample ID: 81 


RT	Response	Ar/Ht	RFact	ECL	Peak Name	Percent	Comment1	Comment2	
0.7654	1.688E+9	0.016	----	7.7123	SOLVENT PEAK	----	< min rt		
0.9509	487	0.012	----	8.7660		----	< min rt		
1.7730	620	0.013	0.999	12.6049	13:0 iso	0.10	ECL deviates -0.008	Reference -0.006	
1.8103	651	0.013	1.002	12.7238	13:0 anteiso	0.10	ECL deviates  0.014	Reference  0.016	
1.9896	1015	0.016	----	13.2334		----			
2.1389	5227	0.016	1.026	13.6092	14:0 iso	0.84	ECL deviates -0.005	Reference -0.006	
2.2663	710	0.014	----	13.9297		----			
2.2935	5928	0.015	1.032	13.9982	14:0	0.96	ECL deviates -0.002	Reference -0.004	
2.3565	1574	0.013	----	14.1290	14:0 iso 3OH	----	ECL deviates  0.004		
2.4548	629	0.014	----	14.3323		----			
2.5066	7772	0.019	1.037	14.4393	15:1 iso w6c	1.27	ECL deviates  0.000		
2.5276	1031	0.011	1.037	14.4826	15:4 w3c	0.17	ECL deviates -0.008		
2.5521	1111	0.014	1.038	14.5332	15:1 anteiso w9c	0.18	ECL deviates  0.003		
2.5912	35664	0.015	1.038	14.6141	15:0 iso	5.81	ECL deviates -0.003	Reference -0.006	
2.6374	22822	0.015	1.039	14.7095	15:0 anteiso	3.72	ECL deviates -0.001	Reference -0.005	
2.7073	694	0.017	1.039	14.8539	15:1 w6c	0.11	ECL deviates -0.006		
2.7777	3565	0.015	1.040	14.9994	15:0	0.58	ECL deviates -0.001	Reference -0.004	
2.8078	924	0.015	----	15.0533		----			
2.9109	1016	0.021	----	15.2356		----			
3.0037	735	0.012	1.039	15.3997	16:1 w7c alcohol	0.12	ECL deviates  0.003		
3.0290	4421	0.017	1.039	15.4443	15:0 DMA	0.72	ECL deviates -0.006		
3.0993	13594	0.016	1.039	15.5686	16:3 w6c	2.22	ECL deviates -0.007		
3.1281	13694	0.016	1.038	15.6196	16:0 iso	2.23	ECL deviates  0.000	Reference -0.005	
3.1833	1611	0.015	1.038	15.7171	16:0 anteiso	0.26	ECL deviates  0.002	Reference -0.003	
3.2138	6309	0.018	1.038	15.7710	16:1 w9c	1.03	ECL deviates -0.004		
3.2429	36858	0.017	1.037	15.8224	16:1 w7c	6.00	ECL deviates -0.002		
3.2945	11561	0.016	1.037	15.9136	16:1 w5c	1.88	ECL deviates  0.002		
3.3144	1622	0.008	1.036	15.9489	16:1 w3c	0.26	ECL deviates -0.003		
3.3439	66660	0.016	1.036	16.0009	16:0	10.84	ECL deviates  0.001	Reference -0.005	
3.3731	2305	0.016	----	16.0476		----			
3.6119	29537	0.019	1.032	16.4249	16:0 10-methyl	4.79	ECL deviates  0.005		
3.6576	90679	0.017	1.031	16.4970	17:1 iso w9c	14.68	ECL deviates -0.001		
3.7388	8966	0.016	1.030	16.6252	17:0 iso	1.45	ECL deviates  0.001	Reference -0.005	
3.7992	9965	0.018	1.029	16.7208	17:0 anteiso	1.61	ECL deviates  0.000		
3.8482	4325	0.017	1.028	16.7980	17:1 w8c	0.70	ECL deviates  0.001		
3.9102	19090	0.018	1.027	16.8959	17:0 cyclo w7c	3.08	ECL deviates  0.002		
3.9774	3215	0.015	1.025	17.0021	17:0	0.52	ECL deviates  0.002	Reference -0.005	
4.0044	3248	0.016	1.025	17.0419	17:1 w7c 10-methyl	0.52	ECL deviates -0.001		
4.0502	719	0.014	----	17.1087		----			
4.1383	764	0.015	1.022	17.2374	16:0 2OH	0.12	ECL deviates -0.003		
4.2539	4407	0.015	1.020	17.4060	17:0 10-methyl	0.71	ECL deviates -0.001		
4.3173	2187	0.023	----	17.4987		----			
4.3727	2419	0.018	1.017	17.5795	18:3 w6c	0.39	ECL deviates -0.001		
4.4021	2543	0.019	1.016	17.6224	18:0 iso	0.41	ECL deviates -0.004	Reference -0.012	
4.4307	891	0.015	----	17.6642		----			
4.4738	16620	0.017	1.015	17.7270	18:2 w6c	2.65	ECL deviates  0.000		
4.5052	29286	0.018	1.014	17.7728	18:1 w9c	4.66	ECL deviates -0.002		
4.5416	46488	0.017	1.013	17.8260	18:1 w7c	7.39	ECL deviates -0.001		
4.6015	8281	0.020	1.012	17.9134	18:1 w5c	1.32	ECL deviates -0.010		
4.6619	11084	0.019	1.010	18.0016	18:0	1.76	ECL deviates  0.002	Reference -0.006	
4.7209	4512	0.016	1.009	18.0843	18:1 w7c 10-methyl	0.71	ECL deviates -0.001		
4.7804	1086	0.020	1.008	18.1673	18:2 DMA	0.17	ECL deviates  0.007		
4.8139	1269	0.024	----	18.2140		----			
4.9402	13870	0.018	1.004	18.3904	18:0 10-methyl	2.19	ECL deviates -0.005		
4.9663	1591	0.012	1.004	18.4269	18:0 DMA	0.25	ECL deviates -0.003		
5.0575	2688	0.019	1.002	18.5542	19:3 w6c	0.42	ECL deviates -0.006		
5.1983	1805	0.027	----	18.7509		----			
5.2437	2359	0.018	0.998	18.8143	19:1 w8c	0.37	ECL deviates  0.003		
5.3095	16916	0.019	0.996	18.9062	19:0 cyclo w7c	2.65	ECL deviates -0.004		
5.3793	59577	0.018	----	19.0037	19:0	----	ECL deviates  0.004		
5.5340	708	0.018	----	19.2139		----			
5.5742	738	0.013	----	19.2686		----			
5.6464	2200	0.019	----	19.3666		----			
5.6709	1192	0.015	0.989	19.3999	20:4 w6c	0.19	ECL deviates -0.004		
5.7874	890	0.020	0.987	19.5581	20:3 w6c	0.14	ECL deviates -0.008		
5.8204	1231	0.018	----	19.6029		----			
5.9423	3725	0.030	0.984	19.7684	20:1 w9c	0.58	ECL deviates -0.004		
5.9690	1346	0.019	0.984	19.8047	20:1 w8c	0.21	ECL deviates -0.008		
6.1135	3042	0.020	0.981	20.0010	20:0	0.47	ECL deviates  0.001	Reference -0.008	
6.2558	1052	0.017	----	20.1938		----			
6.3686	2737	0.013	----	20.3467		----			
6.3986	20152	0.017	0.978	20.3874	20:0 10-methyl	3.09	ECL deviates -0.010		
6.5673	2014	0.022	----	20.6160		----			
6.6494	2644	0.024	----	20.7273		----			
6.7021	1891	0.016	0.975	20.7987	21:1 w8c	0.29	ECL deviates  0.001		
6.7616	1298	0.019	----	20.8793		----			
6.8204	2841	0.016	0.974	20.9590	21:1 w3c	0.43	ECL deviates  0.005		
7.0575	591	0.014	----	21.2813		----			
7.3079	947	0.022	0.973	21.6217	22:0 iso	0.14	ECL deviates  0.004		
7.3633	785	0.014	----	21.6970		----			
7.4564	2260	0.024	----	21.8236		----			
7.5398	923	0.016	0.975	21.9370	22:1 w3c	0.14	ECL deviates -0.010		
7.5867	3510	0.017	0.975	22.0008	22:0	0.54	ECL deviates  0.001	Reference -0.008	
7.7785	94294	0.017	----	22.2661		----			
8.0855	2088	0.022	----	22.6911		----			
8.2547	1380	0.016	0.987	22.9253	23:1 w4c	0.21	ECL deviates -0.001		
8.3092	1000	0.018	0.988	23.0007	23:0	0.16	ECL deviates  0.001	Reference -0.007	
8.5226	812	0.016	----	23.3007		----			
8.7935	2323	0.025	----	23.6815		----			
8.9398	1002	0.018	----	23.8873		----			
9.0189	3202	0.017	1.017	23.9984	24:0	0.51	ECL deviates -0.002	Reference -0.009	
9.3850	6313	0.018	----	24.5130		----	> max rt		
9.4901	852	0.014	----	24.6608		----	> max rt		

ECL Deviation: 0.005                            Reference ECL Shift: 0.007       Number Reference Peaks: 18
Total Response: 754379                         Total Named: 622132
Percent Named: 82.47%                         Total Amount: 637086

(No search libraries specified in method PLFAD1.)
