# Supplementary material for: Microbial regulation of soil carbon properties under nitrogen addition and plant inputs removal
Source: PeerJ. 2019 Jul 17;7:e7343. doi: 10.7717/peerj.7343 (PMC6642627; doi:10.7717/peerj.7343)
Supplement: File S1 — The raw data showed the soil microbial PLFAs files in the year of 2015 and 2016. Each file of rtf. represented the microbial PLFAs for each soil sample. In the Supplemental File, the Excel file named “Numbers” showed the plots names and the related rtf. file names. [file peerj-07-7343-s002.zip › supplementary files/2016/82.rtf]

Volume: DATA            File: E17C203.64A       Samp Ctr: 38                 ID Number: 5055 
Type: Samp                   Bottle: 24                      Method: PLFAD1 
Created: 12/21/2017 1:59:15 AM 
Sample ID: 82 


RT	Response	Ar/Ht	RFact	ECL	Peak Name	Percent	Comment1	Comment2	
0.7654	1.684E+9	0.016	----	7.7159	SOLVENT PEAK	----	< min rt		
0.9513	523	0.011	----	8.7717		----	< min rt		
1.5856	777	0.018	0.973	12.0084	12:0	0.13	ECL deviates  0.008	Reference  0.010	
1.7737	761	0.015	0.999	12.6089	13:0 iso	0.13	ECL deviates -0.004	Reference -0.004	
1.8074	750	0.014	1.002	12.7164	13:0 anteiso	0.13	ECL deviates  0.007	Reference  0.007	
1.9896	1042	0.017	----	13.2348		----			
2.1389	6932	0.016	1.026	13.6104	14:0 iso	1.26	ECL deviates -0.004	Reference -0.006	
2.1810	524	0.012	1.028	13.7163	14:0 anteiso	0.10	ECL deviates  0.000	Reference -0.002	
2.2650	688	0.014	----	13.9274		----			
2.2926	5452	0.014	1.032	13.9970	14:0	1.00	ECL deviates -0.003	Reference -0.006	
2.3553	1256	0.012	----	14.1275	14:0 iso 3OH	----	ECL deviates  0.003		
2.4549	688	0.014	----	14.3333		----			
2.5050	6265	0.019	1.037	14.4368	15:1 iso w6c	1.15	ECL deviates -0.002		
2.5262	986	0.012	1.037	14.4804	15:4 w3c	0.18	ECL deviates -0.010		
2.5492	979	0.012	1.038	14.5281	15:1 anteiso w9c	0.18	ECL deviates -0.002		
2.5906	30598	0.015	1.038	14.6134	15:0 iso	5.64	ECL deviates -0.004	Reference -0.007	
2.6365	18853	0.015	1.039	14.7082	15:0 anteiso	3.47	ECL deviates -0.003	Reference -0.007	
2.7079	784	0.016	1.039	14.8557	15:1 w6c	0.14	ECL deviates -0.004		
2.7766	3313	0.015	1.040	14.9976	15:0	0.61	ECL deviates -0.002	Reference -0.007	
2.8074	1018	0.016	----	15.0529		----			
3.0013	927	0.014	1.039	15.3956	16:1 w7c alcohol	0.17	ECL deviates -0.001		
3.0285	4455	0.021	1.039	15.4436	15:0 DMA	0.82	ECL deviates -0.007		
3.0986	14802	0.015	1.039	15.5674	16:3 w6c	2.73	ECL deviates -0.008		
3.1275	13261	0.016	1.038	15.6186	16:0 iso	2.44	ECL deviates -0.001	Reference -0.006	
3.1811	1649	0.015	1.038	15.7132	16:0 anteiso	0.30	ECL deviates -0.002	Reference -0.007	
3.2138	5702	0.017	1.038	15.7710	16:1 w9c	1.05	ECL deviates -0.004		
3.2419	35070	0.017	1.037	15.8207	16:1 w7c	6.46	ECL deviates -0.004		
3.2938	11254	0.017	1.037	15.9123	16:1 w5c	2.07	ECL deviates  0.001		
3.3431	55526	0.016	1.036	15.9995	16:0	10.21	ECL deviates -0.001	Reference -0.006	
3.3729	2760	0.019	----	16.0471		----			
3.6111	26122	0.019	1.032	16.4233	16:0 10-methyl	4.78	ECL deviates  0.003		
3.6560	74101	0.017	1.031	16.4940	17:1 iso w9c	13.56	ECL deviates -0.004		
3.7377	6980	0.016	1.030	16.6231	17:0 iso	1.28	ECL deviates -0.001	Reference -0.007	
3.7992	8311	0.015	1.029	16.7201	17:0 anteiso	1.52	ECL deviates  0.000		
3.8471	3796	0.018	1.028	16.7957	17:1 w8c	0.69	ECL deviates -0.001		
3.9104	16162	0.017	1.027	16.8956	17:0 cyclo w7c	2.94	ECL deviates  0.002		
3.9764	2696	0.017	1.025	16.9998	17:0	0.49	ECL deviates  0.000	Reference -0.006	
4.0037	3592	0.016	1.025	17.0402	17:1 w7c 10-methyl	0.65	ECL deviates -0.003		
4.0511	808	0.014	----	17.1093		----			
4.1398	844	0.018	1.022	17.2387	16:0 2OH	0.15	ECL deviates -0.001		
4.2544	3979	0.017	1.020	17.4058	17:0 10-methyl	0.72	ECL deviates -0.001		
4.3156	1757	0.026	----	17.4952		----			
4.3726	2359	0.016	1.017	17.5782	18:3 w6c	0.43	ECL deviates -0.002		
4.3994	2042	0.017	1.016	17.6173	18:0 iso	0.37	ECL deviates -0.009	Reference -0.016	
4.4294	833	0.017	----	17.6611		----			
4.4736	14451	0.018	1.015	17.7255	18:2 w6c	2.60	ECL deviates -0.002		
4.5052	29244	0.018	1.014	17.7716	18:1 w9c	5.26	ECL deviates -0.003		
4.5424	41376	0.018	1.013	17.8260	18:1 w7c	7.44	ECL deviates -0.001		
4.5998	6759	0.021	----	17.9096		----			
4.6615	9944	0.017	1.010	17.9996	18:0	1.78	ECL deviates  0.000	Reference -0.007	
4.7199	3875	0.016	1.009	18.0815	18:1 w7c 10-methyl	0.69	ECL deviates -0.004		
4.7801	1157	0.021	1.008	18.1654	18:2 DMA	0.21	ECL deviates  0.005		
4.9410	16774	0.020	1.004	18.3900	18:0 10-methyl	2.99	ECL deviates -0.005		
5.0587	2388	0.018	1.002	18.5543	19:3 w6c	0.42	ECL deviates -0.006		
5.1907	1871	0.026	----	18.7386		----		Reference  0.005	
5.2443	2224	0.017	0.998	18.8133	19:1 w8c	0.39	ECL deviates  0.002		
5.2845	2919	0.017	0.997	18.8694	19:0 cyclo w9c	0.52	ECL deviates -0.003		
5.3106	13751	0.018	0.996	18.9058	19:0 cyclo w7c	2.43	ECL deviates -0.004		
5.3796	58705	0.018	----	19.0020	19:0	----	ECL deviates  0.002		
5.5781	789	0.014	----	19.2716		----			
5.6475	2636	0.026	----	19.3659		----			
5.8202	1904	0.028	----	19.6003		----			
5.9039	779	0.016	----	19.7139		----			
5.9462	2071	0.023	0.984	19.7713	20:1 w9c	0.36	ECL deviates -0.001		
5.9675	494	0.012	0.984	19.8002	20:1 w8c	0.09	ECL deviates -0.013		
6.1149	3004	0.019	0.981	20.0001	20:0	0.52	ECL deviates  0.000	Reference -0.006	
6.2560	972	0.020	----	20.1913		----			
6.3694	2773	0.016	----	20.3450		----			
6.3983	18554	0.017	0.978	20.3841	20:0 10-methyl	3.22	ECL deviates -0.013		
6.5697	2225	0.023	----	20.6162		----			
6.6491	2430	0.024	----	20.7237		----			
6.7017	2079	0.017	0.975	20.7951	21:1 w8c	0.36	ECL deviates -0.003		
6.7632	1133	0.018	----	20.8783		----			
6.8197	3635	0.016	0.974	20.9548	21:1 w3c	0.63	ECL deviates  0.001		
6.8656	1241	0.027	----	21.0169		----		Reference  0.011	
7.0606	742	0.016	----	21.2820		----			
7.3065	2647	0.039	0.973	21.6161	22:0 iso	----	> max ar/ht		
7.3641	1778	0.028	----	21.6944		----			
7.4557	2524	0.025	0.974	21.8188	22:1 w8c	0.44	ECL deviates  0.005		
7.5416	1105	0.017	0.975	21.9355	22:1 w3c	0.19	ECL deviates -0.011		
7.5861	3402	0.019	0.975	21.9960	22:0	0.59	ECL deviates -0.004	Reference -0.009	
7.7761	79514	0.017	----	22.2587		----			
8.0856	1840	0.023	----	22.6868		----			
8.2551	1526	0.016	0.987	22.9212	23:1 w4c	0.27	ECL deviates -0.005		
8.3121	709	0.016	0.988	23.0000	23:0	0.12	ECL deviates  0.000	Reference -0.003	
8.5211	1004	0.016	----	23.2936		----			
8.7920	1635	0.025	----	23.6742		----			
8.9402	1361	0.021	----	23.8825		----			
9.0187	2745	0.018	1.017	23.9928	24:0	0.50	ECL deviates -0.007	Reference -0.009	
9.3828	4730	0.018	----	24.5041		----	> max rt		
9.4891	679	0.014	----	24.6534		----	> max rt		

ECL Deviation: 0.005                            Reference ECL Shift: 0.007       Number Reference Peaks: 22
Total Response: 676182                         Total Named: 550555
Percent Named: 81.42%                         Total Amount: 566141
Profile Comment:   Review report comments.

(No search libraries specified in method PLFAD1.)
