# Supplementary material for: Microbial regulation of soil carbon properties under nitrogen addition and plant inputs removal
Source: PeerJ. 2019 Jul 17;7:e7343. doi: 10.7717/peerj.7343 (PMC6642627; doi:10.7717/peerj.7343)
Supplement: File S1 — The raw data showed the soil microbial PLFAs files in the year of 2015 and 2016. Each file of rtf. represented the microbial PLFAs for each soil sample. In the Supplemental File, the Excel file named “Numbers” showed the plots names and the related rtf. file names. [file peerj-07-7343-s002.zip › supplementary files/2016/83.rtf]

Volume: DATA            File: E17C213.73A       Samp Ctr: 3                   ID Number: 5056 
Type: Samp                   Bottle: 2                        Method: PLFAD1 
Created: 12/21/2017 9:48:17 AM 
Sample ID: 83 


RT	Response	Ar/Ht	RFact	ECL	Peak Name	Percent	Comment1	Comment2	
0.7654	1.713E+9	0.016	----	7.7024	SOLVENT PEAK	----	< min rt		
0.9517	436	0.010	----	8.7641		----	< min rt		
1.8096	512	0.012	1.001	12.7228	13:0 anteiso	0.13	ECL deviates  0.013	Reference  0.018	
1.9898	1038	0.021	----	13.2354		----			
2.1372	3314	0.015	1.025	13.6073	14:0 iso	0.89	ECL deviates -0.007	Reference -0.004	
2.1597	443	0.010	----	13.6639		----			
2.2664	593	0.013	----	13.9331		----			
2.2916	3099	0.015	1.031	13.9965	14:0	0.84	ECL deviates -0.004	Reference -0.002	
2.3532	1162	0.012	----	14.1245	14:0 iso 3OH	----	ECL deviates  0.000		
2.4523	575	0.013	----	14.3294		----			
2.5047	3454	0.018	1.035	14.4380	15:1 iso w6c	0.94	ECL deviates -0.001		
2.5292	628	0.011	1.036	14.4886	15:4 w3c	0.17	ECL deviates -0.002		
2.5475	605	0.012	1.036	14.5267	15:1 anteiso w9c	0.16	ECL deviates -0.003		
2.5889	17533	0.015	1.036	14.6124	15:0 iso	4.75	ECL deviates -0.005	Reference -0.004	
2.6349	12123	0.015	1.037	14.7075	15:0 anteiso	3.29	ECL deviates -0.003	Reference -0.003	
2.7752	2011	0.016	1.038	14.9978	15:0	0.55	ECL deviates -0.002	Reference -0.003	
2.8048	846	0.015	----	15.0506		----			
3.0023	553	0.014	1.038	15.4003	16:1 w7c alcohol	0.15	ECL deviates  0.004		
3.0286	2561	0.019	1.037	15.4469	15:0 DMA	0.69	ECL deviates -0.004		
3.0973	11576	0.016	1.037	15.5686	16:3 w6c	3.14	ECL deviates -0.007		
3.1263	7863	0.016	1.037	15.6200	16:0 iso	2.13	ECL deviates  0.000	Reference -0.001	
3.1826	1209	0.015	1.036	15.7196	16:0 anteiso	0.33	ECL deviates  0.005	Reference  0.003	
3.2124	3099	0.014	1.036	15.7724	16:1 w9c	0.84	ECL deviates -0.003		
3.2403	27220	0.016	1.036	15.8217	16:1 w7c	7.37	ECL deviates -0.003		
3.2912	7154	0.017	1.035	15.9119	16:1 w5c	1.94	ECL deviates  0.001		
3.3408	36691	0.016	1.034	15.9996	16:0	9.93	ECL deviates  0.000	Reference -0.002	
3.3717	2273	0.017	----	16.0487		----			
3.6097	16107	0.019	1.031	16.4246	16:0 10-methyl	4.34	ECL deviates  0.005		
3.6543	67200	0.016	1.030	16.4949	17:1 iso w9c	18.11	ECL deviates -0.003		
3.7359	4617	0.015	1.029	16.6239	17:0 iso	1.24	ECL deviates  0.000	Reference -0.003	
3.7969	5345	0.016	1.028	16.7202	17:0 anteiso	1.44	ECL deviates  0.000		
3.8443	2220	0.015	1.027	16.7949	17:1 w8c	0.60	ECL deviates -0.002		
3.9074	10048	0.018	1.025	16.8945	17:0 cyclo w7c	2.70	ECL deviates  0.001		
3.9728	1539	0.015	1.024	16.9979	17:0	0.41	ECL deviates -0.002	Reference -0.005	
4.0017	2775	0.016	1.024	17.0405	17:1 w7c 10-methyl	0.74	ECL deviates -0.003		
4.2511	2273	0.014	1.019	17.4051	17:0 10-methyl	0.61	ECL deviates -0.002		
4.3060	1162	0.025	----	17.4854		----			
4.3684	2036	0.016	1.016	17.5766	18:3 w6c	0.54	ECL deviates -0.003		
4.3969	1429	0.017	1.015	17.6182	18:0 iso	0.38	ECL deviates -0.008	Reference -0.012	
4.4241	655	0.015	----	17.6580		----			
4.4712	8140	0.018	1.014	17.7268	18:2 w6c	2.16	ECL deviates  0.000		
4.5033	17917	0.018	1.013	17.7737	18:1 w9c	4.75	ECL deviates -0.001		
4.5395	27037	0.018	1.012	17.8267	18:1 w7c	7.16	ECL deviates  0.000		
4.6012	3813	0.022	1.011	17.9168	18:1 w5c	1.01	ECL deviates -0.006		
4.6579	6673	0.017	1.010	17.9997	18:0	1.76	ECL deviates  0.000	Reference -0.004	
4.7168	2560	0.017	1.009	18.0821	18:1 w7c 10-methyl	0.68	ECL deviates -0.003		
4.9378	9247	0.020	1.004	18.3904	18:0 10-methyl	2.43	ECL deviates -0.005		
5.0546	2473	0.018	1.001	18.5533	19:3 w6c	0.65	ECL deviates -0.007		
5.1888	1527	0.028	----	18.7405		----		Reference  0.009	
5.2432	1129	0.019	0.998	18.8163	19:1 w8c	0.29	ECL deviates  0.005		
5.3076	9310	0.019	0.996	18.9062	19:0 cyclo w7c	2.43	ECL deviates -0.004		
5.3758	56978	0.017	----	19.0013	19:0	----	ECL deviates  0.001		
5.6680	1120	0.025	0.989	19.3988	20:4 w6c	0.29	ECL deviates -0.005		
5.8959	1279	0.022	----	19.7086		----			
5.9404	1326	0.019	0.985	19.7691	20:1 w9c	0.34	ECL deviates -0.003		
6.1116	1676	0.020	0.982	20.0018	20:0	0.43	ECL deviates  0.002	Reference -0.003	
6.3672	2378	0.014	----	20.3484		----			
6.3969	17991	0.019	0.978	20.3887	20:0 10-methyl	4.60	ECL deviates -0.008		
6.5646	2032	0.018	----	20.6162		----			
6.6471	2284	0.023	----	20.7281		----			
6.6980	870	0.015	0.976	20.7971	21:1 w8c	0.22	ECL deviates -0.001		
6.8170	1498	0.016	0.975	20.9584	21:1 w3c	0.38	ECL deviates  0.004		
7.3608	585	0.013	----	21.6993		----			
7.4532	1585	0.019	----	21.8253		----			
7.5830	1687	0.016	0.977	22.0022	22:0	0.43	ECL deviates  0.002	Reference -0.002	
7.7739	74765	0.020	----	22.2660		----			
8.0831	1713	0.019	----	22.6929		----			
8.2531	840	0.016	0.989	22.9277	23:1 w4c	0.22	ECL deviates  0.001		
8.7919	1491	0.023	----	23.6818		----			
8.9356	1348	0.019	----	23.8832		----			
9.0161	1623	0.018	1.019	23.9960	24:0	0.43	ECL deviates -0.004	Reference -0.006	
9.3806	4886	0.017	----	24.5068		----	> max rt		
9.4870	1144	0.018	----	24.6559		----	> max rt		

ECL Deviation: 0.004                            Reference ECL Shift: 0.007       Number Reference Peaks: 17
Total Response: 472292                         Total Named: 373721
Percent Named: 79.13%                         Total Amount: 382300

(No search libraries specified in method PLFAD1.)
