# Supplementary material for: Microbial regulation of soil carbon properties under nitrogen addition and plant inputs removal
Source: PeerJ. 2019 Jul 17;7:e7343. doi: 10.7717/peerj.7343 (PMC6642627; doi:10.7717/peerj.7343)
Supplement: File S1 — The raw data showed the soil microbial PLFAs files in the year of 2015 and 2016. Each file of rtf. represented the microbial PLFAs for each soil sample. In the Supplemental File, the Excel file named “Numbers” showed the plots names and the related rtf. file names. [file peerj-07-7343-s002.zip › supplementary files/2016/84.rtf]

Volume: DATA            File: E17C213.73A       Samp Ctr: 4                   ID Number: 5057 
Type: Samp                   Bottle: 3                        Method: PLFAD1 
Created: 12/21/2017 10:16:40 AM 
Sample ID: 84 


RT	Response	Ar/Ht	RFact	ECL	Peak Name	Percent	Comment1	Comment2	
0.7658	1.692E+9	0.016	----	7.7077	SOLVENT PEAK	----	< min rt		
0.9535	991	0.011	----	8.7774		----	< min rt		
1.3864	727	0.015	0.928	11.1766	10:0 2OH	0.04	ECL deviates -0.007		
1.5845	1159	0.016	0.973	12.0066	12:0	0.07	ECL deviates  0.007	Reference  0.008	
1.7712	1239	0.013	0.998	12.6039	13:0 iso	0.08	ECL deviates -0.009	Reference -0.008	
1.8070	770	0.013	1.001	12.7183	13:0 anteiso	0.05	ECL deviates  0.009	Reference  0.010	
1.8917	1523	0.026	1.010	12.9894	13:0	0.10	ECL deviates -0.011	Reference -0.010	
1.9703	431	0.009	----	13.1897	12:0 2OH	----	ECL deviates  0.003		
1.9891	1103	0.015	----	13.2369		----			
2.1377	13307	0.017	1.025	13.6113	14:0 iso	0.87	ECL deviates -0.003	Reference -0.003	
2.1817	913	0.013	1.026	13.7221	14:0 anteiso	0.06	ECL deviates  0.006	Reference  0.006	
2.2144	1560	0.024	1.028	13.8045	14:1 w8c	0.10	ECL deviates  0.003		
2.2922	18441	0.017	1.031	14.0003	14:0	1.22	ECL deviates  0.000	Reference  0.000	
2.3542	2134	0.015	----	14.1286	14:0 iso 3OH	----	ECL deviates  0.004		
2.4495	893	0.018	----	14.3256		----			
2.5043	17986	0.018	1.035	14.4390	15:1 iso w6c	1.19	ECL deviates  0.000		
2.5254	1853	0.010	1.036	14.4826	15:4 w3c	0.12	ECL deviates -0.008		
2.5484	3072	0.013	1.036	14.5303	15:1 anteiso w9c	0.20	ECL deviates  0.000		
2.5895	85397	0.015	1.036	14.6151	15:0 iso	5.66	ECL deviates -0.002	Reference -0.003	
2.6356	47879	0.015	1.037	14.7106	15:0 anteiso	3.17	ECL deviates  0.000	Reference -0.002	
2.7042	1572	0.018	1.037	14.8524	15:1 w6c	0.10	ECL deviates -0.008		
2.7754	9054	0.016	1.038	14.9997	15:0	0.60	ECL deviates  0.000	Reference -0.002	
2.8064	2068	0.017	----	15.0548		----			
2.9072	2108	0.016	----	15.2331		----			
3.0262	12232	0.021	1.037	15.4436	15:0 DMA	0.81	ECL deviates -0.007		
3.0976	14602	0.016	1.037	15.5698	16:3 w6c	0.97	ECL deviates -0.006		
3.1261	33522	0.016	1.037	15.6203	16:0 iso	2.22	ECL deviates  0.001	Reference -0.002	
3.1810	3924	0.016	1.036	15.7175	16:0 anteiso	0.26	ECL deviates  0.003	Reference  0.000	
3.2112	15307	0.018	1.036	15.7708	16:1 w9c	1.01	ECL deviates -0.004		
3.2407	110225	0.018	1.036	15.8231	16:1 w7c	7.30	ECL deviates -0.001		
3.2926	29727	0.016	1.035	15.9148	16:1 w5c	1.97	ECL deviates  0.004		
3.3139	6750	0.011	1.035	15.9525	16:1 w3c	0.45	ECL deviates  0.000		
3.3427	196571	0.015	1.034	16.0033	16:0	13.00	ECL deviates  0.003	Reference  0.001	
3.3716	4895	0.020	----	16.0490		----			
3.4313	1805	0.017	1.033	16.1432	16:2 DMA	0.12	ECL deviates  0.005		
3.4684	1083	0.014	----	16.2018		----			
3.4820	936	0.013	----	16.2232		----			
3.5090	541	0.014	1.032	16.2658	16:1 w9c DMA	0.04	ECL deviates -0.009		
3.5397	938	0.022	1.032	16.3143	16:1 w7c DMA	0.06	ECL deviates  0.004		
3.6095	70643	0.019	1.031	16.4244	16:0 10-methyl	4.65	ECL deviates  0.004		
3.6551	117228	0.017	1.030	16.4963	17:1 iso w9c	7.72	ECL deviates -0.002		
3.7364	22088	0.017	1.029	16.6247	17:0 iso	1.45	ECL deviates  0.001	Reference -0.002	
3.7964	23016	0.018	1.028	16.7194	17:0 anteiso	1.51	ECL deviates -0.001		
3.8443	10831	0.018	1.027	16.7950	17:1 w8c	0.71	ECL deviates -0.002		
3.9083	59246	0.019	1.025	16.8959	17:0 cyclo w7c	3.88	ECL deviates  0.002		
3.9741	8727	0.019	1.024	16.9997	17:0	0.57	ECL deviates  0.000	Reference -0.003	
4.0012	6730	0.016	1.024	17.0396	17:1 w7c 10-methyl	0.44	ECL deviates -0.004		
4.0467	1842	0.014	----	17.1061		----			
4.0829	807	0.016	----	17.1590		----			
4.1365	2583	0.017	1.021	17.2373	16:0 2OH	0.17	ECL deviates -0.003		
4.2507	11981	0.016	1.019	17.4041	17:0 10-methyl	0.78	ECL deviates -0.003		
4.3148	5726	0.024	----	17.4978		----			
4.3709	5384	0.017	1.016	17.5798	18:3 w6c	0.35	ECL deviates  0.000		
4.3968	5943	0.020	1.015	17.6176	18:0 iso	0.39	ECL deviates -0.009	Reference -0.012	
4.4284	1919	0.016	----	17.6637		----			
4.4703	51161	0.016	1.014	17.7249	18:2 w6c	3.32	ECL deviates -0.002		
4.5038	83888	0.018	1.013	17.7739	18:1 w9c	5.43	ECL deviates -0.001		
4.5404	136971	0.018	1.012	17.8274	18:1 w7c	8.86	ECL deviates  0.000		
4.5964	29419	0.021	----	17.9092		----			
4.6591	28656	0.019	1.010	18.0009	18:0	1.85	ECL deviates  0.001	Reference -0.003	
4.7178	16550	0.016	1.009	18.0828	18:1 w7c 10-methyl	1.07	ECL deviates -0.002		
4.7739	3451	0.024	1.007	18.1611	18:2 DMA	0.22	ECL deviates  0.001		
4.8216	2642	0.025	1.006	18.2275	18:1 w9c DMA	0.17	ECL deviates -0.009		
4.8597	641	0.014	1.006	18.2806	18:1 w7c DMA	0.04	ECL deviates -0.002		
4.9381	41201	0.017	1.004	18.3899	18:0 10-methyl	2.64	ECL deviates -0.005		
4.9631	4354	0.012	1.003	18.4249	18:0 DMA	0.28	ECL deviates -0.005		
5.0068	1164	0.018	1.002	18.4858	19:4 w6c	0.07	ECL deviates  0.001		
5.0544	5099	0.021	1.001	18.5522	19:3 w6c	0.33	ECL deviates -0.008		
5.1342	2335	0.021	1.000	18.6635	19:3 w3c	0.15	ECL deviates  0.005		
5.1963	4342	0.025	----	18.7501		----			
5.2379	6200	0.018	0.998	18.8080	19:1 w8c	0.40	ECL deviates -0.003		
5.2732	9159	0.021	0.997	18.8572	19:1 w6c	0.58	ECL deviates  0.005		
5.3067	37104	0.019	0.996	18.9040	19:0 cyclo w7c	2.36	ECL deviates -0.006		
5.3766	63135	0.018	----	19.0015	19:0	----	ECL deviates  0.001		
5.4452	1297	0.019	0.993	19.0948	19:1 w7c 10-methyl	0.08	ECL deviates -0.008		
5.5281	2723	0.018	----	19.2075		----			
5.5721	5978	0.018	----	19.2673		----			
5.6097	1635	0.016	0.990	19.3184	19:0 cyclo 9,10 DMA	0.10	ECL deviates -0.005		
5.6432	7559	0.018	----	19.3639		----			
5.6660	5237	0.018	0.989	19.3949	20:4 w6c	0.33	ECL deviates -0.008		
5.7204	2604	0.019	0.988	19.4688	20:5 w3c	0.16	ECL deviates -0.013		
5.7897	3874	0.031	0.987	19.5631	20:3 w6c	0.24	ECL deviates -0.003		
5.8168	3646	0.019	----	19.5999		----			
5.8520	1099	0.017	----	19.6478		----			
5.9386	9674	0.030	0.985	19.7655	20:1 w9c	0.61	ECL deviates -0.007		
5.9643	3384	0.021	0.984	19.8005	20:1 w8c	0.21	ECL deviates -0.013		
6.1082	9437	0.023	0.982	19.9960	20:0	0.59	ECL deviates -0.004	Reference -0.008	
6.2170	1127	0.015	----	20.1436		----			
6.2515	2888	0.017	----	20.1904		----			
6.3362	743	0.017	----	20.3053		----			
6.3650	3674	0.015	----	20.3443		----			
6.3944	23739	0.018	0.978	20.3842	20:0 10-methyl	1.48	ECL deviates -0.013		
6.5172	1518	0.025	----	20.5507		----			
6.5634	2550	0.017	----	20.6133		----			
6.5921	1342	0.015	0.977	20.6523	21:3 w3c	0.08	ECL deviates -0.001		
6.6455	4307	0.024	----	20.7247		----			
6.6977	4758	0.018	0.976	20.7955	21:1 w8c	0.30	ECL deviates -0.003		
6.7597	4811	0.017	----	20.8796		----			
6.8144	5730	0.016	0.975	20.9537	21:1 w3c	0.36	ECL deviates  0.000		
6.8563	2505	0.027	0.975	21.0106	21:0	0.16	ECL deviates  0.011	Reference  0.007	
6.9334	744	0.014	----	21.1158		----			
7.0538	4541	0.019	----	21.2799		----			
7.1948	1346	0.027	0.974	21.4721	22:5 w3c	0.08	ECL deviates  0.004		
7.3049	7535	0.034	0.975	21.6222	22:0 iso	0.47	ECL deviates  0.004		
7.3327	2188	0.015	----	21.6601		----			
7.3585	2561	0.020	----	21.6952		----			
7.4534	10564	0.035	----	21.8246		----			
7.5382	3085	0.019	0.976	21.9403	22:1 w3c	0.19	ECL deviates -0.007		
7.5821	14862	0.019	0.977	22.0001	22:0	0.93	ECL deviates  0.000	Reference -0.003	
7.7758	114155	0.020	----	22.2677		----			
8.0806	2310	0.020	----	22.6889		----			
8.1469	2669	0.020	----	22.7804		----			
8.2058	1579	0.021	----	22.8618		----			
8.2514	4223	0.019	0.989	22.9248	23:1 w4c	0.27	ECL deviates -0.002		
8.3062	2812	0.018	0.990	23.0005	23:0	0.18	ECL deviates  0.001	Reference -0.002	
8.5187	3615	0.020	----	23.2984		----			
8.7771	5199	0.032	1.007	23.6609	24:3 w3c	0.33	ECL deviates  0.006		
8.8290	5316	0.026	----	23.7336		----			
8.9351	2450	0.017	----	23.8824		----			
9.0164	8903	0.019	1.019	23.9964	24:0	0.58	ECL deviates -0.004	Reference -0.006	
9.3796	6967	0.019	----	24.5056		----	> max rt		
9.4830	1206	0.019	----	24.6506		----	> max rt		

ECL Deviation: 0.005                            Reference ECL Shift: 0.006       Number Reference Peaks: 22
Total Response: 1783210                       Total Named: 1530753
Percent Named: 85.84%                         Total Amount: 1564419

(No search libraries specified in method PLFAD1.)
