# Supplementary material for: Microbial regulation of soil carbon properties under nitrogen addition and plant inputs removal
Source: PeerJ. 2019 Jul 17;7:e7343. doi: 10.7717/peerj.7343 (PMC6642627; doi:10.7717/peerj.7343)
Supplement: File S1 — The raw data showed the soil microbial PLFAs files in the year of 2015 and 2016. Each file of rtf. represented the microbial PLFAs for each soil sample. In the Supplemental File, the Excel file named “Numbers” showed the plots names and the related rtf. file names. [file peerj-07-7343-s002.zip › supplementary files/2016/85.rtf]

Volume: DATA            File: E17C213.73A       Samp Ctr: 5                   ID Number: 5058 
Type: Samp                   Bottle: 4                        Method: PLFAD1 
Created: 12/21/2017 10:45:07 AM 
Sample ID: 85 


RT	Response	Ar/Ht	RFact	ECL	Peak Name	Percent	Comment1	Comment2	
0.7648	1.68E+9	0.016	----	7.6955	SOLVENT PEAK	----	< min rt		
0.9513	1088	0.012	----	8.7583		----	< min rt		
1.5846	712	0.017	0.973	12.0010	12:0	0.07	ECL deviates  0.001	Reference  0.009	
1.7731	892	0.017	0.998	12.6039	13:0 iso	0.09	ECL deviates -0.008	Reference -0.001	
1.8086	628	0.015	1.001	12.7175	13:0 anteiso	0.06	ECL deviates  0.008	Reference  0.015	
1.9916	1289	0.019	----	13.2368		----			
2.1397	9026	0.017	1.025	13.6099	14:0 iso	0.90	ECL deviates -0.004	Reference  0.002	
2.1843	747	0.014	1.026	13.7221	14:0 anteiso	0.07	ECL deviates  0.006	Reference  0.012	
2.2942	10436	0.015	1.031	13.9978	14:0	1.05	ECL deviates -0.002	Reference  0.004	
2.3570	1964	0.014	----	14.1276	14:0 iso 3OH	----	ECL deviates  0.003		
2.4553	625	0.015	----	14.3308		----			
2.5088	10773	0.015	1.035	14.4414	15:1 iso w6c	1.09	ECL deviates  0.002		
2.5291	1388	0.010	1.036	14.4835	15:4 w3c	0.14	ECL deviates -0.007		
2.5519	1814	0.014	1.036	14.5307	15:1 anteiso w9c	0.18	ECL deviates  0.001		
2.5931	52632	0.015	1.036	14.6157	15:0 iso	5.33	ECL deviates -0.001	Reference  0.004	
2.6387	35068	0.015	1.037	14.7100	15:0 anteiso	3.55	ECL deviates -0.001	Reference  0.005	
2.7097	1164	0.017	1.037	14.8568	15:1 w6c	0.12	ECL deviates -0.003		
2.7791	6015	0.015	1.038	14.9994	15:0	0.61	ECL deviates -0.001	Reference  0.005	
2.8106	1255	0.015	----	15.0551		----			
2.9112	1016	0.015	----	15.2332		----			
3.0049	825	0.010	1.038	15.3987	16:1 w7c alcohol	0.08	ECL deviates  0.002		
3.0310	6812	0.020	1.037	15.4450	15:0 DMA	0.69	ECL deviates -0.005		
3.1016	13435	0.016	1.037	15.5698	16:3 w6c	1.36	ECL deviates -0.006		
3.1302	21798	0.015	1.037	15.6205	16:0 iso	2.21	ECL deviates  0.001	Reference  0.006	
3.1850	2866	0.016	1.036	15.7174	16:0 anteiso	0.29	ECL deviates  0.002	Reference  0.007	
3.2159	9448	0.018	1.036	15.7720	16:1 w9c	0.96	ECL deviates -0.003		
3.2452	71219	0.017	1.036	15.8237	16:1 w7c	7.21	ECL deviates -0.001		
3.2969	20075	0.016	1.035	15.9152	16:1 w5c	2.03	ECL deviates  0.004		
3.3161	2516	0.008	1.035	15.9492	16:1 w3c	0.25	ECL deviates -0.003		
3.3463	115803	0.016	1.034	16.0018	16:0	11.71	ECL deviates  0.002	Reference  0.007	
3.3761	3734	0.017	----	16.0488		----			
3.4335	724	0.017	1.033	16.1394	16:2 DMA	0.07	ECL deviates  0.001		
3.4741	1168	0.025	----	16.2035		----			
3.6145	44955	0.019	1.031	16.4251	16:0 10-methyl	4.53	ECL deviates  0.005		
3.6599	99753	0.017	1.030	16.4967	17:1 iso w9c	10.04	ECL deviates -0.001		
3.7415	13569	0.016	1.029	16.6254	17:0 iso	1.36	ECL deviates  0.002	Reference  0.006	
3.8016	16901	0.017	1.028	16.7203	17:0 anteiso	1.70	ECL deviates  0.000		
3.8503	6593	0.018	1.027	16.7970	17:1 w8c	0.66	ECL deviates  0.000		
3.9138	36446	0.018	1.025	16.8973	17:0 cyclo w7c	3.65	ECL deviates  0.004		
3.9809	5306	0.017	1.024	17.0025	17:0	0.53	ECL deviates  0.003	Reference  0.007	
4.0075	5057	0.016	1.024	17.0414	17:1 w7c 10-methyl	0.51	ECL deviates -0.002		
4.0536	1415	0.015	----	17.1088		----			
4.1437	2042	0.021	1.021	17.2405	16:0 2OH	0.20	ECL deviates  0.000		
4.2578	7733	0.016	1.019	17.4071	17:0 10-methyl	0.77	ECL deviates  0.000		
4.3191	3053	0.025	----	17.4967		----			
4.3753	4291	0.017	1.016	17.5788	18:3 w6c	0.43	ECL deviates -0.001		
4.4013	4836	0.018	1.015	17.6167	18:0 iso	0.48	ECL deviates -0.010	Reference -0.006	
4.4329	960	0.013	----	17.6630		----			
4.4754	32952	0.018	1.014	17.7250	18:2 w6c	3.27	ECL deviates -0.002		
4.5079	56053	0.019	1.013	17.7726	18:1 w9c	5.55	ECL deviates -0.002		
4.5452	95480	0.019	1.012	17.8270	18:1 w7c	9.45	ECL deviates  0.000		
4.6034	16209	0.023	----	17.9119		----			
4.6642	18669	0.019	1.010	18.0006	18:0	1.84	ECL deviates  0.001	Reference  0.005	
4.7238	9587	0.019	1.009	18.0837	18:1 w7c 10-methyl	0.95	ECL deviates -0.001		
4.7799	1857	0.021	1.007	18.1620	18:2 DMA	0.18	ECL deviates  0.002		
4.8209	2997	0.029	----	18.2191		----			
4.8956	766	0.019	----	18.3233		----			
4.9435	30027	0.019	1.004	18.3901	18:0 10-methyl	2.95	ECL deviates -0.005		
5.0171	867	0.016	1.002	18.4927	19:4 w6c	0.08	ECL deviates  0.008		
5.0612	3855	0.020	1.001	18.5542	19:3 w6c	0.38	ECL deviates -0.006		
5.1395	1385	0.021	1.000	18.6634	19:3 w3c	0.14	ECL deviates  0.005		
5.1979	3246	0.027	----	18.7449		----			
5.2440	3715	0.018	0.998	18.8092	19:1 w8c	0.36	ECL deviates -0.002		
5.2790	5464	0.018	0.997	18.8580	19:1 w6c	0.53	ECL deviates  0.006		
5.3130	29084	0.018	0.996	18.9054	19:0 cyclo w7c	2.83	ECL deviates -0.004		
5.3821	59641	0.018	----	19.0016	19:0	----	ECL deviates  0.002		
5.4478	872	0.015	----	19.0910		----			
5.5345	1526	0.017	----	19.2088		----			
5.5790	3383	0.016	----	19.2693		----			
5.6134	986	0.016	0.990	19.3161	19:0 cyclo 9,10 DMA	0.10	ECL deviates -0.008		
5.6497	3579	0.019	----	19.3654		----			
5.6720	2549	0.015	0.989	19.3957	20:4 w6c	0.25	ECL deviates -0.008		
5.7276	1603	0.022	0.988	19.4714	20:5 w3c	0.15	ECL deviates -0.011		
5.7651	1175	0.018	----	19.5223		----			
5.7935	1851	0.019	0.987	19.5610	20:3 w6c	0.18	ECL deviates -0.005		
5.8233	3575	0.024	----	19.6014		----			
5.9437	10463	0.042	0.985	19.7651	20:1 w9c	----	> max ar/ht		
6.1169	7450	0.025	0.982	20.0007	20:0	0.72	ECL deviates  0.001	Reference  0.004	
6.2250	1233	0.017	----	20.1473		----			
6.2573	1986	0.019	----	20.1910		----			
6.3721	5005	0.021	----	20.3467		----			
6.4003	22412	0.020	0.978	20.3850	20:0 10-methyl	2.14	ECL deviates -0.012		
6.4347	1450	0.015	----	20.4317		----			
6.4638	1419	0.017	----	20.4712		----			
6.5064	2145	0.031	----	20.5289		----			
6.5715	3509	0.028	----	20.6173		----			
6.6468	4085	0.027	----	20.7194		----			
6.7039	2937	0.018	0.976	20.7968	21:1 w8c	0.28	ECL deviates -0.001		
6.7640	3589	0.020	----	20.8783		----			
6.8211	4177	0.018	0.975	20.9558	21:1 w3c	0.40	ECL deviates  0.002		
6.8654	2198	0.028	----	21.0160		----			
7.0611	2552	0.019	----	21.2829		----			
7.3124	4467	0.034	0.975	21.6256	22:0 iso	0.43	ECL deviates  0.008		
7.3636	2979	0.028	----	21.6954		----			
7.4597	5697	0.029	----	21.8264		----			
7.5420	1289	0.016	0.976	21.9387	22:1 w3c	0.12	ECL deviates -0.008		
7.5887	7890	0.019	0.977	22.0024	22:0	0.75	ECL deviates  0.002	Reference  0.006	
7.7793	134646	0.019	----	22.2659		----			
8.0862	2282	0.022	----	22.6902		----			
8.1529	1854	0.020	----	22.7824		----			
8.2559	2735	0.017	0.989	22.9246	23:1 w4c	0.26	ECL deviates -0.002		
8.3125	1504	0.016	0.990	23.0030	23:0	0.15	ECL deviates  0.003	Reference  0.006	
8.5213	1895	0.016	----	23.2959		----			
8.7883	3608	0.031	----	23.6706		----			
8.8368	3010	0.024	----	23.7387		----			
8.9374	1635	0.017	----	23.8797		----			
9.0206	5421	0.016	1.019	23.9965	24:0	0.54	ECL deviates -0.004	Reference  0.000	
9.3866	8344	0.019	----	24.5100		----	> max rt		
9.4875	835	0.015	----	24.6515		----	> max rt		

ECL Deviation: 0.005                            Reference ECL Shift: 0.007       Number Reference Peaks: 20
Total Response: 1249644                       Total Named: 1000564
Percent Named: 80.07%                         Total Amount: 1033188
Profile Comment:   Review report comments.

(No search libraries specified in method PLFAD1.)
