# Supplementary material for: Microbial regulation of soil carbon properties under nitrogen addition and plant inputs removal
Source: PeerJ. 2019 Jul 17;7:e7343. doi: 10.7717/peerj.7343 (PMC6642627; doi:10.7717/peerj.7343)
Supplement: File S1 — The raw data showed the soil microbial PLFAs files in the year of 2015 and 2016. Each file of rtf. represented the microbial PLFAs for each soil sample. In the Supplemental File, the Excel file named “Numbers” showed the plots names and the related rtf. file names. [file peerj-07-7343-s002.zip › supplementary files/2016/86.rtf]

Volume: DATA            File: E17C213.73A       Samp Ctr: 6                   ID Number: 5059 
Type: Samp                   Bottle: 5                        Method: PLFAD1 
Created: 12/21/2017 11:13:51 AM 
Sample ID: 86 


RT	Response	Ar/Ht	RFact	ECL	Peak Name	Percent	Comment1	Comment2	
0.7653	1.681E+9	0.016	----	7.6800	SOLVENT PEAK	----	< min rt		
1.2953	545	0.012	0.895	10.7115	11:0 anteiso	0.05	ECL deviates  0.006	Reference  0.020	
1.5875	1860	0.022	0.973	12.0092	12:0	0.17	ECL deviates  0.009	Reference  0.018	
1.7715	486	0.012	0.998	12.5988	13:0 iso	0.05	ECL deviates -0.014	Reference -0.007	
1.8075	530	0.012	1.001	12.7143	13:0 anteiso	0.05	ECL deviates  0.005	Reference  0.012	
1.9883	809	0.019	----	13.2298		----			
2.1377	7073	0.015	1.025	13.6068	14:0 iso	0.70	ECL deviates -0.007	Reference -0.003	
2.2643	997	0.014	----	13.9262		----			
2.2921	8782	0.015	1.031	13.9961	14:0	0.87	ECL deviates -0.004	Reference -0.001	
2.3550	2521	0.014	----	14.1266	14:0 iso 3OH	----	ECL deviates  0.002		
2.3929	1326	0.022	----	14.2050		----			
2.4525	1074	0.018	----	14.3284		----			
2.5051	11291	0.019	1.035	14.4372	15:1 iso w6c	1.12	ECL deviates -0.002		
2.5271	1491	0.012	1.036	14.4827	15:4 w3c	0.15	ECL deviates -0.007		
2.5499	1846	0.013	1.036	14.5300	15:1 anteiso w9c	0.18	ECL deviates  0.000		
2.5902	54662	0.015	1.036	14.6135	15:0 iso	5.44	ECL deviates -0.004	Reference -0.002	
2.6358	32822	0.015	1.037	14.7079	15:0 anteiso	3.27	ECL deviates -0.003	Reference -0.001	
2.7076	1814	0.026	1.037	14.8564	15:1 w6c	0.18	ECL deviates -0.004		
2.7766	5899	0.016	1.038	14.9993	15:0	0.59	ECL deviates -0.001	Reference  0.000	
2.8068	1691	0.017	----	15.0526		----			
2.9102	1622	0.016	----	15.2359		----			
2.9738	574	0.013	----	15.3485		----			
3.0007	1289	0.013	1.038	15.3961	16:1 w7c alcohol	0.13	ECL deviates  0.000		
3.0287	6498	0.021	1.037	15.4457	15:0 DMA	0.65	ECL deviates -0.005		
3.0985	12169	0.016	1.037	15.5694	16:3 w6c	1.21	ECL deviates -0.007		
3.1273	20600	0.016	1.037	15.6203	16:0 iso	2.05	ECL deviates  0.001	Reference  0.001	
3.1804	2850	0.015	1.036	15.7143	16:0 anteiso	0.28	ECL deviates -0.001	Reference -0.001	
3.2127	9518	0.017	1.036	15.7714	16:1 w9c	0.95	ECL deviates -0.004		
3.2426	77554	0.017	1.036	15.8244	16:1 w7c	7.72	ECL deviates  0.000		
3.2941	24733	0.017	1.035	15.9156	16:1 w5c	2.46	ECL deviates  0.004		
3.3436	114152	0.017	1.034	16.0029	16:0	11.34	ECL deviates  0.003	Reference  0.002	
3.3729	3551	0.015	----	16.0492		----			
3.3935	888	0.009	----	16.0818		----			
3.4405	1280	0.022	----	16.1560		----			
3.4687	639	0.016	----	16.2006		----			
3.6114	47487	0.019	1.031	16.4259	16:0 10-methyl	4.70	ECL deviates  0.006		
3.6564	84877	0.017	1.030	16.4970	17:1 iso w9c	8.40	ECL deviates -0.001		
3.7373	14094	0.017	1.029	16.6248	17:0 iso	1.39	ECL deviates  0.001	Reference  0.000	
3.7974	18595	0.018	1.028	16.7197	17:0 anteiso	1.84	ECL deviates -0.001		
3.8472	7236	0.018	1.027	16.7983	17:1 w8c	0.71	ECL deviates  0.001		
3.9103	36275	0.018	1.025	16.8979	17:0 cyclo w7c	3.57	ECL deviates  0.004		
3.9770	5537	0.018	1.024	17.0031	17:0	0.54	ECL deviates  0.003	Reference  0.001	
4.0035	5692	0.017	1.024	17.0419	17:1 w7c 10-methyl	0.56	ECL deviates -0.001		
4.0508	1481	0.016	----	17.1110		----			
4.1160	1908	0.016	----	17.2064		----			
4.1396	1760	0.017	1.021	17.2409	16:0 2OH	0.17	ECL deviates  0.001		
4.2536	7006	0.016	1.019	17.4076	17:0 10-methyl	0.69	ECL deviates  0.001		
4.3162	2761	0.027	----	17.4991		----			
4.3734	3235	0.018	1.016	17.5827	18:3 w6c	0.32	ECL deviates  0.003		
4.3993	5044	0.020	1.015	17.6205	18:0 iso	0.49	ECL deviates -0.006	Reference -0.009	
4.4291	1137	0.014	----	17.6641		----			
4.4726	19659	0.016	1.014	17.7277	18:2 w6c	1.91	ECL deviates  0.001		
4.5060	50276	0.018	1.013	17.7764	18:1 w9c	4.89	ECL deviates  0.002		
4.5416	94323	0.019	1.012	17.8285	18:1 w7c	9.17	ECL deviates  0.002		
4.5998	16887	0.021	1.011	17.9136	18:1 w5c	1.64	ECL deviates -0.009		
4.6604	23368	0.017	1.010	18.0022	18:0	2.27	ECL deviates  0.002	Reference -0.001	
4.7208	8328	0.018	1.009	18.0864	18:1 w7c 10-methyl	0.81	ECL deviates  0.001		
4.7773	2073	0.021	1.007	18.1652	18:2 DMA	0.20	ECL deviates  0.005		
4.8160	2081	0.019	----	18.2192		----			
4.8318	2204	0.019	1.006	18.2413	18:1 w9c DMA	0.21	ECL deviates  0.004		
4.9392	27504	0.021	1.004	18.3911	18:0 10-methyl	2.65	ECL deviates -0.004		
5.0102	2451	0.026	1.002	18.4901	19:4 w6c	0.24	ECL deviates  0.005		
5.0552	5763	0.020	1.001	18.5529	19:3 w6c	0.55	ECL deviates -0.007		
5.1341	1345	0.021	1.000	18.6630	19:3 w3c	0.13	ECL deviates  0.005		
5.1857	5399	0.023	0.999	18.7350	19:0 anteiso	0.52	ECL deviates  0.008	Reference  0.005	
5.2419	3936	0.017	0.998	18.8132	19:1 w8c	0.38	ECL deviates  0.002		
5.2790	4753	0.018	0.997	18.8650	19:0 cyclo w9c	0.46	ECL deviates -0.007		
5.3088	27629	0.019	0.996	18.9065	19:0 cyclo w7c	2.64	ECL deviates -0.003		
5.3783	59045	0.018	----	19.0035	19:0	----	ECL deviates  0.003		
5.4461	1179	0.017	0.993	19.0956	19:1 w7c 10-methyl	0.11	ECL deviates -0.007		
5.5336	2758	0.020	----	19.2147		----			
5.5745	2853	0.016	----	19.2703		----			
5.6117	1553	0.017	0.990	19.3208	19:0 cyclo 9,10 DMA	0.15	ECL deviates -0.003		
5.6475	4991	0.019	----	19.3695		----			
5.6690	2374	0.014	0.989	19.3987	20:4 w6c	0.23	ECL deviates -0.005		
5.6986	642	0.012	----	19.4390		----			
5.7231	1130	0.017	0.988	19.4723	20:5 w3c	0.11	ECL deviates -0.010		
5.7921	2483	0.024	0.987	19.5661	20:3 w6c	0.24	ECL deviates  0.000		
5.8174	3916	0.021	----	19.6004		----			
5.8529	728	0.016	----	19.6487		----			
5.8983	2141	0.018	----	19.7105		----			
5.9420	9345	0.035	0.985	19.7699	20:1 w9c	0.88	ECL deviates -0.003		
6.1134	6792	0.021	0.982	20.0029	20:0	0.64	ECL deviates  0.003	Reference -0.001	
6.2203	1091	0.016	----	20.1478		----			
6.2534	2065	0.017	----	20.1927		----			
6.3696	5123	0.018	----	20.3502		----			
6.3983	26912	0.019	0.978	20.3891	20:0 10-methyl	2.53	ECL deviates -0.008		
6.4338	1610	0.014	----	20.4373		----			
6.4624	1712	0.017	----	20.4761		----			
6.5189	2830	0.031	----	20.5527		----			
6.5641	5068	0.021	----	20.6139		----			
6.5918	1020	0.011	0.977	20.6514	21:3 w3c	0.10	ECL deviates -0.002		
6.6490	4966	0.029	----	20.7290		----			
6.7008	3542	0.020	0.976	20.7993	21:1 w8c	0.33	ECL deviates  0.001		
6.7626	3461	0.022	----	20.8831		----			
6.8170	4448	0.020	0.975	20.9568	21:1 w3c	0.42	ECL deviates  0.003		
6.8703	4201	0.031	----	21.0292		----			
6.9350	1078	0.017	----	21.1173		----			
6.9623	1387	0.022	----	21.1545		----			
7.0570	3161	0.025	----	21.2836		----			
7.3082	4773	0.026	0.975	21.6260	22:0 iso	0.45	ECL deviates  0.008		
7.3613	4405	0.027	----	21.6982		----			
7.4088	2253	0.027	0.975	21.7629	22:1 w9c	0.21	ECL deviates -0.010		
7.4573	7270	0.027	----	21.8291		----			
7.5382	1438	0.016	0.976	21.9393	22:1 w3c	0.13	ECL deviates -0.008		
7.5855	6848	0.018	0.977	22.0038	22:0	0.64	ECL deviates  0.004	Reference  0.001	
7.7738	92022	0.020	----	22.2638		----			
8.0817	3400	0.022	----	22.6889		----			
8.1496	1739	0.019	----	22.7827		----			
8.1928	2454	0.028	----	22.8424		----			
8.2518	3469	0.017	0.989	22.9238	23:1 w4c	0.33	ECL deviates -0.003		
8.3076	2145	0.017	0.990	23.0008	23:0	0.20	ECL deviates  0.001	Reference  0.000	
8.3487	1241	0.022	----	23.0584		----			
8.5179	2230	0.018	----	23.2955		----			
8.7878	5751	0.030	----	23.6736		----			
8.8293	4622	0.026	----	23.7318		----			
8.9381	3787	0.019	----	23.8842		----			
9.0161	6549	0.018	1.019	23.9933	24:0	0.64	ECL deviates -0.007	Reference -0.006	
9.3820	9820	0.020	----	24.5059		----	> max rt		
9.4827	2061	0.018	----	24.6469		----	> max rt		

ECL Deviation: 0.005                            Reference ECL Shift: 0.007       Number Reference Peaks: 21
Total Response: 1227976                       Total Named: 1019455
Percent Named: 83.02%                         Total Amount: 1041069

(No search libraries specified in method PLFAD1.)
