# Supplementary material for: Microbial regulation of soil carbon properties under nitrogen addition and plant inputs removal
Source: PeerJ. 2019 Jul 17;7:e7343. doi: 10.7717/peerj.7343 (PMC6642627; doi:10.7717/peerj.7343)
Supplement: File S1 — The raw data showed the soil microbial PLFAs files in the year of 2015 and 2016. Each file of rtf. represented the microbial PLFAs for each soil sample. In the Supplemental File, the Excel file named “Numbers” showed the plots names and the related rtf. file names. [file peerj-07-7343-s002.zip › supplementary files/2016/87.rtf]

Volume: DATA            File: E17C213.73A       Samp Ctr: 7                   ID Number: 5060 
Type: Samp                   Bottle: 6                        Method: PLFAD1 
Created: 12/21/2017 11:42:28 AM 
Sample ID: 87 


RT	Response	Ar/Ht	RFact	ECL	Peak Name	Percent	Comment1	Comment2	
0.7653	1.676E+9	0.015	----	7.7063	SOLVENT PEAK	----	< min rt		
1.5903	1614	0.023	----	12.0256		----			
1.7707	639	0.013	0.998	12.6028	13:0 iso	0.07	ECL deviates -0.010	Reference -0.009	
1.8089	655	0.015	1.001	12.7250	13:0 anteiso	0.07	ECL deviates  0.016	Reference  0.016	
2.1369	6944	0.015	1.025	13.6099	14:0 iso	0.79	ECL deviates -0.004	Reference -0.005	
2.2911	8434	0.016	1.031	13.9984	14:0	0.96	ECL deviates -0.002	Reference -0.003	
2.3540	2241	0.015	----	14.1291	14:0 iso 3OH	----	ECL deviates  0.004		
2.4501	856	0.017	----	14.3279		----			
2.5037	8918	0.018	1.035	14.4387	15:1 iso w6c	1.02	ECL deviates  0.000		
2.5255	1152	0.010	1.036	14.4837	15:4 w3c	0.13	ECL deviates -0.006		
2.5477	1831	0.016	1.036	14.5297	15:1 anteiso w9c	0.21	ECL deviates  0.000		
2.5887	48276	0.015	1.036	14.6145	15:0 iso	5.55	ECL deviates -0.002	Reference -0.005	
2.6350	31065	0.015	1.037	14.7103	15:0 anteiso	3.57	ECL deviates -0.001	Reference -0.003	
2.7062	1513	0.025	1.037	14.8574	15:1 w6c	0.17	ECL deviates -0.003		
2.7756	5453	0.015	1.038	15.0010	15:0	0.63	ECL deviates  0.001	Reference -0.002	
2.8053	1653	0.015	----	15.0538		----			
2.9077	1194	0.016	----	15.2349		----			
3.0003	1059	0.012	1.038	15.3988	16:1 w7c alcohol	0.12	ECL deviates  0.002		
3.0268	6221	0.019	1.037	15.4457	15:0 DMA	0.72	ECL deviates -0.005		
3.0971	11486	0.015	1.037	15.5700	16:3 w6c	1.32	ECL deviates -0.006		
3.1256	20983	0.016	1.037	15.6204	16:0 iso	2.41	ECL deviates  0.001	Reference -0.002	
3.1800	2711	0.015	1.036	15.7168	16:0 anteiso	0.31	ECL deviates  0.002	Reference -0.001	
3.2115	8539	0.016	1.036	15.7725	16:1 w9c	0.98	ECL deviates -0.003		
3.2402	63194	0.018	1.036	15.8232	16:1 w7c	7.26	ECL deviates -0.001		
3.2914	16564	0.016	1.035	15.9139	16:1 w5c	1.90	ECL deviates  0.003		
3.3129	2147	0.008	1.035	15.9518	16:1 w3c	0.25	ECL deviates  0.000		
3.3412	99296	0.016	1.034	16.0019	16:0	11.40	ECL deviates  0.002	Reference -0.002	
3.3712	4155	0.019	----	16.0494		----			
3.4421	2093	0.023	----	16.1614		----			
3.4836	1417	0.025	----	16.2268		----			
3.6089	44140	0.019	1.031	16.4247	16:0 10-methyl	5.05	ECL deviates  0.005		
3.6539	79998	0.017	1.030	16.4957	17:1 iso w9c	9.14	ECL deviates -0.002		
3.7347	14262	0.017	1.029	16.6232	17:0 iso	1.63	ECL deviates -0.001	Reference -0.005	
3.7954	16201	0.018	1.028	16.7190	17:0 anteiso	1.85	ECL deviates -0.001		
3.8450	6514	0.019	1.027	16.7973	17:1 w8c	0.74	ECL deviates  0.000		
3.9067	33781	0.018	1.025	16.8947	17:0 cyclo w7c	3.84	ECL deviates  0.001		
3.9731	5317	0.019	1.024	16.9995	17:0	0.60	ECL deviates -0.001	Reference -0.005	
4.0001	5649	0.017	1.024	17.0392	17:1 w7c 10-methyl	0.64	ECL deviates -0.004		
4.0458	1433	0.015	----	17.1061		----			
4.1114	1560	0.015	----	17.2019		----			
4.1362	1831	0.016	1.021	17.2381	16:0 2OH	0.21	ECL deviates -0.002		
4.2492	8703	0.017	1.019	17.4033	17:0 10-methyl	0.98	ECL deviates -0.004		
4.2866	605	0.011	1.018	17.4579	17:0 DMA	0.07	ECL deviates  0.000		
4.3111	2889	0.020	----	17.4937		----			
4.3701	3177	0.017	1.016	17.5799	18:3 w6c	0.36	ECL deviates  0.000		
4.3939	4930	0.025	1.015	17.6146	18:0 iso	0.56	ECL deviates -0.012	Reference -0.017	
4.4692	19664	0.017	1.014	17.7246	18:2 w6c	2.21	ECL deviates -0.002		
4.5020	47296	0.019	1.013	17.7726	18:1 w9c	5.32	ECL deviates -0.002		
4.5382	73535	0.017	1.012	17.8254	18:1 w7c	8.26	ECL deviates -0.002		
4.5967	13150	0.023	----	17.9110		----			
4.6573	17737	0.017	1.010	17.9995	18:0	1.99	ECL deviates -0.001	Reference -0.005	
4.7164	7795	0.017	1.009	18.0821	18:1 w7c 10-methyl	0.87	ECL deviates -0.003		
4.7739	2080	0.023	1.007	18.1624	18:2 DMA	0.23	ECL deviates  0.002		
4.8122	3216	0.035	----	18.2158		----			
4.9374	32141	0.020	1.004	18.3904	18:0 10-methyl	3.58	ECL deviates -0.005		
5.0093	786	0.018	1.002	18.4906	19:4 w6c	0.09	ECL deviates  0.006		
5.0532	4616	0.019	1.001	18.5519	19:3 w6c	0.51	ECL deviates -0.008		
5.1346	1316	0.023	1.000	18.6653	19:3 w3c	0.15	ECL deviates  0.007		
5.1886	3148	0.025	----	18.7406		----		Reference  0.009	
5.2384	3768	0.017	0.998	18.8100	19:1 w8c	0.42	ECL deviates -0.001		
5.2742	4811	0.019	0.997	18.8600	19:1 w6c	0.53	ECL deviates  0.008		
5.3061	21864	0.018	0.996	18.9044	19:0 cyclo w7c	2.42	ECL deviates -0.005		
5.3751	60295	0.018	----	19.0006	19:0	----	ECL deviates  0.001		
5.4427	830	0.015	0.993	19.0927	19:1 w7c 10-methyl	0.09	ECL deviates -0.010		
5.5286	2473	0.018	----	19.2094		----			
5.5717	2650	0.015	----	19.2681		----			
5.6073	1155	0.017	0.990	19.3164	19:0 cyclo 9,10 DMA	0.13	ECL deviates -0.007		
5.6435	3283	0.017	----	19.3657		----			
5.6666	2632	0.019	0.989	19.3970	20:4 w6c	0.29	ECL deviates -0.006		
5.7224	859	0.016	0.988	19.4730	20:5 w3c	0.09	ECL deviates -0.009		
5.7567	749	0.015	----	19.5196		----			
5.7932	1321	0.019	0.987	19.5692	20:3 w6c	0.14	ECL deviates  0.003		
5.8182	2434	0.022	----	19.6031		----			
5.8961	1535	0.015	----	19.7090		----			
5.9379	5191	0.026	0.985	19.7659	20:1 w9c	0.57	ECL deviates -0.007		
5.9674	2494	0.022	0.984	19.8060	20:1 w8c	0.27	ECL deviates -0.007		
6.1079	5043	0.019	0.982	19.9970	20:0	0.55	ECL deviates -0.003	Reference -0.008	
6.2191	726	0.015	----	20.1478		----			
6.2498	1692	0.017	----	20.1894		----			
6.3647	4255	0.018	----	20.3453		----			
6.3949	23765	0.019	0.978	20.3862	20:0 10-methyl	2.58	ECL deviates -0.011		
6.4323	1468	0.016	----	20.4369		----			
6.4561	1419	0.017	----	20.4692		----			
6.5168	1678	0.028	----	20.5515		----			
6.5619	4509	0.022	----	20.6127		----			
6.6314	4280	0.027	----	20.7069		----			
6.6972	3285	0.017	0.976	20.7961	21:1 w8c	0.36	ECL deviates -0.002		
6.7579	2460	0.019	----	20.8785		----			
6.8130	4090	0.019	0.975	20.9531	21:1 w3c	0.44	ECL deviates -0.001		
6.8630	1821	0.028	----	21.0211		----			
7.0529	1778	0.017	----	21.2799		----			
7.3073	4145	0.034	0.975	21.6267	22:0 iso	0.45	ECL deviates  0.009		
7.3585	3053	0.021	----	21.6965		----			
7.4065	1839	0.027	----	21.7619		----			
7.4545	5233	0.025	----	21.8273		----			
7.5364	1474	0.016	0.976	21.9390	22:1 w3c	0.16	ECL deviates -0.008		
7.5828	6448	0.018	0.977	22.0022	22:0	0.70	ECL deviates  0.002	Reference -0.002	
7.7729	89059	0.019	----	22.2648		----			
8.0804	3486	0.018	----	22.6897		----			
8.1474	1485	0.019	----	22.7823		----			
8.2515	2200	0.016	0.989	22.9261	23:1 w4c	0.24	ECL deviates  0.000		
8.3058	1432	0.016	0.990	23.0011	23:0	0.16	ECL deviates  0.001	Reference -0.003	
8.5180	1976	0.019	----	23.2987		----			
8.7908	5161	0.026	----	23.6811		----			
8.8315	2747	0.026	----	23.7382		----			
8.9371	3210	0.020	----	23.8862		----			
9.0163	6002	0.018	1.019	23.9972	24:0	0.68	ECL deviates -0.003	Reference -0.006	
9.3816	4638	0.018	----	24.5094		----	> max rt		
9.4824	1864	0.016	----	24.6507		----	> max rt		

ECL Deviation: 0.005                            Reference ECL Shift: 0.007       Number Reference Peaks: 19
Total Response: 1076797                       Total Named: 881963
Percent Named: 81.91%                         Total Amount: 901343

(No search libraries specified in method PLFAD1.)
